# The C9orf72/SMCR8 complex maintains microglial homeostasis via RAB8A-ESCRT-mediated lysosomal repair

**DOI:** 10.1038/s44318-026-00817-w

**Published:** 2026-05-29

**Authors:** Shan Li, Shidong Xu, Feng Li, Qirui Zhao, Penghui Zhang, Qinghua Guan, Xiangxiang Sun, Jundong Bi, Hu Xiao, Yiyuli Tang, Cheng Peng, Qingfeng Chen, Yonghua Wang, Mei Yang

**Affiliations:** Center for Life Sciences, Yunnan Key Laboratory of Cell Metabolism and Diseases, School of Life Sciences, Yunnan University, Kunming, China

**Keywords:** Membranes & Trafficking, Neuroscience

## Abstract

Microglia are critical regulators of neuroinflammation and neurodegeneration. Haploinsufficiency of *C9orf72*, the most frequently mutated gene in amyotrophic lateral sclerosis and frontotemporal dementia, has been linked to autophagy-lysosomal pathway defects, but the role of C9orf72 in microglia remains unclear. Here, we identify the C9orf72/SMCR8 complex as a key regulator of microglial homeostasis through promoting lysosomal membrane repair. Loss of C9orf72 and SMCR8 in mice causes age‑dependent neuroinflammation and microgliosis, with microglia adopting a disease-associated state. In aged brain and spinal cord tissue, microglia display lysosomal damage marked by galectin‑3 accumulation. Using a lysosomotropic agent to induce lysosomal damage in microglia, we find that C9orf72/SMCR8-deficient cells accumulate damaged lysosomes and show defective recruitment of phosphorylated RAB8A and the Endosomal Sorting Complexes Required for Transport (ESCRT) machinery to damaged lysosomes. Notably, mutant microglia accumulate GTP‑bound RAB8A, which becomes hyperphosphorylated and mislocalized to RAB7-positive, LAMP1-negative vesicles. The GTPase-activating activity of the C9orf72/SMCR8 complex is essential for lysosomal repair. Our findings reveal that the C9orf72/SMCR8 complex coordinates RAB8A-ESCRT-mediated lysosomal repair to safeguard microglial homeostasis and limit neuroinflammation.

## Synopsis

Haploinsufficiency of *C9orf72* is linked to amyotrophic lateral sclerosis and frontotemporal dementia. This study shows that C9orf72 and its binding partner SMCR8 protect against neurodegeneration in old mice by promoting lysosomal membrane repair in microglia.

- C9orf72/SMCR8 deficiency causes age-dependent neurodegeneration, neuroinflammation, and induction of disease-associated microglial (DAM) state.
- In vivo and in vitro models demonstrate that C9orf72/SMCR8 deficiency leads to accumulation of damaged lysosomes.
- Loss of C9orf72/SMCR8 impairs ESCRT complex recruitment to damaged lysosomes, preventing membrane repair.
- C9orf72/SMCR8 GAP activity regulates RAB8A GTP loading, phosphorylation, and spatial localization during lysosomal damage responses.

## Abstract (web summary)

Loss of C9orf72/SMCR8 in microglia causes age-dependent neurodegeneration associated with lysosomal damage and microglial activation.

## Graphical abstract

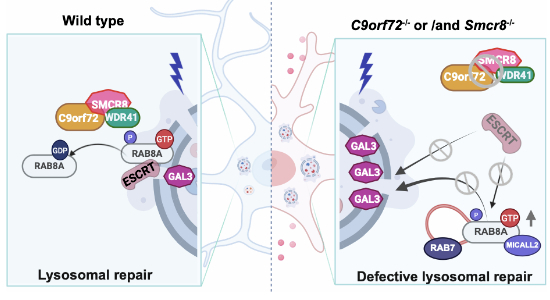

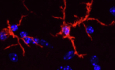

## Introduction

Microglial dysfunction represents a central pathogenic mechanism in neurodegenerative diseases, with aberrant activation states contributing to neuroinflammation and neuronal loss (Salter and Stevens, 2017). In neurodegenerative diseases, microglia adopt distinct activation states, including disease-associated microglia (DAM) and the microglial neurodegenerative phenotype (MGnD), characterized by altered gene expression profiles and enhanced phagocytic activity (Castro-Gomez and Heneka, 2024; Chen et al, 2024; Fumagalli et al, 2025; Keren-Shaul et al, 2017; Paolicelli et al, 2022). These activated microglia can contribute to neurodegeneration through excessive synaptic pruning, production of inflammatory mediators, and impaired clearance of protein aggregates (Heneka et al, 2025; Shi and Yong, 2025). Importantly, aging microglia exhibit compromised lysosomal function, characterized by accumulation of undegraded materials, elevated lysosomal pH, and increased susceptibility to membrane permeabilization. These age-related changes render microglia more prone to inflammatory activation and impair their neuroprotective functions (Quick et al, 2023).

Hexanucleotide repeat expansions in *C9orf72* are the most common genetic cause of amyotrophic lateral sclerosis (ALS) and frontotemporal dementia (FTD) (DeJesus-Hernandez et al, 2011; Dobson-Stone et al, 2012; Rademakers, 2012; Sha et al, 2012). In addition to toxic gain-of-function effects from repeat-associated non-ATG translation and RNA foci, C9orf72 haploinsufficiency has emerged as a critical driver of disease progression (Boivin et al, 2020; Shao et al, 2019; Zhu et al, 2020). C9orf72 forms a heterotrimeric complex with SMCR8 (Smith-Magenis syndrome chromosome region candidate 8) and WDR41 (WD repeat-containing protein 41), which regulates diverse cellular processes including autophagy, endolysosomal pathways, ciliogenesis, and membrane trafficking (Amick et al, 2016; Sellier et al, 2016; Shi et al, 2018; Su et al, 2020; Sullivan et al, 2016; Tang et al, 2020; Ugolino et al, 2016; Yang et al, 2016; Zhang et al, 2023; Zhang et al, 2018). Structural analyses have shown that this complex exhibits GTPase-activating protein (GAP) activity toward multiple small GTPases, including ARF GTPases, RAB8A, and RAB11A (Su et al, 2020; Tang et al, 2020; Tang et al, 2023).

Evidence from human iPSC-derived models, mouse genetics, and patient tissue consistently demonstrates that C9orf72, together with SMCR8, is essential for immune homeostasis particularly in myeloid cells. At the systemic level, C9orf72 or SMCR8 deficiency results in splenomegaly, lymphadenopathy, autoimmunity, and premature mortality, phenotypes linked to dysregulated mTORC1–AKT signaling, excessive lysosomal exocytosis, and prolonged TLR signaling (Lan et al, 2019; McAlpine et al, 2018; O’Rourke et al, 2016; Shao et al, 2020; Zhang et al, 2018). In myeloid cells (macrophages, monocytes, microglia, dendritic cells), loss of C9orf72 disrupts lysosomal function, impairs autophagy initiation, and triggers aberrant activation of STING-type I interferon and JAK/STAT signaling, while also enhancing antigen presentation and CD80 upregulation. (Burberry et al, 2020; Lall et al, 2021; Limone et al, 2024; McCauley et al, 2020; O’Rourke et al, 2016; Pang and Hu, 2023; Vahsen et al, 2023). In microglia, C9orf72 deficiency drives a transition from a homeostatic to inflammatory state, leading to excessive release of MMP9, abnormal synaptic pruning, and non–cell-autonomous neurotoxicity that contributes to cognitive deficits (Banerjee et al, 2023; Vahsen et al, 2023; Wang et al, 2022).

Although the inflammatory phenotypes of C9orf72/SMCR8-deficient microglia are well established, the upstream mechanisms remain incompletely understood. Lysosomal dysfunction has emerged as a convergent driver of age-related and neurodegenerative processes (Ferrari et al, 2024). In aging microglia, lysosomes accumulate undegradable substances including lipofuscin, oxidized proteins, and iron, leading to elevated lysosomal pH, membrane permeabilization, and inflammatory activation (Bussi et al, 2018; Quick et al, 2023). Upon lysosomal membrane permeabilization, cells engage quality control pathways, including galectin sensing, Endosomal Sorting Complexes Required for Transport (ESCRT)-mediated repair, lysophagy, and additional mechanisms such as ER-mediated lipid transfer, sphingomyelinase-driven ceramide generation, annexin recruitment, and stress granule stabilization (Radulovic et al, 2025). Studies in macrophages revealed that leucine-rich repeat kinase 2 (LRRK2)-mediated phosphorylation of RAB8A at threonine 72 (pT72-RAB8A) is critical for recruiting ESCRT components to damaged lysosomes and facilitating membrane repair (Eguchi et al, 2018; Herbst et al, 2020). Despite these advances, the precise molecular mechanisms by which C9orf72/SMCR8 deficiency leads to microglial dysfunction remain largely unknown. Given the emerging role of lysosomal membrane damage and repair in microglial aging, we hypothesized that the C9orf72/SMCR8 complex regulates microglial homeostasis through control of lysosomal repair pathways.

Here, using knockout mouse models and cellular systems, we demonstrate that loss of the C9orf72/SMCR8 complex results in age-dependent microglial activation, accumulation of damaged lysosomes marked by GAL3, and progressive neuroinflammation. Mechanistically, we reveal that C9orf72/SMCR8 deficiency disrupts RAB8A GTPase cycling and phosphorylation dynamics, impairing ESCRT recruitment to damaged lysosomes. Loss of GAP activity causes GTP-RAB8A accumulation and hyperphosphorylation, leading to mistargeting to RAB7-positive and LAMP1-negative vesicles and preventing ESCRT-mediated membrane repair. These findings establish a previously unrecognized role of the C9orf72/SMCR8 complex in microglial lysosomal quality control and provide mechanistic insight into how its loss contributes to neuroinflammation in ALS/FTD.

## Results

### Loss of *C9orf72* and *Smcr8* leads to age-dependent neuroinflammation and microgliosis in mice

**Figure EV1:**
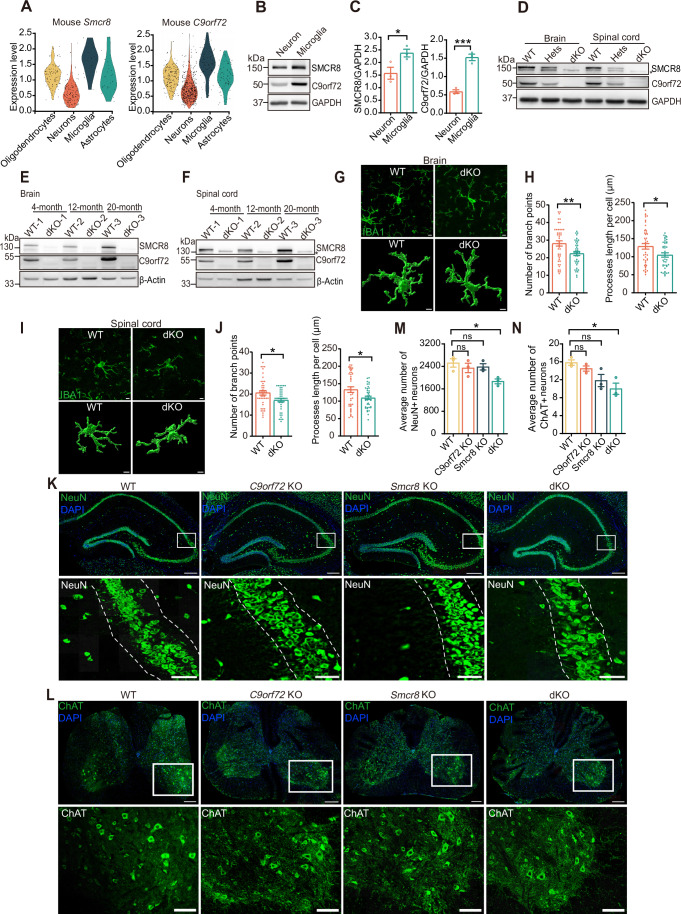
C9orf72 and SMCR8 are highly expressed in microglia and exhibit age-dependent upregulation in the CNS. (**A**) Violin plots showing *C9orf72* and *Smcr8* expression levels across major CNS cell types from single-cell RNA-sequencing dataset (Yao et al, 2021; *n* = 54,390 cells). (**B**, **C**) Immunoblotting analysis of C9orf72 and SMCR8 protein levels in primary neurons and microglia (**B**). GAPDH serves as loading control. Quantification of protein levels normalized to GAPDH is shown in (**C**) (*n* = 3 independent experiments, SMCR8: *P* = 0.0472, C9orf72: *P* = 0.0008). (**D**) Validation of knockout efficiency in C9orf72/SMCR8 dKO mice by Western blot analysis of brain and spinal cord tissue lysates. Representative blots show protein expression in WT, heterozygous (Het), and dKO genotypes. GAPDH serves as loading control. Asterisks indicate nonspecific bands. (**E**, **F**) Age-dependent expression of SMCR8 and C9orf72 in brain (**E**) and spinal cord (**F**) from WT mice at 4, 12, and 20 months of age. β-Actin serves as loading control. Asterisks indicate nonspecific bands. (**G**–**J**) Representative immunofluorescence images of IBA1 (green) in brain (**G**) and spinal cord (**I**) sections from 20-month-old WT and dKO mice, with three-dimensional surface reconstructions of microglia (Imaris Surface module). Scale bar, 5  μm. Quantitative analysis of microglial branch number (branch points) and process length in brain (**H**: branch points, *P* = 0.0038; process length, *P* = 0.0192) and spinal cord (**J**: branch points, *P* = 0.0125; process length, *P* = 0.0147) using Imaris Filament module (*n* = 40–45 cells per genotype from three independent experiments). (**K**–**N**) Representative confocal images of NeuN immunostaining (green) and DAPI (blue) in the hippocampus (**K**), and ChAT immunostaining (green) and DAPI (blue) in the spinal cord (**L**) of 12-month-old WT, *C9orf72* KO, *Smcr8* KO, and dKO mice, with quantification of NeuN-positive neurons in the hippocampus (**M**) and ChAT-positive motor neurons in the spinal cord (**N**). Boxed areas are enlarged below; white dotted lines in (**K**) indicate part of the CA3 subregion. Scale bars, 200 μm (main), 50 μm (**K**, insets), 100 μm (**L**, insets). *n* = 3 mice per group. Hippocampus (**M**): WT vs *C9orf72* KO: *P* = 0.4718; WT vs *Smcr8* KO: *P* = 0.5031; WT vs dKO: *P* = 0.0194. Spinal cord (**N**): WT vs *C9orf72* KO: *P* = 0.1848; WT vs *Smcr8* KO: *P* = 0.055; WT vs dKO: *P* = 0.0139. Data information: In (**C**, **H**, **J**, **M**, **N**), data are presented as mean ± SEM from three independent experiments. Statistical significance was assessed by unpaired two-tailed Student’s *t* test. *P < 0.05, **P < 0.01; ns, not significant.

To investigate the role of C9orf72 and SMCR8 in microglia, we first examined the expression levels of these proteins in various cell types of the central nervous system (CNS). Analysis of single cell RNA-seq data from the *Whole Cortex & Hippocampus – 10x genomics dataset* (Yao et al, 2021) revealed that C9orf72 and SMCR8 are highly expressed in microglia compared to other CNS cell types (Fig. EV1A). Western blot analysis demonstrated differential expression of C9orf72 and SMCR8, with higher protein levels detected in primary microglia compared to neurons isolated from wild-type mice (Fig. EV1B,C). Western blot analysis across age groups showed a modest increase in C9orf72 and SMCR8 protein levels in 20-month-old mice compared to 4- and 12-month-old animals in the brain and spinal cord (Fig. EV1E,F). This age-dependent upregulation suggests that the C9orf72-SMCR8 complex may play an important role in maintaining microglial homeostasis during aging.

**Figure 1:**
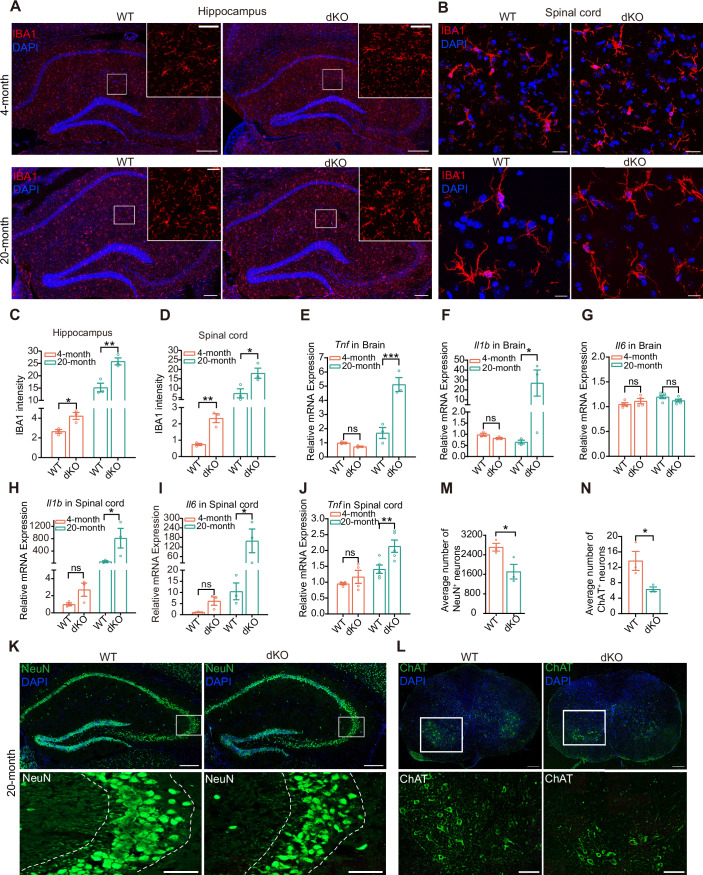
*C9orf72*/*Smcr8* double knockout mice develop age-dependent neuroinflammation and neurodegeneration. (**A**, **B**) Representative immunofluorescence images of IBA1 (red) and DAPI (blue) in hippocampus (**A**) and spinal cord (**B**) from WT and *C9orf72/Smcr8* dKO mice at 4 and 20 months of age. Scale bars, 200 μm (hippocampus), 20 μm (spinal cord). Insets show higher magnification; scale bar, 10 μm. (**C**, **D**) Quantification of IBA1 fluorescence intensity in hippocampus (**C**) and spinal cord (D) (*n* = 3 mice per group). Hippocampus: *P* = 0.0291 (4-month), *P* = 0.0091 (20-month). Spinal cord: *P* = 0.0047 (4-month), *P* = 0.0416 (20-month). (**E**–**J**) qRT-PCR analysis of cytokine mRNA expression in brain (*Tnf*, *Il1b*, *Il6*; **E**–**G**) and spinal cord (*Il1b*, *Il6*, *Tnf*; **H**–**J**) at 4 and 20 months (*n* = 3–5 mice per group). Data are normalized to *Actb* expression. *Tnf* (**E**): *P* = 0.8189 (4-month), *P* = 0.0001 (20-month); *Il1b*(F): *P* = 0.9869 (4-month), *P* = 0.0223 (20-month). *Il6* (**G**): *P* = 0.4668 (4-month), *P* = 0.3055 (20-month). *Il1b* (**H**): *P* = 0.9999 (4-month), *P* = 0.0196 (20-month). *Il6* (I): *P* = 0.9944 (4-month), *P* = 0.0454 (20-month). *Tnf* (J): *P* = 0.3777 (4-month), *P* = 0.0044 (20-month). (**K**, **L**) Representative confocal images showing NeuN (green) in the hippocampus (**K**), and ChAT (green) in the spinal cord (**L**) of 20-month-old mice. Nuclei were stained with DAPI (blue). Boxed areas are enlarged below. White dotted lines indicate part of the cornu ammonis 3 (CA3) subregion in (**K**). Scale bars, 200 μm (main), 50 μm (hippocampus inset), 100 μm (spinal cord inset). (**M**, **N**) Quantification of NeuN-positive neurons in hippocampus (**M**) and ChAT-positive motor neurons in spinal cord (**N**) (*n* = 3 mice per group). Hippocampus (**M**): *P* = 0.0418 (20-month). Spinal cord (**N**): *P* = 0.0435 (20-month). Data information: In (**C**–**J**, **M**, **N**), data are presented as mean ± SEM from three independent experiments. Statistical significance was assessed by unpaired two-tailed Student’s *t* test (**C**, **D**, **M**, **N**) or two-way ANOVA with Bonferroni post-hoc test (**E**–**J**). *P < 0.05, **P < 0.01, ***P < 0.001; ns, not significant.

To elucidate the functional consequences of *C9orf72* and *Smcr8* loss, we generated double knockout (dKO) mice (Fig. EV1D). Immunofluorescence analysis revealed significantly increased IBA1-positive activated microglia in the hippocampus (Fig. 1A,C) and spinal cord (Fig. 1B,D) of dKO mice compared to age-matched WT. This microgliosis phenotype intensified with age, suggesting an age-dependent effect of C9orf72 and SMCR8 deficiency. Morphological analysis of microglia revealed that dKO microglia exhibited reduced ramification, with decreased branch numbers (branch points) and shorter process lengths in both brain (Fig. EV1G,H) and spinal cord (Fig. EV1I,J), indicating a shift toward reactive microglia. To further investigate the inflammatory status of dKO mice, we measured the mRNA levels of pro-inflammatory cytokines in the brain and spinal cord of 4-month-old and 20-month-old mice. We observed significant upregulation of *Tnf* and *Il1b* in the brain, and *Tnf*, *Il1b* and *Il6* in the spinal cord of dKO mice compared to WT controls (Fig. 1E–J). These differences were predominantly observed in 20-month-old aged mice, with the inflammatory response being more pronounced in the spinal cord. Immunofluorescence staining for neuronal markers revealed significantly decreased numbers of NeuN- and ChAT-positive neurons in both hippocampus and spinal cord of 20-month-old dKO mice compared to age-matched WT mice, indicating age-dependent neurodegeneration (Fig. 1K–N). To assess whether individual gene deletion contributes to this phenotype, we analyzed 12-month-old WT, single knockout and dKO mice. Single knockouts showed a trend toward reduced ChAT-positive neurons, though this did not reach statistical significance. dKO mice exhibited significant reductions in NeuN-positive and ChAT-positive neurons (Fig. EV1K–N), demonstrating that while C9orf72 and SMCR8 are mutually dependent for complex stability (Amick et al, 2016; Lan et al, 2019), simultaneous loss of both proteins is required to drive significant neuronal loss. These results demonstrate that loss of *C9orf72* and *Smcr8* leads to age-dependent neuroinflammation, microgliosis and neurodegeneration.

**Figure 2:**
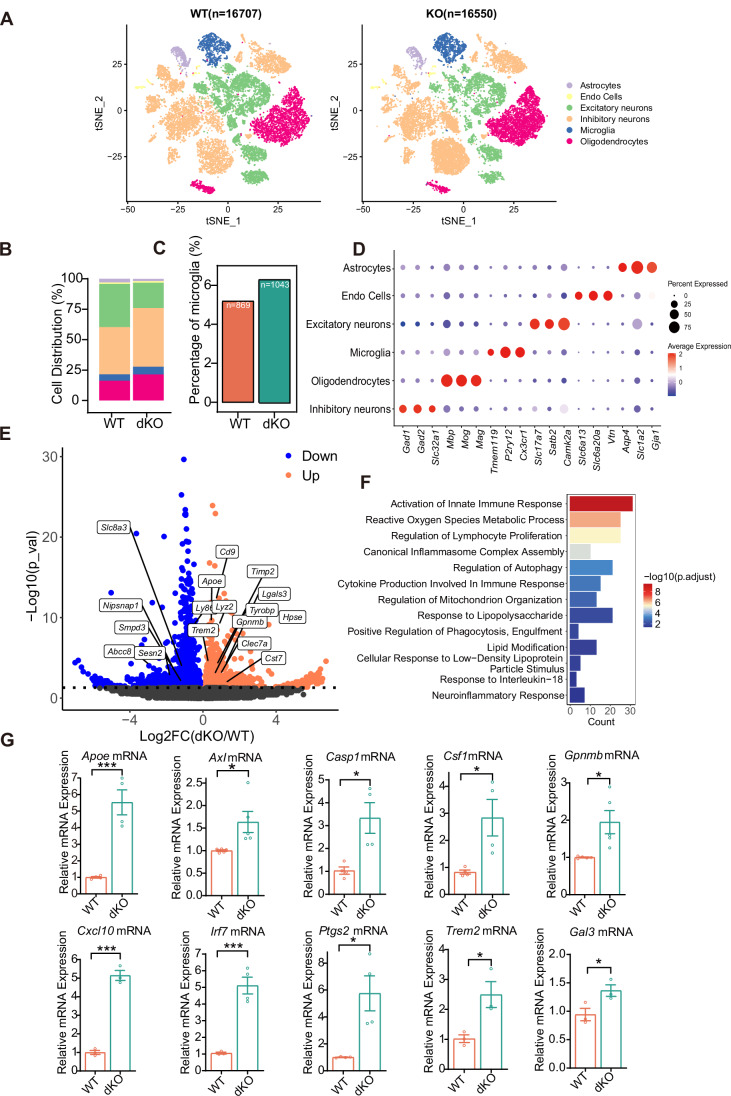
snRNA-seq and primary microglia qRT-PCR reveal disease-associated microglia (DAM) activation in dKO mice. (**A**) UMAP of 33,257 single‑nucleus transcriptomes from brains of 20- to 24‑month-old WT and dKO mice (WT: *n* = 16,707 nuclei; dKO: *n* = 16,550 nuclei). Major CNS cell types were annotated by canonical marker gene expression and are color-coded as indicated. (**B**) Stacked bar plot showing cell-type composition in WT and dKO samples. Bars are colored by their corresponding cell classes as labeled in (**A**). (**C**) Comparison of the proportion of microglial cells among total cells in WT and dKO samples (WT: 5.2%; dKO: 6.3%). (**D**) Dot plot showing expression of selected cell-type-specific marker genes across identified cell populations. Circle size indicates percentage of expressing cells; color intensity represents average normalized expression level. (**E**) Volcano plot showing DEGs in dKO versus WT microglia (WT: *n *= 869 nuclei; dKO: *n* = 1043 nuclei). DEGs were identified using the FindMarkers function from the Seurat package with the default Wilcoxon rank-sum test and a log_2_ fold-change threshold of 0.1. Orange dots, upregulated genes; blue dots, downregulated genes. Key DAM markers are labeled. (**F**) GO enrichment of biological processes for upregulated genes in dKO microglia. GO enrichment analysis was performed using the clusterProfiler package with the default hypergeometric test. Color scale represents −log10 (adjusted *P* value). (**G**) qRT‑PCR validation of DAM-associated genes (*Apoe, Axl, Casp1, Csf1, Gpnmb, Cxcl10, Irf7, Ptgs2, Trem2, Gal3)* in primary microglia from WT and dKO mice (*n* = 3–5 mice per group). Data are normalized to *Actb* expression. *Apoe*: *P* = 0.0009; *Axl*: *P* = 0.0273; *Casp1*: *P* = 0.0156; *Csf1*: *P* = 0.0252; *Gpnmb*: *P* = 0.0163; *Cxcl10*: *P* = 0.0001; *Irf7*: *P* = 0.0002; *Ptgs2*: *P* = 0.0105; *Trem2*: *P* = 0.0309; *Gal3*: *P* = 0.0472. Data information: In (**G**), data are presented as mean ± SEM from three independent experiments. Statistical significance was assessed by unpaired two-tailed Student’s *t* test. *P < 0.05, ***P < 0.001. Source data are available online for this figure.

Recent studies have identified distinct microglial phenotypes in neurodegenerative conditions, including DAM and MGnD, which share overlapping characteristics. Both phenotypes share common molecular and functional characteristics, suggesting they may represent overlapping states of microglial activation in response to pathological conditions. To gain deeper insights into the microglial response, we performed single-nucleus RNA sequencing (snRNA-seq) on brains from aged (20- to 24-month-old) WT and dKO mice. After stringent quality control including doublet removal, we obtained 33,257 high-quality single-cell transcriptomes (WT: *n* = 16,707 cells; dKO: *n* = 16,550 cells) that were annotated into major cell types based on cell-type-specific markers (Fig. 2A). UMAP visualization demonstrated distinct clustering of major CNS cell types including neurons, astrocytes, oligodendrocytes, and microglia (Fig. 2A,D). Cell-type composition analysis revealed an expanding trend in the microglial population in dKO mice compared to WT controls (Fig. 2B,C). Further analysis identified differentially expressed genes (DEGs) in dKO microglia compared to WT, with significant upregulation of DAM/MGnD markers including *Apoe*, *Gpnmb*, *Lgals3(gal3)*, *Trem2* and *Clec7a* (Fig. 2E). Gene Ontology (GO) analysis of upregulated genes revealed enrichment of pathways involved in innate immune response, reactive oxygen species metabolic processes, regulation of lymphocyte proliferation, canonical inflammasome complex assembly, and cytokine production (Fig. 2F). Consistent with the snRNA-seq data, qPCR analysis of isolated primary microglia confirmed significant upregulation of these DAM-associated genes in dKO mice (Fig. 2G). This molecular signature suggests that loss of *C9orf72* and *Smcr8* leads to acquisition of a disease-associated microglial phenotype characterized by enhanced inflammatory responses and altered cellular homeostasis.

### Age-dependent inflammation correlates with increased levels of Galectin-3

**Figure 3:**
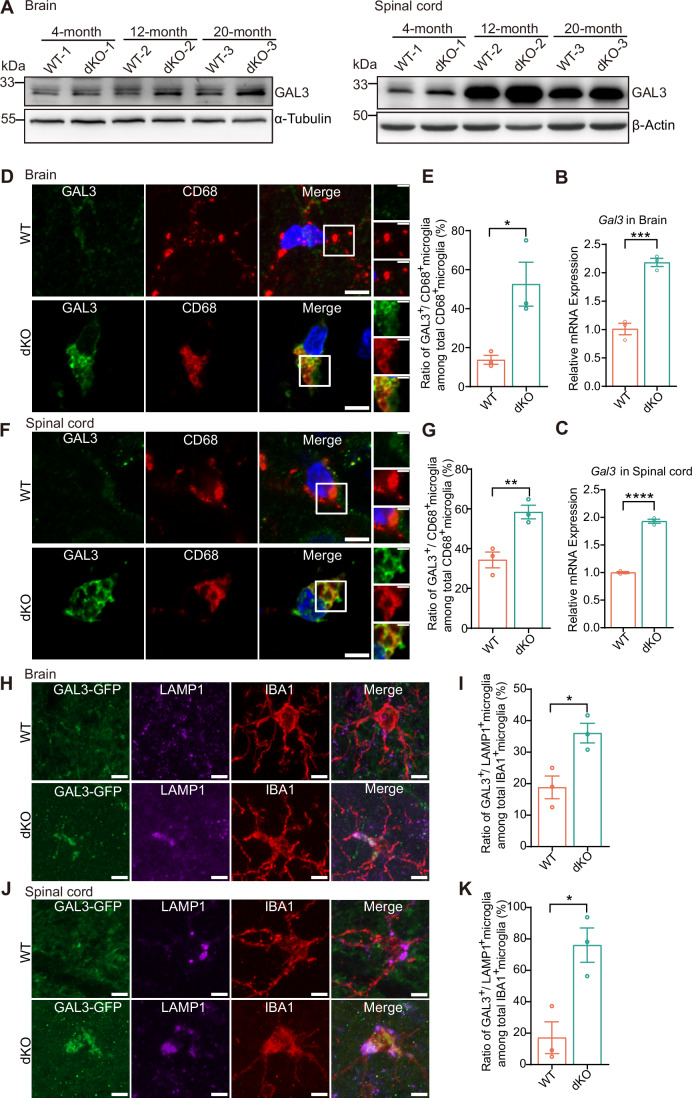
Age-dependent GAL3 upregulation and enhanced lysosomal localization in *C9orf72/Smcr8* dKO mice. (**A**) Western blot analysis of GAL3 protein levels in brain and spinal cord tissue at 4, 12, and 20 months. α-Tubulin and β-actin serve as loading controls. (**B**, **C**) qRT-PCR analysis of *Gal3* mRNA in brain (**B**) and spinal cord (**C**) from 20-month-old mice (*n* = 3 mice per group). Data are normalized to *Actb* expression. Brain (**B**): *P* = 0.0007. Spinal cord (**C**): *P* < 0.0001. (**D**, **E**) Representative immunofluorescence images of GAL3 (green), CD68 (red), and DAPI (blue) in the brain cortex of 20-month-old mice (**D**), and quantification of the percentage of GAL3⁺/CD68⁺ microglia among total CD68⁺ microglia (**E**). Boxed regions are magnified in the right panels. Scale bars, 5 μm (main), 2 μm (insets). *P* = 0.0278. (**F**, **G**) Representative immunofluorescence images of GAL3 (green), CD68 (red), and DAPI (blue) in the spinal cord of 20-month-old mice (**F**), and quantification of the percentage of GAL3⁺/CD68⁺ microglia among total CD68⁺ microglia (**G**). Boxed regions are magnified in the right panels. Scale bars, 5 μm (main), 2 μm (insets). *P* = 0.01. (**H**, **I**) Representative confocal images of GAL3-GFP (green), LAMP1 (magenta), and IBA1 (red) in the brain cortex of 20-month-old mice following AAV-PHP.eB-GAL3-GFP delivery (**H**), and quantification of the percentage of GAL3-GFP⁺/LAMP1⁺ microglia among total IBA1⁺ microglia (**I**). Scale bars, 5 μm. *P* = 0.0227. (**J**, **K**) Representative confocal images of GAL3-GFP (green), LAMP1 (magenta), and IBA1 (red) in the spinal cord of 20-month-old mice following AAV-PHP.eB-GAL3-GFP delivery (**J**), and quantification of the percentage of GAL3-GFP⁺/LAMP1⁺ microglia among total IBA1⁺ microglia (**K**). Scale bars, 5 μm. *P* = 0.0166. Data information: In (**B**, **C**, **E**, **G**, **I**, **K**), data are presented as mean ± SEM (*n* = 3 mice per group; for (**E**, **G**, **I**, **K**), 3–5 fields from three sections per mouse were analyzed). Statistical significance was assessed by unpaired two-tailed Student’s *t* test. *P < 0.05, **P < 0.01, ***P < 0.001, ****P < 0.0001. Source data are available online for this figure.

**Figure EV2:**
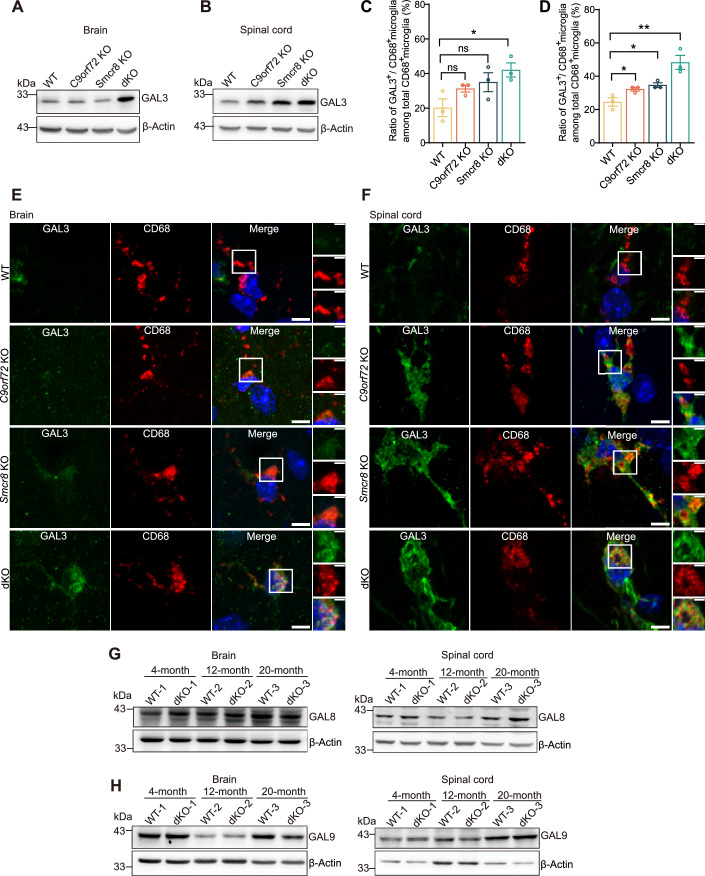
GAL3 accumulation predominantly occurs in *C9orf72/Smcr8* double knockout mice. (**A**, **B**) Immunoblotting analysis of GAL3 protein levels in brain (**A**) and spinal cord (**B**) tissue from 12-month-old WT, *C9orf72* KO, *Smcr8* KO, and dKO mice. β-Actin serves as loading control. (**C**–**F**) Representative immunofluorescence images of GAL3 (green), CD68 (red), and DAPI (blue) in brain cortex (**E**) and spinal cord (**F**) sections from 12-month-old WT, *C9orf72* KO, *Smcr8* KO, and dKO mice. Boxed areas are enlarged on the right showing individual channels and merged images. Scale bars, 5 μm (main), 2 μm (insets). Quantification of GAL3-positive puncta in CD68-positive microglia in brain cortex (**C**) and spinal cord (**D**) (*n* = 3 mice per genotype; 3–5 fields from 3 sections per mouse analyzed). Brain: WT vs *C9* KO: *P* = 0.1158, WT vs *S8* KO: *P* = 0.1196, WT vs dKO: *P* = 0.0291; Spinal cord: WT vs *C9* KO: *P* = 0.0475, WT vs *S8* KO: *P* = 0.0231, WT vs dKO: *P* = 0.0082. (**G**, **H**) Age-dependent expression of GAL8 (**G**) and GAL9 (**H**) in brain and spinal cord tissue from WT and dKO mice at 4, 12, and 20 months of age. β-Actin serves as loading control. Data information: In (**C**, **D**), data are presented as mean ± SEM from three independent experiments. Statistical significance was assessed by unpaired two-tailed Student’s *t* test. *P < 0.05, **P < 0.01; ns, not significant. Source data are available online for this figure.

Our snRNA-seq analysis and primary microglia revealed significant upregulation of Galectin-3 (*Gal3*) mRNA expression in microglia from dKO mice, prompting us to investigate the role of GAL3 in the observed neuroinflammatory phenotype. Given that GAL3 serves as a key marker for lysosomal dysfunction and microglial inflammatory responses (Jia et al, 2020; Shan et al, 2024; Siew et al, 2019), several studies indicate lysosome repair linked to neurodegeneration (Chou et al, 2025; Radulovic et al, 2025). Western blot analysis revealed a progressive age-dependent increase in GAL3 protein levels in brain and spinal cord of dKO mice compared to WT controls (Fig. 3A). Consistent with the protein data, qRT-PCR analysis confirmed significant elevation of *Gal3* mRNA levels in both brain and spinal cord of 20-month-old dKO mice (Fig. 3B,C). To visualize GAL3 localization and assess its relationship with microglial activation, we performed immunofluorescence staining using GAL3 antibodies on cortical and spinal cord sections from 20-month-old WT and dKO mice. We observed a higher proportion of GAL3-positive microglia in dKO mice, with strong colocalization between GAL3 and CD68, a lysosomal marker of activated microglia (Fig. 3D–G). To further characterize GAL3 subcellular distribution and its association with lysosomes, we administered AAV-PHP.eB-GAL3-GFP reporter vectors via tail vein injection to achieve broad CNS expression. Remarkably, dKO mice exhibited increased GAL3-GFP puncta in IBA1-positive microglia, with colocalization with the lysosomal marker LAMP1 (Fig. 3H,J). Quantification revealed a significantly higher percentage of microglia containing GAL3^+^LAMP1^+^ lysosomes in dKO mice compared to WT, in both brain and spinal cord (Fig. 3I,K), suggesting accumulation of damaged lysosomes in mutant microglia. Given that C9orf72 and SMCR8 are mutually dependent for protein stability (Amick et al, 2016; Lan et al, 2019), we examined whether loss of individual proteins was sufficient to induce GAL3 upregulation. Analysis of 12-month-old mice revealed that while single KO mice showed modest GAL3 elevation compared to WT in spinal cord, dKO mice displayed significantly greater GAL3 upregulation in both brain and spinal cord (Fig. EV2A,B). Consistent with this pattern, GAL3^+^CD68^+^ lysosomal puncta were significantly increased in dKO microglia compared to WT in both brain and spinal cord, whereas single KO mice showed intermediate phenotypes with regional differences: brain tissue from single KO mice was comparable to WT, while spinal cord showed a modest but significant increase that was less pronounced than in dKO mice (Fig. EV2C–F). Notably, other galectin family members, GAL8 and GAL9, showed no significant differences between dKO and WT mice (Fig. EV2G,H), indicating specificity of the GAL3 response. Collectively, these results demonstrate that the age-dependent neuroinflammation observed in dKO mice correlates with selective upregulation of GAL3 and accumulation of damaged lysosomes in microglia, suggesting a critical role for the C9orf72-SMCR8 complex in maintaining lysosomal homeostasis.

### C9orf72/SMCR8 deficiency enhances stress-induced inflammatory and phagocytic responses in microglia

**Figure 4:**
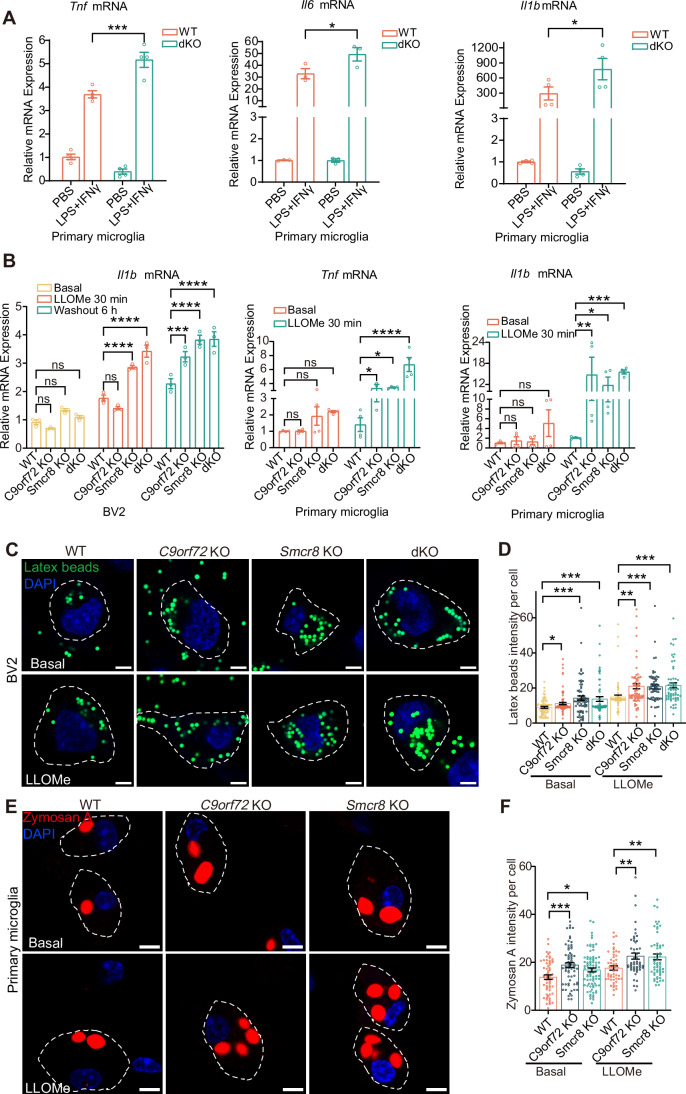
*C9orf72/Smcr8* deficiency enhances stress-induced microglial hyperactivation and enhanced phagocytosis. (**A**) qRT‑PCR analysis of *Tnf*, *Il6*, and *Il1b* mRNA expression in primary microglia from WT and dKO under basal conditions or LPS (100 ng/mL)/IFN‑γ (50 ng/mL) stimulation for 24 h (*n* = 3–4 mice per group). Data are normalized to *Actb* expression. LPS/IFN-γ stimulation - *Tnf*: *P* = 0.0004, *Il6*: *P* = 0.0227, *Il1b*: *P* = 0.034. (**B**) Cytokine mRNA expression following LLOMe-induced lysosomal damage. BV2 cells (WT, *C9orf72* KO, *Smcr8* KO, dKO): basal, 30 min LLOMe (1 mM), 6 h washout. Primary microglia (WT, *C9orf72* KO, *Smcr8* KO, dKO): basal, 30 min LLOMe (0.5 mM) (*n* = 3–4 biological replicates per group). Data are normalized to *Actb* expression. BV2 cells - 30 min LLOMe: WT vs *C9orf72* KO (*C9* KO) *P* = 0.1924, WT vs *Smcr8* KO (*S8* KO) *P* < 0.0001, WT vs dKO *P* < 0.0001; 6 h washout: WT vs *C9* KO *P* = 0.0002, WT vs *S8* KO *P* < 0.0001, WT vs dKO *P* < 0.0001. Primary microglia - 30 min LLOMe: *Tnf* WT vs *C9* KO *P* = 0.0341, WT vs *S8* KO *P* = 0.0138, WT vs dKO *P* < 0.0001; Primary microglia - 30 min LLOMe: *Il1b* WT vs *C9* KO *P* = 0.0014, WT vs *S8* KO *P* = 0.0147, WT vs dKO *P* = 0.0007. (**C**) Representative confocal images of latex bead phagocytosis in BV2 cells. Internalized beads (green) and nuclei (DAPI, blue) are shown. Cell boundaries are outlined with dashed lines. Scale bar, 5 μm. (**D**) Quantification of internalized bead fluorescence intensity per cell (*n* = 60 - 89 cells per genotype per condition, from three independent experiments). Basal: WT vs *C9* KO *P* = 0.0133, WT vs *S8* KO *P* = 0.0002, WT vs dKO *P* = 0.0009. LLOMe: WT vs *C9* KO *P* = 0.001, WT vs *S8* KO *P* = 0.0002, WT vs dKO *P* = 0.0002. (**E**) Representative confocal images of zymosan A bioparticle uptake in primary microglia. Internalized particles (red) and nuclei (DAPI, blue) are shown. Cell boundaries are outlined with dashed lines. Scale bar, 5 μm. (**F**) Quantification of internalized particle fluorescence intensity per cell (*n* = 50–81 cells per genotype per condition, from three independent experiments). Basal: WT vs *C9* KO *P* = 0.0003, WT vs *S8* KO *P* = 0.0176. LLOMe: WT vs *C9* KO *P* = 0.0018, WT vs *S8* KO *P* = 0.0031. Data information: In (**A**, **B**, **D**, **F**), data are presented as mean ± SEM. Statistical significance was assessed by two-way ANOVA with Bonferroni post-hoc test (**A**, **B**) or unpaired two-tailed Student’s *t* test (**D**, **F**). *P < 0.05, **P < 0.01, ***P < 0.001, ****P < 0.0001; ns, not significant. Source data are available online for this figure.

**Figure EV3:**
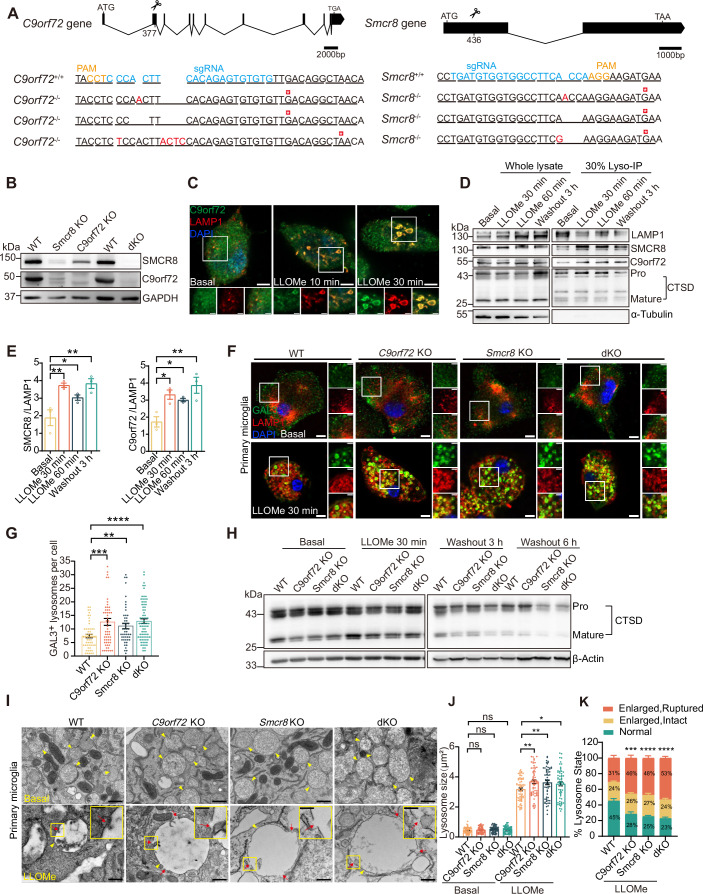
C9orf72 and SMCR8 are critical for maintaining lysosomal membrane integrity and repair. (**A**) Schematic of *C9orf72* and *Smcr8* gene structures showing CRISPR/Cas9 targeting strategy. Representative sequencing results from knockout clones showing insertions/deletions at target sites leading to frameshift mutations and premature stop codons. Blue indicates sgRNA target sites, orange indicates PAM sequences, and asterisks mark stop codons. Scale bars, 2000 bp (*C9orf72*), 1000 bp (*Smcr8*). (**B**) Immunoblotting analysis confirming knockout efficiency in generated BV2 cell lines. SMCR8, C9orf72, and GAPDH protein levels were assessed in WT, *Smcr8* KO, *C9orf72* KO, and dKO cells. GAPDH serves as loading control. (**C**) Representative immunofluorescence images of endogenous C9orf72 (green) and LAMP1 (red) in microglia under basal conditions and after LLOMe treatment (1 mM) for 10 and 30 min. Boxed areas are enlarged below showing colocalization. Scale bars, 5 μm (main), 2 μm (insets). (**D**, **E**) LysoIP analysis showing association of endogenous C9orf72 and SMCR8 with lysosomes under basal conditions, after LLOMe treatment (1 mM, 30 and 60 min), and following 3 h washout (**D**). Input and LAMP1-IP fractions were analyzed by immunoblotting. Quantification of SMCR8/LAMP1 and C9orf72/LAMP1 ratios in IP fractions is shown in (**E**) (*n* = 3 independent experiments). SMCR8: LLOMe 30 min vs Basal, *P* = 0.0042; LLOMe 60 min vs Basal, *P* = 0.05; Washout 3 h vs Basal, *P* = 0.0031; C9orf72: LLOMe 30 min vs Basal, *P* = 0.0174; LLOMe 60 min vs Basal, *P* = 0.0491; Washout 3 h vs Basal, *P* = 0.0033). (**F**, **G**) Representative immunofluorescence images of GAL3 (green), LAMP1 (red), and DAPI (blue) in WT, *C9orf72* KO, *Smcr8* KO, and dKO primary microglia under basal conditions and after LLOMe treatment (0.5 mM, 30 min) (**F**). Boxed areas are enlarged on the right. Scale bar, 5 μm. Quantification of GAL3-positive lysosomes per cell is shown in (**G**). Each dot represents an individual cell (*n* = 51–78 cells per genotype from three independent experiments). WT vs *C9* KO, *P* = 0.0005; WT vs *S8* KO, *P* = 0.0019; WT vs dKO, *P* < 0.0001. (**H**) Immunoblotting analysis of cathepsin D (CTSD) processing in WT, *C9orf72* KO, *Smcr8* KO, and dKO BV2 cells under basal conditions, after LLOMe treatment (1 mM, 30 min), and during washout periods (3 and 6 h). Pro-CTSD and mature CTSD are indicated. β-Actin serves as loading control. (**I**–**K**) TEM images of primary microglia from WT, *C9orf72* KO, *Smcr8* KO, and dKO mice under basal conditions (upper row) and after LLOMe treatment (1 mM, 30 min; lower row) (**I**). Yellow arrowheads indicate lysosomes. Red arrowheads indicate sites of lysosomal membrane rupture (membrane discontinuity). Yellow boxed areas showing ruptured lysosomes are enlarged in the upper right corner of each panel. Scale bars, 500 nm (main), 200 nm (insets). Quantification of lysosome area is shown in (**J**). Each dot represents a single lysosome (*n* = 58–64 lysosomes per genotype from three independent experiments). *P* values - LLOMe: WT vs *C9* KO, *P* = 0.0015; WT vs *S8* KO, *P* = 0.0075; WT vs dKO, *P* =0.0272 . Percentage distribution of lysosome states following LLOMe treatment is shown in (**K**). Normal (green, area ≤3 μm²); enlarged with intact membrane (yellow, area >3  μm²); enlarged with ruptured membrane (orange, area >3 μm² with membrane discontinuity) (*n* = 21–23 cells per genotype from three independent experiments). WT vs *C9* KO, *P* = 0.0002; WT vs *S8* KO, *P* < 0.0001; WT vs dKO, *P* < 0.0001. Data information: In (**E**, **G**, **J**, **K**), data are presented as mean ± SEM from three independent experiments. Statistical significance was assessed by one-way ANOVA with Tukey’s post-hoc test (**E**) or unpaired two-tailed Student’s *t* test (**G**, **J**, **K**). *P < 0.05, **P < 0.01, ***P < 0.001, ****P < 0.0001; ns, not significant. Source data are available online for this figure.

To investigate how C9orf72/SMCR8 deficiency affects microglial function, we isolated primary microglia from WT and KO mice and examined their inflammatory responses. Under basal conditions, primary microglia from dKO mice showed *Tnf*, *Il6*, and *Il1β* levels comparable to those in WT mice. Upon lipopolysaccharide (LPS) and interferon‑γ (IFN‑γ) stimulation, which promotes classical microglial activation, dKO primary microglia exhibited significantly elevated expression of these pro-inflammatory cytokines (Fig. 4A), consistent with previous findings that C9orf72 deficiency leads to enhanced pro-inflammatory responses in myeloid cells (Lall et al, 2021; Limone et al, 2024; Pang and Hu, 2023). Given the accumulation of GAL3-positive damaged lysosomes observed in dKO microglia in vivo, we hypothesized that C9orf72/SMCR8 deficiency might also render microglia hypersensitive to lysosomal stress. To test this, we generated *C9orf72* and *Smcr8* single and double knockout BV2 microglial cell lines using CRISPR-Cas9 technology (Fig. EV3A,B) and challenged them with L-leucyl-L-leucine methyl ester (LLOMe), which induces acute lysosomal membrane permeabilization. Following 30-min treatment with 1 mM LLOMe, *C9orf72* KO cells displayed comparable *Il1b* expression to WT, whereas *Smcr8* KO and dKO cells showed significantly elevated *Il1b* mRNA levels (Fig. 4A). After a 6-h washout period, all KO lines exhibited significantly enhanced *Il1b* expression compared to WT (Fig. 4B), indicating sustained inflammatory activation following lysosomal damage. We validated these findings in primary microglia, which demonstrated markedly greater sensitivity to lysosomal damage than BV2 cells. In primary microglia, both single and double knockout cells displayed significantly increased *Tnf* and *Il1b* expression after 30-min LLOMe treatment (Fig. 4B), confirming enhanced inflammatory responses upon lysosomal damage. This heightened inflammatory response suggests a critical role for C9orf72 and SMCR8 in maintaining lysosomal integrity and regulating inflammatory signaling pathways upon lysosomal damage.

Phagocytosis is a key microglial function that enables clearance of apoptotic cells, protein aggregates, and cellular debris from the CNS. Using latex beads uptake assays in BV2 cells, we found that both single and double knockout cells exhibited increased phagocytic activity under basal conditions, with further enhancement following 1 mM LLOMe treatment (Fig. 4C,D). Similarly, when assessing primary microglia using zymosan A particles, both *C9orf72* KO and *Smcr8* KO cells showed enhanced phagocytic activity at baseline, with further increases observed after 0.5 mM LLOMe treatment (Fig. 4E,F). These results demonstrate that loss of either *C9orf72* or *Smcr8* leads to dysregulated microglial responses, particularly under stress conditions, suggesting their cooperative role in maintaining proper microglial function.

### *C9orf72/Smcr8* loss of function exacerbates lysosomal damage and impairs recovery following LLOMe treatment

**Figure 5:**
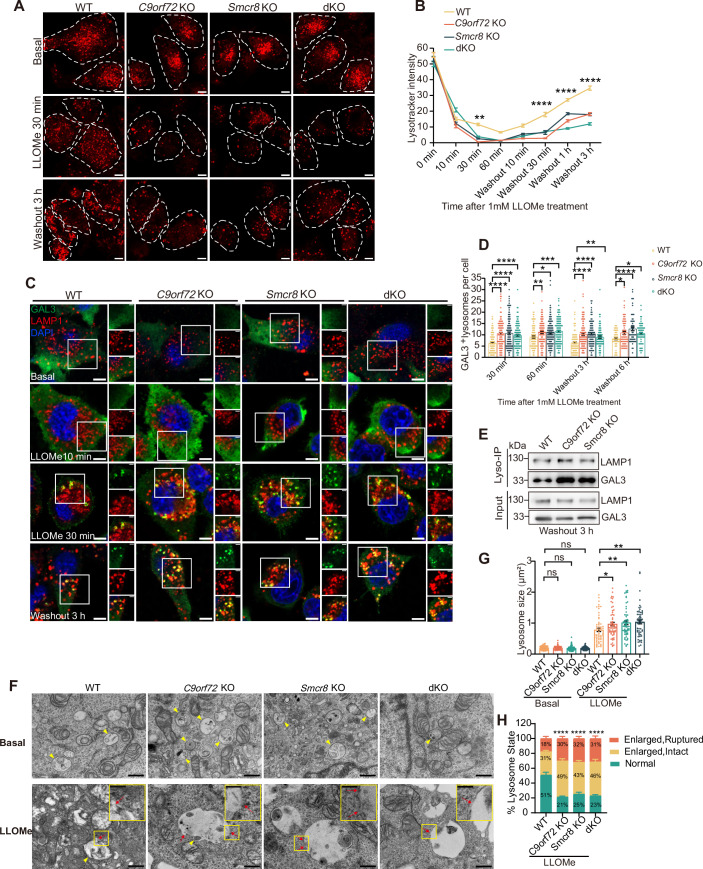
*C9orf72/Smcr8* deficiency impairs lysosomal membrane repair and increases lysosomal damage. (**A**) Representative fluorescence images of LysoTracker Red in WT, *C9orf72* KO, *Smcr8* KO, and dKO BV2 cells under basal conditions, after 30 min LLOMe (1 mM) treatment, and following 3 h washout. Nuclei were stained with DAPI (blue). Dashed lines indicate cell boundaries. Scale bar: 5 μm. (**B**) Time-course quantification of LysoTracker Red fluorescence intensity following LLOMe treatment and washout (*n* = 3 independent experiments). Asterisks indicate statistical significance for WT vs dKO comparisons at each time point: 30 min *P* = 0.0031, Washout 30 min *P* < 0.0001, Washout 1 h *P* < 0.0001, Washout 3 h *P* < 0.0001. (**C**) Immunofluorescence of GAL3 (green), LAMP1 (red), and DAPI (blue) at indicated times after LLOMe treatment. Scale bar: 5 µm (main), 2 μm (insets). Boxed areas are enlarged on the right. (**D**) Quantification of GAL3-positive lysosomes per cell across time points (*n* = 62–148 cells per genotype per time point, from three independent experiments). *P* values: 30 min (WT vs *C9* KO *P* < 0.0001, WT vs *S8* KO *P* < 0.0001, WT vs dKO *P* < 0.0001); 60 min (WT vs *C9* KO *P* = 0.009, WT vs *S8* KO *P* = 0.0219, WT vs dKO *P* = 0.0006); Washout 3 h (WT vs *C9* KO *P* < 0.0001, WT vs *S8* KO *P* < 0.0001, WT vs dKO *P* = 0.0035); Washout 6 h (WT vs *C9* KO *P* = 0.0119, WT vs *S8* KO *P* < 0.0001, WT vs dKO *P* = 0.0238). (**E**) LysoIP using TMEM192-3xHA system in WT, *C9orf72* KO, and *Smcr8* KO BV2 cells at 3 h post-LLOMe washout. Input and IP fractions were analyzed by immunoblotting for GAL3 and LAMP1. (**F**) TEM images of BV2 cells under basal (upper row) and 30 min of LLOMe treatment (lower row). Yellow arrowheads indicate lysosomes (intact lysosomes in basal condition; representative damaged lysosomes after LLOMe treatment). Red arrowheads indicate sites of lysosomal membrane rupture (membrane discontinuity). Yellow boxed areas showing ruptured lysosomes are enlarged in the upper right corner of each panel. Scale bars, 500 nm (main), 200 nm (insets). (**G**) Quantification of lysosome area in BV2 cells under basal and LLOMe conditions. Each dot represents a single lysosome (*n* =60 –77 lysosomes per genotype per condition, from three independent experiments). *P* values - LLOMe: WT vs *C9* KO *P* = 0.0197, WT vs *S8* KO *P* = 0.0062, WT vs dKO *P* = 0.0027. (**H**) Percentage distribution of lysosome states following LLOMe treatment. Normal (green, area ≤0.6 μm²); enlarged with intact membrane (yellow, area >0.6 μm²); enlarged with ruptured membrane (orange, area >0.6 μm² with membrane discontinuity). Data represent the average percentage of each lysosome state per cell (*n* = 20–23 cells per genotype per condition, from three independent experiments). *P* values for percentage of normal lysosomes: WT vs *C9* KO *P* < 0.0001, WT vs *S8* KO *P* < 0.0001, WT vs dKO *P* < 0.0001. Data information: In (**B**, **D**, **G**, **H**), data are presented as mean ± SEM from three independent experiments. Statistical significance was assessed by two-way ANOVA with Bonferroni post-hoc test (**B**, **D**) or unpaired two-tailed Student’s *t* test (**G**, **H**). **P < 0.01, ***P < 0.001, ****P < 0.0001; ns, not significant. Source data are available online for this figure.

Given our observations of enhanced inflammatory and phagocytic responses in C9orf72/SMCR8-deficient microglia along with GAL3-positive lysosomal accumulation in vivo, we investigated the subcellular localization and function of C9orf72/SMCR8 during lysosomal stress. Immunofluorescence analysis revealed that C9orf72 significantly colocalized with LAMP1 upon lysosomal damage induced by LLOMe treatment (Fig. EV3C). Using the TMEM192-3×HA lysosomal immunoprecipitation (Lyso-IP) system, we further confirmed enhanced association of both C9orf72 and SMCR8 with lysosomes during lysosomal stress conditions (Fig. EV3D,E), suggesting their potential roles in lysosomal stress response. To directly assess lysosomal functional integrity, we performed LysoTracker staining in BV2 cells to monitor lysosomal pH and membrane integrity over time. Under basal conditions, all genotypes showed comparable LysoTracker signals (Fig. 5A,B). Following 1 mM LLOMe treatment, WT cells showed an initial decrease in LysoTracker signal, followed by recovery after 3-h washout. In contrast, both *C9orf72* KO, *Smcr8* KO, and dKO cells exhibited markedly slower recovery, with LysoTracker signal remaining consistently and significantly lower than that of WT cells throughout the observation period (Fig. 5A,B), indicating impaired lysosomal repair and recovery following damage. To further characterize lysosomal damage, we examined GAL3 that specifically binds to β-galactoside residues exposed upon lysosomal membrane permeabilization and damage. Immunofluorescence analysis revealed significantly increased GAL3-positive lysosomes in both single and double knockout cells. This increase was evident during 30–60 min of LLOMe treatment. The enhanced GAL3 puncta persisted even after LLOMe washout (at 3-, and 6-h post-washout), indicating prolonged lysosomal damage in mutant cells (Fig. 5C,D). Lyso-IP analysis confirmed increased lysosome-associated GAL3 in mutant cells (Fig. 5E). Recapitulating these findings with even greater sensitivity, primary microglia from single and double knockout mice showed markedly elevated GAL3 levels as early as 30 min post-LLOMe (0.5 mM) treatment (Fig. EV3F,G). In BV2 cells, *C9orf72* KO, *Smcr8* KO, and dKO cells showed significantly reduced mature cathepsin D (CTSD) levels, indicating defective lysosomal protease activity (Fig. EV3H). To visualize lysosomal morphology and membrane integrity at ultrastructural resolution, we performed transmission electron microscopy (TEM) on BV2 cells under basal and LLOMe-treated conditions. Under basal conditions, lysosomes displayed comparable morphology and size distribution across all genotypes, with most lysosomes exhibiting normal electron density and intact membranes. Following LLOMe treatment, lysosomes in all genotypes exhibited characteristic stress responses including enlargement and decreased electron density. Quantitative analysis of lysosome size distribution revealed that *C9orf72* KO, *Smcr8* KO, and dKO cells harbored significantly larger lysosomes compared to WT cells (Fig. 5F,G). To further characterize lysosomal damage, we performed morphological classification, categorizing lysosomes into three distinct groups: normal, enlarged with intact membranes, and enlarged with ruptured membranes. Quantification showed that mutant cells had a significantly higher proportion of enlarged and ruptured lysosomes compared to WT (Fig. 5H). Lysosomal membrane ruptures, characterized by loss of membrane continuity, were readily visible in mutant cells following LLOMe treatment (Fig. 5F, red arrowheads indicate sites of membrane discontinuity in representative fields). Consistent with these observations, primary microglia from knockout mice displayed more severe lysosomal abnormalities following LLOMe treatment, including increased lysosomal size and membrane disruption (Fig. EV3I–K). Interestingly, primary microglia lysosomes were larger than BV2 lysosomes after LLOMe treatment (~ 2–4 μm² vs ~0.5–2 μm²), reflecting greater sensitivity to damage; therefore, lysosome size classifications (normal, enlarged, ruptured) were defined relative to wild-type controls within each cell type. These findings suggest that C9orf72 and SMCR8 play critical roles in maintaining lysosomal membrane integrity and facilitating lysosomal repair following damage.

### Impaired recruitment of ESCRT machinery to damaged lysosomes in C9orf72/SMCR8-deficient cells

**Figure 6:**
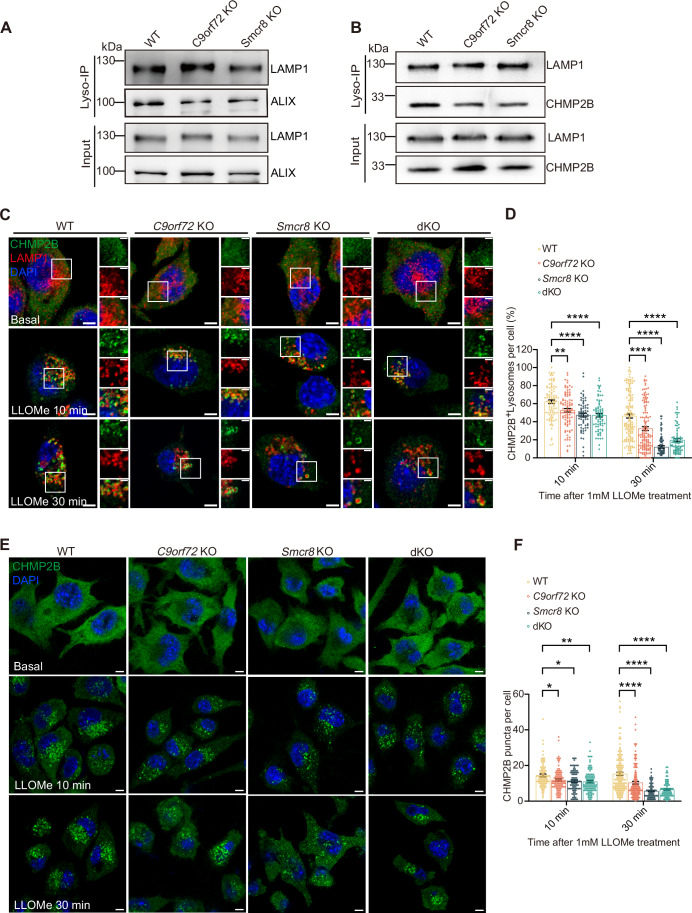
C9orf72 and SMCR8 deficiency impairs ESCRT recruitment to lysosomes. (**A**, **B**) LysoIP analysis of WT, *C9orf72* KO, and *Smcr8* KO BV2 cells. Total lysates and LysoIP fractions analyzed by Western blot for LAMP1, ALIX (**A**), and CHMP2B (**B**). (**C**) Representative immunofluorescence images of CHMP2B (green), LAMP1 (red), and DAPI (blue) in WT and knockout BV2 cells under basal conditions and after LLOMe treatment (1 mM) for 10- and 30-min. Boxed areas are enlarged on the right showing individual channels and merged images. Scale bars, 5 μm (main), 2 μm (insets). (**D**) Quantification of CHMP2B recruitment to lysosomes, expressed as percentage of LAMP1-positive lysosomes showing CHMP2B per cell. Each dot represents an individual cell (*n* = 70–140 cells per genotype per condition, from three independent experiments). *P* values - 10 min LLOMe: WT vs *C9* KO *P* = 0.0043, WT vs *S8* KO *P* < 0.0001, WT vs dKO *P* < 0.0001; 30 min LLOMe: WT vs *C9* KO *P* < 0.0001, WT vs *S8* KO *P* < 0.0001, WT vs dKO *P* < 0.0001. (**E**) Representative immunofluorescence images of CHMP2B (green) and DAPI (blue) in WT, and knockout BV2 cells under basal conditions and after LLOMe treatment for 10 and 30 min, showing overall CHMP2B distribution pattern. Scale bars, 5 μm. (**F**) Quantification of CHMP2B puncta per cell at indicated time points after LLOMe treatment. Each dot represents an individual cell (*n* = 78–141 cells per genotype per condition, from three independent experiments). *P* values - 10 min: WT vs *C9* KO *P* = 0.0492, WT vs *S8* KO *P* = 0.0123, WT vs dKO *P* = 0.0041; 30 min: WT vs *C9* KO *P* < 0.0001, WT vs *S8* KO *P* < 0.0001, WT vs dKO *P* < 0.0001. Data information: In (**D**, **F**), data are presented as mean ± SEM from three independent experiments. Statistical significance was assessed by two-way ANOVA with Bonferroni post-hoc test. *P < 0.05, **P < 0.01, ****P < 0.0001. Source data are available online for this figure.

Since ESCRT machinery is rapidly recruited to repair damaged lysosomes, acting as a primary defense mechanism that precedes autophagy-mediated clearance (lysophagy) (Radulovic et al, 2018; Skowyra et al, 2018), we examined whether ESCRT recruitment was compromised in *C9orf72/Smcr8*-deficient cells. ALIX (ALG-2-interacting protein X) functions as an ESCRT-associated adaptor protein that bridges ESCRT-I and ESCRT-III complexes to facilitate membrane scission during lysosomal repair processes (Radulovic et al, 2018), while CHMP2B is a core component of the ESCRT-III complex essential for membrane remodeling and repair. Lyso-IP experiments revealed significantly reduced association of ALIX and CHMP2B with lysosomes in mutant cells (Fig. 6A,B). To further characterize ESCRT recruitment dynamics, we examined CHMP2B recruitment to lysosomes using immunofluorescence analysis. Under basal conditions, CHMP2B showed diffuse cytoplasmic distribution across all genotypes. Following 1 mM LLOMe treatment, WT cells exhibited increased CHMP2B puncta that recruited to lysosomes (LAMP1-positive vesicles) within 10–30 min. In contrast, *C9orf72* KO, *Smcr8* KO, and dKO cells showed significantly reduced CHMP2B-positive lysosomes at these early time points (Fig. 6C,D). The total number of LLOMe-induced CHMP2B puncta per cell was also significantly decreased in all mutant cell lines compared to WT (Fig. 6E,F), further demonstrating defective ESCRT-III recruitment in response to lysosomal damage. These findings suggest that the C9orf72/SMCR8 complex plays a crucial role in facilitating ESCRT-mediated lysosomal repair.

### C9orf72/SMCR8 deficiency causes RAB8A hyperphosphorylation and mislocalization to RAB7^+^/LAMP1^−^ vesicles upon lysosomal damage

**Figure EV4:**
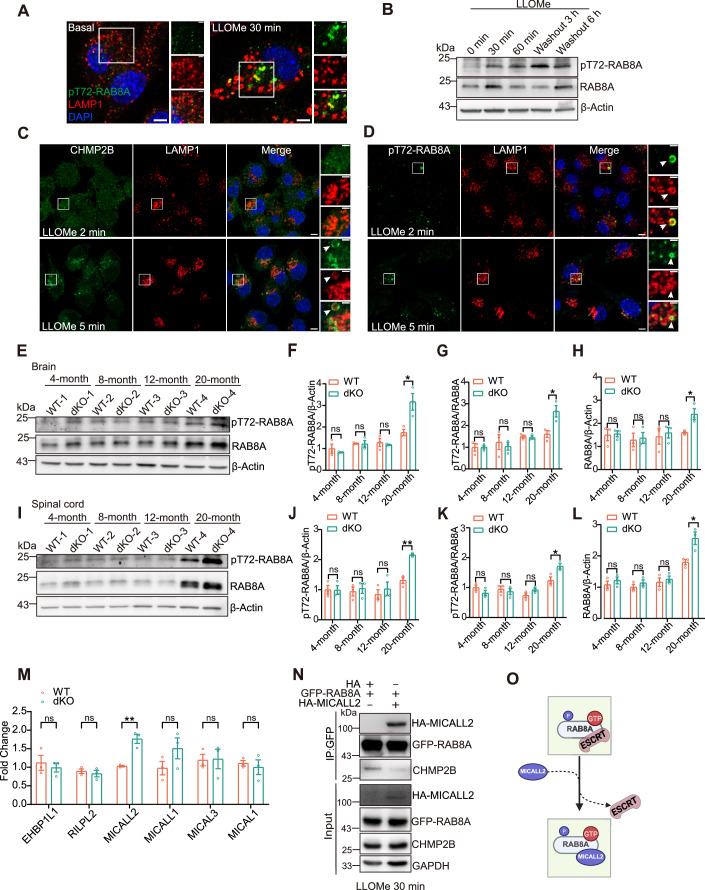
pT72-RAB8A accumulation in C9orf72/SMCR8-deficient tissues and cells. (**A**) Representative immunofluorescence images of pT72-RAB8A (green), LAMP1 (red), and DAPI (blue) in BV2 cells under basal conditions and after LLOMe treatment (1 mM, 30 min). Boxed areas are enlarged on the right. Scale bars, 5 μm (main), 2 μm (insets). (**B**) Time‑course immunoblotting analysis of pT72‑RAB8A dynamics in BV2 cells following LLOMe treatment and washout. Cell lysates were probed with antibodies against pT72‑RAB8A (top), total RAB8A (middle), and β‑actin (loading control). (**C**, **D**) Representative immunofluorescence images showing temporal recruitment of CHMP2B (**C**) and pT72-RAB8A (**D**) (green) to lysosomes (LAMP1, red) at 2 and 5 min after LLOMe treatment (1 mM). DAPI (blue) marks nuclei. White arrows indicate colocalization of CHMP2B or pT72-RAB8A with LAMP1-positive lysosomes. Boxed areas are enlarged on the right showing individual channels and merged images. Scale bars, 5 μm (main), 2 μm (insets). (**E**–**H**) Age-dependent expression of pT72-RAB8A and total RAB8A in brain tissue from WT and dKO mice at 4, 8, 12, and 20 months of age (**E**). β-Actin serves as loading control. Quantification of pT72-RAB8A normalized to β-actin (**F**), pT72-RAB8A normalized to total RAB8A (**G**), and total RAB8A normalized to β-actin (**H**) (*n *= 3 mice per group). 20-month: pT72-RAB8A/β-actin, *P* = 0.0256; pT72-RAB8A/RAB8A, *P* = 0.0281; RAB8A/β-actin, *P* = 0.0299 (**I**–**L**) Age-dependent expression of pT72-RAB8A and total RAB8A in spinal cord tissue from WT and dKO mice at 4, 8, 12, and 20 months of age (**I**). β-Actin serves as loading control. Quantification of pT72-RAB8A normalized to β-actin (**J**), pT72-RAB8A normalized to total RAB8A (**K**), and total RAB8A normalized to β-actin (**L**) (*n* = 3 mice per group). 20-month: pT72-RAB8A/β-actin, *P* = 0.0018; pT72-RAB8A/RAB8A, *P* = 0.0347; RAB8A/β-actin, *P* = 0.0257). (**M**) Quantification of RAB8A effector protein binding fold-change (dKO/WT) from mass spectrometry analysis of RAB8A immunoprecipitates. WT and dKO BV2 cells were treated with LLOMe (1 mM, 30 min) prior to lysis and immunoprecipitation (*n* = 3 biological replicates; MICALL2: *P* = 0.0041, all other effectors: ns). (**N**) Co-IP analysis of GFP-RAB8A with HA-MICALL2 in HEK293T cells after LLOMe treatment (1 mM, 30 min). Input and IP fractions were analyzed by immunoblotting for HA-MICALL2, GFP-RAB8A, CHMP2B, and GAPDH. (**O**) Schematic model illustrating competitive binding of ESCRT machinery and MICALL2 to RAB8A upon lysosomal damage. Data information: In (**F**–**H**, **J**–**L**, **M**), data are presented as mean ± SEM from three independent experiments. Statistical significance was assessed unpaired two-tailed Student’s *t* test. *P < 0.05, **P < 0.01, ****P < 0.0001; ns, not significant. Source data are available online for this figure.

**Figure 7:**
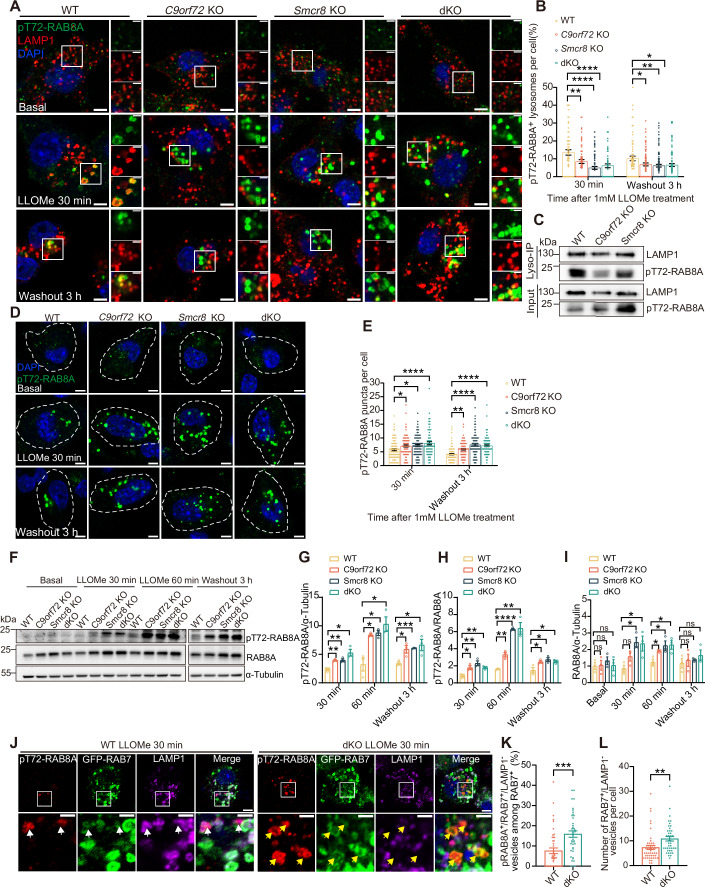
*C9orf72/Smcr8* deficiency causes RAB8A hyperphosphorylation and impaired lysosomal recruitment after lysosomal damage. (**A**) Representative immunofluorescence images of pT72-RAB8A (green), LAMP1 (red), and DAPI (blue) in WT, *C9orf72* KO, *Smcr8* KO, and dKO BV2 cells under basal conditions, after LLOMe treatment (1 mM, 30 min), and following 3 h washout. Boxed areas are enlarged on the right. Scale bars, 5 μm (main), 2 μm (insets). (**B**) Quantification of pT72-RAB8A recruitment to lysosomes, expressed as percentage of LAMP1-positive lysosomes showing pT72-RAB8A colocalization per cell. Each dot represents an individual cell (*n* = 60–102 cells per genotype per condition, from three independent experiments). *P* values - 30 min LLOMe: WT vs *C9* KO *P* = 0.003, WT vs *S8* KO *P* < 0.0001, WT vs dKO *P* < 0.0001; Washout 3 h: WT vs *C9* KO *P* = 0.0452, WT vs *S8* KO *P* = 0.0084, WT vs dKO *P* = 0.0157. (**C**) LysoIP using TMEM192-3×HA system in WT, *C9orf72* KO, and *Smcr8* KO BV2 cells after LLOMe treatment (1 mM, 30 min). Input and IP fractions were analyzed by immunoblotting for pT72-RAB8A and LAMP1. (**D**) Representative immunofluorescence images of pT72-RAB8A (green) and DAPI (blue) in WT and knockout BV2 cells, showing overall pT72-RAB8A distribution. Dashed lines indicate cell boundaries. Scale bar: 5 μm. (**E**) Quantification of pT72-RAB8A puncta per cell (*n* = 70–98 cells per genotype per time point, from three independent experiments). *P* values - 30 min: WT vs *C9* KO *P* = 0.0286, WT vs *S8* KO *P* = 0.0114, WT vs dKO *P* < 0.0001; Washout 3 h: WT vs *C9* KO *P* = 0.0014, WT vs *S8* KO *P* < 0.0001, WT vs dKO *P* < 0.0001. (**F**) Immunoblotting analysis of pT72-RAB8A levels in WT, C*9orf72* KO, *Smcr8* KO, and dKO BV2 cells under basal conditions and at indicated time points after LLOMe treatment and washout. Lysates were probed for pT72-RAB8A, total RAB8A, and α-tubulin (loading control). (**G**–**I**) Quantification of pT72-RAB8A protein levels normalized to α-tubulin (**G**) *P* values- 30 min: WT vs *C9* KO *P* = 0.0034, WT vs *S8* KO *P* = 0.0091, WT vs dKO *P* = 0.01, 60 min: WT vs *C9* KO *P* = 0.0111, WT vs *S8* KO *P* = 0.0108, WT vs dKO *P* = 0.0145, Washout 3 h: WT vs *C9* KO *P* = 0.027, WT vs *S8* KO *P* = 0.0006, WT vs dKO *P* = 0.0341; pT72-RAB8A protein levels normalized to normalized to total RAB8A (**H**) *P* values- 30 min: WT vs *C9* KO *P* = 0.0237, WT vs *S8* KO *P* = 0.0096, WT vs dKO *P* = 0.0073, 60 min: WT vs *C9* KO *P* = 0.0035, WT vs *S8* KO *P* < 0.0001, WT vs dKO *P* = 0.0022, Washout 3 h: WT vs *C9* KO *P* = 0.0477, WT vs *S8* KO *P* = 0.0316, WT vs dKO *P* = 0.041; and total RAB8A normalized to α-tubulin (**I**) *P* values- Basal: WT vs *C9* KO *P* = 0.9137, WT vs *S8* KO *P* = 0.3774, WT vs dKO *P* = 0.9129, *P* values- 30 min: WT vs *C9* KO *P* = 0.1013, WT vs *S8* KO *P* = 0.012, WT vs dKO *P* = 0.0384, 60 min: WT vs *C9* KO *P* = 0.0191, WT vs *S8* KO *P* = 0.0387, WT vs dKO *P* = 0.0308, Washout 3 h: WT vs *C9* KO *P* = 0.7186, WT vs *S8* KO *P* = 0.5191, WT vs dKO *P* = 0.3278; (*n* = 3 independent experiments). (**J**) Representative immunofluorescence images of BV2 cells transfected with GFP-RAB7 (green) and stained for pT72-RAB8A (red), LAMP1 (magenta), and DAPI (blue) following LLOMe treatment (1 mM, 30 min). Boxed areas are enlarged below. White arrows indicate pT72-RAB8A-positive, GFP-RAB7-positive, LAMP1-positive vesicles (damaged lysosomes). Yellow arrows indicate pT72-RAB8A-positive, GFP-RAB7-positive, LAMP1-negative vesicles. Scale bars, 5 μm (main), 2 μm (insets). (**K**) Quantification of pT72-RAB8A recruitment to LAMP1-negative, RAB7-positive vesicles. Data are expressed as percentage of pT72-RAB8A-positive, LAMP1-negative, RAB7-positive vesicles relative to total RAB7-positive vesicles per cell. Each dot represents an individual cell (*n* = 42–46 cells per genotype, from three independent experiments). *P* = 0.0002. (**L**) Quantification of RAB7^+^/LAMP1^−^ (RAB7-positive, LAMP1-negative) vesicles per cell. Each dot represents an individual cell (*n* = 42–49 cells per genotype, from three independent experiments). *P* = 0.0055. Data information: In (**B**, **E**,** G**–**I**, **K**, **L**), data are presented as mean ± SEM from three independent experiments. Statistical significance was assessed by two-way ANOVA with Bonferroni post-hoc test (**B**, **E**) or unpaired two-tailed Student’s *t* test (**G**, **H**, **I**, **K**, **L**). *P < 0.05, **P < 0.01, ***P < 0.001, ****P < 0.0001; ns, not significant. Source data are available online for this figure.

Phosphorylated RAB8A (pT72-RAB8A), activated by LRRK2 kinase, plays a critical role in recruiting ESCRT-III components to damaged lysosomes for membrane repair in macrophages (Herbst et al, 2020). To investigate how C9orf72/SMCR8 deficiency affects this process, we examined pT72-RAB8A dynamics following LLOMe-induced lysosomal damage. In WT BV2 cells, pT72-RAB8A displayed minimal punctate staining under basal conditions but was rapidly recruited to LAMP1-positive lysosomes within 30 min of 1 mM LLOMe treatment (Fig. EV4A). Western blot analysis revealed time-dependent increases in pT72-RAB8A levels following LLOMe treatment, with signals persisting even after washout (Fig. EV4B). Notably, time-course analysis revealed that pT72-RAB8A was recruited to damaged lysosomes at 2 min after LLOMe treatment (Fig. EV4C), earlier than CHMP2B, which became detectable at 5 min (Fig. EV4D), suggesting that RAB8A phosphorylation precedes and likely recruits ESCRT machinery at sites of lysosomal damage. *C9orf72* KO, *Smcr8* KO, and dKO cells, however, exhibited markedly altered pT72-RAB8A distribution patterns. While WT cells showed robust pT72-RAB8A colocalization with LAMP1-positive lysosomes following LLOMe treatment, knockout cells displayed significantly reduced lysosomal localization of pT72-RAB8A (Fig. 7A,B). Lyso-IP analysis confirmed significantly decreased lysosome-associated pT72-RAB8A in mutant cells (Fig. 7C). Interestingly, despite reduced lysosomal recruitment, total cellular pT72-RAB8A puncta were increased in mutant cells, with enhanced accumulation in non-lysosomal cytoplasmic compartments (Fig. 7A–E). Western blot analysis revealed distinct pT72-RAB8A dynamics between genotypes. While WT cells showed modest and transient pT72-RAB8A elevation following LLOMe treatment, *C9orf72* KO, *Smcr8* KO, and dKO cells displayed significantly elevated pT72-RAB8A levels at all time points examined, including 30 min and 60 min of LLOMe treatment, as well as 3 h post-washout (Fig. 7F–I). The phosphorylation levels peaked at 60 min of LLOMe treatment in the knockout cells, suggesting that lysosomal damage repair defects in mutant cells are accompanied by hyperphosphorylation of RAB8A, along with a slight increase in total RAB8A protein levels (Fig.7F,I). This dysregulated RAB8A phosphorylation phenotype was recapitulated in vivo, with elevated pT72-RAB8A signals detected in brain and spinal cord tissues from 20-month-old knockout mice (Fig. EV4E–L), indicating that the dysregulated RAB8A phosphorylation observed in cell culture models reflects pathological changes occurring in the aging nervous system.

**Figure EV5:**
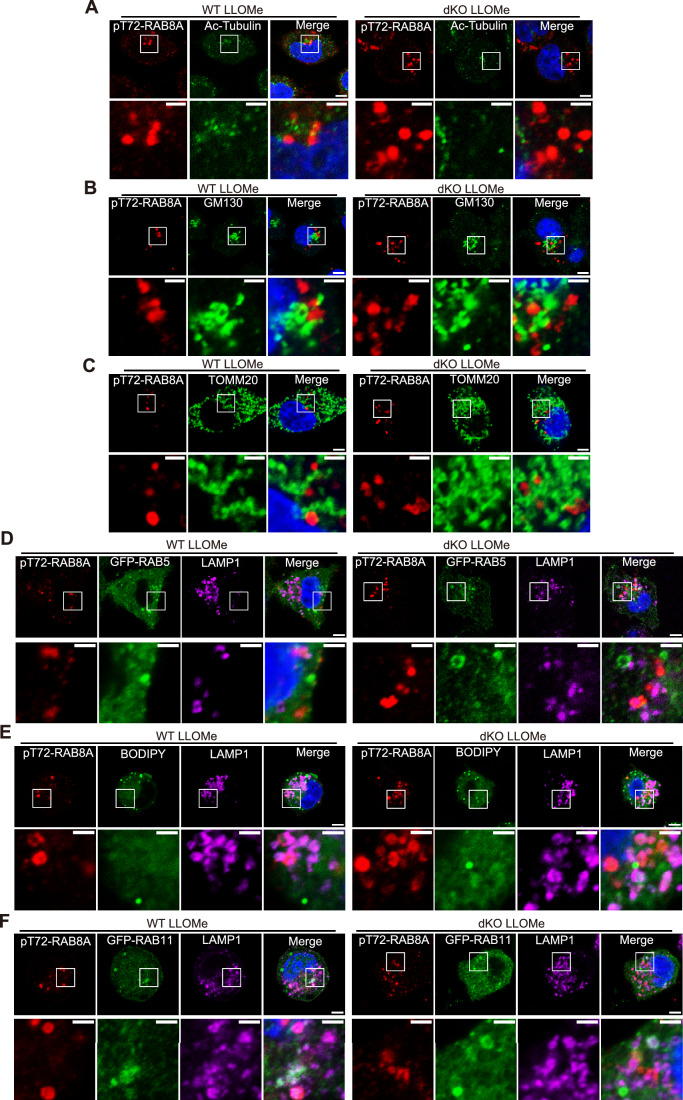
pT72-RAB8A does not localize to primary cilia, Golgi, mitochondria, or lipid droplets following lysosomal damage. (**A**) Representative immunofluorescence images of pT72-RAB8A (red) and acetylated α-tubulin (Ac-tubulin, green; primary cilia marker) in WT and dKO BV2 cells after LLOMe treatment (1 mM, 30 min). DAPI (blue) marks nuclei. Boxed areas are enlarged below showing individual channels and merged images. Scale bar, 5 μm. (**B**) Representative immunofluorescence images of pT72-RAB8A (red) and GM130 (green; Golgi marker) in WT and dKO BV2 cells after LLOMe treatment (1 mM, 30 min). DAPI (blue) marks nuclei. Boxed areas are enlarged below. Scale bar, 5 μm. (**C**) Representative immunofluorescence images of pT72-RAB8A (red) and TOMM20 (green; mitochondrial marker) in WT and dKO BV2 cells after LLOMe treatment (1 mM, 30 min). DAPI (blue) marks nuclei. Boxed areas are enlarged below. Scale bar, 5 μm. (**D**) Representative immunofluorescence images of pT72-RAB8A (red), GFP-RAB5 (green; early endosome marker), and LAMP1 (magenta; lysosome marker) in WT and dKO BV2 cells after LLOMe treatment (1 mM, 30 min). DAPI (blue) marks nuclei. Boxed areas are enlarged below. Scale bar, 5 μm. (**E**) Representative immunofluorescence images of pT72-RAB8A (red), BODIPY (green; lipid droplet marker), and LAMP1 (magenta) in WT and dKO BV2 cells after LLOMe treatment (1 mM, 30 min). DAPI (blue) marks nuclei. Boxed areas are enlarged below. Scale bar, 5 μm. (**F**) Representative immunofluorescence images of pT72-RAB8A (red), GFP-RAB11 (green; recycling endosome marker), and LAMP1 (magenta) in WT and dKO BV2 cells after LLOMe treatment (1 mM, 30 min). DAPI (blue) marks nuclei. Boxed areas are enlarged below. Scale bar, 5 μm. Data information: Images are representative of three independent experiments. Source data are available online for this figure.

To characterize the identity of the non-lysosomal compartments harboring pT72-RAB8A in mutant cells, we performed systematic colocalization analysis with various organelle markers. Immunofluorescence staining revealed that LAMP1-negative pT72-RAB8A puncta in dKO cells did not colocalize with markers for primary cilia (acetylated α-tubulin), early endosomes (GFP-RAB5), lipid droplets (BODIPY), Golgi apparatus (GM130), mitochondria (TOMM20), or recycling endosomes (GFP-RAB11) (Fig. EV5A–F). However, a substantial proportion of LAMP1-negative pT72-RAB8A puncta showed colocalization with the late endosome marker GFP-RAB7 in dKO cells (Fig. 7J,K). Quantitative analysis demonstrated that dKO cells exhibited both increased accumulation of pT72-RAB8A on RAB7^+^/LAMP1^−^ vesicles (Fig. 7K) and an expanded pool of RAB7^+^/LAMP1^-^ late endosomes (Fig. 7L). These findings reveal that under lysosomal damage conditions, hyperphosphorylated RAB8A is sequestered on RAB7^+^ late endosomal intermediates that have not matured to LAMP1^+^ lysosomes. RAB7^+^/LAMP1^−^ vesicles represent “early late endosomes”—pre-lysosomal intermediates that require RAB7-dependent transport to fuse with terminal LAMP1-positive lysosomes (Henry et al, 2006; Yap et al, 2018).

Given that pT72-RAB8A recruits ESCRT components to damaged lysosomes (Herbst et al, 2020), we observed that in knockout cells, despite elevated pT72-RAB8A accumulation on non-lysosomal RAB7^+^/LAMP1^-^ compartments (Fig. 7J–L), ESCRT recruitment was impaired at both lysosomes and these non-lysosomal sites, with globally reduced ESCRT puncta (Fig. 6). This suggests that pT72-RAB8A at ectopic late endosome locations cannot recruit ESCRT machinery, leading us to hypothesize that effector proteins competitively block RAB8A-ESCRT interaction. RAB8A immunoprecipitation followed by mass spectrometry revealed increased association of the effector MICALL2 with RAB8A in dKO cells compared to WT cells following LLOMe treatment (Fig. EV4M). Co-IP experiments confirmed that MICALL2 overexpression markedly reduced RAB8A-CHMP2B association (Fig. EV4N,O), demonstrating competitive binding. These data reveal that hyperphosphorylated RAB8A on RAB7^+^/LAMP1^−^ late endosomes is bound by MICALL2, which occupies ESCRT-binding sites, preventing both ESCRT recruitment and pT72-RAB8A trafficking to damaged lysosomes. Collectively, these results demonstrate that the C9orf72-SMCR8 complex is essential for proper targeting of pT72-RAB8A to damaged lysosomes. Loss of C9orf72 or SMCR8 results in RAB8A hyperphosphorylation combined with mistargeting to late endosomes, thereby impairing ESCRT recruitment and lysosomal membrane repair.

### Loss of *C9orf72/Smcr8* leads to accumulation of GTP-Bound RAB8A and enhanced phosphorylation

**Figure 8:**
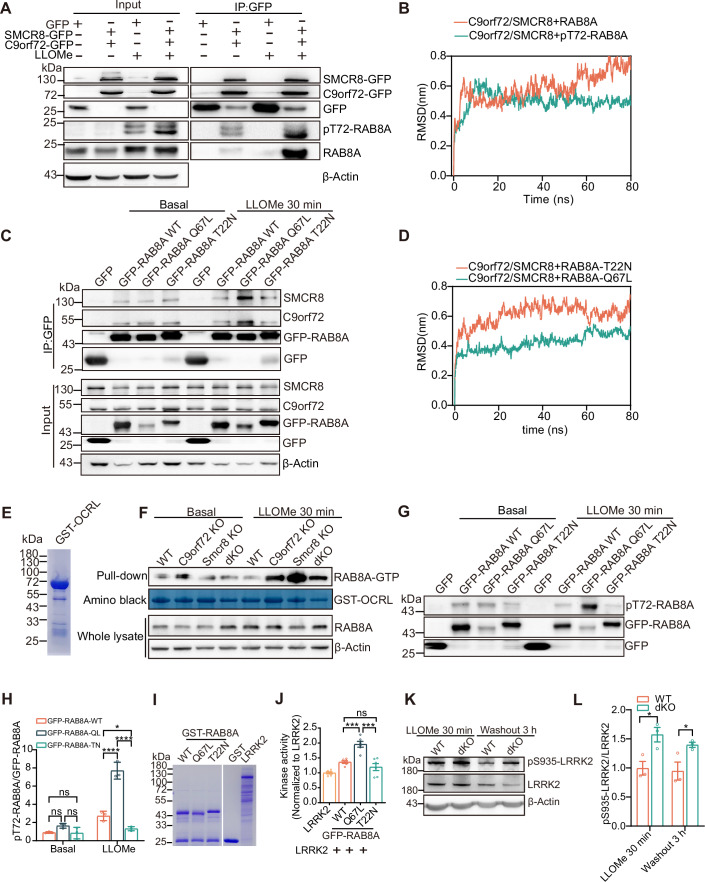
GTP-bound RAB8A shows enhanced interaction with the C9orf72/SMCR8 complex and promotes T72 phosphorylation. (**A**) Co-immunoprecipitation analysis of BV2 cells expressing SMCR8-GFP, C9orf72-GFP, or control GFP vector under basal conditions or after LLOMe treatment (1 mM, 30 min). Input and GFP-IP fractions were analyzed by immunoblotting for GFP, SMCR8-GFP, C9orf72-GFP, pT72-RAB8A, total RAB8A, and β-actin. (**B**) Molecular dynamics simulation showing enhanced stability of the C9orf72-SMCR8-pT72-RAB8A complex (green) compared to the C9orf72-SMCR8-RAB8A complex (orange) over simulation time. (**C**) Co-immunoprecipitation analysis of HEK293T cells expressing GFP-tagged RAB8A variants (WT, Q67L, T22N) under basal conditions or after LLOMe treatment (1 mM, 30 min). Input and GFP-IP fractions were analyzed by immunoblotting for SMCR8, C9orf72, pT72-RAB8A, GFP, and β-actin. (**D**) Molecular dynamics simulation demonstrating greater stability of C9orf72-SMCR8 interaction with GTP-bound RAB8A Q67L (green) versus GDP-bound T22N (orange). (**E**) Coomassie blue staining of purified recombinant GST-OCRL protein used for RAB8A-GTP pulldown assays. (**F**) GST-OCRL pulldown analysis of lysates from WT, *C9orf72* KO, *Smcr8* KO, and dKO BV2 cells under basal conditions or after LLOMe treatment (1 mM, 30 min). Pulldown fractions were analyzed by immunoblotting for RAB8A-GTP. Amido black staining shows GST-OCRL loading. Whole lysates were analyzed for total RAB8A and GAPDH. (**G**, **H**) GFP immunoprecipitation of RAB8A variants (WT, Q67L, T22N) from HEK293T cells under basal conditions or after LLOMe treatment (1 mM, 30 min). IP fractions were analyzed by immunoblotting for pT72-RAB8A and GFP-RAB8A. Target bands are detected above 43 kDa. Quantification of pT72-RAB8A normalized to total GFP-RAB8A is shown in (**H**) (*n* = 3 independent experiments, *P* values - LLOMe: WT vs Q67L: *P* < 0.0001, WT vs T22N: *P* = 0.0172, Q67L vs T22N: *P* < 0.0001. (**I**, **J**) In vitro LRRK2 kinase assay using recombinant RAB8A protein variants. Coomassie blue staining confirms equal protein loading (**I**). Quantification demonstrates enhanced kinase activity toward the GTP‑bound RAB8A Q67L compared with other variants (**J**). (*n* = 6 independent experiments, WT vs Q67L: *P* = 0.0002, WT vs T22N: *P* = 0.2139, Q67L vs T22N: *P* = 0.0005). (**K**, **L**) Immunoblotting analysis of pS935-LRRK2, total LRRK2, and β-actin in WT and dKO BV2 cells after LLOMe treatment (1 mM, 30 min) or 3-h washout following LLOMe (K). Quantification of pS935-LRRK2 normalized to total LRRK2 is shown in (**L**) (*n* = 3 independent experiments; LLOMe 30 min: WT vs dKO: *P* = 0.0279; Washout 3 h: WT vs dKO: *P* = 0.0486). Data information: In (**H**, **J**, **L**), data are presented as mean ± SEM from three independent experiments. Statistical significance was assessed by two-way ANOVA with Bonferroni post-hoc test (**H**) or unpaired two-tailed Student’s *t* test (**J**, **L**). *P < 0.05, **P < 0.01, ***P < 0.001, ****P < 0.0001; ns, not significant. Source data are available online for this figure.

Given previous findings that RAB8A is a substrate of the C9orf72/SMCR8 GAP complex in ciliogenesis, where RAB8A shows abnormal ciliary accumulation in C9orf72/SMCR8-deficient cells (Tang et al, 2023), we investigated whether similar dysregulation occurs in microglia during lysosomal damage. Notably, BV2 microglia lack detectable cilia under LLOMe treatment conditions (Fig. EV5A), indicating that the RAB8A dysregulation observed in C9orf72/SMCR8-deficient cells reflects cilium-independent regulation of RAB8A activity during lysosomal damage responses. To examine the interaction between the C9orf72/SMCR8 complex and RAB8A during lysosomal damage, we co-expressed SMCR8-GFP and C9orf72-GFP in BV2 cells and performed co-immunoprecipitation under basal and LLOMe-treated conditions. Remarkably, the C9orf72/SMCR8 complex showed significantly enhanced association with pT72-RAB8A following LLOMe treatment compared to basal conditions (Fig. 8A). Molecular dynamics simulations further unveiled that the phosphorylation of RAB8A enhanced its binding stability with the C9orf72/SMCR8 complex (Fig. 8B). To directly assess the GAP relationship, we performed co-immunoprecipitation experiments using GFP-tagged RAB8A variants (wild-type, GTP-bound Q67L, and GDP-bound T22N) following LLOMe treatment. Co-immunoprecipitation experiments and molecular dynamics simulations demonstrated that the C9orf72/SMCR8 complex showed preferential binding to the GTP-bound form of RAB8A (Fig. 8C,D). We employed GST-OCRL as bait, which specifically binds the GTP-bound form of RAB8A (Hou et al, 2011). Following LLOMe treatment, *C9orf72* KO, *Smcr8* KO, and dKO cells exhibited significantly increased levels of GTP-bound RAB8A compared to WT cells (Fig. 8E,F), indicating impaired GTPase activity and accumulation of the active, GTP-bound form of RAB8A in mutant cells. Given the observed accumulation of GTP-bound RAB8A and the increased phosphorylation in knockout cells, we hypothesized that the GTP-bound state of RAB8A might be preferentially phosphorylated by LRRK2. To test this, we examined phospho-T72 levels in cells expressing different RAB8A variants under basal and LLOMe-treated conditions. While all RAB8A variants showed minimal phosphorylation under basal conditions, the GTP-bound Q67L mutant exhibited markedly enhanced phospho-T72 signals following LLOMe treatment compared to wild-type or GDP-bound T22N RAB8A (Fig. 8G,H), suggesting that the GTP-bound state promotes LRRK2-mediated RAB8A phosphorylation. To directly demonstrate that GTP-bound RAB8A is a preferred substrate for LRRK2, we performed in vitro kinase assays using purified GST-fused RAB8A variants (WT, Q67L, and T22N) and recombinant LRRK2-HIS. Kinase activity was quantified using an ATP-Glo luminescence assay, which measures ATP consumption as a readout of phosphorylation. The GTP-bound Q67L mutant showed significantly higher LRRK2-mediated phosphorylation compared to wild-type and GDP-bound forms (Fig. 8I,J).

To directly assess LRRK2 activity in knockout cells, we examined phosphorylation of LRRK2 at Ser935 (pS935-LRRK2), a phosphorylation site whose level correlates with LRRK2 kinase activity (Chiang et al, 2025; Dzamko et al, 2010; Kalogeropulou et al, 2022). Western blot analysis revealed that pS935-LRRK2 levels were elevated in dKO BV2 cells following LLOMe treatment compared to WT cells, indicating enhanced LRRK2 kinase activity in knockout cells upon lysosomal damage (Fig. 8K,L). These findings establish that the increased phosphorylation of RAB8A in C9orf72/SMCR8-deficient cells is attributable to both enhanced LRRK2 kinase activity and the accumulation of the GTP-bound form of RAB8A, which serves as a preferred substrate for LRRK2-mediated phosphorylation.

### Rescue of lysosomal repair requires C9orf72/SMCR8 GAP activity

**Figure 9:**
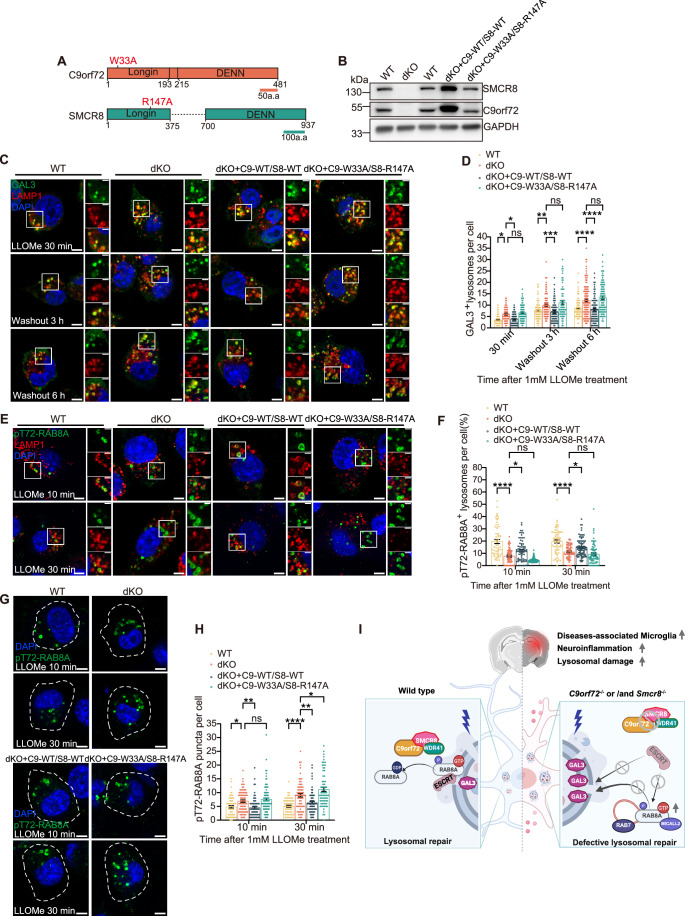
GAP activity of C9orf72/SMCR8 is required for lysosomal damage rescue. (**A**) Schematic representation of C9orf72 and SMCR8 protein domain architecture. C9orf72 contains a longin domain and a DENN domain. SMCR8 contains a longin domain and a DENN domain. GAP-deficient mutations are indicated: W33A in C9orf72 longin domain, R147A in SMCR8 longin domain. Numbers indicate amino acid positions. (**B**) Immunoblotting analysis showing expression levels of WT and GAP-mutant C9orf72 and SMCR8 proteins in dKO BV2 cells. GAPDH serves as loading control. (**C**) Representative immunofluorescence images of WT, dKO, and dKO BV2 cells expressing WT or GAP-mutant (C9-W33A/S8-R147A) C9orf72/SMCR8 proteins after LLOMe treatment (1 mM, 30 min) and following 3 h and 6 h washout. Cells were stained for GAL3 (green), LAMP1 (red), and DAPI (blue). Boxed areas are enlarged on the right showing individual puncta. Scale bar, 5 μm (main), 2 μm (insets). (**D**) Quantification of GAL3-positive lysosomes per cell in WT, dKO, dKO+C9-WT/S8-WT, and dKO+C9-W33A/S8-R147A at indicated time points after LLOMe treatment. Each dot represents one cell (*n* = 61–142 cells per genotype per condition from three independent experiments). 30 min: WT vs dKO, *P* = 0.0176; dKO+C9-WT/S8-WT vs dKO, *P* = 0.0439; dKO+C9-W33A/S8-R147A vs dKO, *P* = 0.8403; 3 h: WT vs dKO, *P* = 0.0097; dKO+C9-WT/S8-WT vs dKO, *P* = 0.0007; dKO+C9-W33A/S8-R147A vs dKO, *P* = 0.3958; 6 h: WT vs dKO, *P* < 0.0001; dKO+C9-WT/S8-WT vs dKO, *P* < 0.0001; dKO+C9-W33A/S8-R147A vs dKO, *P* = 0.255). (**E**) Representative immunofluorescence images of WT, dKO, and dKO BV2 cells expressing WT or GAP-mutant C9orf72/SMCR8 proteins after LLOMe treatment (1 mM) for 10 min and 30 min. Cells were stained for pT72-RAB8A (green), LAMP1 (red), and DAPI (blue). Boxed areas are enlarged on the right. Scale bar, 5 μm (main), 2 μm (insets). (**F**) Quantification of pT72-RAB8A recruitment to lysosomes in WT, dKO, dKO+C9-WT/S8-WT, and dKO+C9-W33A/S8-R147A. Data are expressed as percentage of LAMP1-positive lysosomes showing pT72-RAB8A colocalization per cell. Each dot represents one cell (*n* = 59-88 cells per condition from three independent experiments). 10 min: WT vs dKO, *P* < 0.0001; dKO+C9-WT/S8-WT vs dKO, *P* = 0.0118; dKO+C9-W33A/S8-R147A vs dKO, *P* = 0.1003; 30 min: WT vs dKO, *P* < 0.0001; dKO+C9-WT/S8-WT vs dKO, *P* = 0.0445; dKO+C9-W33A/S8-R147A vs dKO, *P* = 0.5845. (**G**) Representative immunofluorescence images showing overall pT72-RAB8A distribution pattern in WT, dKO, and dKO cells expressing WT or GAP-mutant C9orf72/SMCR8 proteins after LLOMe treatment for 10 min and 30 min. pT72-RAB8A (green), DAPI (blue). Dashed lines indicate cell boundaries. Scale bar, 5 μm. (**H**) Quantification of pT72-RAB8A puncta per cell in WT, dKO, dKO+C9-WT/S8-WT, and dKO+C9-W33A/S8-R147A at indicated time points after LLOMe treatment. Each dot represents one cell (*n* = 64–93 cells per condition from three independent experiments). 10 min: WT vs dKO, *P* = 0.0275; dKO+C9-WT/S8-WT vs dKO, *P* = 0.0045; dKO+C9-W33A/S8-R147A vs dKO, *P* = 0.7682; 30 min: WT vs dKO, *P* < 0.0001; dKO+C9-WT/S8-WT vs dKO, *P* = 0.0016; dKO+C9-W33A/S8-R147A vs dKO, *P* = 0.0191). (**I**) Schematic model of C9orf72/SMCR8-mediated lysosomal repair in microglia-associated neuroinflammation. Data information: In (**D**, **F**, **H**), data are presented as mean ± SEM from three independent experiments. Statistical significance was assessed by two-way ANOVA followed by Bonferroni post-test. *P < 0.05, **P < 0.01, ***P < 0.001, ****P < 0.0001; ns, not significant. Source data are available online for this figure.

To establish whether the GAP activity of C9orf72/SMCR8 complex is essential for lysosomal repair, we performed rescue experiments in dKO cells. We first generated key mutations in both proteins based on previous structural and functional studies. The C9orf72 W33A mutation targets a residue in the longin domain critical for RAB8A substrate recognition, while the SMCR8 R147A mutation resides in the catalytic domain and has been demonstrated to abolish the GAP activity of the C9orf72-SMCR8 complex toward RAB8A (Su et al, 2021; Su et al, 2020; Tang et al, 2020; Tang et al, 2023) (Fig. 9A). We then generated lentiviral constructs expressing either wild-type or mutant proteins and confirmed their expression in dKO cells by western blot (Fig. 9B). To assess the functional consequences of GAP activity on lysosomal repair, we first examined GAL3 recruitment to damaged lysosomes. Expression of wild-type C9orf72/SMCR8 complex in dKO cells effectively rescued the accumulation of damaged lysosomes, as evidenced by significantly reduced GAL3-positive lysosomes at 30 min of LLOMe treatment, as well as at 3 h and 6 h post-washout, restoring levels comparable to WT cells (Fig. 9C,D). In contrast, GAP-deficient mutants (C9orf72-W33A and SMCR8-R147A) failed to rescue these defects, with GAL3-positive lysosome levels remaining as elevated as in untransduced dKO cells (Fig. 9C,D). We next examined whether GAP activity is required for proper pT72-RAB8A recruitment to damaged lysosomes. At early time points following LLOMe treatment (10 min and 30 min), expression of wild-type C9orf72/SMCR8 complex significantly increased the percentage of pT72-RAB8A localized to LAMP1-positive lysosomes in dKO cells, restoring recruitment to WT levels (Fig. 9E,F). In contrast, GAP-deficient mutants (C9orf72-W33A and SMCR8-R147A) failed to rescue pT72-RAB8A recruitment (Fig. 9E,F). Furthermore, we assessed whether GAP activity could normalize the aberrant accumulation of pT72-RAB8A puncta observed in dKO cells. Expression of wild-type C9orf72/SMCR8 complex significantly reduced the total number of pT72-RAB8A puncta per cell to WT levels at both 10 min and 30 min of LLOMe treatment (Fig. 9G,H). Conversely, GAP-deficient mutants failed to reduce pT72-RAB8A puncta accumulation, with levels remaining as high as in untransduced dKO cells (Fig. 9G,H). These results demonstrate that both proper RAB8A binding and GAP activity of the C9orf72/SMCR8 complex are essential for efficient lysosomal repair.

## Discussion

Our study identifies the C9orf72/SMCR8 complex as a central regulator of microglial homeostasis by safeguarding lysosomal repair (Fig. 9I). Loss of this complex in mice causes progressive neuroinflammation, microglial activation with DAM/MGnD-like features, and age-dependent neurodegeneration. Mechanistically, we show that C9orf72/SMCR8 acts as a GAP for RAB8A, and that loss of this activity causes accumulation of GTP-bound RAB8A that becomes hyperphosphorylated by LRRK2. Critically, this hyperphosphorylated RAB8A fails to properly localize to damaged lysosomes, instead accumulating on RAB7-positive and LAMP1-negative late endosomes, preventing ESCRT recruitment and membrane repair. These findings establish impaired lysosomal repair as a key mechanism linking C9orf72/SMCR8 deficiency to microglial dysfunction and neuroinflammation.

The age-dependent neuroinflammatory phenotype observed in C9orf72/SMCR8-deficient mice provides insights into how chronic loss of this complex affects microglial function over time. Young (4-month-old) knockout mice showed minimal microglial activation, whereas aged (20-month-old) mice exhibited robust microgliosis, elevated pro-inflammatory cytokines, and significant neuronal loss in hippocampus and spinal cord (Fig. 1), consistent with progressive accumulation of defective lysosomes (Figs. 3, 4, 5, EV1, EV2 and EV3). C9orf72 deficiency dysregulates immune responses across multiple cell types, with early studies demonstrating myeloid expansion, splenomegaly, and systemic inflammation (Atanasio et al, 2016; O’Rourke et al, 2016), later mechanistically linked to enhanced STING-mediated interferon signaling in myeloid cells (McCauley et al, 2020), elevated CD80-mediated costimulation and IL-17A production (Limone et al, 2024). In microglia specifically, C9orf72-deficient patient cells and mouse models show impaired phagocytosis, MMP9-mediated inflammation, disrupted autophagy, and age-dependent synaptic pruning (Banerjee et al, 2023; Lall et al, 2021; Vahsen et al, 2023). However, prior studies examined predominantly younger animals or single gene knockouts rather than complete complex deficiency, precluding assessment of microglial phenotypes during aging when damaged lysosomes accumulate through lipid and protein overload (Choi et al, 2022; Gabandé-Rodríguez et al, 2019; Marschallinger et al, 2020). Systematic comparison revealed that double knockouts exhibited more severe age-dependent neurodegeneration and GAL3 accumulation than single knockouts in vivo (Figs. 1, EV1 and EV2), whereas both genotypes showed comparable repair defects following acute LLOMe-induced lysosomal damage in vitro (Figs. 5 and EV3). Given that single knockouts retained only minimal partner protein, these findings indicate that trace complex levels provide partial protection during chronic stress but are insufficient under acute damage, underscoring the obligate nature of the C9orf72/SMCR8 complex (Amick et al, 2016; Shao et al, 2020; Zhang et al, 2018).

Our demonstration of progressive DAM activation in C9orf72/SMCR8-deficient microglia extends these findings by revealing the underlying organellar basis in the context of aging. snRNA-seq revealed that C9orf72/SMCR8-deficient microglia adopted a DAM-like transcriptional profile characterized by upregulation of *Apoe*, *Axl*, and *Lgals3* (Fig. 2), consistent with the MGnD implicated in other neurodegenerative conditions (Deczkowska et al, 2018; Depp et al, 2025). DAM represent a distinct microglial state characterized by downregulation of homeostatic genes (e.g., P2RY12, CX3CR1) and upregulation of disease-associated genes including APOE, TREM2, and LGALS3, originally identified in Alzheimer’s disease but now recognized as a convergent response across neurodegenerative diseases (Fumagalli et al, 2025; Keren-Shaul et al, 2017). Critically, DAM-like microglia have been identified in SOD1 mutant ALS mouse models and human C9-ALS tissue (Chen et al, 2024). Our aged knockout mice recapitulate key features of this patient phenotype, showing progressive DAM activation that mirrors late-onset disease. We extend these observations by revealing progressive lysosomal membrane damage as the mechanistic basis for DAM activation in this context.

Our findings position C9orf72/SMCR8 deficiency within an emerging paradigm of lysosomal repair failure in neurodegeneration. Multiple neurodegenerative diseases converge on lysosomal quality control defects: Impaired clearance of damaged lysosomes through lysophagy has been linked to neurodegeneration in Niemann-Pick disease (Liu et al, 2020) and α-synuclein propagation in synucleinopathies (Kakuda et al, 2024), while GAL3-positive damaged lysosomes have been documented in microglia from Huntington’s disease patients and mouse models, where GAL3 upregulation precedes motor symptoms and contributes to neuroinflammation (Siew et al, 2019). Additionally, Parkinson’s disease-associated mutations in LRRK2 and VPS35 compromise lysosomal repair (Bentley-DeSousa et al, 2025; Eguchi et al, 2018; Pal et al, 2023). Most direct in vivo evidence comes from recent studies demonstrating constitutive lysosomal membrane damage and defective ESCRT-mediated repair in age-preserved transdifferentiated neurons from Alzheimer’s disease patient fibroblasts, with similar damage phenotypes observed in patient postmortem brain tissue and amyloid mouse models (Chou et al, 2025). These findings underscore that damaged lysosomes are a common pathological feature across multiple neurodegenerative diseases. However, while lysosomal damage has been observed in diverse disease contexts, the molecular mechanisms governing membrane repair—and whether defects in these mechanisms contribute to pathogenesis—remain incompletely understood. Our demonstration of progressive GAL3 accumulation in aged C9orf72/SMCR8-deficient mouse brains and spinal cords provides critical in vivo evidence that lysosomal damage manifests in the nervous system and worsens with age. It will be important to investigate whether C9orf72/SMCR8 participates in lysosomal damage repair across these degenerative disease processes.

Mechanistically, we reveal that C9orf72/SMCR8 operates as a critical regulatory node integrating RAB8A GTPase activity with LRRK2-mediated phosphorylation during lysosomal repair. Previous work established C9orf72/SMCR8 as a RAB8A GAP in ciliogenesis (Tang et al, 2023) and identified LRRK2-mediated RAB8A phosphorylation as a key event in lysosomal damage responses in macrophages(Eguchi et al, 2018; Herbst et al, 2020). We demonstrate that C9orf72/SMCR8 regulates RAB8A in a cilium-independent context in microglia during lysosomal stress. Notably, we did not detect primary cilium formation in BV2 cells following LLOMe treatment (Fig. EV5A), consistent with the non-ciliated nature of microglial cells (Sipos et al, 2018; Sterpka and Chen, 2018). Mutant cells accumulated GTP-bound RAB8A, which was preferentially phosphorylated by LRRK2 (Fig. 8). This resulted in elevated pT72-RAB8A levels in both knockout cell lines after LLOMe treatment and in aged double knockout CNS tissue (Figs. 7D–I and EV4). RAB10, another LRRK2 substrate implicated in lysosomal function (Dhekne et al, 2018; Eguchi et al, 2018), showed no obvious change in phosphorylation status; further studies are needed to determine RAB10’s potential involvement in this lysosomal repair process. Strikingly, this hyperphosphorylated RAB8A fails to properly localize to damaged lysosomes, instead accumulating on RAB7-positive, LAMP1-negative late endosomes, thereby preventing ESCRT recruitment (Fig. 7J-L). This mislocalization distinguishes the C9orf72/SMCR8-deficient state from Parkinson’s disease-associated LRRK2 mutations, which cause excessive RAB8A phosphorylation and sequestration at lysosomes and centrosomes (Madero-Pérez et al, 2018; Mamais et al, 2021). Thus, proper RAB8A function in lysosomal repair requires coordinated regulation of both GTP/GDP cycling and phosphorylation, with disruption of either causing distinct defects.

The C9orf72/SMCR8 complex’s role extends beyond RAB8A to encompass a broader network of small GTPases, positioning it as a master coordinator of organellar homeostasis. Structural studies have demonstrated that the C9orf72/SMCR8 complex possesses GAP activity toward multiple small GTPase substrates, while other studies have reported GEF activity for some substrates (Su et al, 2020; Tang et al, 2020; Sellier et al, 2016; Yang et al, 2016). Together, the complex regulates diverse cellular processes including vesicle trafficking, lysosomal homeostasis, mTORC1 signaling, autophagy, and ciliogenesis (Amick et al, 2020; Lan et al, 2019; McAlpine et al, 2018; Shao et al, 2020; Shi et al, 2018; Tang et al, 2023; Yang et al, 2016; Zhang et al, 2018), likely reflecting coordinated regulation of GTPases at multiple membrane compartments. We found that ARF1, another C9orf72/SMCR8 GAP substrate, did not relocalize to damaged lysosomes following LLOMe treatment, whereas RAB8A was specifically recruited, suggesting context-dependent engagement of specific GTPases during lysosomal repair. How the complex achieves specificity in different cellular contexts—whether through cell-type-specific cofactors, stimulus-dependent regulation, or differential GTPase substrate recognition—remains to be fully elucidated. Beyond their shared activities within the complex, C9orf72 and SMCR8 may also possess GAP complex-independent functions, which could help explain why dKO mice exhibit substantially more severe in vivo phenotypes than either single knockout, particularly in the context of chronic, age-dependent stress that engages multiple parallel pathways beyond acute lysosomal membrane repair.

Lysosomal damage activates multiple coordinated repair pathways (Radulovic et al, 2025). ESCRT complexes mediate membrane remodeling and damage removal (Herbst et al, 2020; Radulovic et al, 2018; Skowyra et al, 2018), membrane contact sites (MCSs) between the endoplasmic reticulum and lysosomes facilitate lipid transfer via ORP proteins and VPS13C (Höglinger et al, 2019; Radulovic et al, 2018; Tan and Finkel, 2022; Wang et al, 2025), and lysosomal damage can trigger stress granule formation that recruits repair machinery to damaged organelles (Bussi et al, 2023). Additional mechanisms include sphingomyelinase and annexin-mediated repair (Niekamp et al, 2022; Yim et al, 2022). While mild lysosomal damage is reversible through these repair mechanisms, severely or irreparably damaged lysosomes are removed through lysophagy or lysosomal exocytosis (Domingues et al, 2024; Liu et al, 2020; Sho et al, 2024). RAB8A dysregulation in C9orf72/SMCR8-deficient cells could affect multiple aspects of this repair network, and whether C9orf72/SMCR8 coordinates both repair and clearance pathways warrants further investigation. MCSs coordinate proteostasis, lipid metabolism, and organellar quality control across neurodegenerative diseases (Calì et al, 2025; Vrijsen et al, 2022). Intriguingly, RAB8A has been reported to localize to lipid droplet-mitochondria contact sites (Ouyang et al, 2023), yet we did not observe pT72-RAB8A recruitment to mitochondria or lipid droplets following lysosomal damage, suggesting that C9orf72/SMCR8-regulated RAB8A functions specifically in endolysosomal repair. Additional organellar contacts, including mitochondria-lysosome, lipid droplet-lysosome, and other multi-organelle MCSs, may also contribute to lysosomal damage sensing and repair, though their roles—particularly in neurodegenerative disease contexts (Calì et al, 2025) and in regulating microglial functional states—remain incompletely characterized. Direct investigation of these contacts during lysosomal damage is an important future direction.

In conclusion, our findings establish impaired lysosomal repair as a key mechanism linking C9orf72/SMCR8 deficiency to microglial dysfunction, thereby providing a mechanistic framework that may contribute to our understanding of neuroinflammation in ALS and FTD. By uncovering disrupted ESCRT recruitment and aberrant RAB8A dynamics, we extend the functional repertoire of the C9orf72/SMCR8 complex beyond its previously known roles. These findings advance our understanding of C9orf72/SMCR8 function in microglia and suggest that targeting lysosomal repair pathways or RAB8A regulation could represent potential therapeutic strategies for conditions involving microglial dysfunction and lysosomal pathology.

## Methods

**Table:** Reagents and tools table

Reagent/resource	Reference or source	Identifier or catalog number
**Experimental models**		
C57BL/6J *C9orf72* ^−/−^ mice	Jackson	027068
C57BL/6 J *Smcr8* ^−/−^ mice	Gift from Dr. Jianfu. Chen	N/A
BV2 cells	Gift from Dr. Qun Lu	N/A
HEK 293 T cells	Gift from Dr. Jianwei. Sun	N/A
Primary microglia (postnatal mouse brains)	Primary Microglia Isolation from Postnatal Mouse Brains	N/A
**Recombinant DNA**		
pVSVG	Gift from Dr. Jianwei Sun	N/A
psPAX2	Gift from Dr. Jianwei Sun	N/A
pLenti-CRISPRv2	Gift from Dr. Jianwei Sun	N/A
pLenti-EGFP-hRAB8A	This study	N/A
pLenti-EGFP-hRAB8A Q67L	This study	N/A
pLenti-EGFP-hRAB8A T22N	This study	N/A
pLenti-EGFP-mRAB7	This study	N/A
pEGFP- C1-RAB5	Gift from Dr. Chonglin Yang	N/A
pEGFP- C1-RAB11	Gift from Dr. Chonglin Yang	N/A
pLenti-EGFP-mSMCR8	This study	N/A
pLenti-EGFP-mC9orf72	This study	N/A
pLenti-TMEM192-3×HA-Blasticidin	This study	N/A
pLenti-mC9orf72(W33A)-P2A-GFP	This study	N/A
pLenti-mSmcr8(R147A)-P2A-GFP	This study	N/A
pFastBac-hLRRK2 (RCKW1327–2527)-HIS	This study	N/A
pGEX-4T-GST-hRAB8A	This study	N/A
pGEX-4T-GST-hRAB8A Q67L	This study	N/A
pGEX-4T-GST-hRAB8A T22N	This study	N/A
pGEX-4T-GST-hOCRL-(RBD1618 ~ 2709)	This study	N/A
AAV-mGalectin 3-EGFP	This study	N/A
pCMV-HA-MICALL2	MIAOLING BIOLOGY	P4955
**Antibodies**		
C9orf72	Proteintech	25757-1-AP
SMCR8	GeneTex	GTX635798
SMCR8	BETHYL	A304-694A-M
Galectin 3	Proteintech	14979-1-AP
Galectin 8	GeneTex	GTX33214
Galectin 9	Abcam	ab69630
ALIX	HUABIO	JM85-31
CHMP2B	Abcam	ab157208
RAB8A (phospho T72)	Abcam	ab230260
RAB8A	HUABIO	HA721127
GAPDH	Proteintech	60004-1-Ig
β-Actin	Proteintech	66009-1-Ig
α-Tubulin	Proteintech	66031-1-Ig
Choline Acetyltransferase	Abcam	ab178850
IBA1	DAKO	019-19741
GFP	AVES	GFP-1010
NeuN	MilliporeSigma	MAB377
Cathepsin D	R&D Systems	AF1029-SP
CD68	Abcam	ab53444
Acetyl-Tubulin (Lys40)	Proteintech	66200-1-Ig
LAMP1	DHSB	AB-528127
GM130	BOSTER	M05865-2
TOMM20	HUABIO	HA601454
HA	SIGMA	H3663
LRRK2	Abcam	ab133474
LRRK2 (phospho S935)	Abcam	ab133450
HRP-Goat anti-Mouse IgG (H + L)	ZSGB-BIO	ZB-2305
HRP-Goat anti-Rabbit IgG (H + L)	ZSGB-BIO	ZB-2306
HRP-Goat anti-Rat IgG (H + L)	ZSGB-BIO	ZB-2307
Alexa Fluor 488 Goat anti-Rabbit IgG (H + L)	Jackson	111-545-003
Alexa Fluor 555 Goat anti-Rabbit IgG (H + L)	Thermo Fisher Scientific	A-21428
Alexa Fluor 488 Goat anti-Mouse IgG1	Thermo Fisher Scientific	A-21121
Alexa Fluor 555 Goat anti-Mouse IgG1	Thermo Fisher Scientific	A-21127
Alexa Fluor 647 Goat anti-Mouse IgG1	Thermo Fisher Scientific	A-21240
Alexa Fluor 488 Donkey anti-Chicken IgG (H + L)	Jackson	703-545-155
Alexa Fluor 647 Goat anti-Rat IgG (H + L)	Jackson	112-605-003
Alexa Fluor 488 Goat anti-Mouse IgG (H + L)	Jackson	115-545-003
**Oligonucleotides and other sequence-based reagents**		
sgRNA sequences	This study	Appendix Table 1
qPCR primers	This study	Appendix Table 2
**Chemicals, enzymes and other reagents**		
BODIPY™ 493/503	Thermo Fisher Scientific	D3922
LysoTracker™ Red DND-99	Thermo Fisher Scientific	L7528
PBS buffer	Gibco	C10010500BT
DMEM	Gibco	C11005500BT
DMEM/F-12	Gibco	C11330500BT
FBS	Sigma	12003C
FBS	Vivacell	C04001
Penicillin–Streptomycin	Vivacell	C3420
Trypsin EDTA Solution A	Vivacell	C3530
Glial-Mag kit	OZ Biosciences	GL00250
Polyethylenimine (PEI)	YEASEN	40820
Puromycin	Thermo Fisher Scientific	AC227420100
Blasticidin S HCl	Beyotime	ST018
Protease inhibitor cocktail	MCE	HY-K0011
Phosphatase inhibitor cocktail	MCE	HY-K0021
Dounce homogenizer (B pestle)	Sigma	D8938
Pierce BCA Protein Assay kit	BIOMIKY	MK043A
Mini-PROTEAN® TGX™ Precast Protein Gels	BioRad	4561094
PVDF membrane	Millipore	IPFL00010
Ultra High Sensitivity ECL Kit	SINSAGE	BL520B
Glutaraldehyde	SPI Supplies	02607-BA
Osmium tetroxide	SPI Supplies	02602-AB
Uranyl acetate	SPI Supplies	02624-AB
EMBED-812	Electron Microscopy Sciences	14900
NMA	Electron Microscopy Sciences	19000
DDSA	Electron Microscopy Sciences	13710
DMP-30	Electron Microscopy Sciences	13600
Lead citrate	Alfa Aesar	A10701
Papain	Sigma	P5306
DNase I	Sigma	DN25
GM-CSF	MCE	HY-P7361
Poly-D-lysine	Sigma	P2636
4,6-diamidino-2-phenylindole (DAPI)	CST	4083S
Anti-fade mounting medium	Beyotime	P0126
Anti-HA magnetic beads	Thermo Fisher Scientific	88836
Glutathione Beads	Smart-Lifesciences	SA008005
Ni NTA Beads	Smart-Lifesciences	SA004005
LysoTracker Red DND-99	Invitrogen	L7528
ADP-Glo Kinase Assay	Promega	V6930
GDP	MCE	HY-113066
BSA	Sigma	B2064
GFP-TrapⓇ Magnetic Agarose	Chromotek	gtma-20
Paraformaldehyde	Sigma	158127
Trizol Up RNA extraction reagent	TransGen Biotech	ET111
Reverse transcription kit	TransGen Biotech	AU341
PerfectStart SYBR Green Supermix	TransGen Biotech	AQ601
Protein Marker	YEASEN	20350ES
L-Leucyl-L-Leucine methyl ester hydrochloride (LLOMe)	MCE	HY-129905
Phanta® Super-Fidelity DNA Polymerase	Vazyme	P505
**Software**		
Fiji/ImageJ	https://imagej.net/software/fiji/	
DNBC4TOOLS	https://github.com/MGI-tech-bioinformatics/DNBelab_C_Series_HT_scRNA-analysis-software?tab=readme-ov-file/	
GraphPad Prism	https://www.graphpad.com/	
R	https://cloud.r-project.org/	
RStudio	https://posit.co/download/rstudio-desktop/	
SnapGene	https://www.snapgene.com/	
CHARMM-GUI	https://charmm-gui.org/	
GROMACS	https://www.gromacs.org/	
Imaris 10.1.0	https://imaris.oxinst.com/	
**Other**		
N/A	N/A	N/A

### Ethical statement

All animal experiments were conducted in compliance with the ethical guidelines and approval from the Institutional Animal Care and Use Committee (IACUC) of Yunnan University (Protocol number: YUNCARE20200341).

### Animals

*C9orf72* knockout mice (Jackson Laboratory, stock #027068) and *Smcr8* knockout mice (as described in Shao et al, 2020) were maintained on a C57BL/6 J background. Double knockout mice were generated by intercrossing *C9orf72*^*−/−*^ and *Smcr8*^*−/−*^ lines. All mice were housed in pathogen-free barrier facilities under a 12-h light/dark cycle at 21 ± 2 °C and 40–60% humidity. Age-matched wild-type, heterozygous and double knockout littermates ranging from 3 to 24 months were used for experimental analyses. Euthanasia was performed by CO_2_ inhalation followed by cervical dislocation according to IACUC guidelines.

### snRNA-seq samples and data processing

snRNA‑seq was performed on pooled brain samples from 20‑ to 24‑month‑old mice, with three WT and three dKO brains pooled separately to generate sequencing libraries using the BGI platform. The raw data were aligned to the mouse genome GRCm39 using DNBC4TOOLS (v2.1.3). The data analysis and visualization were carried out using the Seurat R package (v5.0) (Hao et al, 2024). Cells were filtered based on the following criteria: 100 < nFeatures < 7500 and 200 < nCountFeatures <  30000. Cells with percent.mt >20 were further excluded. The data were normalized using NormalizeData function with default parameters. The data were then scaled by using ScaleData function with vars.to.regress = c(“percent.mt”, “percent.Rpsl”, “percent.Hb”). The data were then integrated using IntegrateLayers function with method = HarmonyIntegration. Unsupervised clustering was then performed by FindNeighbors function with dims = 1:30 and FindClusters function with resolution = 0.05. Cells were annotated using a published mouse brain transcriptomic atlas (Yao et al, 2021). A total of 33,257 cells were obtained, including 14,460 excitatory neurons, 9379 inhibitory neurons, 6267 oligodendrocytes, 1912 microglia, 832 astrocytes, and 407 epithelial cells. DEGs between dKO and WT conditions within each cell-type were identified using the FindMarkers function with default parameters. Functional enrichment analysis of Kyoto Encyclopedia of Genes and Genomes (KEGG) (Ogata et al, 1999) and GO (Ashburner et al, 2000) were performed using the clusterProfiler package (v4.10.1) (Wu et al, 2021; Yu et al, 2010; Yu et al, 2012). In brief, the enrichGO function was utilized to identify enriched GO terms with (ont = “BP”/“MF”/“CC”, pvalueCutoff = 0.05, qvalueCutoff = 0.2) and then the similar GO terms were merged based on semantic similarity using the simplify function with cutoff = 0.7. The KEGG enrichment analysis was performed using enrichKEGG function with pvalueCutoff = 0.05, qvalueCutoff = 0.2, minGSSize = 5.

### Immunostaining of sections and cultured cells

Mice aged 4 to 24 months were anesthetized with CO₂ and transcardially perfused with ice-cold PBS, followed by perfusion-fixation with 4% paraformaldehyde (PFA) in PBS. Brains were collected and post-fixed in 4% PFA at 4 °C for 24 h. After washing with PBS, tissues were cryoprotected in 30% sucrose for 2 days and then embedded in OCT. Coronal sections (15 µm) were prepared using an HM525 NX cryostat (Thermo Fisher Scientific). Sections were washed three times in PBS (5 min each) and incubated in blocking buffer (2% normal goat serum, 1% BSA, 0.1% Triton X-100 in PBS) for 1 h at room temperature. Subsequently, sections were incubated with primary antibodies diluted in blocking buffer overnight at 4 °C. After three PBS washes, sections were incubated with secondary antibodies in blocking buffer for 1 h at room temperature. Nuclei were counterstained with DAPI, and sections were mounted with antifade mounting medium. Once dried, slides were stored at 4 °C until imaging.

For immunostaining of cultured cells, coverslip-seeded cells were fixed with freshly prepared 4% PFA for 10 min at room temperature and washed three times with PBS. Cells were permeabilized with 0.1% Tween-20 for 10 min, washed, and then blocked with 2% normal goat serum and 1% BSA in PBS for 1 h at room temperature. Cells were incubated with primary antibodies in blocking buffer overnight at 4 °C, washed three times with PBS, and incubated with secondary antibodies in blocking buffer for 45 min at room temperature. After final PBS washes, coverslips were mounted in antifade medium. Images were acquired using a Zeiss LSM 800 confocal laser scanning microscope.

### Cells, transfection, and reagents

BV2 cells (kindly provided by Dr. Qun Lu) and 293 T cells (provided by Dr. Jianwei Sun) were cultured at 37 °C with 5% CO_2_ in DMEM supplemented with 10% FBS (Vivacell), 100 U/mL penicillin, and 100 mg/mL streptomycin. Transfections for BV2 cells were performed using the Glial-Mag kit (Oz Biosciences) according to the manufacturer’s instructions. Briefly, 1 × 10^5^ BV2 cells were plated on an 18 mm coverslip in a 24-well plate, with a ratio of 2.5 μL Glial-Mag to 1 μg DNA. 250 μL Glial-Boost was added to the well and incubated for 1 h. The transfection mix was then removed, and imaging was captured, or immunostaining was performed after 24 h. Transfections for 293T cells were performed using Polyethylenimine (PEI 40 K, MX2203), with a ratio of 4 μL PEI to 1 μg DNA.

### Generation of *C9orf72*, *Smcr8* single and double KO cell lines

The CRISPR/Cas9 technology was used to knock out *C9orf72* and *Smcr8* genes in mouse BV2 cell line. First, the complete sequence information of mouse *C9orf72* and *Smcr8* genes was obtained from the NCBI website to determine the sgRNA targeting sequences. The first exon was chosen as the target fragment for designing sgRNA to ensure that only the long isoform of the C9orf72 gene and all isoforms of the Smcr8 gene were knocked out. The sgRNA sequences for *C9orf72* and *Smcr8* were designed using the Broad Institute’s CRISPick tool (https://portals.broadinstitute.org/gppx/crispick/public). The sgRNA sequences (Appendix Table S1) were synthesized with compatible sticky ends cloned into lentiCRISPRv2 vectors for lentiviral packaging. For lentiviral transduction, BV2 cells were seeded in six-well plates and infected with lentivirus containing either *C9orf72*-sgRNA or *Smcr8*-sgRNA. Infected cells were selected with puromycin (2 μg/mL) for 5–7 days. A portion of the cells was used to detect the gene knockout efficiency by Western Blot. The cells with better knockout efficiency were sorted into single cells using flow cytometry for further experiments. The knockout of *C9orf72* and *Smcr8* genes in the selected single-cell clones was confirmed by Western Blot and DNA sequencing. To generate *C9orf72*/*Smcr8* double knockout cells, validated *Smcr8* KO clones were infected with the *C9orf72*-sgRNA lentivirus, followed by single-cell cloning, and the double knockout was confirmed by Western Blot and DNA sequencing.

### Quantitative real-time PCR (qRT-PCR)

Total RNA was extracted using TRIzol reagent (Invitrogen) and reverse transcribed into cDNA using the TransScript All-in-One First-Strand cDNA Synthesis SuperMix for qPCR (TransGen Biotech) with 1 μg RNA as template. Quantitative PCR was performed using SYBR Green qPCR Master Mix (TransGen Biotech) on a Real-Time PCR System (BioRad). Each 10 μL reaction contained 5 μL of 2× qPCR Mix, 22 ng or 100 ng cDNA template, 0.3 μL each of forward and reverse primers (10 μM), and nuclease-free water. Cycling conditions were: 95 °C for 5 min, followed by 40 cycles of 95 °C for 15 s and 60 °C for 30 s. Relative gene expression was calculated using the 2^(-ΔΔCt) method with *Actb* as reference. Primer sequences are listed in Appendix Table S2.

### Lysosomal immunoprecipitation (Lyso-IP) and immunoblot

Lyso IP immunoblotting assay was performed according to previously described protocols (Abu-Remaileh et al, 2017). To establish Lyso IP stably expressing cell line, the pLenti-TMEM192-3×HA-Blasticidin construct was packaged into lentiviral particles combination with pCMV-VSVG and psPAX2 packaging plasmids in HEK293T cells. BV2 *C9orf72* knockout and *Smcr8* knockout cells were seeded in 6-cm culture dish and infected with 700 µL of TMEM192-3×HA virus. Twenty-four hours post-infection, 6 µg/mL Blasticidin S HCl was added for selection. Following expansion and counting, 8.0–8.5 × 10⁶ cells were seeded in 10 cm culture dishes. After cells reached ~85% confluency. Cells were washed twice with pre-cold 1× PBS and once with pre-cold KPBS buffer (136 mM KCl,10 mM KH₂PO₄, pH 7.25 adjusted with KOH). Subsequently, 500 µL KPBS supplemented with protease and phosphatase inhibitors was added, and cells were gently scraped on ice. Cell suspensions were gently homogenized for 120 strokes use a 2 mL Dounce homogenizer (B pestle) (D8938, Sigma-Aldrich) until ~80% trypan blue-positive cells were observed. The lysate was centrifuged at 3000 rpm for 2 min at 4 °C to remove pellet nuclei and cellular debris. The supernatant was incubated with 80 µL KPBS prewashed anti-HA magnetic beads (88836, Thermo Fisher) at 4 °C for 15 min with gentle rotation. Beads were washed five times with KPBS, then resuspended in 50 µL of 1× Laemmli sample buffer, and boiled at 95 °C for 10 min. Eluted proteins were analyzed by western blotting.

### Protein extraction and immunoblotting analysis

Cells were collected by centrifugation at 300× *g* for 4 min at 4 °C and washed twice with cold PBS. Cell lysates for western blot were prepared using protein lysis buffer (50 mM Tris-HCl, pH 7.5, 150 mM NaCl, 1% Triton X-100, 1 mM EDTA) supplemented with protease inhibitor cocktail (MCE) and incubated on ice for 30 min. After centrifugation at 17,000 g for 20 min at 4 °C, cleared supernatant was transferred to new clean tubes, and total protein concentration was measured using BCA Protein Assay kit (Pierce). Protein samples were boiled for 10 min at 95 °C in 1xSDS sample buffer with β-mercaptoethanol (1610747, BioRad), resolved on 10% polyacrylamide gels or pre-cast 4–15% Mini-PROTEAN^®^ TGX™ Precast Protein Gels (#4561094), and transferred to a PVDF membrane (IPFL00010; Millipore) using a wet transfer module (BIO-RAD Mini-PROTEAN Tetra System). Membranes were blocked in 5% non-fat milk powder, and resuspended in PBS-T for 30 min at room temperature, followed by incubation with primary antibodies diluted in PBST 5% milk overnight at 4 °C. After washing with PBST, membranes were developed using Ultra High Sensitivity ECL Kit (BL520B). Western blotting images were acquired by SINSAGE smartchemi 610 and the grayscale value of protein bands was quantified using ImageJ software.

### Immunoprecipitation and GST pull-down

For immunoprecipitation experiments, BV2 cells were either left untreated (basal) or treated with 1 mM LLOMe for 30 min, washed with PBS, and lysed in lysis buffer (20 mM Tris-HCl pH 7.4, 150 mM NaCl, 0.5% NP-40, Protease Inhibitor Cocktail, Phosphatase Inhibitor Cocktail). For GFP-based IP, cells expressing GFP-tagged proteins (C9orf72, SMCR8, RAB8A, or RAB8A mutants Q67L/T22N/T72A) were incubated with GFP-trap beads (ChromoTek) overnight at 4 °C. For MICALL2 competitive binding experiments, 293T cells were co-transfected with GFP-RAB8A and HA-MICALL2 or empty vector, and GFP-based IP was performed using GFP-trap beads. For RAB8A IP-MS, WT and dKO BV2 cells were subjected to Dounce homogenization, centrifuged at 1000 rpm for 3 min, and lysates were incubated with anti-RAB8A antibody (HUABIO, HA721127) followed by Protein A/G beads. All beads were washed five times with wash buffer (20 mM Tris-HCl pH 7.4, 150 mM NaCl, 0.5% NP-40, 1 mM PMSF) and analyzed mass spectrometry (Yunnan University).

For GST pull-down, lysates from WT, *C9orf72* KO, *Smcr8* KO, and *dKO* cells (basal or LLOMe-treated) were incubated with GST-OCRL immobilized on glutathione Sepharose beads overnight at 4 °C, washed, and analyzed by western blotting.

### Kinase activity assay

The kinase activity assay was performed using the ADP-Glo™ Kinase Assay kit (Promega, V6930) according to the manufacturer’s protocol. In total, 5 μg recombinant LRRK2 protein were incubated with 9 μg substrates [WT-RAB8A, GTP-bound RAB8A (Q67L) and GDP-bound RAB8A (T22N)] in reaction buffer. After in vitro kinase assay, ADP-Glo reagent and kinase detection reagent was added to terminate the reaction and deplete residual ATP. The generated ADP is subsequently converted to ATP, and the newly formed ATP is quantified by chemiluminescence.

### Transmission electron microscopy

BV2 cells or primary microglia were grown in 24-well plates and processed for transmission electron microscopy as follows. Cells were fixed with PBS containing 2.5% glutaraldehyde (02607-BA, SPI Supplies) at 4 °C and washed with PBS. Samples were post-fixed in 1% osmium tetroxide (OsO₄, 02602-AB, SPI Supplies) in distilled water at 4 °C and subsequently rinsed with distilled water. En bloc staining was performed at room temperature with 2% aqueous uranyl acetate (02624-AB, SPI Supplies), followed by distilled water washes. Samples were dehydrated through a graded ethanol series, then infiltrated using a graded series of EMBED-812 resin diluted in anhydrous ethanol (EMBED-812, 14900, NMA, 19000, DDSA, 13710, Electron Microscopy Sciences). After overnight infiltration in 100% resin at room temperature, samples were further infiltrated with 100% resin containing the accelerator DMP-30 (13600, Electron Microscopy Sciences), embedded in molds, and polymerized at 60 °C. Ultrathin sections ( ~ 70 nm) were cut on an ultramicrotome (EM UC7, Leica Biosystems) and collected on copper grids. Sections were post-stained with 2% aqueous uranyl acetate and then with 0.2% lead citrate (A10701, Alfa Aesar), rinsed with distilled water and air-dried. Specimens were examined using a transmission electron microscope (HT7800, Hitachi) operated at an accelerating voltage of 80 kV.

### Primary microglia isolation

Primary microglia were isolated following the procedure described previously (Du et al, 2021). Postnatal mice (P0-P1) were anesthetized by placing them on ice for 30–50 s. After sterilization with 75% ethanol, all pups were quickly decapitated with surgical scissors and put in the ice-cold 1× PBS. Meanwhile, rinse all heads with ice-cold 1× PBS to remove excess blood. Transfer the heads to a new plate containing 2 mL of pre-cold 1× PBS. The olfactory bulbs and cerebellum were discarded, and the remaining brain tissue was gently transferred to a chilled 6-well plate containing 0.5 mL of pre-cold 1× PBS. The brain tissue was finely minced into 1–2 mm³ pieces with the help of spring scissors. Tissues were incubated in digestion buffer (DMEM containing 8 U/mL papain [P5306, Sigma] and 125 U/mL DNase I [DN25, Sigma]) at a ratio of 0.5 mL digestion buffer per brain tissue, at 37 °C for 20 min with gentle agitation every 5 min. The papain digestion was terminated by adding 3 mL of pre-warmed complete culture medium (DMEM/F12 supplemented with 10% fetal bovine serum and 1% penicillin–streptomycin). The digested tissue was gently triturated ten times using a 1 mL pipette. Let it settle for 1 min. Carefully pass the cell suspension through a pre-wetted 70 µm cell strainer and collect the flow-through in the 50 mL collection tube. Repeat this step until all the big clumps have been removed. The cell suspension was centrifuged at 200×*g* for 10 min at room temperature, and then discard the remaining cell debris. Primary cells were resuspended in complete culture medium supplemented with 2 ng/mL GM-CSF (HY-P7361, MCE) and seeded in culture dishes pre-coated with 0.1 mg/mL poly-D-lysine (P2636, SIGMA). On the second day, wash the dishes with pre-warmed PBS to remove non-adherent cells and debris. Fresh medium containing 2 ng/mL GM-CSF was added, and cells were maintained for 4–7 days before microglia were harvested for the desired functional assay.

### Microglia morphology analysis

Microglial morphology was analyzed using three-dimensional surface reconstruction in Imaris software (v10.1, Bitplane). Z-stack images of IBA1-stained spinal cord sections were acquired on a confocal microscope (×63 oil objective, 1 µm z-step, total stack depth 10 μm). Individual microglia were reconstructed using the Surface module with automatic threshold detection. Microglial processes were reconstructed as tree-like structures originating from the soma. Branch points were defined as sites where a single process bifurcates into two or more daughter branches, and the total number of branch points per cell was calculated. Total process length per cell was obtained by summing the maximum branch length of all processes from an individual cell. Branch points and total process length were quantified for 40–45 cells per genotype from *n* = 3 mice per group.

### Graphics

The graphics in Fig. 9I, the synopsis and Fig. EV4O were created with BioRender.com.

### Statistics

Data derived from different genotypes or different treatments were analyzed using Student’s two-tailed unpaired *t* test, one-way ANOVA followed by Tukey’s multiple comparison test or two-way ANOVA followed by Bonferroni post-test for multiple comparisons. Data are represented as mean ± standard error of the mean (SEM). A value of *P*  <  0.05 was considered significant (*P  <  0.05; **P  <  0.01; ***P  <  0.001; ****P < 0.0001).

## Supplementary information

Appendix

Peer Review File

Source data Fig. 2

Source data Fig. 3

Source data Fig. 4

Source data Fig. 5

Source data Fig. 6

Source data Fig. 7

Source data Fig. 8

Source data Fig. 9

Figure EV2 Source Data

Figure EV3 Source Data

Figure EV4 Source Data

Figure EV5 Source Data

Expanded View Figures

## Data Availability

The snRNA-seq reads were deposited in National Genomics Data Center (https://ngdc.cncb.ac.cn) with the BioProject accession number PRJCA055728. Source data for Figs. 1 and EV1 were deposited to BioImage Archive under accession codes S-BSST2788 and S-BSST2792, respectively. Source data for all other figures are available as source data files linked to each figure in the online version of this article. The source data of this paper are collected in the following database record: biostudies:S-SCDT-10_1038-S44318-026-00817-w.
