# Supplementary material for: The C9orf72/SMCR8 complex maintains microglial homeostasis via RAB8A-ESCRT-mediated lysosomal repair
Source: EMBO J. 2026 May 29;45(13):4531–68. doi: 10.1038/s44318-026-00817-w (PMC13324726; doi:10.1038/s44318-026-00817-w)
Supplement: Supplementary file 1 — Appendix [file 44318_2026_817_MOESM1_ESM.pdf]

Appendix for:

The C9orf72/SMCR8 complex maintains microglial homeostasis via RAB8A ESCRT-mediated lysosomal repair

Table of Contents:

Appendix Table S1 Page 2

Appendix Table S2 Page 3

Appendix Table S1. Primer sequences for sgRNA.

Primer	Sequence (5'-3')
C9orf72-sgRNA-2F	CACCGCACACACTCTGTGAAGTGGG
C9orf72-sgRNA-2R	AAACCCCACTTCACAGAGTGTGTGC
Smcr8--sgRNA-2F	CACCGTGATGTGGTGGCCTTCACCA
Smcr8--sgRNA-2R	AAACTGGTGAAGGCCACCACATCAC

Appendix Table S2. Primer sequences for qRT-PCR.

Primer	Sequence (5'-3')
Actin-Mouse qPCR F	ACGAGGCCCAGAGCAAGAG
Actin-Mouse qPCR R	TCTCCAAGTCGTCCCAGTTG
C9orf72-Mouse qPCR F	TTGGCGGCTACCTTTGCTTAC
C9orf72-Mouse qPCR R	CATTCCAGTTTCCGTCGAAGA
Smcr8-Mouse qPCR F	TCCTCATTTCCGAGTTCTCTGA
Smcr8-Mouse qPCR R	CACCGACATAATCCGCAAAGA
Tnf -Mouse qPCR F	CCCTCACACTCAGATCATCTTCT
Tnf -Mouse qPCR R	GCTACGACGTGGGCTACAG
Il1b-Mouse qPCR F	GAAATGCCACCTTTTGACAGTG
Il1b-Mouse qPCR R	TGGATGCTCTCATCAGGACAG
Il6-Mouse qPCR F	TAGTCCTTCCTACCCCAATTTCC
Il6-Mouse qPCR R	TTGGTCCTTAGCCACTCCTTC
Apoe-Mouse qPCR F	CTGACAGGATGCCTAGCCG
Apoe-Mouse qPCR R	CGCAGGTAATCCCAGAAGC
Csf1-Mouse qPCR F	GGCTTGGCTTGGGATGATTCT
Csf1-Mouse qPCR R	GAGGGTCTGGCAGGTACTC
Trem2-Mouse qPCR F	CTGGAACCGTCACCATCACTC
Trem2-Mouse qPCR R	CGAAACTCGATGACTCCTCGG
Casp1-Mouse qPCR F	CTATGGACAAGGCACGGGAC
Casp1-Mouse qPCR R	TCAGCTGATGGAGCTGATTGA
Ptgs2-Mouse qPCR F	TTCAACACACTCTATCACTGGC
Ptgs2-Mouse qPCR R	AGAAGCGTTTGCGGTACTCAT
Axl-Mouse qPCR F	ATGGCCGACATTGCCAGTG
Axl-Mouse qPCR R	CGGTAGTAATCCCCGTTGTAGA
Cxcl10-Mouse qPCR F	CCAAGTGCTGCCGTCATTTTC
Cxcl10-Mouse qPCR R	GGCTCGCAGGGATGATTTCAA
Irf7-Mouse qPCR F	TCTCACCGAGCGCAGC
Irf7-Mouse qPCR R	TGCAGCTGCTTCTGATCTGG
Gal3-Mouse qPCR F	CTGGCCCTTATGGTGTCCCC
Gal3-Mouse qPCR R	AGCGGGGGTTAAAGTGGAAGG
Gpnmb-Mouse qPCR F	GCTGGTCTTCGGATGAAAATGA
Gpnmb -Mouse qPCR R	CCACAAAGGTGATATTGGAACCC
