# Supplementary figures and images for: The C9orf72/SMCR8 complex maintains microglial homeostasis via RAB8A-ESCRT-mediated lysosomal repair (part 1 of 3)
Source: EMBO J. 2026 May 29;45(13):4531–68. doi: 10.1038/s44318-026-00817-w (PMC13324726; doi:10.1038/s44318-026-00817-w)

Brain

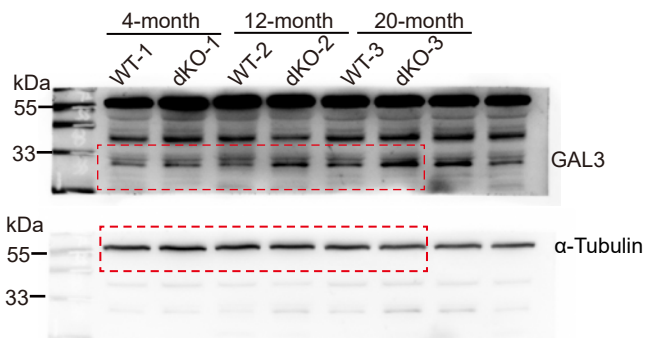

Spinal cord

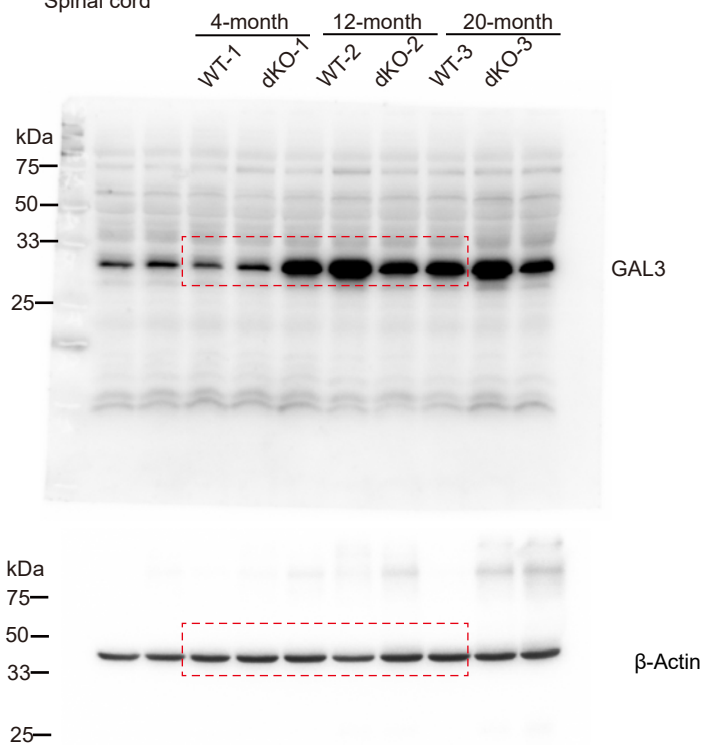

Supplement: Supplementary file 4 — Source data Fig. 3 [file 44318_2026_817_MOESM4_ESM.zip › 3A/3A.pdf]

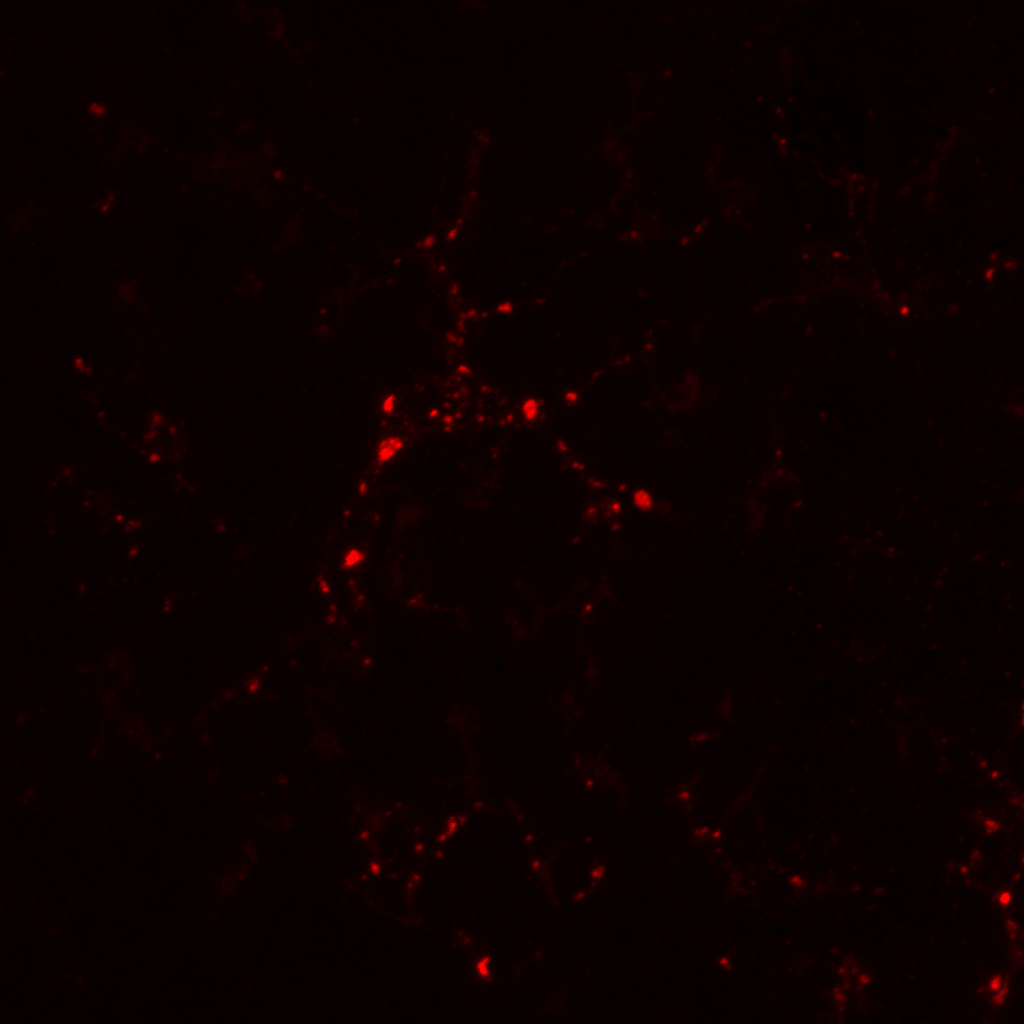

Supplement: Supplementary file 4 — Source data Fig. 3 [file 44318_2026_817_MOESM4_ESM.zip › 3D/3D-1-WT_CD68.tif]

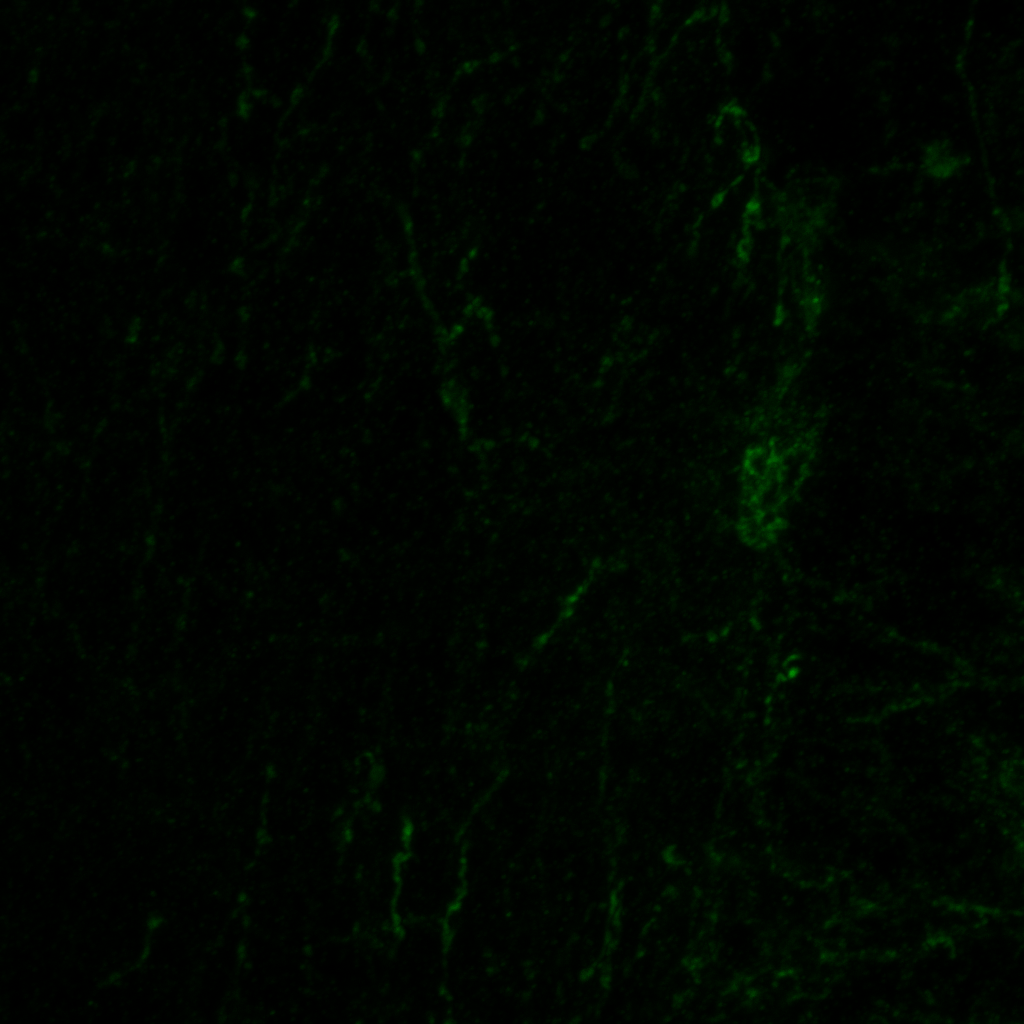

Supplement: Supplementary file 4 — Source data Fig. 3 [file 44318_2026_817_MOESM4_ESM.zip › 3D/3D-1-WT_GAL3.tif]

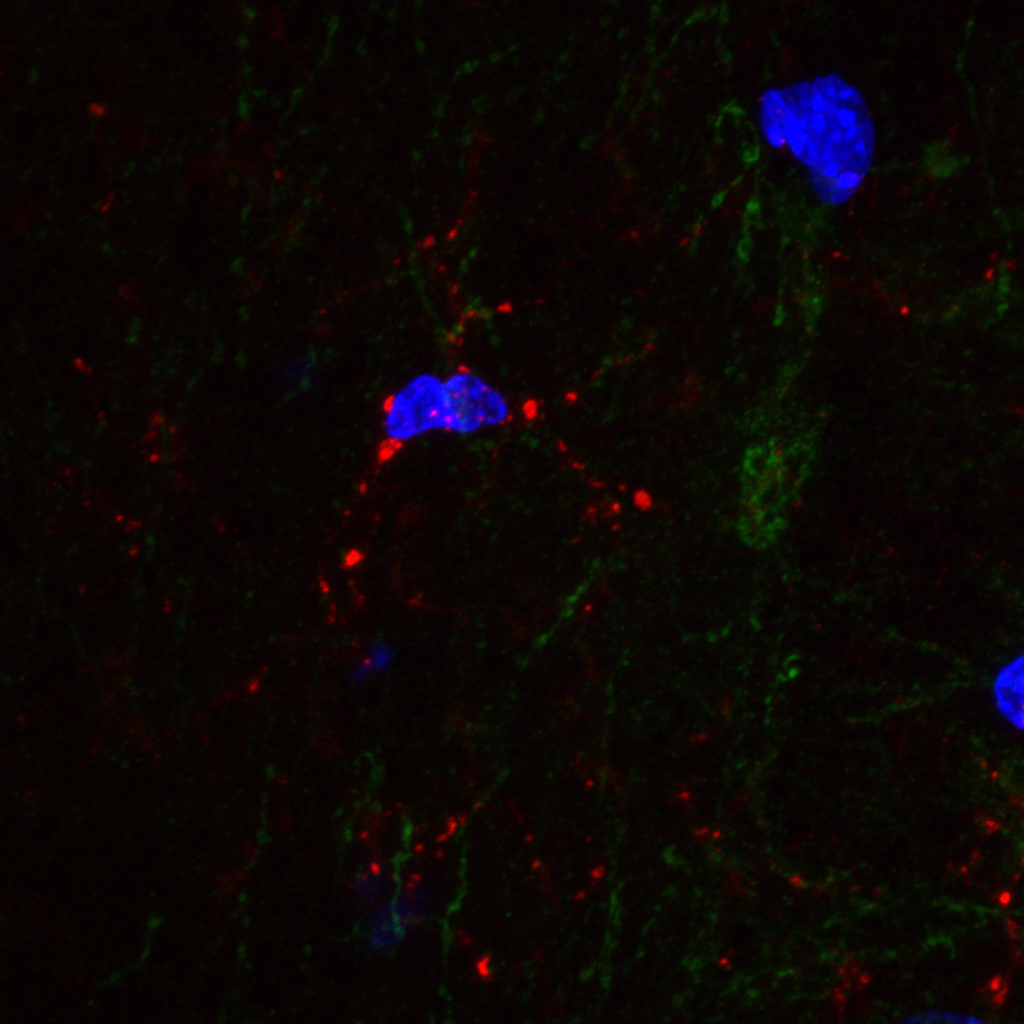

Supplement: Supplementary file 4 — Source data Fig. 3 [file 44318_2026_817_MOESM4_ESM.zip › 3D/3D-1-WT_Merge.tif]

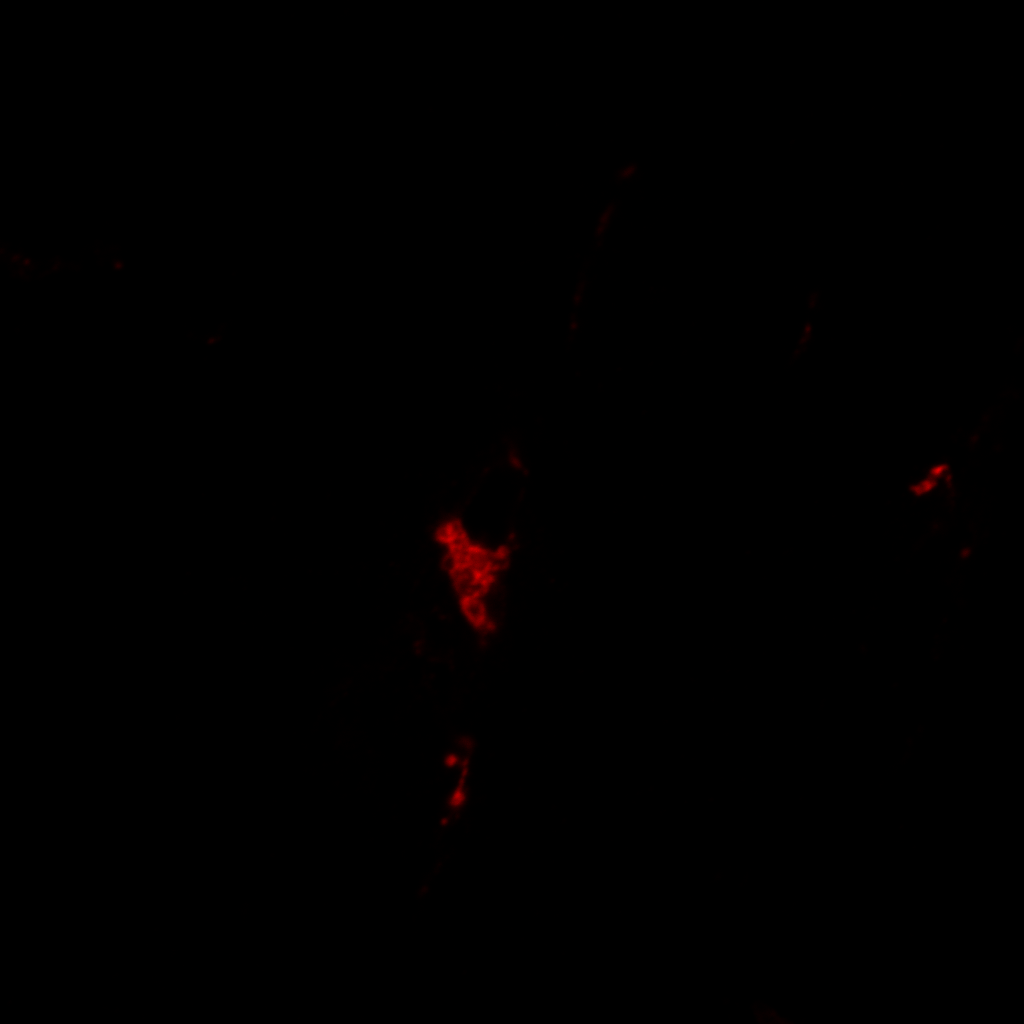

Supplement: Supplementary file 4 — Source data Fig. 3 [file 44318_2026_817_MOESM4_ESM.zip › 3D/3D-2-dKO_CD68.tif]

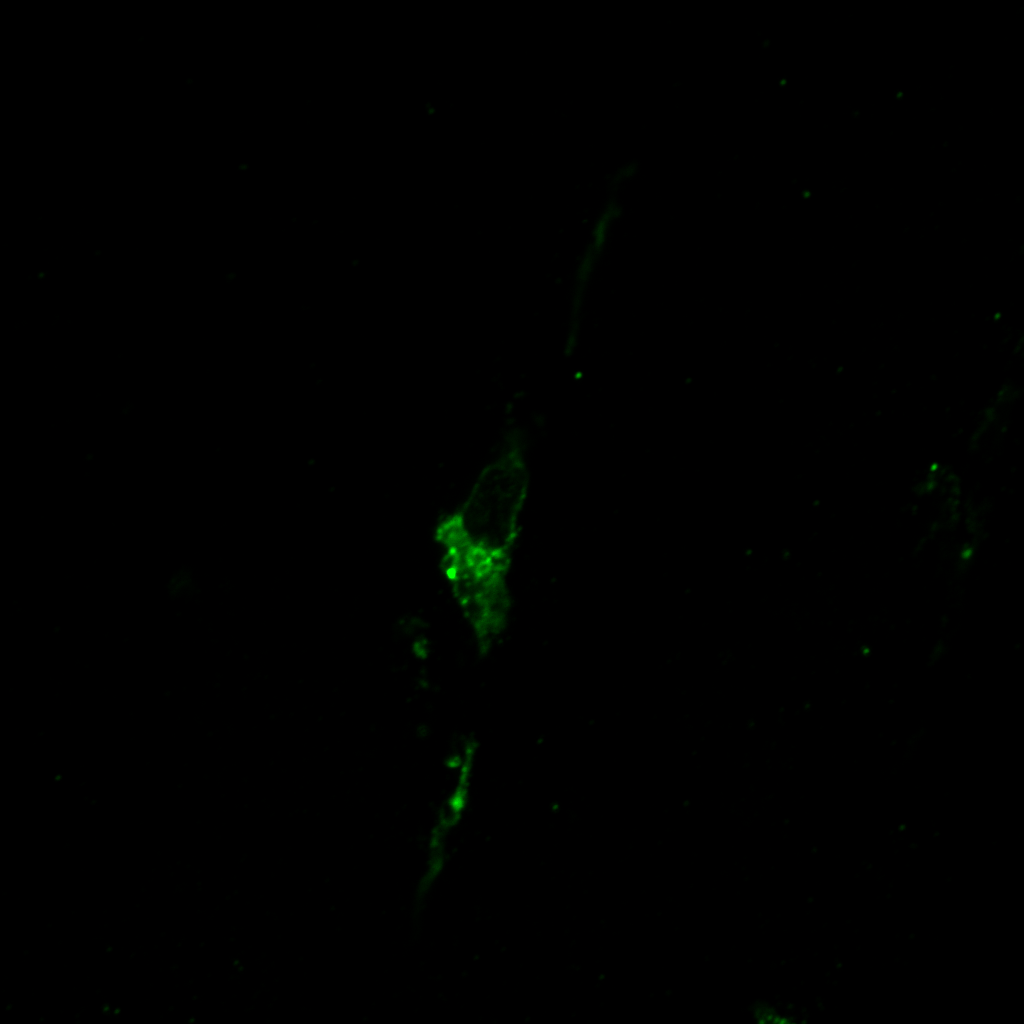

Supplement: Supplementary file 4 — Source data Fig. 3 [file 44318_2026_817_MOESM4_ESM.zip › 3D/3D-2-dKO_GAL3.tif]

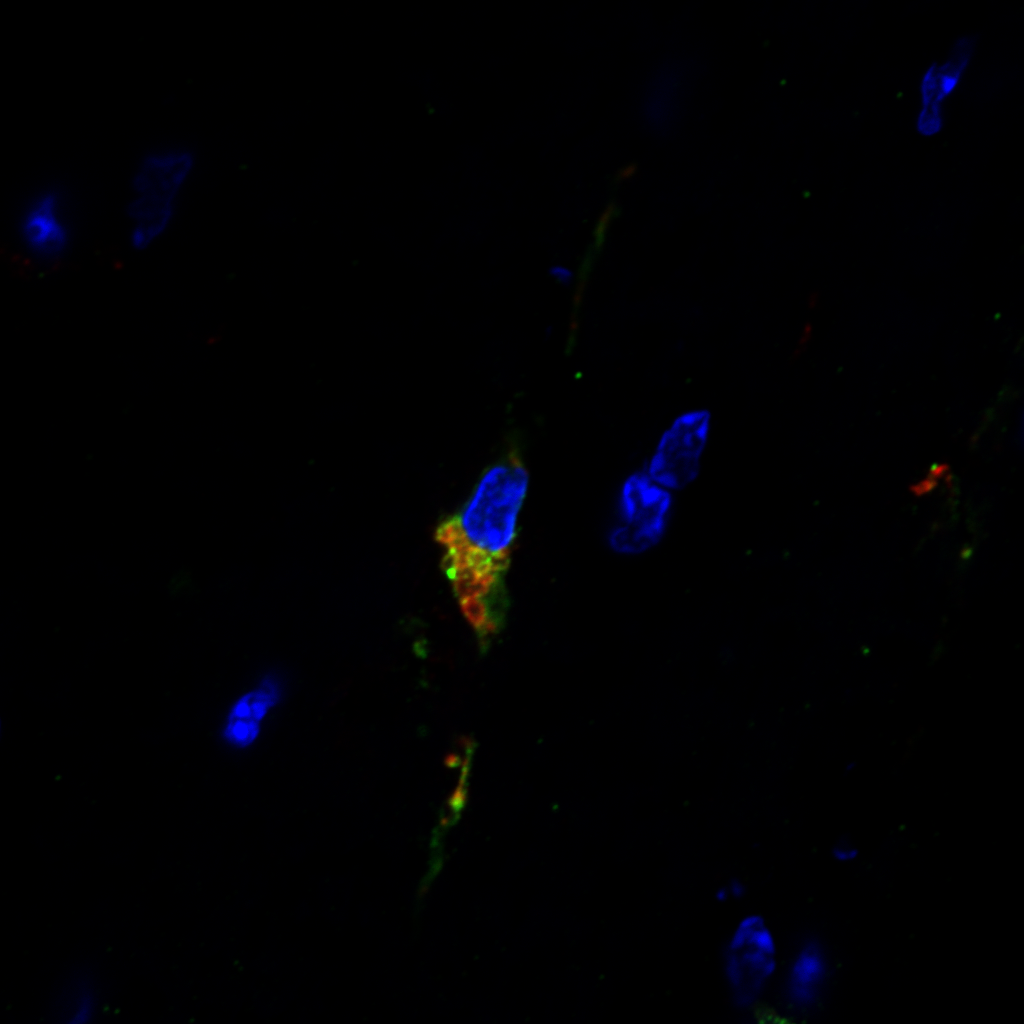

Supplement: Supplementary file 4 — Source data Fig. 3 [file 44318_2026_817_MOESM4_ESM.zip › 3D/3D-2-dKO_Merge.tif]

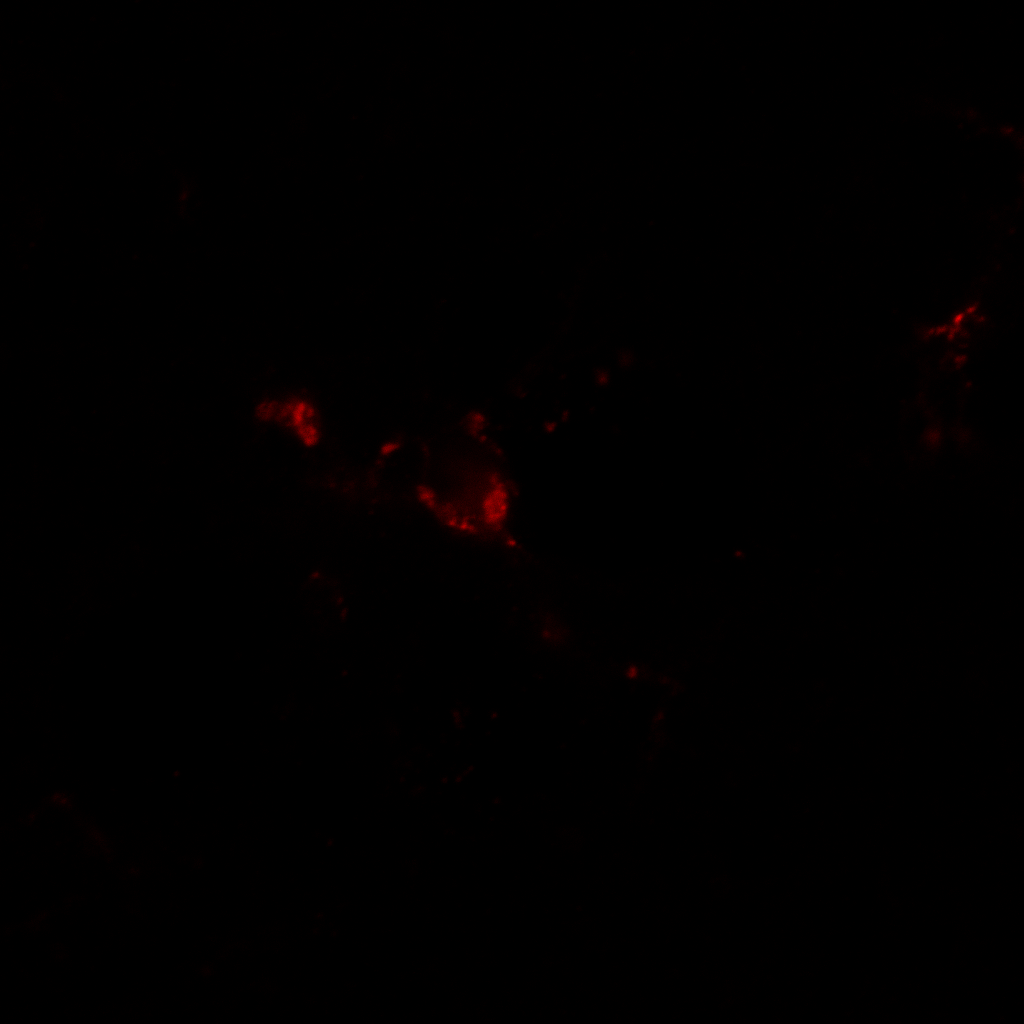

Supplement: Supplementary file 4 — Source data Fig. 3 [file 44318_2026_817_MOESM4_ESM.zip › 3F/3F-1-WT_CD68.tif]

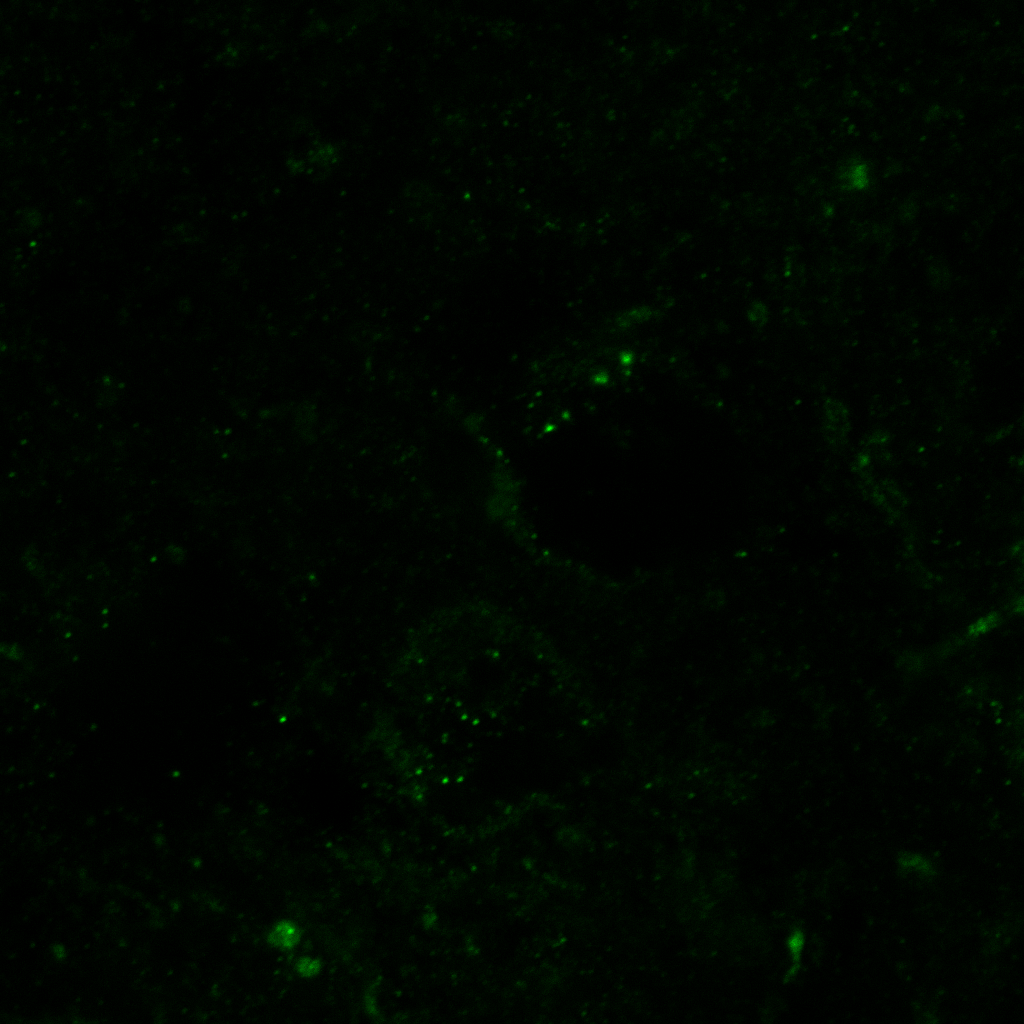

Supplement: Supplementary file 4 — Source data Fig. 3 [file 44318_2026_817_MOESM4_ESM.zip › 3F/3F-1-WT_GAL3.tif]

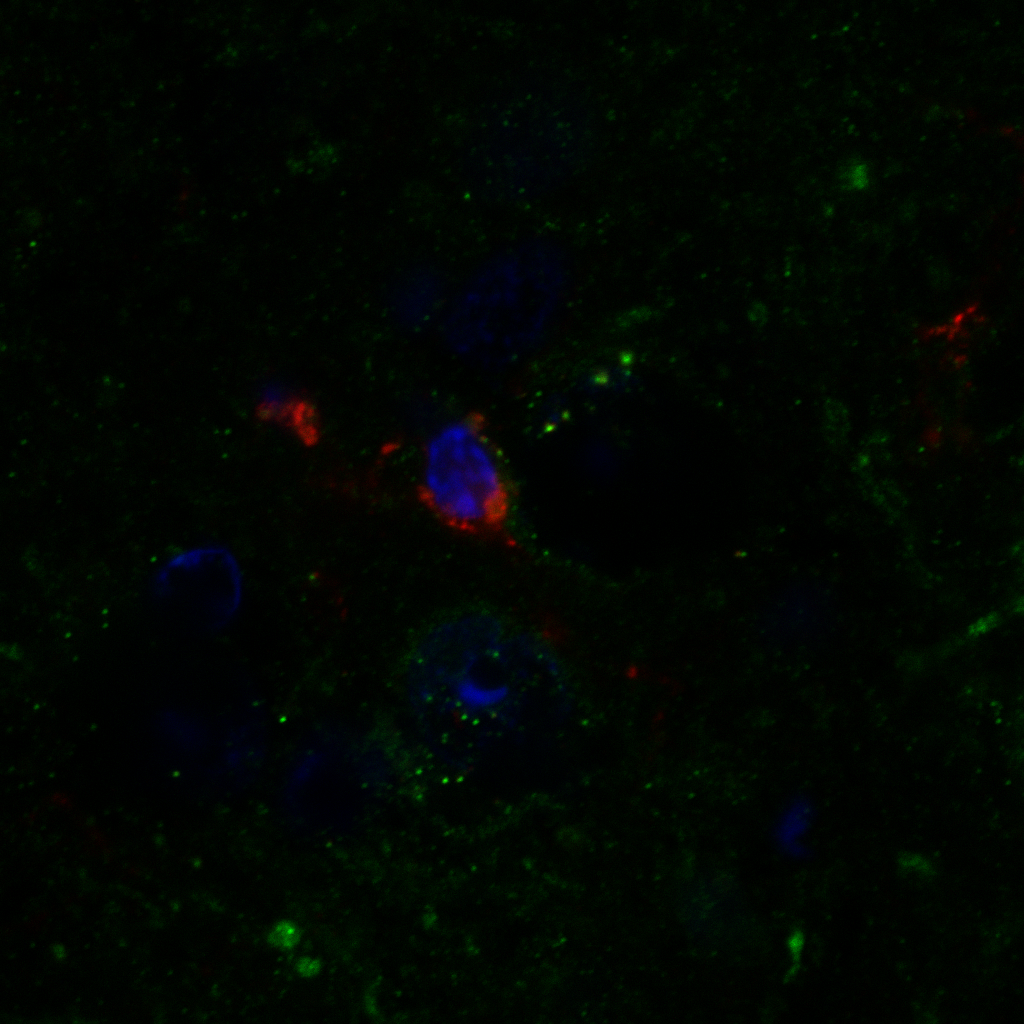

Supplement: Supplementary file 4 — Source data Fig. 3 [file 44318_2026_817_MOESM4_ESM.zip › 3F/3F-1-WT_Merge.tif]

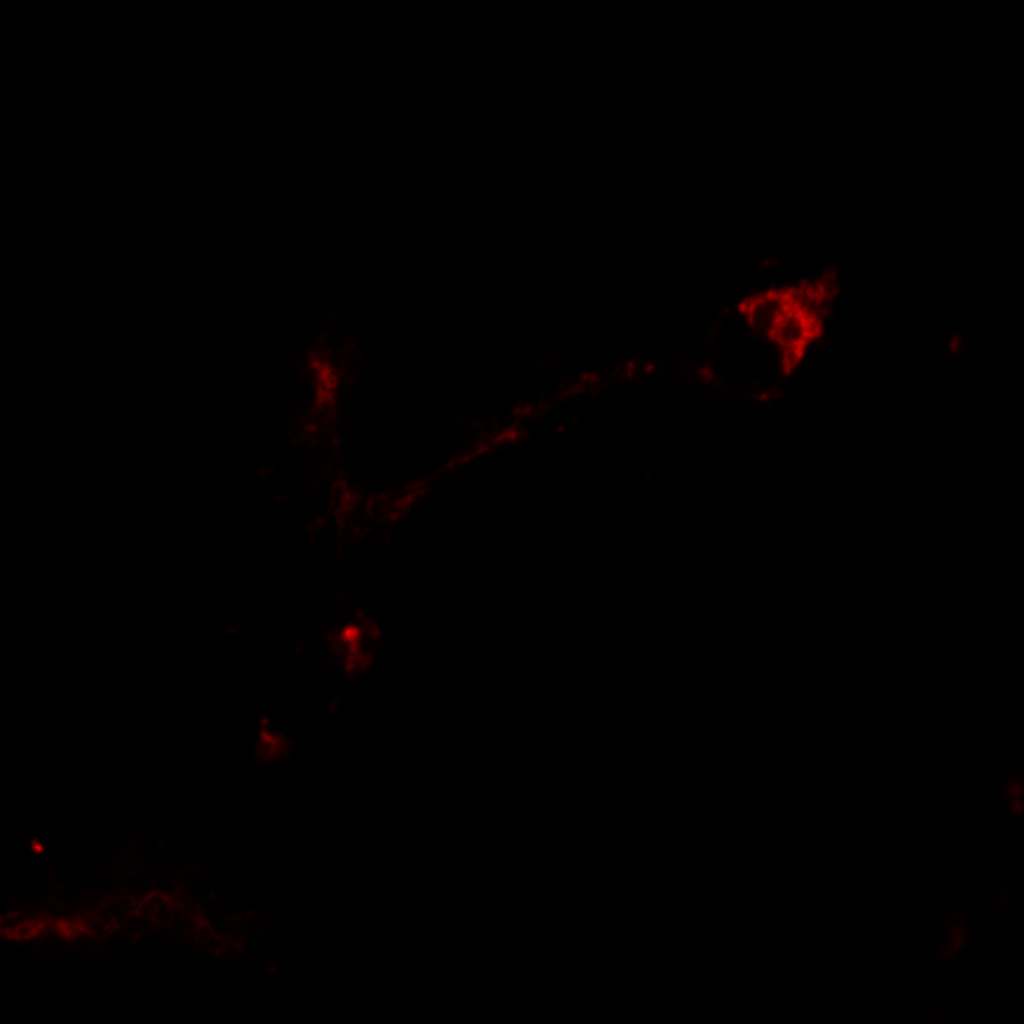

Supplement: Supplementary file 4 — Source data Fig. 3 [file 44318_2026_817_MOESM4_ESM.zip › 3F/3F-2-dKO_CD68.tif]

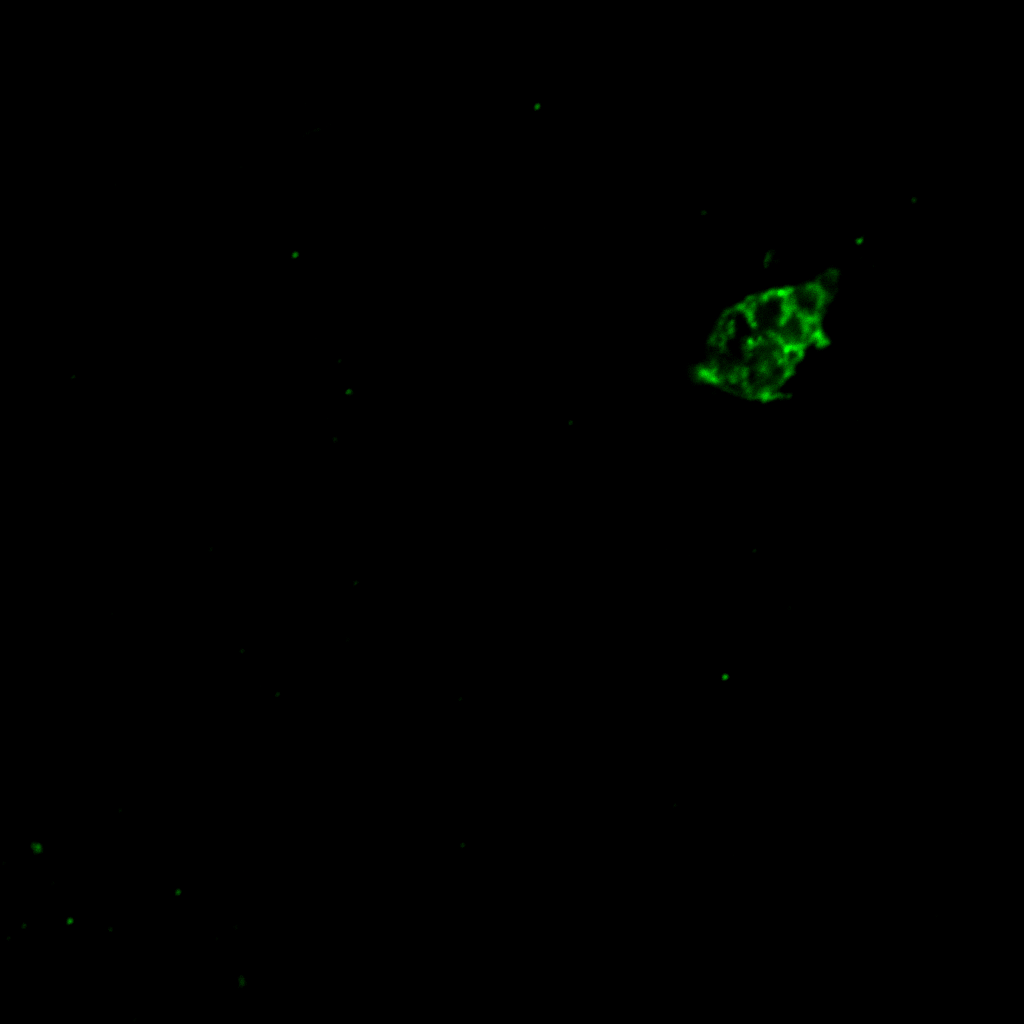

Supplement: Supplementary file 4 — Source data Fig. 3 [file 44318_2026_817_MOESM4_ESM.zip › 3F/3F-2-dKO_GAL3.tif]

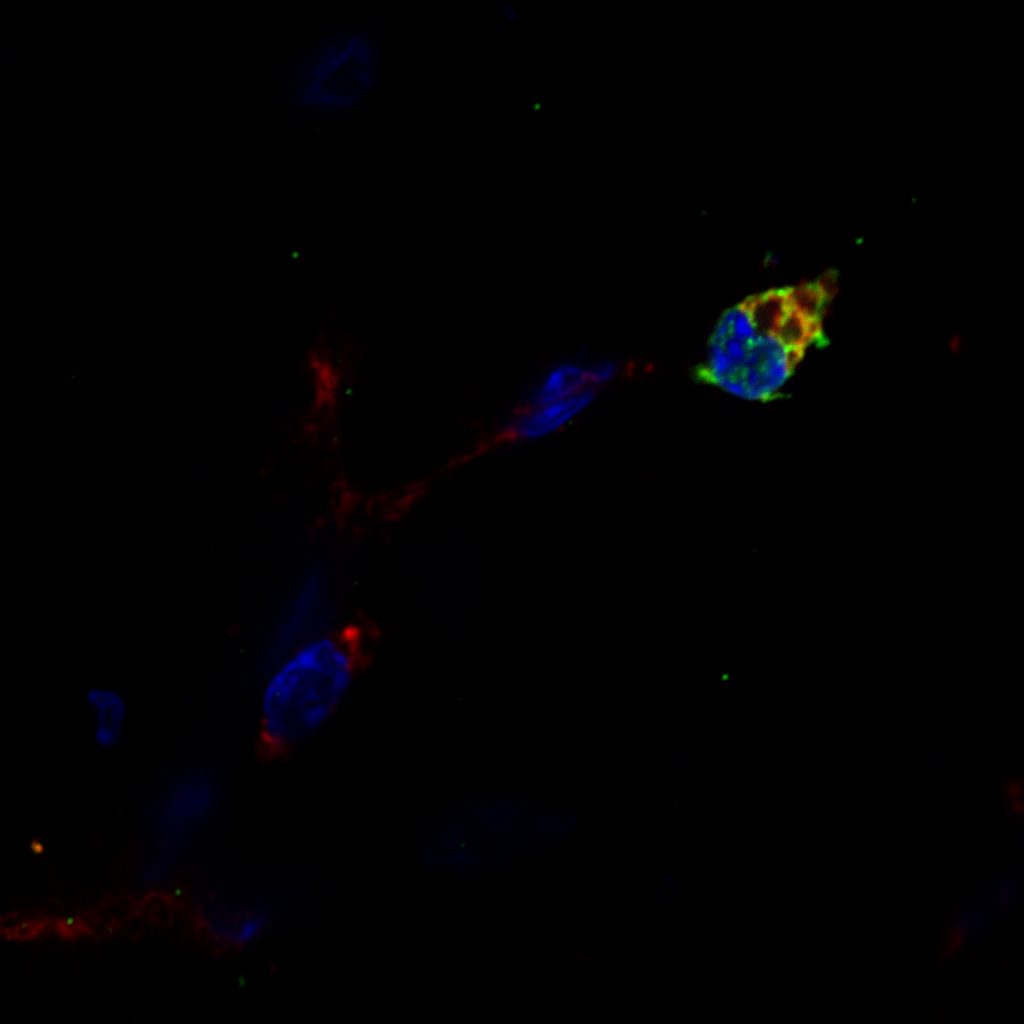

Supplement: Supplementary file 4 — Source data Fig. 3 [file 44318_2026_817_MOESM4_ESM.zip › 3F/3F-2-dKO_Merge.tif]

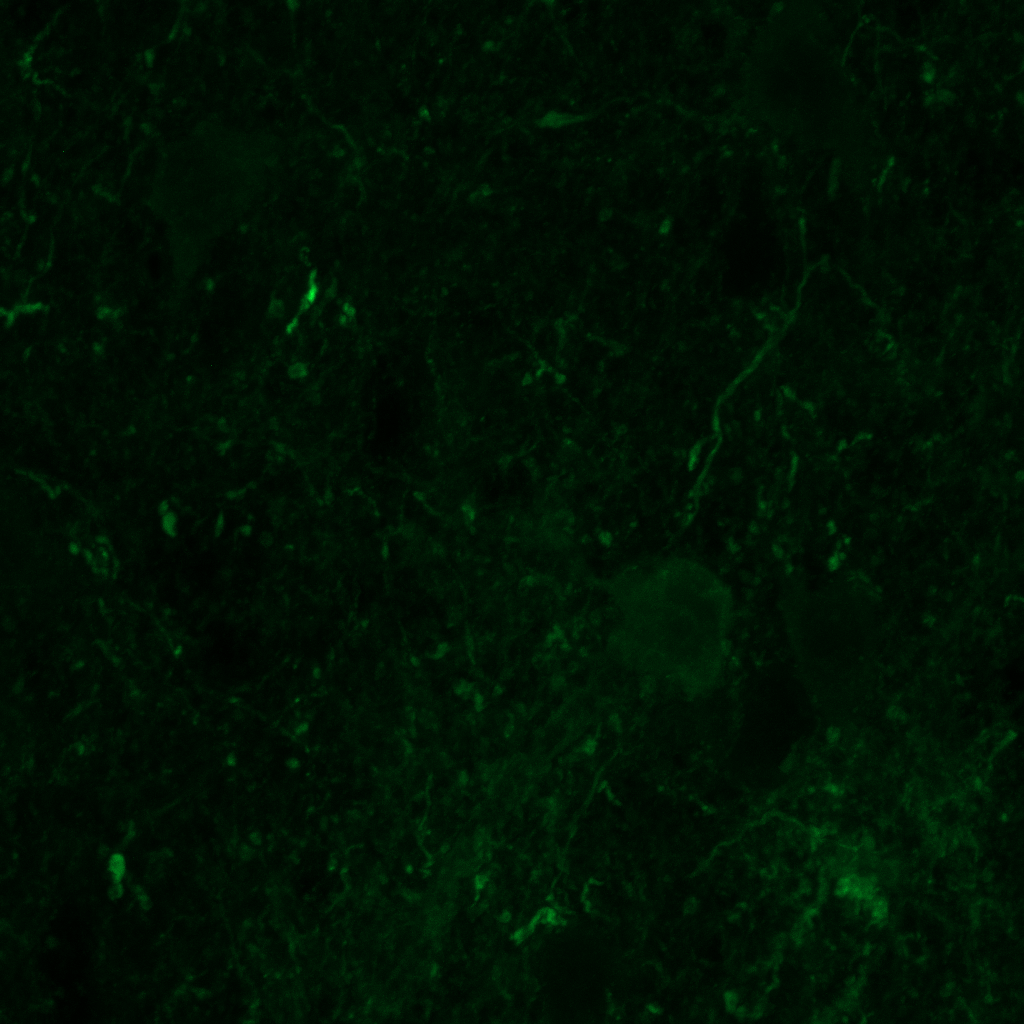

Supplement: Supplementary file 4 — Source data Fig. 3 [file 44318_2026_817_MOESM4_ESM.zip › 3H/3H-1-WT_expressing-GAL3-GFP-_GFP.tif]

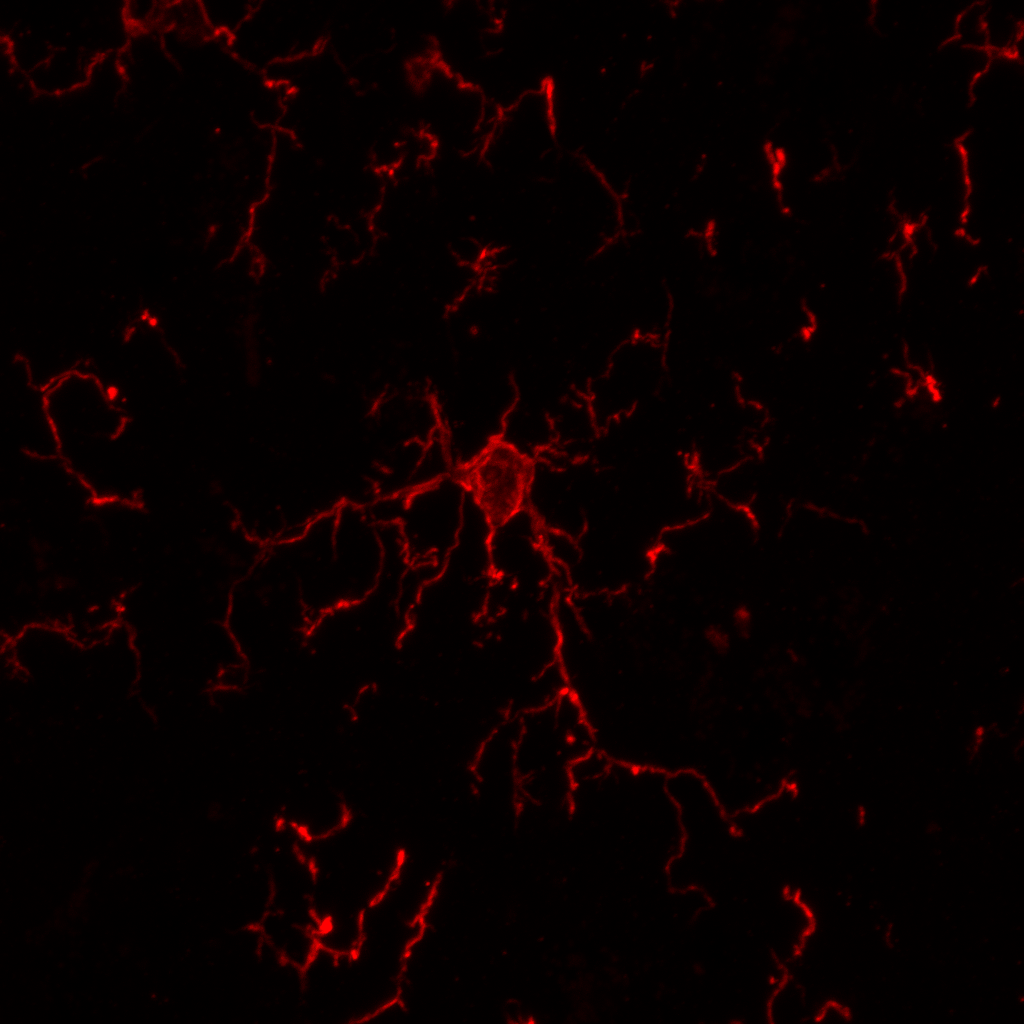

Supplement: Supplementary file 4 — Source data Fig. 3 [file 44318_2026_817_MOESM4_ESM.zip › 3H/3H-1-WT_expressing-GAL3-GFP-_IBA1.tif]

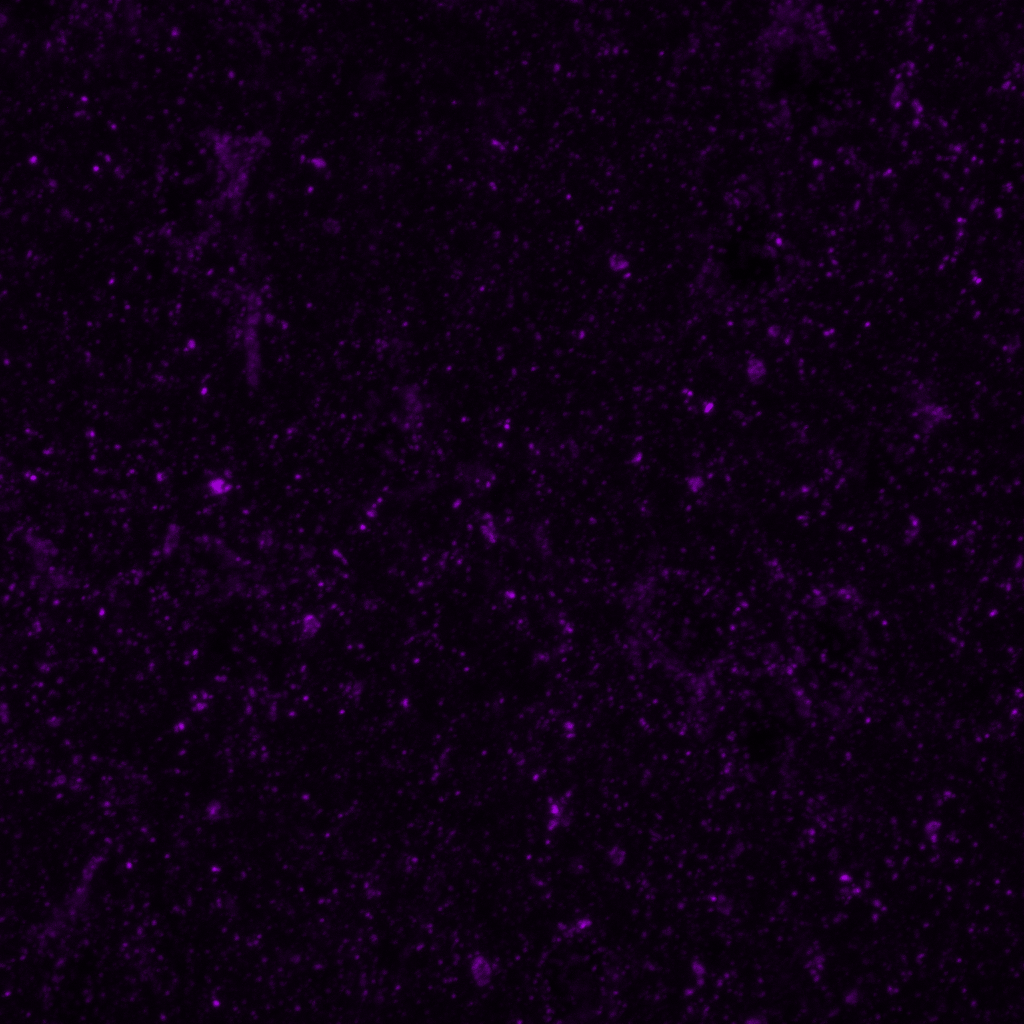

Supplement: Supplementary file 4 — Source data Fig. 3 [file 44318_2026_817_MOESM4_ESM.zip › 3H/3H-1-WT_expressing-GAL3-GFP-_LAMP1.tif]

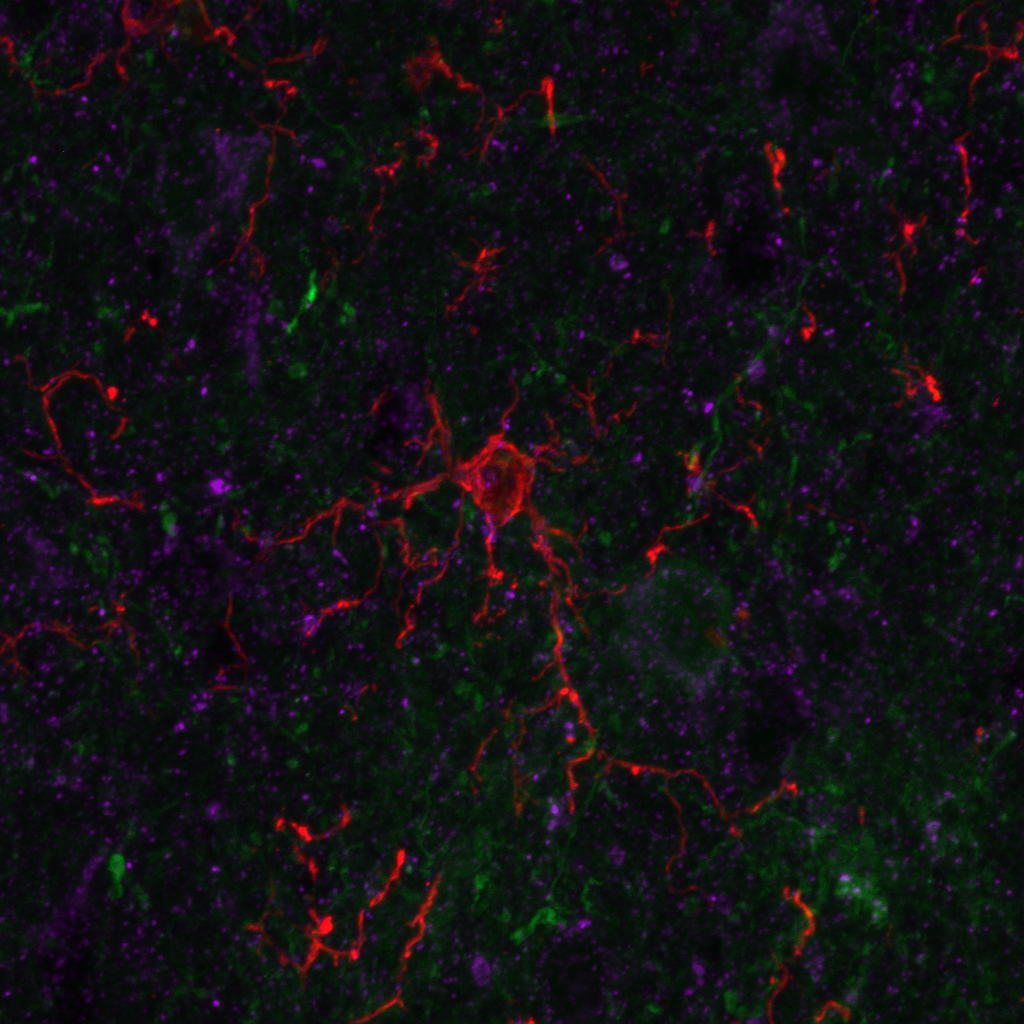

Supplement: Supplementary file 4 — Source data Fig. 3 [file 44318_2026_817_MOESM4_ESM.zip › 3H/3H-1-WT_expressing-GAL3-GFP-_Merge.tif]

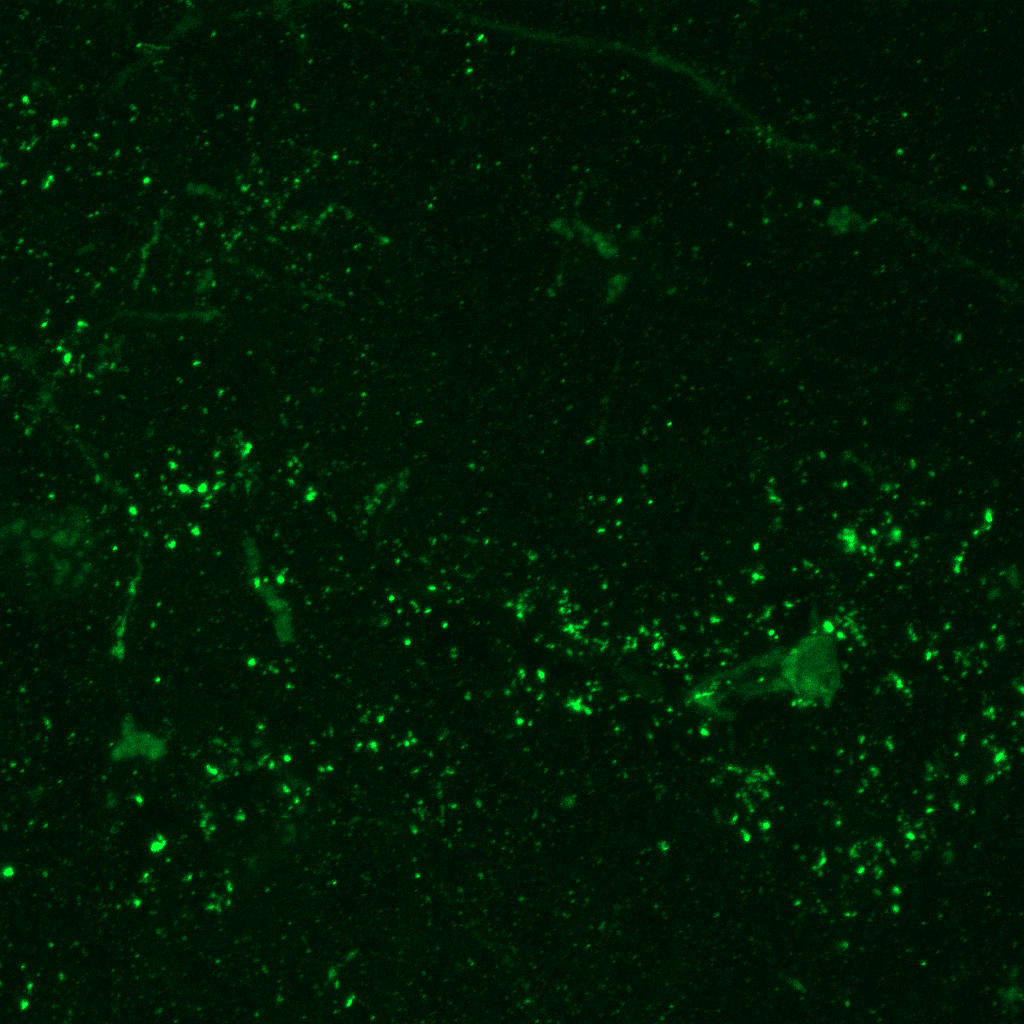

Supplement: Supplementary file 4 — Source data Fig. 3 [file 44318_2026_817_MOESM4_ESM.zip › 3H/3H-2-dKO_expressing-GAL3-GFP-_GFP.tif]

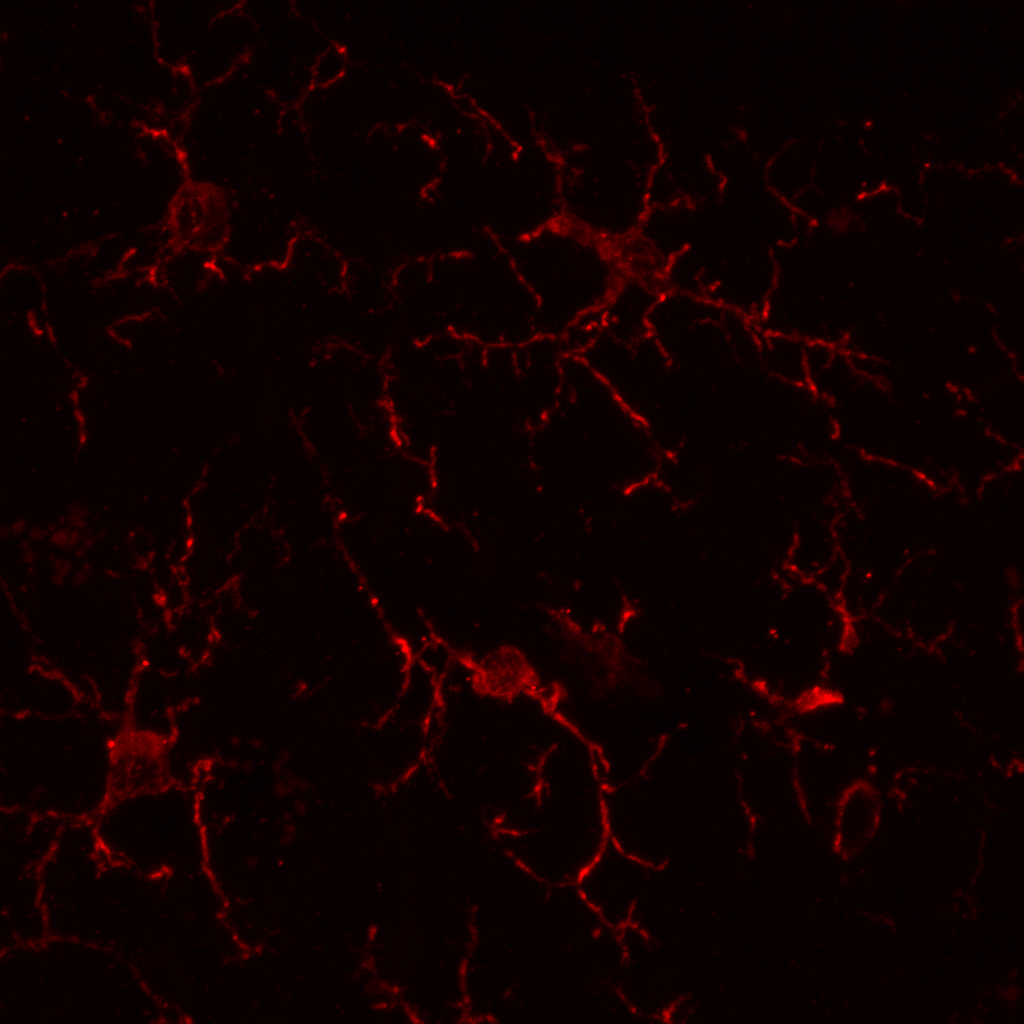

Supplement: Supplementary file 4 — Source data Fig. 3 [file 44318_2026_817_MOESM4_ESM.zip › 3H/3H-2-dKO_expressing-GAL3-GFP-_IBA1.tif]

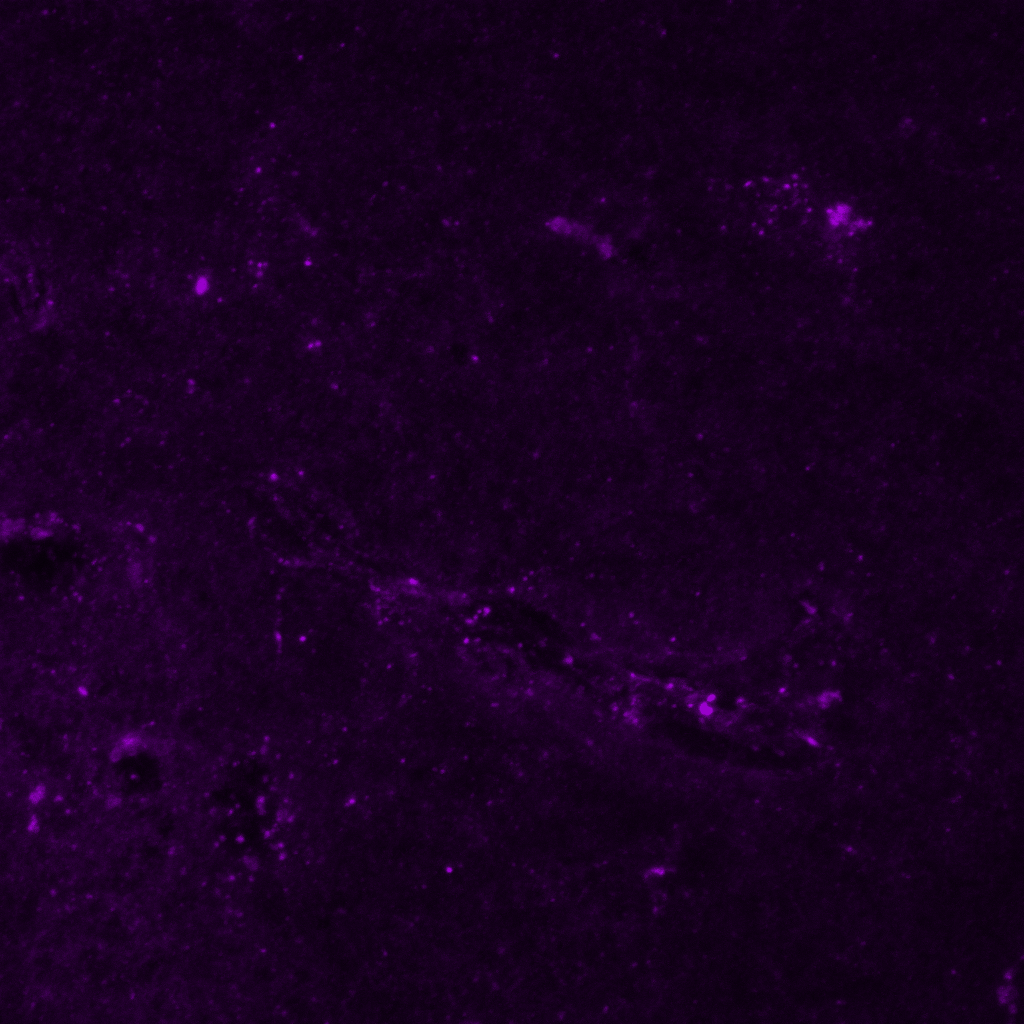

Supplement: Supplementary file 4 — Source data Fig. 3 [file 44318_2026_817_MOESM4_ESM.zip › 3H/3H-2-dKO_expressing-GAL3-GFP-_LAMP1.tif]

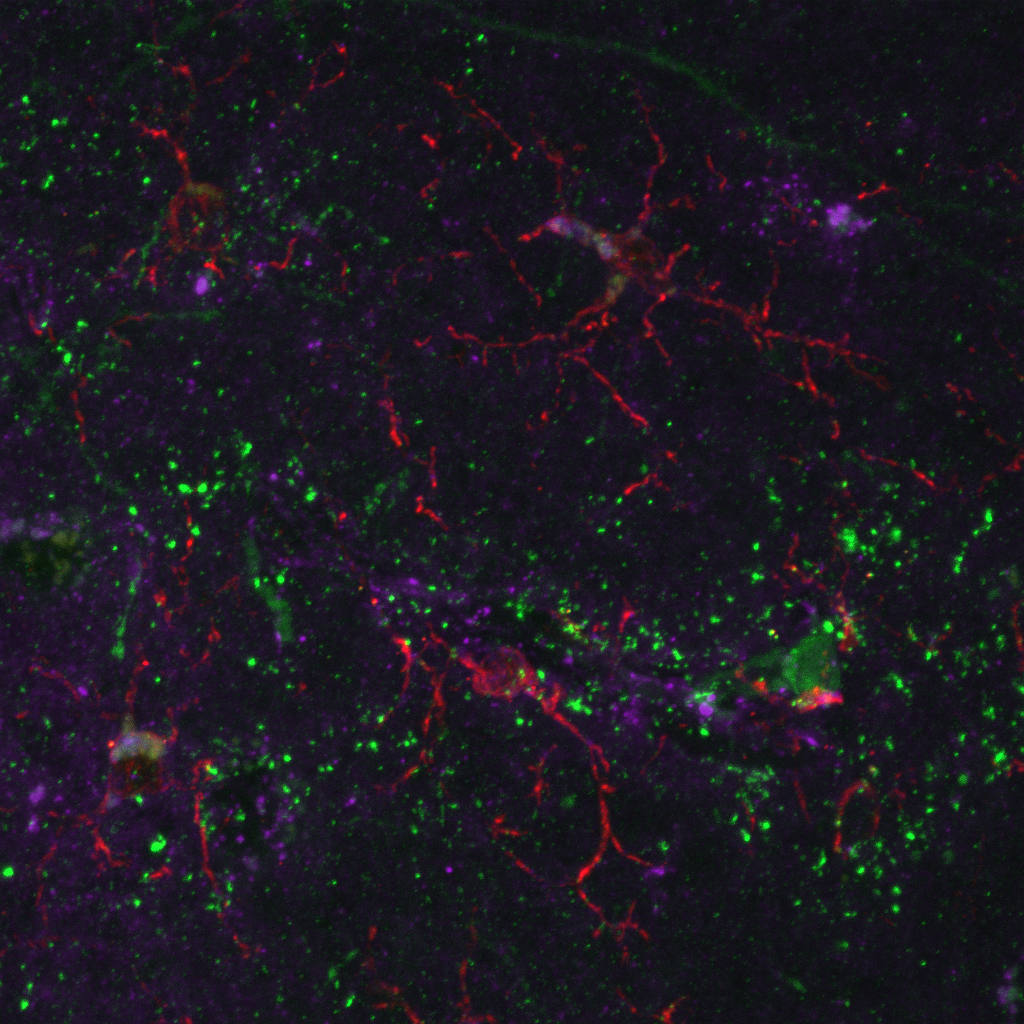

Supplement: Supplementary file 4 — Source data Fig. 3 [file 44318_2026_817_MOESM4_ESM.zip › 3H/3H-2-dKO_expressing-GAL3-GFP-_Merge.tif]

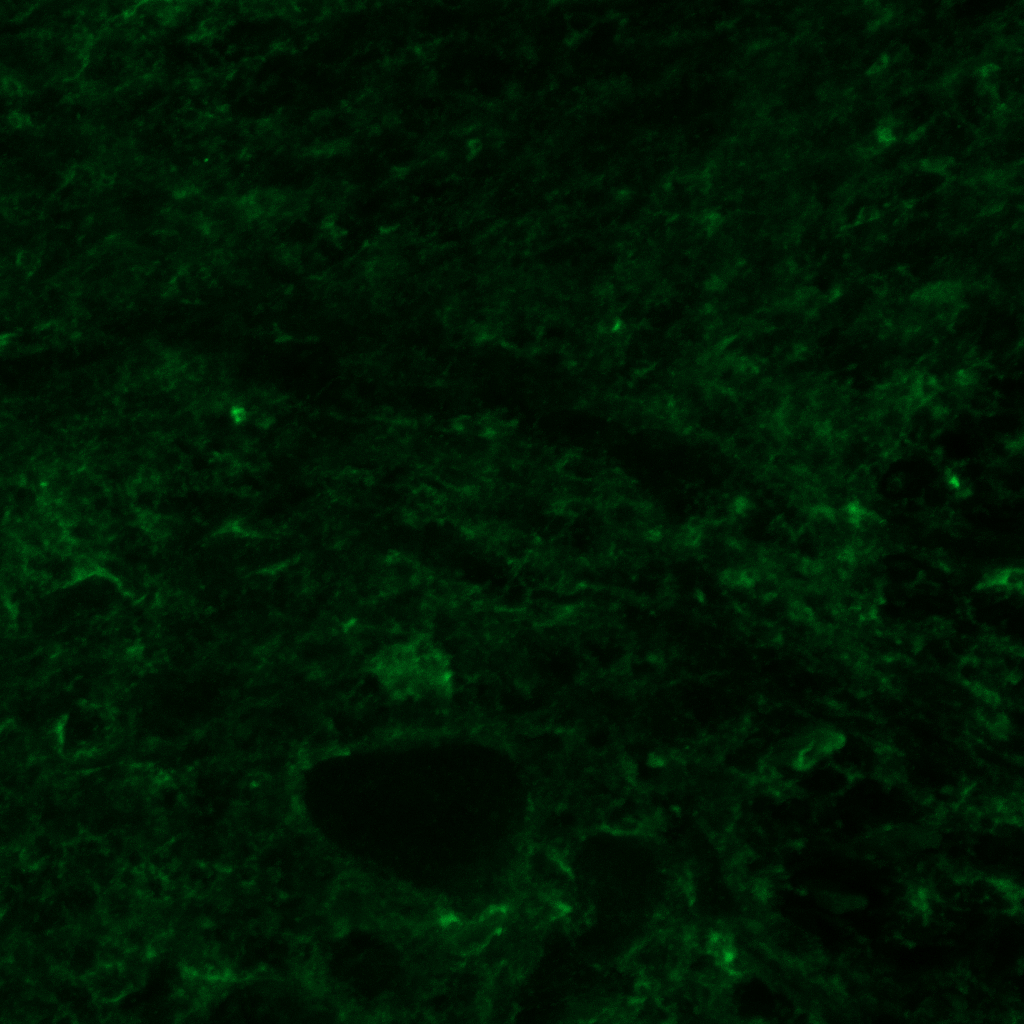

Supplement: Supplementary file 4 — Source data Fig. 3 [file 44318_2026_817_MOESM4_ESM.zip › 3J/3J-1-WT_expressing-GAL3-GFP-_GFP.tif]

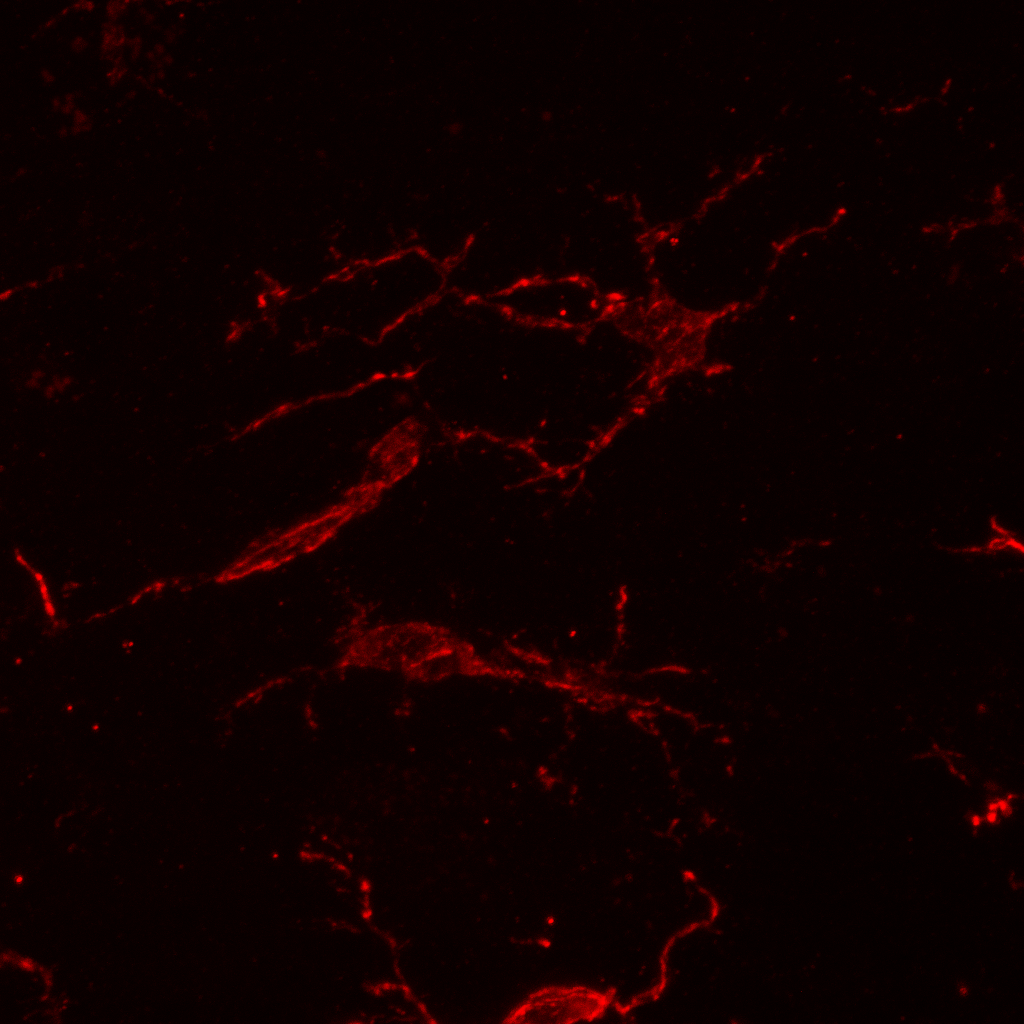

Supplement: Supplementary file 4 — Source data Fig. 3 [file 44318_2026_817_MOESM4_ESM.zip › 3J/3J-1-WT_expressing-GAL3-GFP-_IBA1.tif]

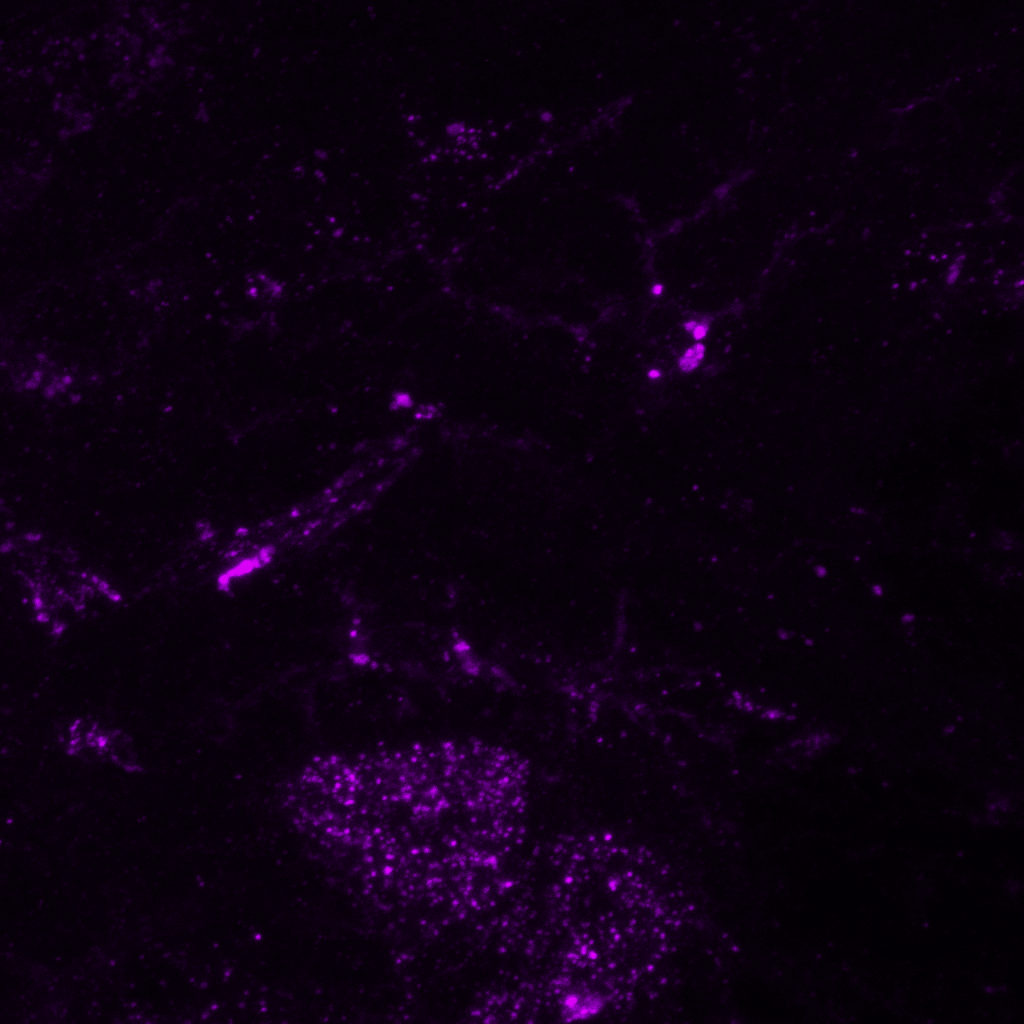

Supplement: Supplementary file 4 — Source data Fig. 3 [file 44318_2026_817_MOESM4_ESM.zip › 3J/3J-1-WT_expressing-GAL3-GFP-_LAMP1.tif]

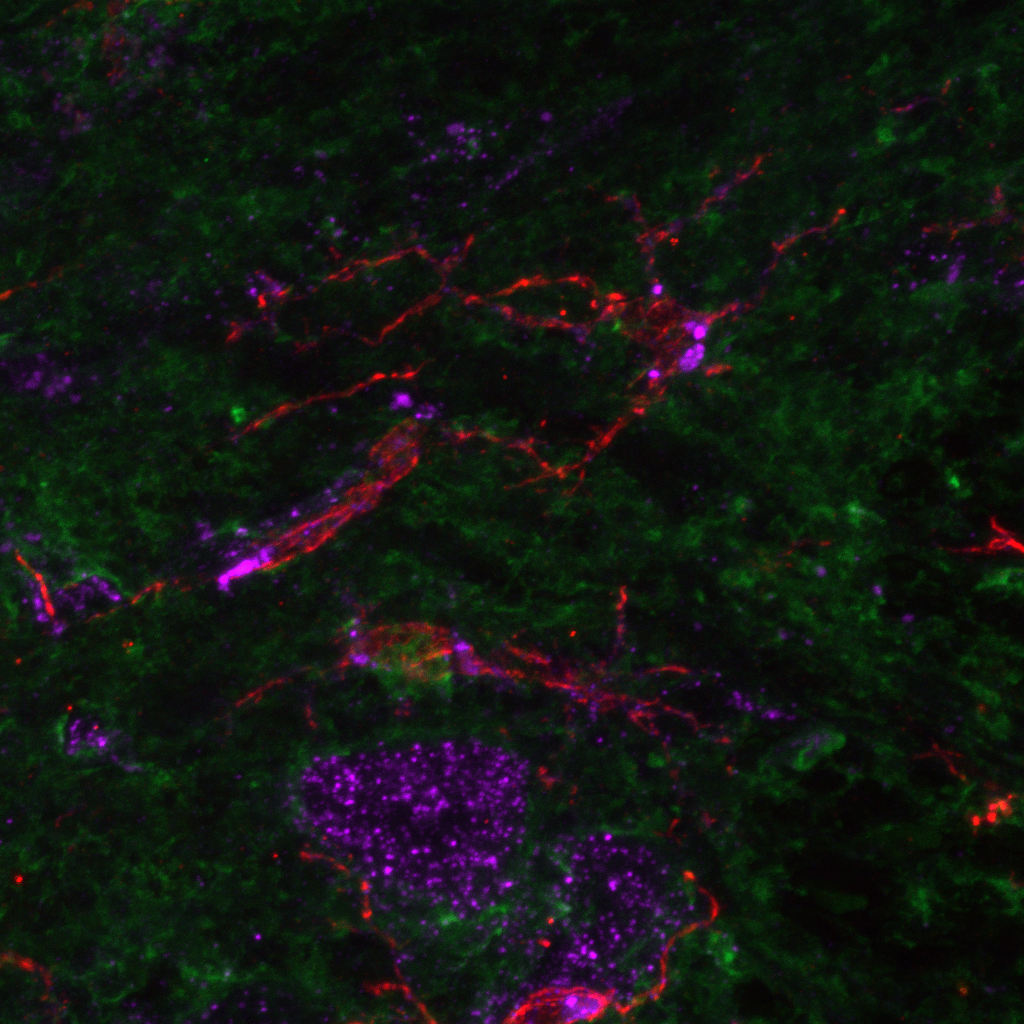

Supplement: Supplementary file 4 — Source data Fig. 3 [file 44318_2026_817_MOESM4_ESM.zip › 3J/3J-1-WT_expressing-GAL3-GFP-_Merge.tif]

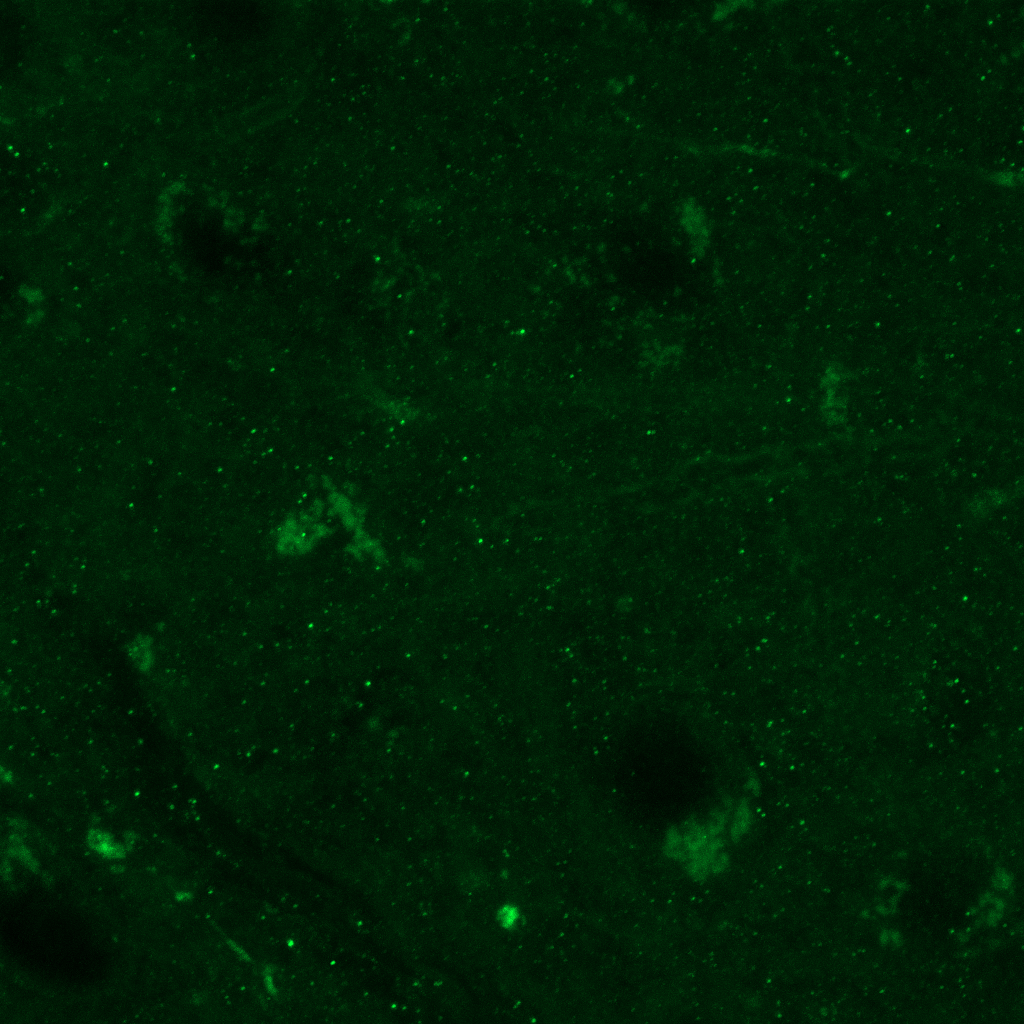

Supplement: Supplementary file 4 — Source data Fig. 3 [file 44318_2026_817_MOESM4_ESM.zip › 3J/3J-2-dKO_expressing-GAL3-GFP-_GFP.tif]

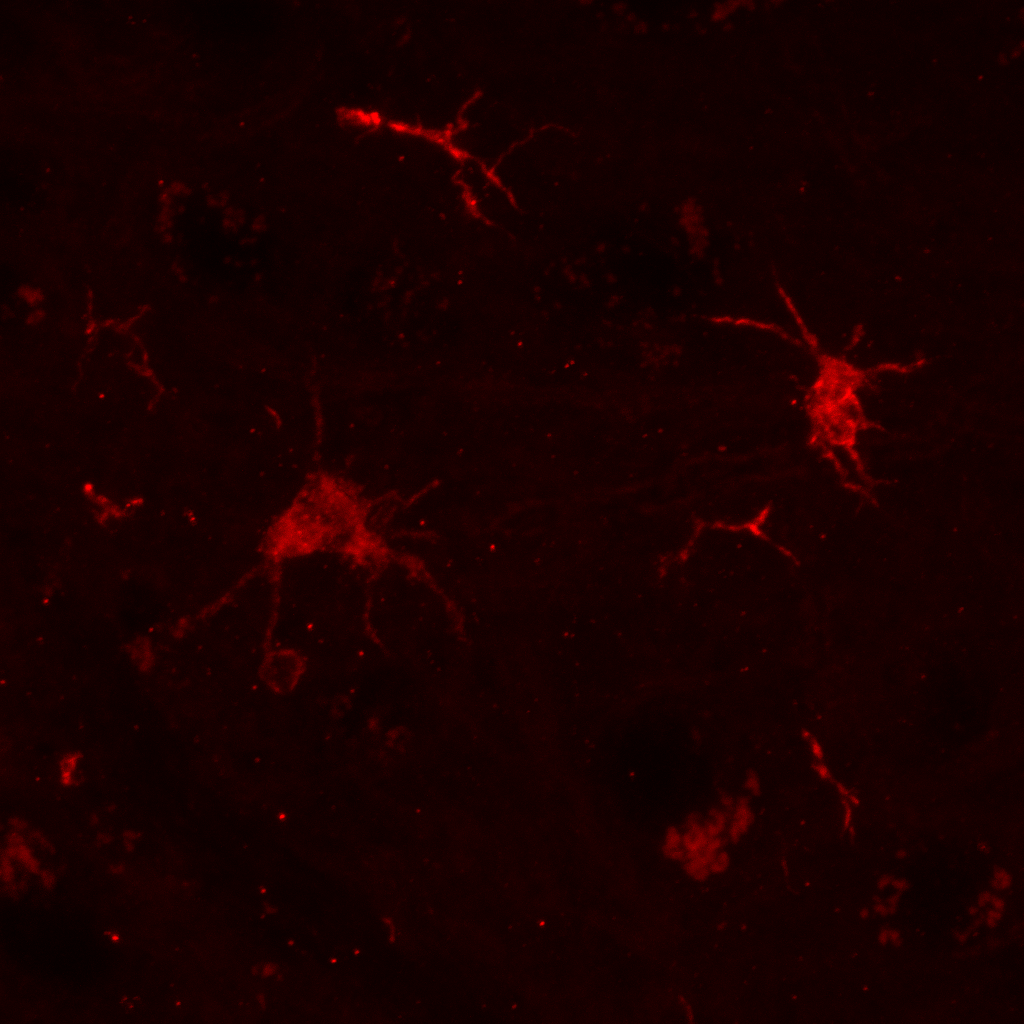

Supplement: Supplementary file 4 — Source data Fig. 3 [file 44318_2026_817_MOESM4_ESM.zip › 3J/3J-2-dKO_expressing-GAL3-GFP-_IBA1.tif]

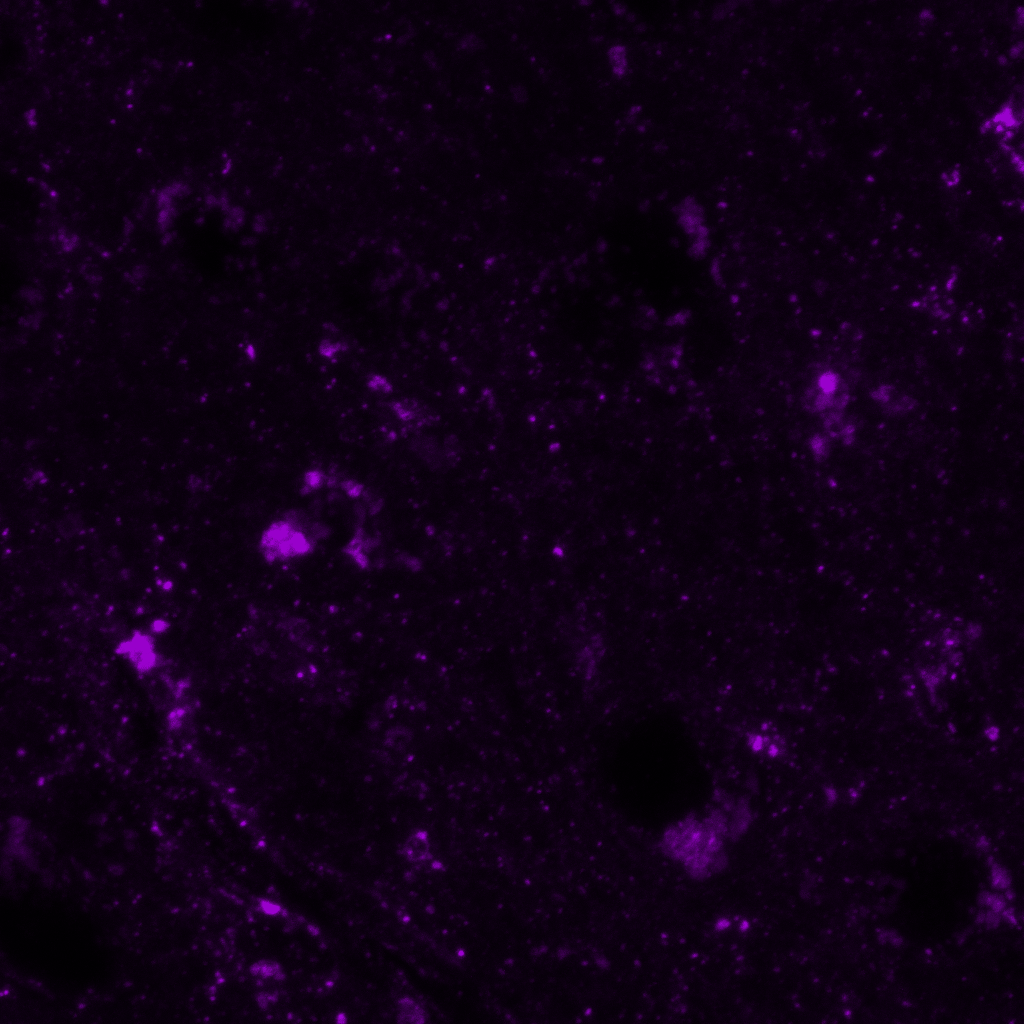

Supplement: Supplementary file 4 — Source data Fig. 3 [file 44318_2026_817_MOESM4_ESM.zip › 3J/3J-2-dKO_expressing-GAL3-GFP-_LAMP1.tif]

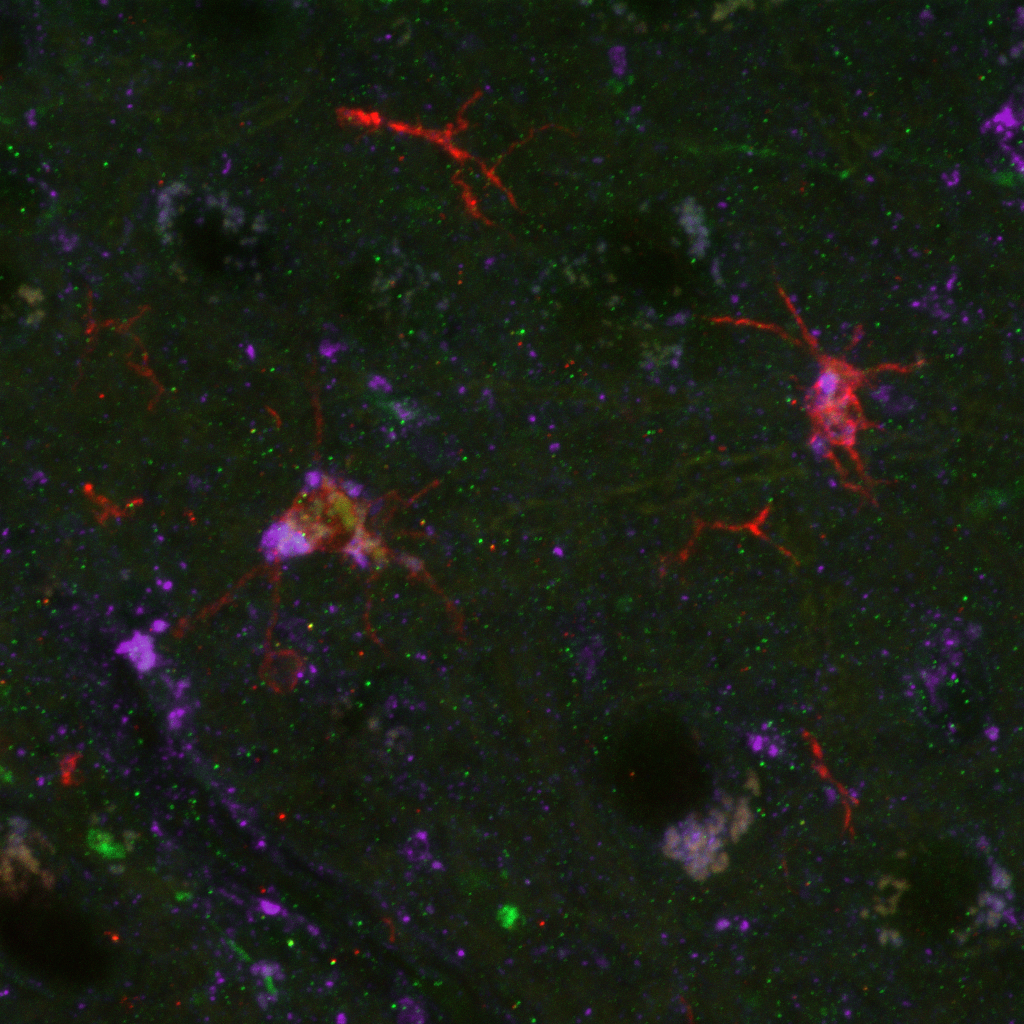

Supplement: Supplementary file 4 — Source data Fig. 3 [file 44318_2026_817_MOESM4_ESM.zip › 3J/3J-2-dKO_expressing-GAL3-GFP-_Merge.tif]

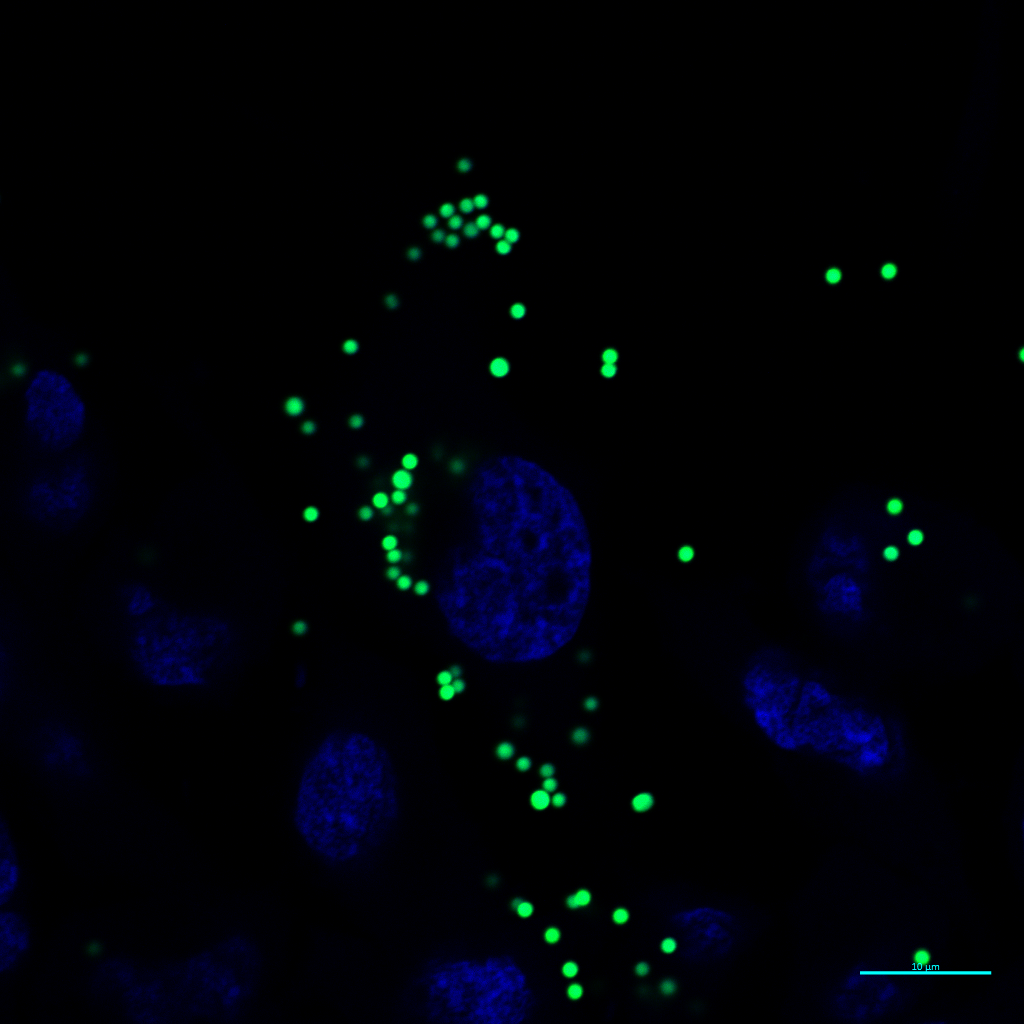

Supplement: Supplementary file 5 — Source data Fig. 4 [file 44318_2026_817_MOESM5_ESM.zip › 4C/4C-1-Basal-C9orf72 KO_Latex beads.tif]

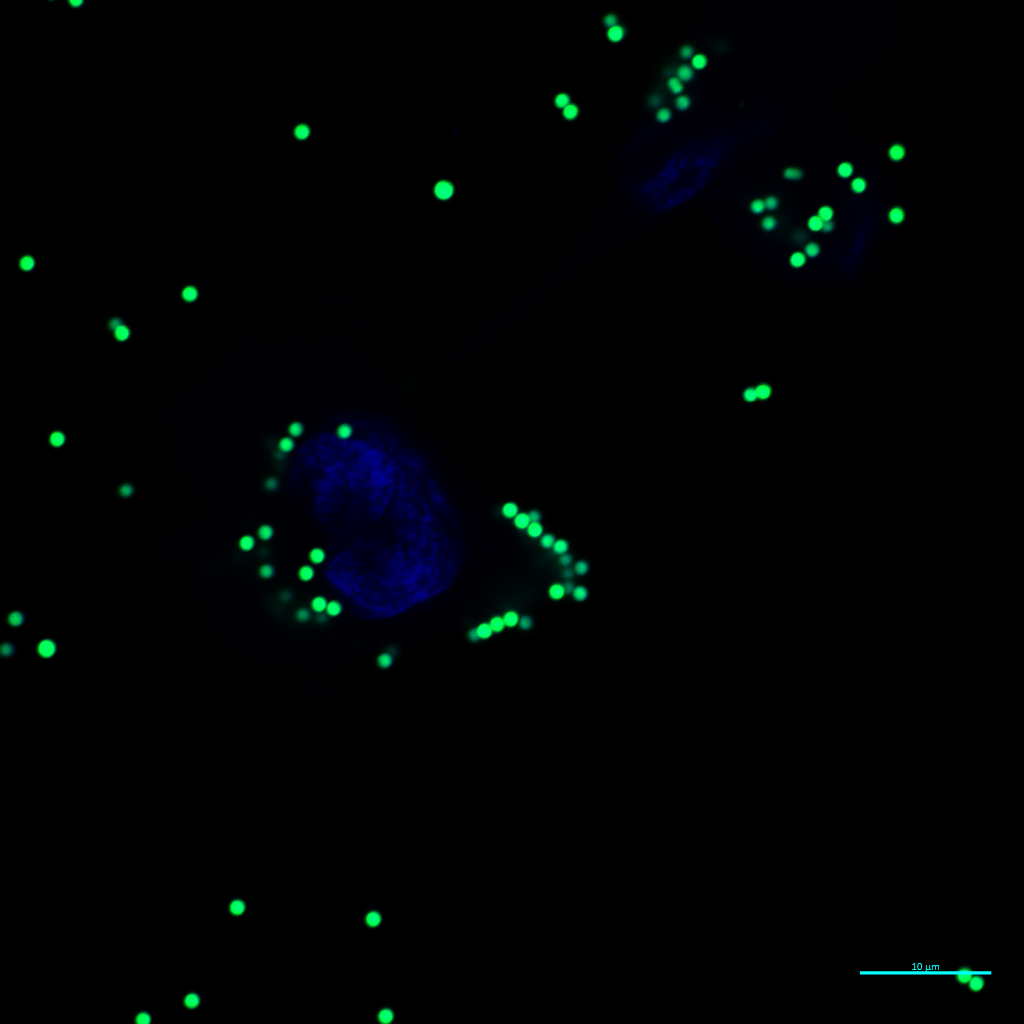

Supplement: Supplementary file 5 — Source data Fig. 4 [file 44318_2026_817_MOESM5_ESM.zip › 4C/4C-1-Basal-dKO_Latex beads.tif]

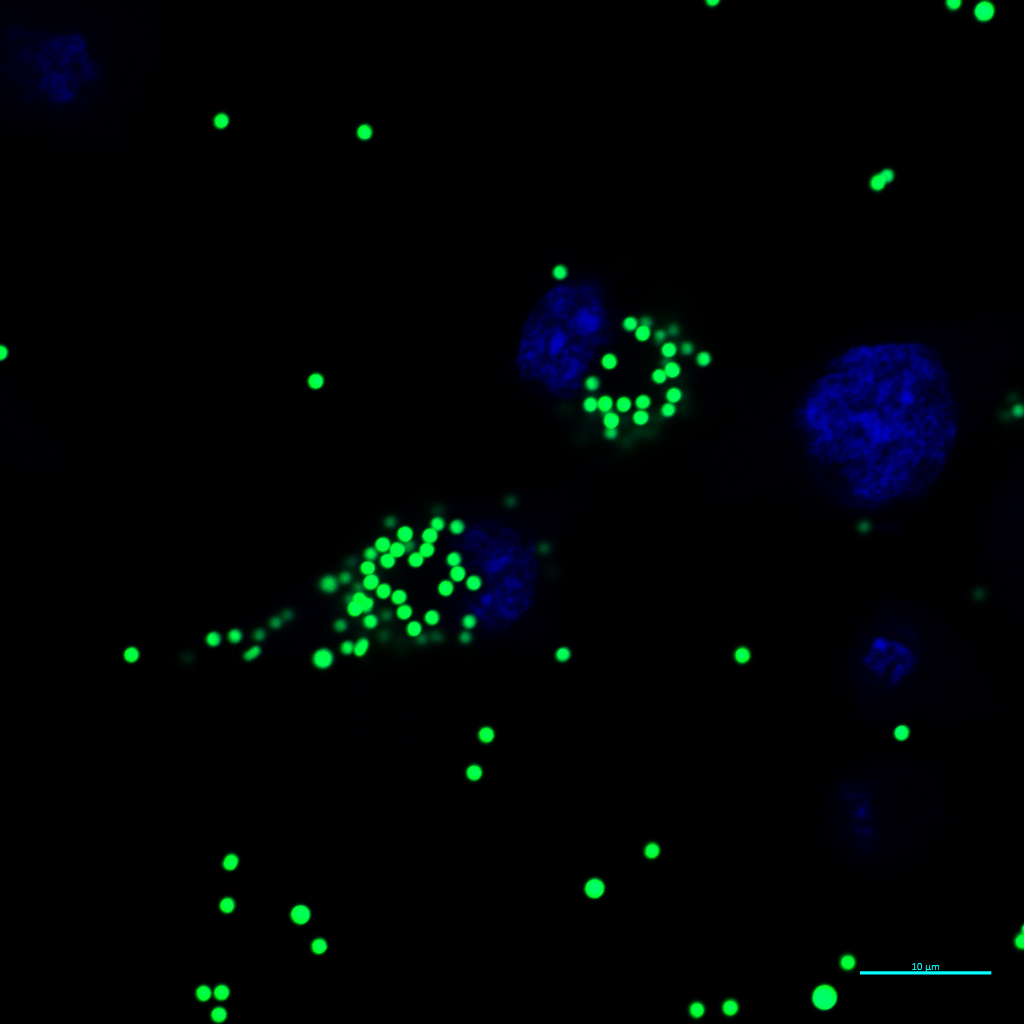

Supplement: Supplementary file 5 — Source data Fig. 4 [file 44318_2026_817_MOESM5_ESM.zip › 4C/4C-1-Basal-Smcr8 KO_Latex beads.tif]

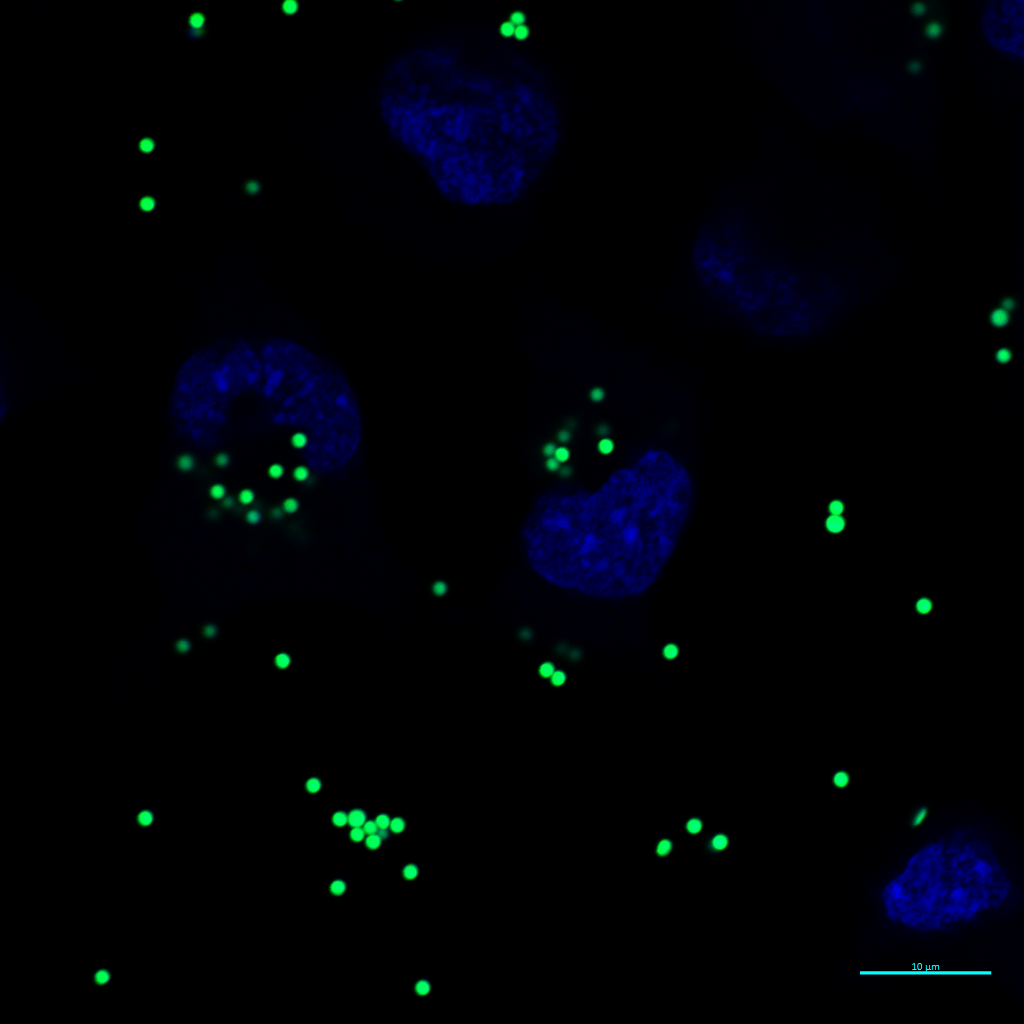

Supplement: Supplementary file 5 — Source data Fig. 4 [file 44318_2026_817_MOESM5_ESM.zip › 4C/4C-1-Basal-WT_Latex beads.tif]

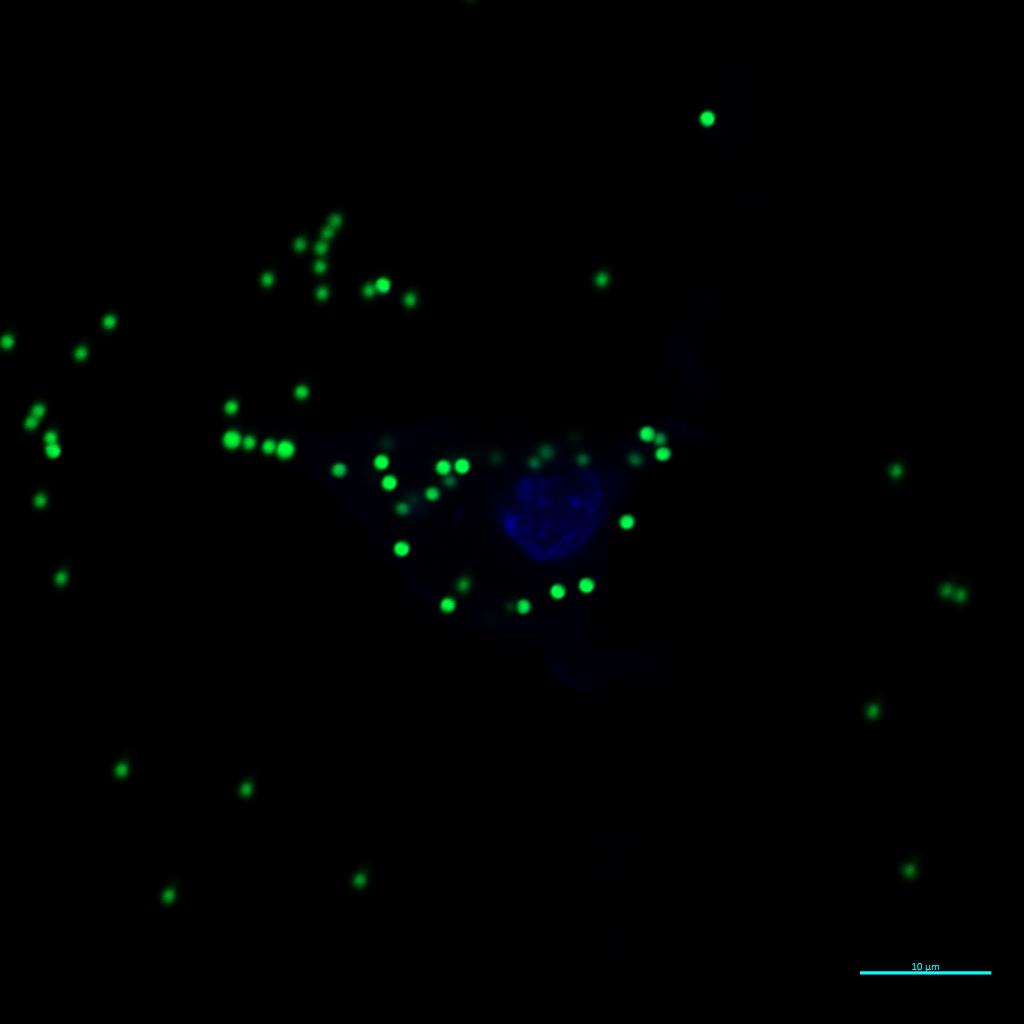

Supplement: Supplementary file 5 — Source data Fig. 4 [file 44318_2026_817_MOESM5_ESM.zip › 4C/4C-2-LLOMe-C9orf72 KO_Latex beads.tif]

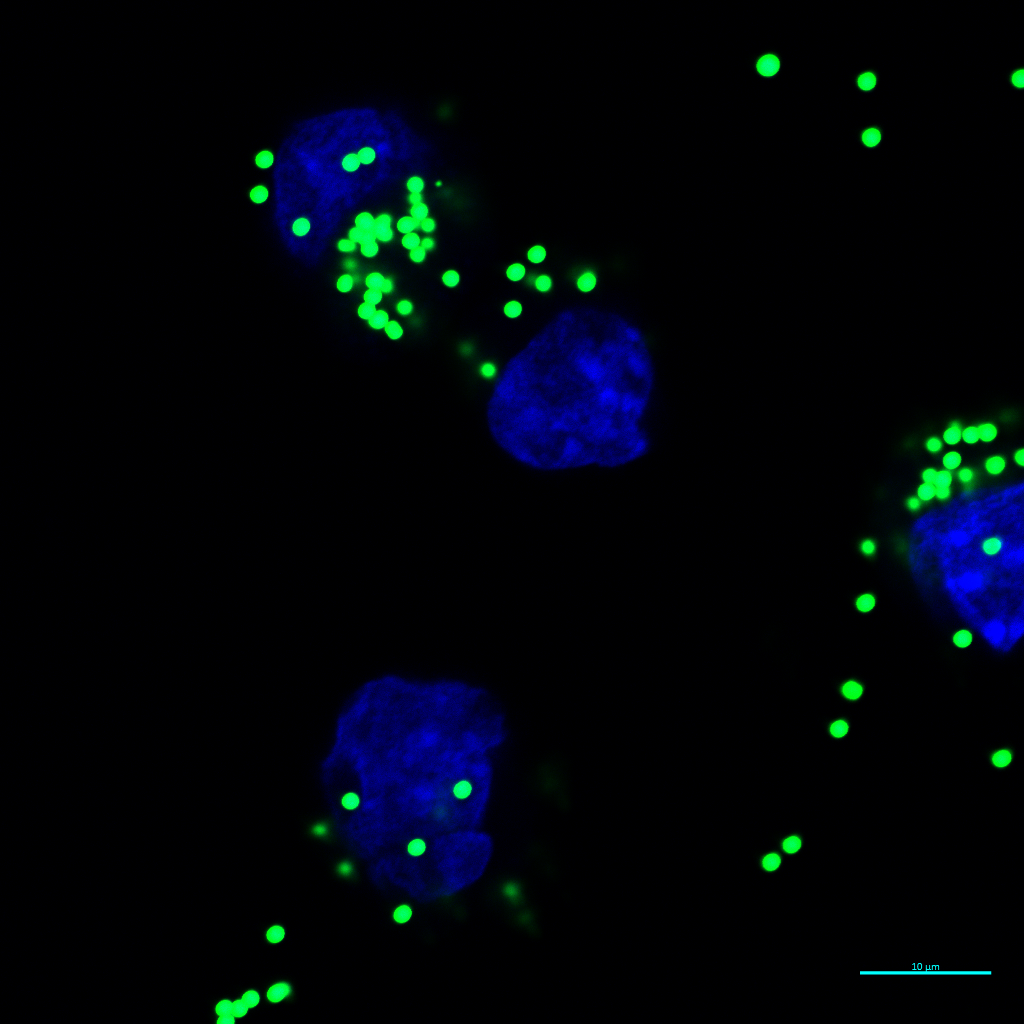

Supplement: Supplementary file 5 — Source data Fig. 4 [file 44318_2026_817_MOESM5_ESM.zip › 4C/4C-2-LLOMe-dKO_Latex beads.tif]

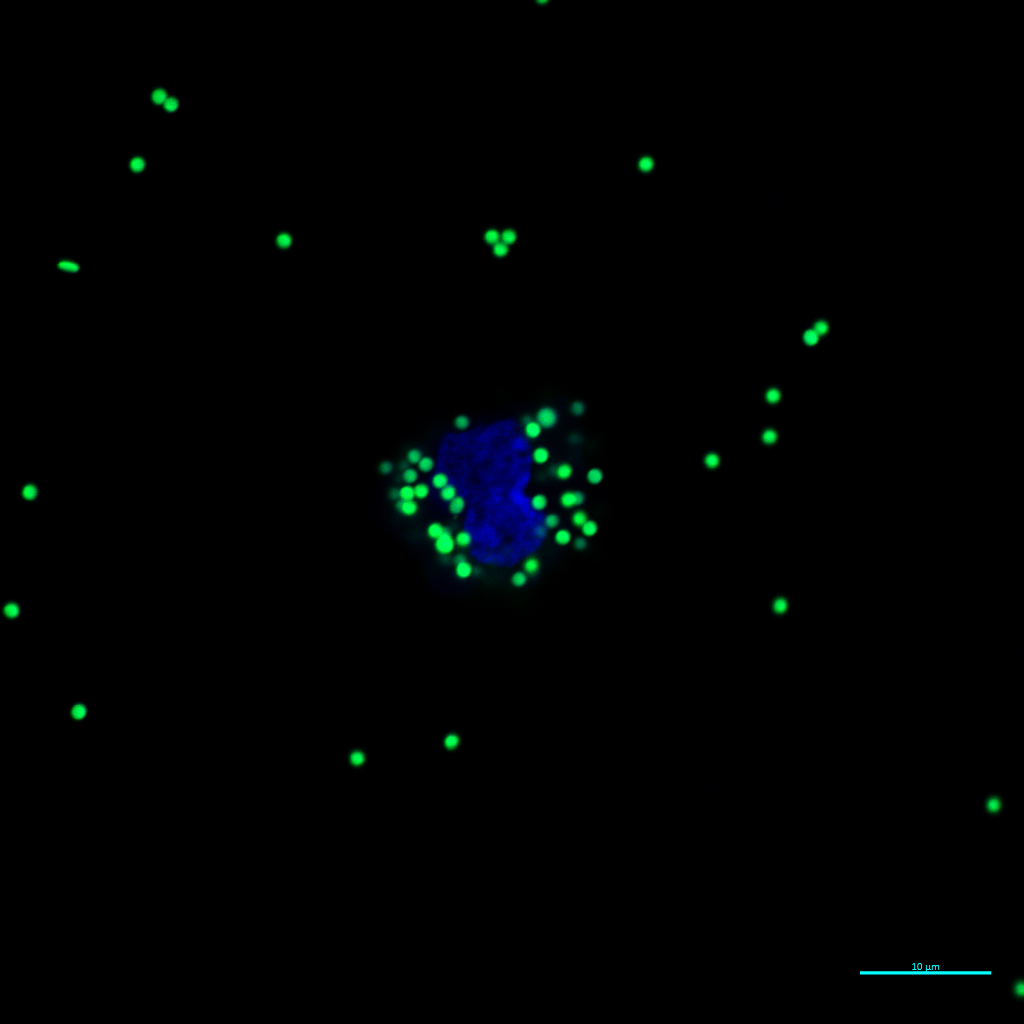

Supplement: Supplementary file 5 — Source data Fig. 4 [file 44318_2026_817_MOESM5_ESM.zip › 4C/4C-2-LLOMe-Smcr8 KO_Latex beads.tif]

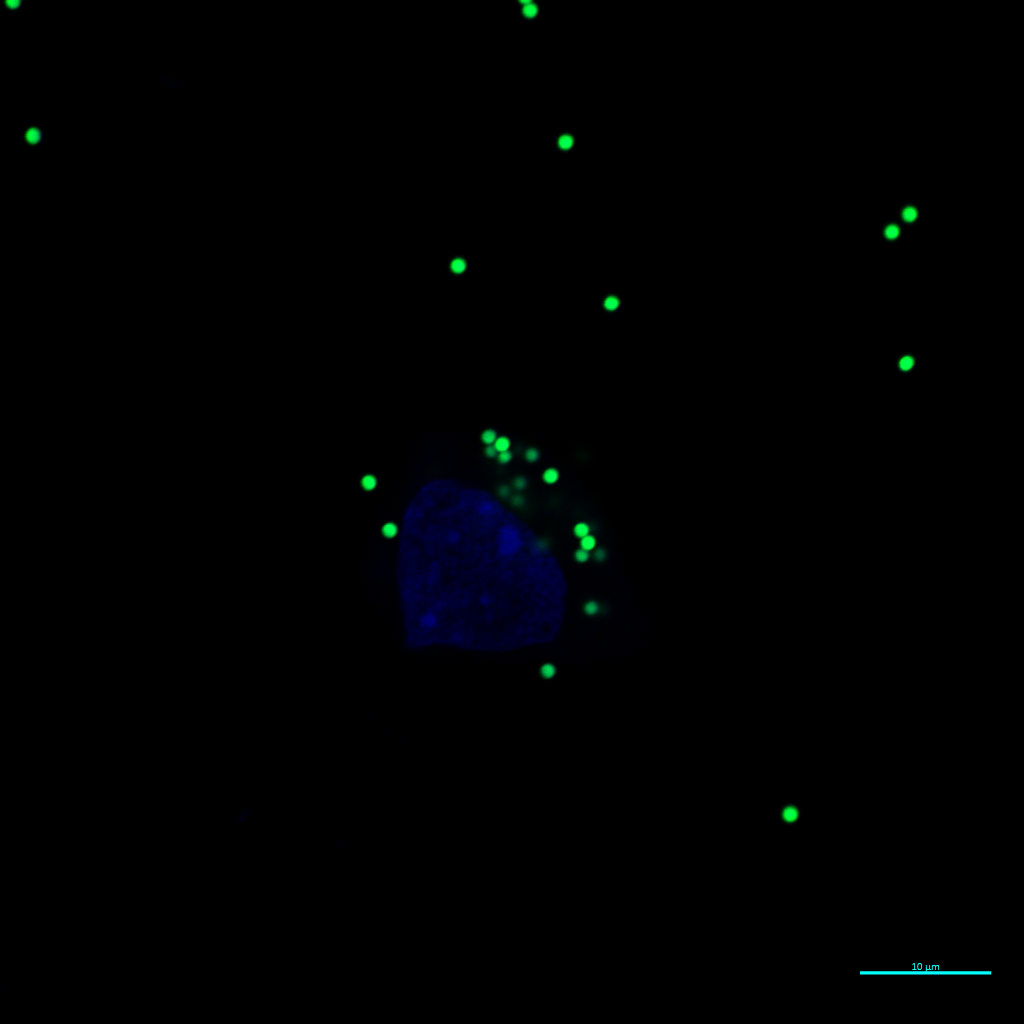

Supplement: Supplementary file 5 — Source data Fig. 4 [file 44318_2026_817_MOESM5_ESM.zip › 4C/4C-2-LLOMe-WT_Latex beads.tif]

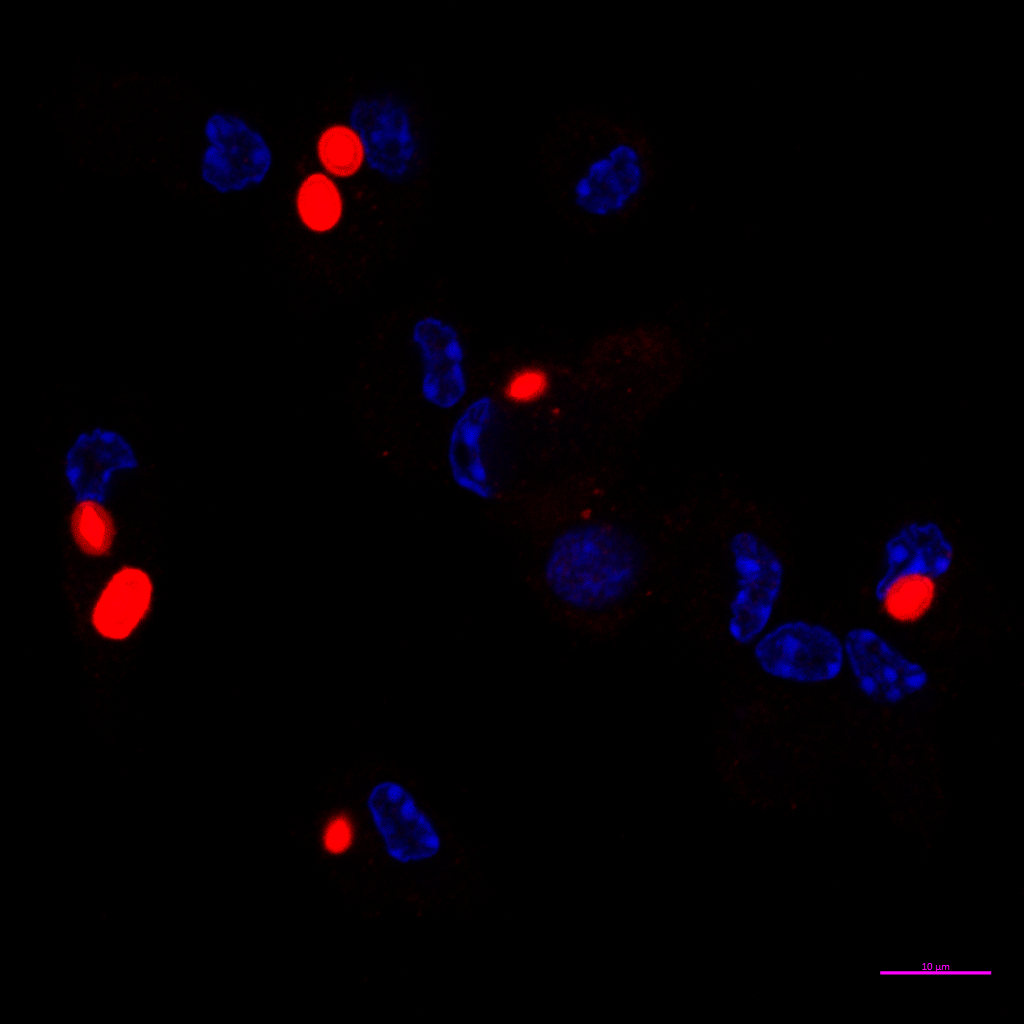

Supplement: Supplementary file 5 — Source data Fig. 4 [file 44318_2026_817_MOESM5_ESM.zip › 4E/4E-1-Basal-C9orf72 KO_ZymosanA.tif]

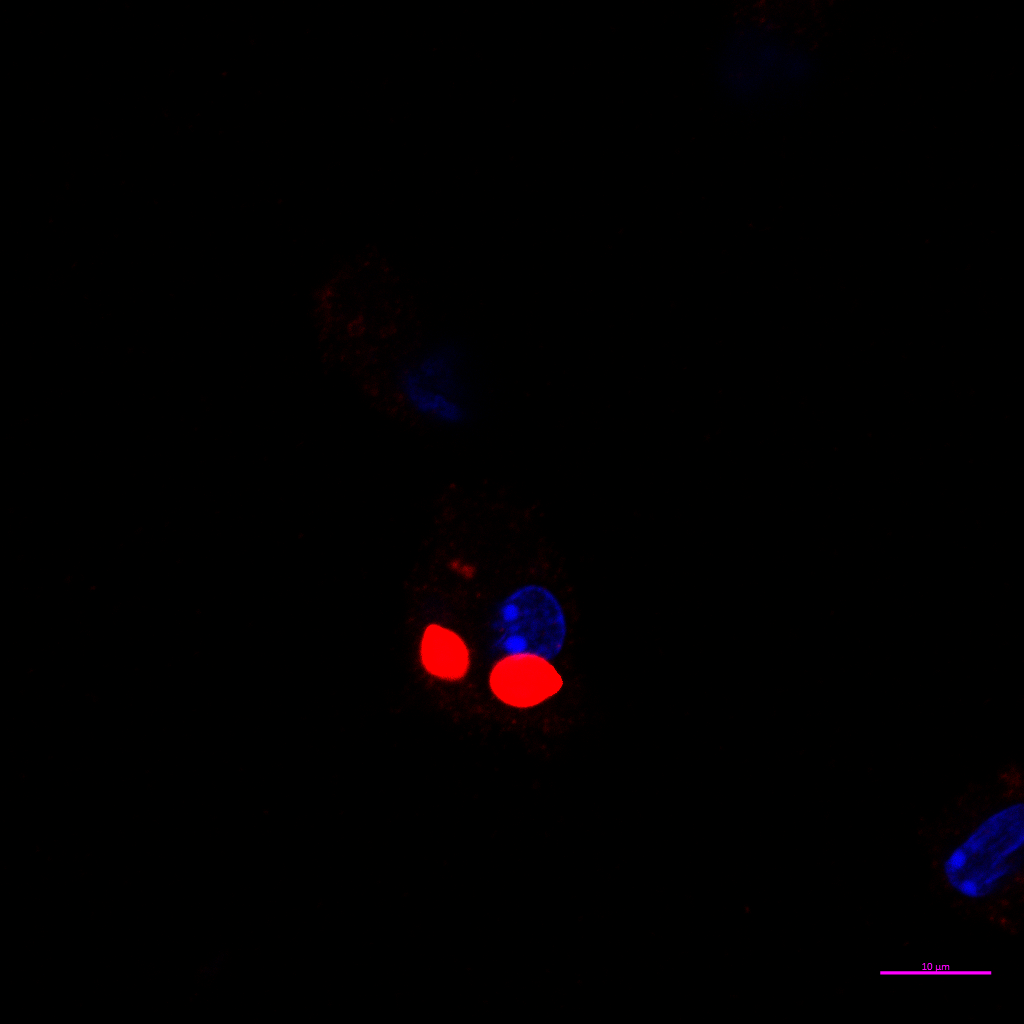

Supplement: Supplementary file 5 — Source data Fig. 4 [file 44318_2026_817_MOESM5_ESM.zip › 4E/4E-1-Basal-Smcr8 KO_ZymosanA.tif]

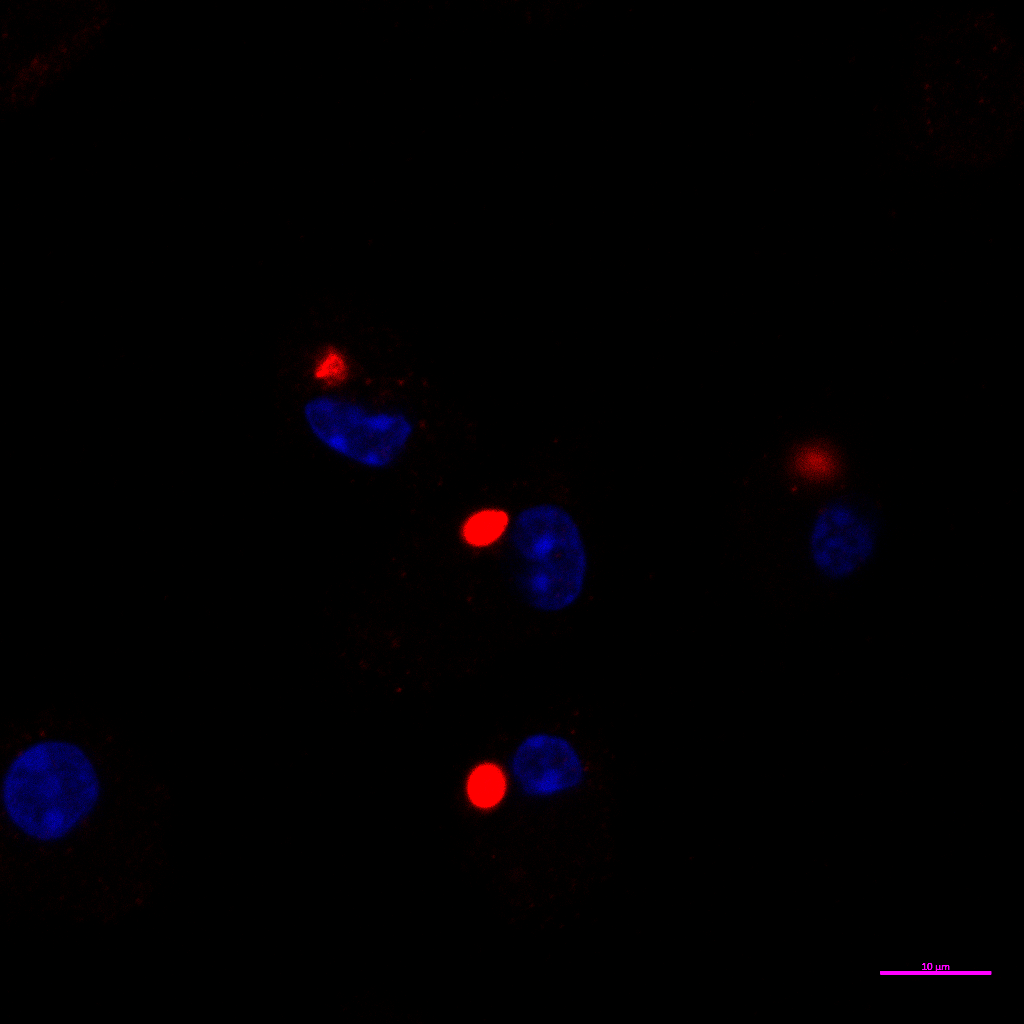

Supplement: Supplementary file 5 — Source data Fig. 4 [file 44318_2026_817_MOESM5_ESM.zip › 4E/4E-1-Basal-WT_ZymosanA.tif]

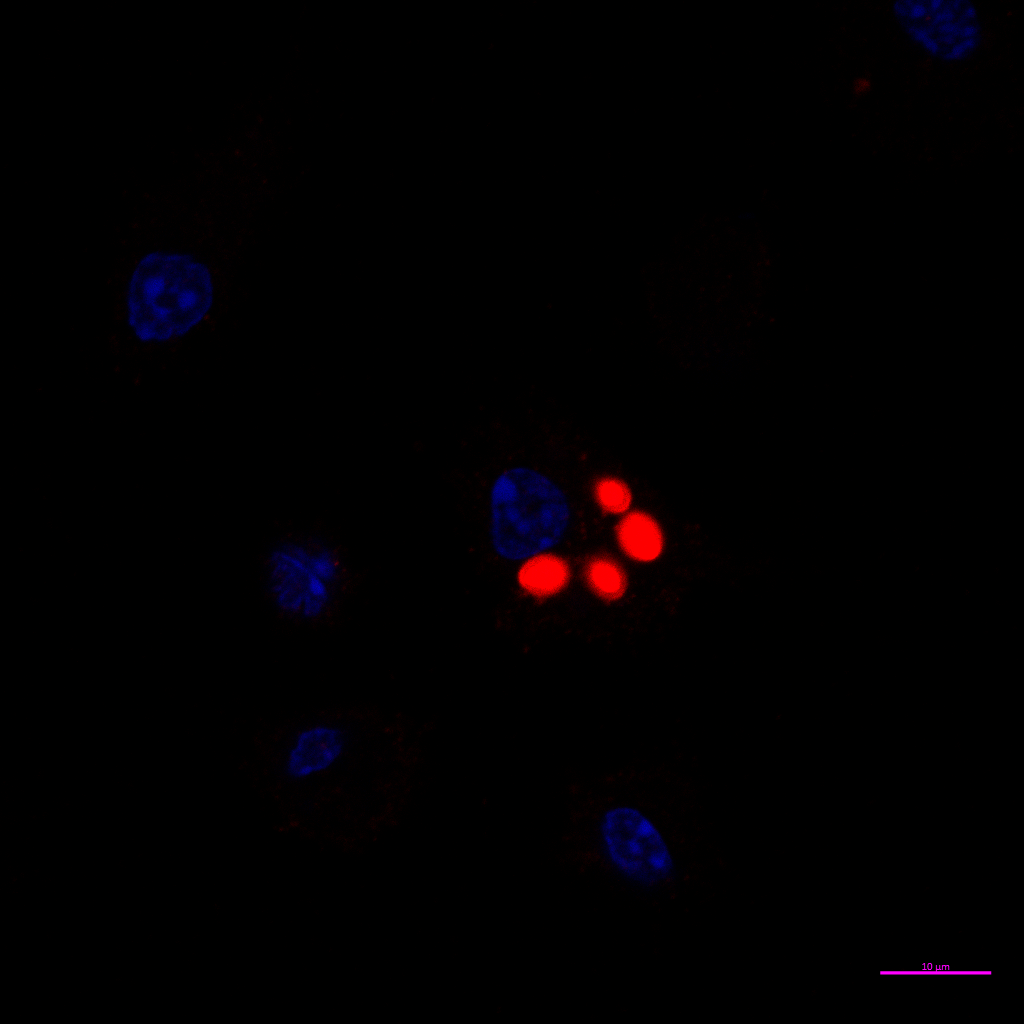

Supplement: Supplementary file 5 — Source data Fig. 4 [file 44318_2026_817_MOESM5_ESM.zip › 4E/4E-2-LLOMe-C9orf72 KO_ZymosanA.tif]

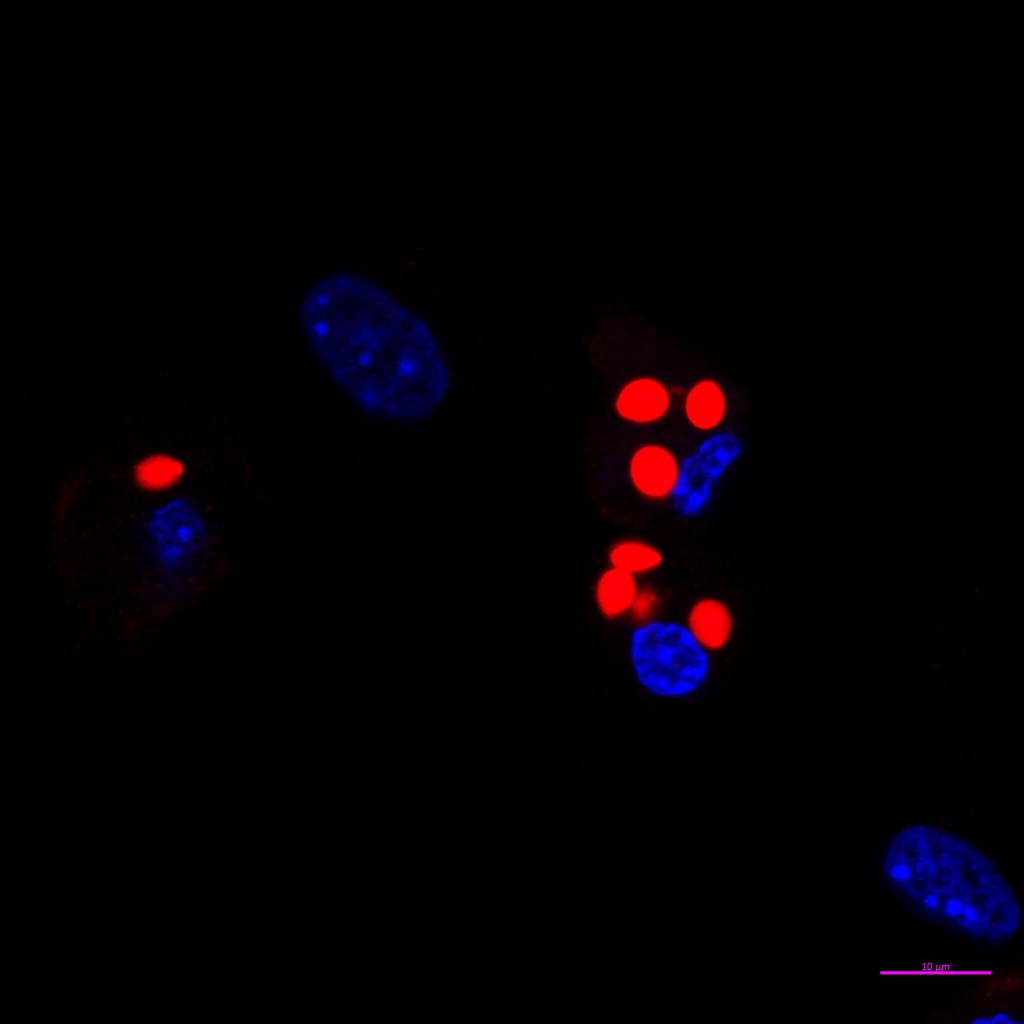

Supplement: Supplementary file 5 — Source data Fig. 4 [file 44318_2026_817_MOESM5_ESM.zip › 4E/4E-2-LLOMe-Smcr8 KO_ZymosanA.tif]

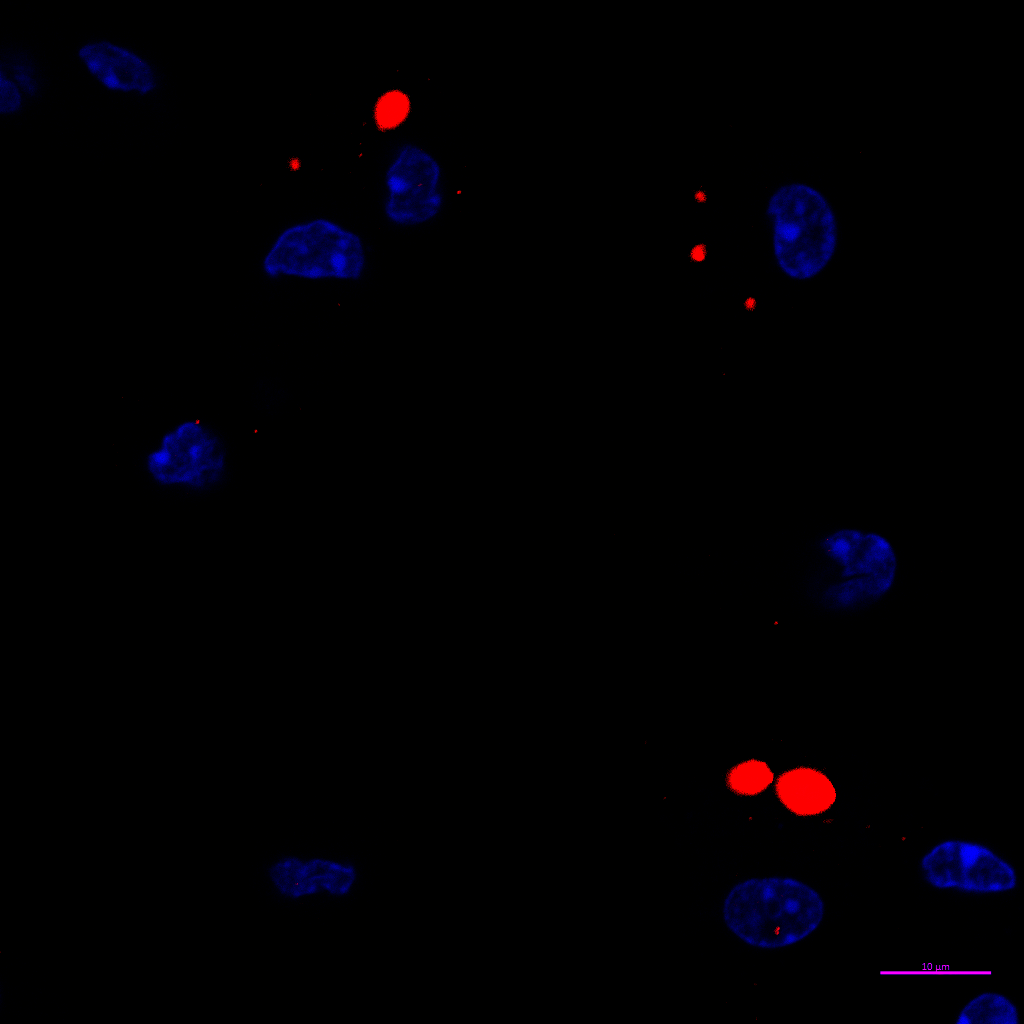

Supplement: Supplementary file 5 — Source data Fig. 4 [file 44318_2026_817_MOESM5_ESM.zip › 4E/4E-2-LLOMe-WT_ZymosanA.tif]

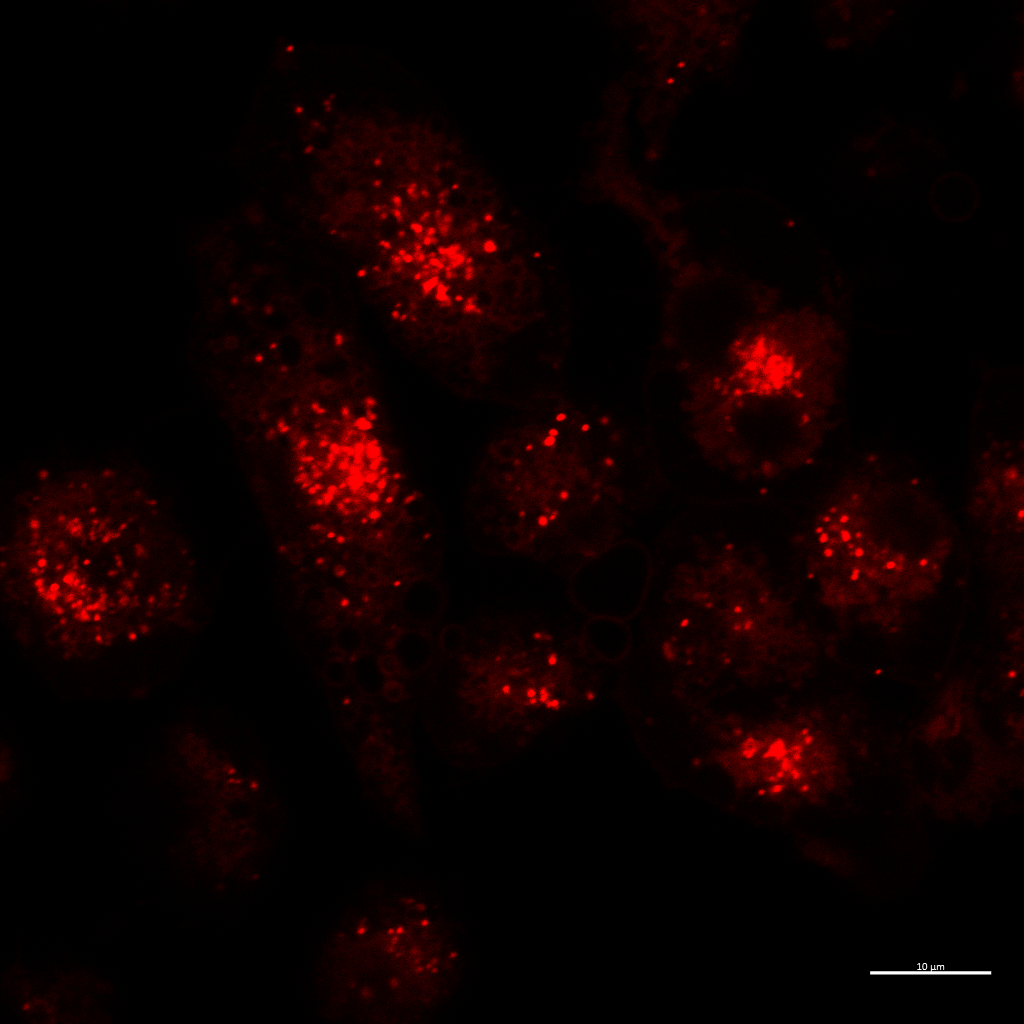

Supplement: Supplementary file 6 — Source data Fig. 5 [file 44318_2026_817_MOESM6_ESM.zip › 5A/5A-1-Basal-C9orf72 KO_Lysotracker.tif]

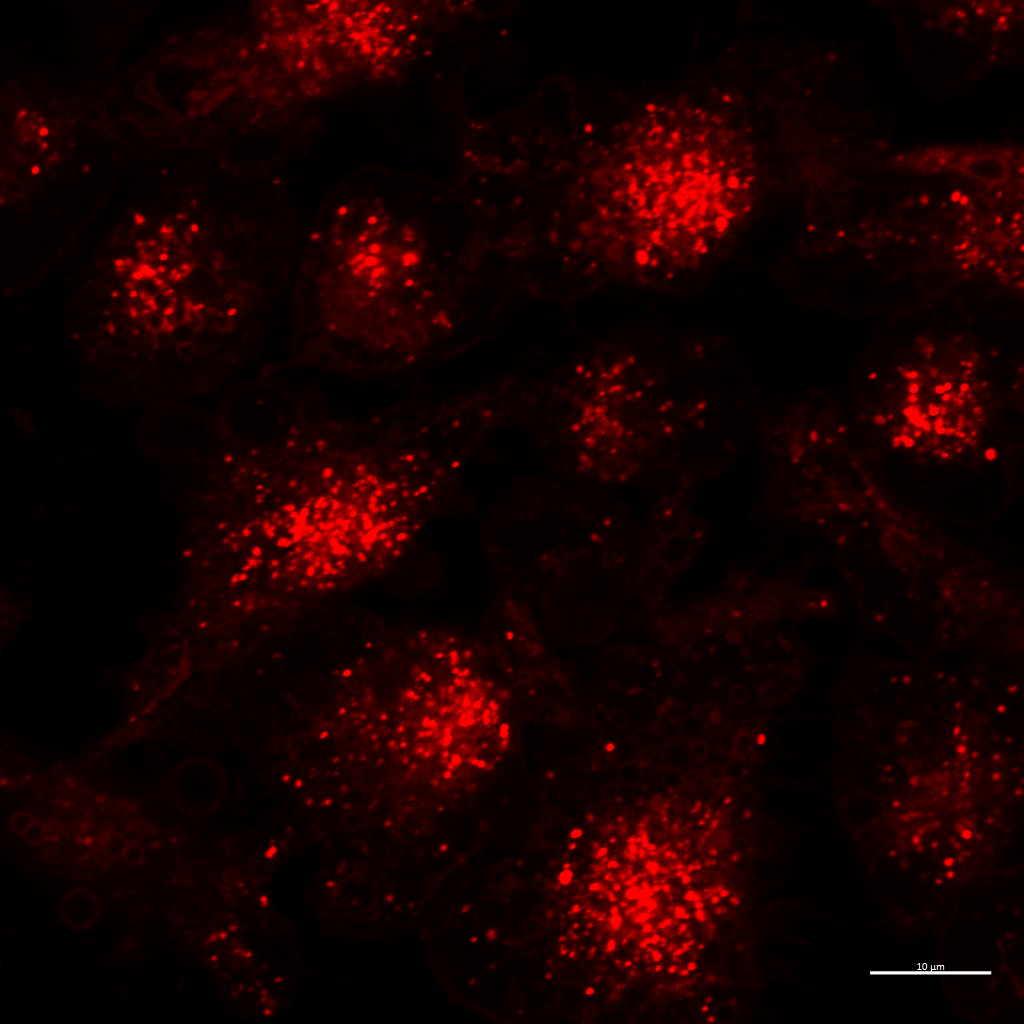

Supplement: Supplementary file 6 — Source data Fig. 5 [file 44318_2026_817_MOESM6_ESM.zip › 5A/5A-1-Basal-dKO_Lysotracker.tif]

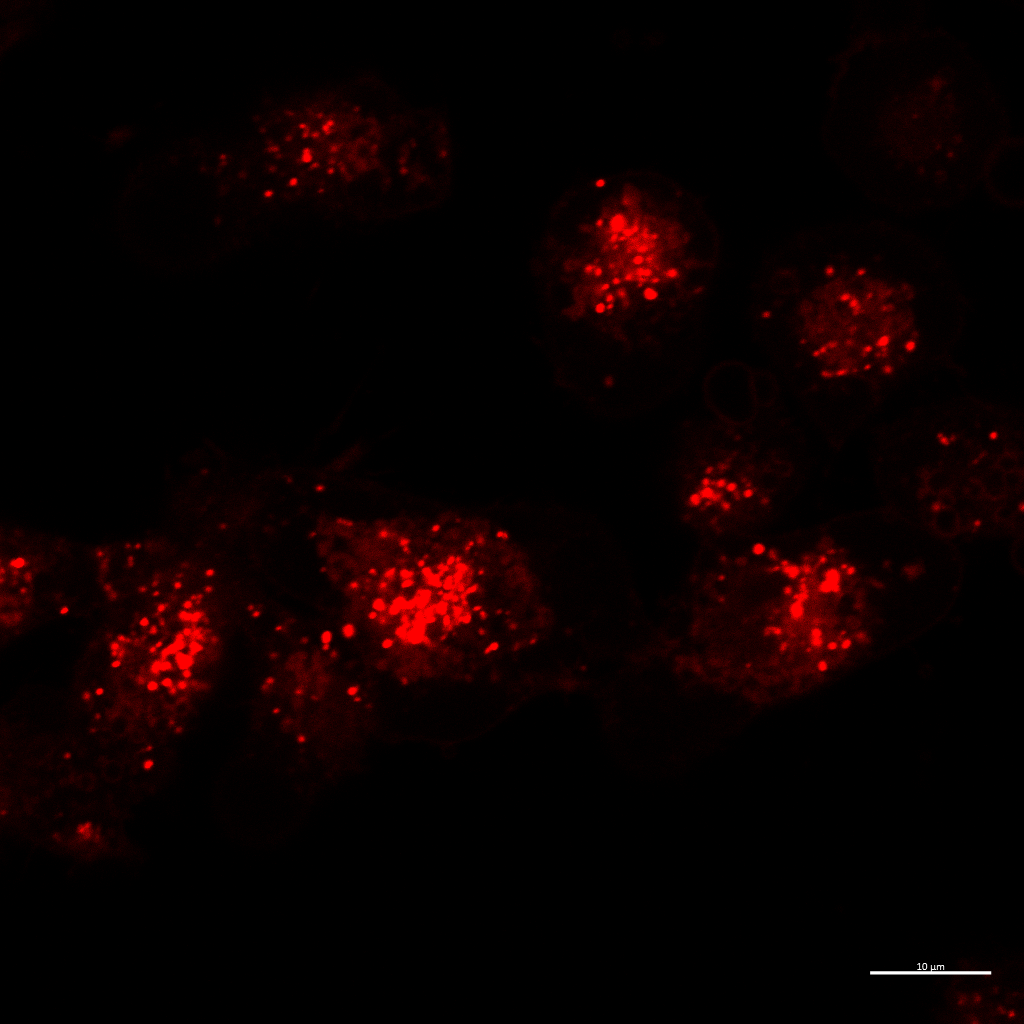

Supplement: Supplementary file 6 — Source data Fig. 5 [file 44318_2026_817_MOESM6_ESM.zip › 5A/5A-1-Basal-Smcr8 KO_Lysotracker.tif]

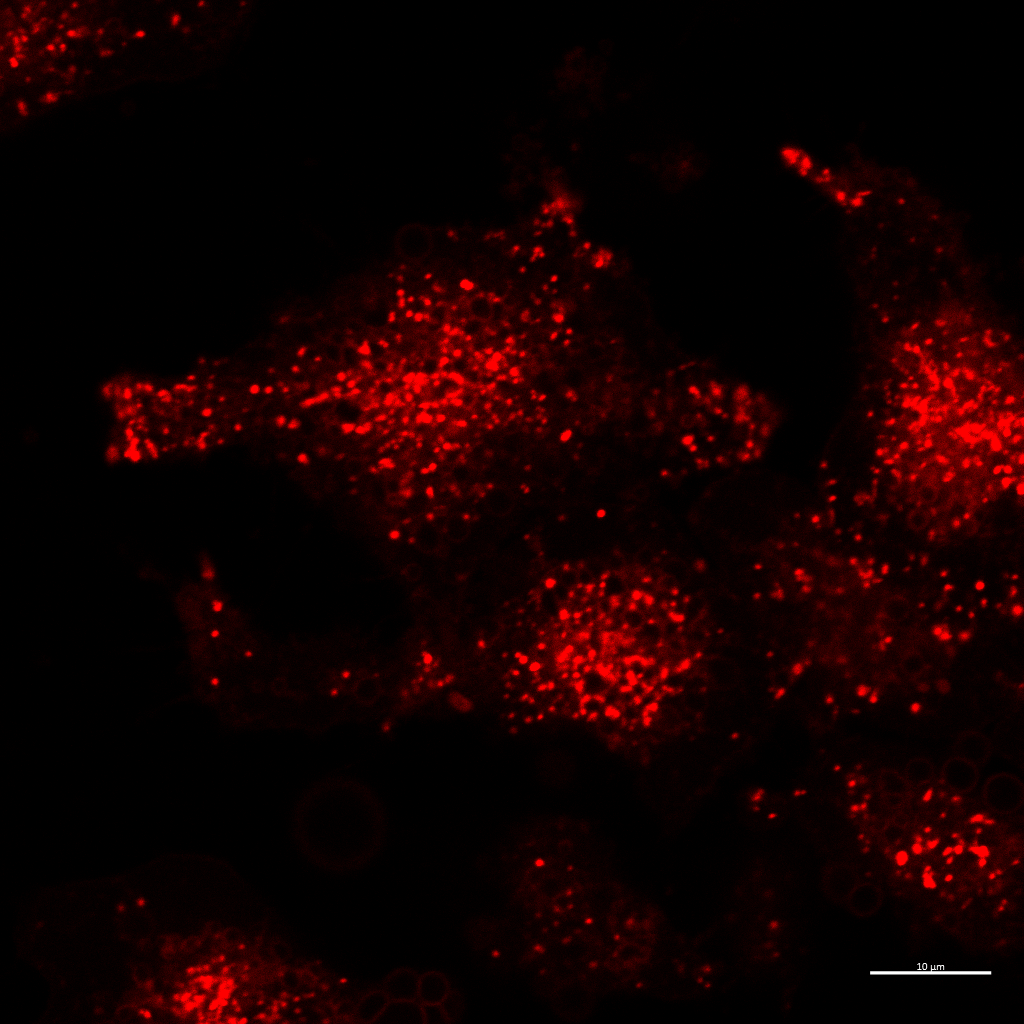

Supplement: Supplementary file 6 — Source data Fig. 5 [file 44318_2026_817_MOESM6_ESM.zip › 5A/5A-1-Basal-WT_Lysotracker.tif]

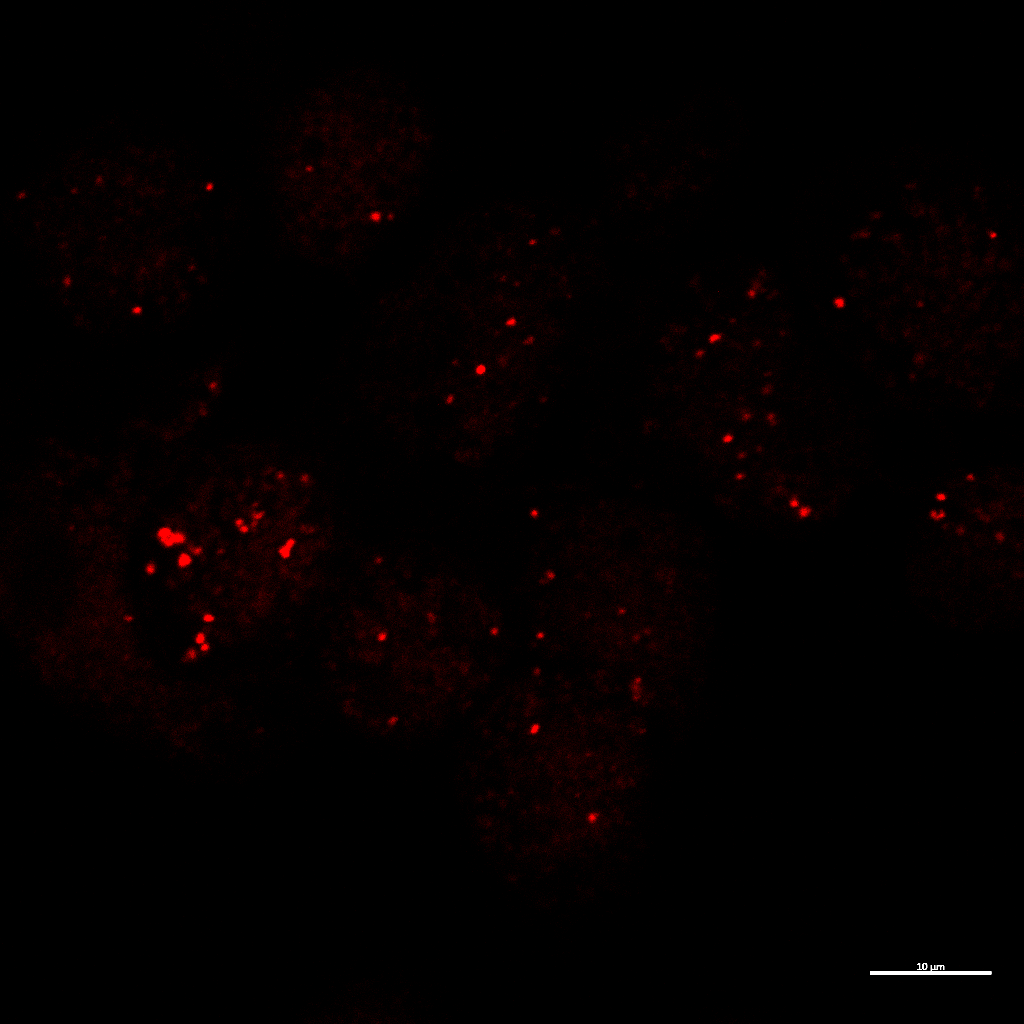

Supplement: Supplementary file 6 — Source data Fig. 5 [file 44318_2026_817_MOESM6_ESM.zip › 5A/5A-2-LLOMe 30 min-C9orf72 KO_Lysotracker.tif]

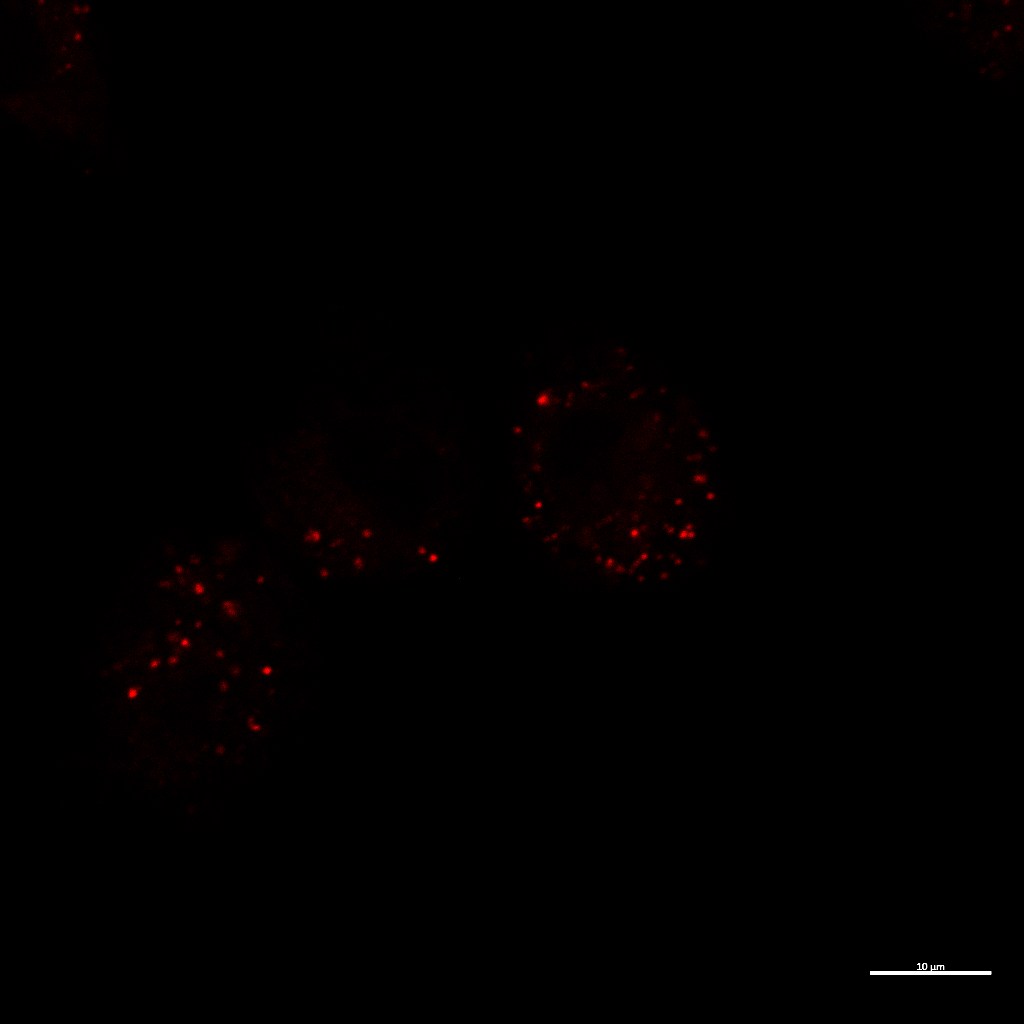

Supplement: Supplementary file 6 — Source data Fig. 5 [file 44318_2026_817_MOESM6_ESM.zip › 5A/5A-2-LLOMe 30 min-dKO_Lysotracker.tif]

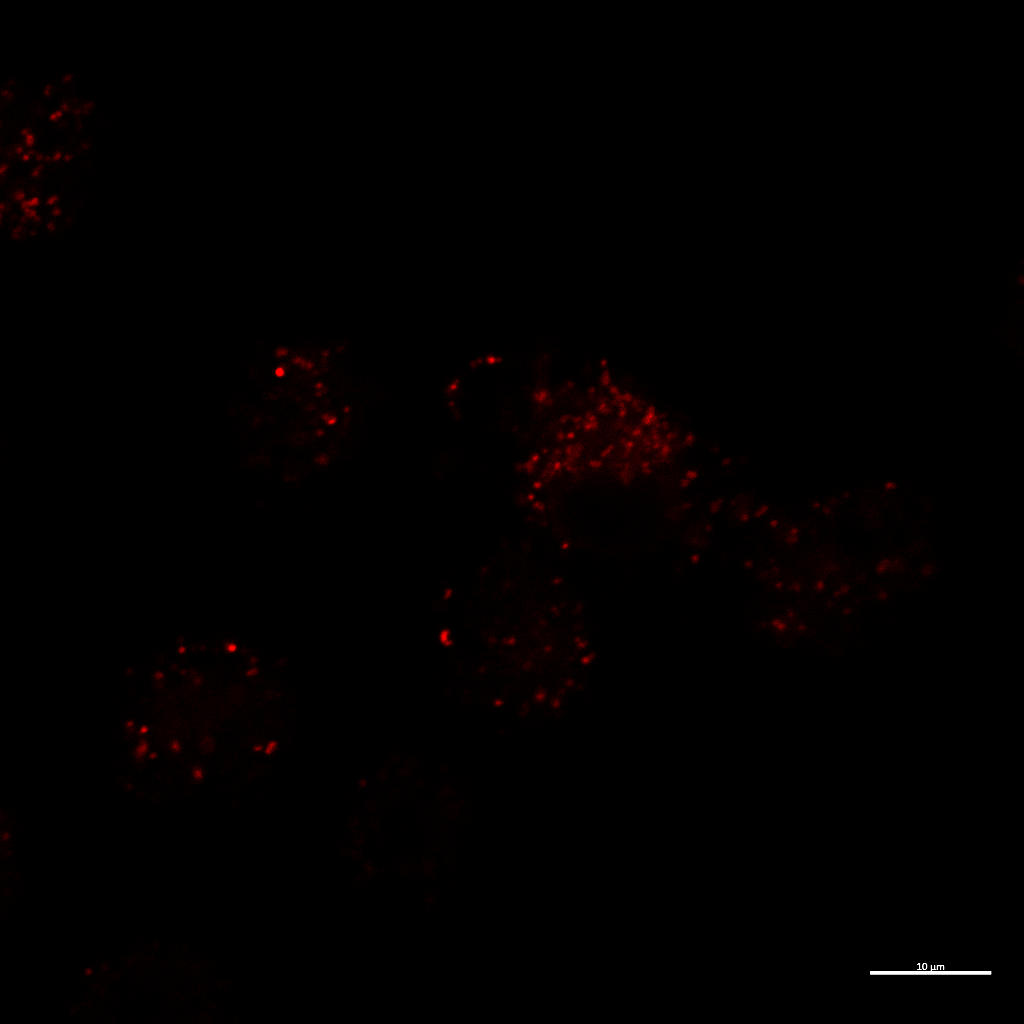

Supplement: Supplementary file 6 — Source data Fig. 5 [file 44318_2026_817_MOESM6_ESM.zip › 5A/5A-2-LLOMe 30 min-Smcr8 KO_Lysotracker.tif]

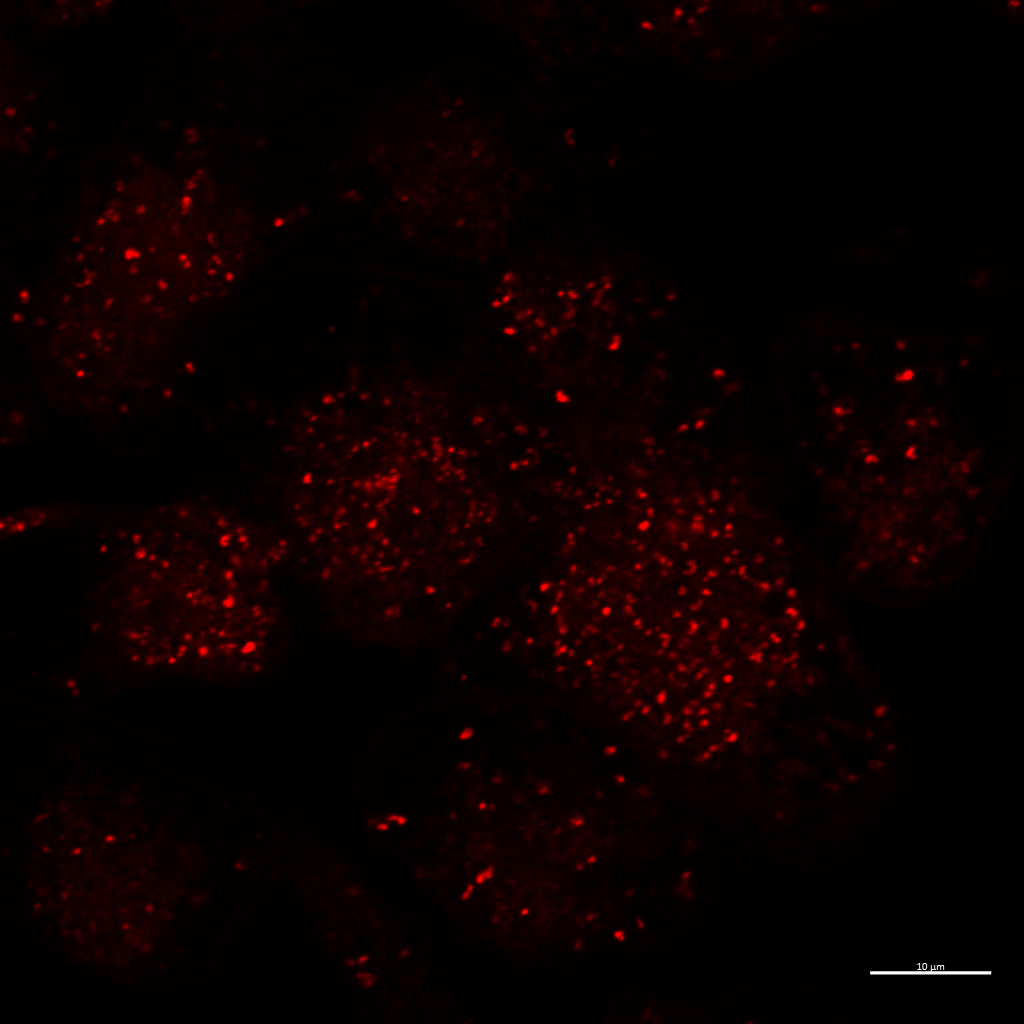

Supplement: Supplementary file 6 — Source data Fig. 5 [file 44318_2026_817_MOESM6_ESM.zip › 5A/5A-2-LLOMe 30 min-WT_Lysotracker.tif]

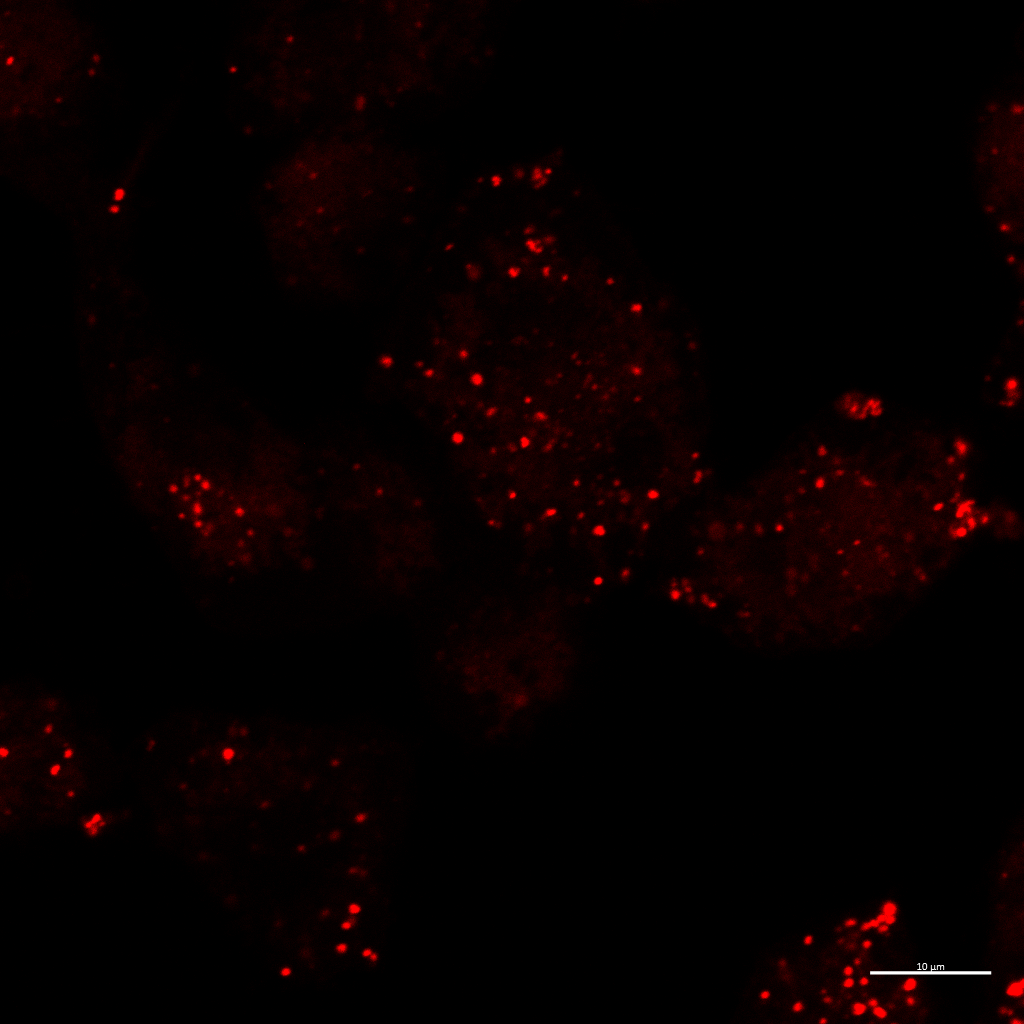

Supplement: Supplementary file 6 — Source data Fig. 5 [file 44318_2026_817_MOESM6_ESM.zip › 5A/5A-3-Washout 3 h-C9orf72 KO_Lysotracker.tif]

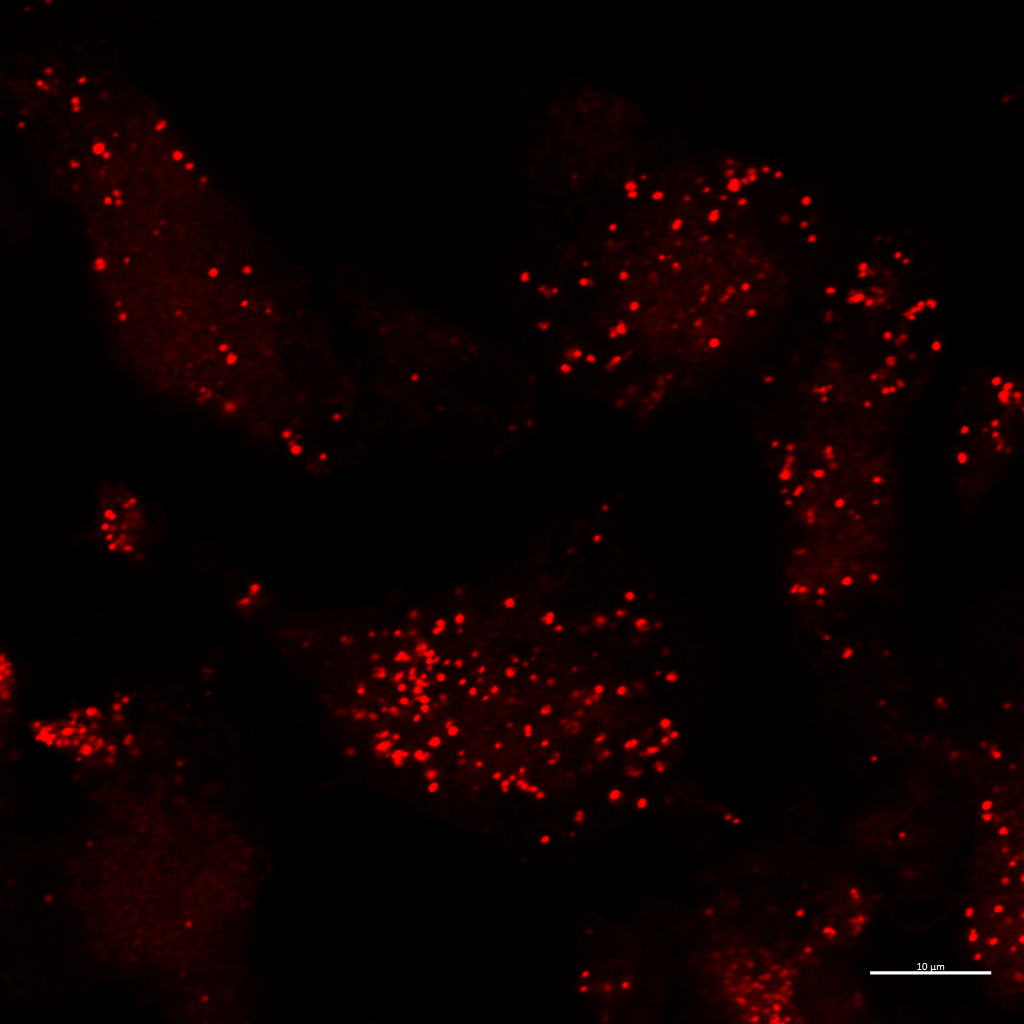

Supplement: Supplementary file 6 — Source data Fig. 5 [file 44318_2026_817_MOESM6_ESM.zip › 5A/5A-3-Washout 3 h-dKO_Lysotracker.tif]

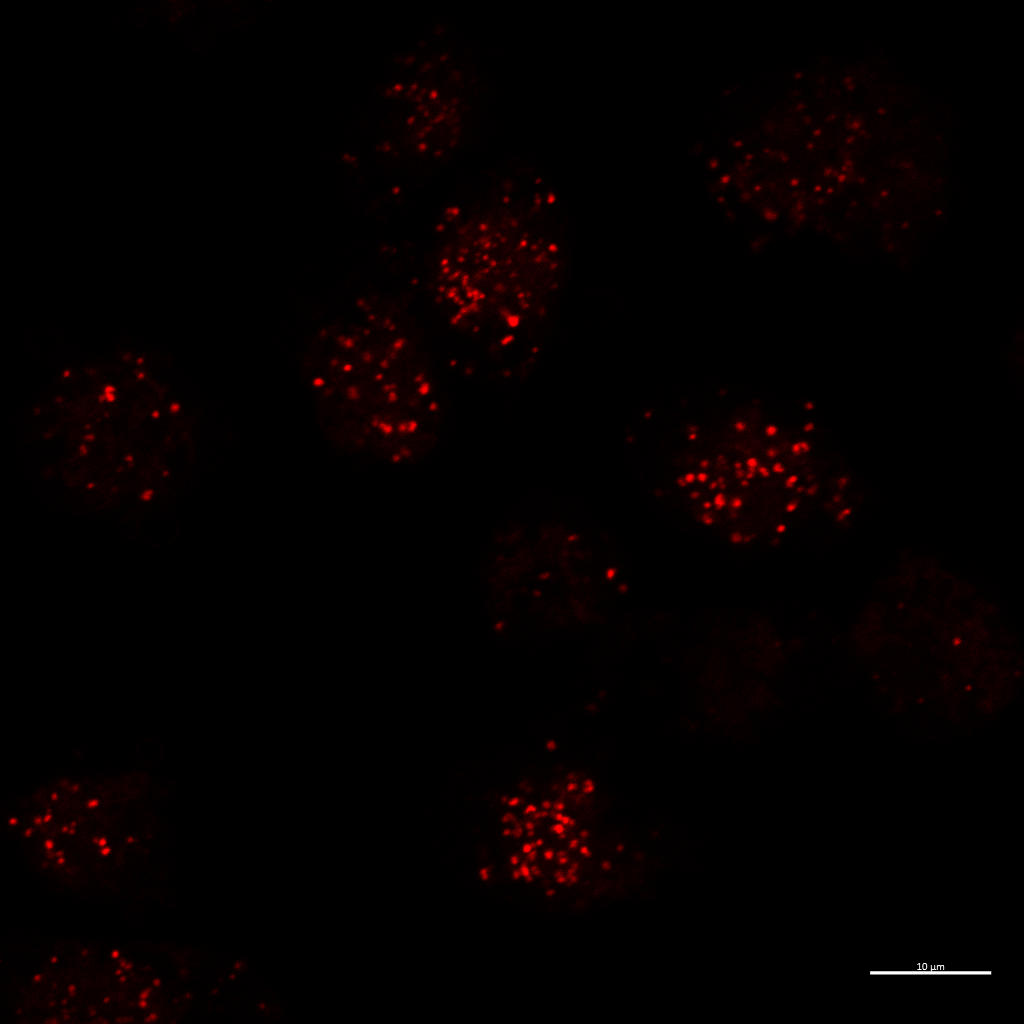

Supplement: Supplementary file 6 — Source data Fig. 5 [file 44318_2026_817_MOESM6_ESM.zip › 5A/5A-3-Washout 3 h-Smcr8 KO_Lysotracker.tif]

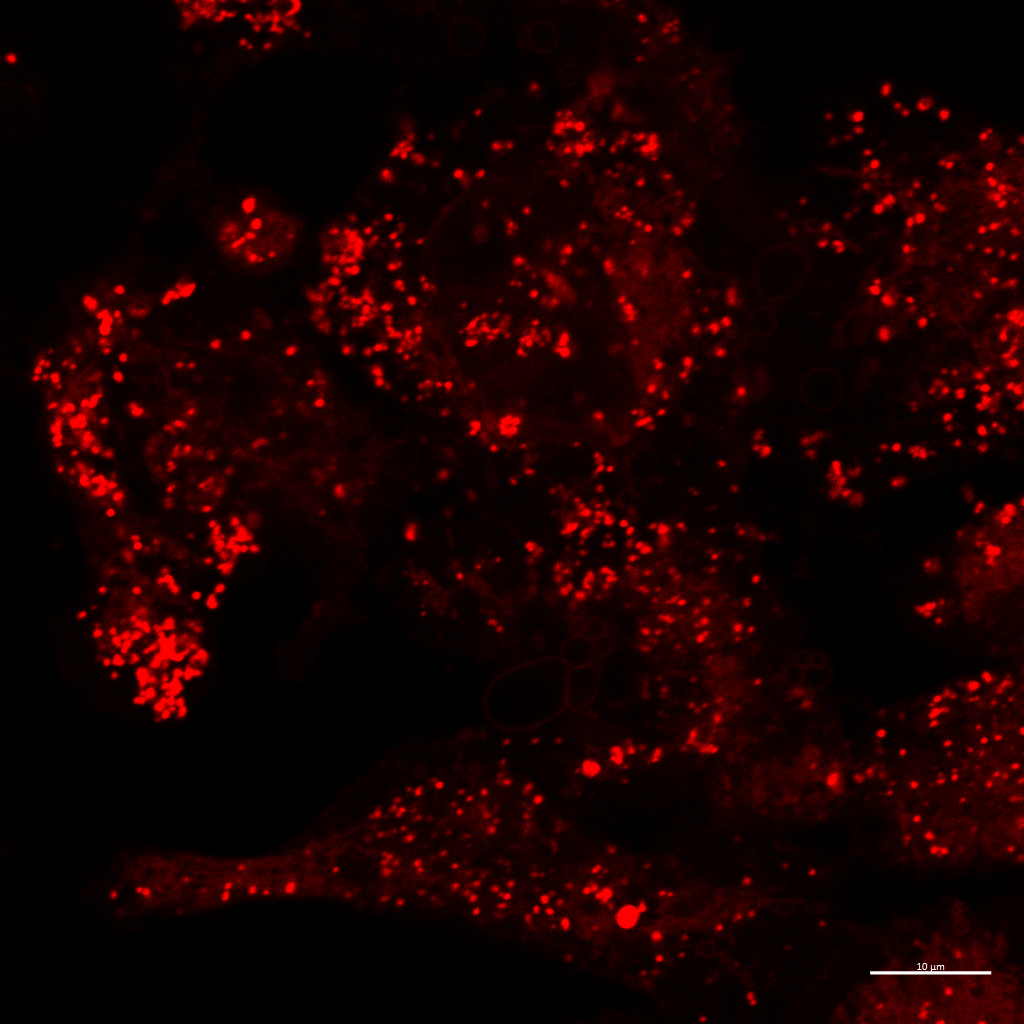

Supplement: Supplementary file 6 — Source data Fig. 5 [file 44318_2026_817_MOESM6_ESM.zip › 5A/5A-3-Washout 3 h-WT_Lysotracker.tif]

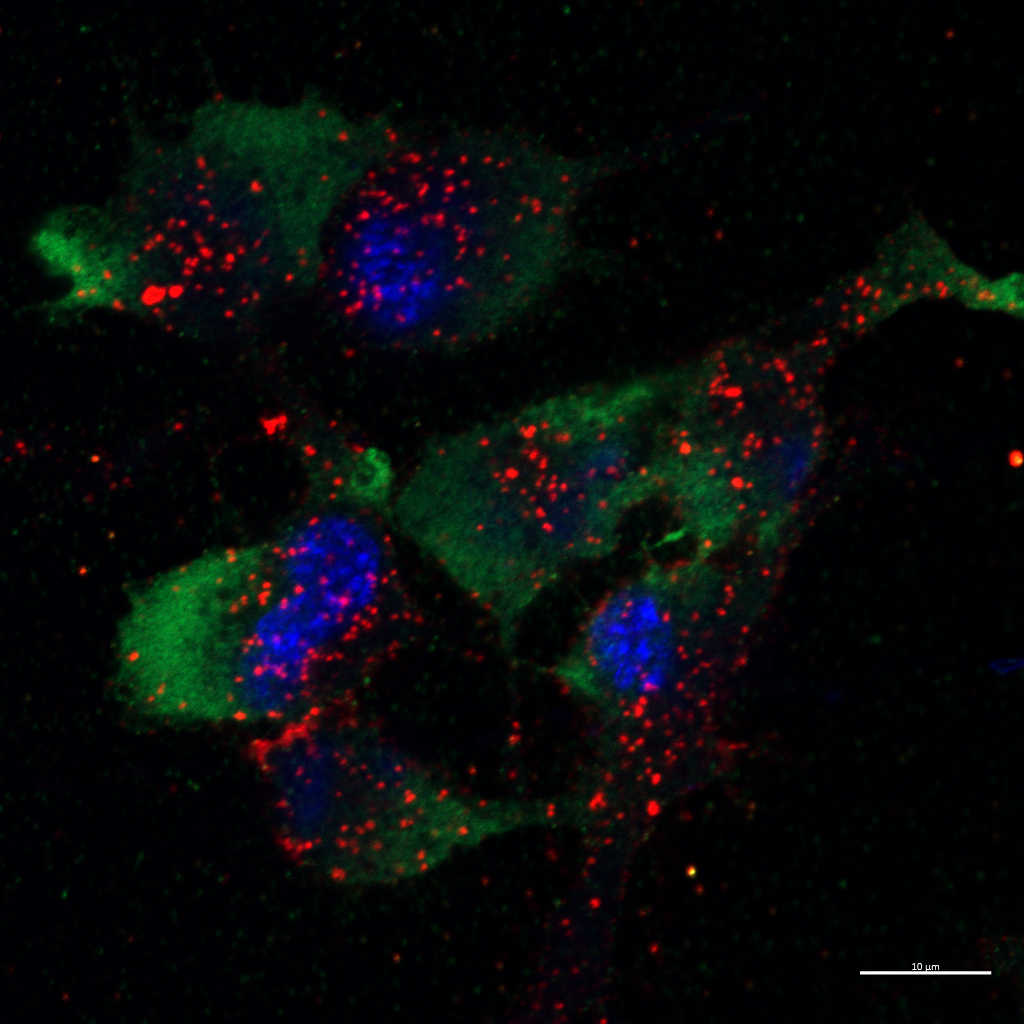

Supplement: Supplementary file 6 — Source data Fig. 5 [file 44318_2026_817_MOESM6_ESM.zip › 5C/5C-1-Basal-C9orf72 KO.tif]

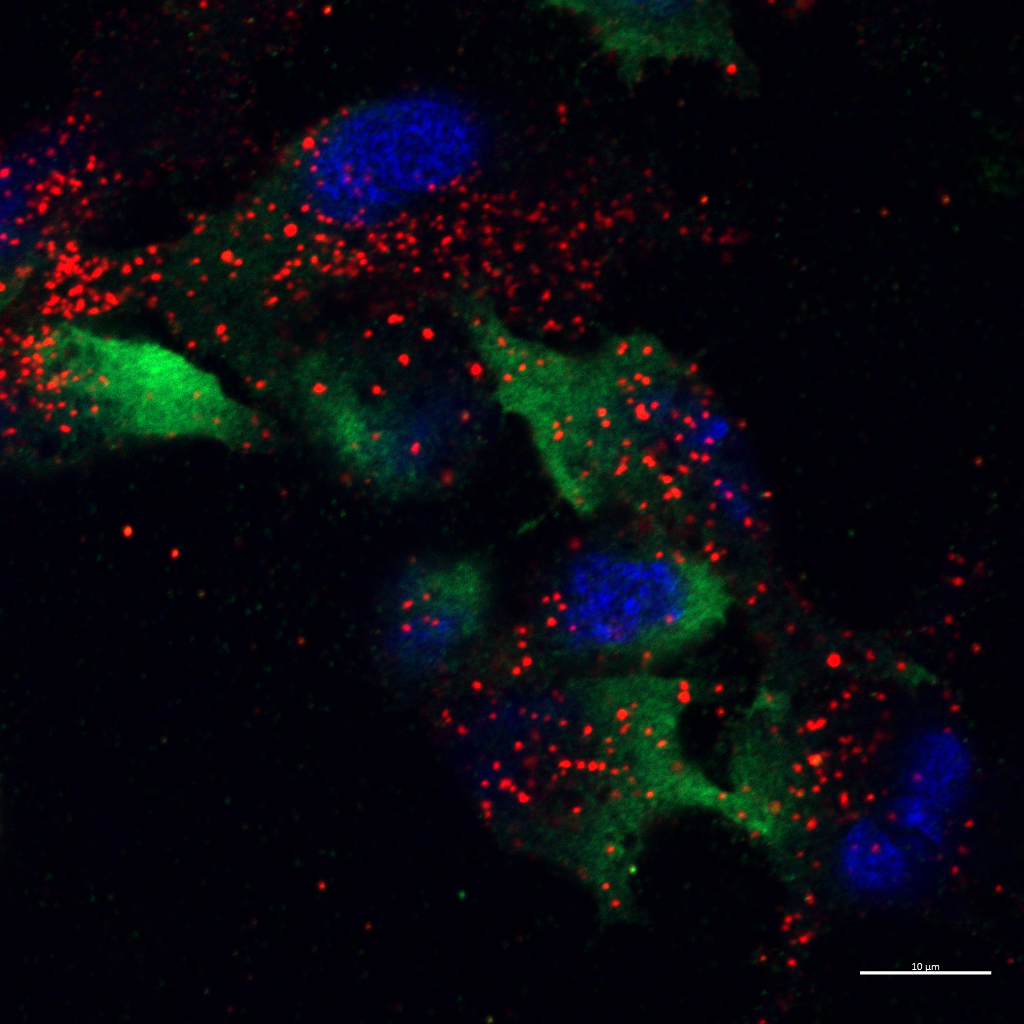

Supplement: Supplementary file 6 — Source data Fig. 5 [file 44318_2026_817_MOESM6_ESM.zip › 5C/5C-1-Basal-dKO.tif]

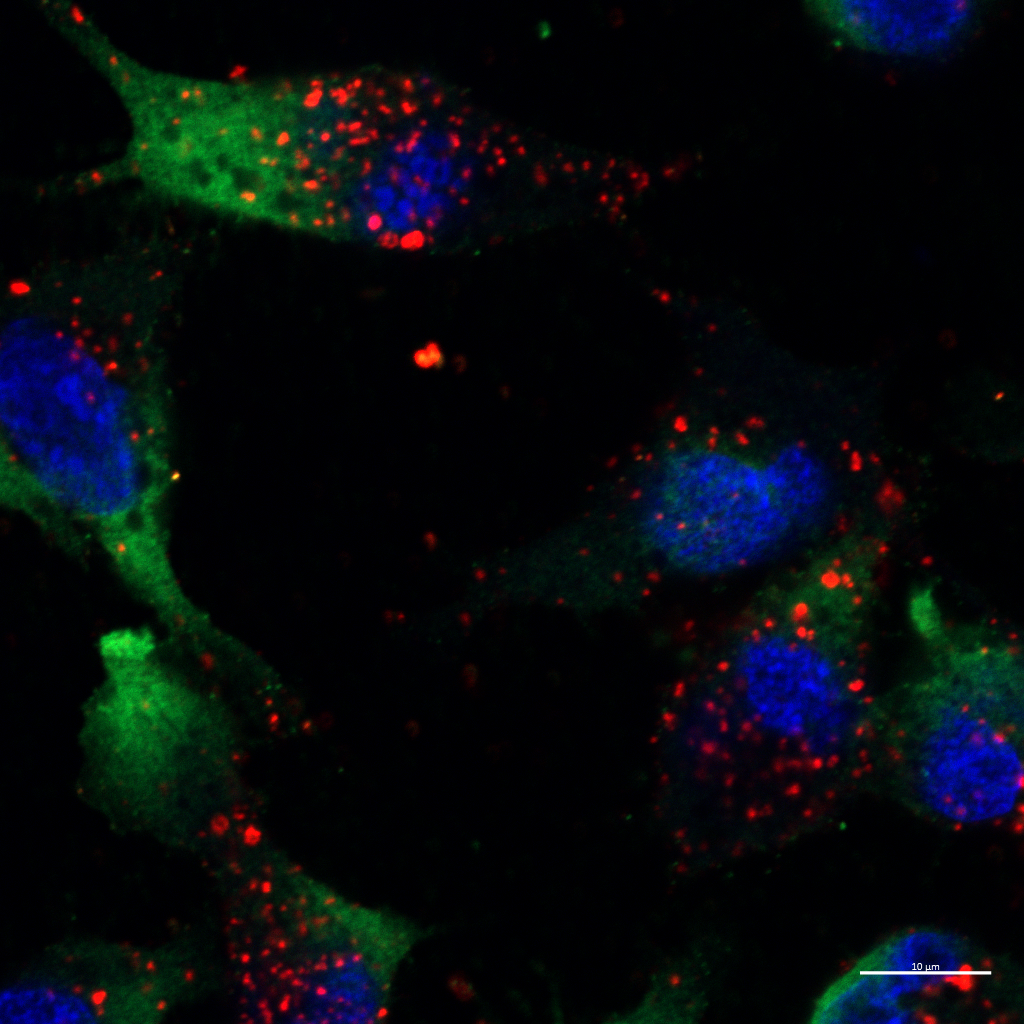

Supplement: Supplementary file 6 — Source data Fig. 5 [file 44318_2026_817_MOESM6_ESM.zip › 5C/5C-1-Basal-Smcr8 KO.tif]

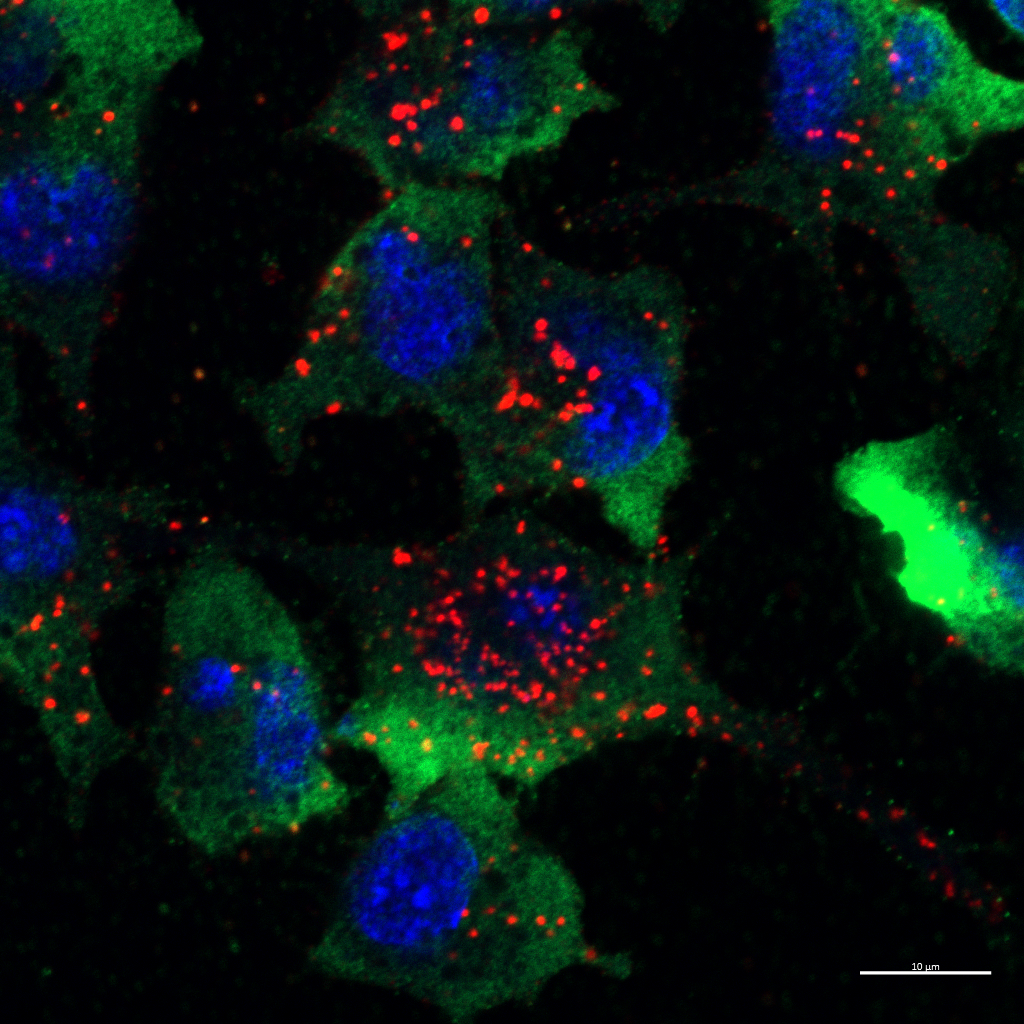

Supplement: Supplementary file 6 — Source data Fig. 5 [file 44318_2026_817_MOESM6_ESM.zip › 5C/5C-1-Basal-WT.tif]

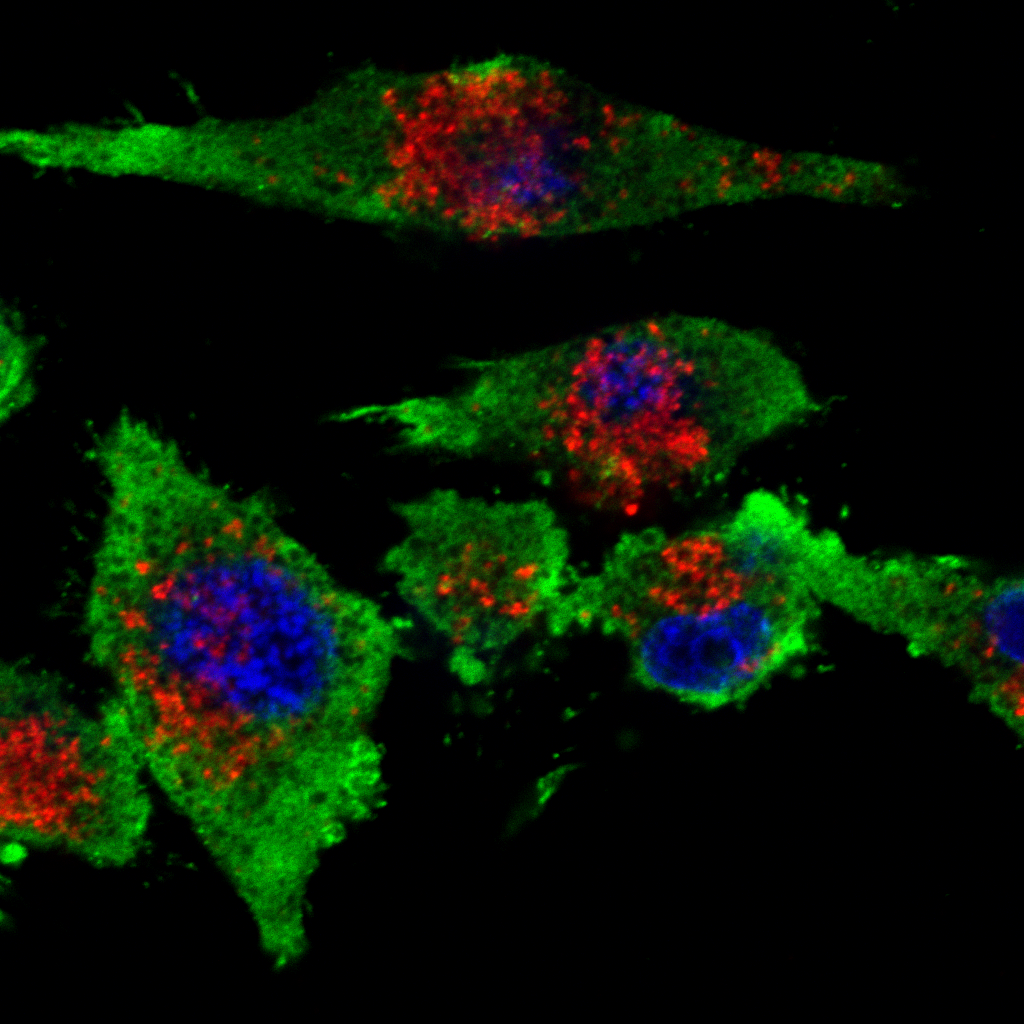

Supplement: Supplementary file 6 — Source data Fig. 5 [file 44318_2026_817_MOESM6_ESM.zip › 5C/5C-2-LLOMe 10 min-C9orf72 KO.tif]

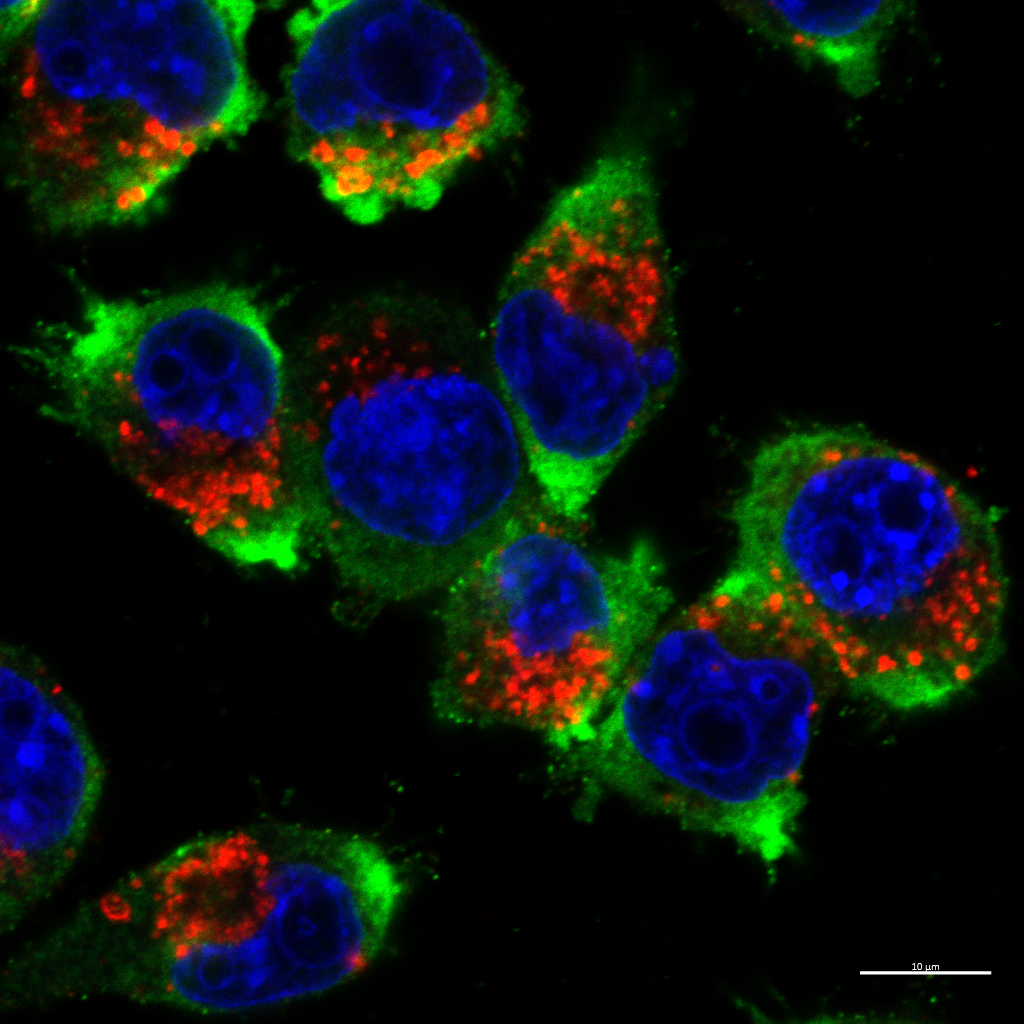

Supplement: Supplementary file 6 — Source data Fig. 5 [file 44318_2026_817_MOESM6_ESM.zip › 5C/5C-2-LLOMe 10 min-dKO.tif]

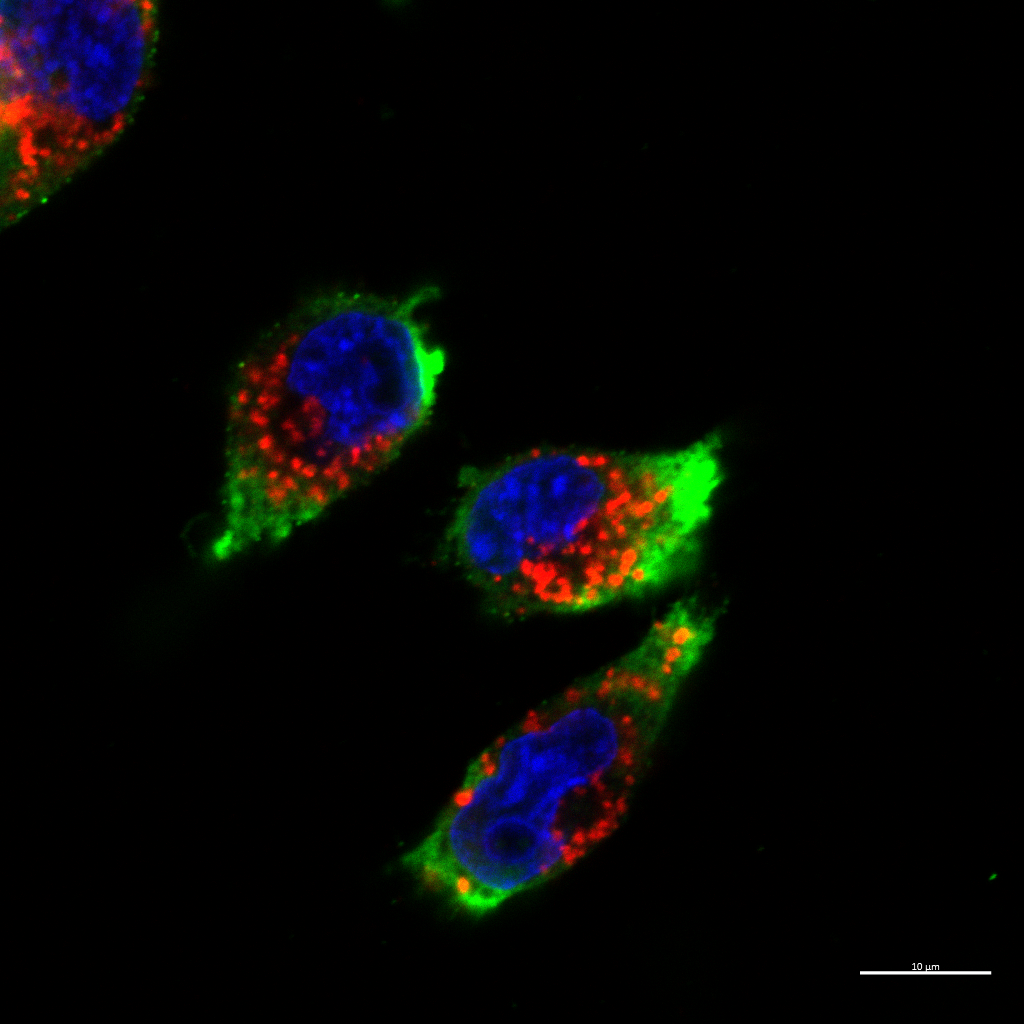

Supplement: Supplementary file 6 — Source data Fig. 5 [file 44318_2026_817_MOESM6_ESM.zip › 5C/5C-2-LLOMe 10 min-Smcr8 KO.tif]

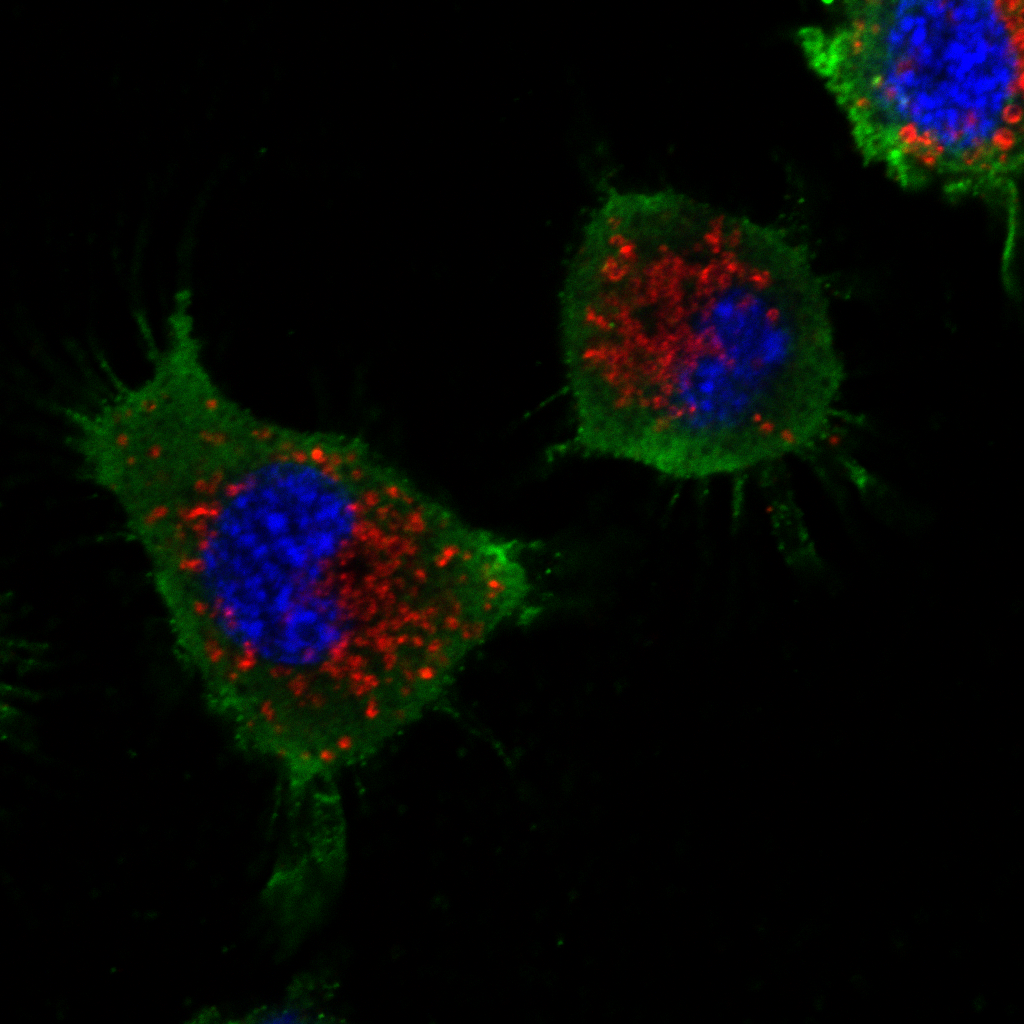

Supplement: Supplementary file 6 — Source data Fig. 5 [file 44318_2026_817_MOESM6_ESM.zip › 5C/5C-2-LLOMe 10 min-WT.tif]

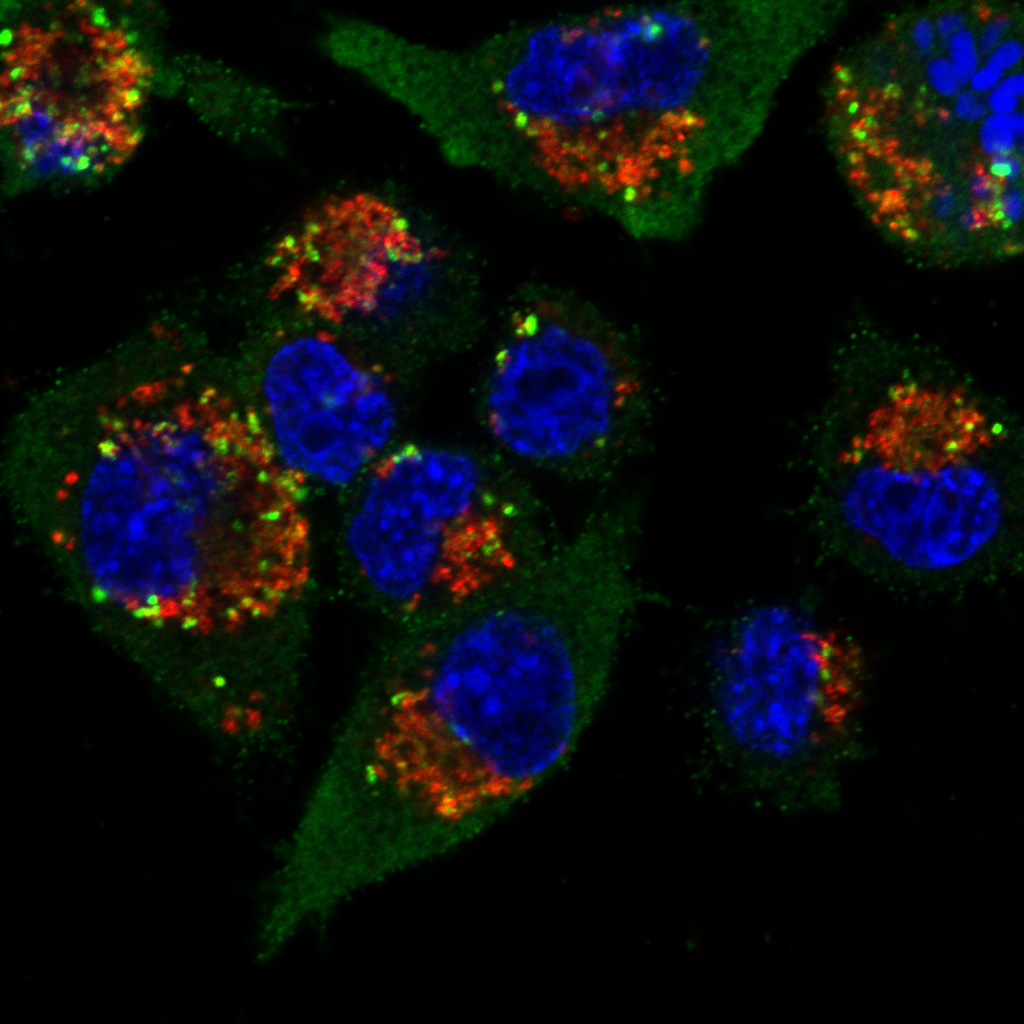

Supplement: Supplementary file 6 — Source data Fig. 5 [file 44318_2026_817_MOESM6_ESM.zip › 5C/5C-3-LLOMe 30 min-C9orf72 KO.tif]

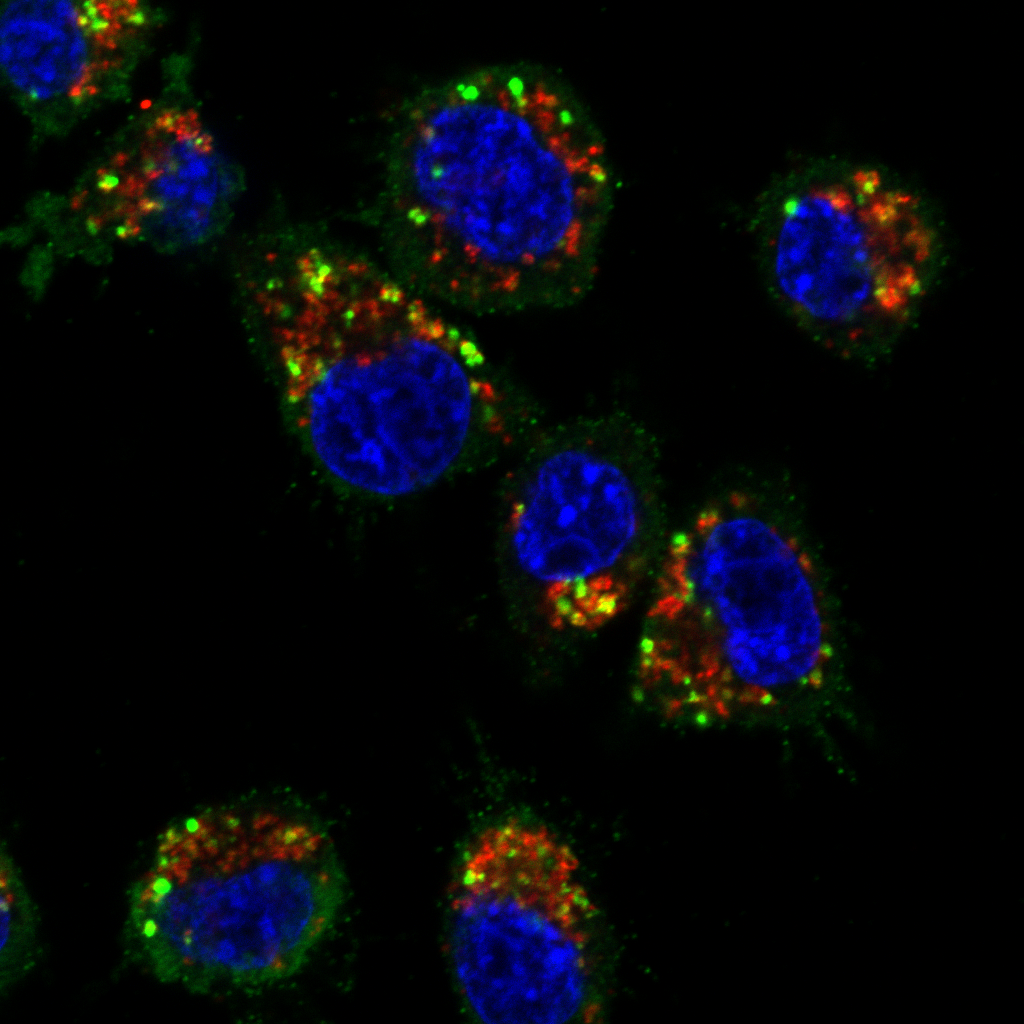

Supplement: Supplementary file 6 — Source data Fig. 5 [file 44318_2026_817_MOESM6_ESM.zip › 5C/5C-3-LLOMe 30 min-dKO.tif]

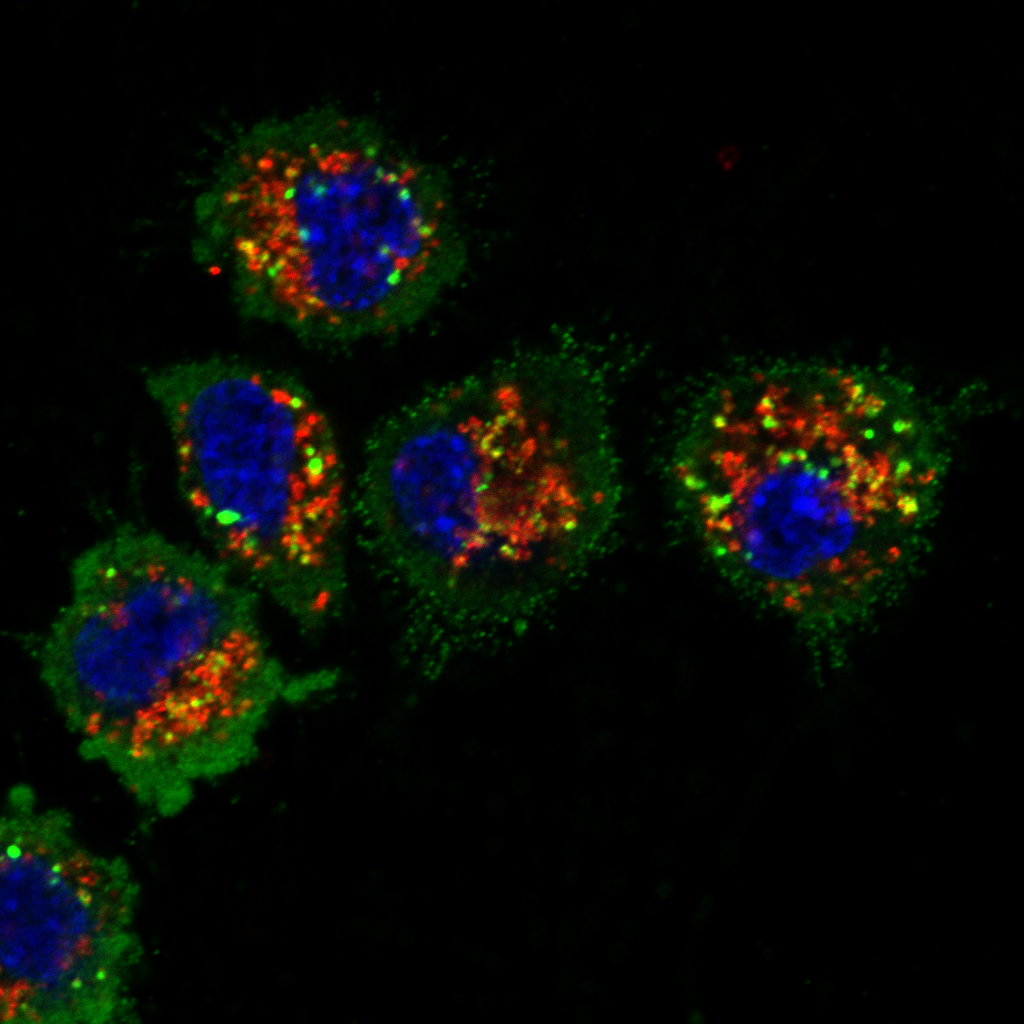

Supplement: Supplementary file 6 — Source data Fig. 5 [file 44318_2026_817_MOESM6_ESM.zip › 5C/5C-3-LLOMe 30 min-Smcr8 KO.tif]

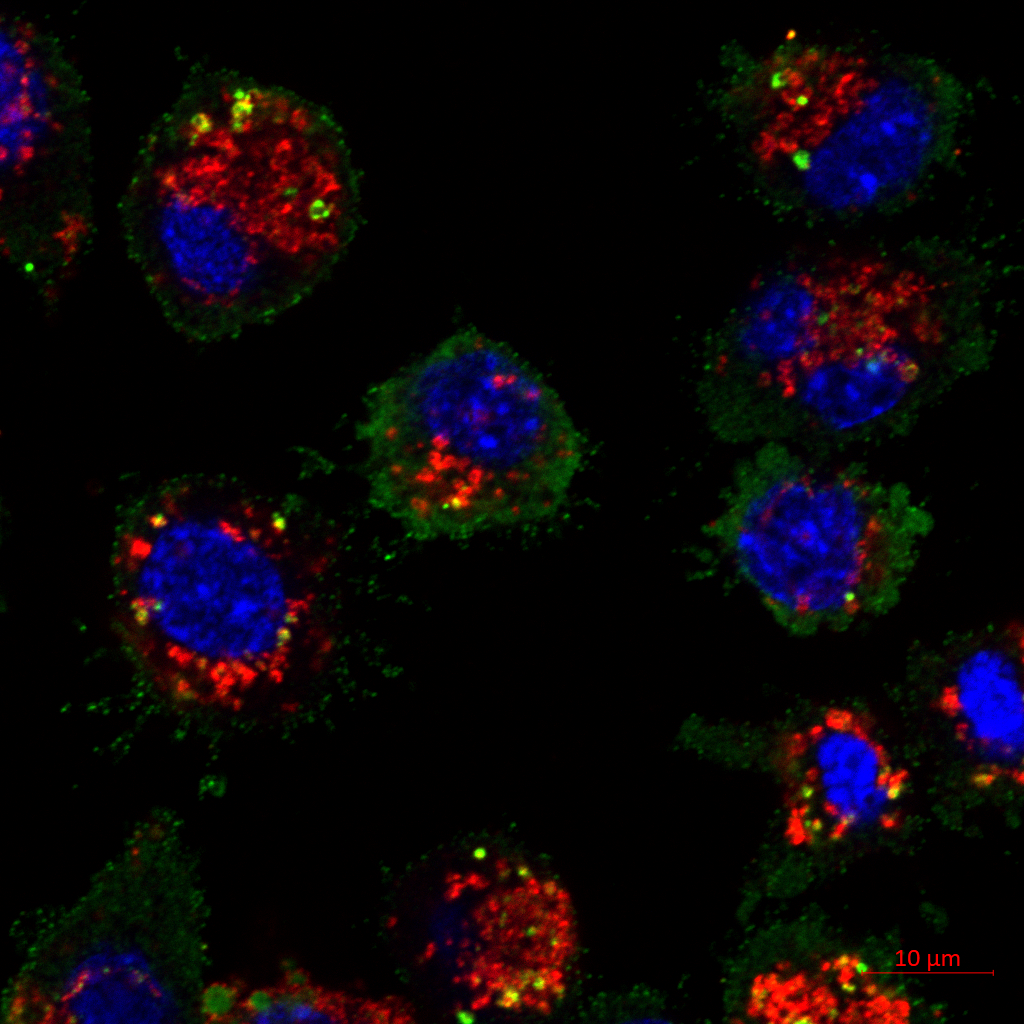

Supplement: Supplementary file 6 — Source data Fig. 5 [file 44318_2026_817_MOESM6_ESM.zip › 5C/5C-3-LLOMe 30 min-WT.tif]

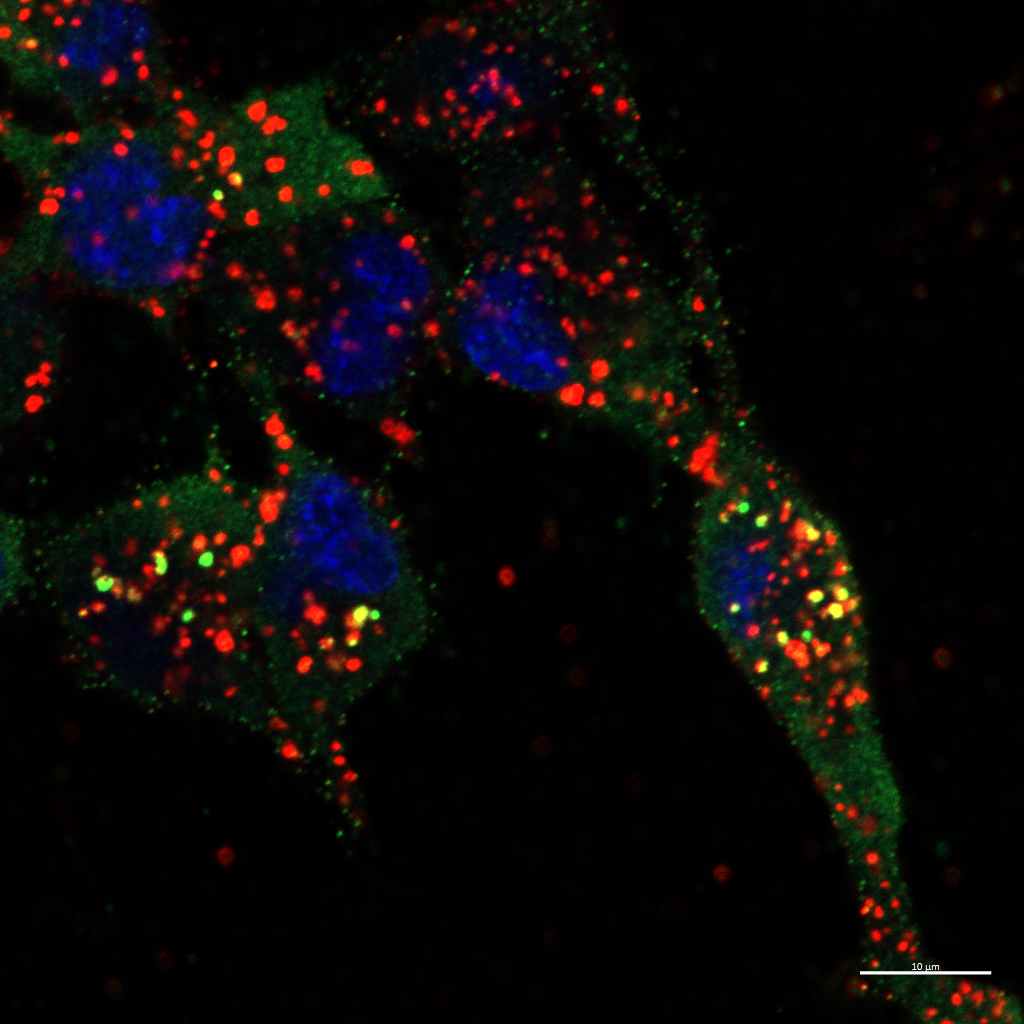

Supplement: Supplementary file 6 — Source data Fig. 5 [file 44318_2026_817_MOESM6_ESM.zip › 5C/5C-4-Washout 3 h -C9orf72 KO.tif]

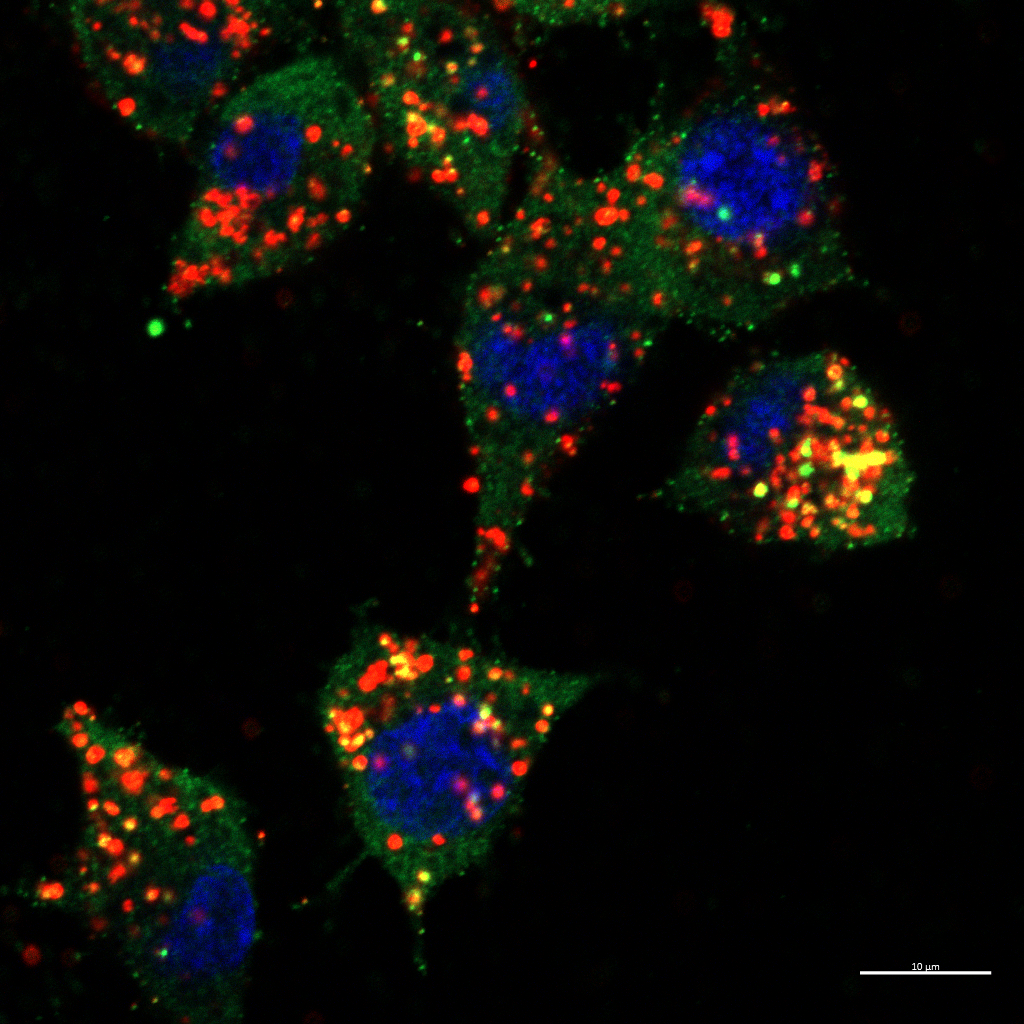

Supplement: Supplementary file 6 — Source data Fig. 5 [file 44318_2026_817_MOESM6_ESM.zip › 5C/5C-4-Washout 3 h-dKO.tif]

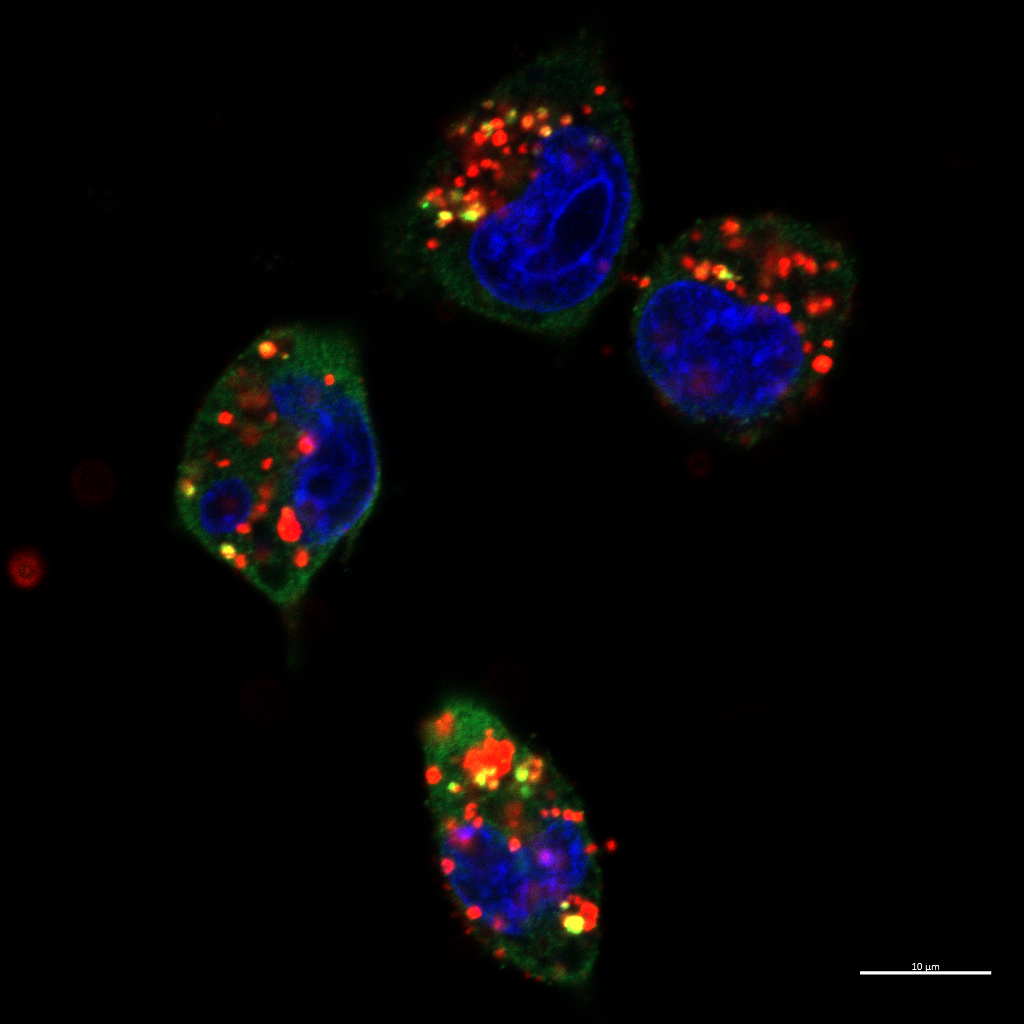

Supplement: Supplementary file 6 — Source data Fig. 5 [file 44318_2026_817_MOESM6_ESM.zip › 5C/5C-4-Washout 3 h-Smcr8 KO.tif]

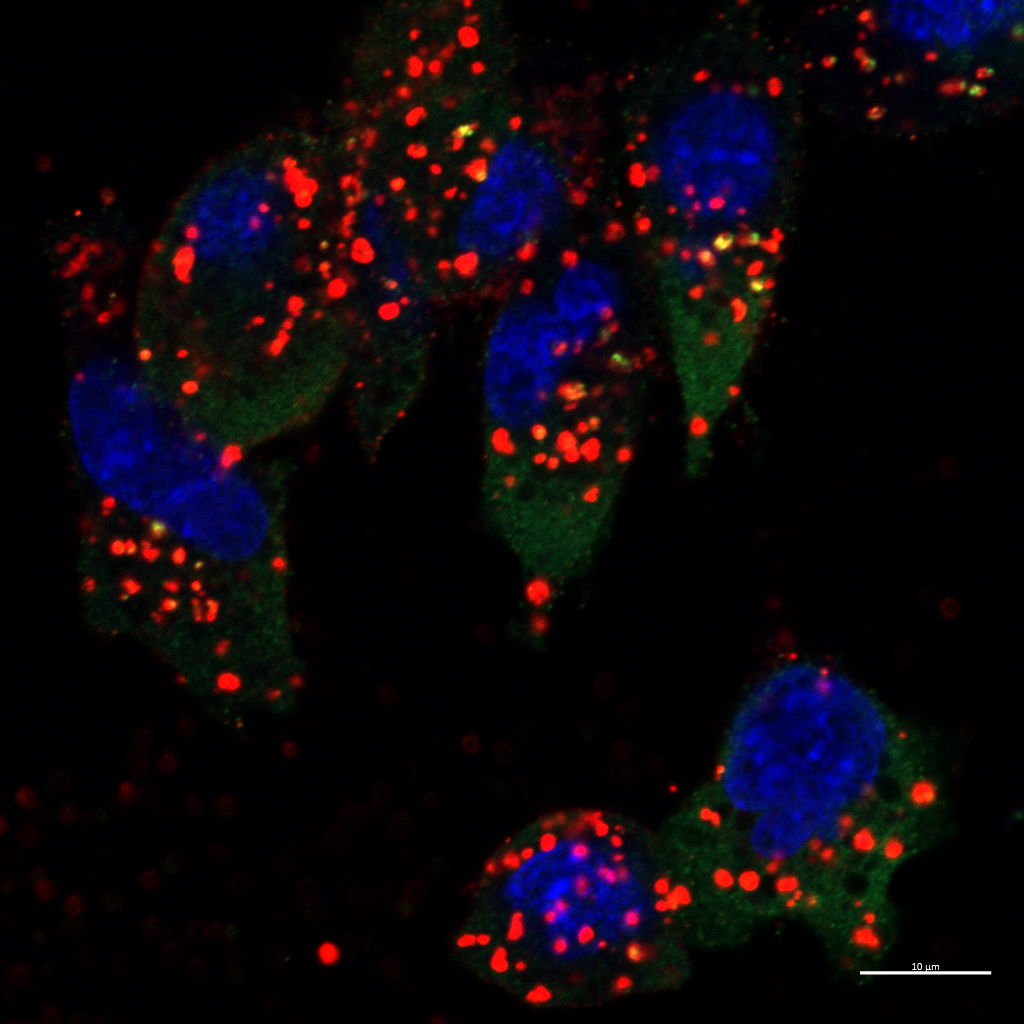

Supplement: Supplementary file 6 — Source data Fig. 5 [file 44318_2026_817_MOESM6_ESM.zip › 5C/5C-4-Washout 3 h-WT.tif]

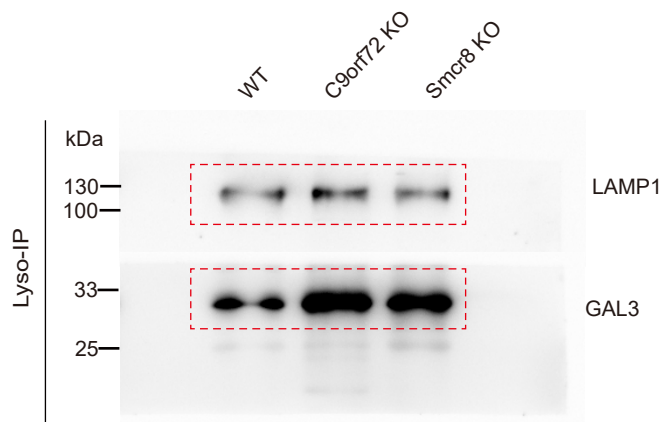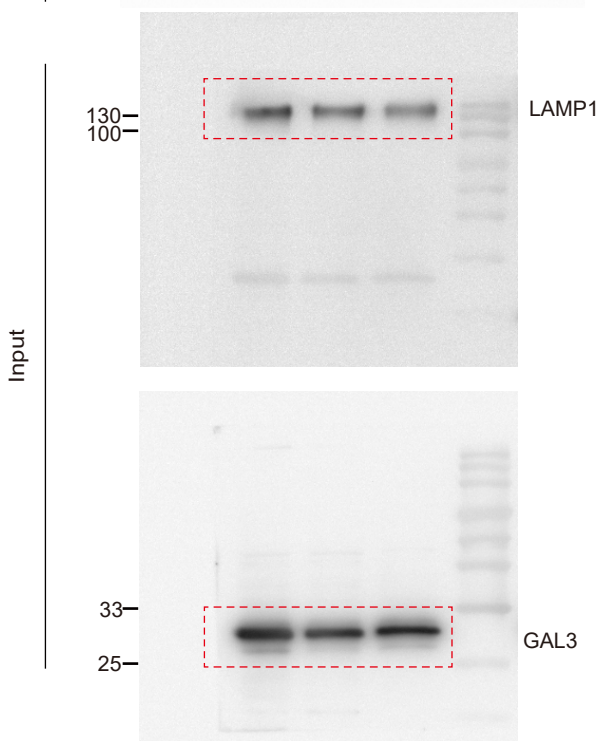

Supplement: Supplementary file 6 — Source data Fig. 5 [file 44318_2026_817_MOESM6_ESM.zip › 5E/5E.pdf]

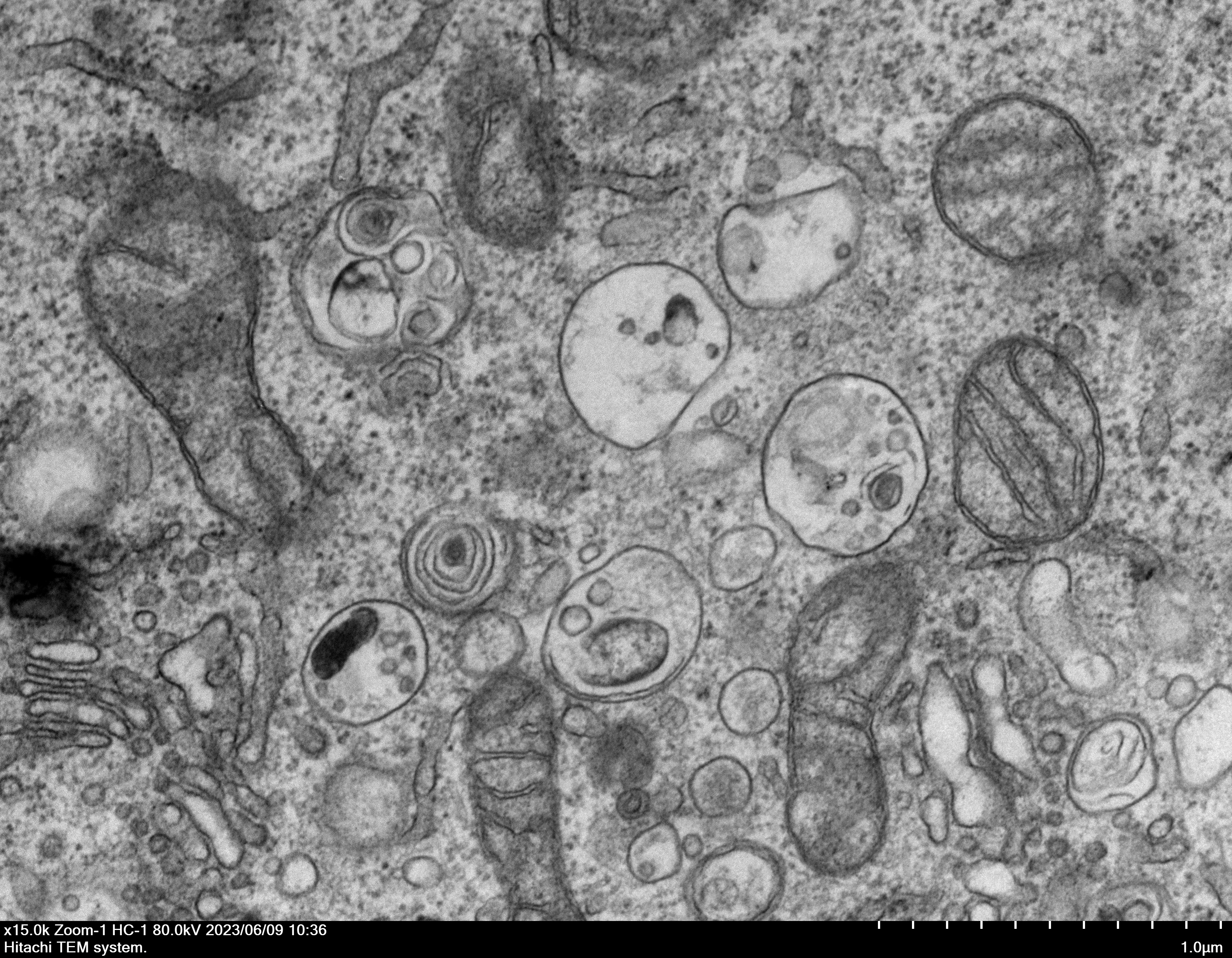

Supplement: Supplementary file 6 — Source data Fig. 5 [file 44318_2026_817_MOESM6_ESM.zip › 5F/5F-1-Basal-C9orf72 KO_TEM.tif]

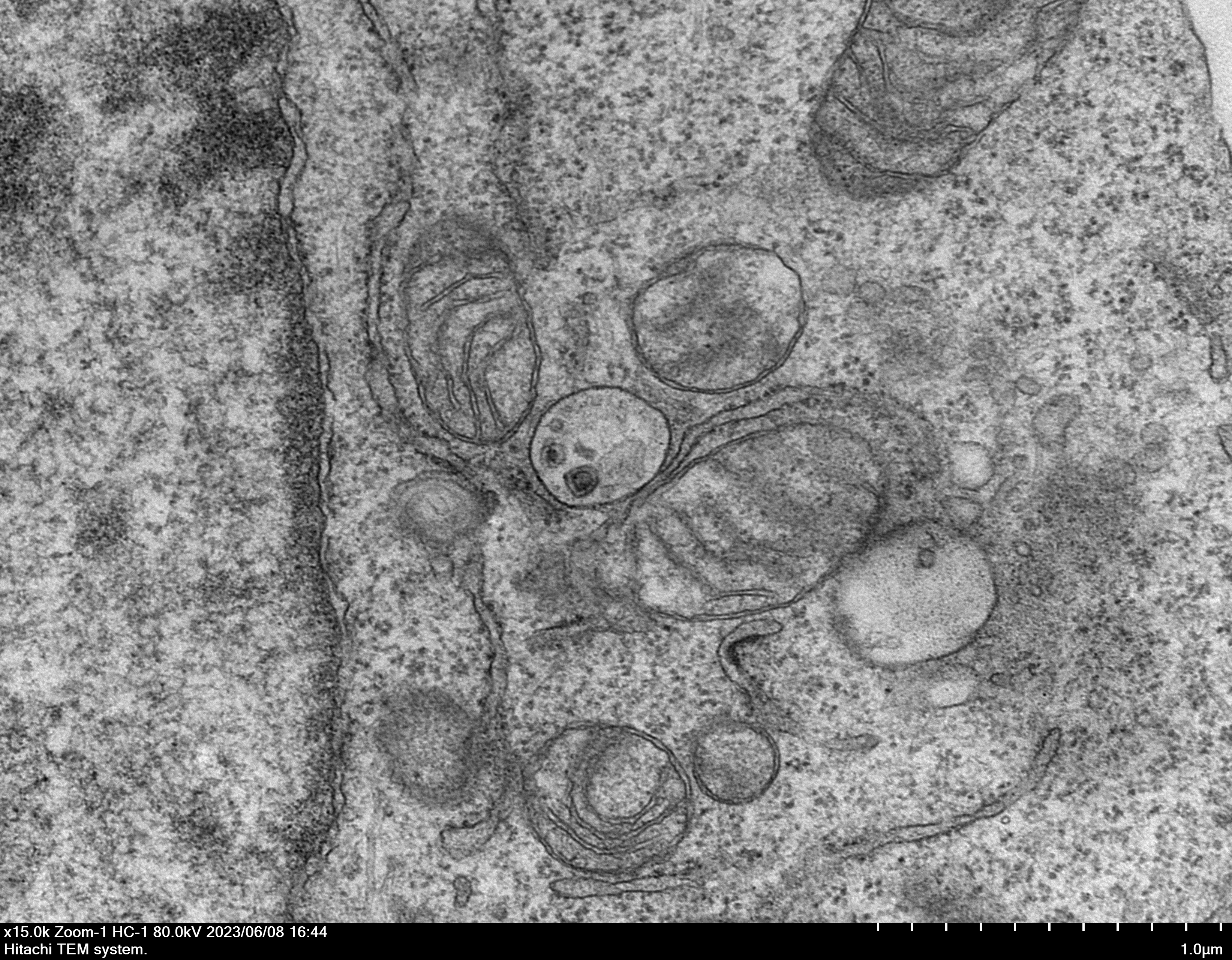

Supplement: Supplementary file 6 — Source data Fig. 5 [file 44318_2026_817_MOESM6_ESM.zip › 5F/5F-1-Basal-dKO_TEM.tif]

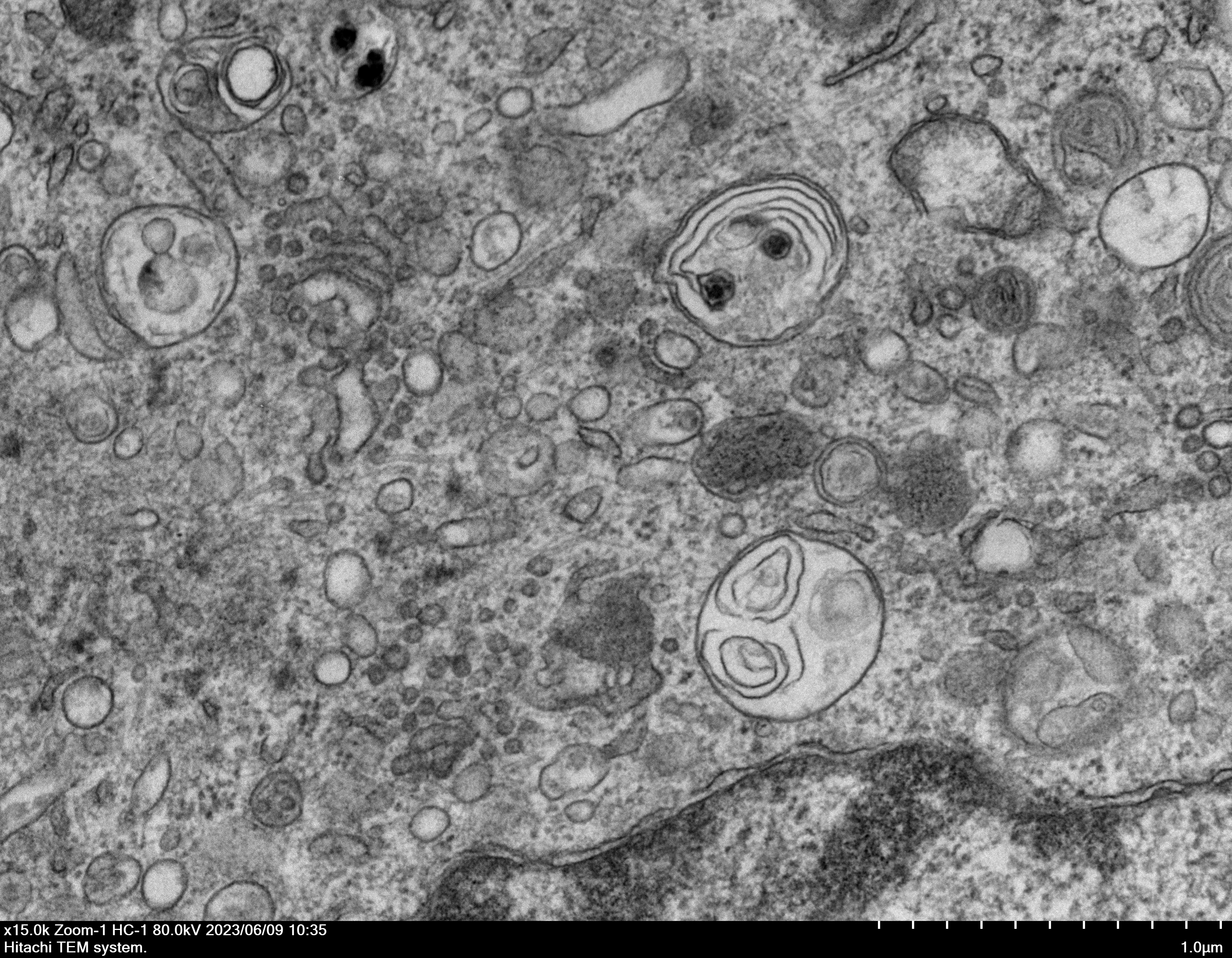

Supplement: Supplementary file 6 — Source data Fig. 5 [file 44318_2026_817_MOESM6_ESM.zip › 5F/5F-1-Basal-Smcr8 KO_TEM.tif]

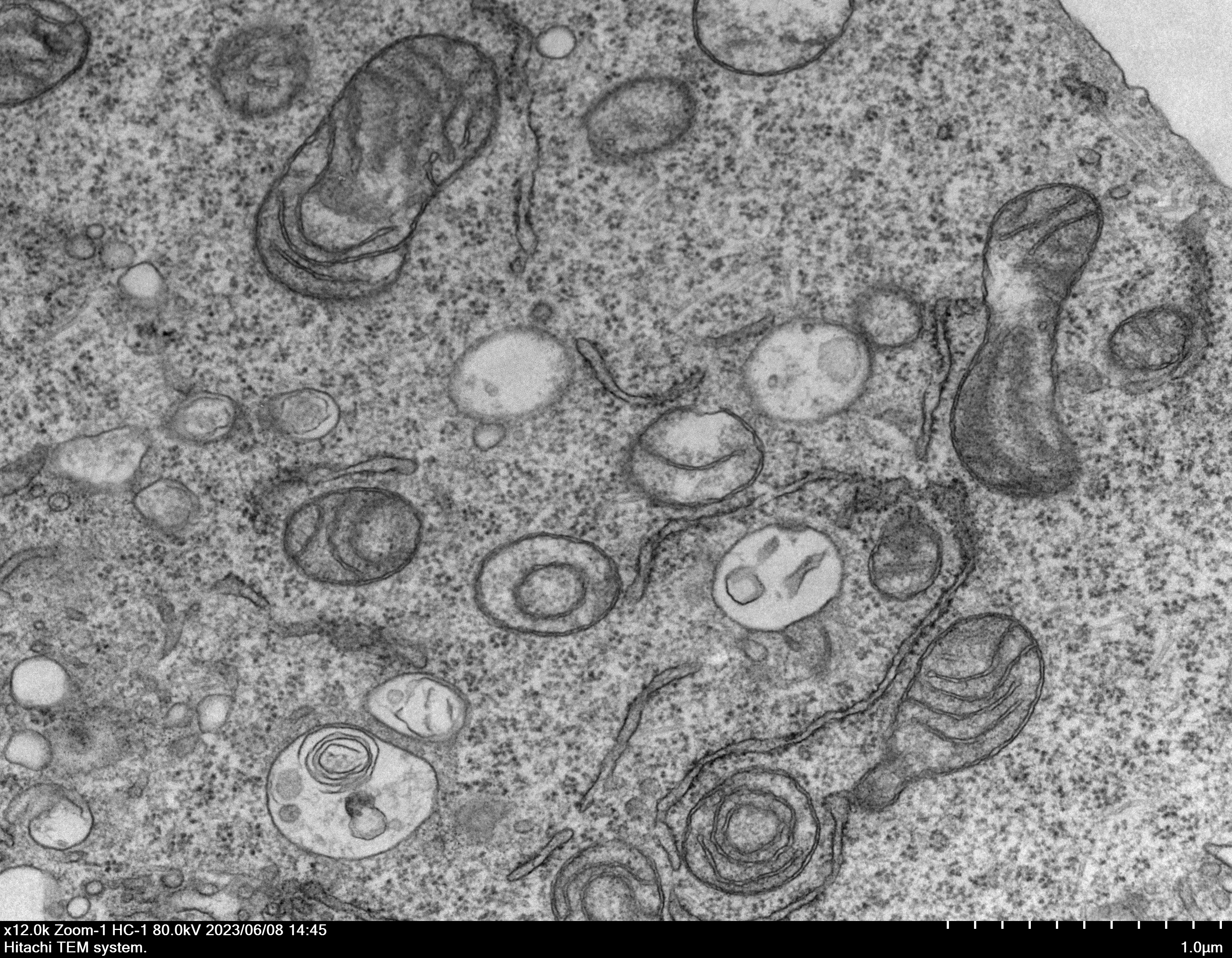

Supplement: Supplementary file 6 — Source data Fig. 5 [file 44318_2026_817_MOESM6_ESM.zip › 5F/5F-1-Basal-WT_TEM.tif]

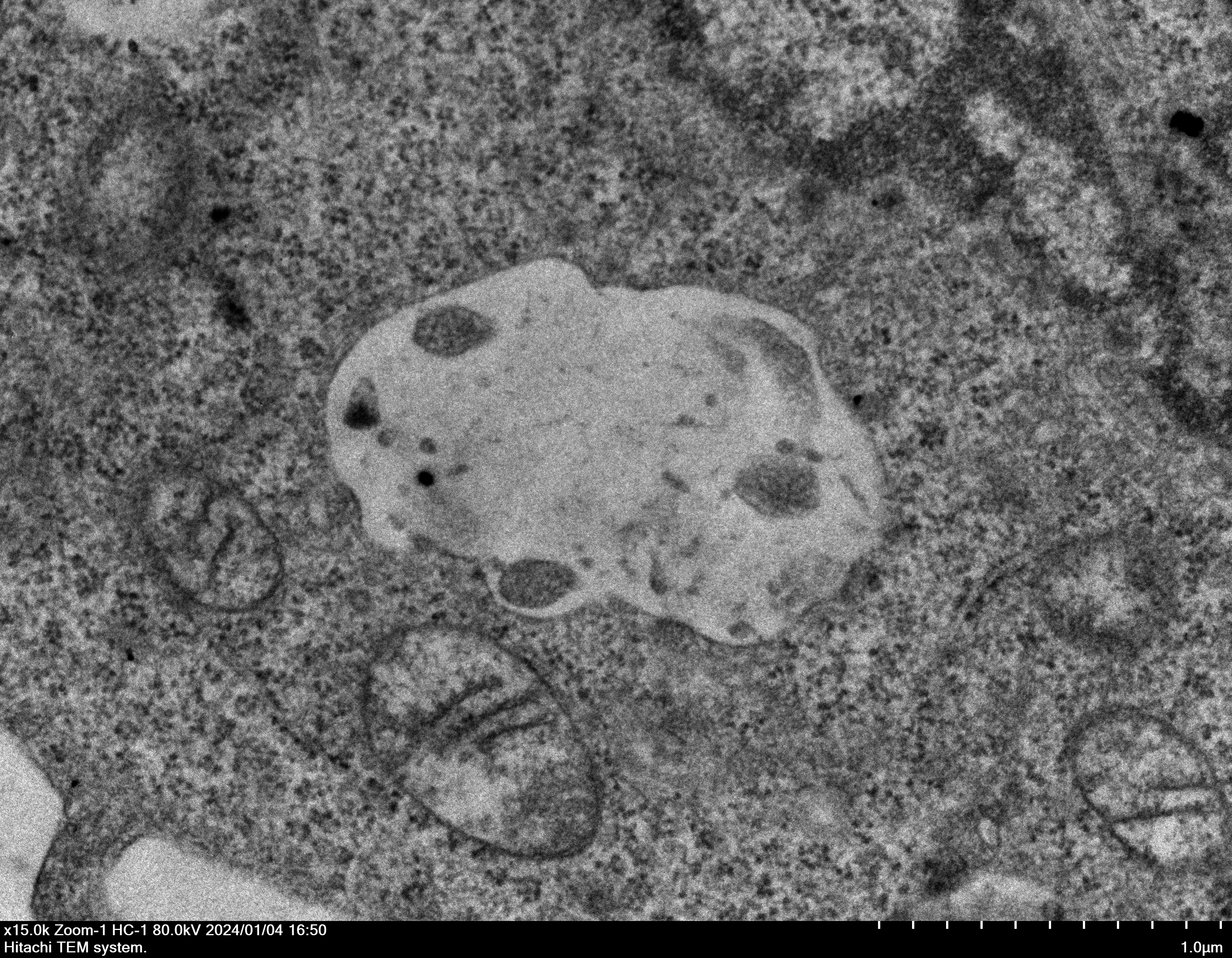

Supplement: Supplementary file 6 — Source data Fig. 5 [file 44318_2026_817_MOESM6_ESM.zip › 5F/5F-2-LLOMe-C9orf72 KO_TEM.tif]

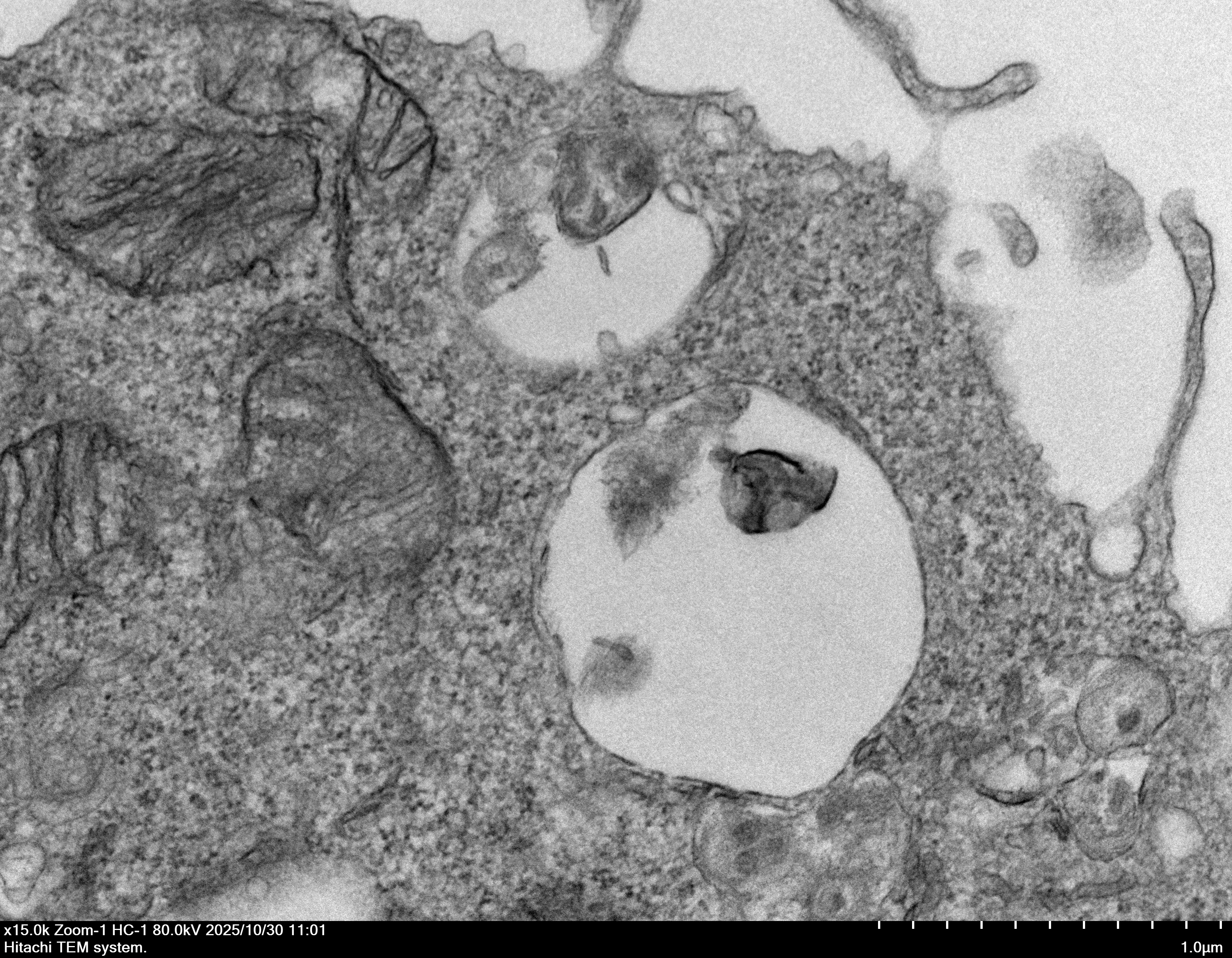

Supplement: Supplementary file 6 — Source data Fig. 5 [file 44318_2026_817_MOESM6_ESM.zip › 5F/5F-2-LLOMe-dKO_TEM.tif]

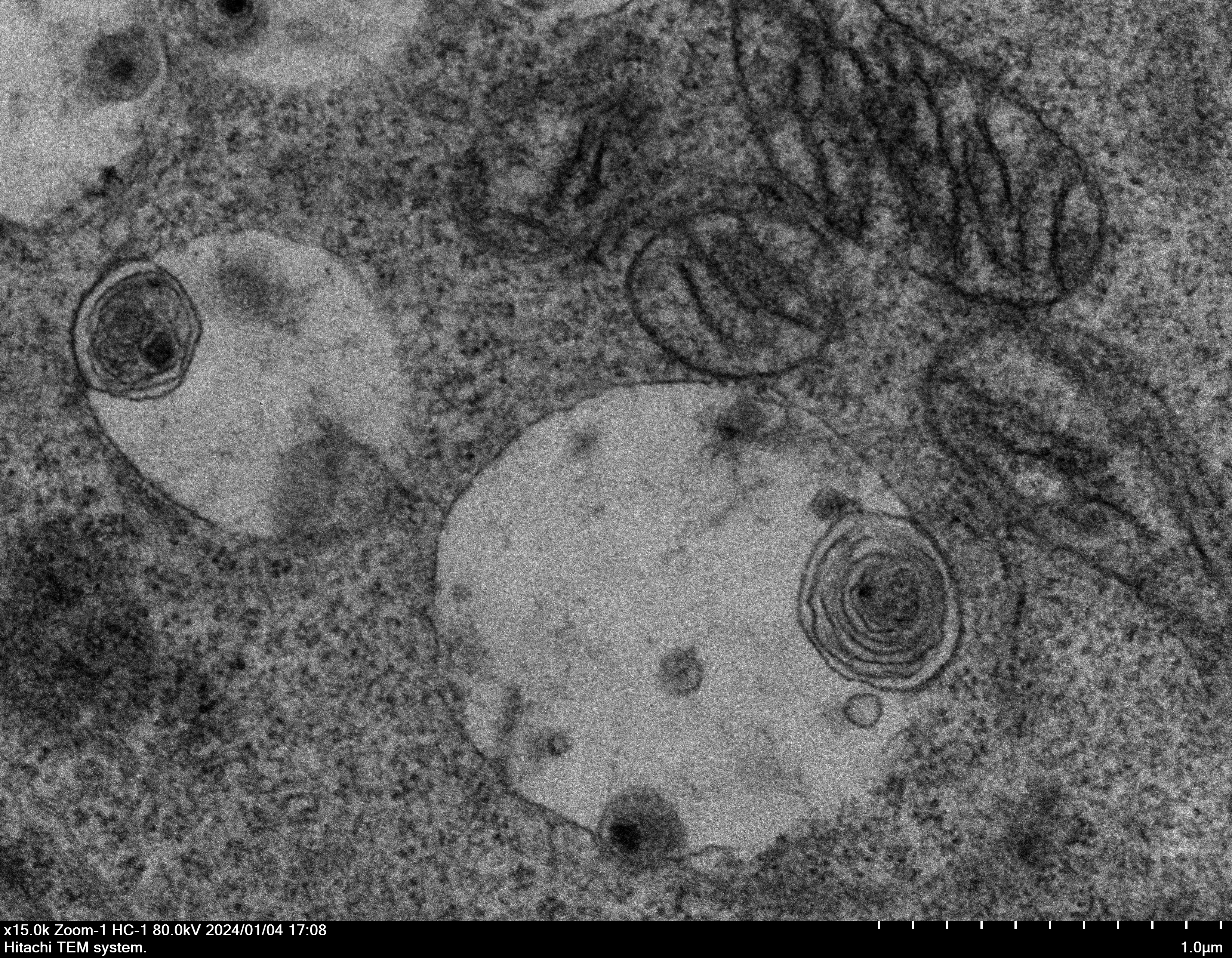

Supplement: Supplementary file 6 — Source data Fig. 5 [file 44318_2026_817_MOESM6_ESM.zip › 5F/5F-2-LLOMe-Smcr8 KO_TEM.tif]

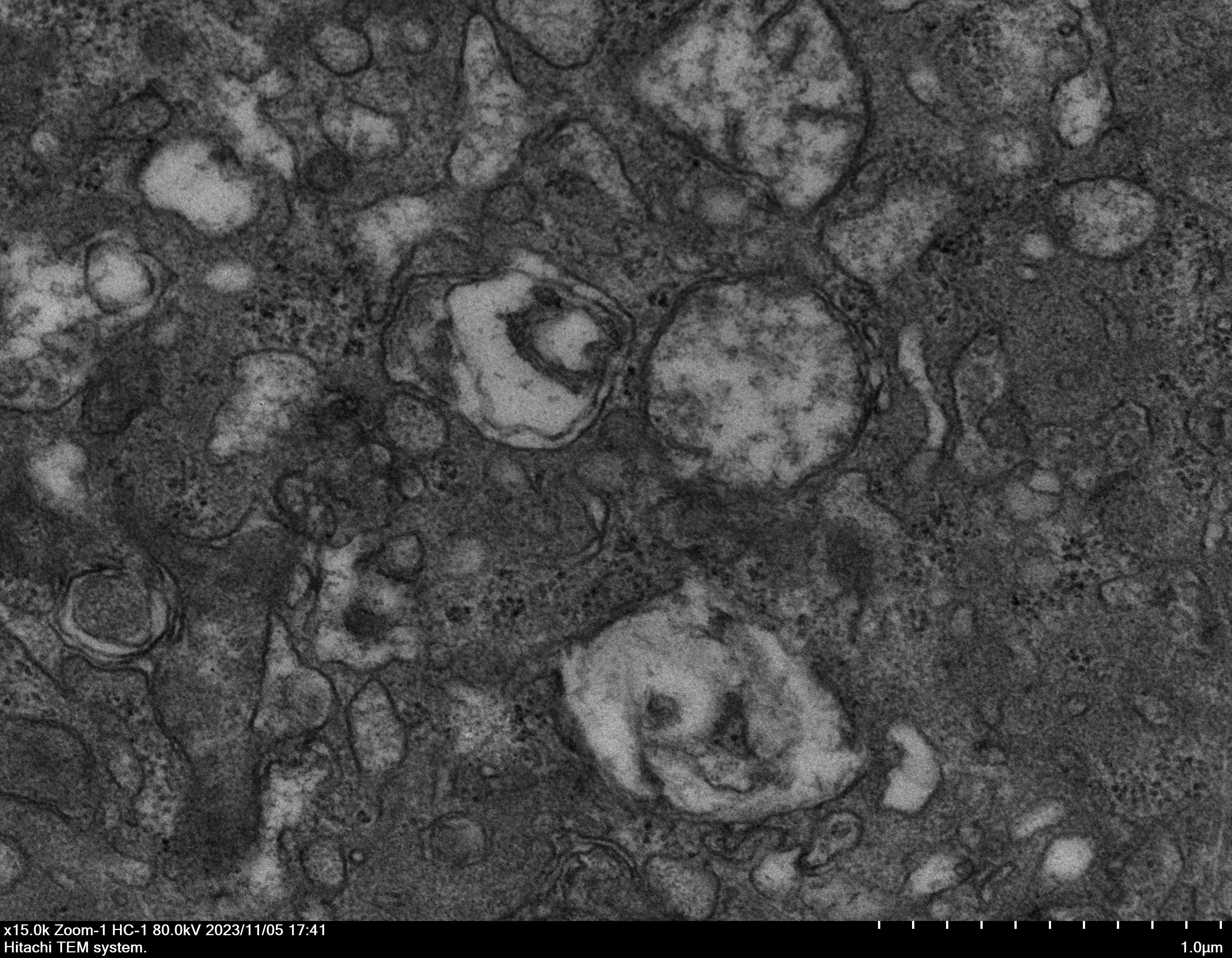

Supplement: Supplementary file 6 — Source data Fig. 5 [file 44318_2026_817_MOESM6_ESM.zip › 5F/5F-2-LLOMe-WT_TEM.tif]

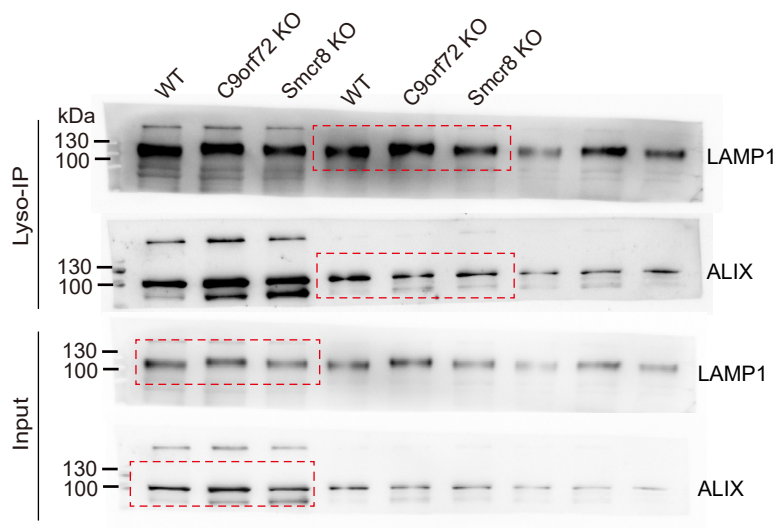

Supplement: Supplementary file 7 — Source data Fig. 6 [file 44318_2026_817_MOESM7_ESM.zip › 6A/6A.pdf]

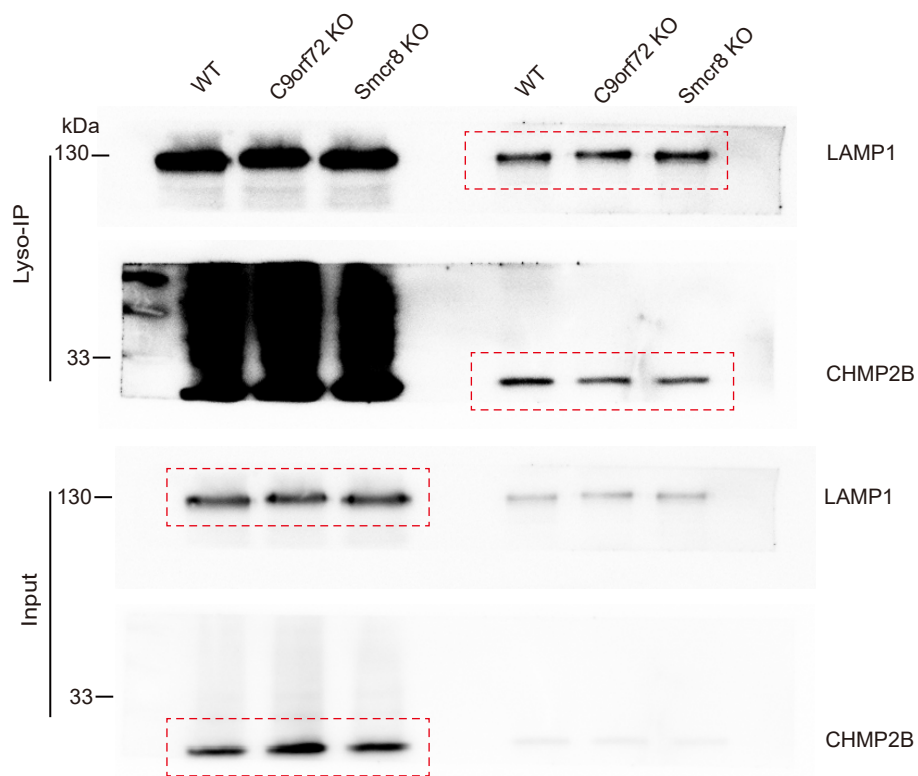

Supplement: Supplementary file 7 — Source data Fig. 6 [file 44318_2026_817_MOESM7_ESM.zip › 6B/6B.pdf]

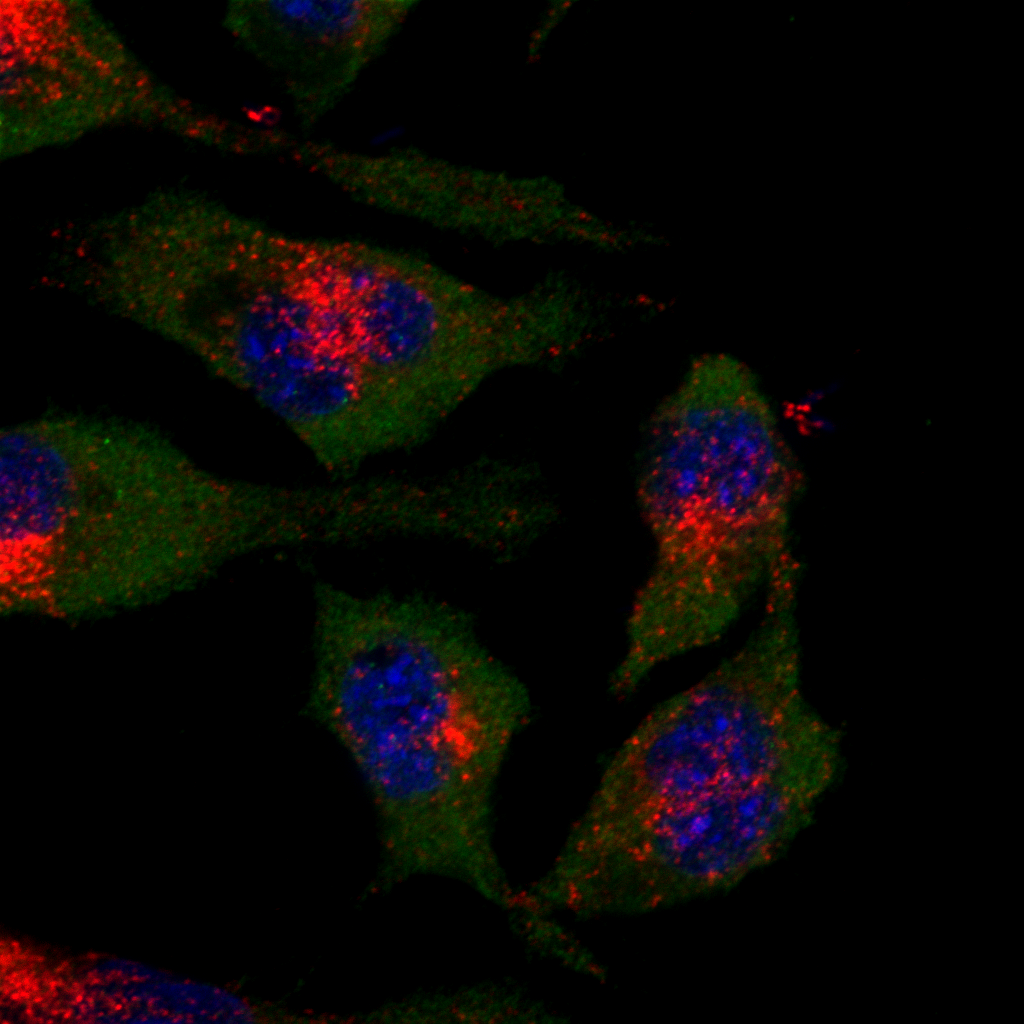

Supplement: Supplementary file 7 — Source data Fig. 6 [file 44318_2026_817_MOESM7_ESM.zip › 6C/6C-1-Basal-C9orf72 KO.tif]

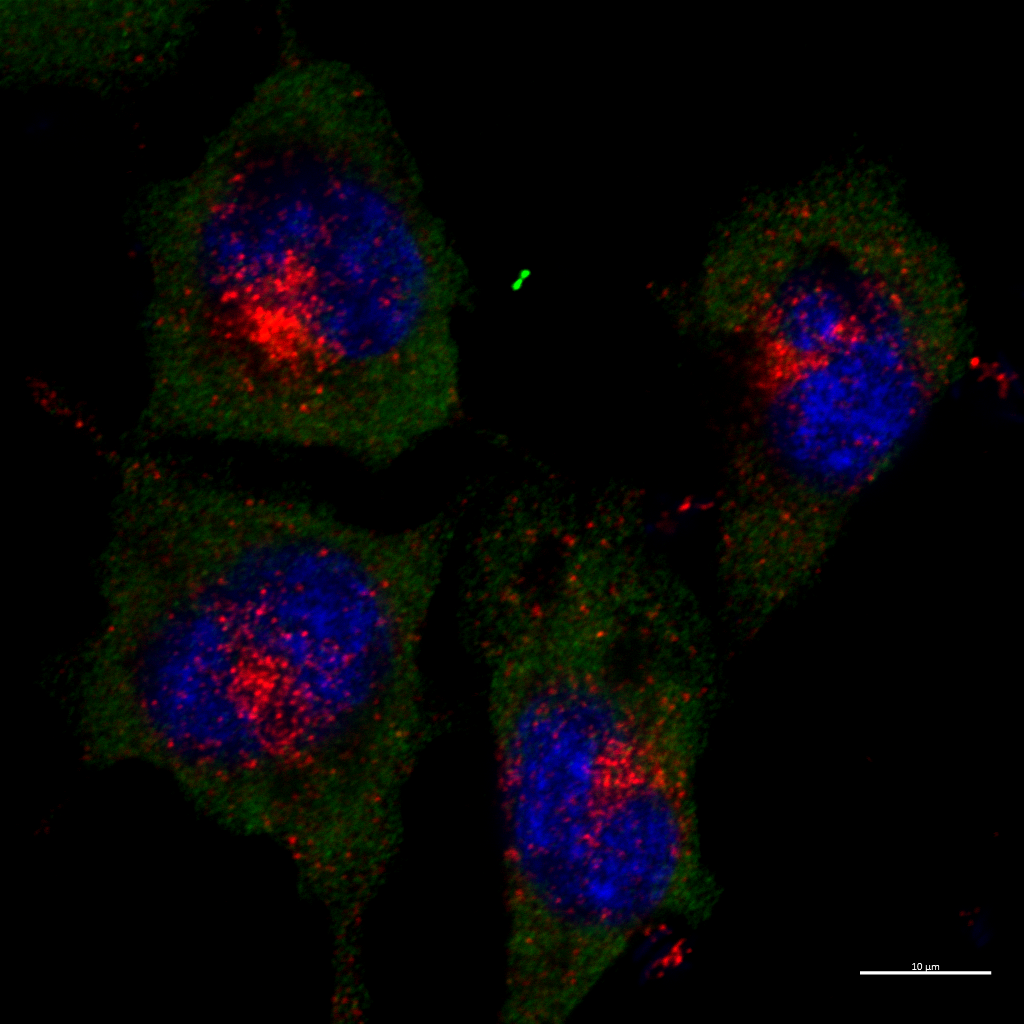

Supplement: Supplementary file 7 — Source data Fig. 6 [file 44318_2026_817_MOESM7_ESM.zip › 6C/6C-1-Basal-dKO.tif]

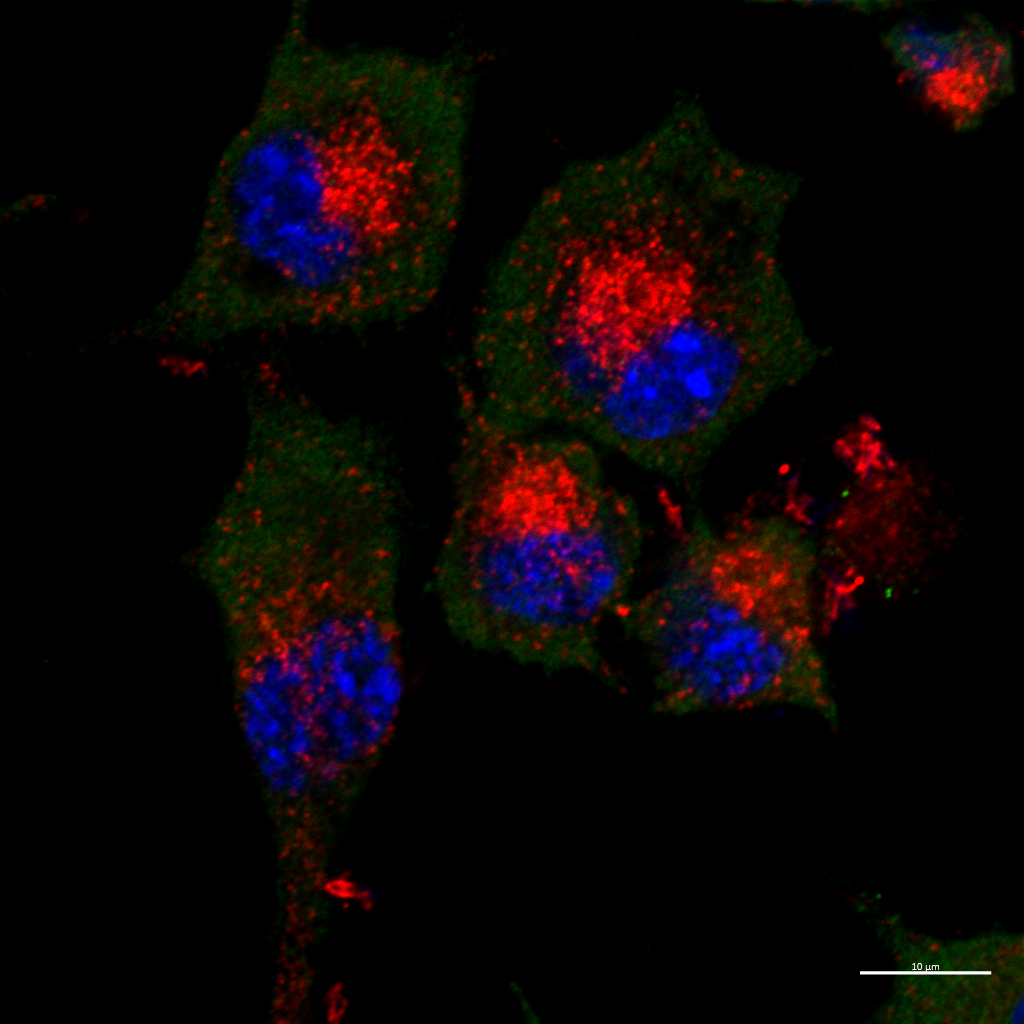

Supplement: Supplementary file 7 — Source data Fig. 6 [file 44318_2026_817_MOESM7_ESM.zip › 6C/6C-1-Basal-Smcr8 KO.tif]

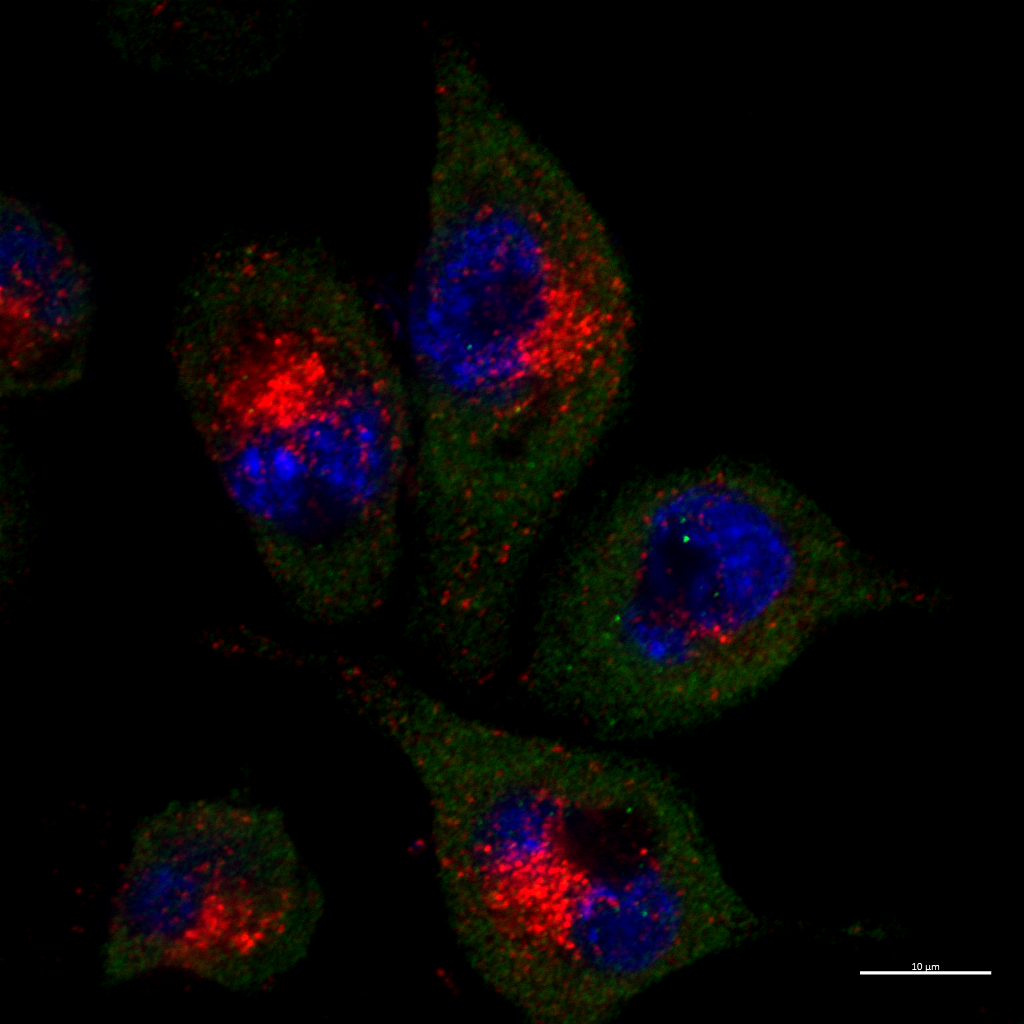

Supplement: Supplementary file 7 — Source data Fig. 6 [file 44318_2026_817_MOESM7_ESM.zip › 6C/6C-1-Basal-WT.tif]

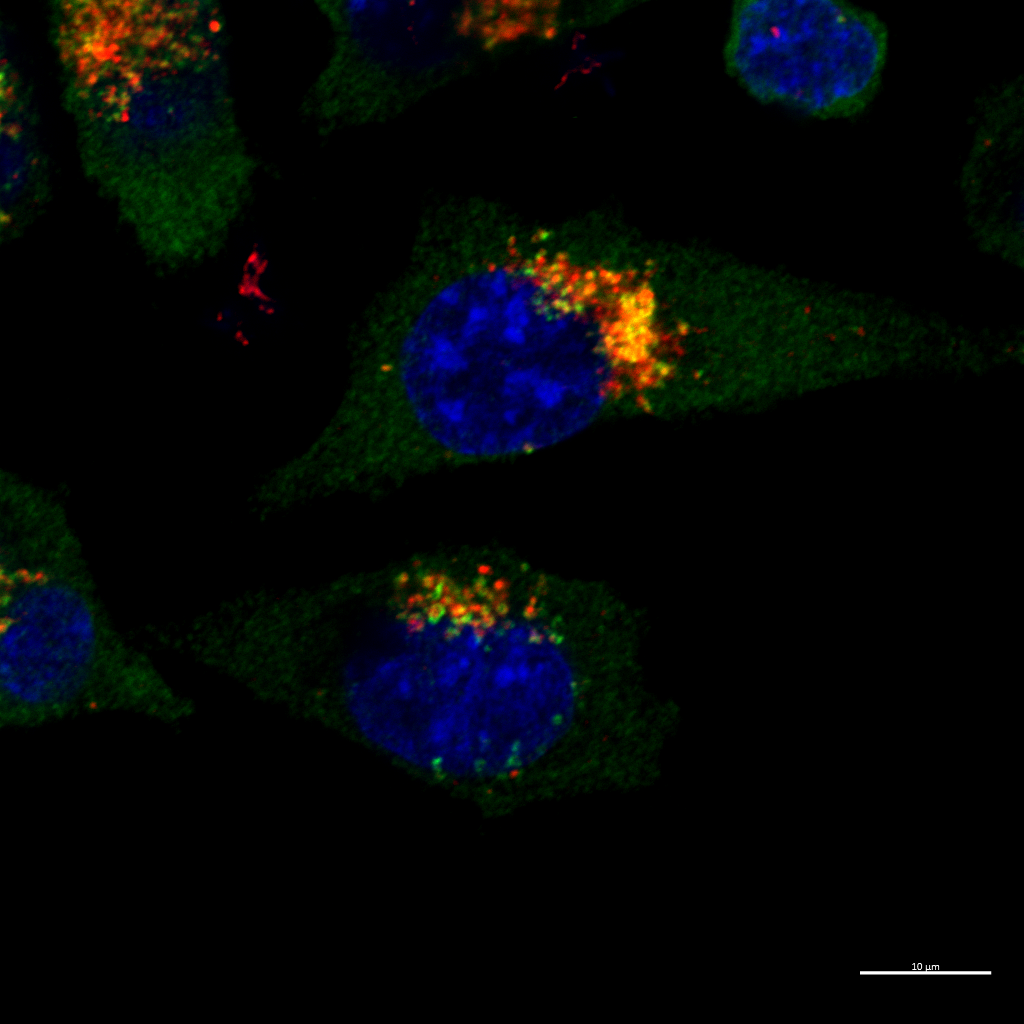

Supplement: Supplementary file 7 — Source data Fig. 6 [file 44318_2026_817_MOESM7_ESM.zip › 6C/6C-2-LLOMe 10 min-C9orf72 KO.tif]

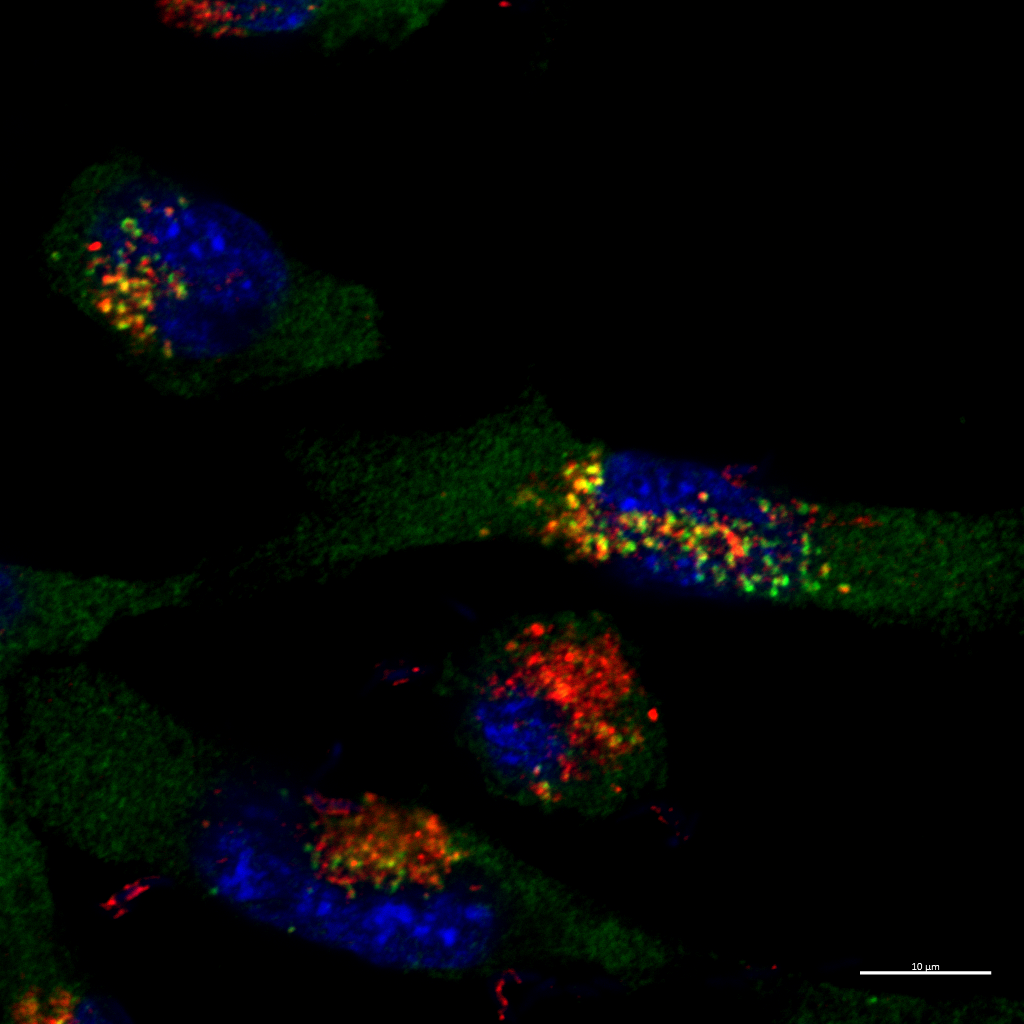

Supplement: Supplementary file 7 — Source data Fig. 6 [file 44318_2026_817_MOESM7_ESM.zip › 6C/6C-2-LLOMe 10 min-dKO.tif]

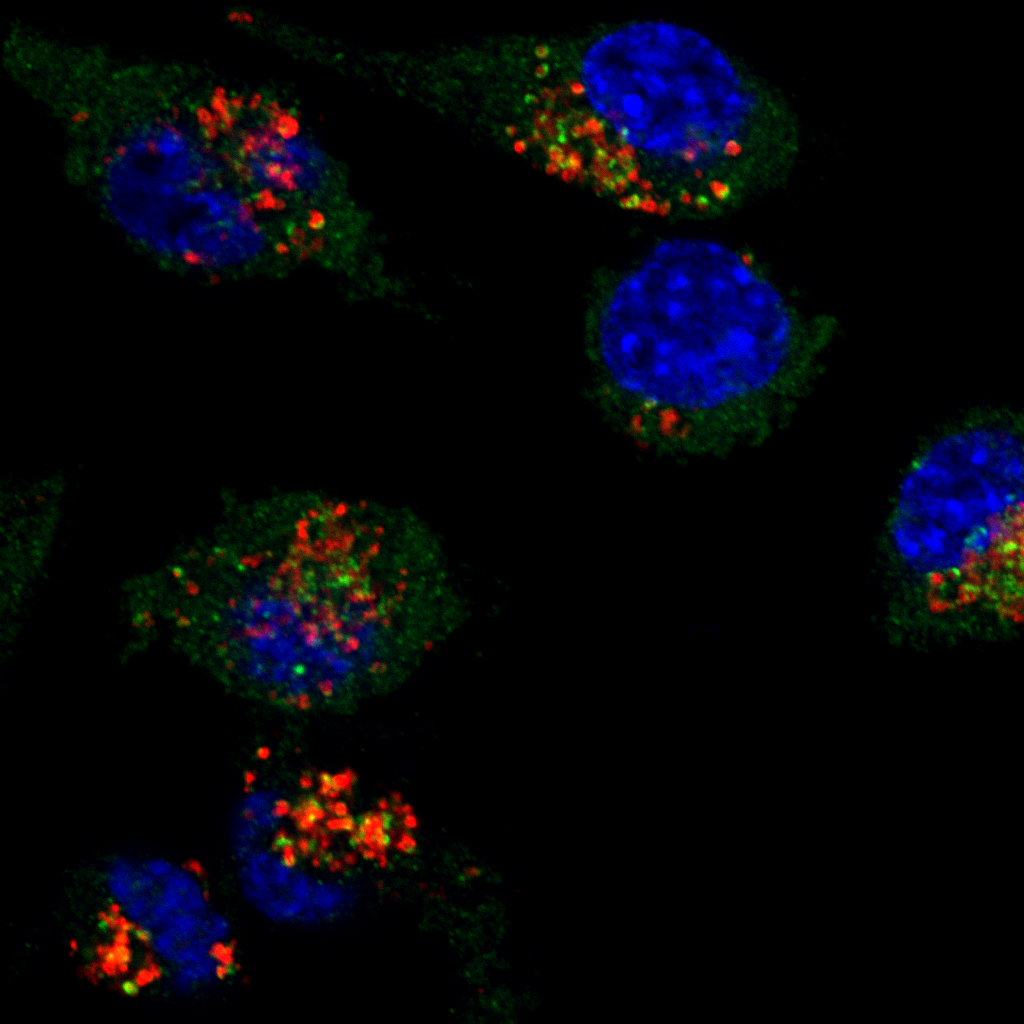

Supplement: Supplementary file 7 — Source data Fig. 6 [file 44318_2026_817_MOESM7_ESM.zip › 6C/6C-2-LLOMe 10 min-Smcr8 KO.tif]

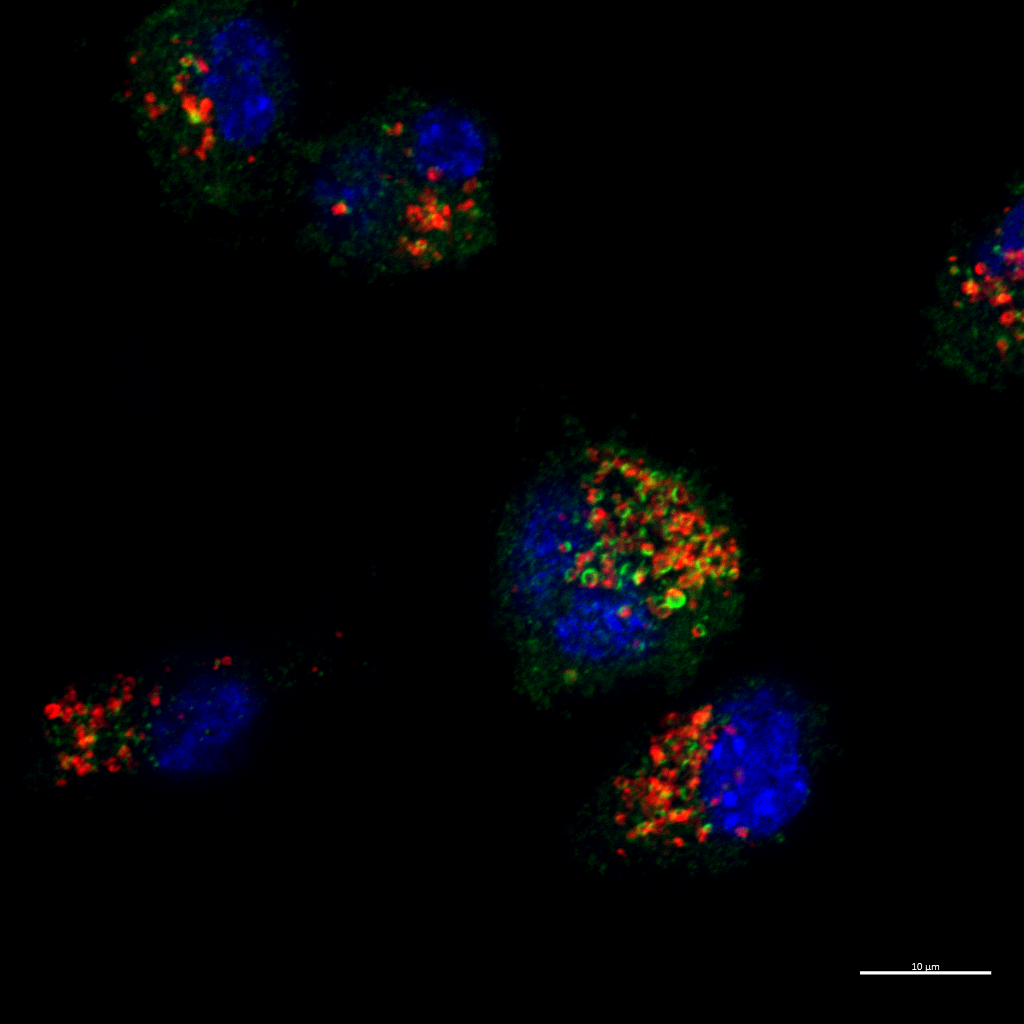

Supplement: Supplementary file 7 — Source data Fig. 6 [file 44318_2026_817_MOESM7_ESM.zip › 6C/6C-2-LLOMe 10 min-WT.tif]

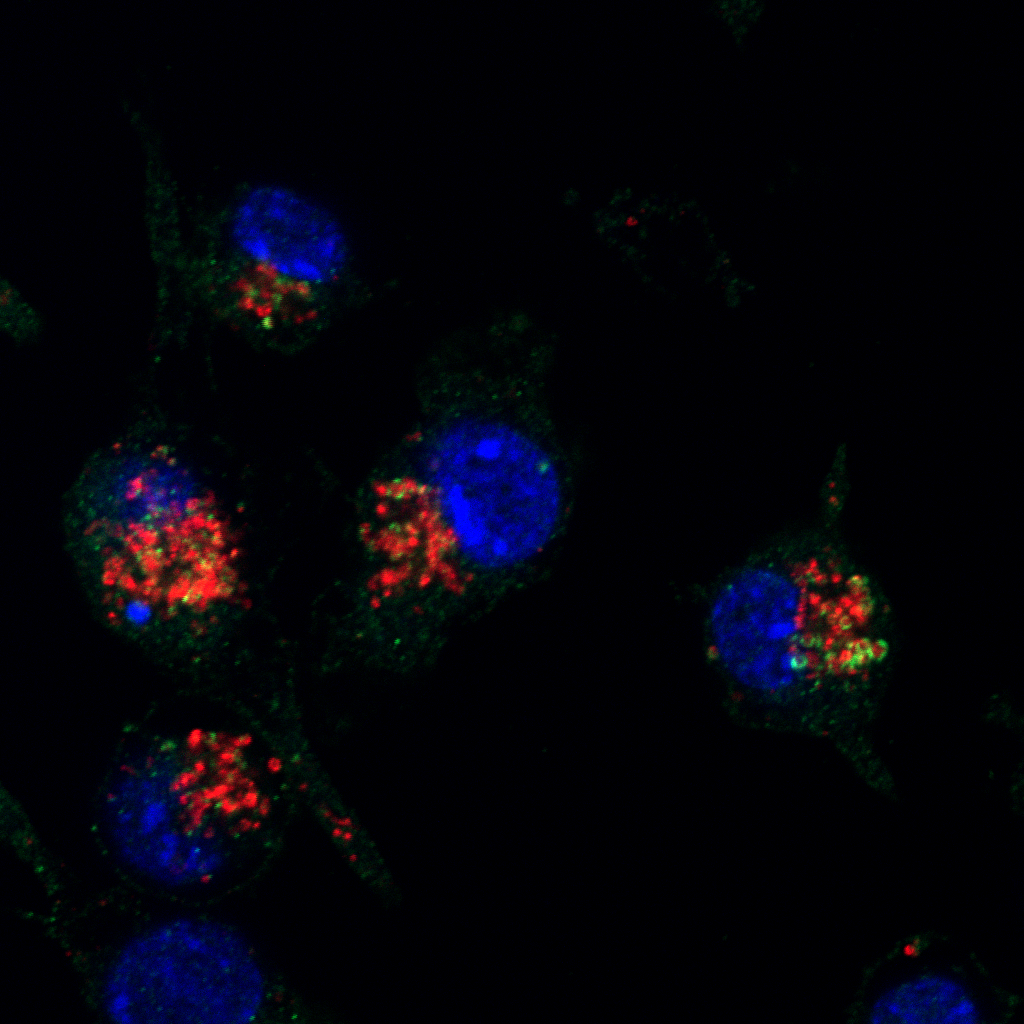

Supplement: Supplementary file 7 — Source data Fig. 6 [file 44318_2026_817_MOESM7_ESM.zip › 6C/6C-3-LLOMe 30 min-C9orf72 KO.tif]

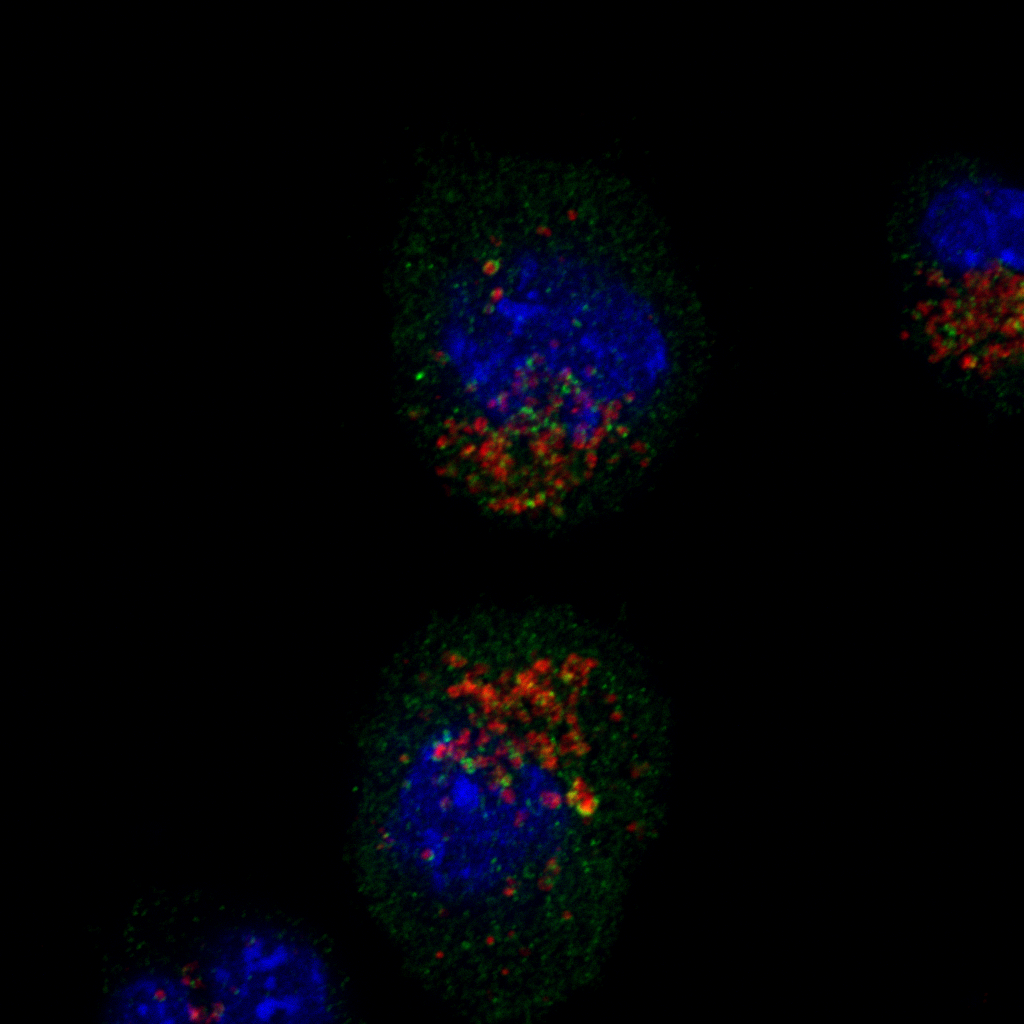

Supplement: Supplementary file 7 — Source data Fig. 6 [file 44318_2026_817_MOESM7_ESM.zip › 6C/6C-3-LLOMe 30 min-dKO.tif]

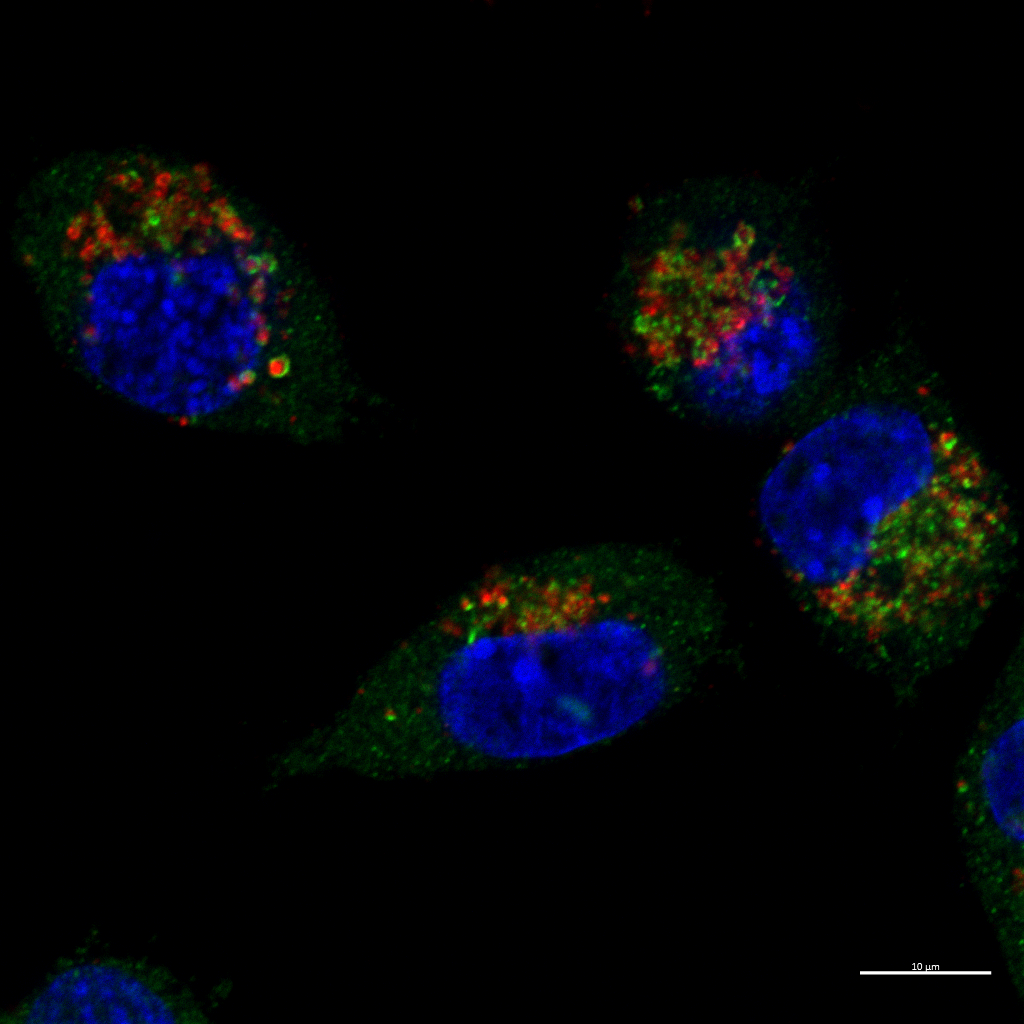

Supplement: Supplementary file 7 — Source data Fig. 6 [file 44318_2026_817_MOESM7_ESM.zip › 6C/6C-3-LLOMe 30 min-Smcr8 KO.tif]

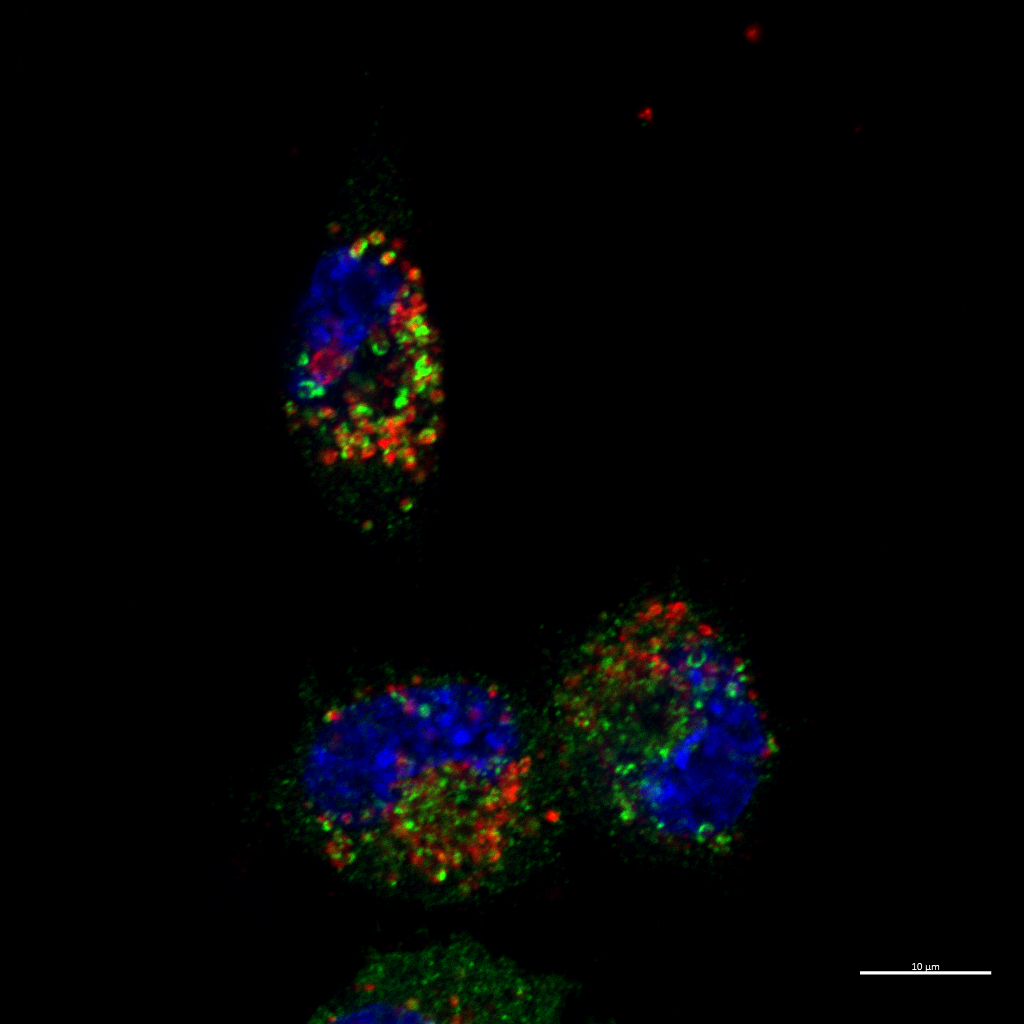

Supplement: Supplementary file 7 — Source data Fig. 6 [file 44318_2026_817_MOESM7_ESM.zip › 6C/6C-3-LLOMe 30 min-WT.tif]

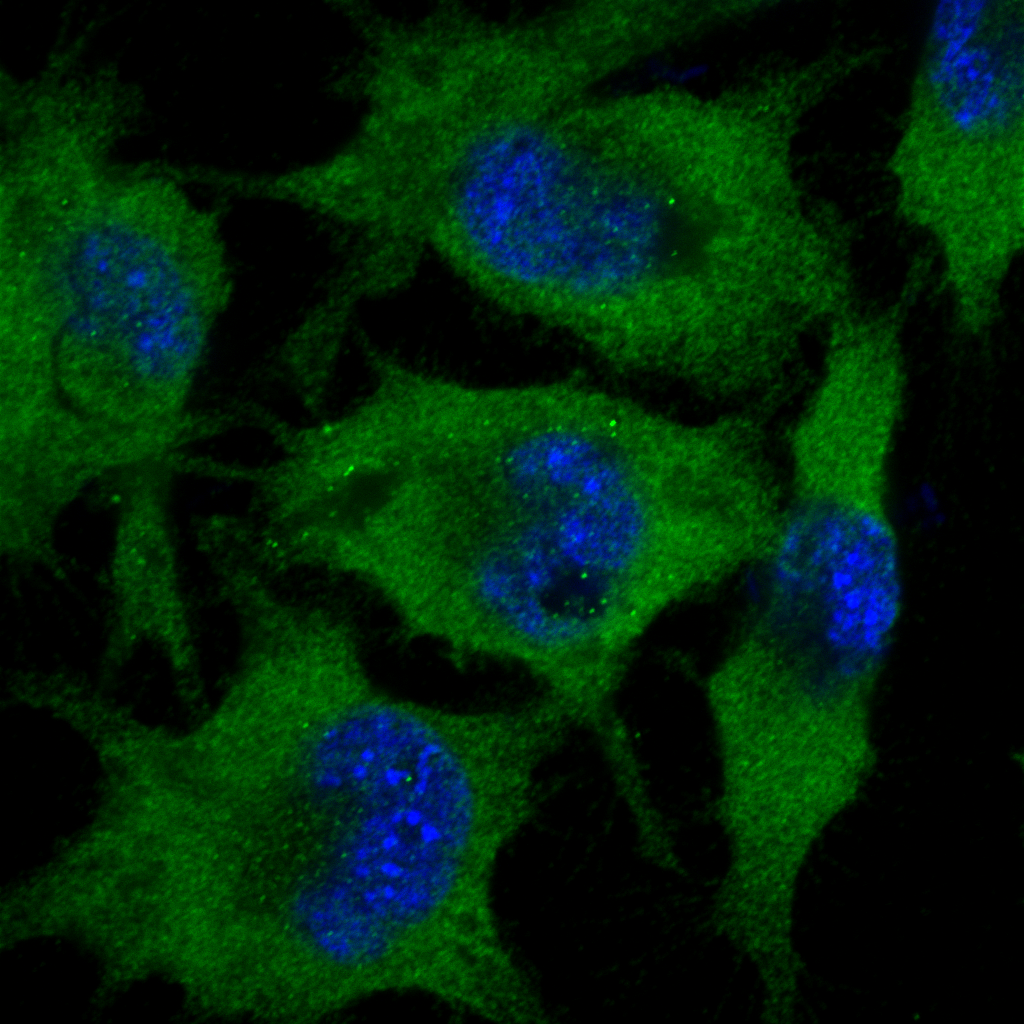

Supplement: Supplementary file 7 — Source data Fig. 6 [file 44318_2026_817_MOESM7_ESM.zip › 6E/6E-1-Basal-C9orf72 KO.tif]

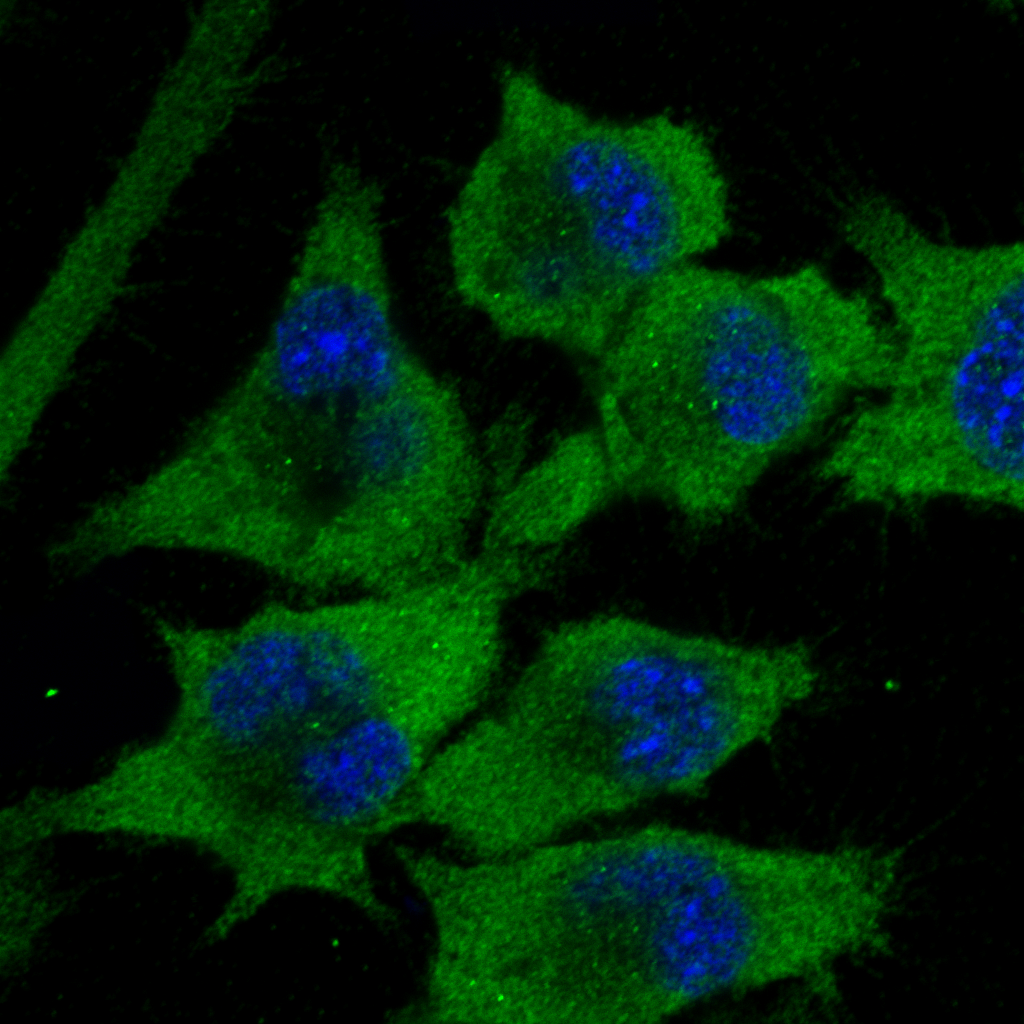

Supplement: Supplementary file 7 — Source data Fig. 6 [file 44318_2026_817_MOESM7_ESM.zip › 6E/6E-1-Basal-dKO.tif]

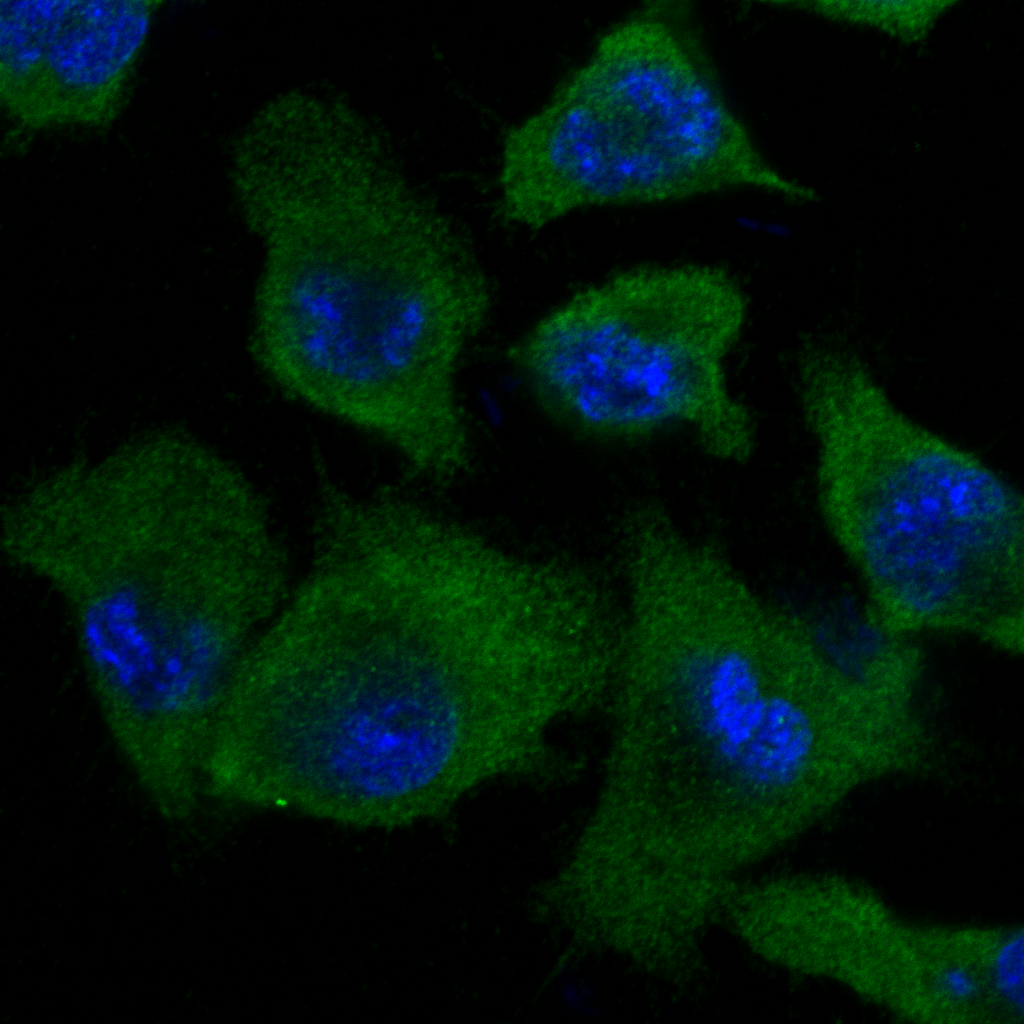

Supplement: Supplementary file 7 — Source data Fig. 6 [file 44318_2026_817_MOESM7_ESM.zip › 6E/6E-1-Basal-Smcr8 KO.tif]

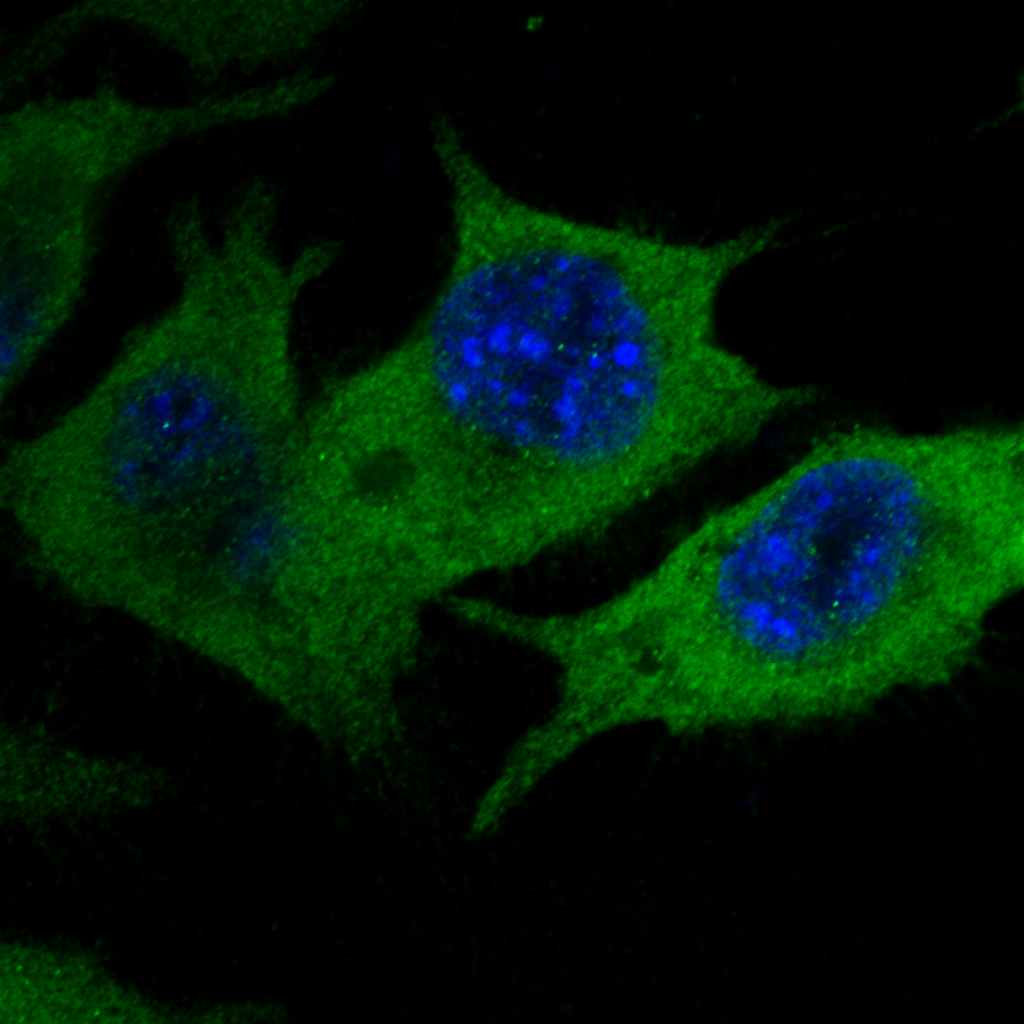

Supplement: Supplementary file 7 — Source data Fig. 6 [file 44318_2026_817_MOESM7_ESM.zip › 6E/6E-1-Basal-WT.tif]

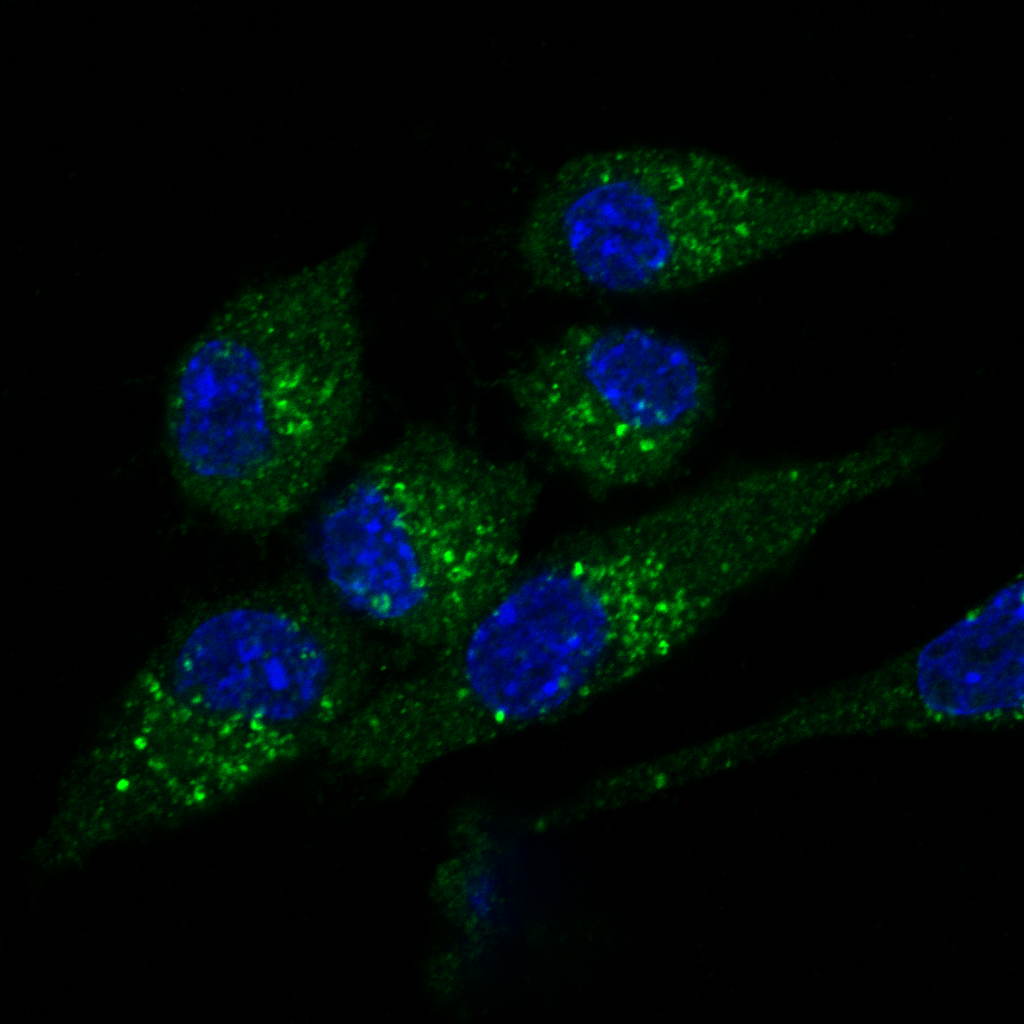

Supplement: Supplementary file 7 — Source data Fig. 6 [file 44318_2026_817_MOESM7_ESM.zip › 6E/6E-2-LLOMe 10 min-C9orf72 KO.tif]

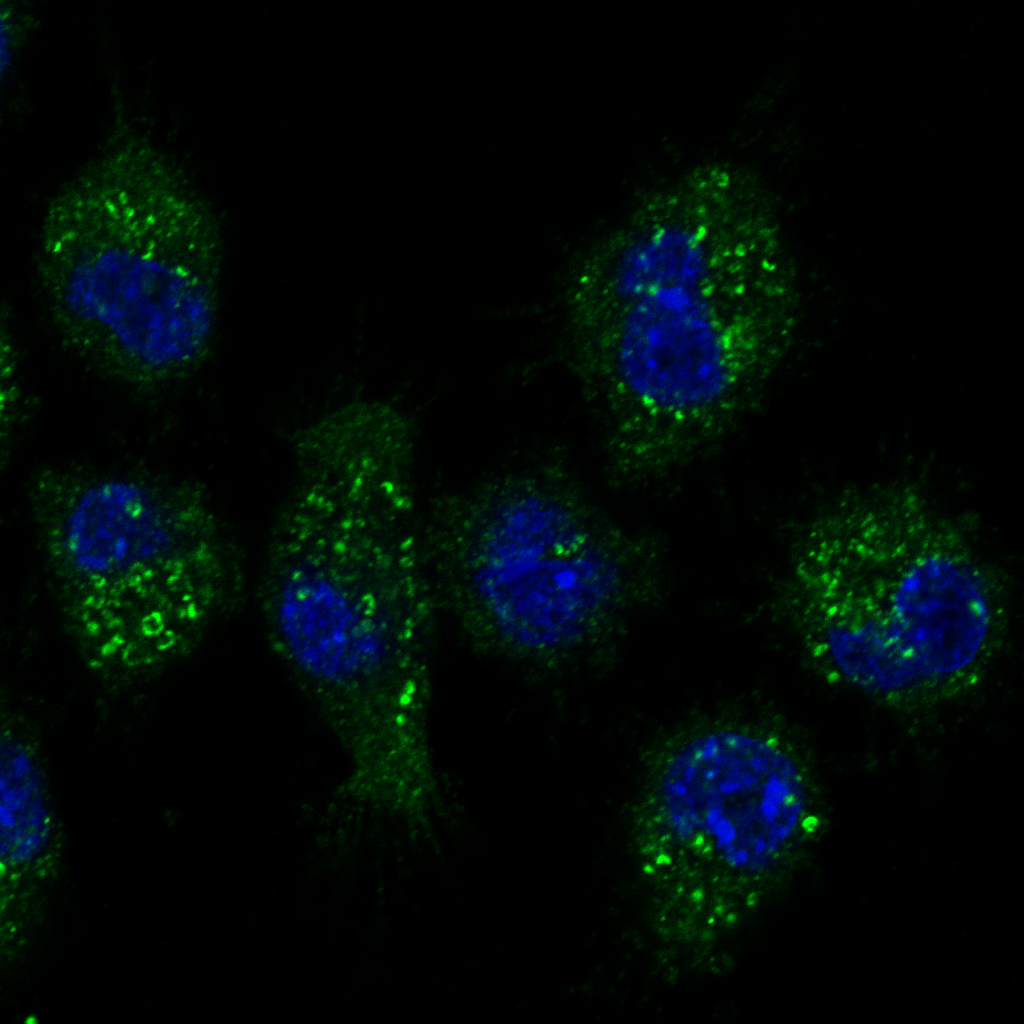

Supplement: Supplementary file 7 — Source data Fig. 6 [file 44318_2026_817_MOESM7_ESM.zip › 6E/6E-2-LLOMe 10 min-dKO.tif]
