# Supplementary figures and images for: The C9orf72/SMCR8 complex maintains microglial homeostasis via RAB8A-ESCRT-mediated lysosomal repair (part 2 of 3)
Source: EMBO J. 2026 May 29;45(13):4531–68. doi: 10.1038/s44318-026-00817-w (PMC13324726; doi:10.1038/s44318-026-00817-w)

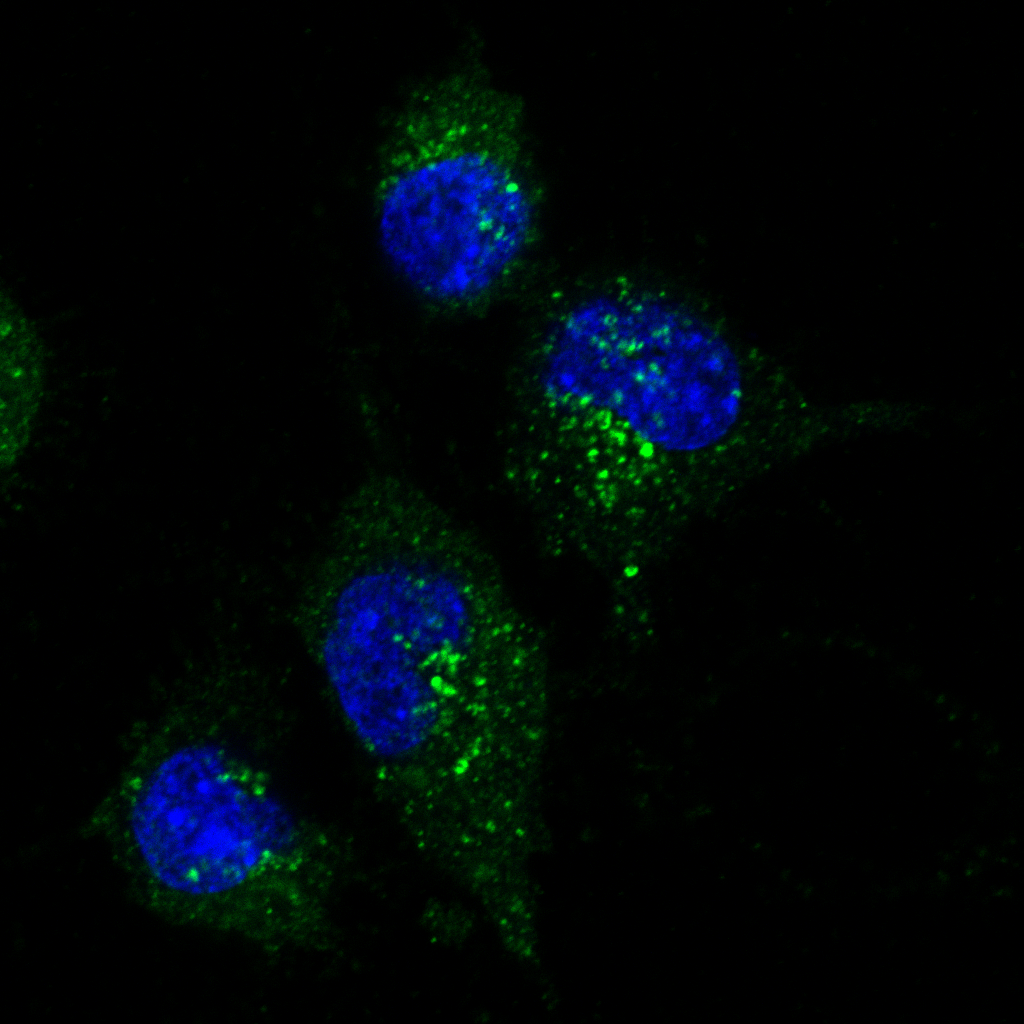

Supplement: Supplementary file 7 — Source data Fig. 6 [file 44318_2026_817_MOESM7_ESM.zip › 6E/6E-2-LLOMe 10 min-Smcr8 KO.tif]

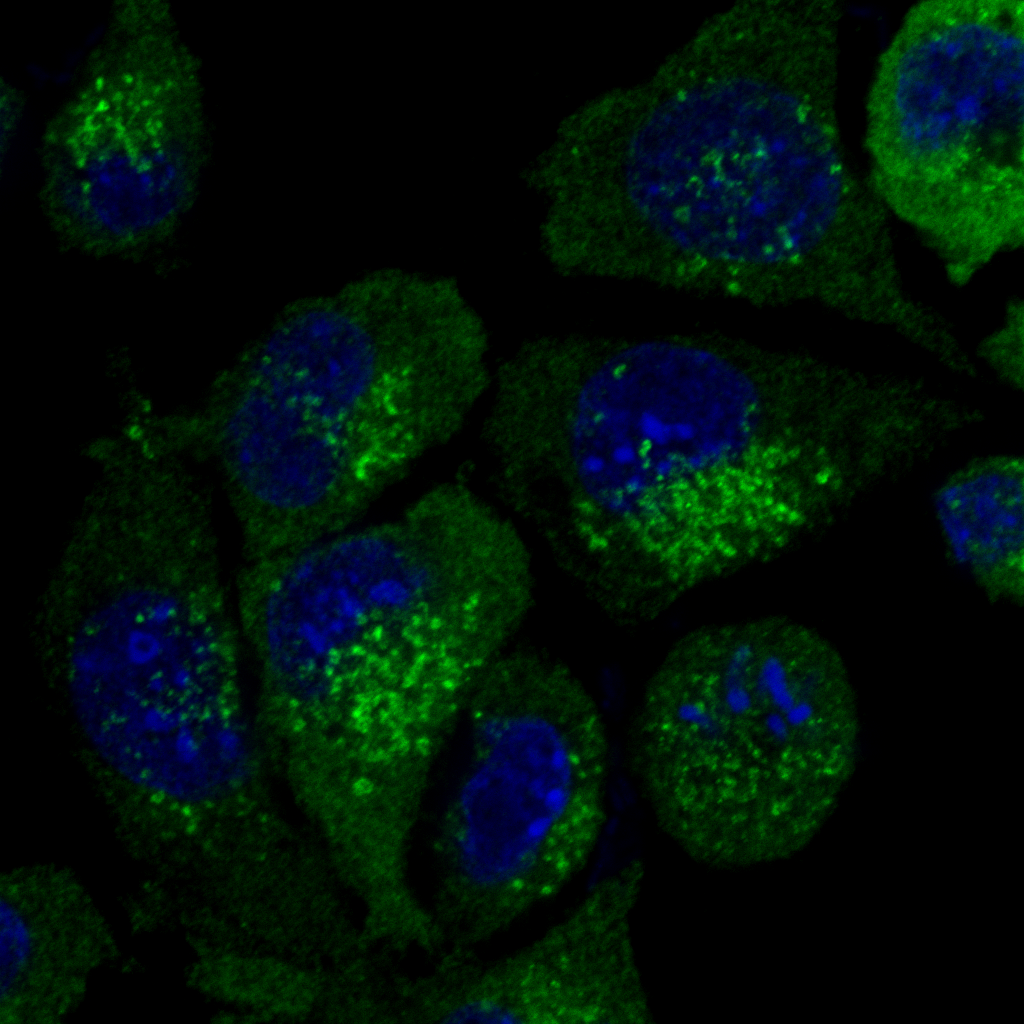

Supplement: Supplementary file 7 — Source data Fig. 6 [file 44318_2026_817_MOESM7_ESM.zip › 6E/6E-2-LLOMe 10 min-WT.tif]

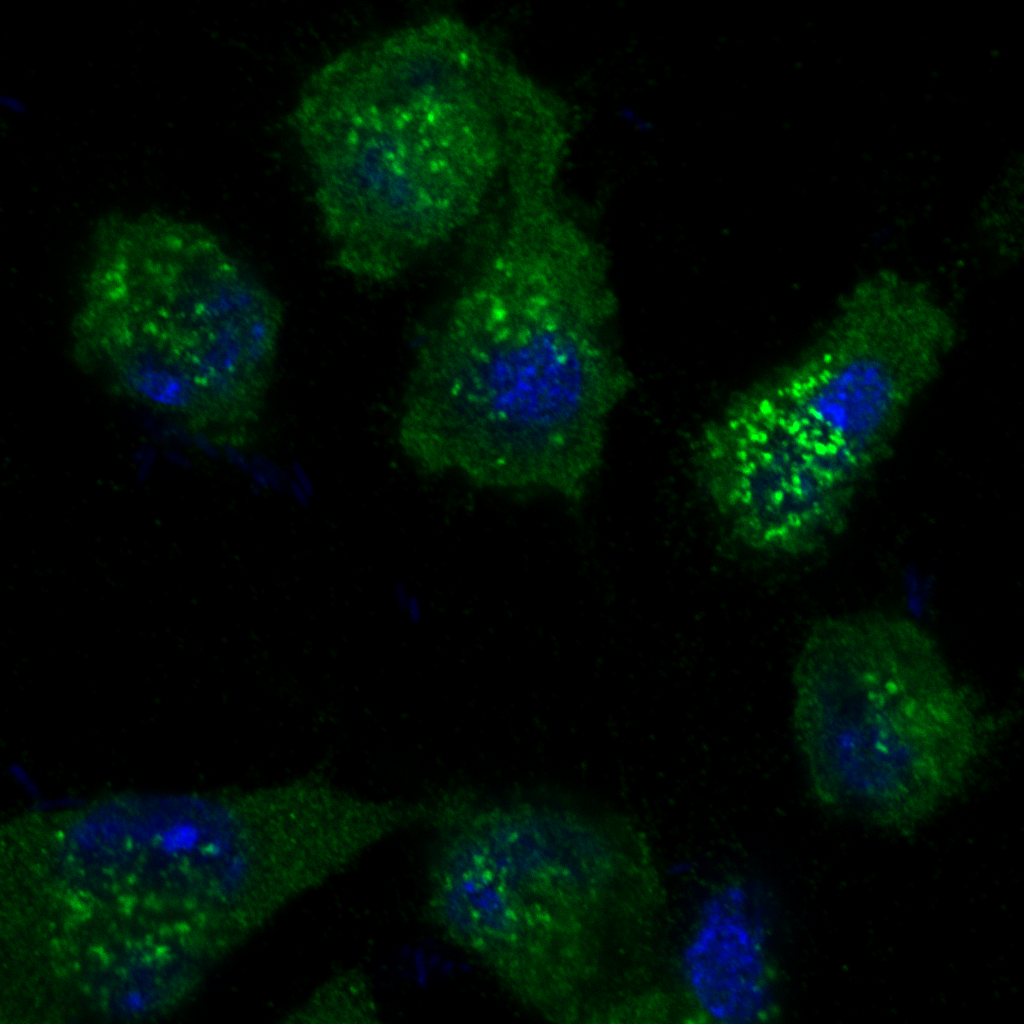

Supplement: Supplementary file 7 — Source data Fig. 6 [file 44318_2026_817_MOESM7_ESM.zip › 6E/6E-3-LLOMe 30 min-C9orf72 KO.tif]

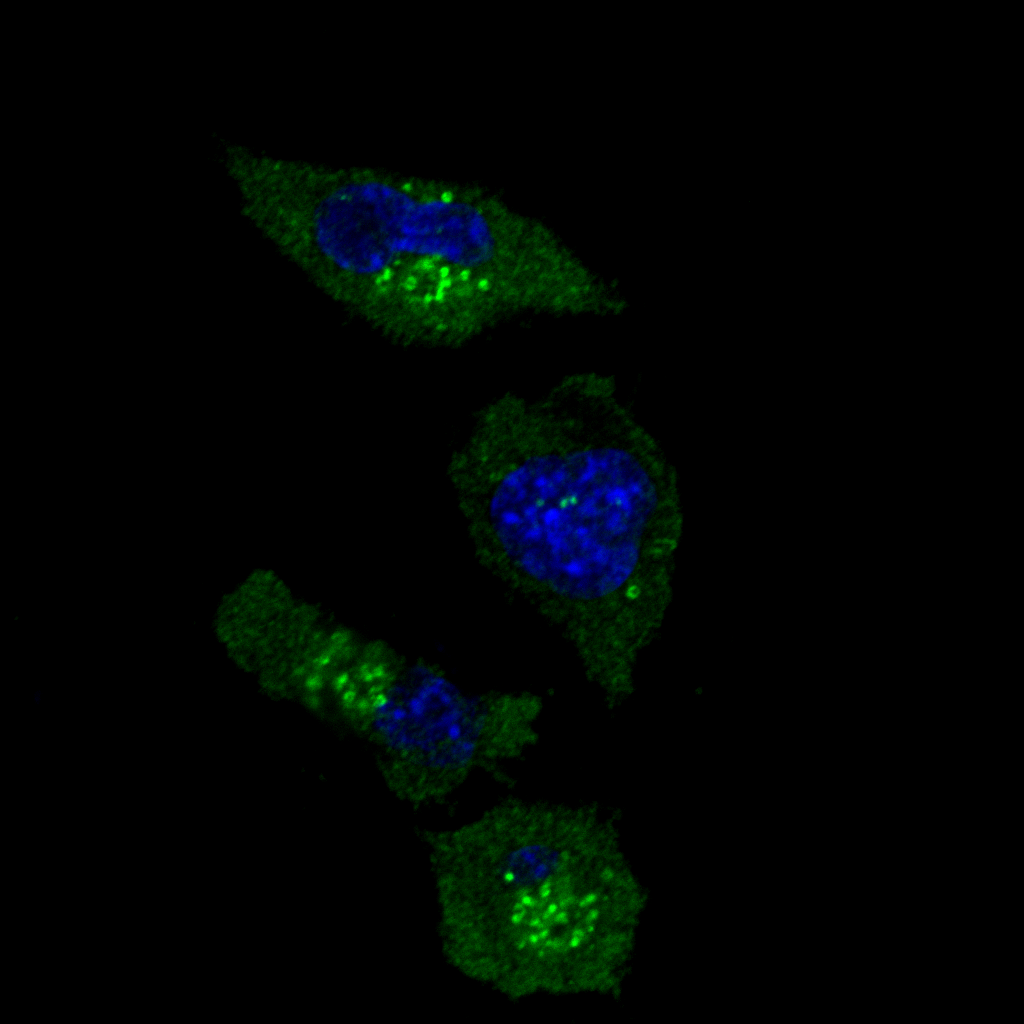

Supplement: Supplementary file 7 — Source data Fig. 6 [file 44318_2026_817_MOESM7_ESM.zip › 6E/6E-3-LLOMe 30 min-dKO.tif]

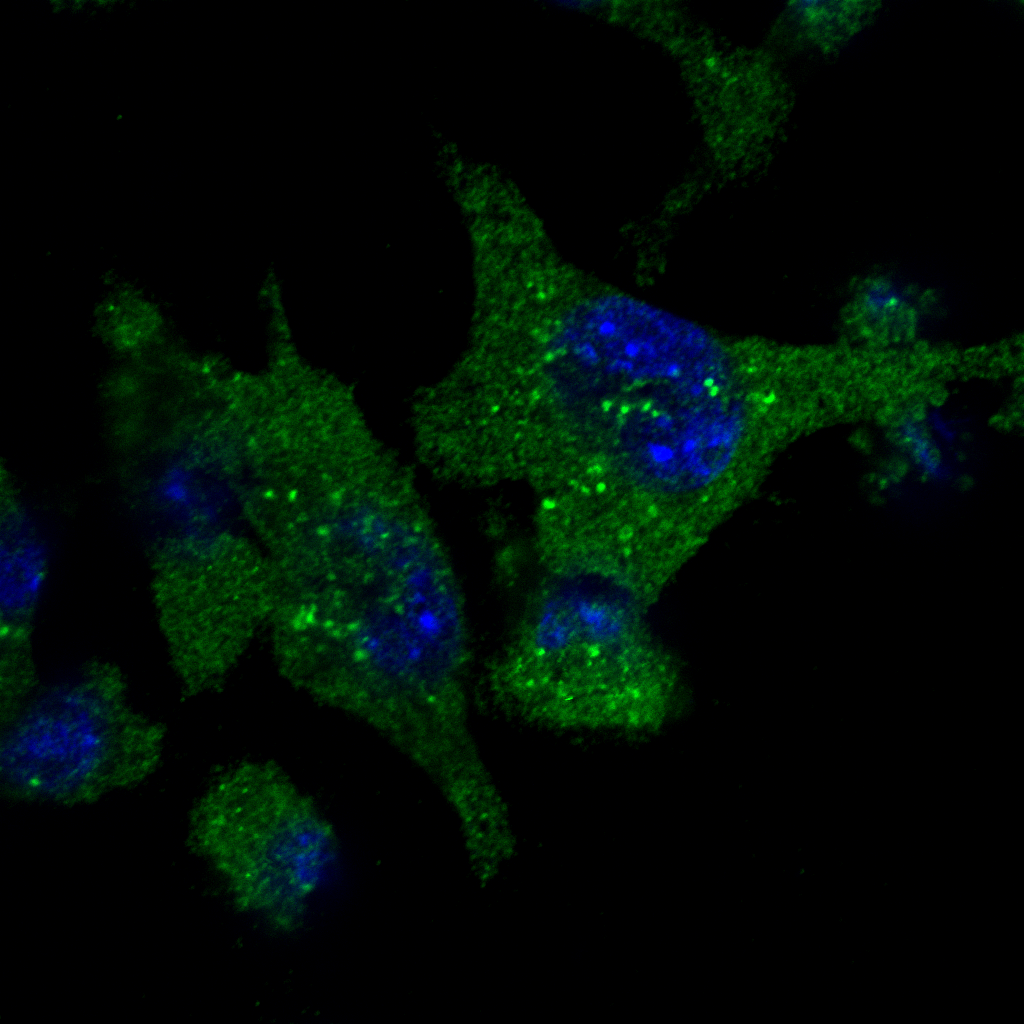

Supplement: Supplementary file 7 — Source data Fig. 6 [file 44318_2026_817_MOESM7_ESM.zip › 6E/6E-3-LLOMe 30 min-Smcr8 KO.tif]

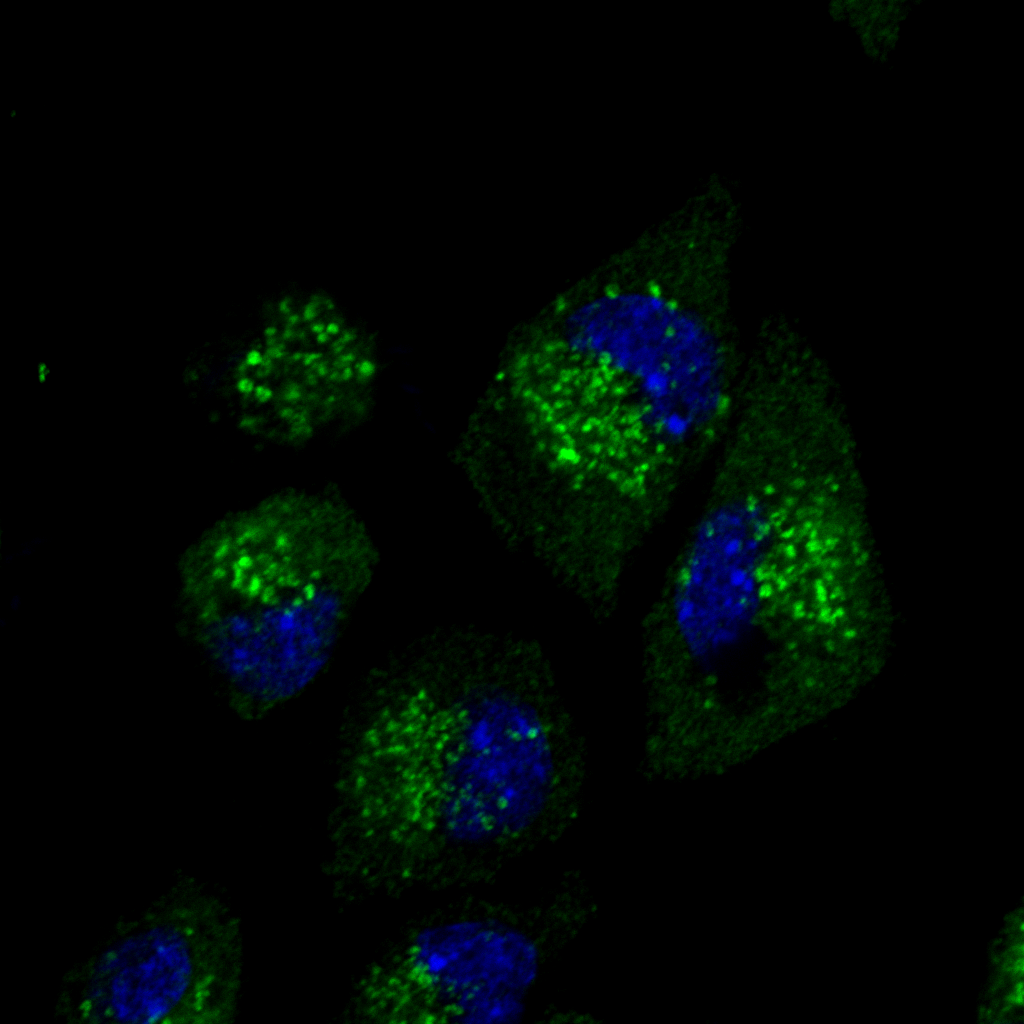

Supplement: Supplementary file 7 — Source data Fig. 6 [file 44318_2026_817_MOESM7_ESM.zip › 6E/6E-3-LLOMe 30 min-WT.tif]

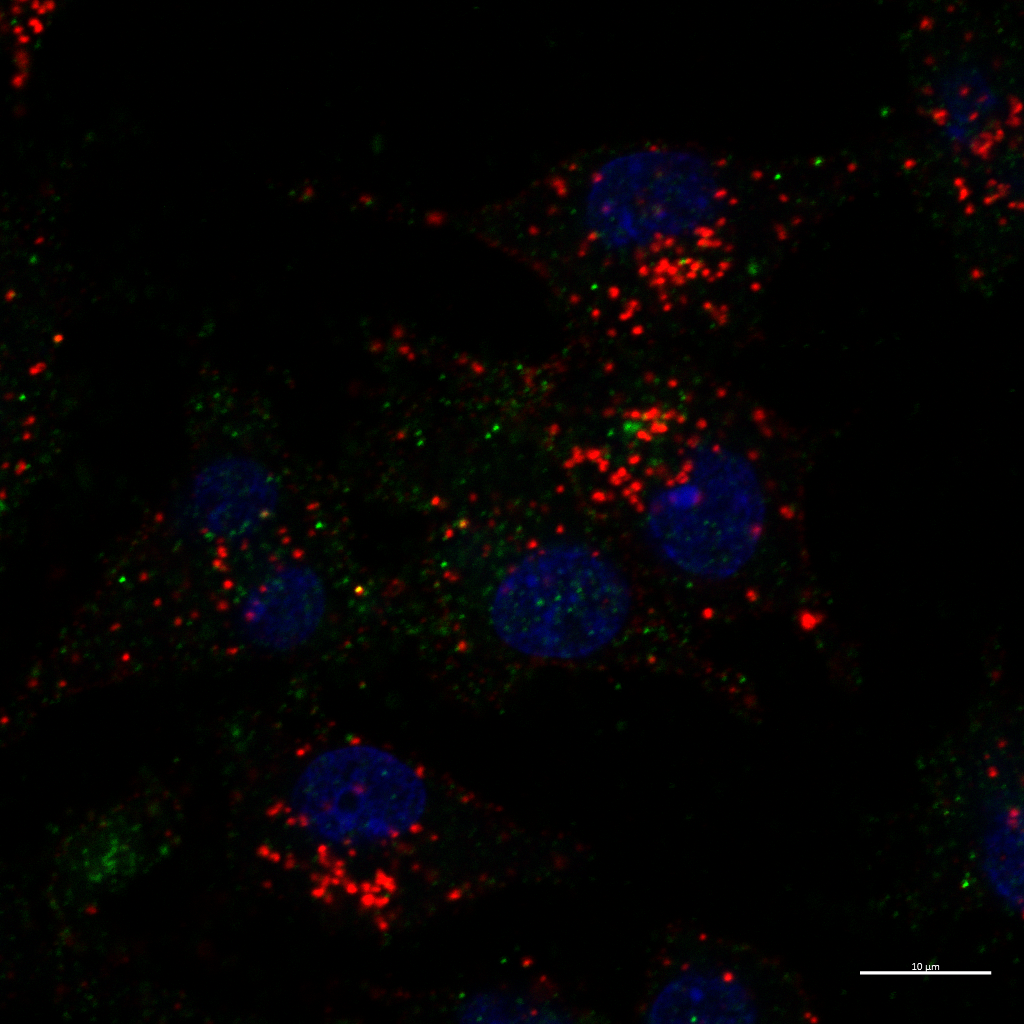

Supplement: Supplementary file 8 — Source data Fig. 7 [file 44318_2026_817_MOESM8_ESM.zip › Figure 7/7A/7A-1-Basal-C9orf72 KO.tif]

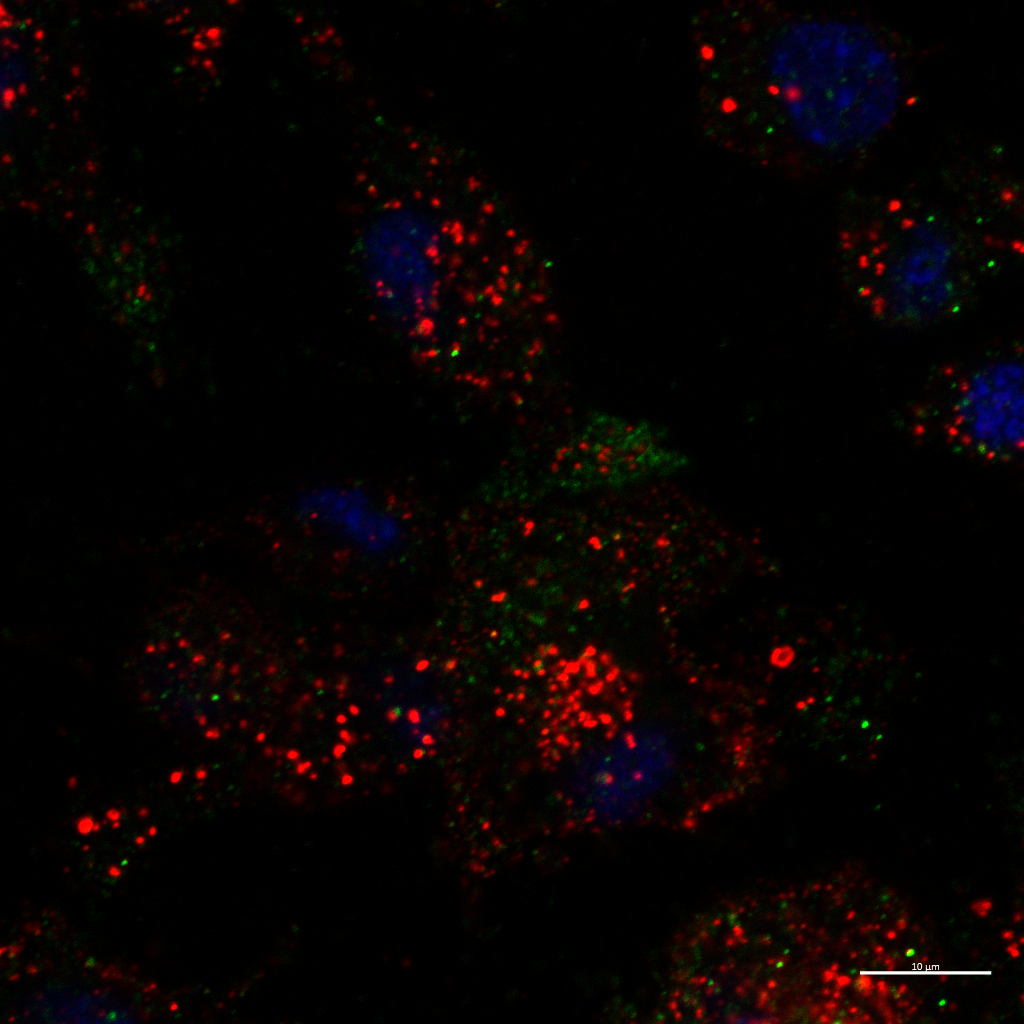

Supplement: Supplementary file 8 — Source data Fig. 7 [file 44318_2026_817_MOESM8_ESM.zip › Figure 7/7A/7A-1-Basal-dKO.tif]

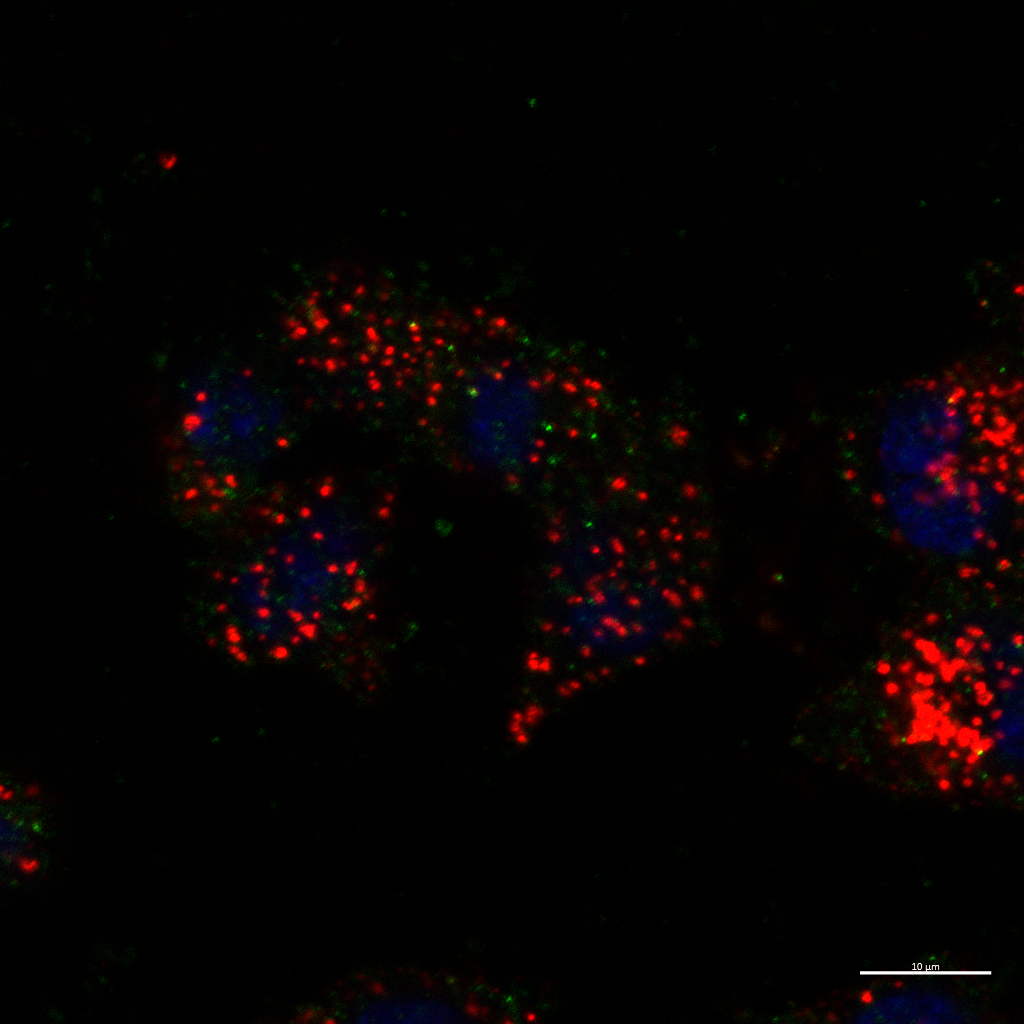

Supplement: Supplementary file 8 — Source data Fig. 7 [file 44318_2026_817_MOESM8_ESM.zip › Figure 7/7A/7A-1-Basal-Smcr8 KO.tif]

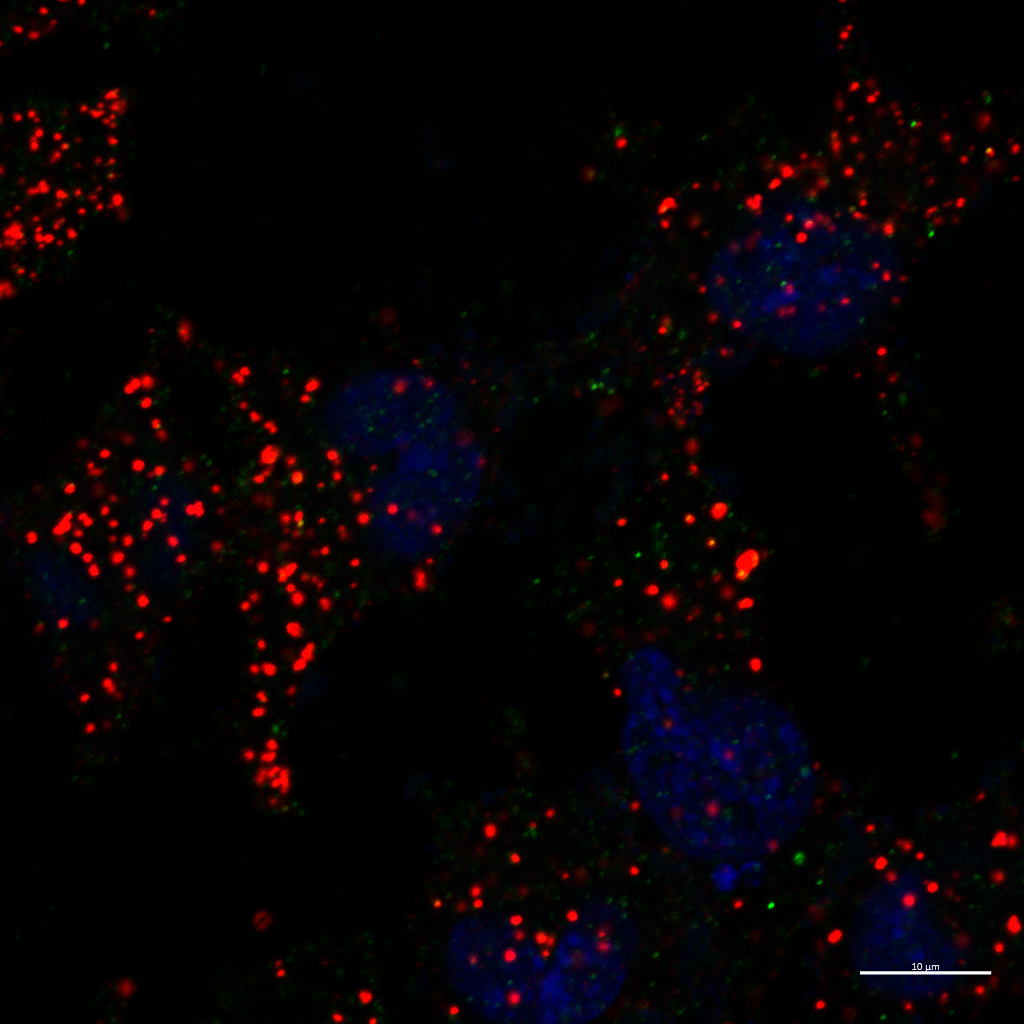

Supplement: Supplementary file 8 — Source data Fig. 7 [file 44318_2026_817_MOESM8_ESM.zip › Figure 7/7A/7A-1-Basal-WT.tif]

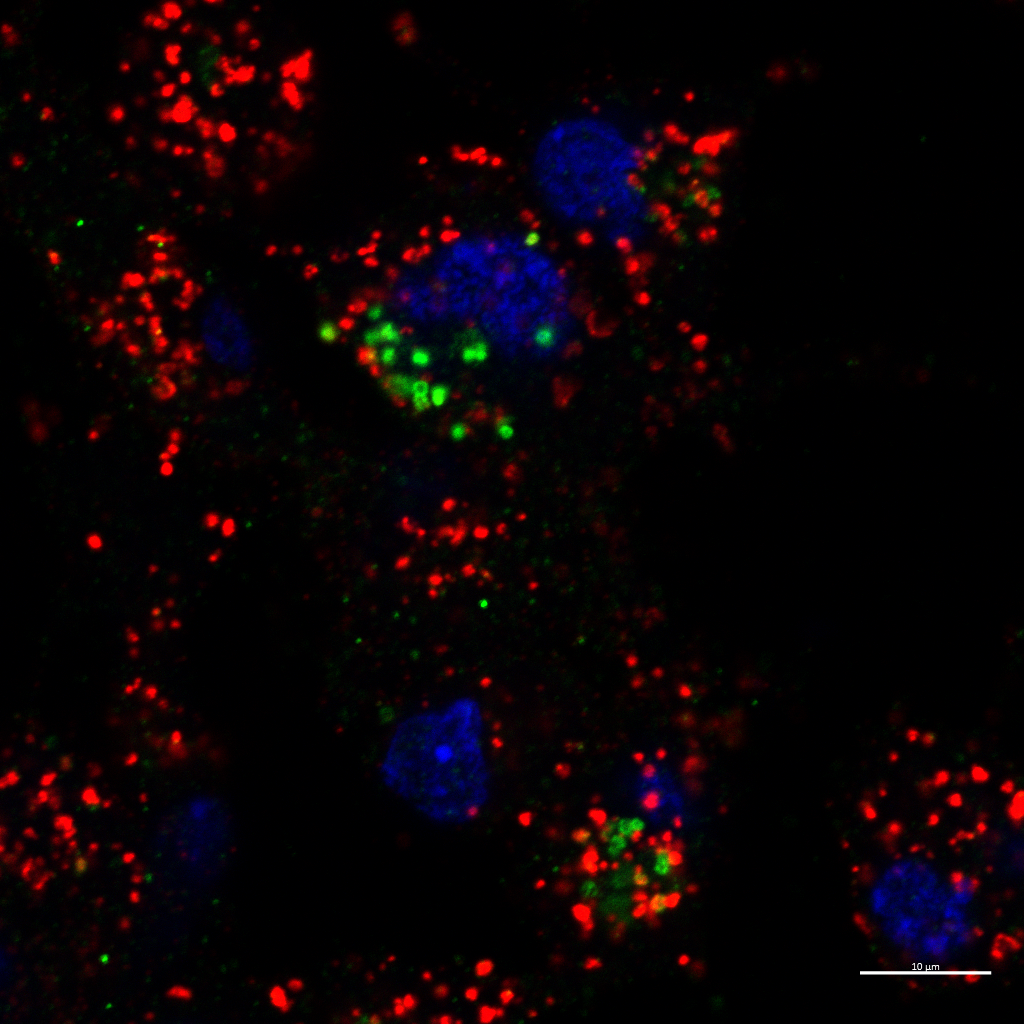

Supplement: Supplementary file 8 — Source data Fig. 7 [file 44318_2026_817_MOESM8_ESM.zip › Figure 7/7A/7A-2-LLOMe 30 min-C9orf72 KO.tif]

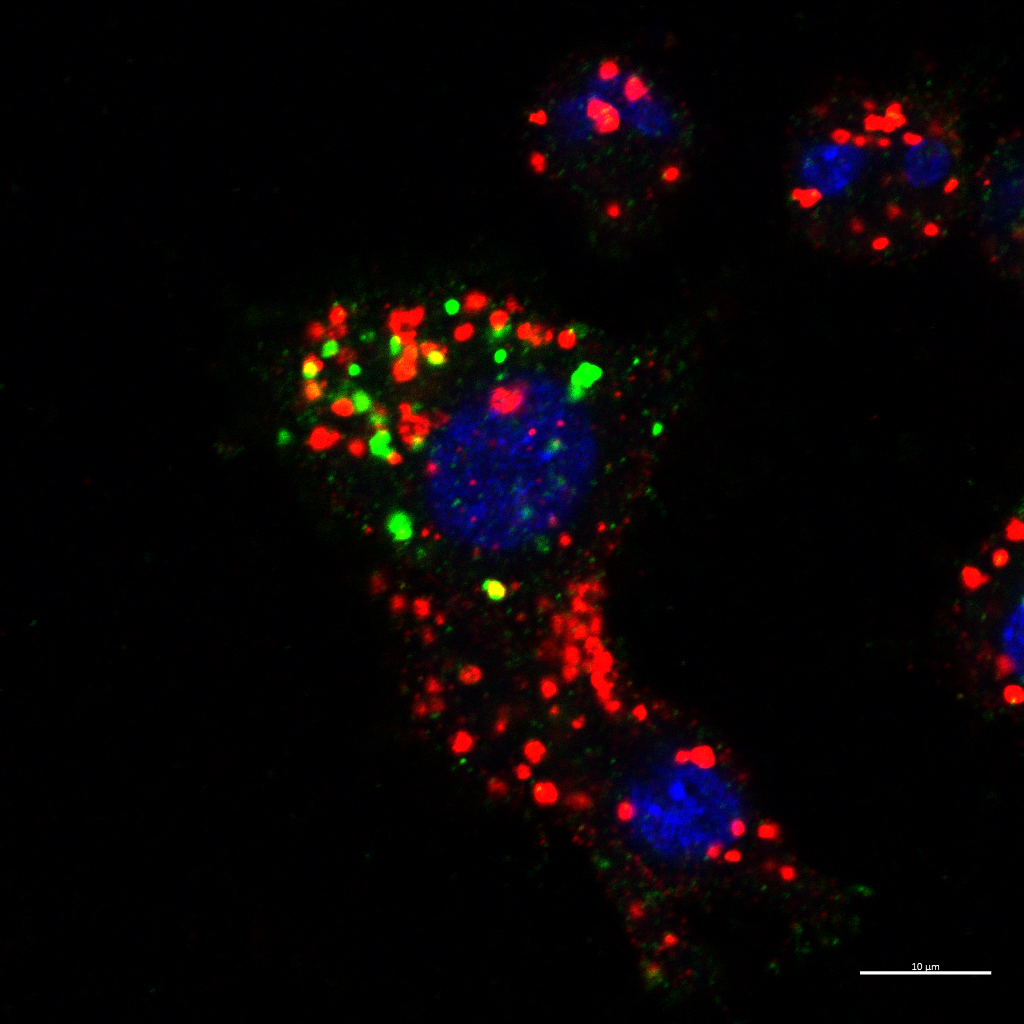

Supplement: Supplementary file 8 — Source data Fig. 7 [file 44318_2026_817_MOESM8_ESM.zip › Figure 7/7A/7A-2-LLOMe 30 min-dKO.tif]

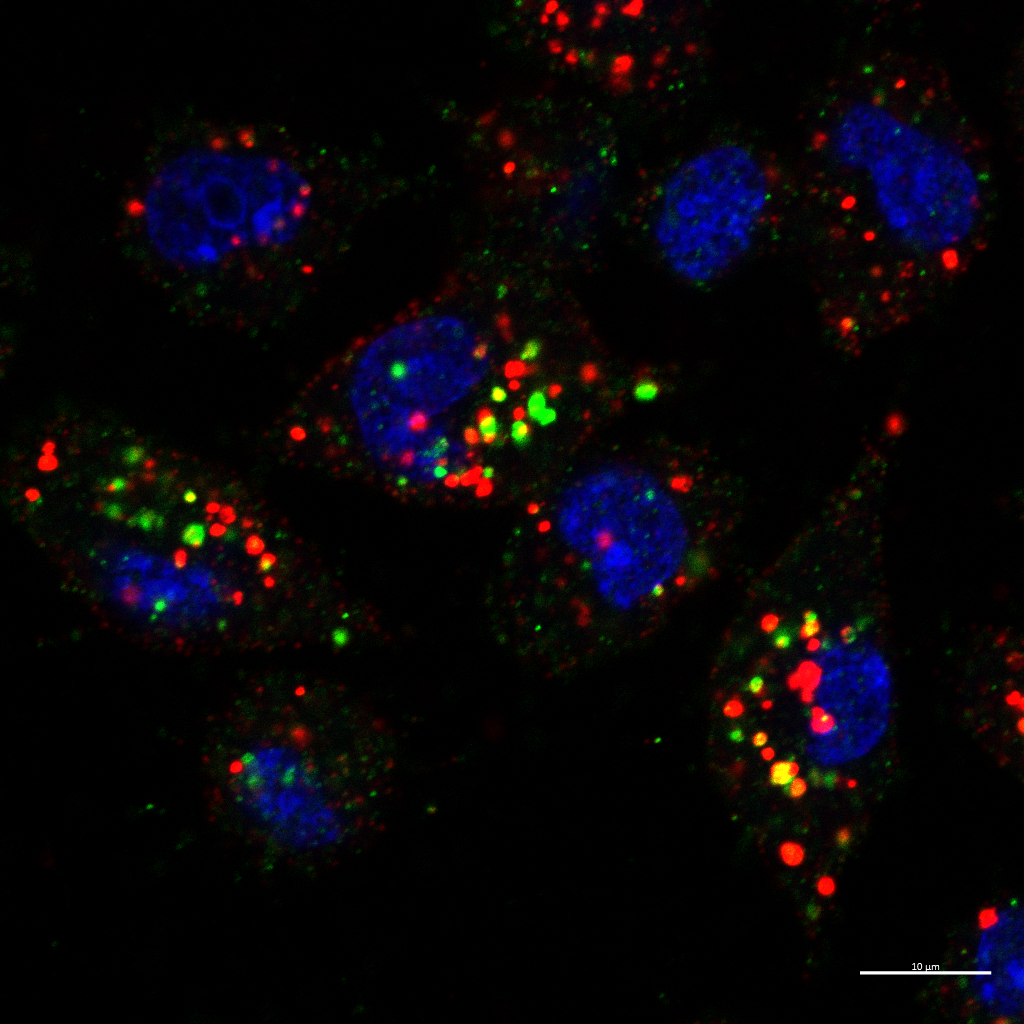

Supplement: Supplementary file 8 — Source data Fig. 7 [file 44318_2026_817_MOESM8_ESM.zip › Figure 7/7A/7A-2-LLOMe 30 min-Smcr8 KO.tif]

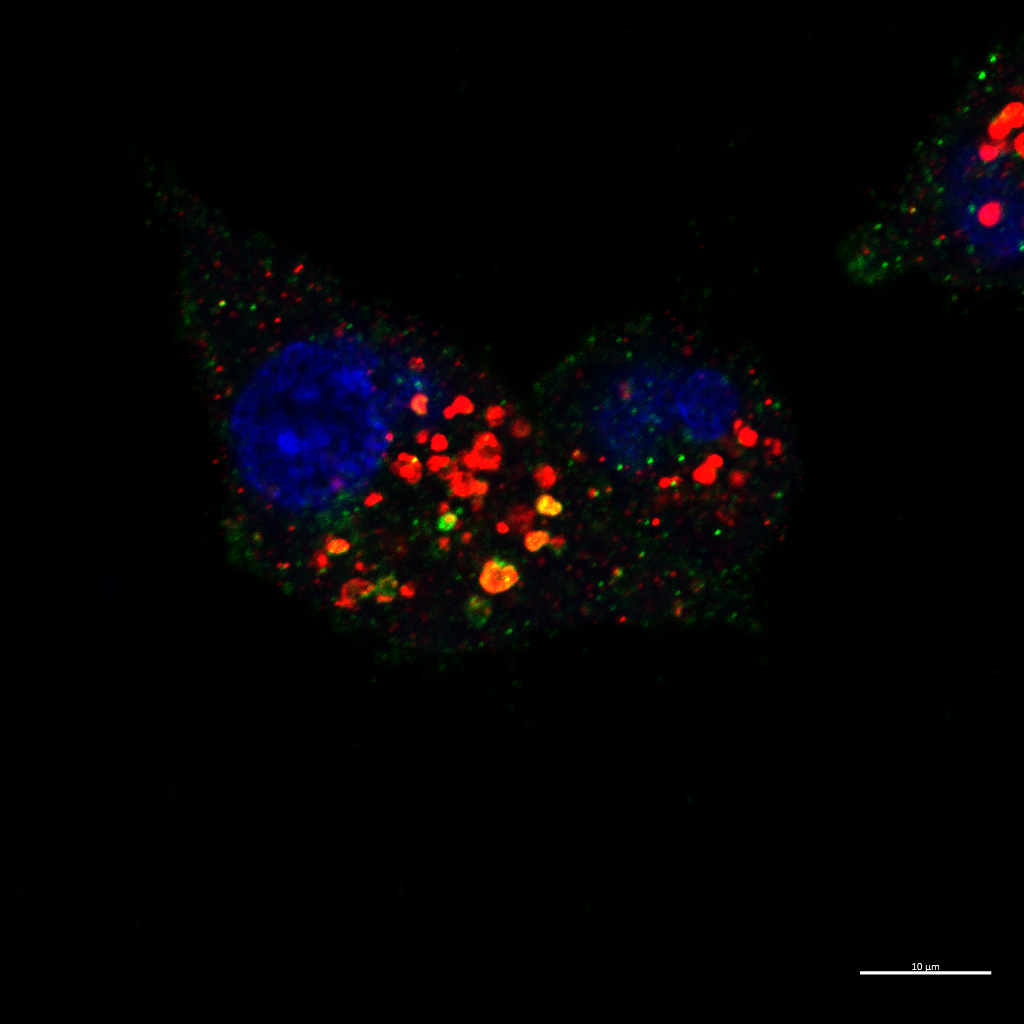

Supplement: Supplementary file 8 — Source data Fig. 7 [file 44318_2026_817_MOESM8_ESM.zip › Figure 7/7A/7A-2-LLOMe 30 min-WT.tif]

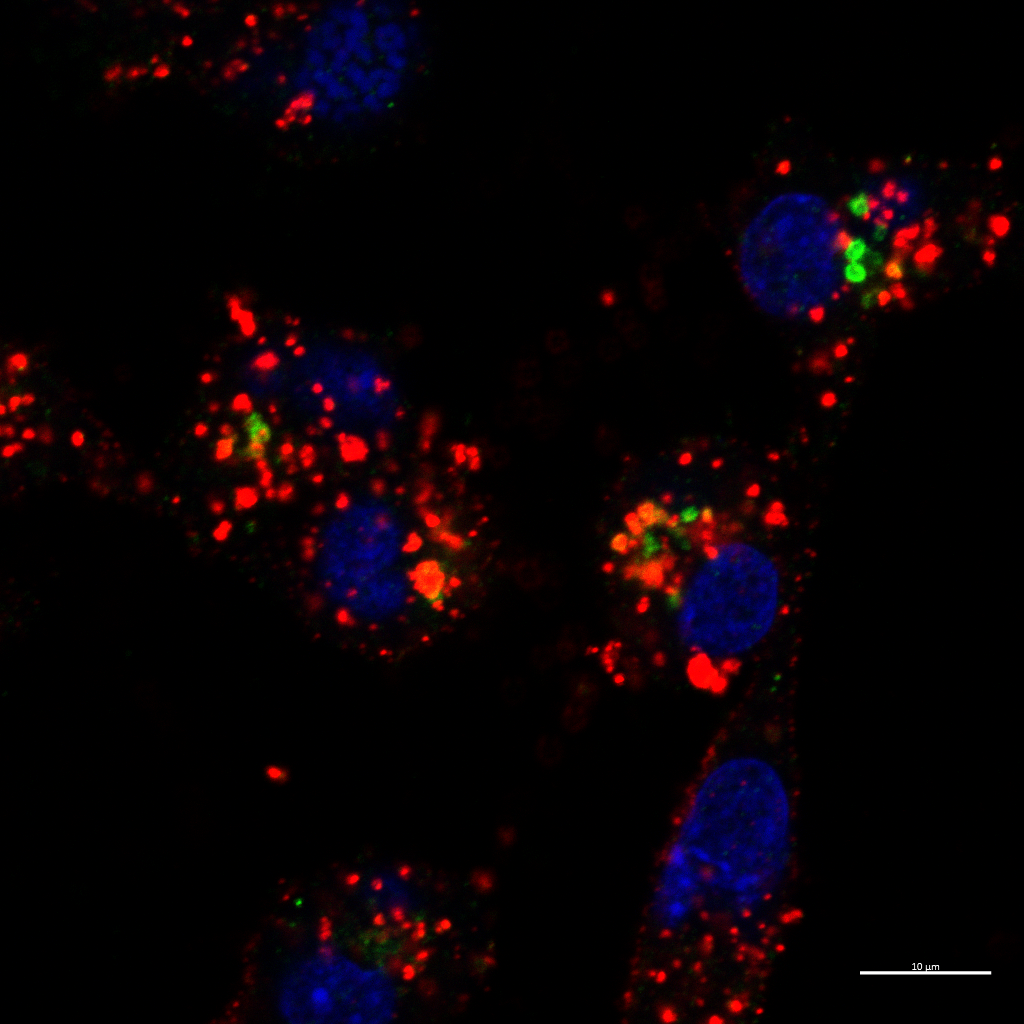

Supplement: Supplementary file 8 — Source data Fig. 7 [file 44318_2026_817_MOESM8_ESM.zip › Figure 7/7A/7A-3-Washout 3 h -C9orf72 KO.tif]

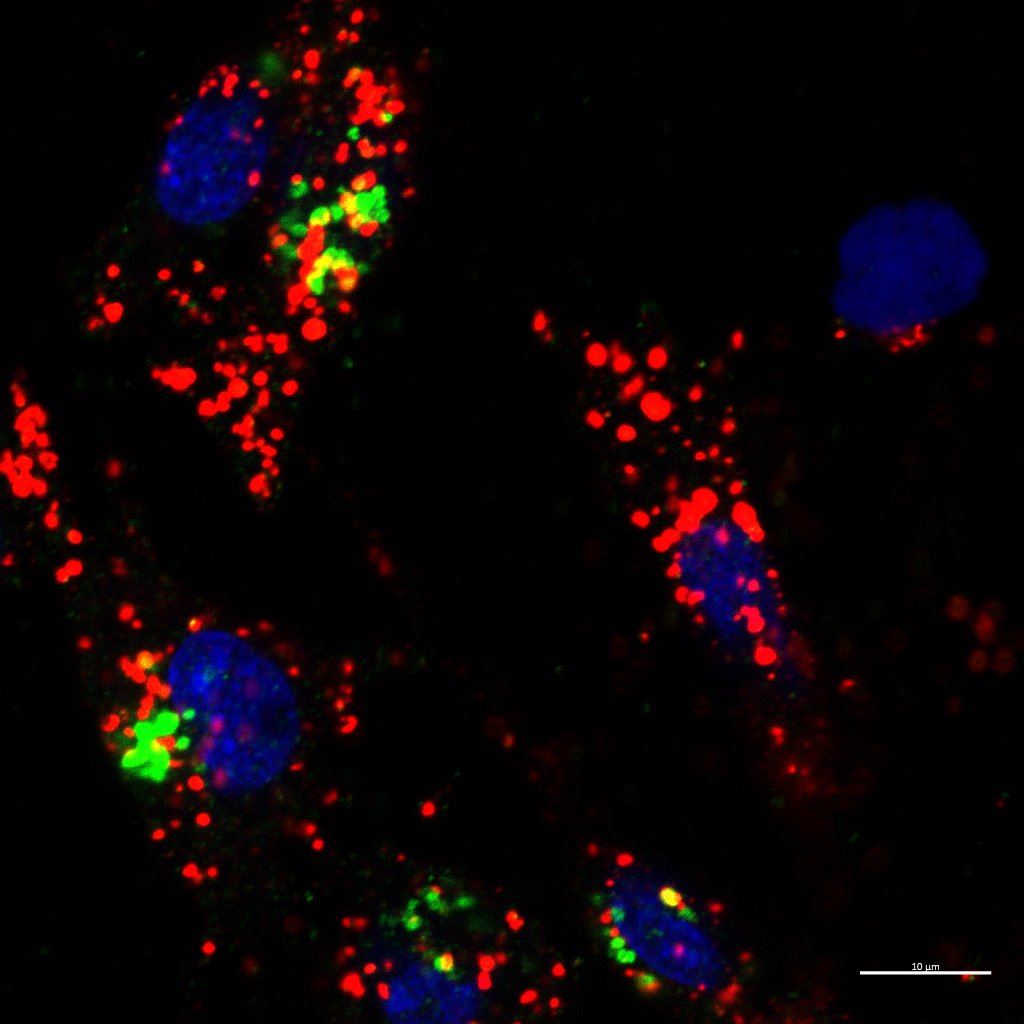

Supplement: Supplementary file 8 — Source data Fig. 7 [file 44318_2026_817_MOESM8_ESM.zip › Figure 7/7A/7A-3-Washout 3 h-dKO.tif]

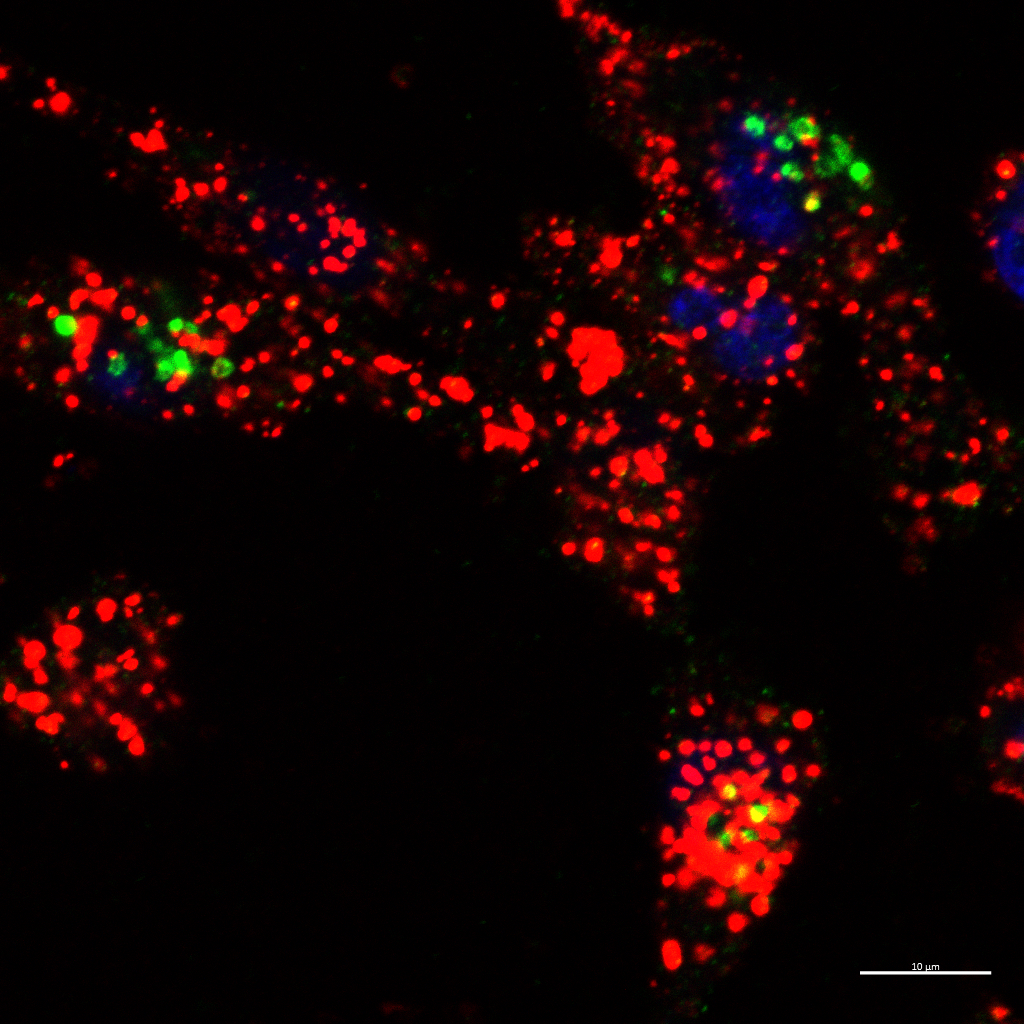

Supplement: Supplementary file 8 — Source data Fig. 7 [file 44318_2026_817_MOESM8_ESM.zip › Figure 7/7A/7A-3-Washout 3 h-Smcr8 KO.tif]

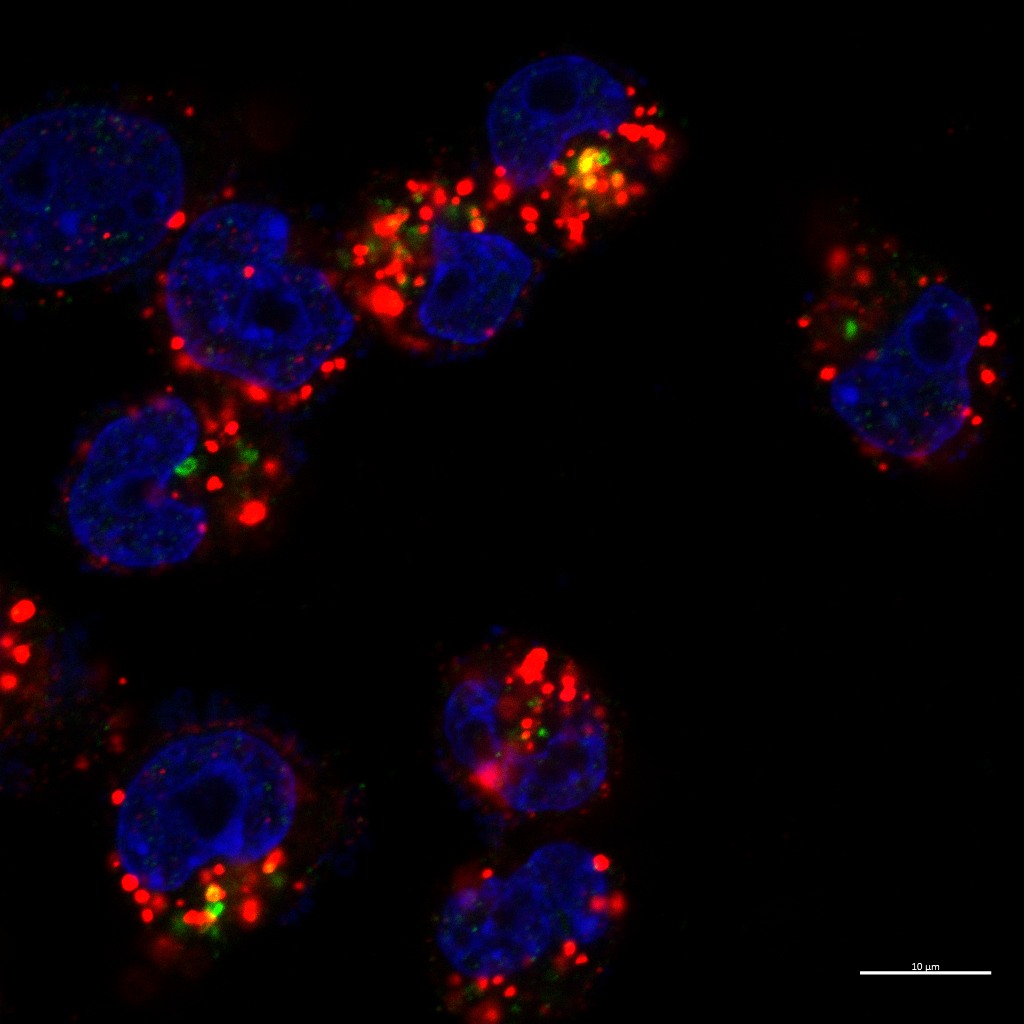

Supplement: Supplementary file 8 — Source data Fig. 7 [file 44318_2026_817_MOESM8_ESM.zip › Figure 7/7A/7A-3-Washout 3 h-WT.tif]

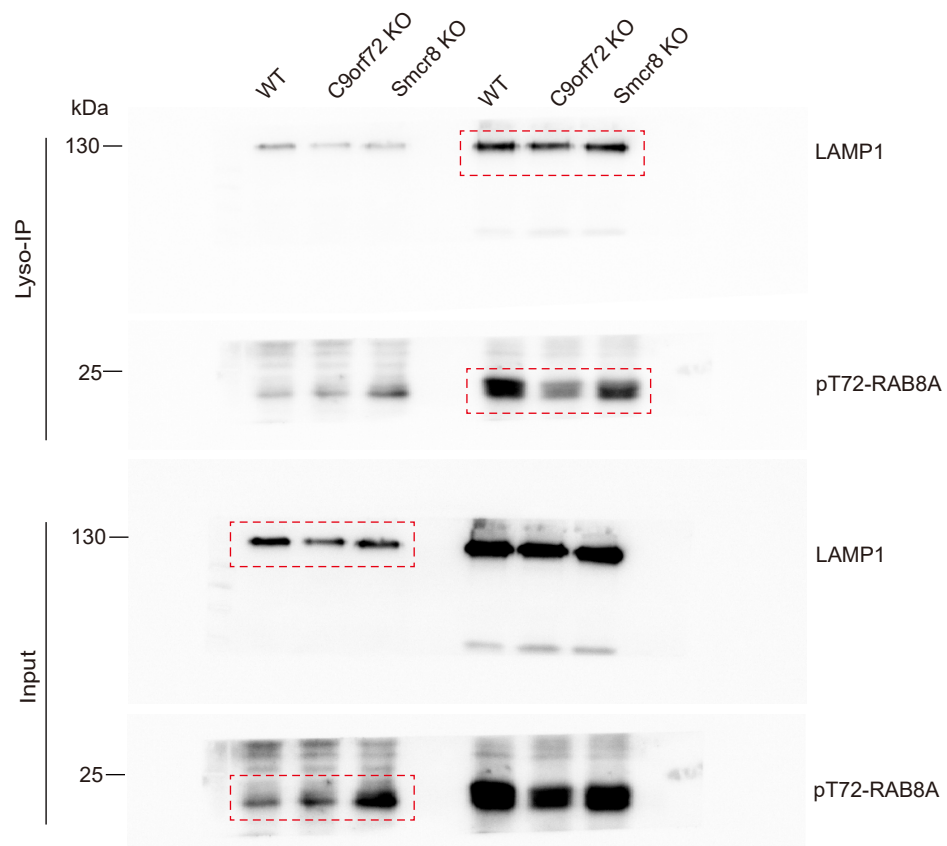

Supplement: Supplementary file 8 — Source data Fig. 7 [file 44318_2026_817_MOESM8_ESM.zip › Figure 7/7C/7C.pdf]

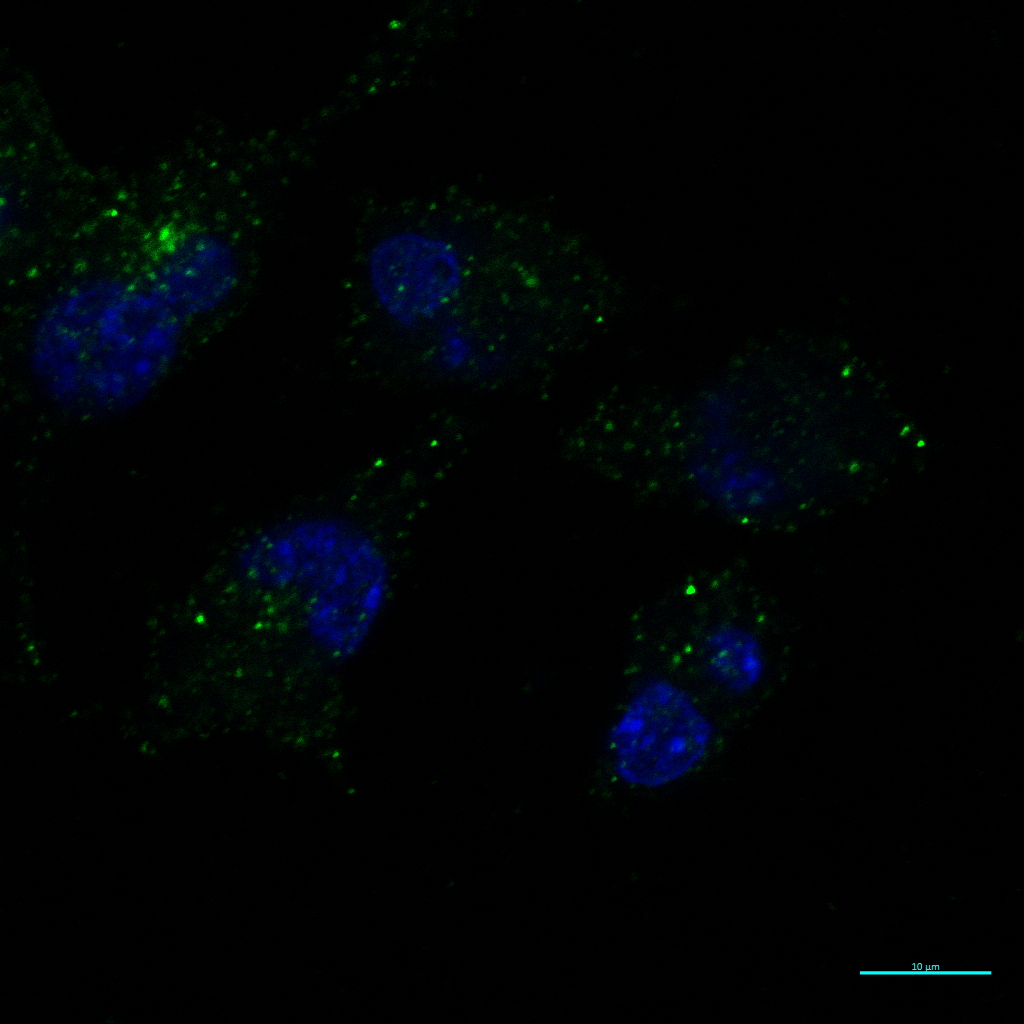

Supplement: Supplementary file 8 — Source data Fig. 7 [file 44318_2026_817_MOESM8_ESM.zip › Figure 7/7D/7D-1-Basal-C9orf72 KO.tif]

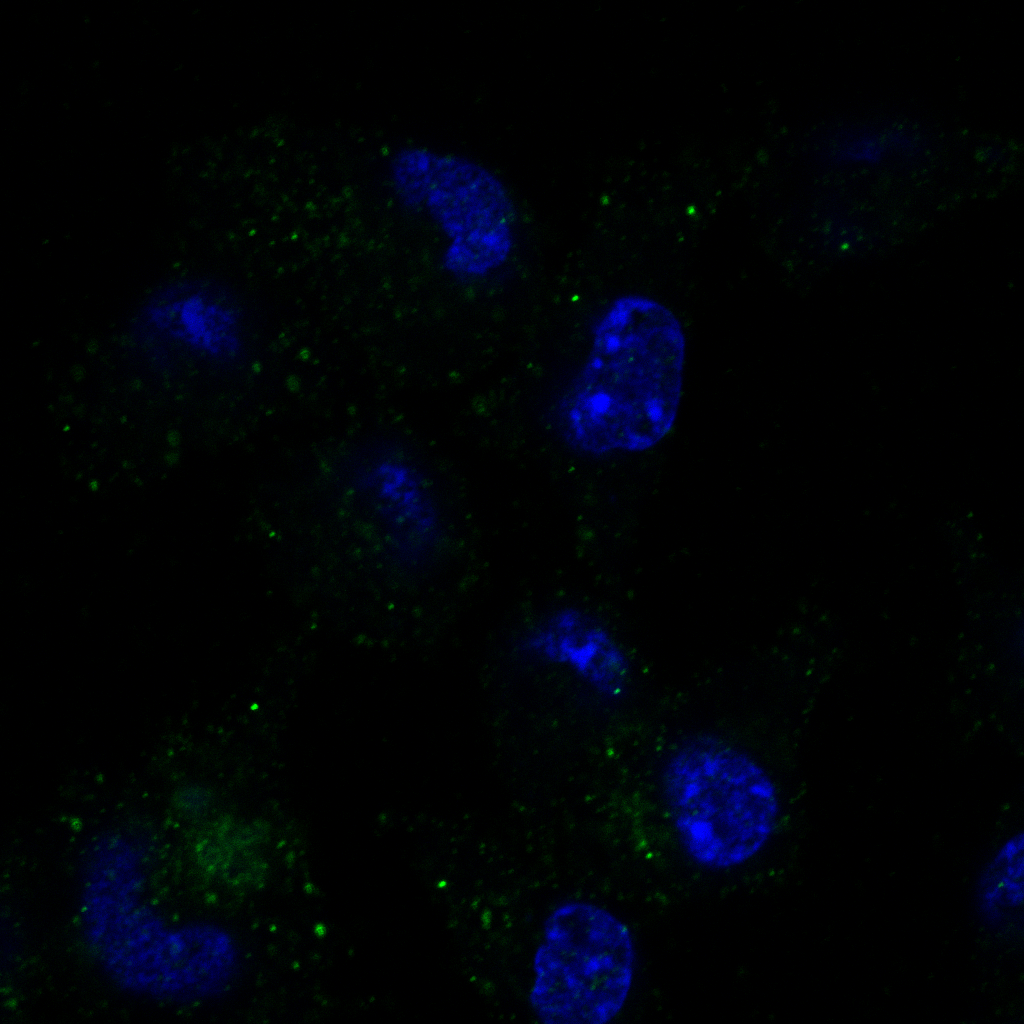

Supplement: Supplementary file 8 — Source data Fig. 7 [file 44318_2026_817_MOESM8_ESM.zip › Figure 7/7D/7D-1-Basal-dKO.tif]

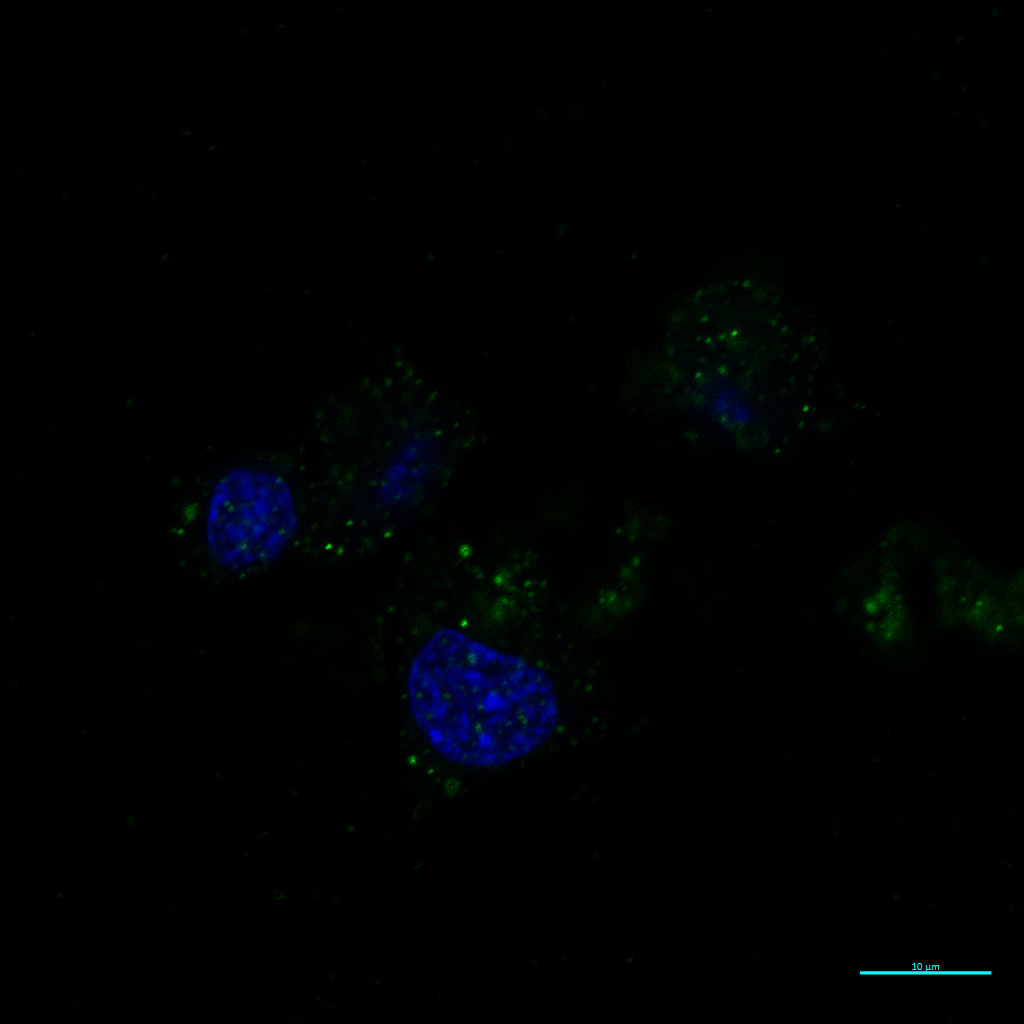

Supplement: Supplementary file 8 — Source data Fig. 7 [file 44318_2026_817_MOESM8_ESM.zip › Figure 7/7D/7D-1-Basal-Smcr8 KO.tif]

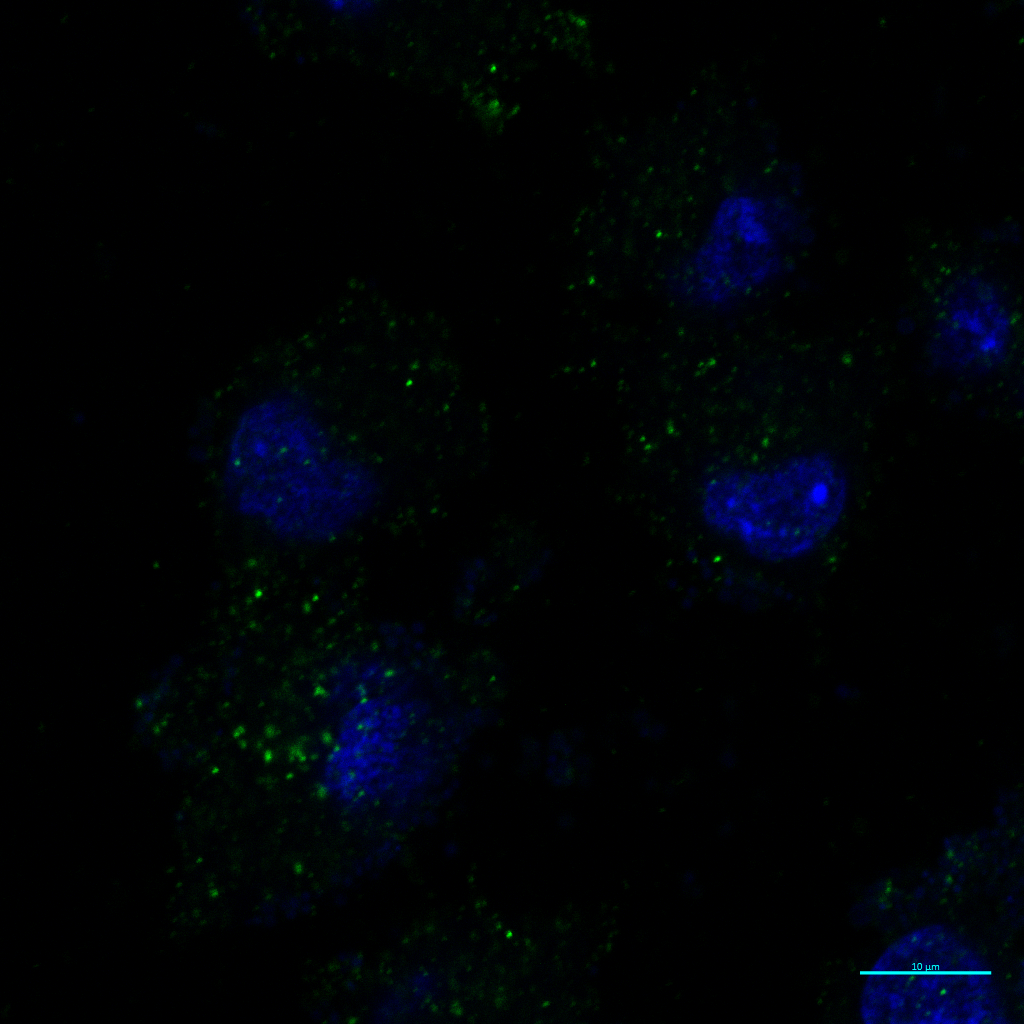

Supplement: Supplementary file 8 — Source data Fig. 7 [file 44318_2026_817_MOESM8_ESM.zip › Figure 7/7D/7D-1-Basal-WT.tif]

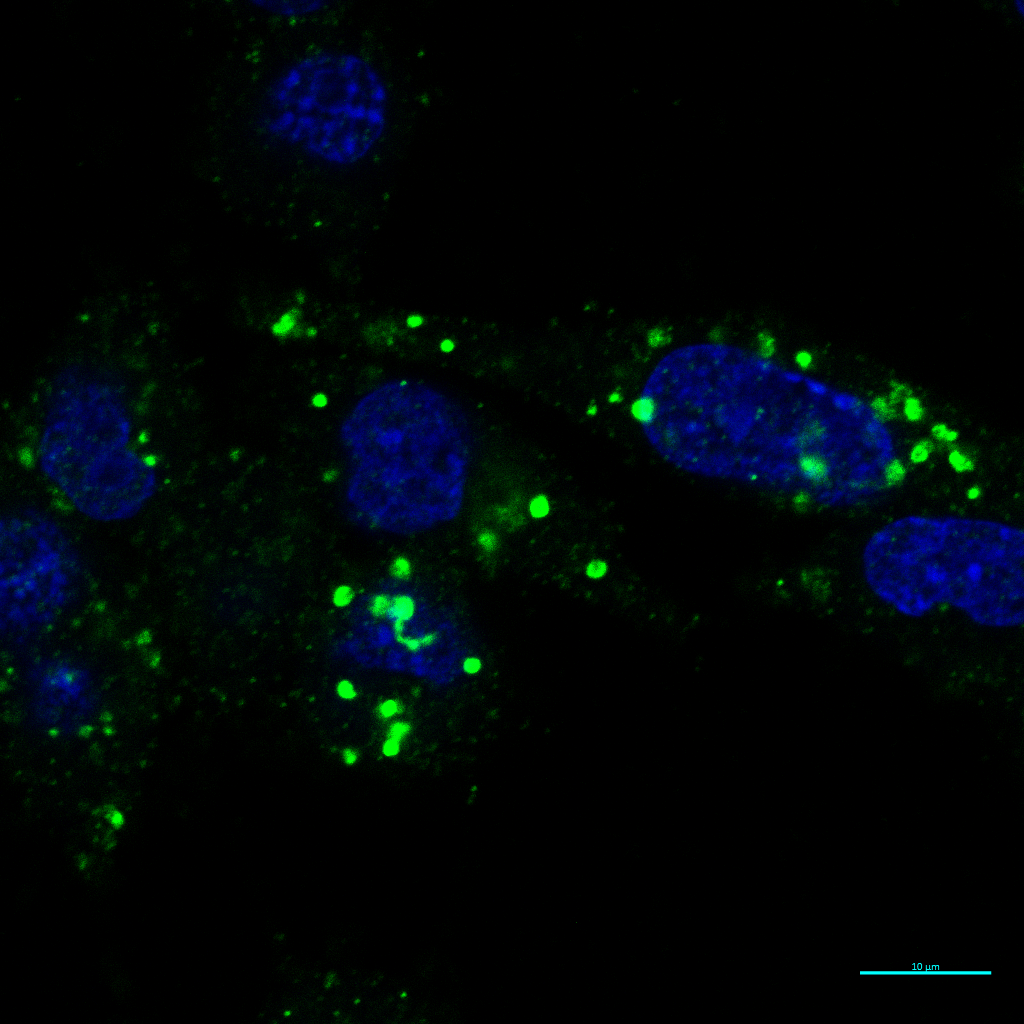

Supplement: Supplementary file 8 — Source data Fig. 7 [file 44318_2026_817_MOESM8_ESM.zip › Figure 7/7D/7D-2-LLOMe 30 min-C9orf72 KO.tif]

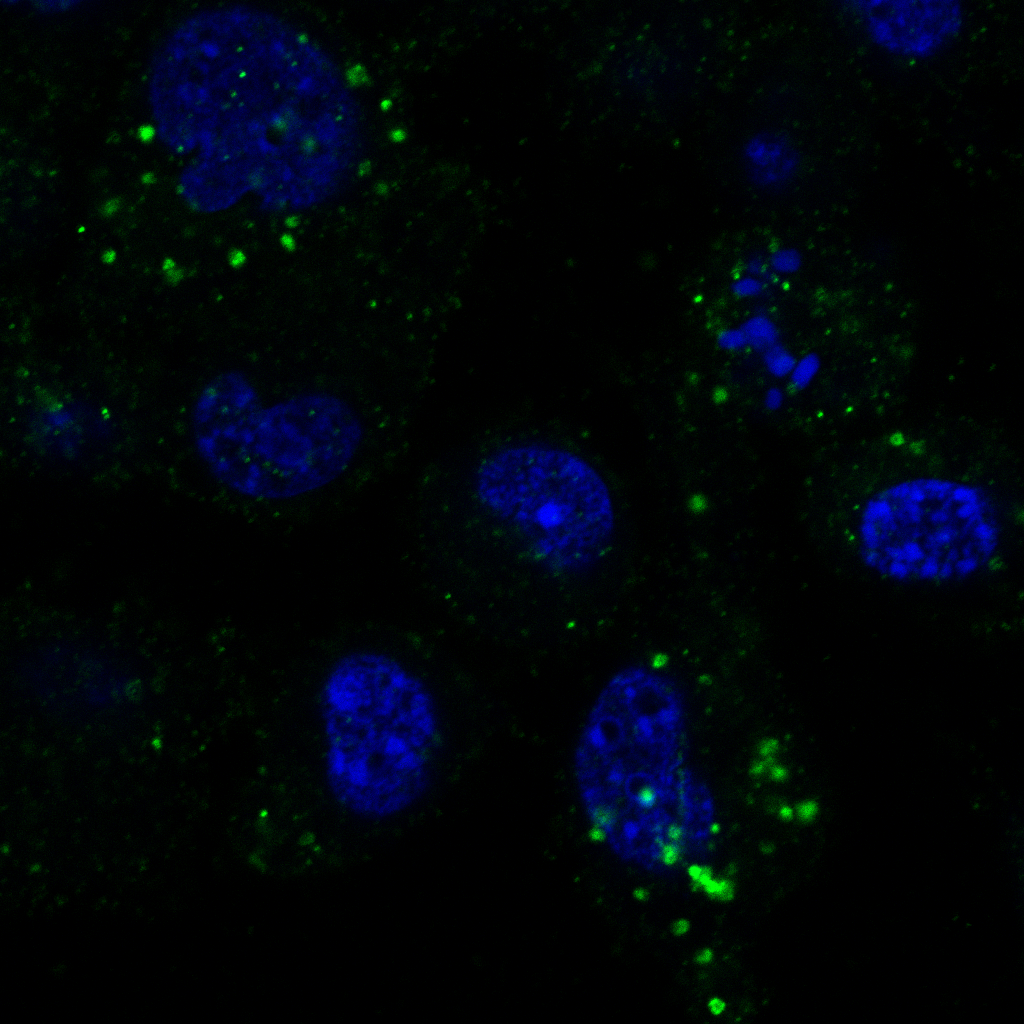

Supplement: Supplementary file 8 — Source data Fig. 7 [file 44318_2026_817_MOESM8_ESM.zip › Figure 7/7D/7D-2-LLOMe 30 min-dKO.tif]

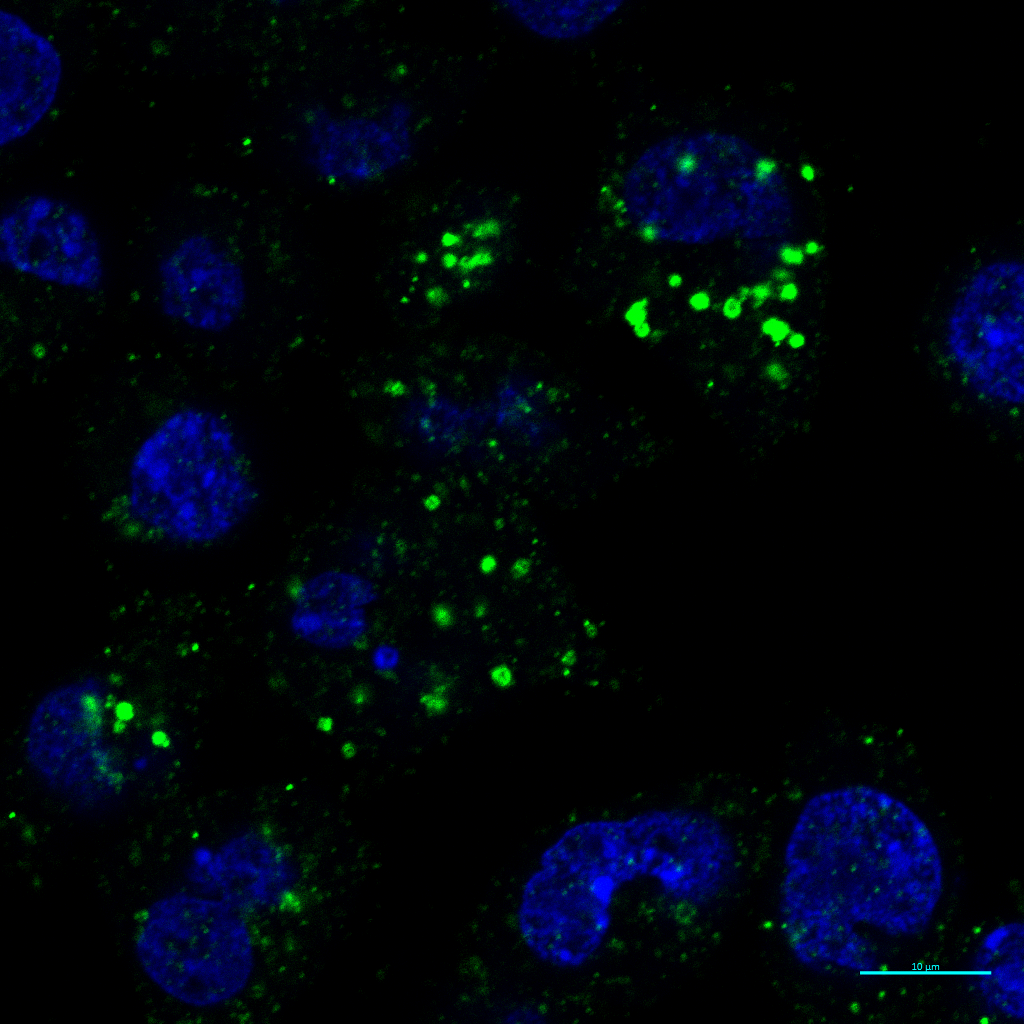

Supplement: Supplementary file 8 — Source data Fig. 7 [file 44318_2026_817_MOESM8_ESM.zip › Figure 7/7D/7D-2-LLOMe 30 min-Smcr8 KO.tif]

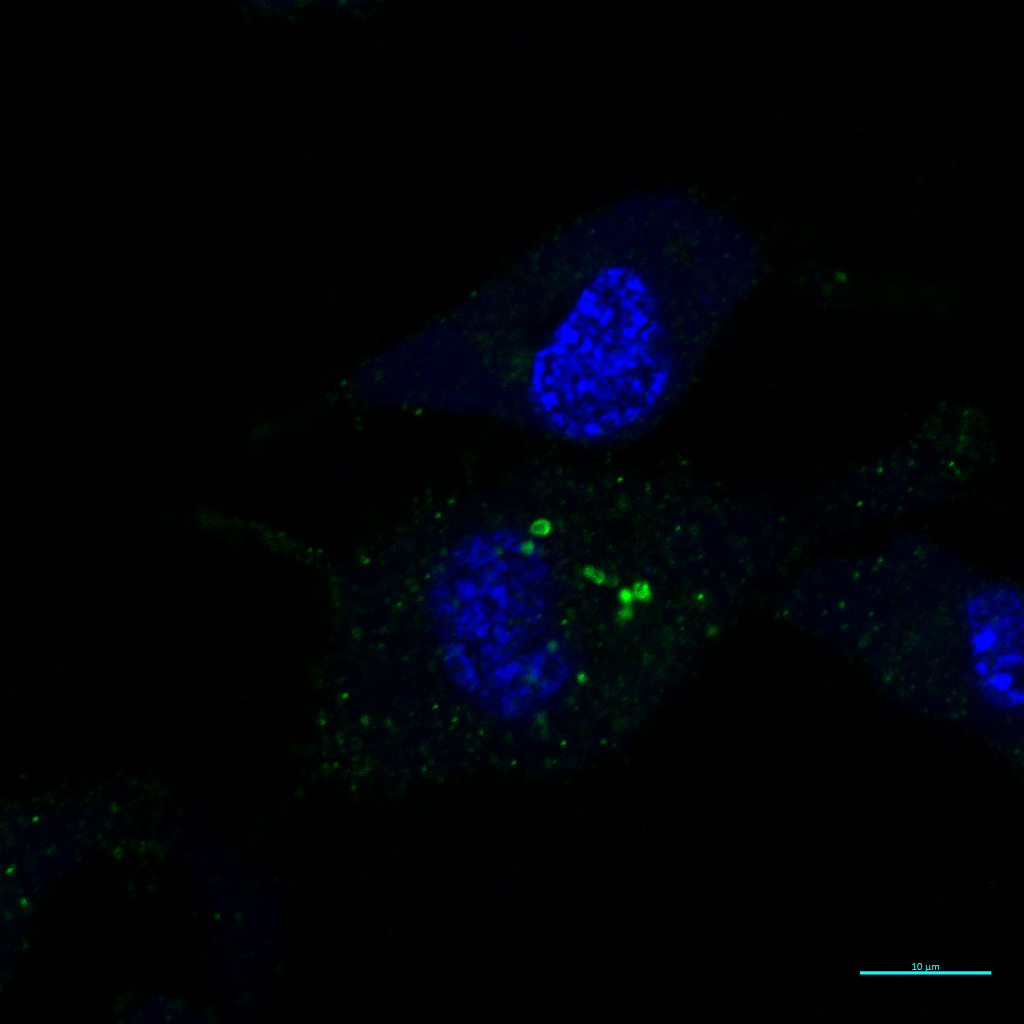

Supplement: Supplementary file 8 — Source data Fig. 7 [file 44318_2026_817_MOESM8_ESM.zip › Figure 7/7D/7D-2-LLOMe 30 min-WT.tif]

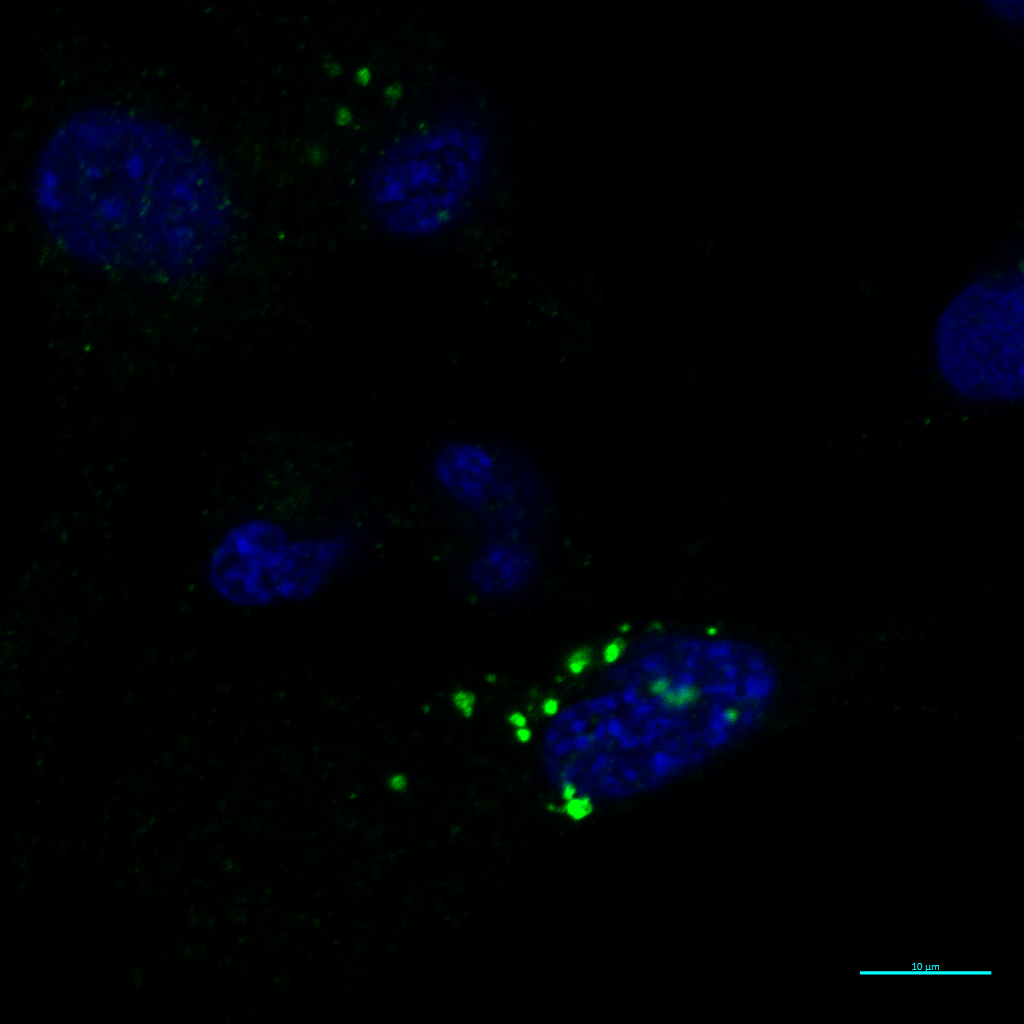

Supplement: Supplementary file 8 — Source data Fig. 7 [file 44318_2026_817_MOESM8_ESM.zip › Figure 7/7D/7D-2-Washout 3 h-C9orf72 KO.tif]

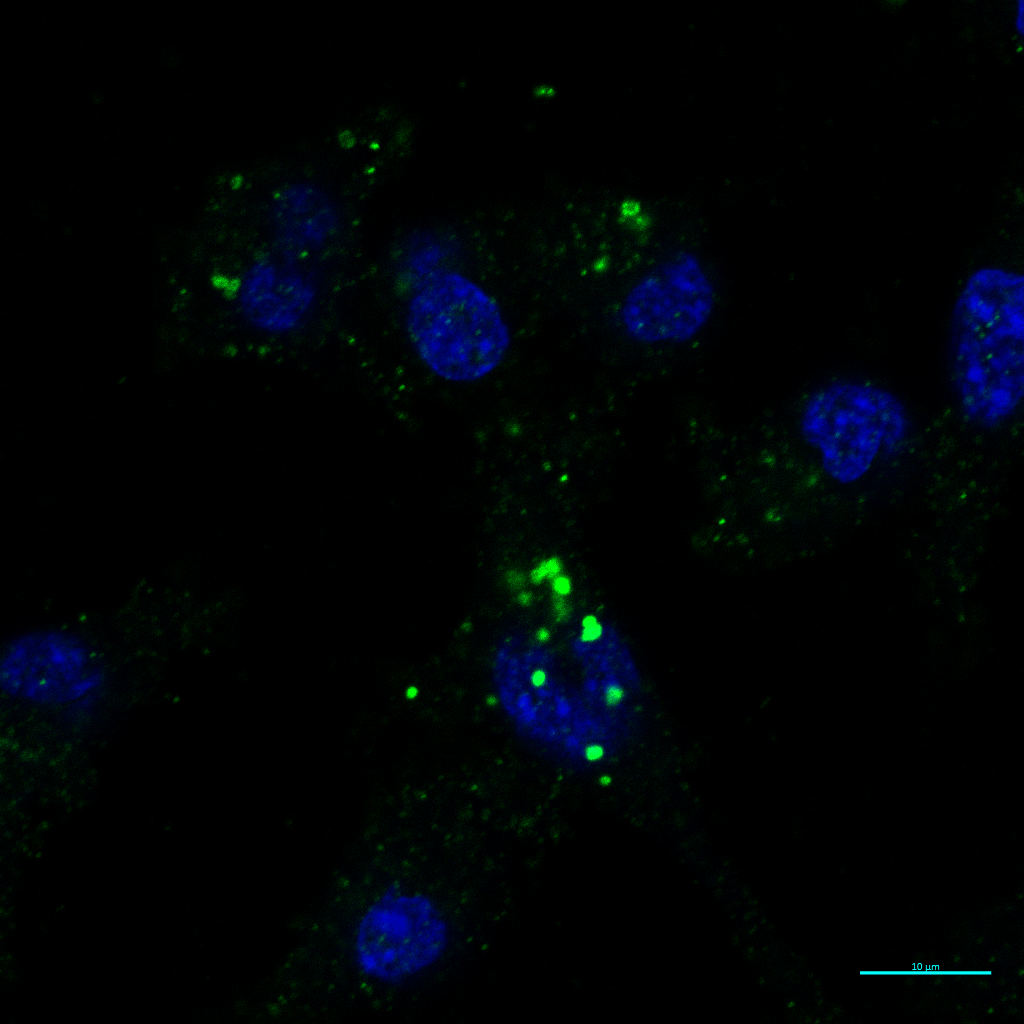

Supplement: Supplementary file 8 — Source data Fig. 7 [file 44318_2026_817_MOESM8_ESM.zip › Figure 7/7D/7D-2-Washout 3 h-dKO.tif]

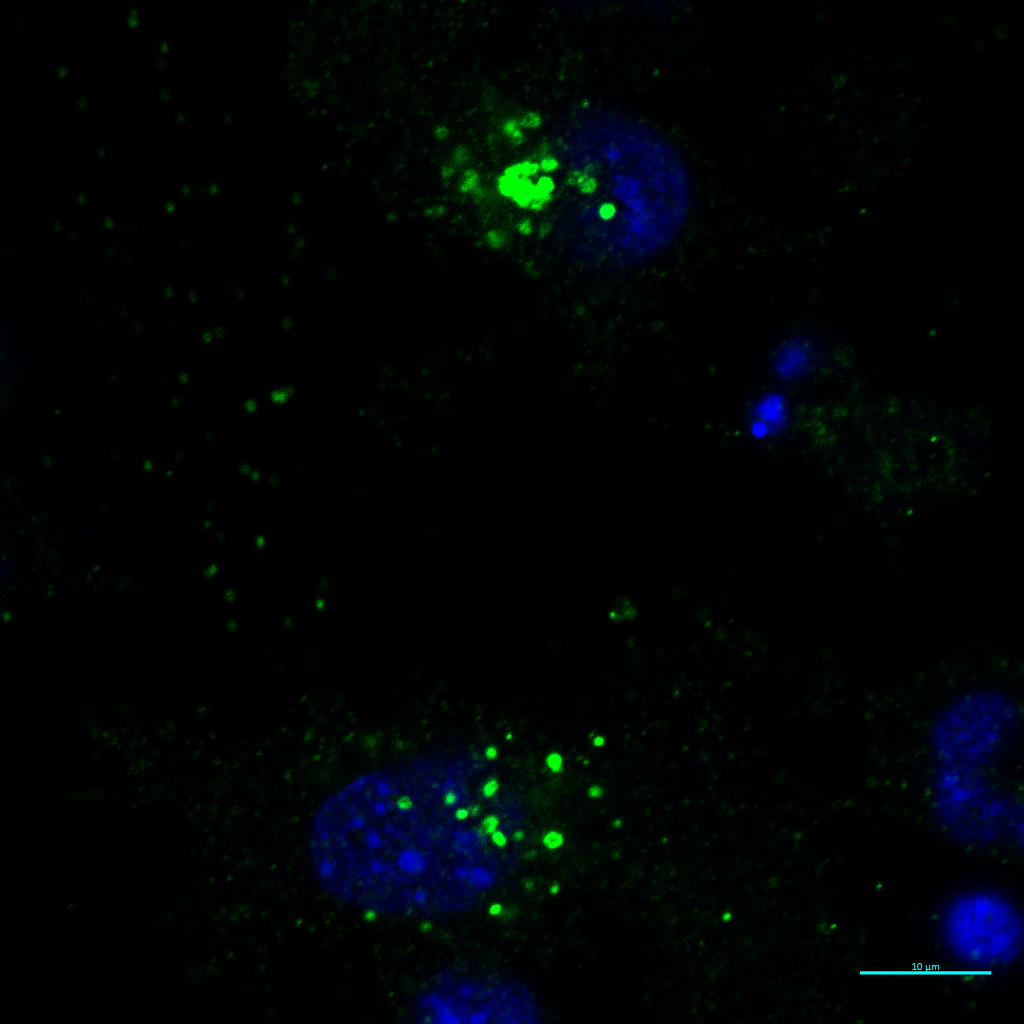

Supplement: Supplementary file 8 — Source data Fig. 7 [file 44318_2026_817_MOESM8_ESM.zip › Figure 7/7D/7D-2-Washout 3 h-Smcr8 KO.tif]

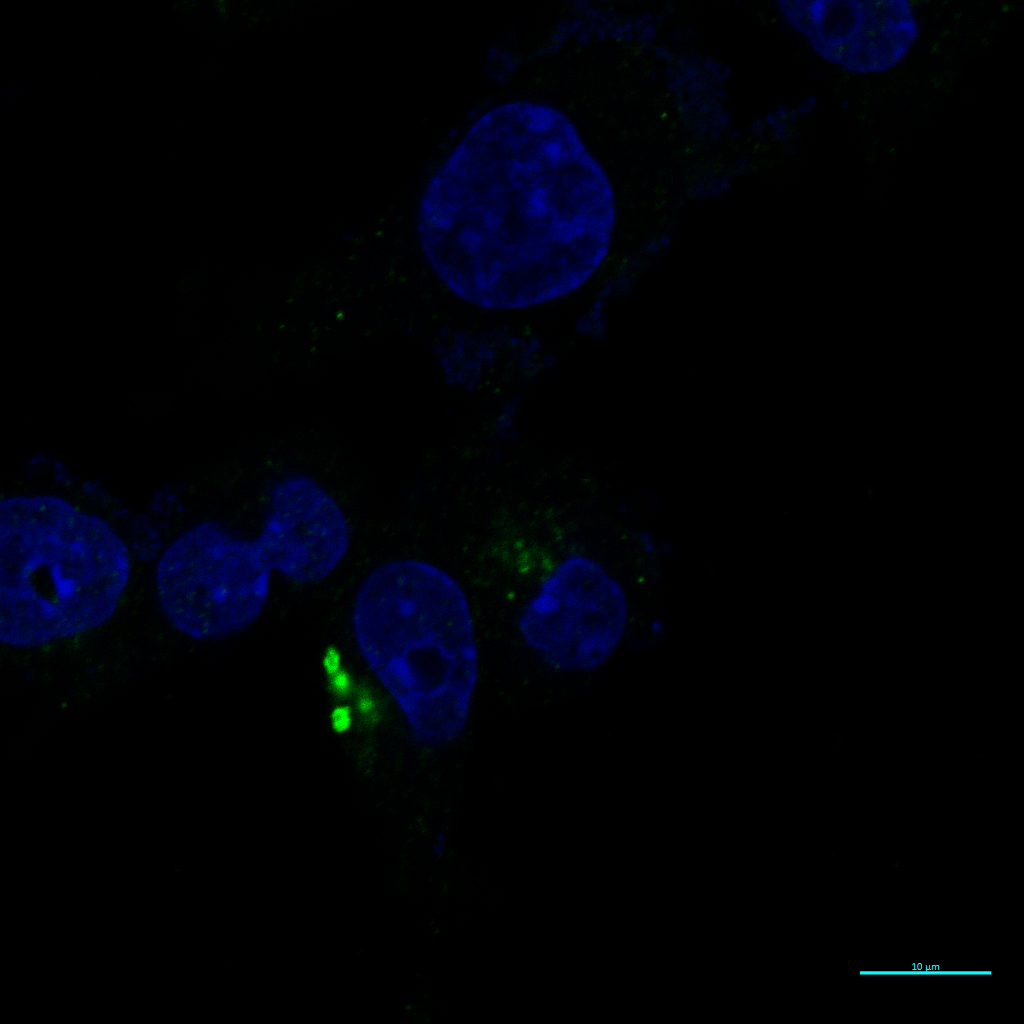

Supplement: Supplementary file 8 — Source data Fig. 7 [file 44318_2026_817_MOESM8_ESM.zip › Figure 7/7D/7D-2-Washout 3 h-WT.tif]

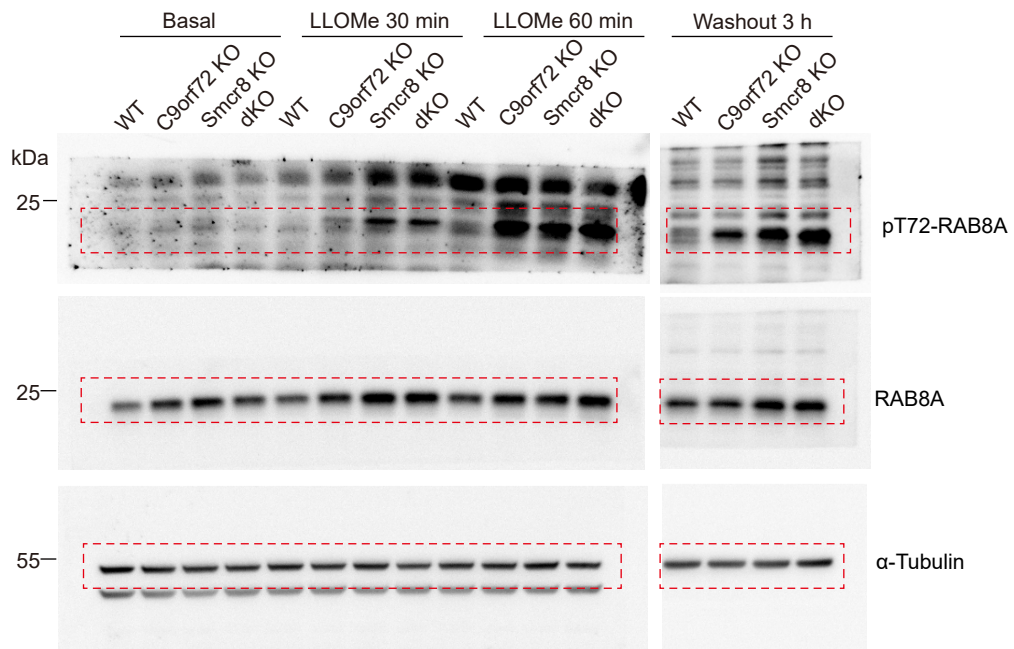

Supplement: Supplementary file 8 — Source data Fig. 7 [file 44318_2026_817_MOESM8_ESM.zip › Figure 7/7F/7F.pdf]

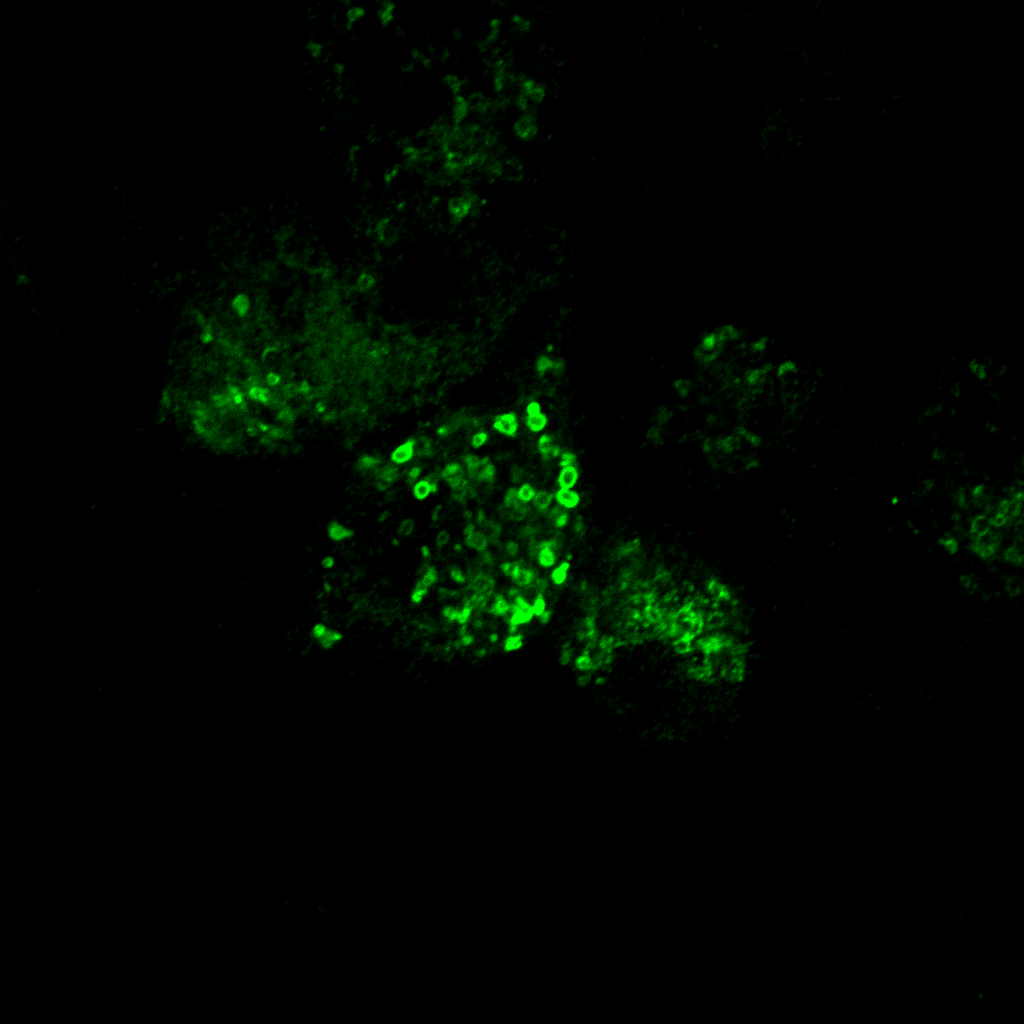

Supplement: Supplementary file 8 — Source data Fig. 7 [file 44318_2026_817_MOESM8_ESM.zip › Figure 7/7J/7J-dKO_expressing-GFP-RAB5-_GFP.tif]

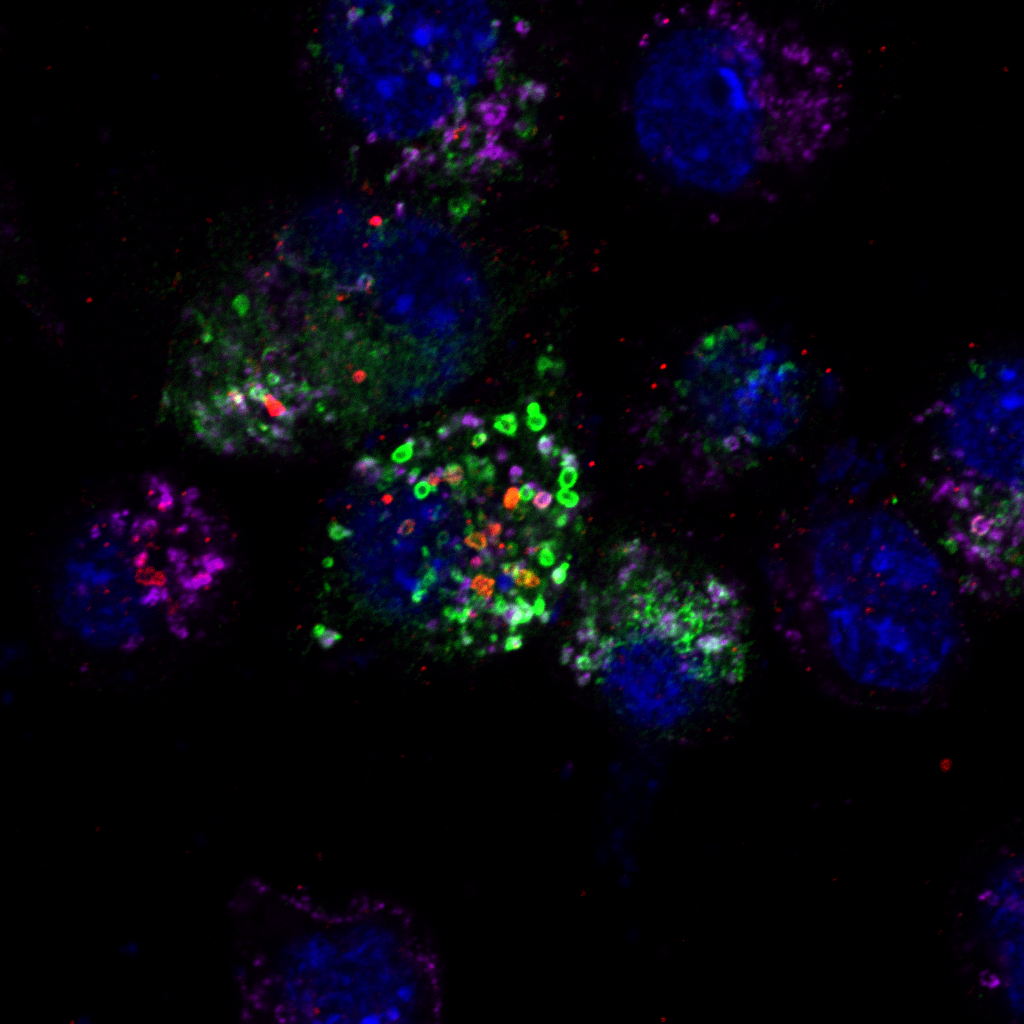

Supplement: Supplementary file 8 — Source data Fig. 7 [file 44318_2026_817_MOESM8_ESM.zip › Figure 7/7J/7J-dKO_expressing-GFP-RAB5-_Merge.tif]

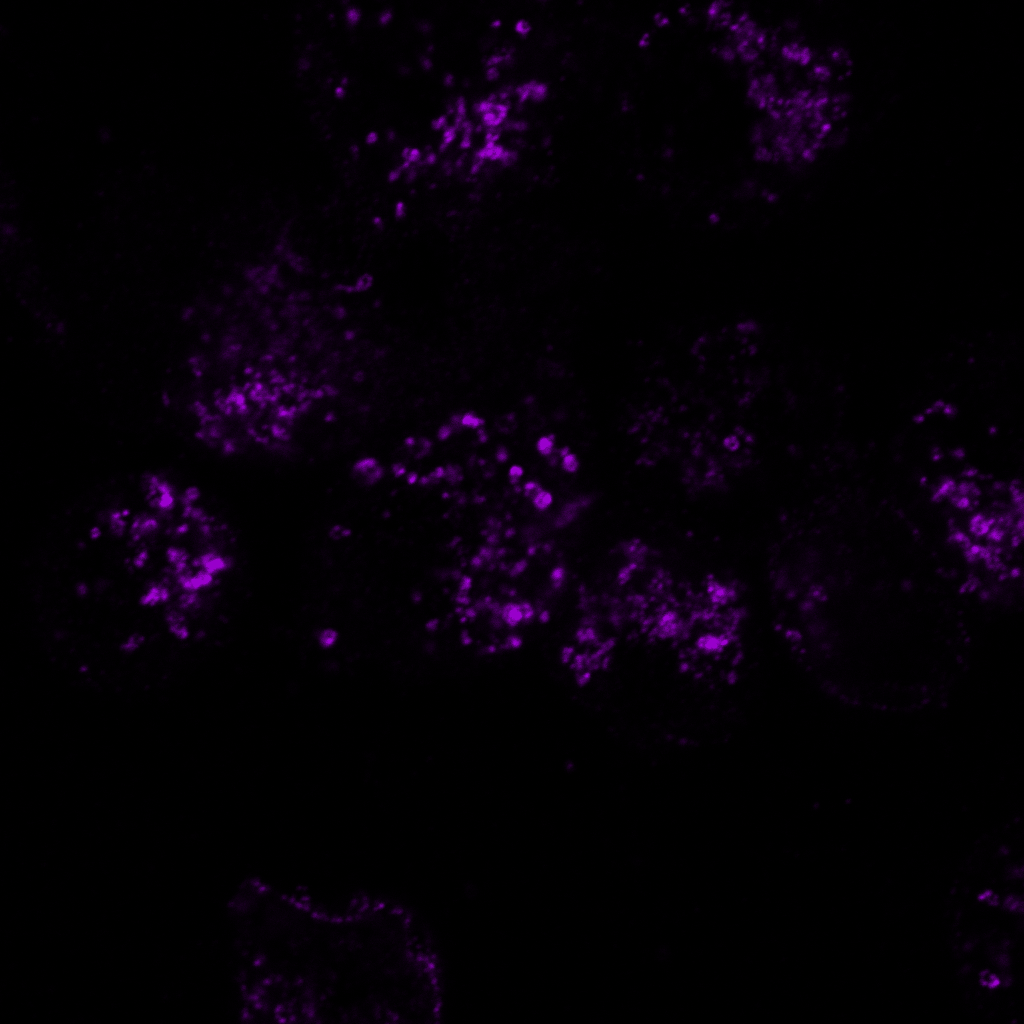

Supplement: Supplementary file 8 — Source data Fig. 7 [file 44318_2026_817_MOESM8_ESM.zip › Figure 7/7J/7J-dKO_expressing-GFP-RAB7-_LAMP1.tif]

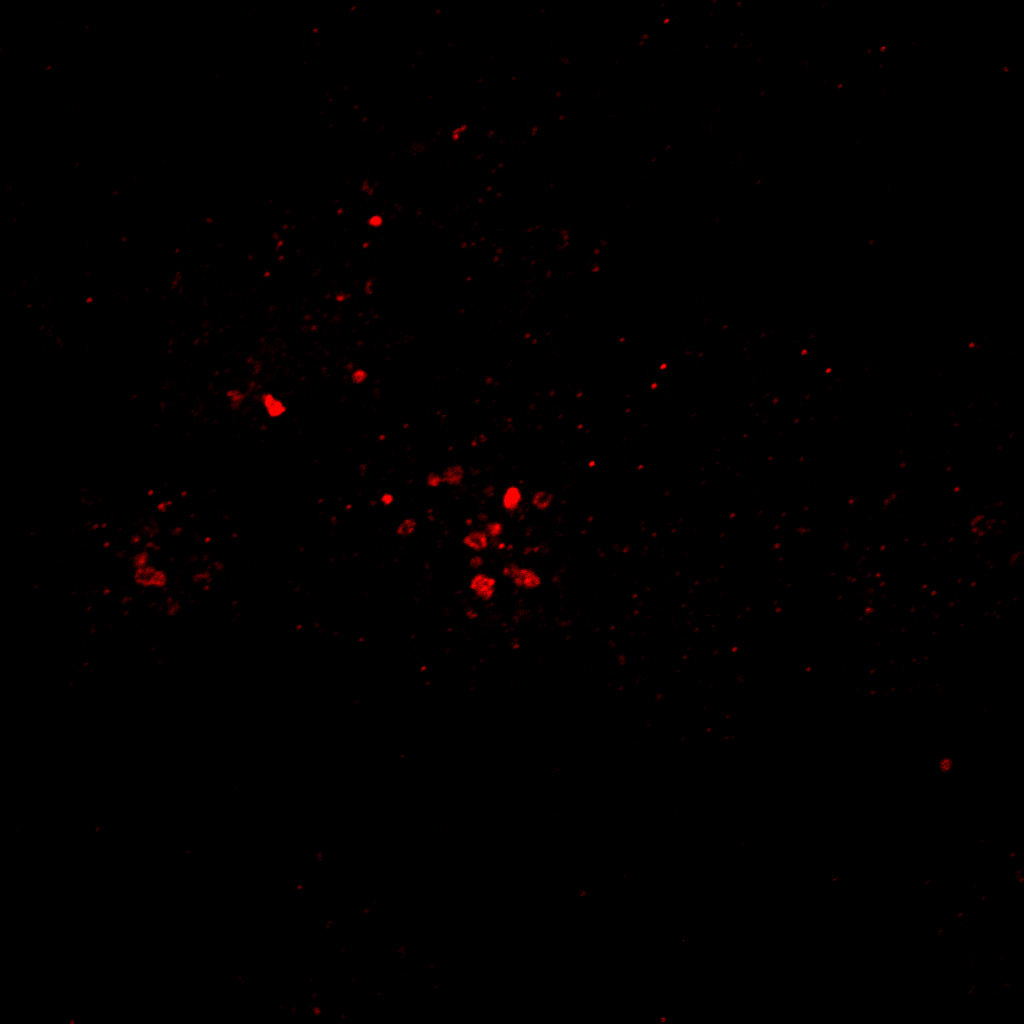

Supplement: Supplementary file 8 — Source data Fig. 7 [file 44318_2026_817_MOESM8_ESM.zip › Figure 7/7J/7J-dKO_expressing-GFP-RAB7-_pT72-RAB8A.tif]

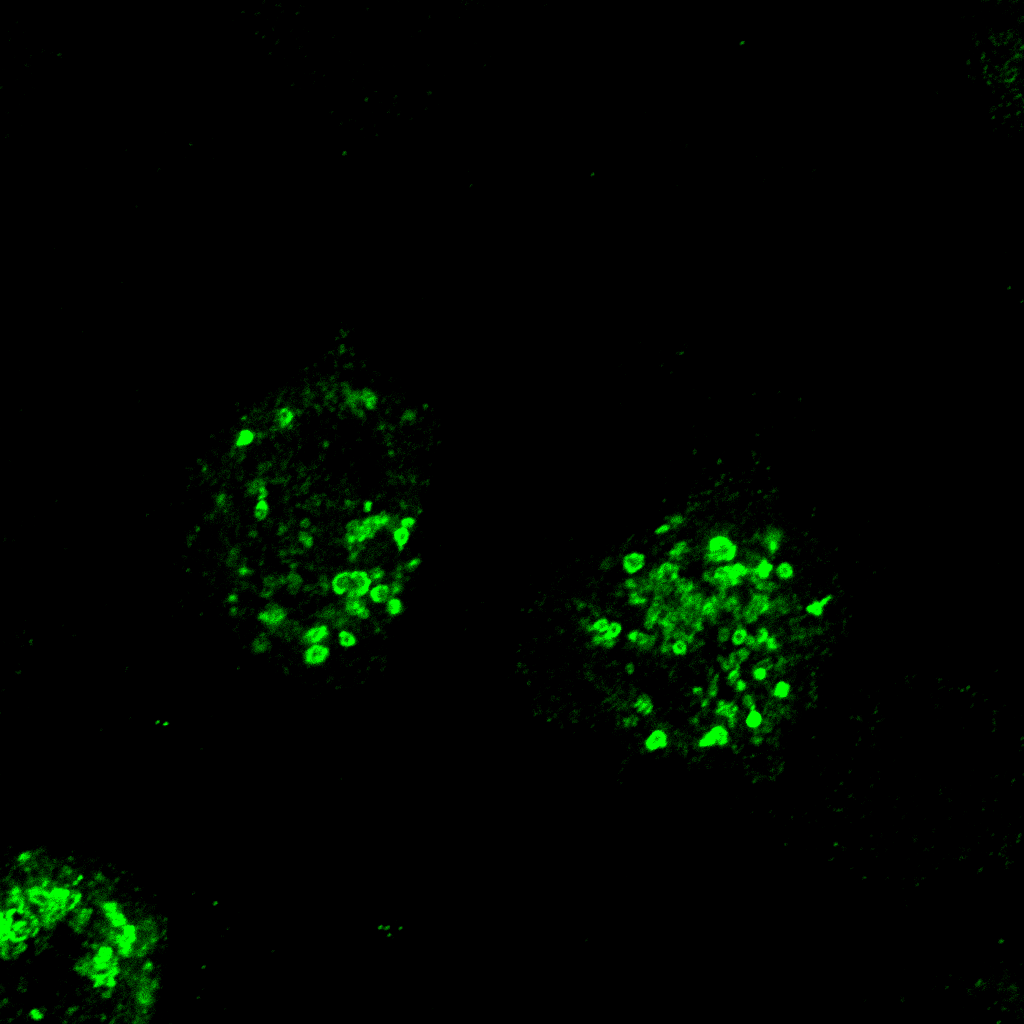

Supplement: Supplementary file 8 — Source data Fig. 7 [file 44318_2026_817_MOESM8_ESM.zip › Figure 7/7J/7J-WT_expressing-GFP-RAB5-_GFP.tif]

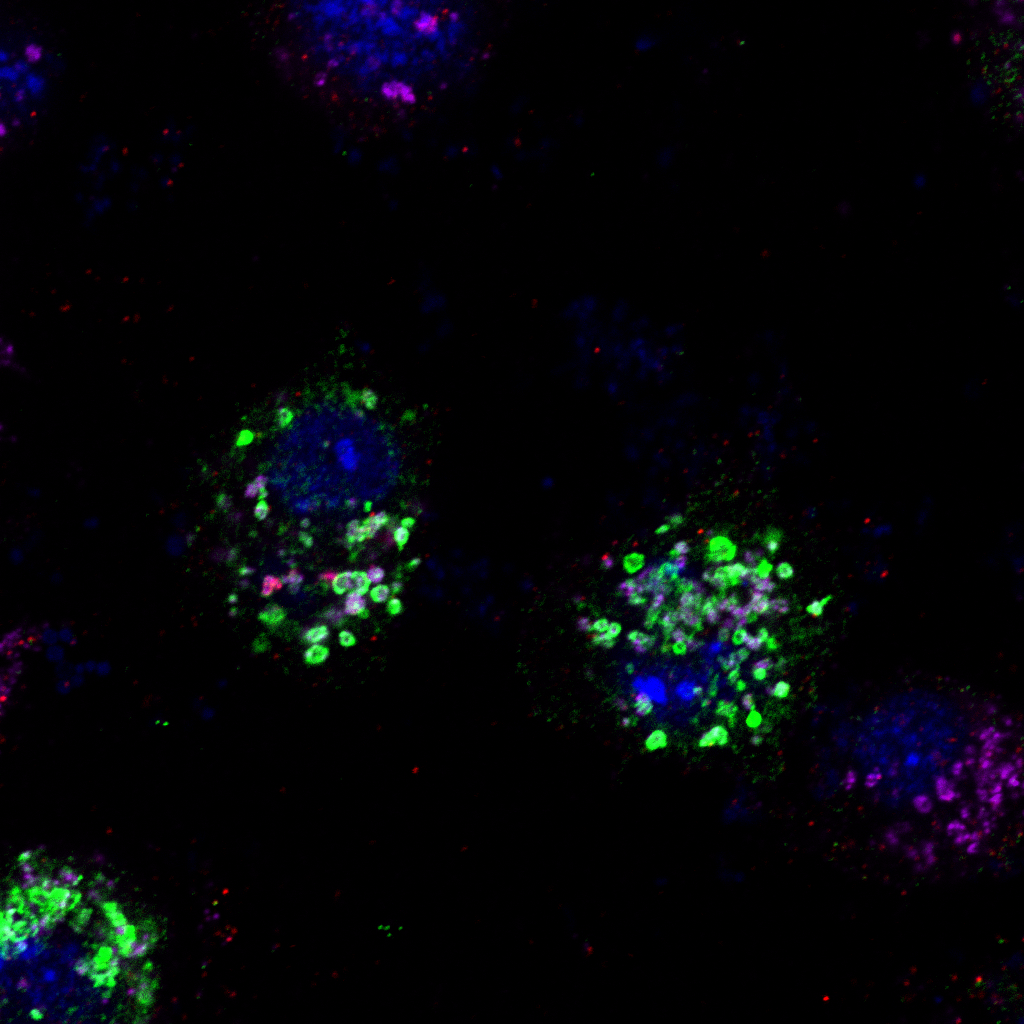

Supplement: Supplementary file 8 — Source data Fig. 7 [file 44318_2026_817_MOESM8_ESM.zip › Figure 7/7J/7J-WT_expressing-GFP-RAB5-_Merge.tif]

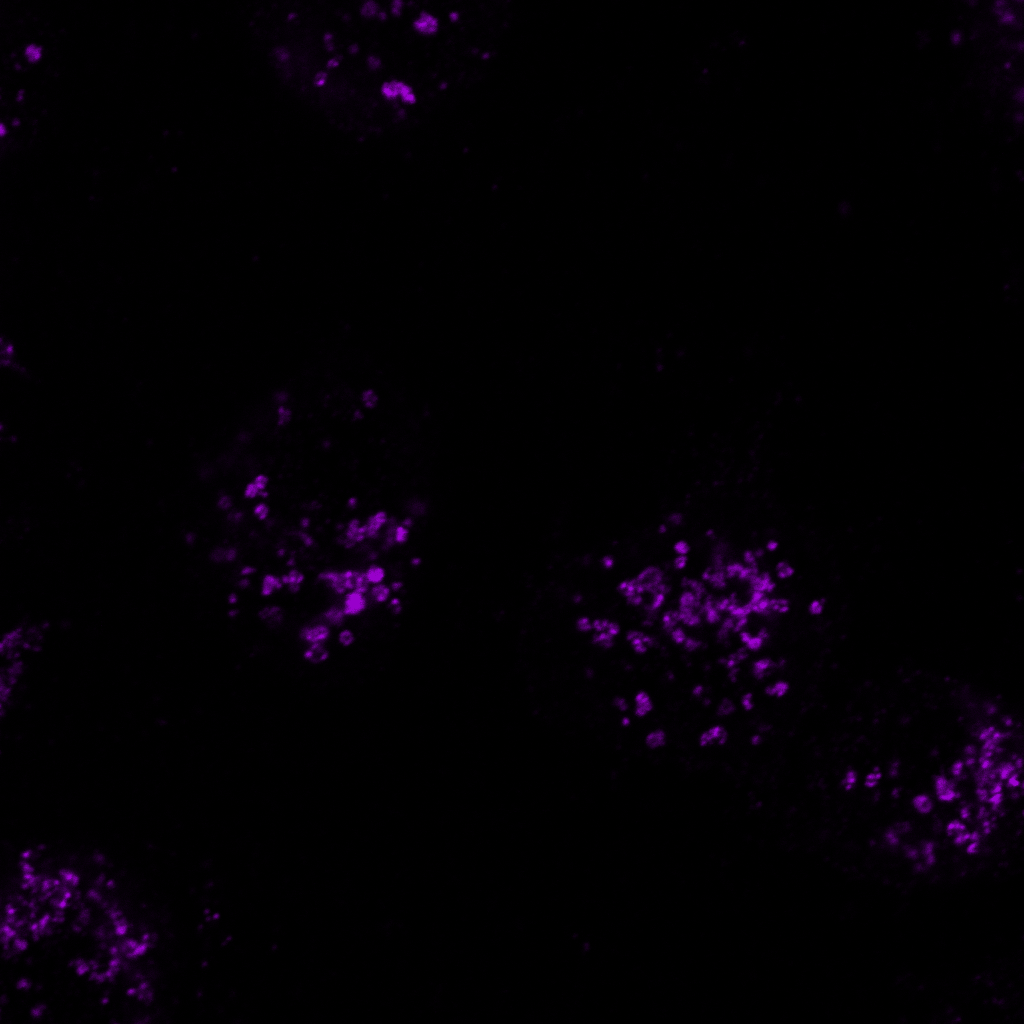

Supplement: Supplementary file 8 — Source data Fig. 7 [file 44318_2026_817_MOESM8_ESM.zip › Figure 7/7J/7J-WT_expressing-GFP-RAB7-_LAMP1.tif]

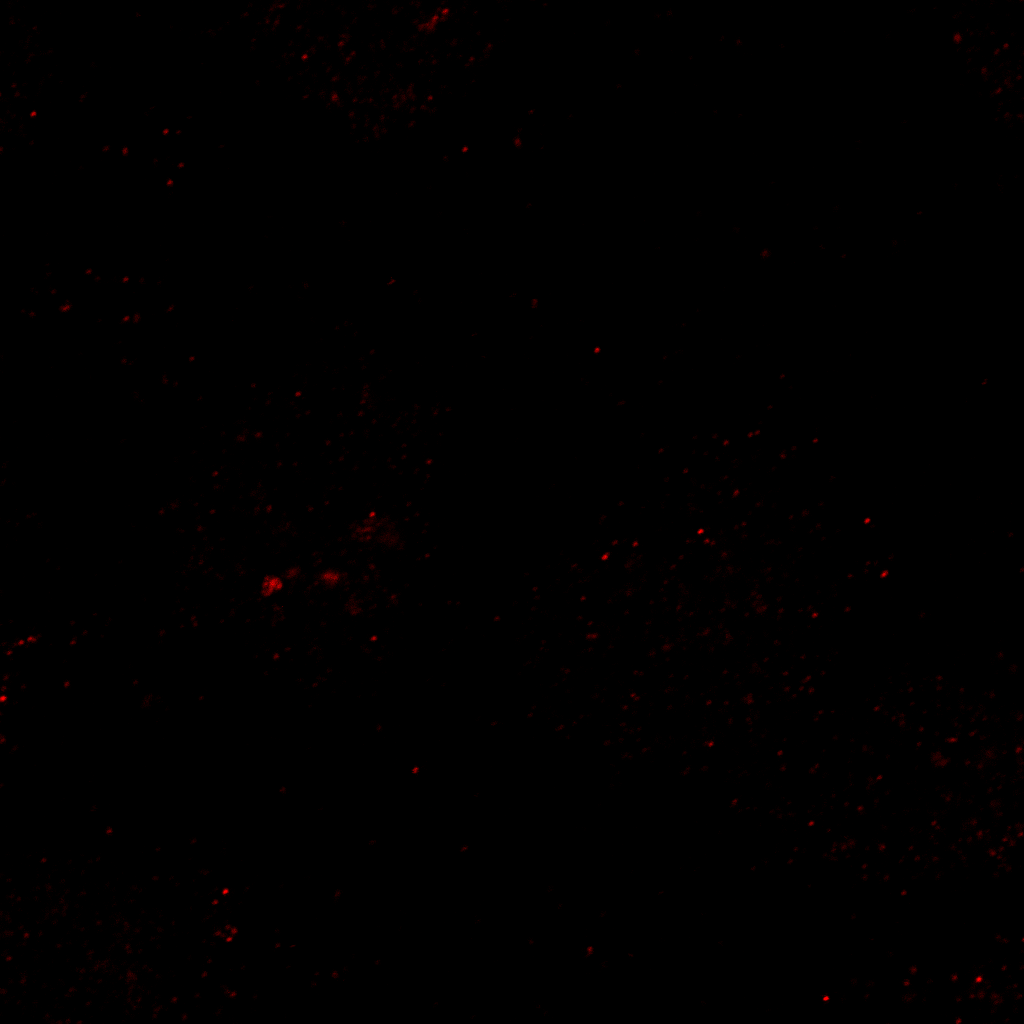

Supplement: Supplementary file 8 — Source data Fig. 7 [file 44318_2026_817_MOESM8_ESM.zip › Figure 7/7J/7J-WT_expressing-GFP-RAB7-_pT72-RAB8A.tif]

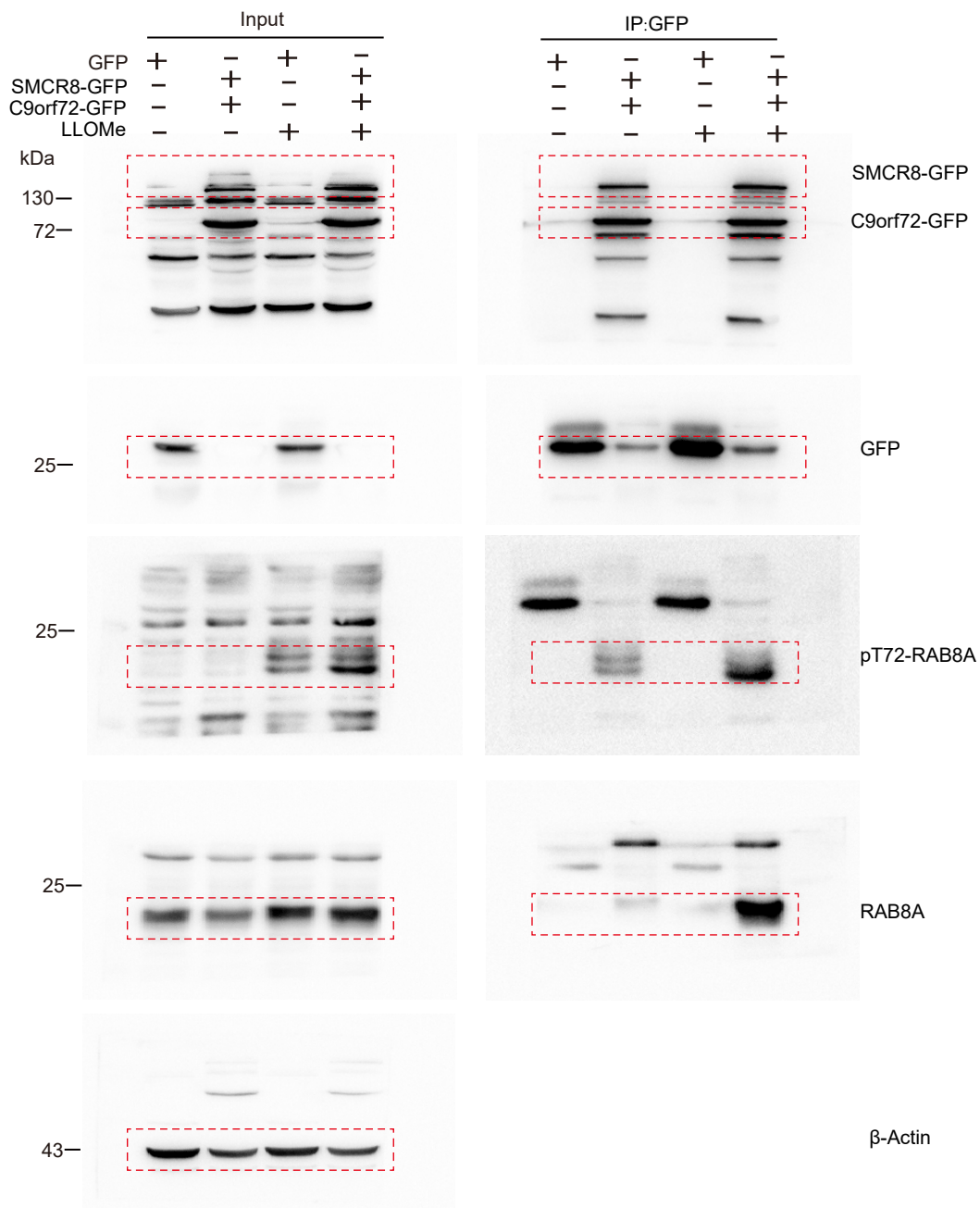

Supplement: Supplementary file 9 — Source data Fig. 8 [file 44318_2026_817_MOESM9_ESM.zip › 8A/8A.pdf]

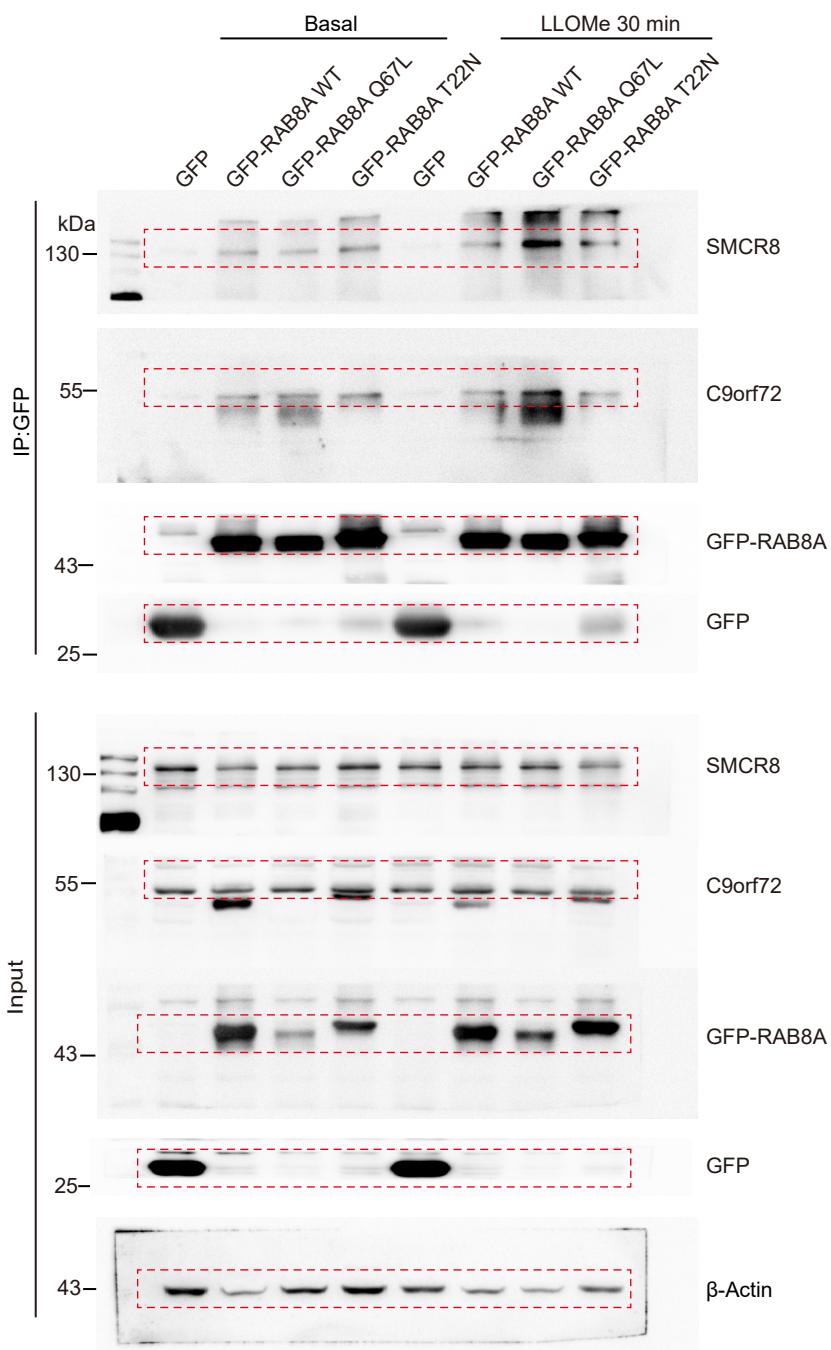

Supplement: Supplementary file 9 — Source data Fig. 8 [file 44318_2026_817_MOESM9_ESM.zip › 8C/8C.pdf]

GST-OCRL

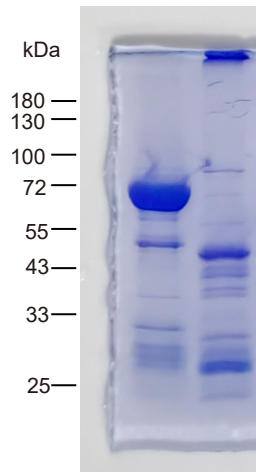

Supplement: Supplementary file 9 — Source data Fig. 8 [file 44318_2026_817_MOESM9_ESM.zip › 8E/8E.pdf]

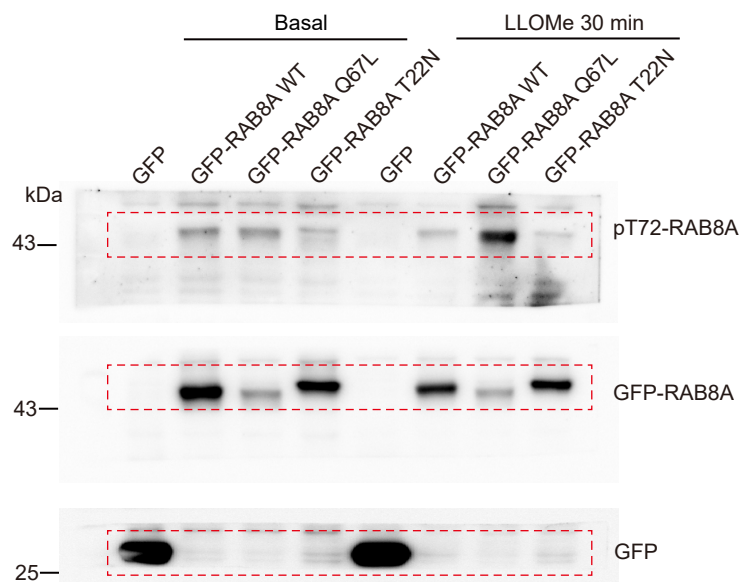

Supplement: Supplementary file 9 — Source data Fig. 8 [file 44318_2026_817_MOESM9_ESM.zip › 8G/8G.pdf]

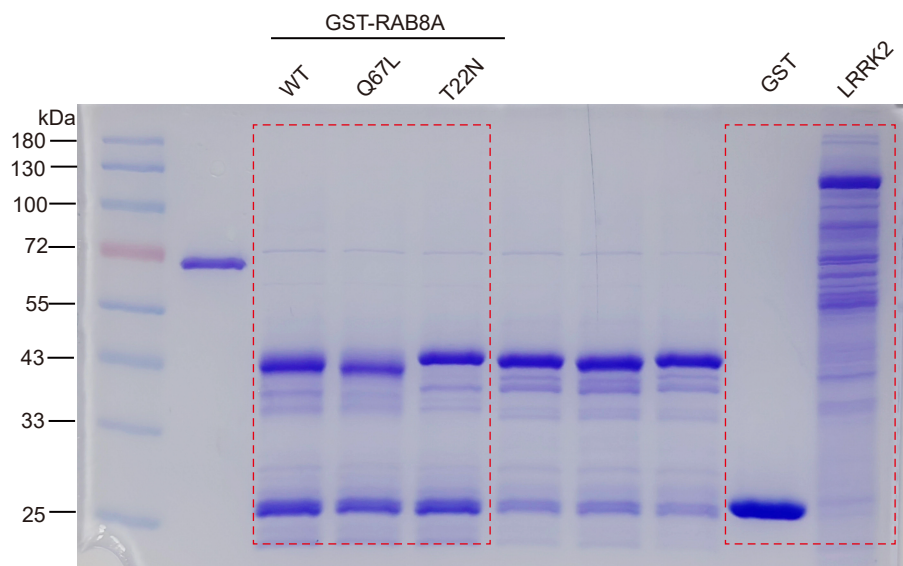

Supplement: Supplementary file 9 — Source data Fig. 8 [file 44318_2026_817_MOESM9_ESM.zip › 8I/8I.pdf]

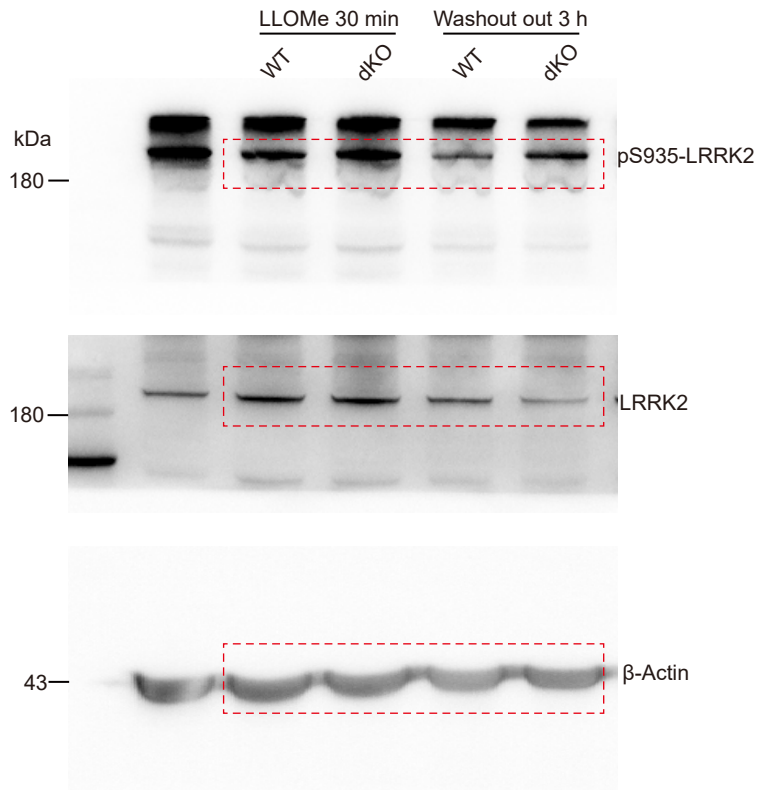

Supplement: Supplementary file 9 — Source data Fig. 8 [file 44318_2026_817_MOESM9_ESM.zip › 8K/8K.pdf]

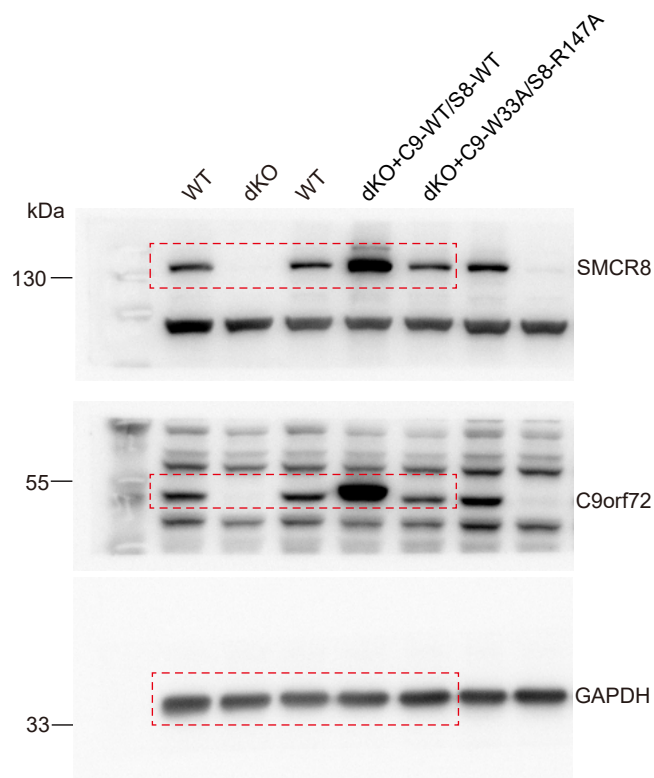

Supplement: Supplementary file 10 — Source data Fig. 9 [file 44318_2026_817_MOESM10_ESM.zip › 9B/9B.pdf]

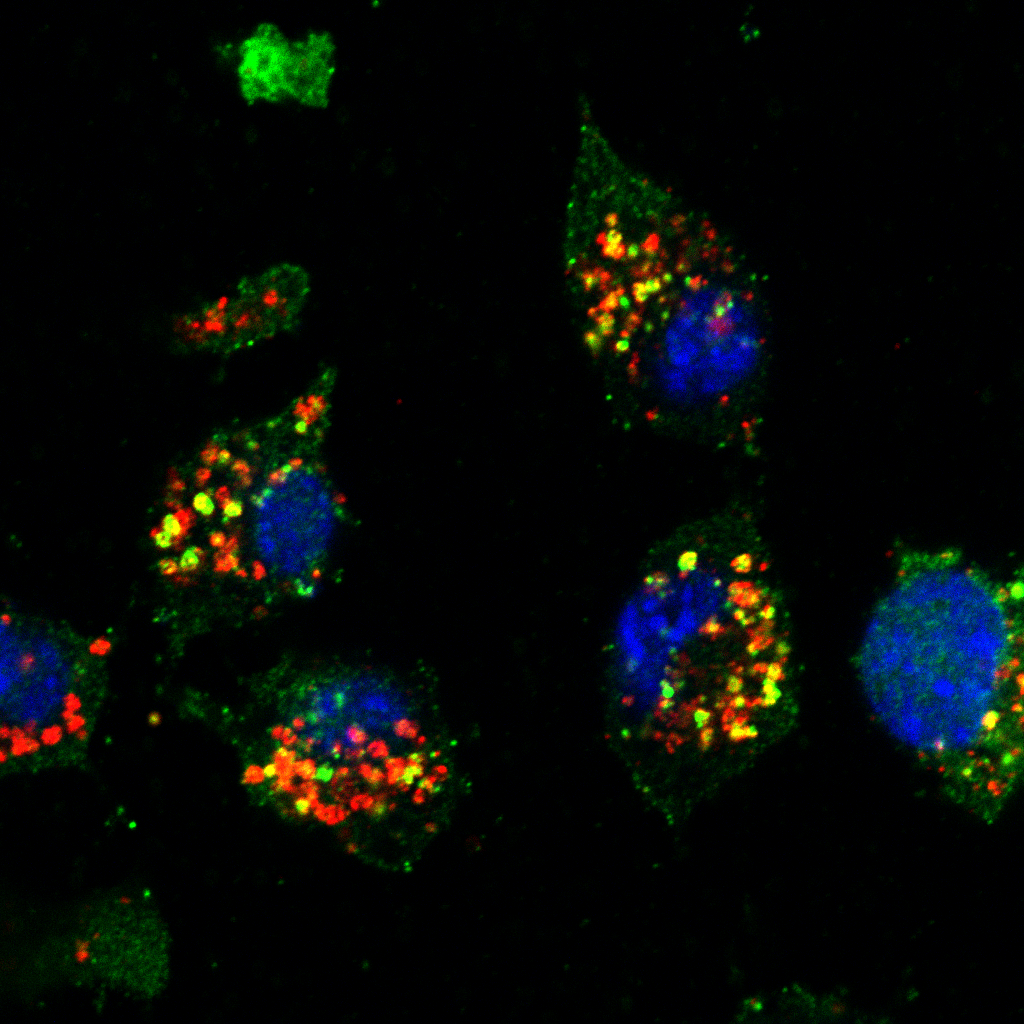

Supplement: Supplementary file 10 — Source data Fig. 9 [file 44318_2026_817_MOESM10_ESM.zip › 9C/9C-1-LLOMe 30 min-dKO.tif]

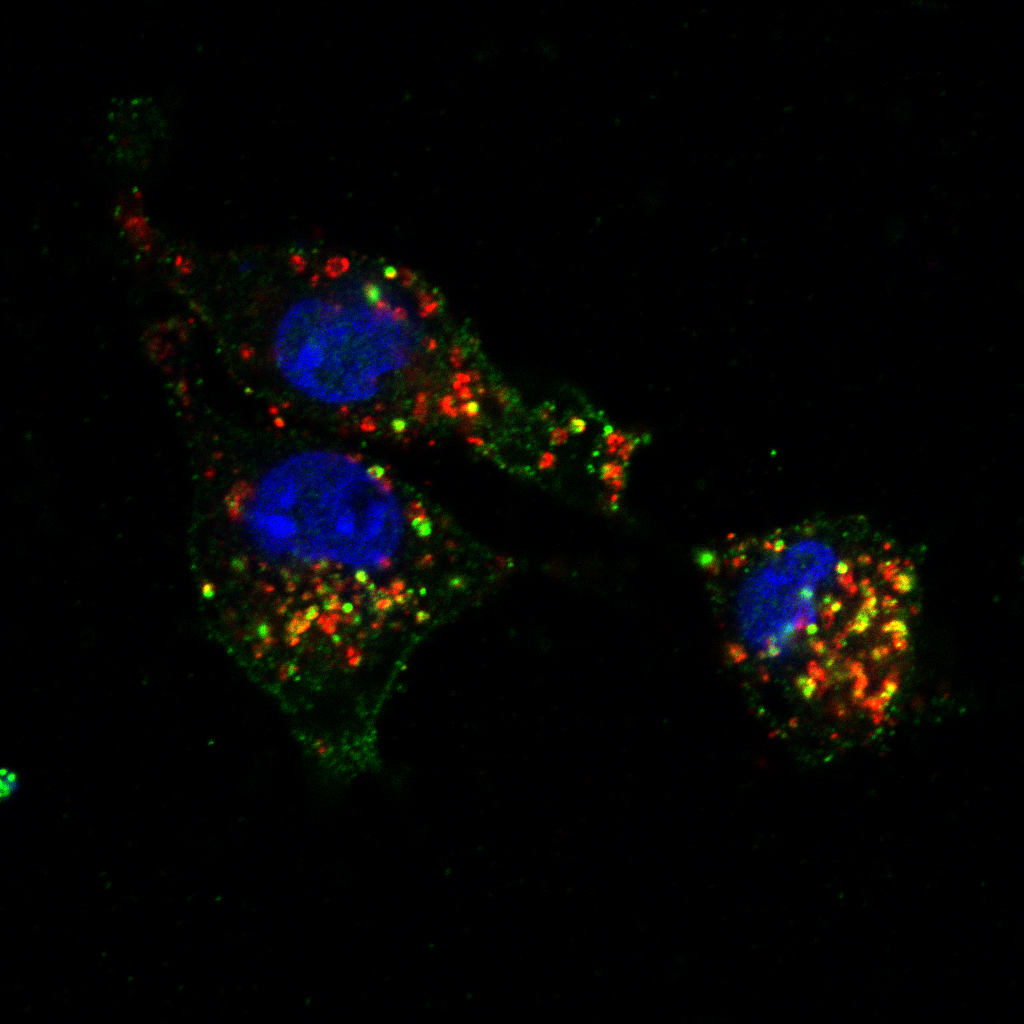

Supplement: Supplementary file 10 — Source data Fig. 9 [file 44318_2026_817_MOESM10_ESM.zip › 9C/9C-1-LLOMe 30 min-dKO_expressing-C9-W33A, S8-R147A.tif]

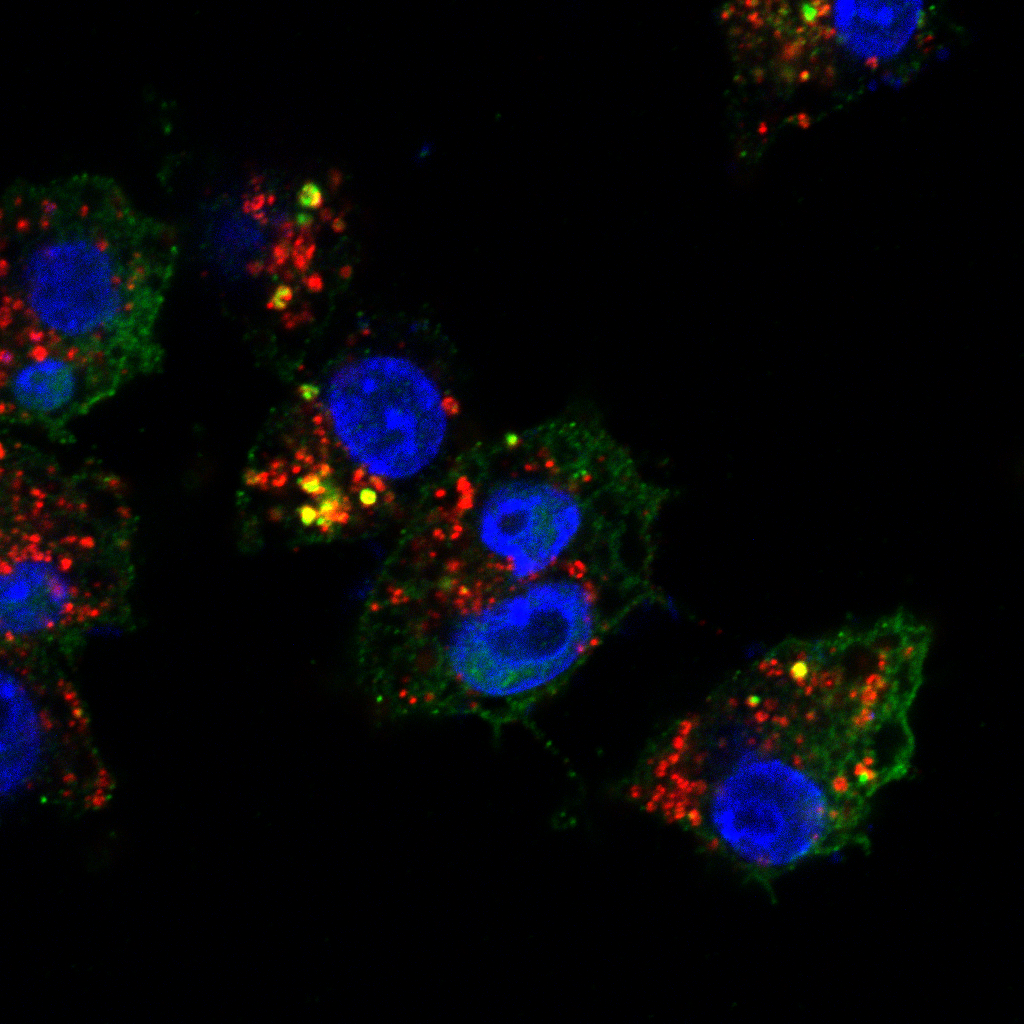

Supplement: Supplementary file 10 — Source data Fig. 9 [file 44318_2026_817_MOESM10_ESM.zip › 9C/9C-1-LLOMe 30 min-dKO_expressing-C9-WT, S8-WT.tif]

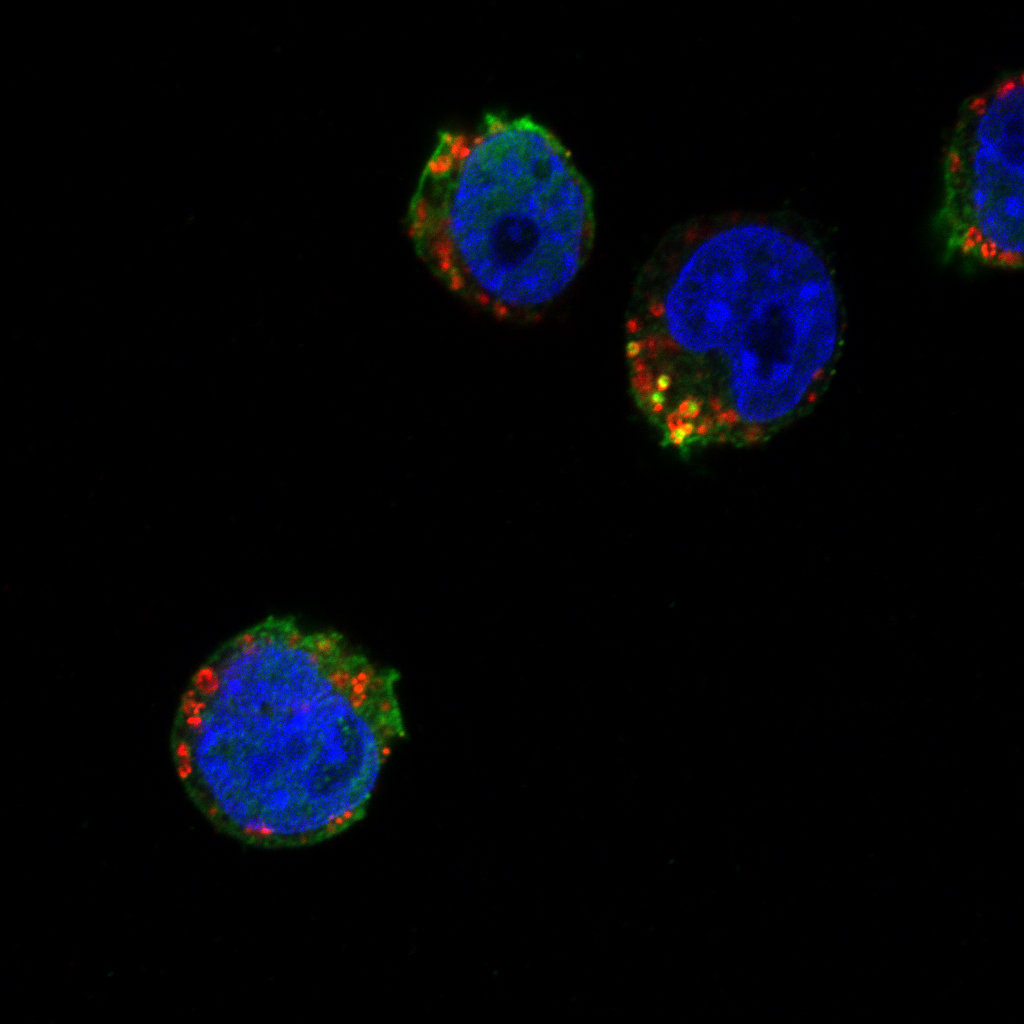

Supplement: Supplementary file 10 — Source data Fig. 9 [file 44318_2026_817_MOESM10_ESM.zip › 9C/9C-1-LLOMe 30 min-WT.tif]

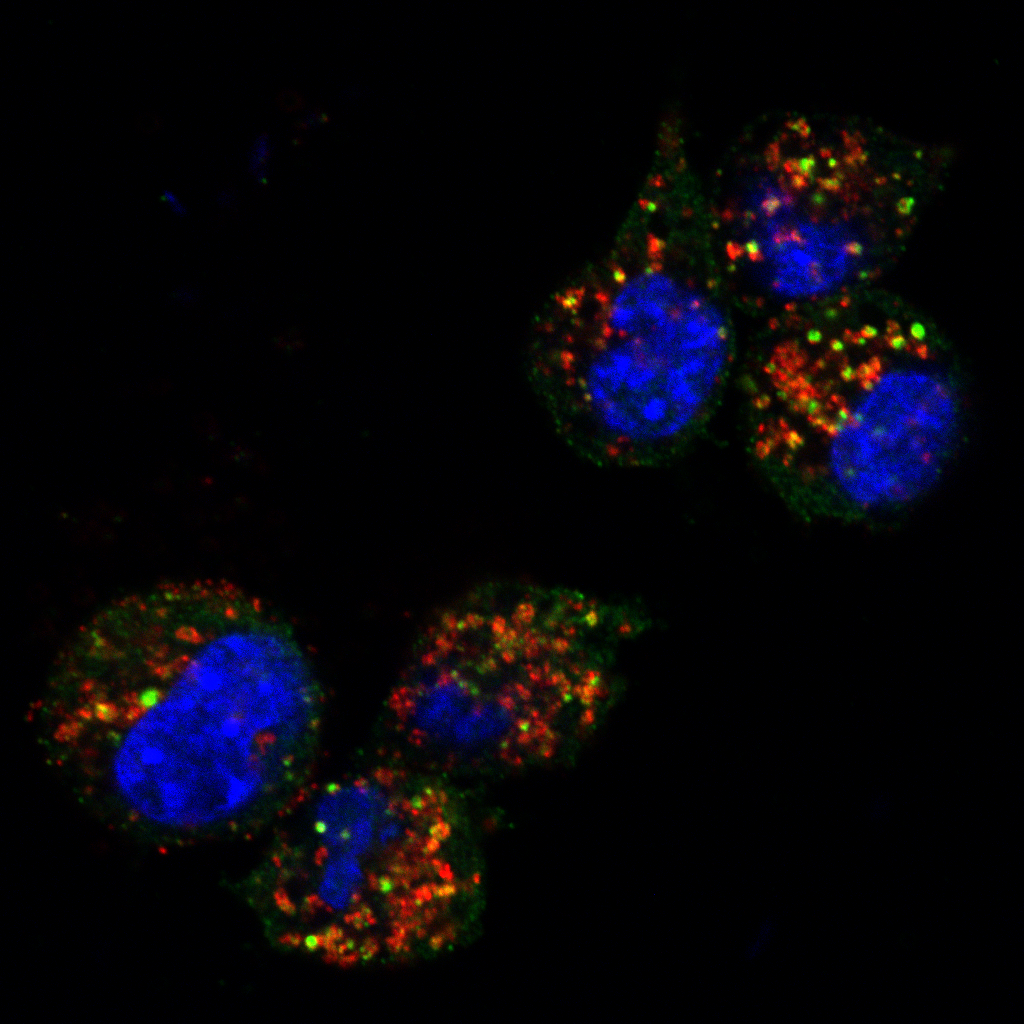

Supplement: Supplementary file 10 — Source data Fig. 9 [file 44318_2026_817_MOESM10_ESM.zip › 9C/9C-1-Washout 3 h-dKO.tif]

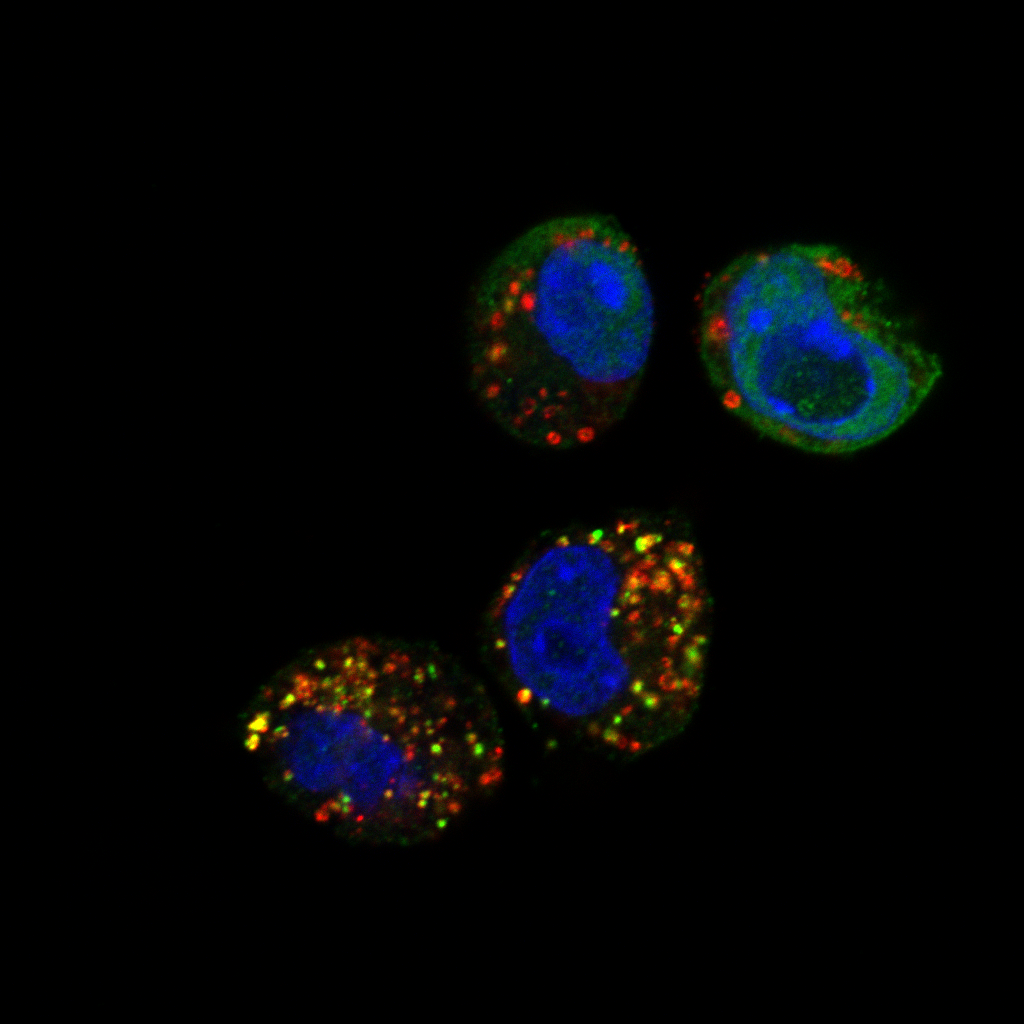

Supplement: Supplementary file 10 — Source data Fig. 9 [file 44318_2026_817_MOESM10_ESM.zip › 9C/9C-1-Washout 3 h-dKO_expressing-C9-W33A, S8-R147A.tif]

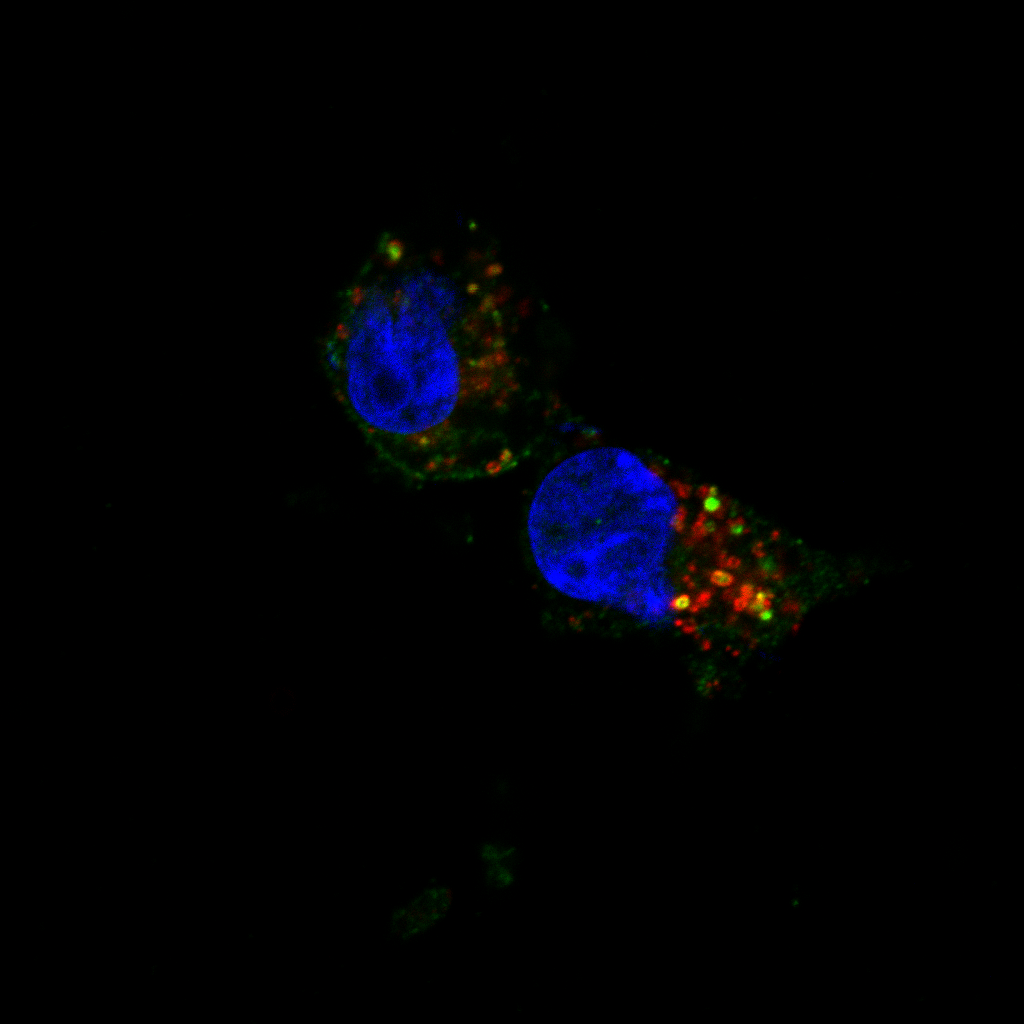

Supplement: Supplementary file 10 — Source data Fig. 9 [file 44318_2026_817_MOESM10_ESM.zip › 9C/9C-1-Washout 3 h-dKO_expressing-C9-WT, S8-WT.tif]

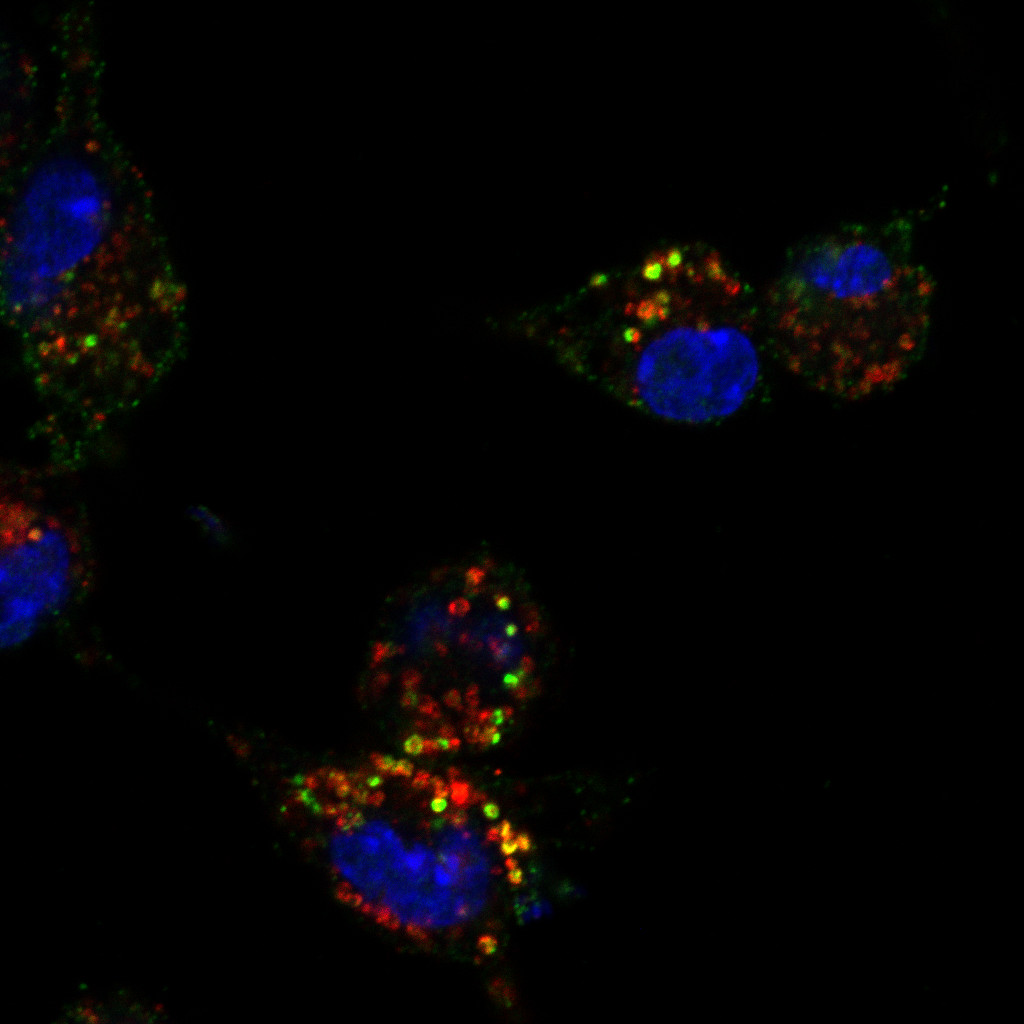

Supplement: Supplementary file 10 — Source data Fig. 9 [file 44318_2026_817_MOESM10_ESM.zip › 9C/9C-1-Washout 3 h-WT.tif]

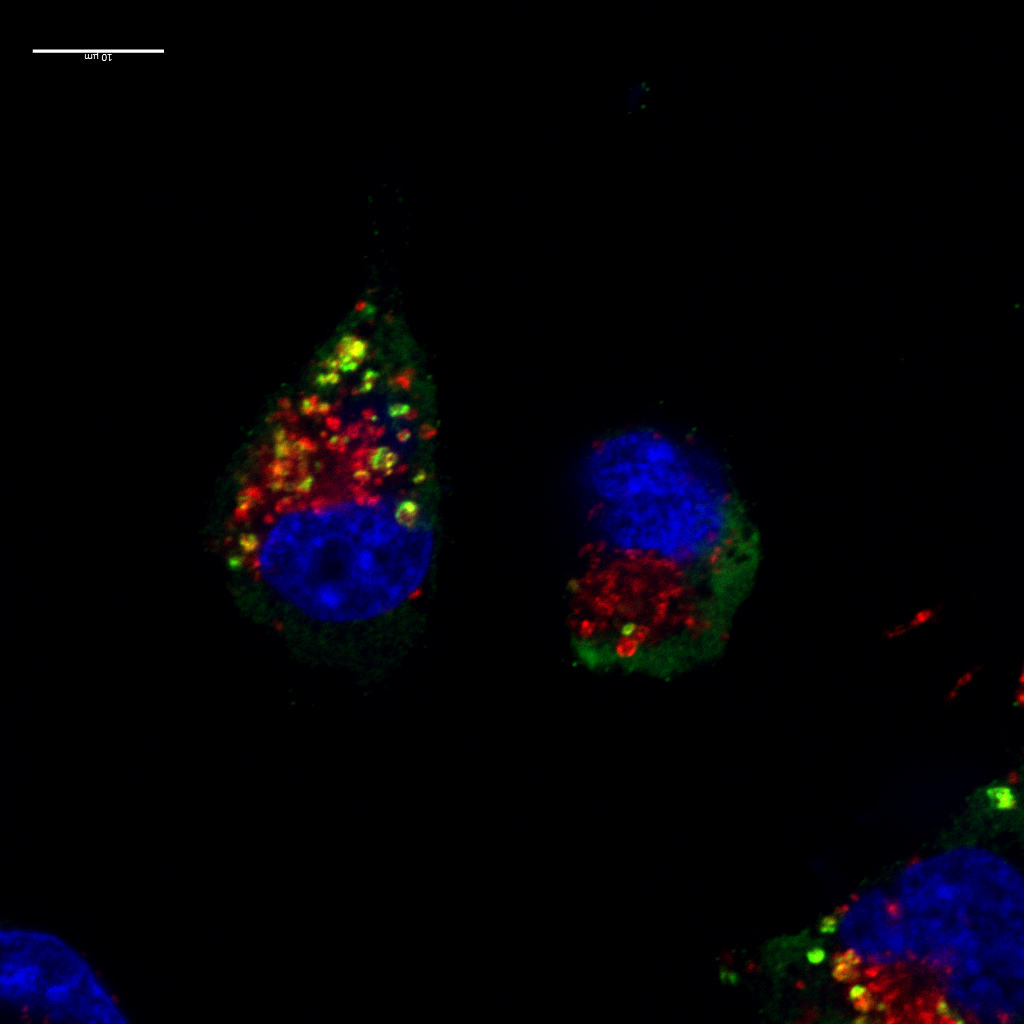

Supplement: Supplementary file 10 — Source data Fig. 9 [file 44318_2026_817_MOESM10_ESM.zip › 9C/9C-1-Washout 6 h-dKO.tif]

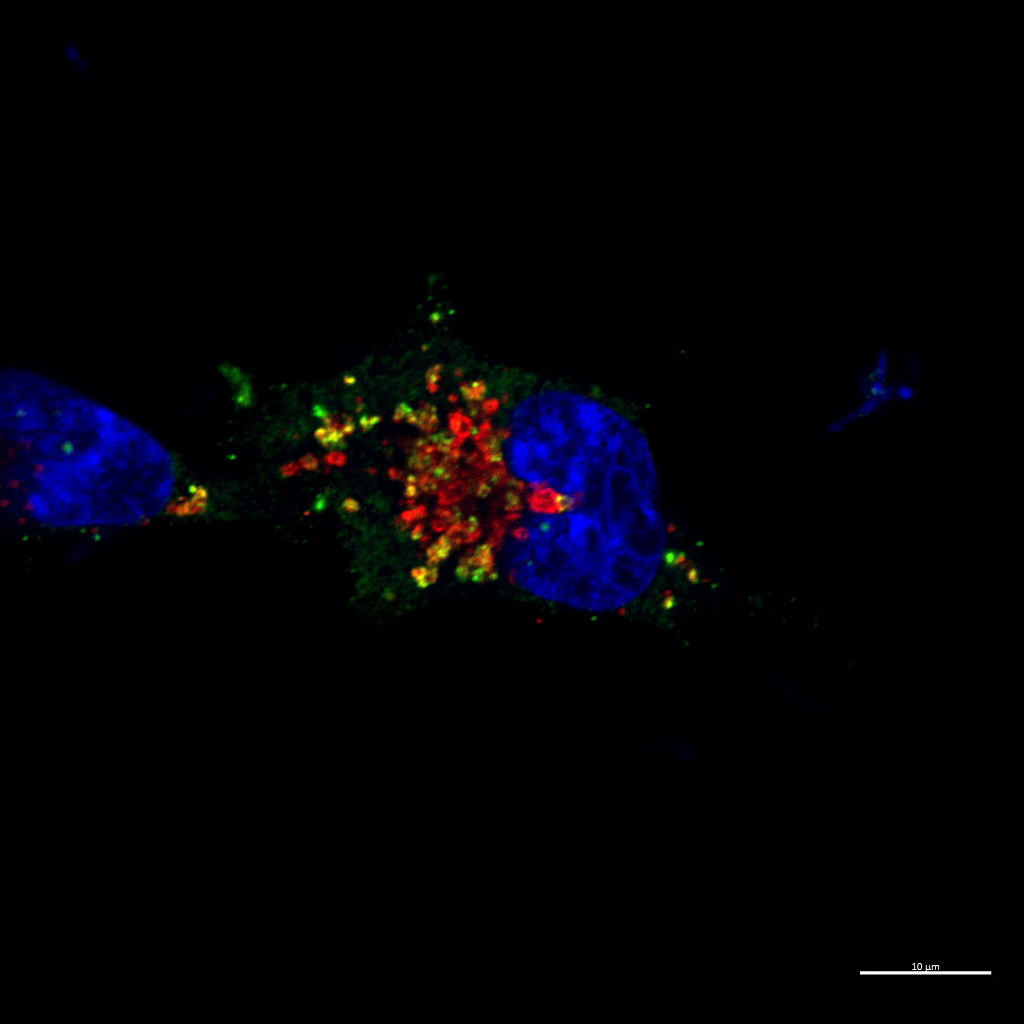

Supplement: Supplementary file 10 — Source data Fig. 9 [file 44318_2026_817_MOESM10_ESM.zip › 9C/9C-1-Washout 6 h-dKO_expressing-C9-W33A, S8-R147A.tif]

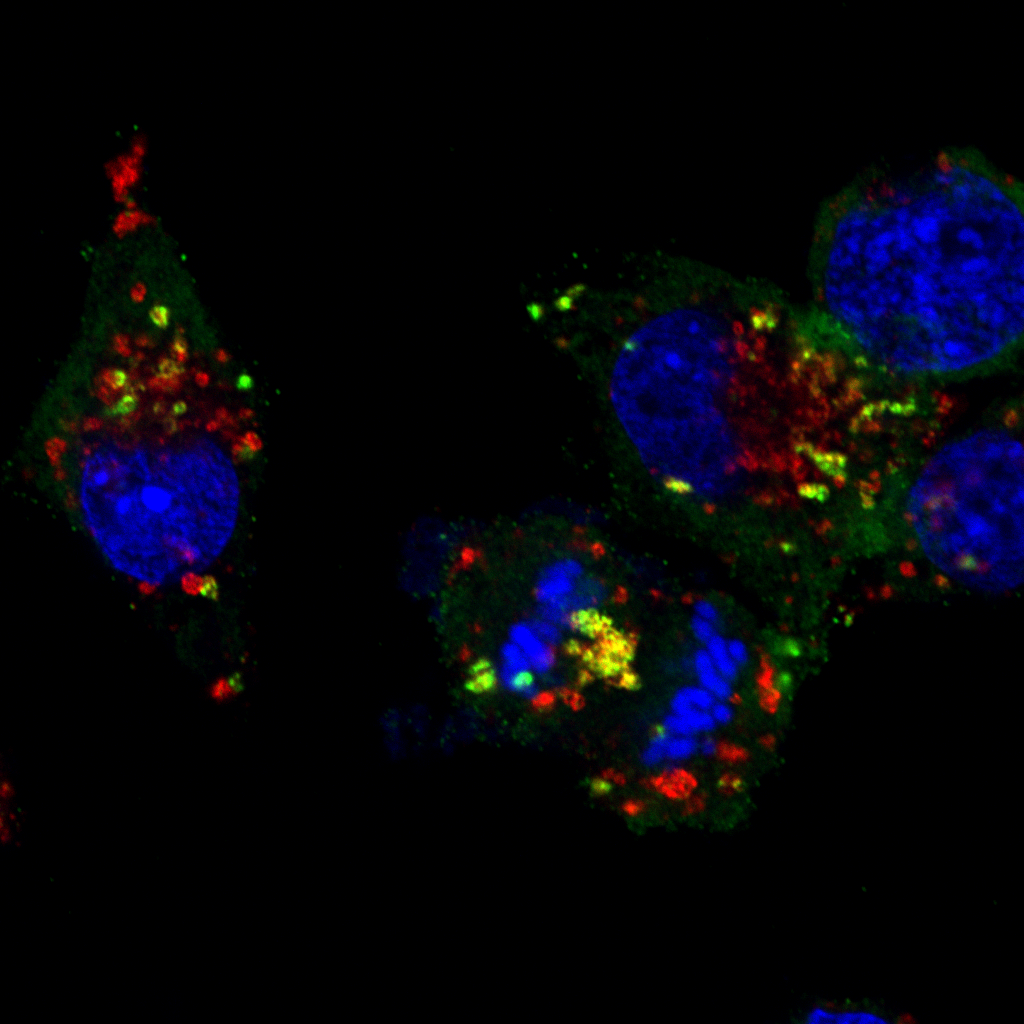

Supplement: Supplementary file 10 — Source data Fig. 9 [file 44318_2026_817_MOESM10_ESM.zip › 9C/9C-1-Washout 6 h-dKO_expressing-C9-WT, S8-WT.tif]

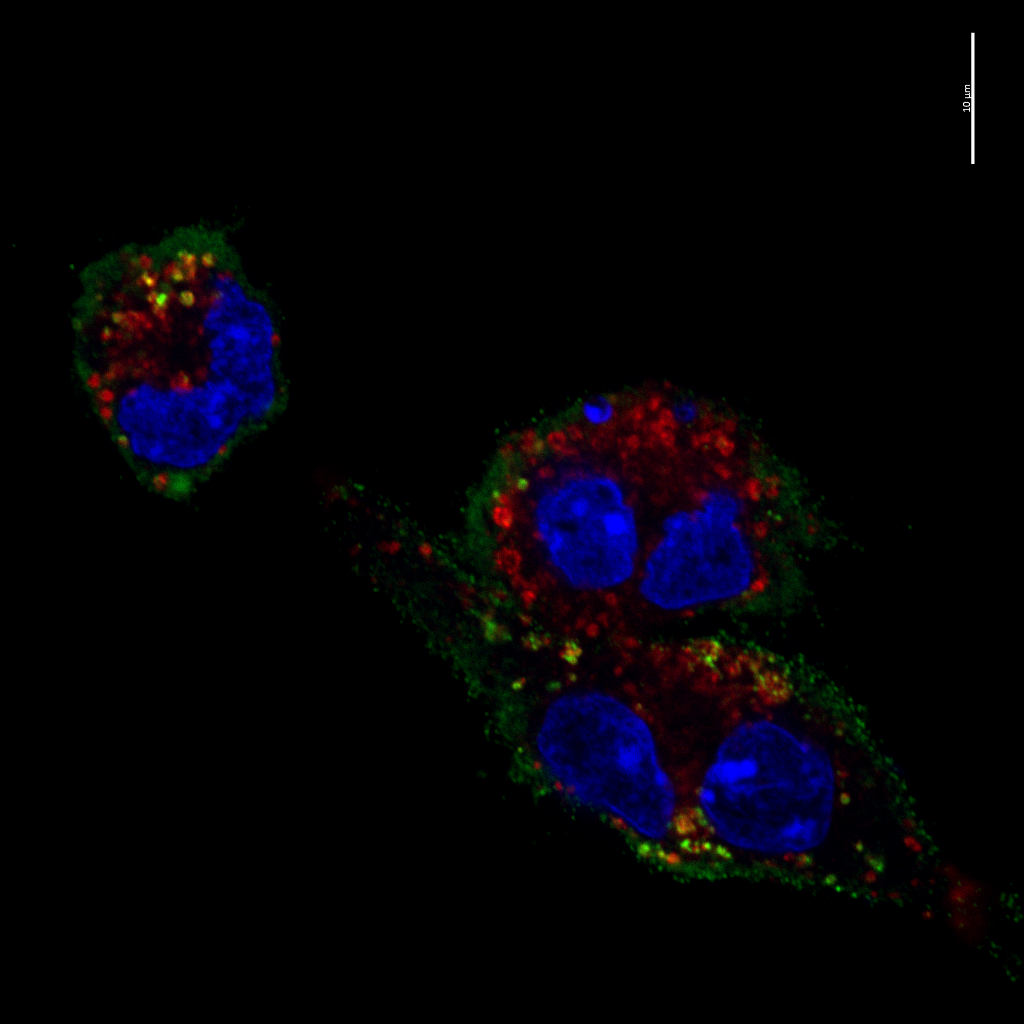

Supplement: Supplementary file 10 — Source data Fig. 9 [file 44318_2026_817_MOESM10_ESM.zip › 9C/9C-1-Washout 6 h-WT.tif]

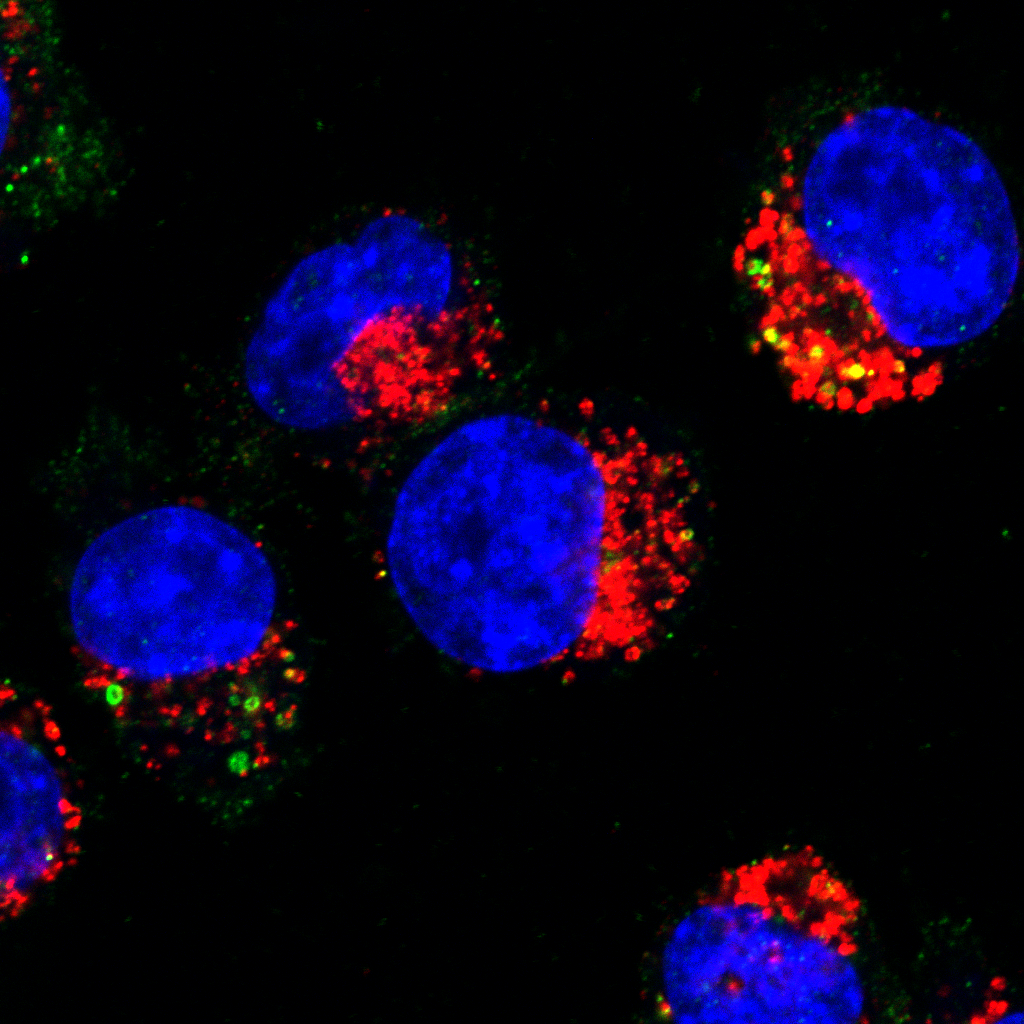

Supplement: Supplementary file 10 — Source data Fig. 9 [file 44318_2026_817_MOESM10_ESM.zip › 9E/9E-1-LLOMe 10 min-dKO.tif]

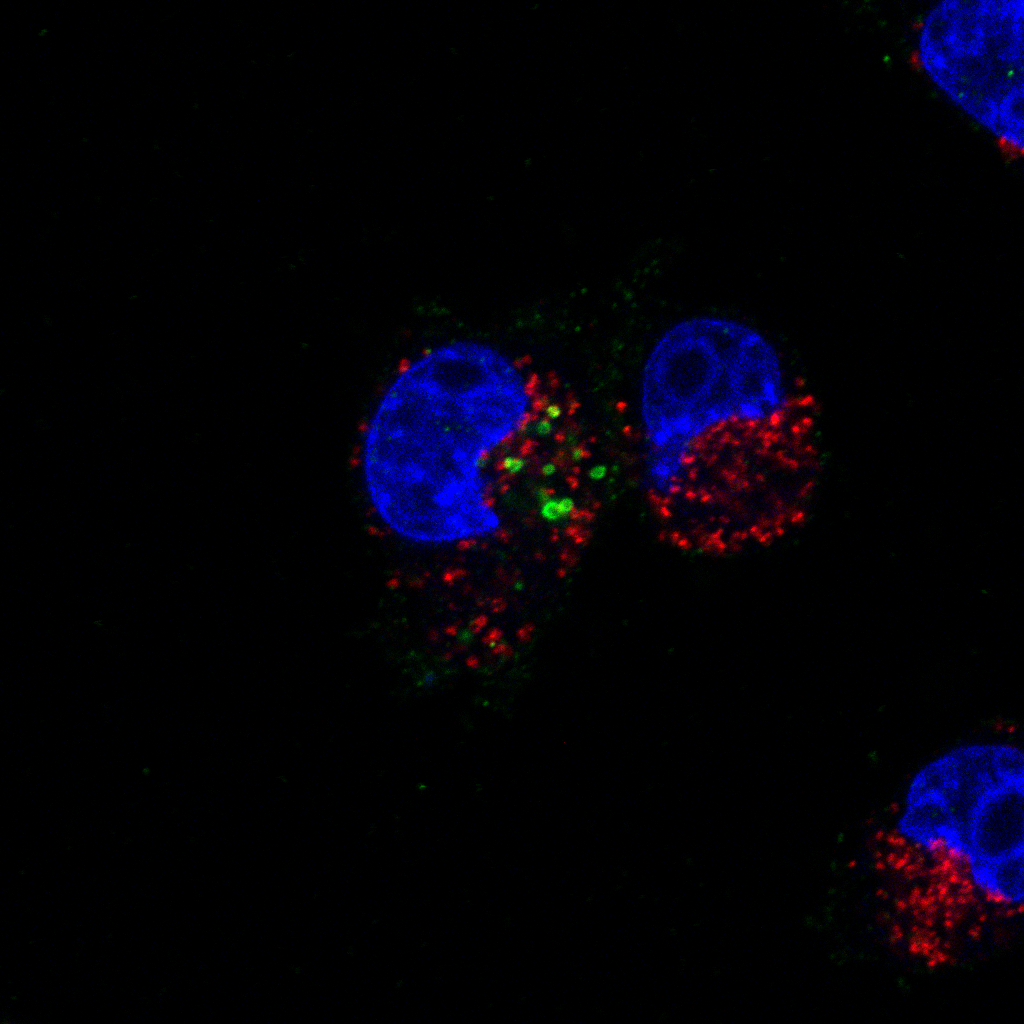

Supplement: Supplementary file 10 — Source data Fig. 9 [file 44318_2026_817_MOESM10_ESM.zip › 9E/9E-1-LLOMe 10 min-dKO_expressing-C9-W33A, S8-R147A.tif]

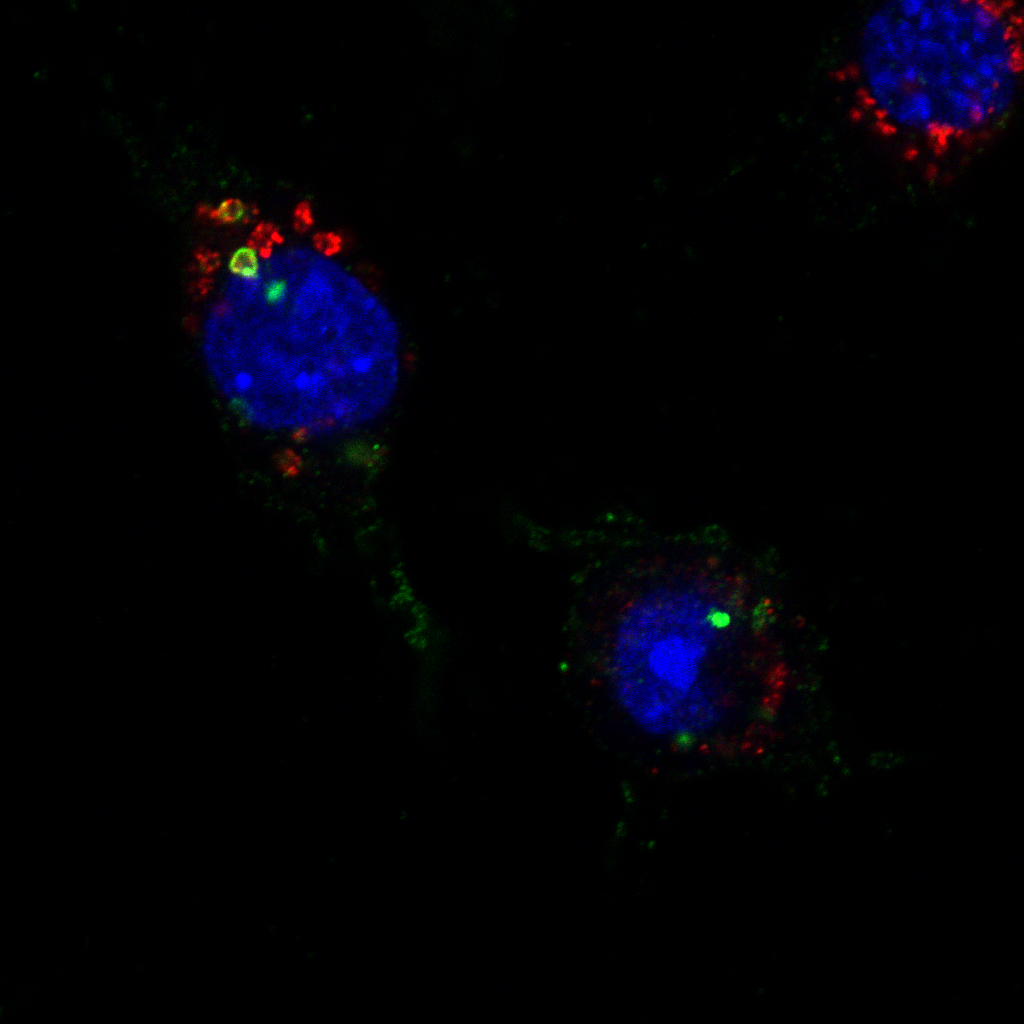

Supplement: Supplementary file 10 — Source data Fig. 9 [file 44318_2026_817_MOESM10_ESM.zip › 9E/9E-1-LLOMe 10 min-dKO_expressing-C9-WT, S8-WT.tif]

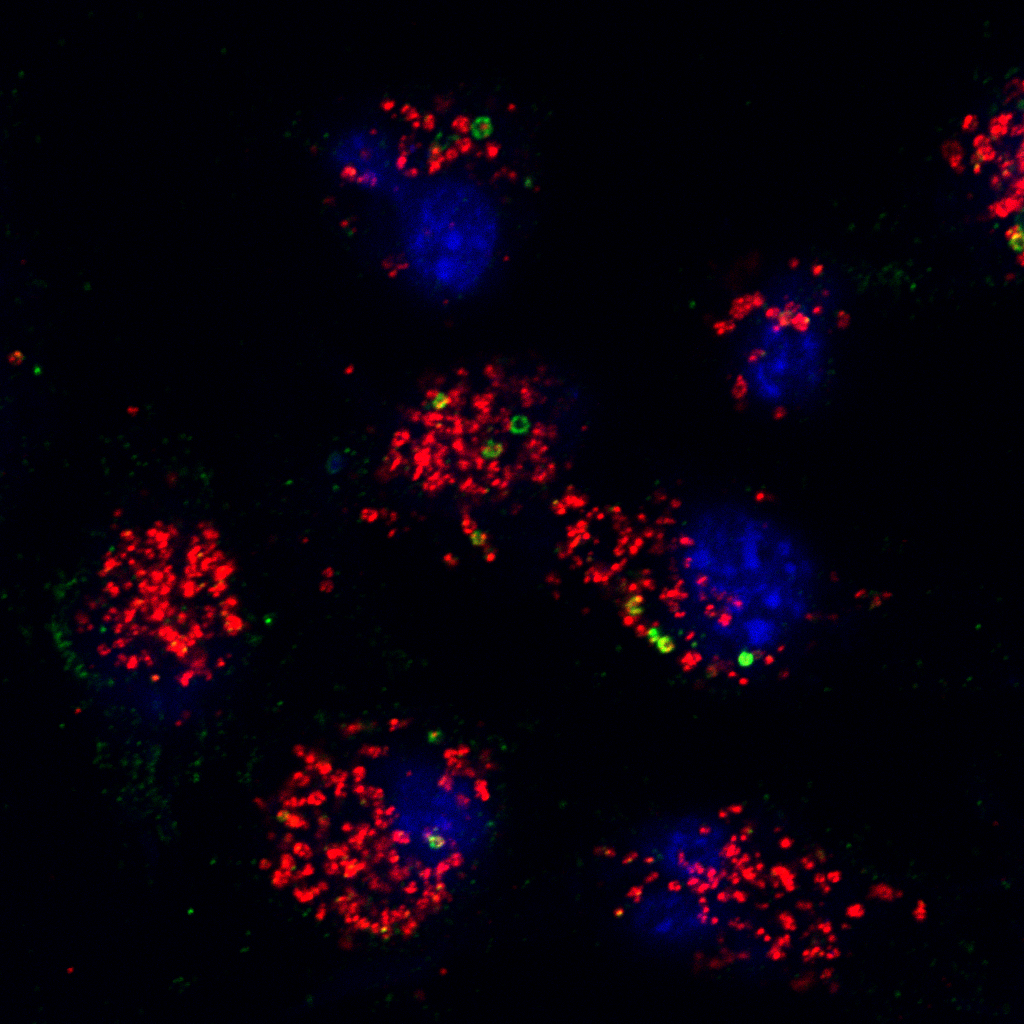

Supplement: Supplementary file 10 — Source data Fig. 9 [file 44318_2026_817_MOESM10_ESM.zip › 9E/9E-1-LLOMe 10 min-WT.tif]

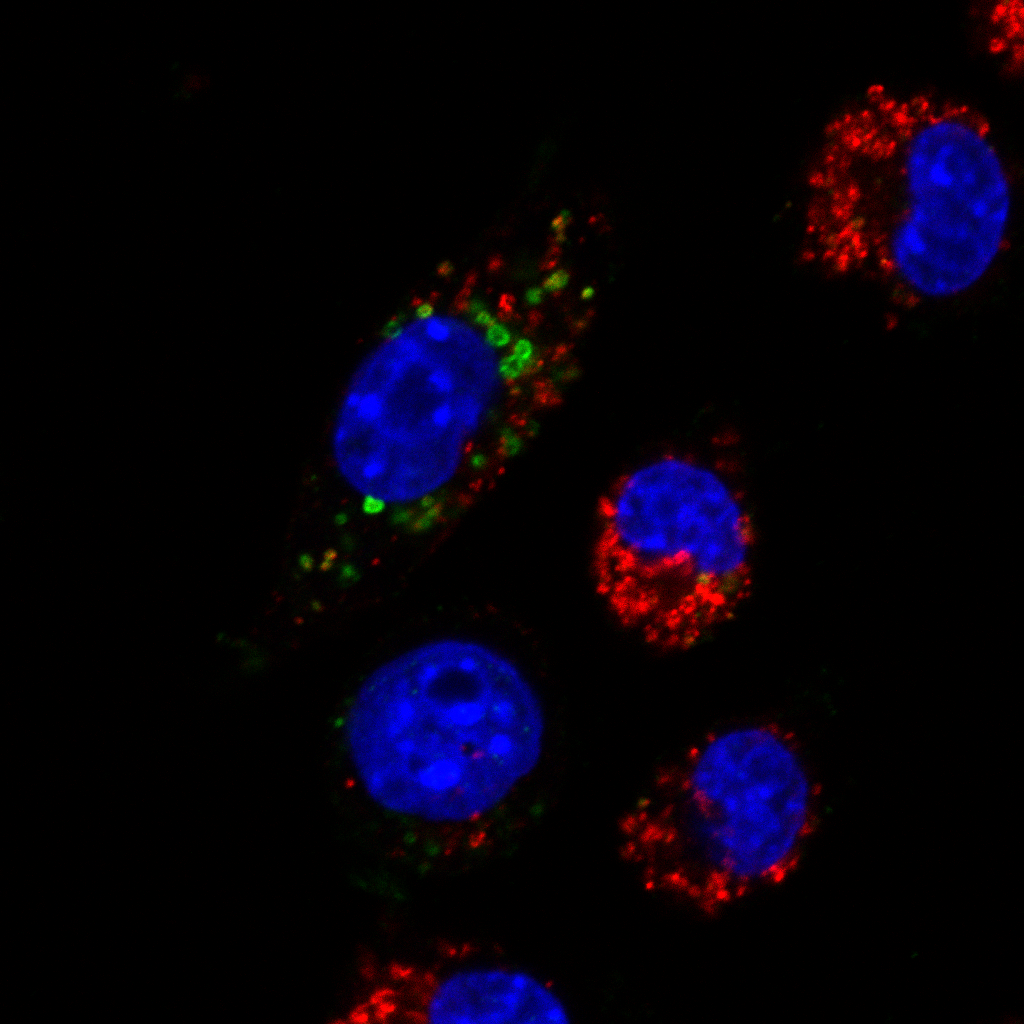

Supplement: Supplementary file 10 — Source data Fig. 9 [file 44318_2026_817_MOESM10_ESM.zip › 9E/9E-2-LLOMe 30 min-dKO.tif]

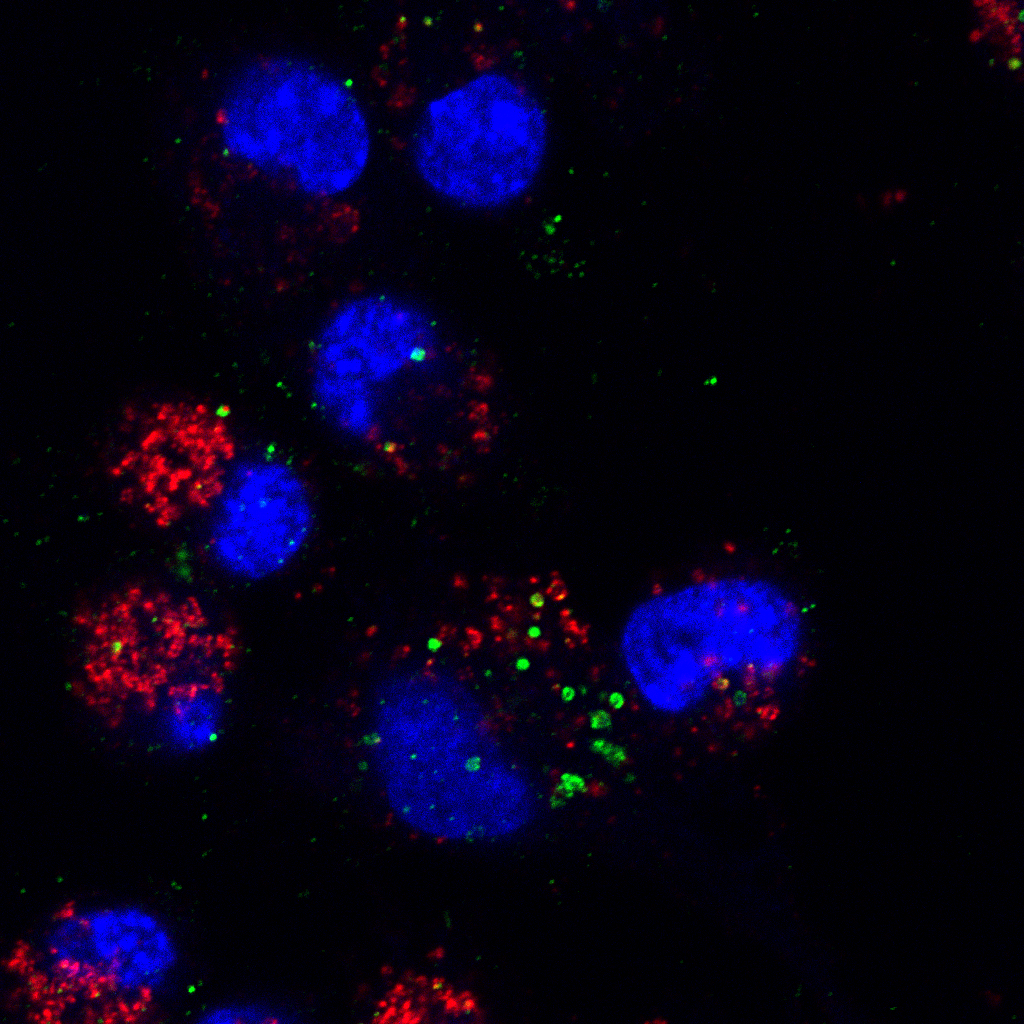

Supplement: Supplementary file 10 — Source data Fig. 9 [file 44318_2026_817_MOESM10_ESM.zip › 9E/9E-2-LLOMe 30 min-dKO_expressing-C9-W33A, S8-R147A.tif]

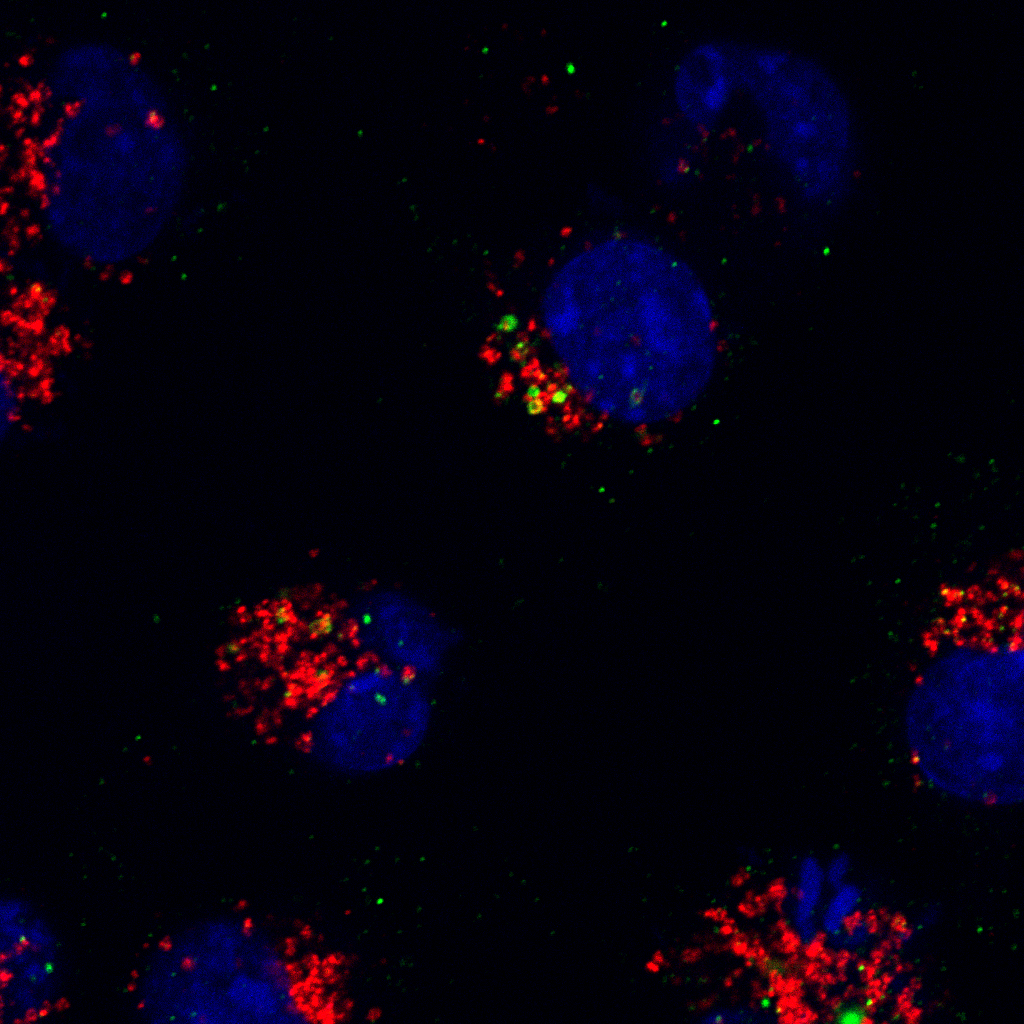

Supplement: Supplementary file 10 — Source data Fig. 9 [file 44318_2026_817_MOESM10_ESM.zip › 9E/9E-2-LLOMe 30 min-dKO_expressing-C9-WT, S8-WT.tif]

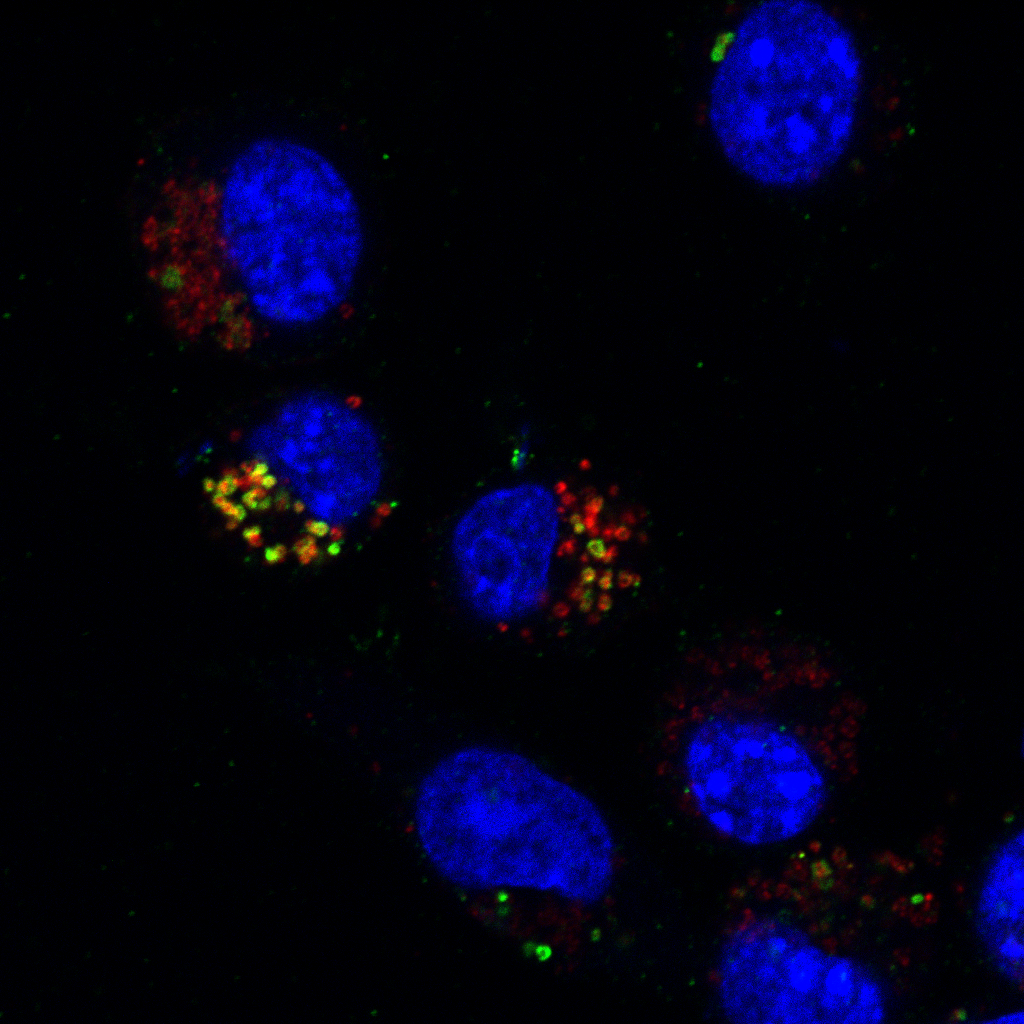

Supplement: Supplementary file 10 — Source data Fig. 9 [file 44318_2026_817_MOESM10_ESM.zip › 9E/9E-2-LLOMe 30 min-WT.tif]

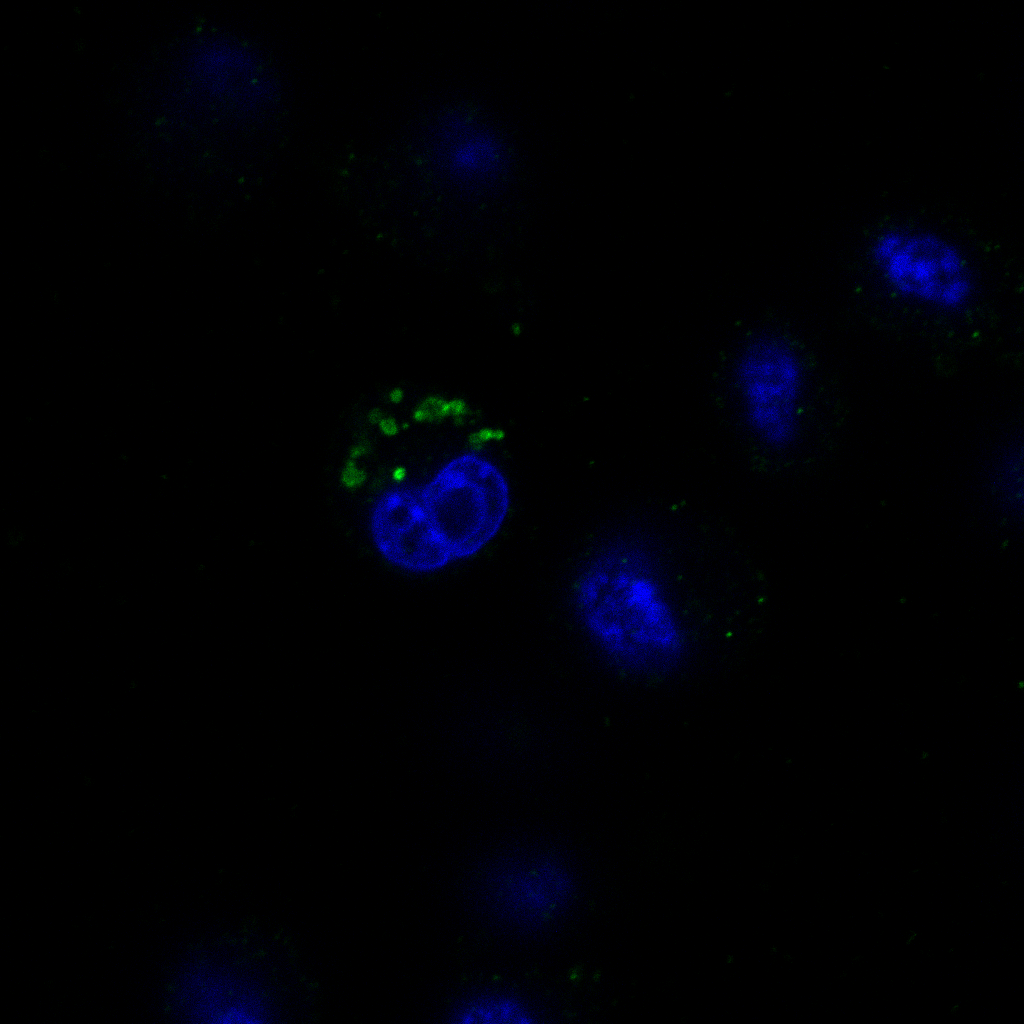

Supplement: Supplementary file 10 — Source data Fig. 9 [file 44318_2026_817_MOESM10_ESM.zip › 9G/9G-1-LLOMe 10 min-dKO.tif]

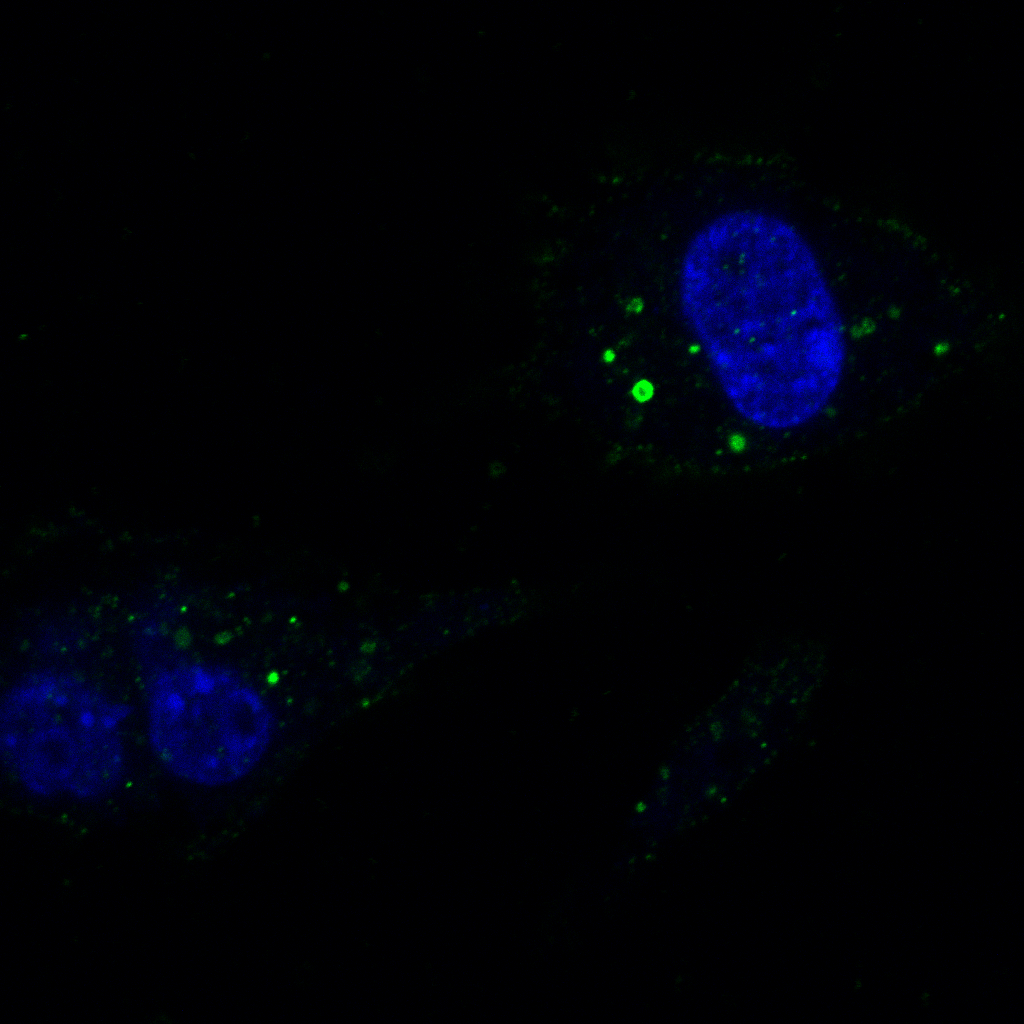

Supplement: Supplementary file 10 — Source data Fig. 9 [file 44318_2026_817_MOESM10_ESM.zip › 9G/9G-1-LLOMe 10 min-WT.tif]

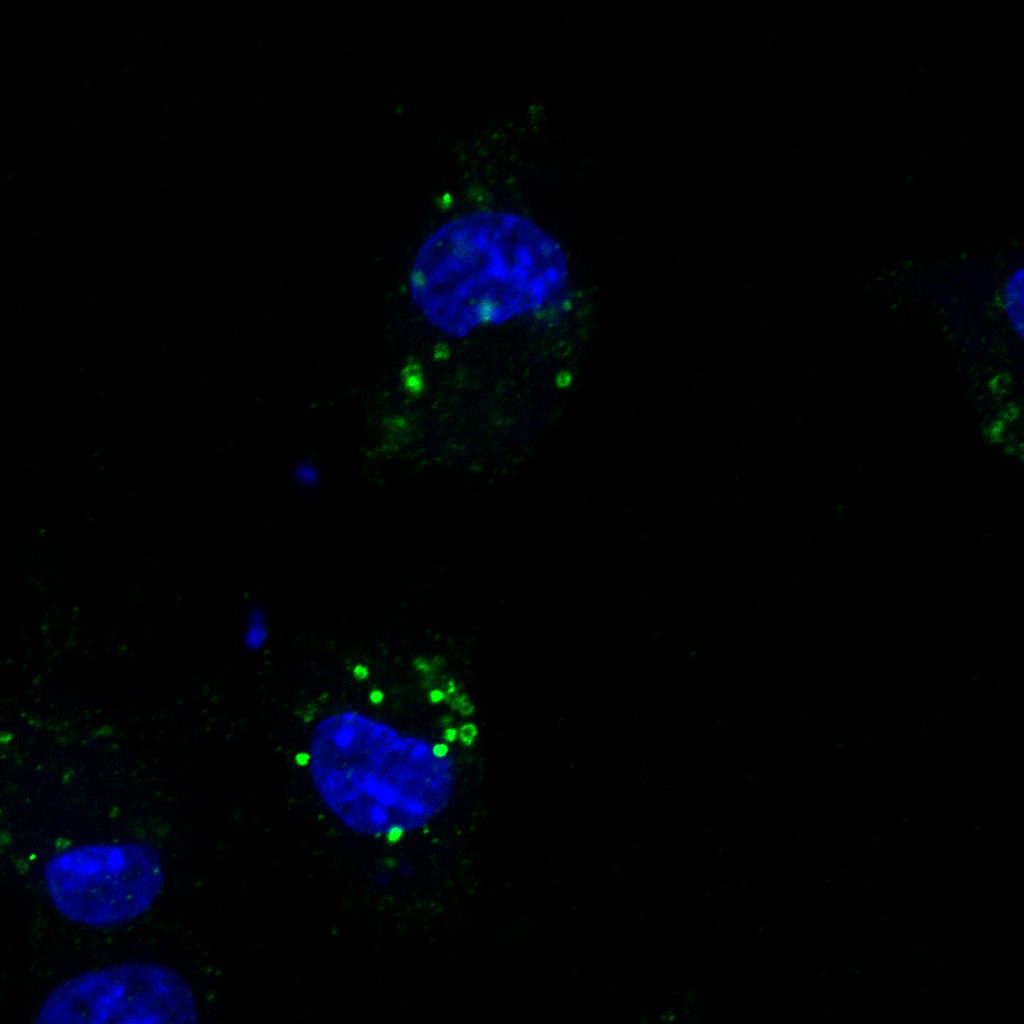

Supplement: Supplementary file 10 — Source data Fig. 9 [file 44318_2026_817_MOESM10_ESM.zip › 9G/9G-2-LLOMe 30 min-dKO.tif]

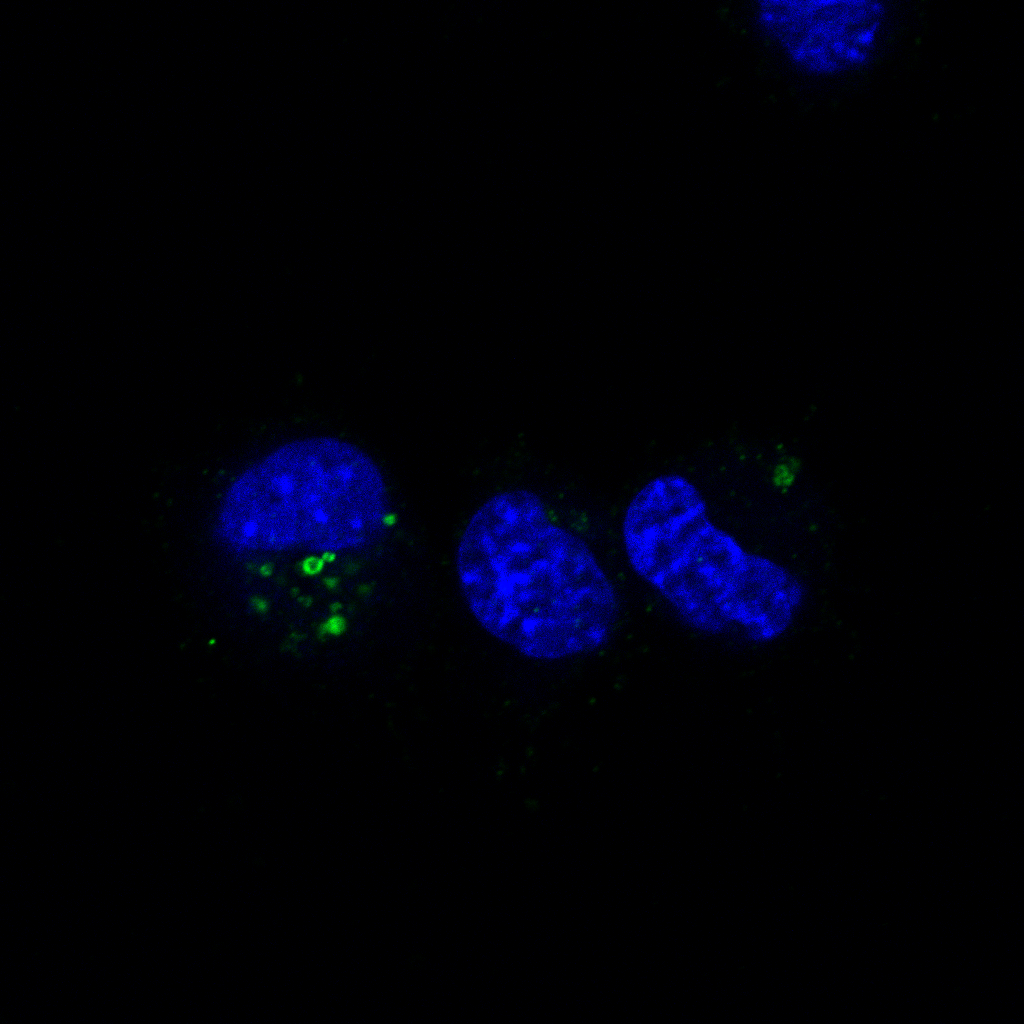

Supplement: Supplementary file 10 — Source data Fig. 9 [file 44318_2026_817_MOESM10_ESM.zip › 9G/9G-2-LLOMe 30 min-WT.tif]

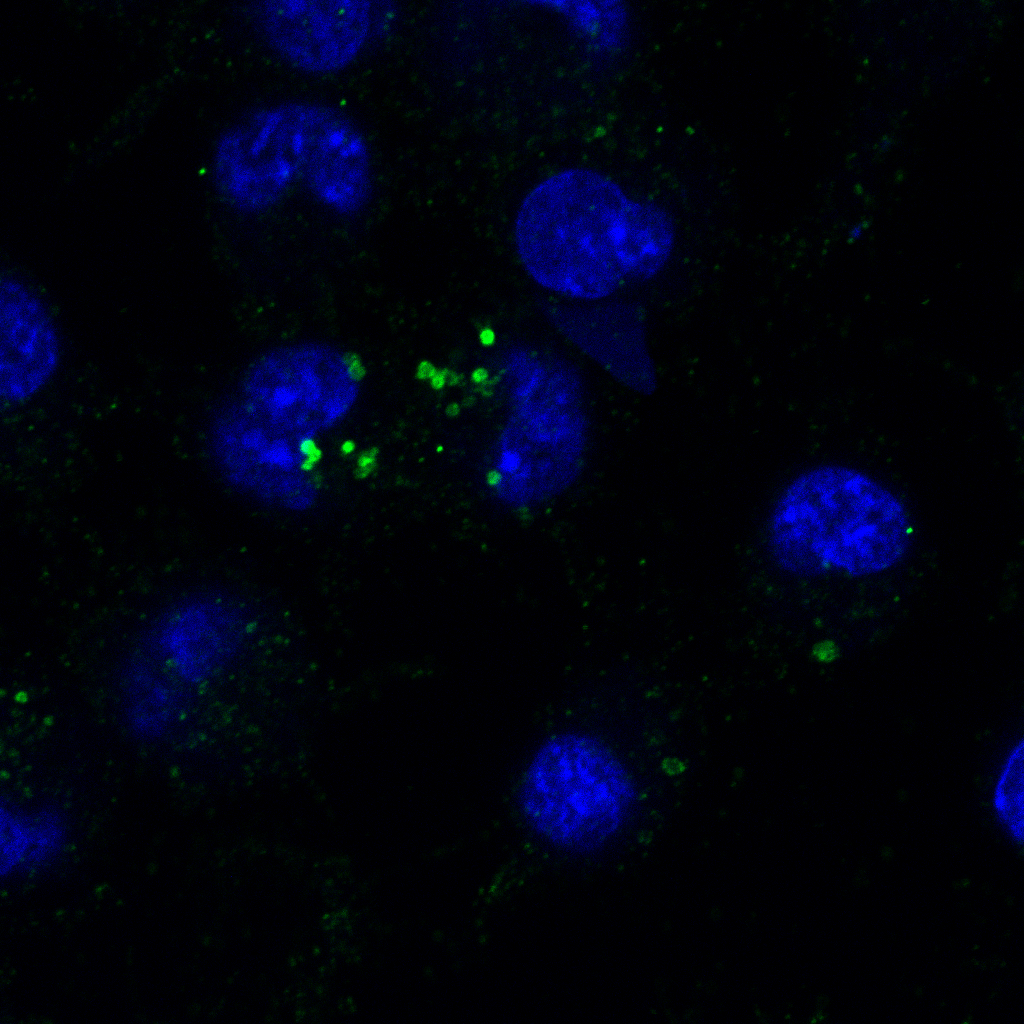

Supplement: Supplementary file 10 — Source data Fig. 9 [file 44318_2026_817_MOESM10_ESM.zip › 9G/9G-3-LLOMe 10 min-dKO_expressing-C9-W33A, S8-R147A.tif]

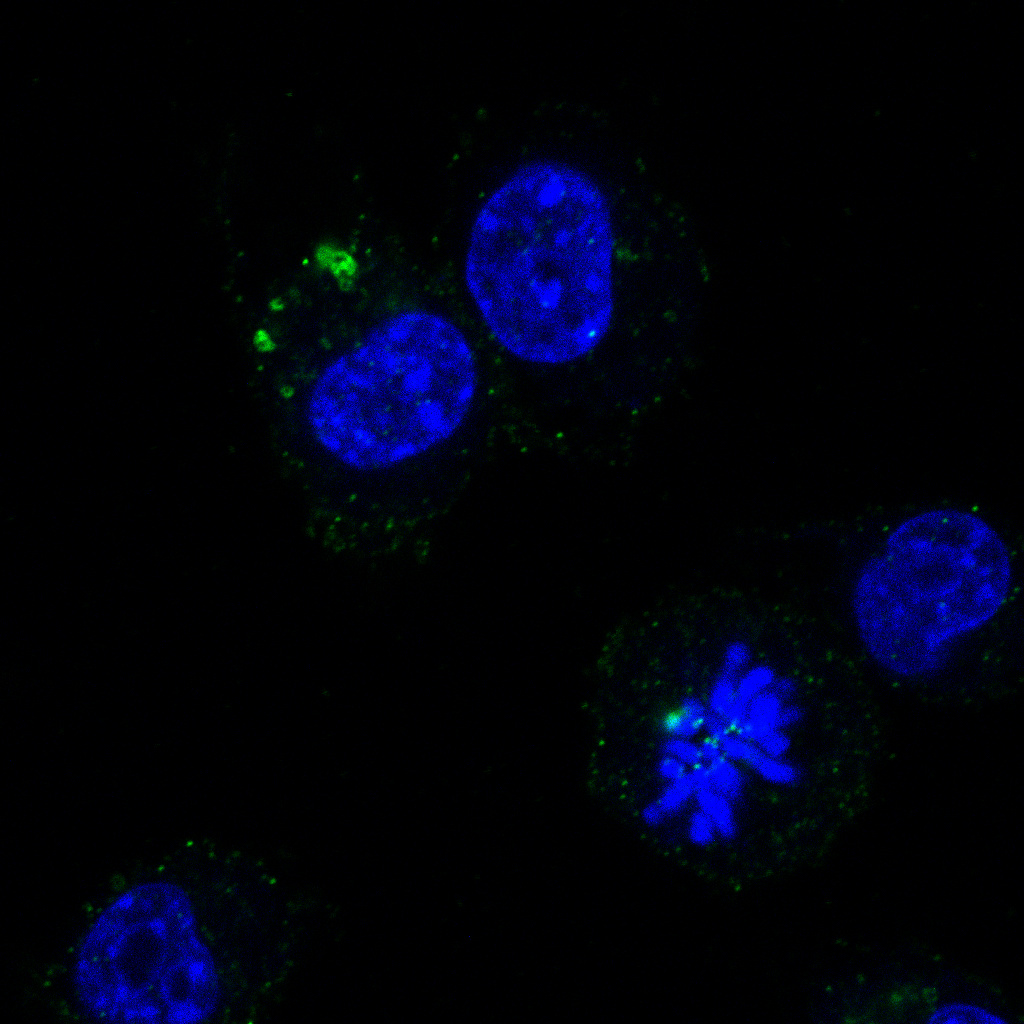

Supplement: Supplementary file 10 — Source data Fig. 9 [file 44318_2026_817_MOESM10_ESM.zip › 9G/9G-3-LLOMe 10 min-dKO_expressing-C9-WT, S8-WT.tif]

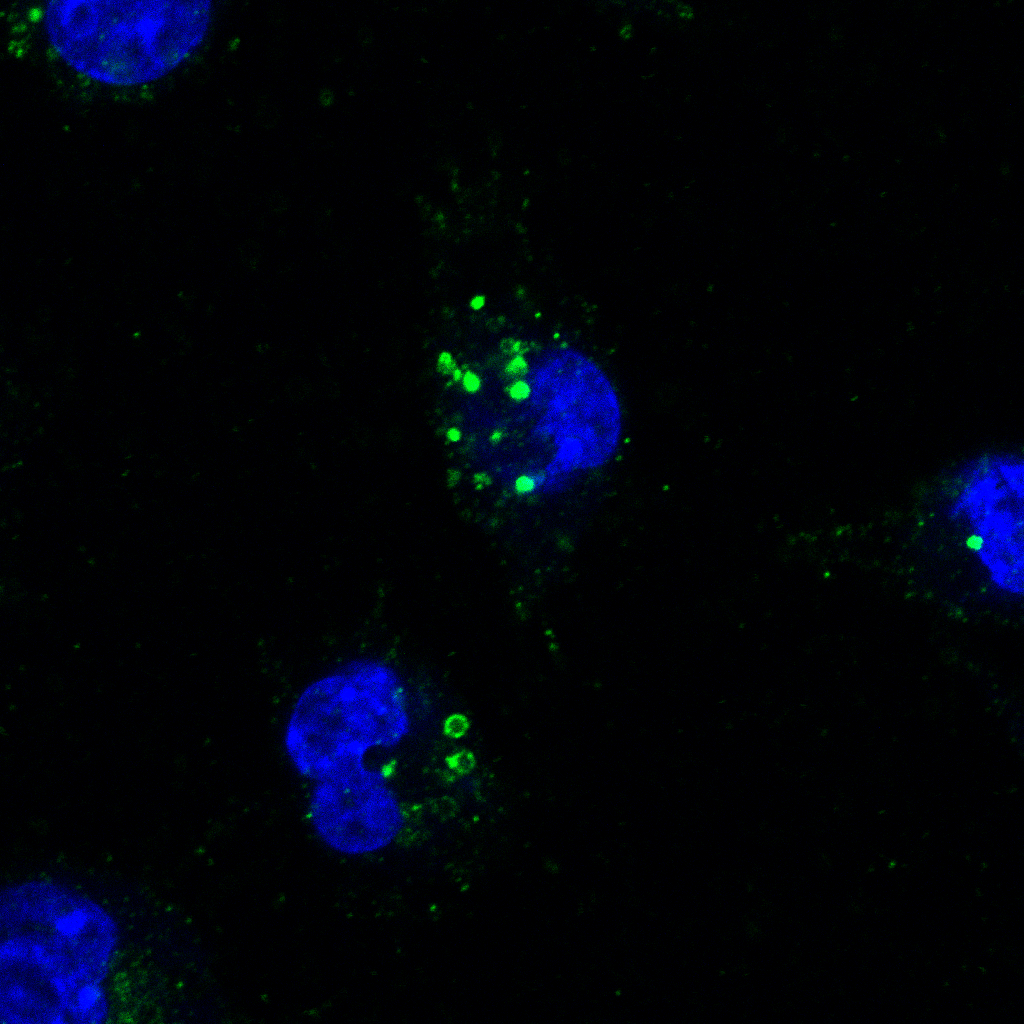

Supplement: Supplementary file 10 — Source data Fig. 9 [file 44318_2026_817_MOESM10_ESM.zip › 9G/9G-4-LLOMe 30 min-dKO_expressing-C9-W33A, S8-R147A.tif]

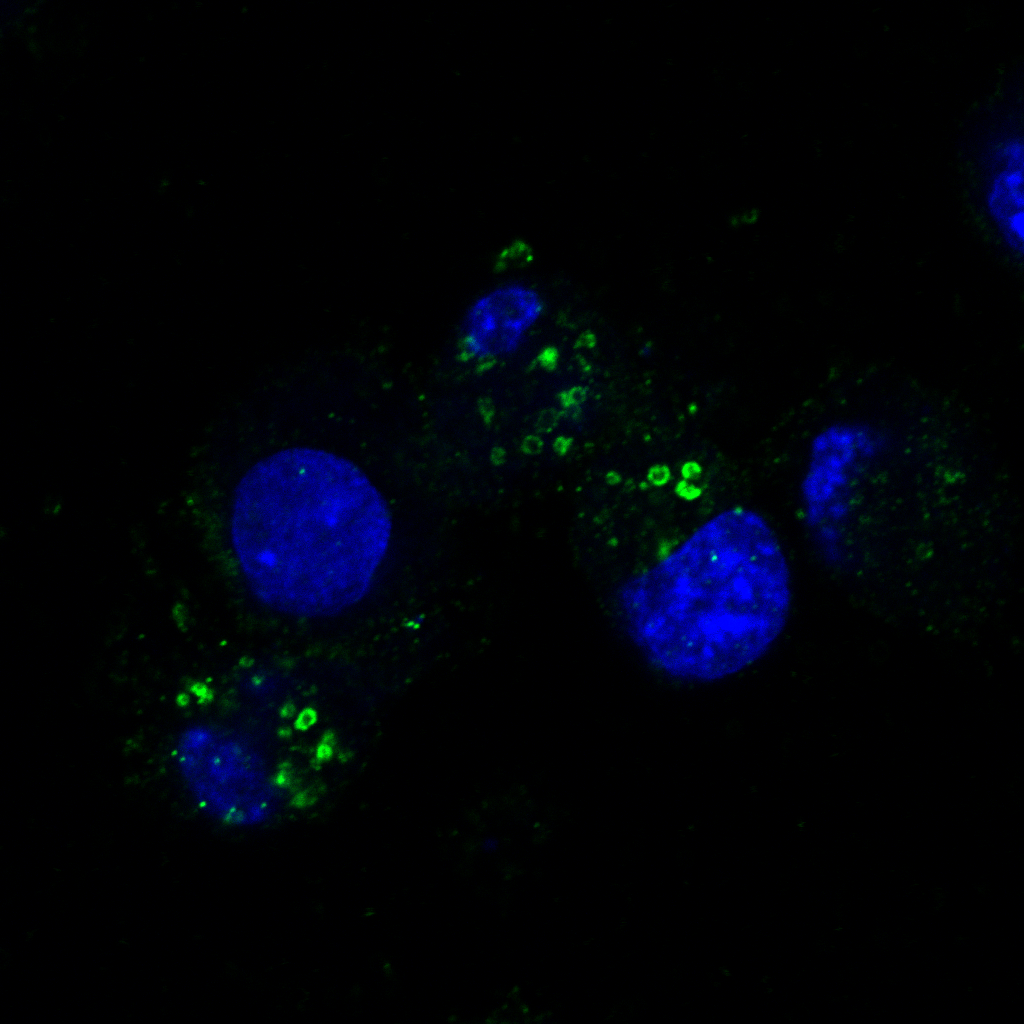

Supplement: Supplementary file 10 — Source data Fig. 9 [file 44318_2026_817_MOESM10_ESM.zip › 9G/9G-4-LLOMe 30 min-dKO_expressing-C9-WT, S8-WT.tif]

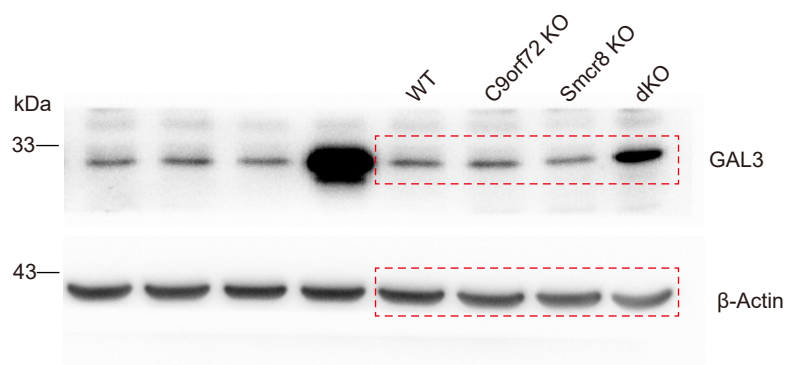

Supplement: Supplementary file 11 — Figure EV2 Source Data [file 44318_2026_817_MOESM11_ESM.zip › EV2A/EV2A.pdf]

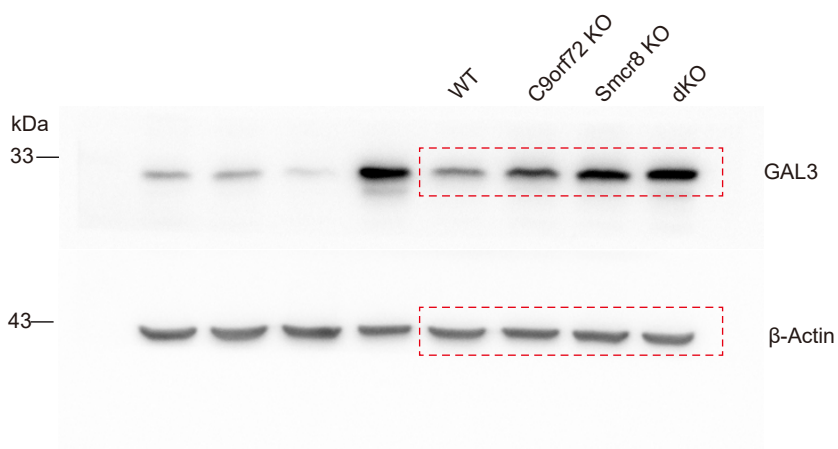

Supplement: Supplementary file 11 — Figure EV2 Source Data [file 44318_2026_817_MOESM11_ESM.zip › EV2B/EV2B.pdf]

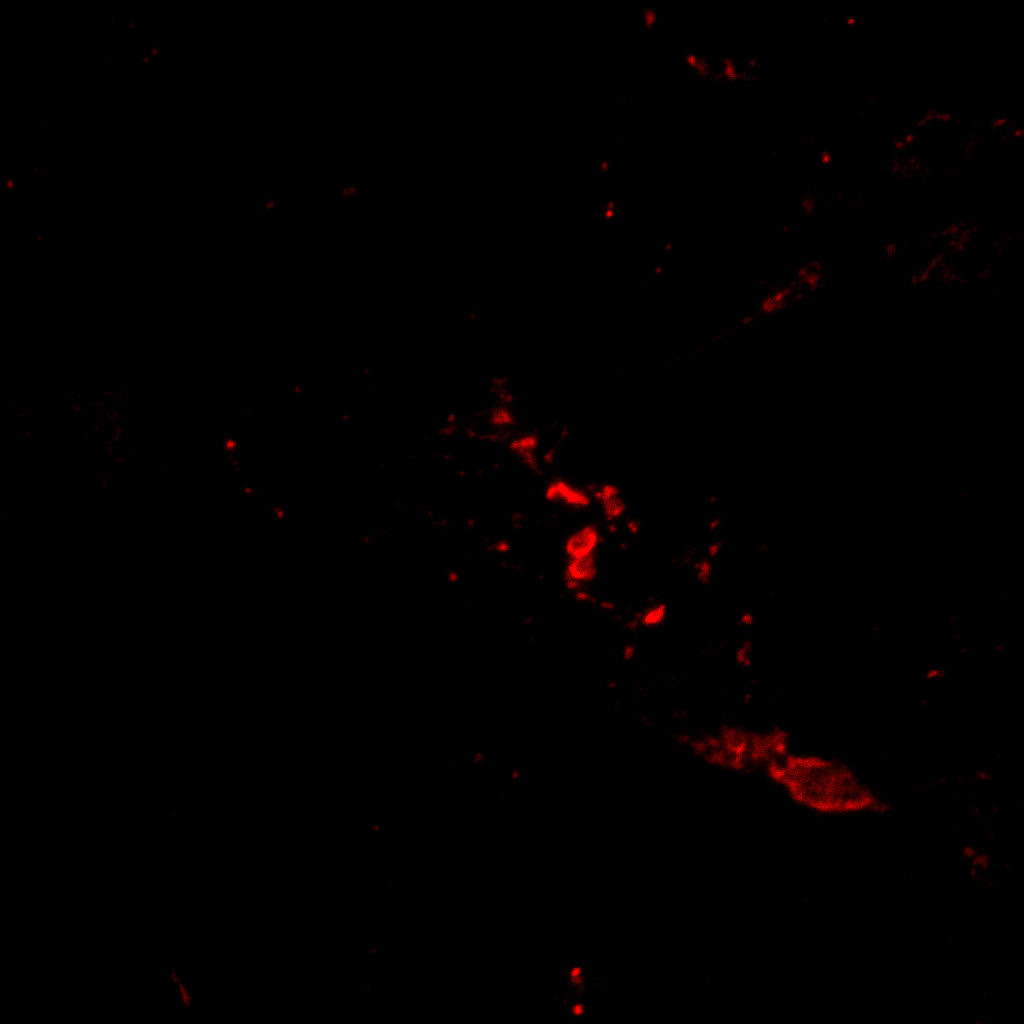

Supplement: Supplementary file 11 — Figure EV2 Source Data [file 44318_2026_817_MOESM11_ESM.zip › EV2E/EV2E-1-WT_CD68.tif]

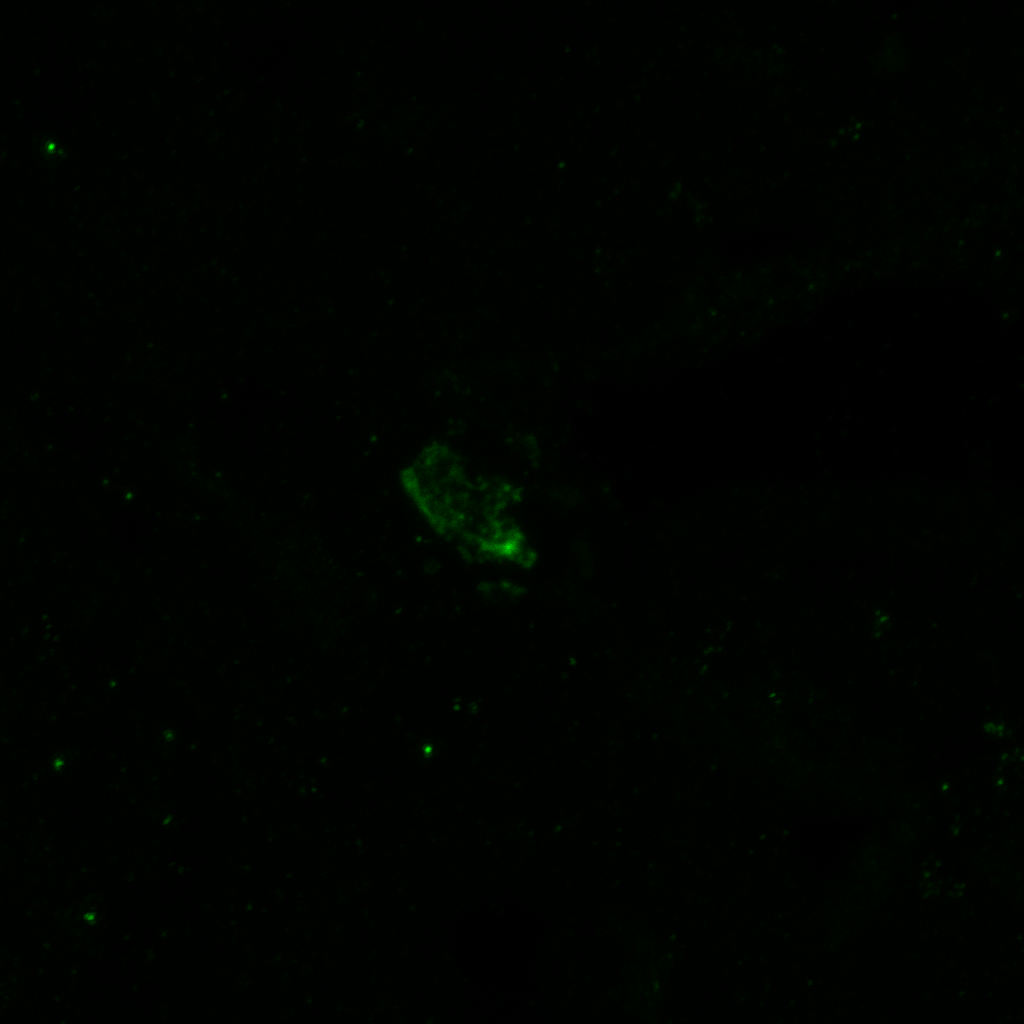

Supplement: Supplementary file 11 — Figure EV2 Source Data [file 44318_2026_817_MOESM11_ESM.zip › EV2E/EV2E-1-WT_GAL3.tif]

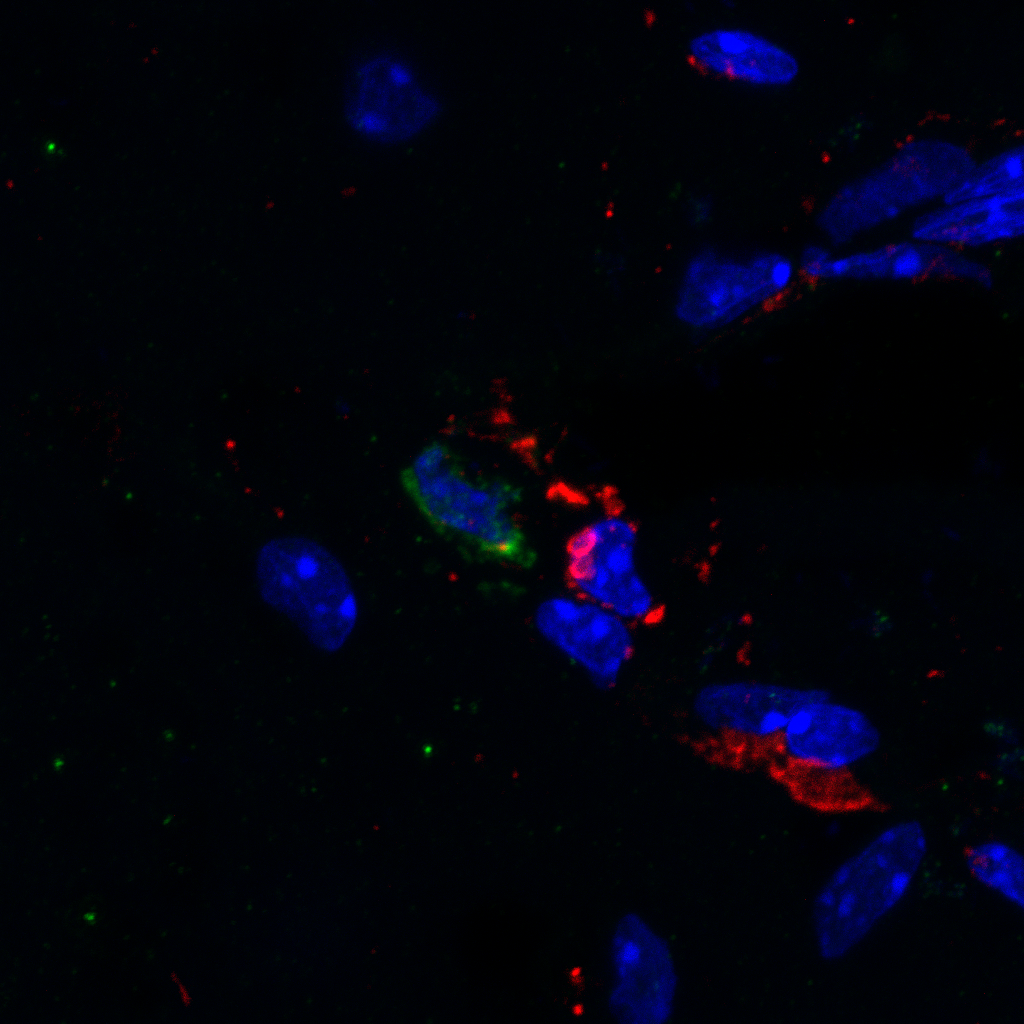

Supplement: Supplementary file 11 — Figure EV2 Source Data [file 44318_2026_817_MOESM11_ESM.zip › EV2E/EV2E-1-WT_Merge.tif]

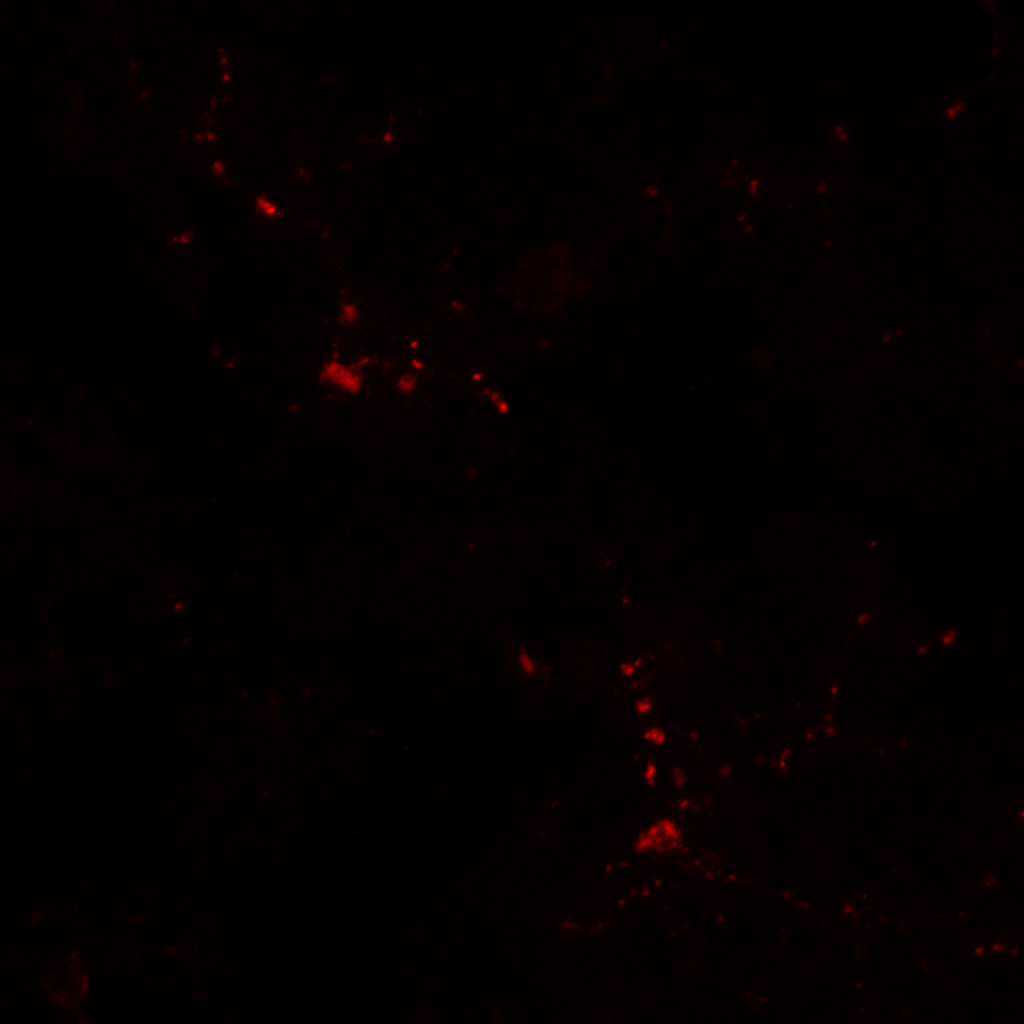

Supplement: Supplementary file 11 — Figure EV2 Source Data [file 44318_2026_817_MOESM11_ESM.zip › EV2E/EV2E-2-C9orf72 KO_CD68.tif]

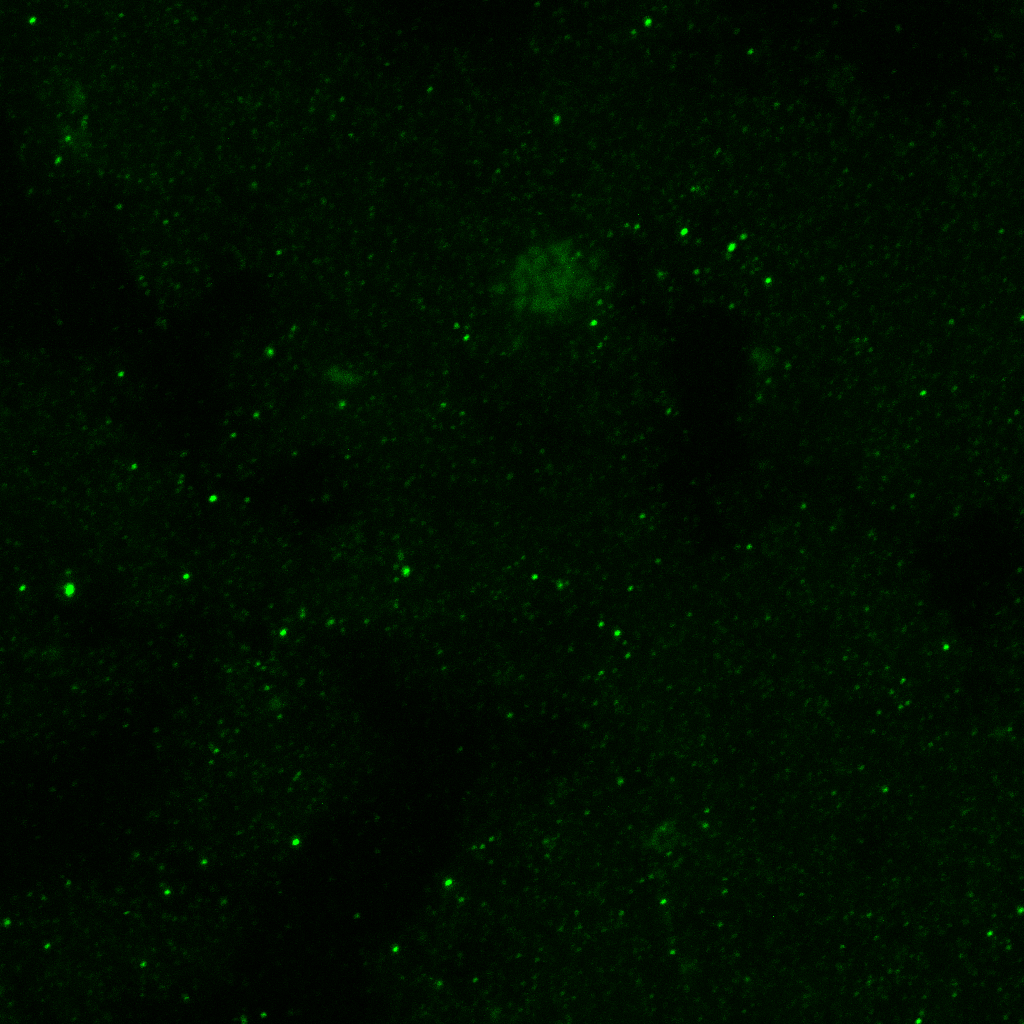

Supplement: Supplementary file 11 — Figure EV2 Source Data [file 44318_2026_817_MOESM11_ESM.zip › EV2E/EV2E-2-C9orf72 KO_GAL3.tif]

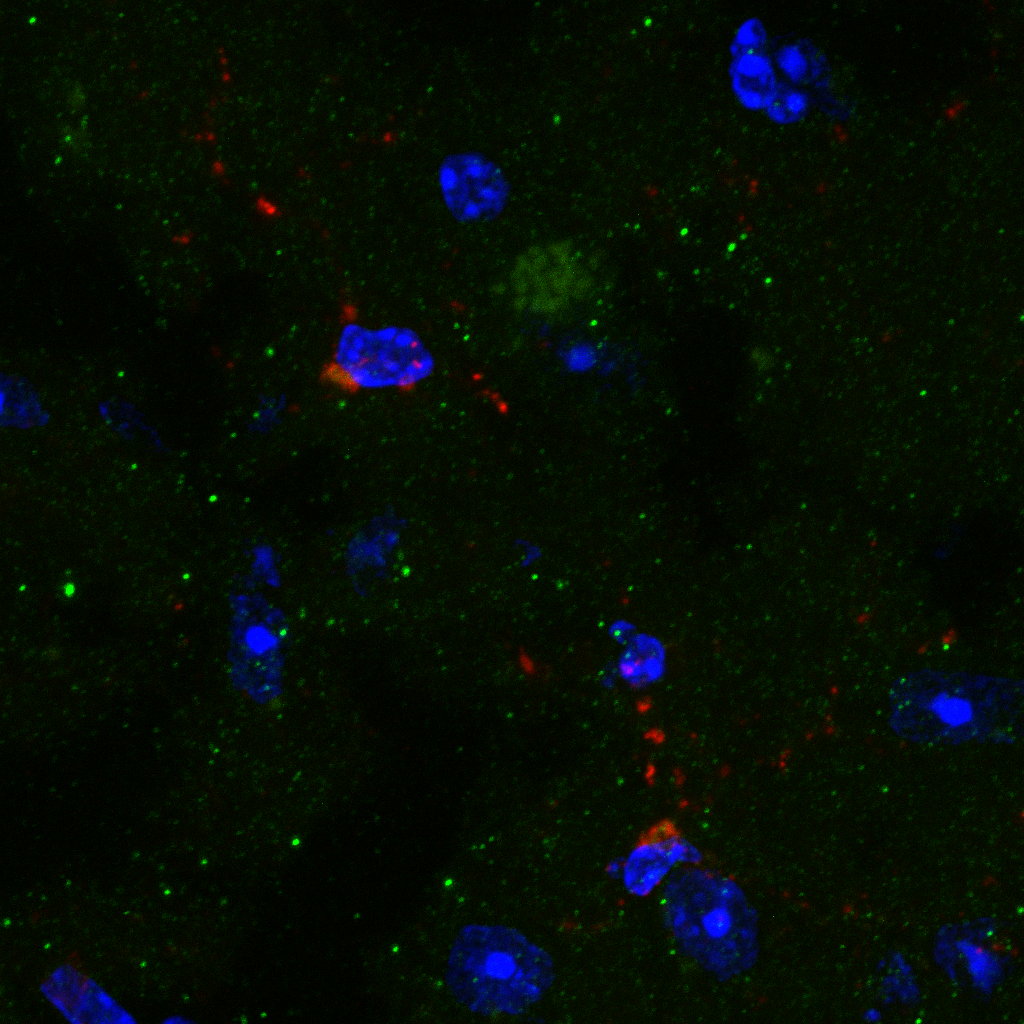

Supplement: Supplementary file 11 — Figure EV2 Source Data [file 44318_2026_817_MOESM11_ESM.zip › EV2E/EV2E-2-C9orf72 KO_Merge.tif]

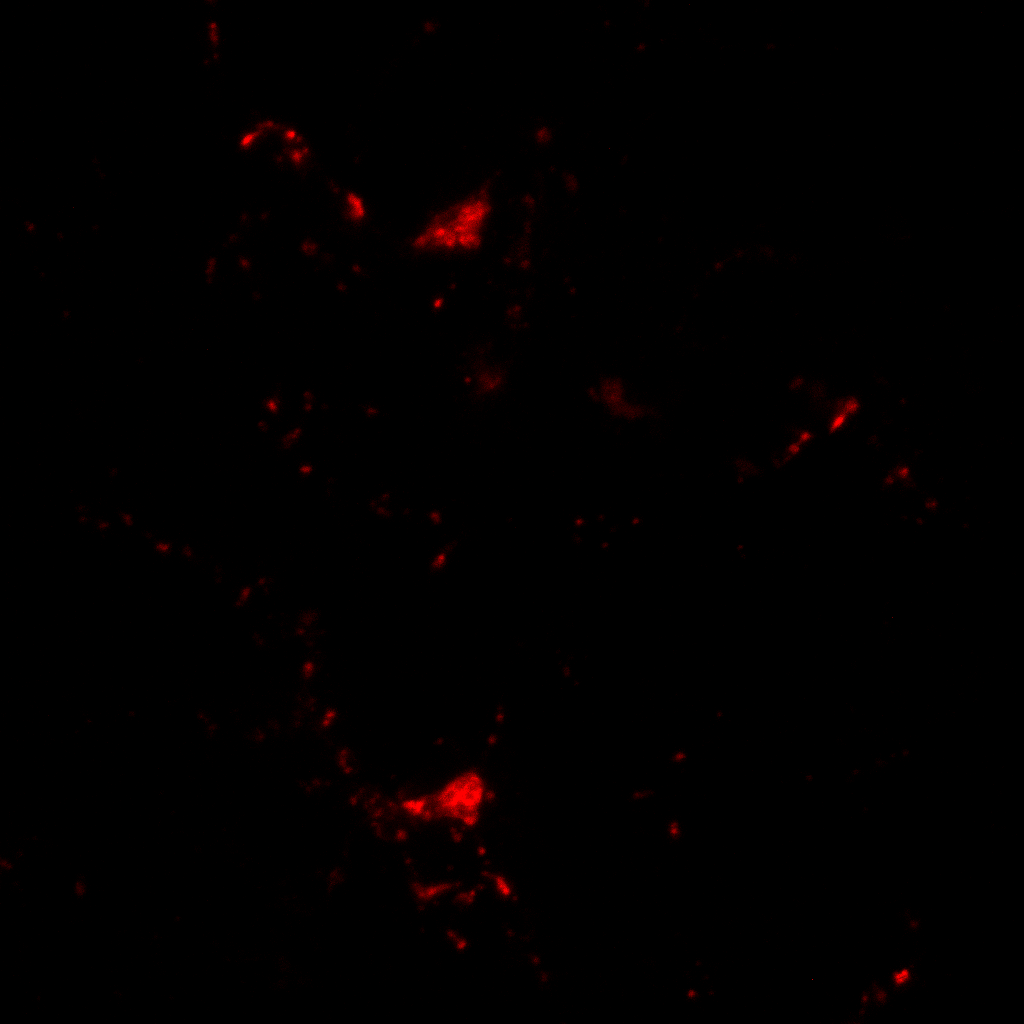

Supplement: Supplementary file 11 — Figure EV2 Source Data [file 44318_2026_817_MOESM11_ESM.zip › EV2E/EV2E-3-Smcr8 KO_CD68.tif]

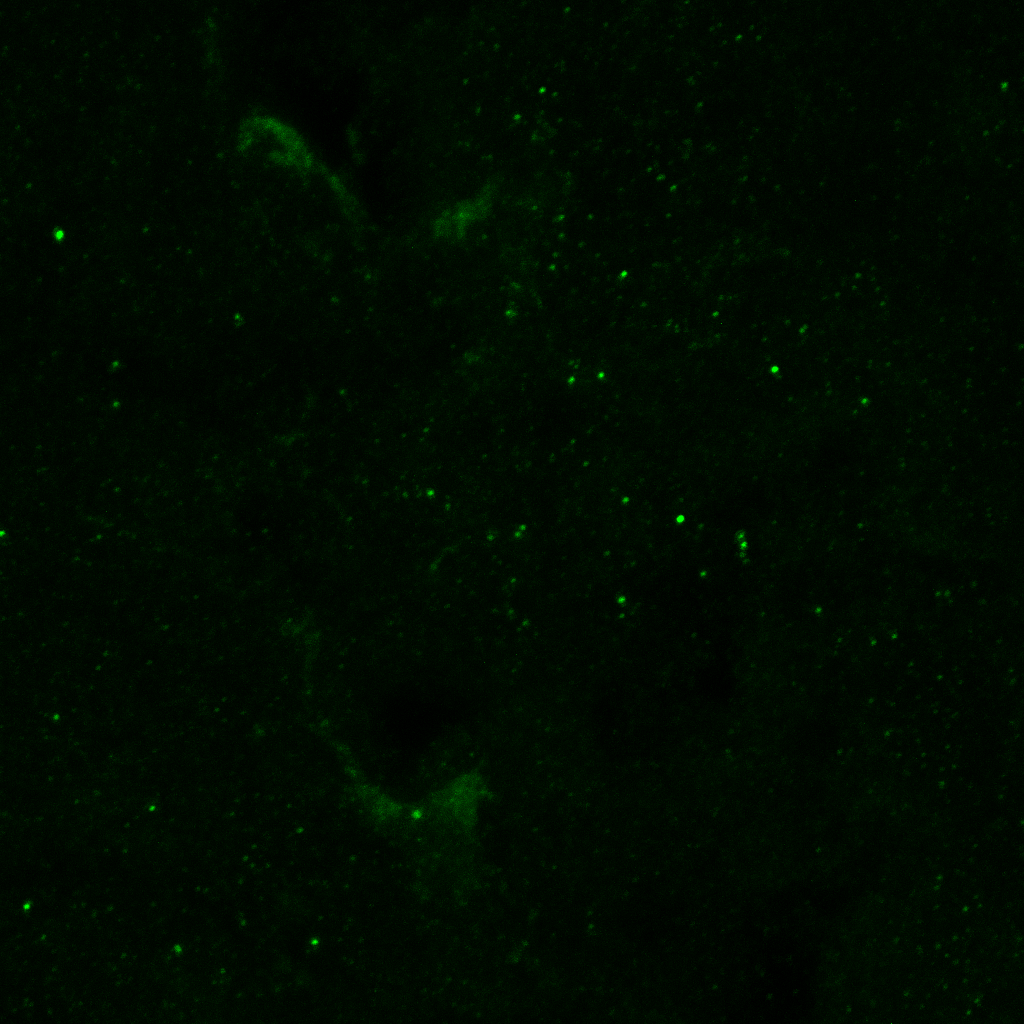

Supplement: Supplementary file 11 — Figure EV2 Source Data [file 44318_2026_817_MOESM11_ESM.zip › EV2E/EV2E-3-Smcr8 KO_GAL3.tif]

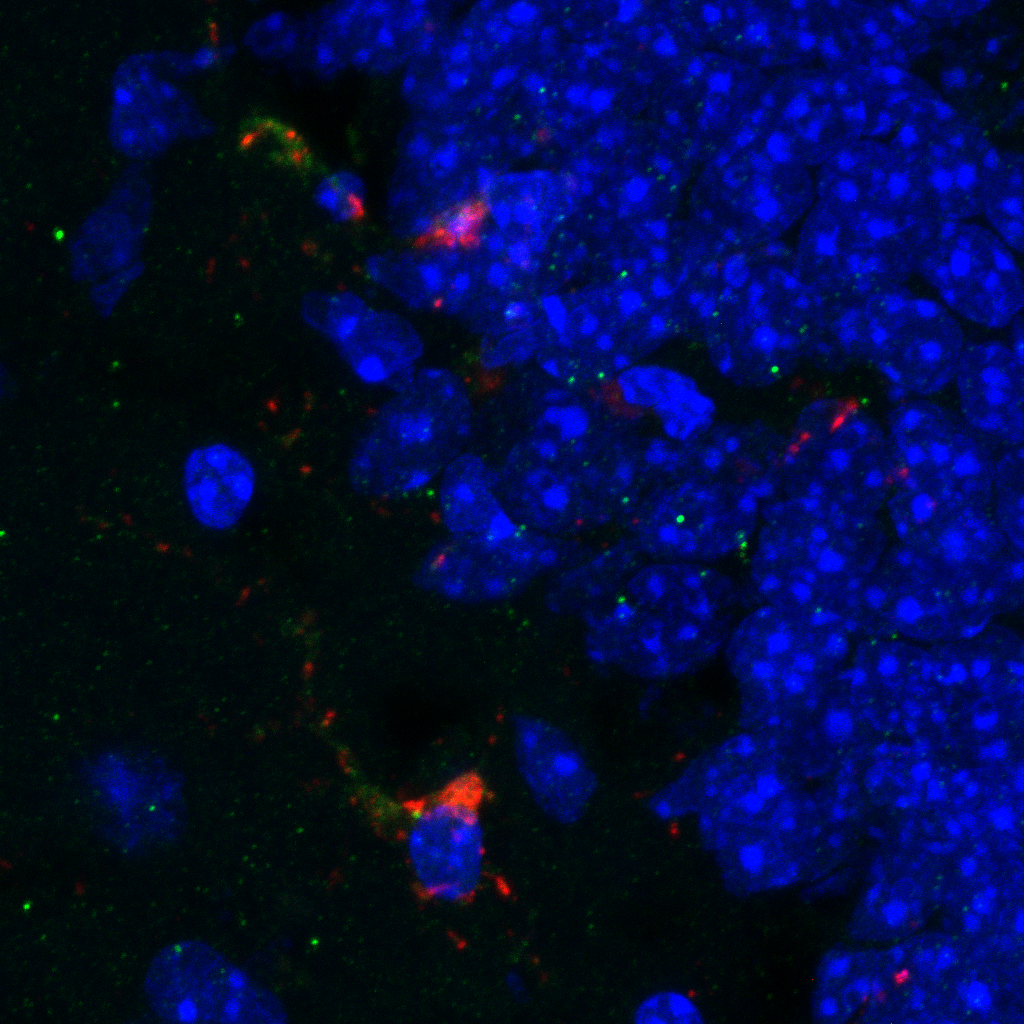

Supplement: Supplementary file 11 — Figure EV2 Source Data [file 44318_2026_817_MOESM11_ESM.zip › EV2E/EV2E-3-Smcr8 KO_Merge.tif]

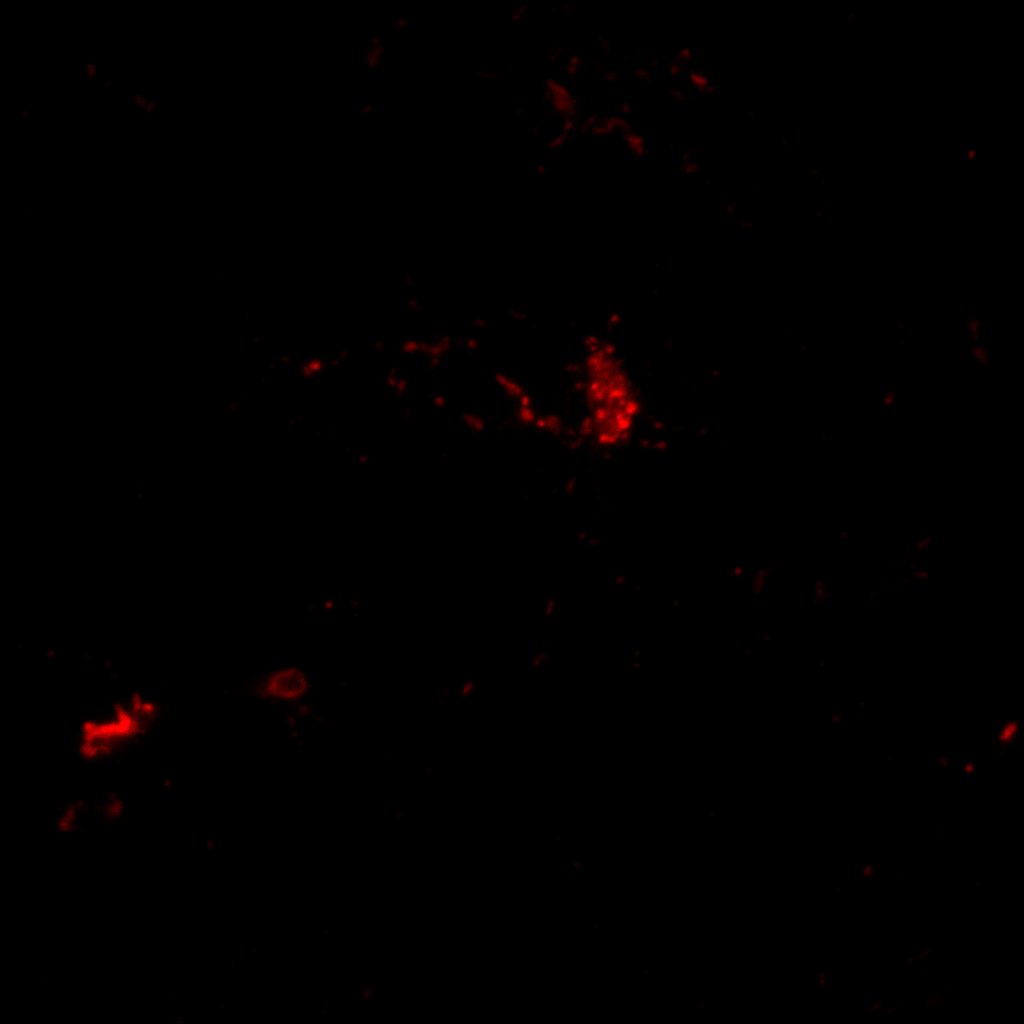

Supplement: Supplementary file 11 — Figure EV2 Source Data [file 44318_2026_817_MOESM11_ESM.zip › EV2E/EV2E-4-dKO_CD68.tif]

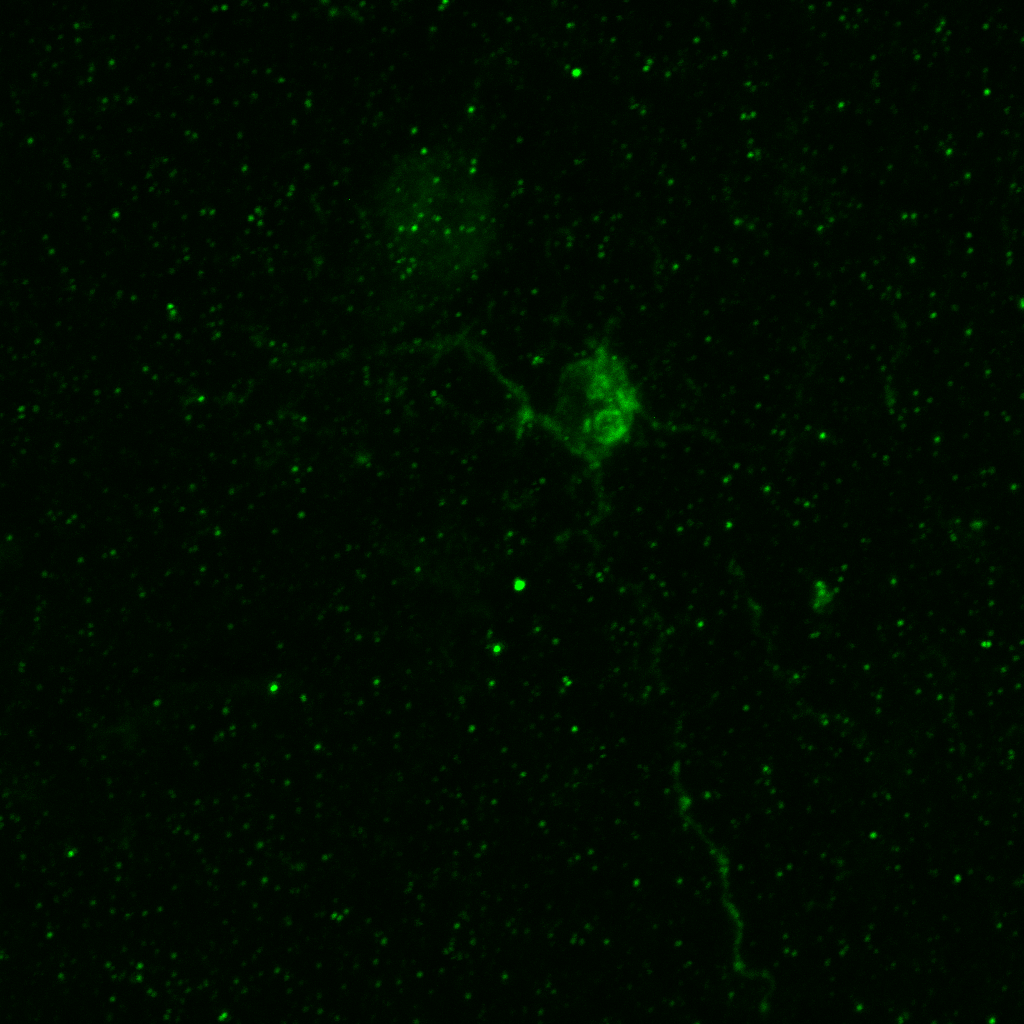

Supplement: Supplementary file 11 — Figure EV2 Source Data [file 44318_2026_817_MOESM11_ESM.zip › EV2E/EV2E-4-dKO_GAL3.tif]

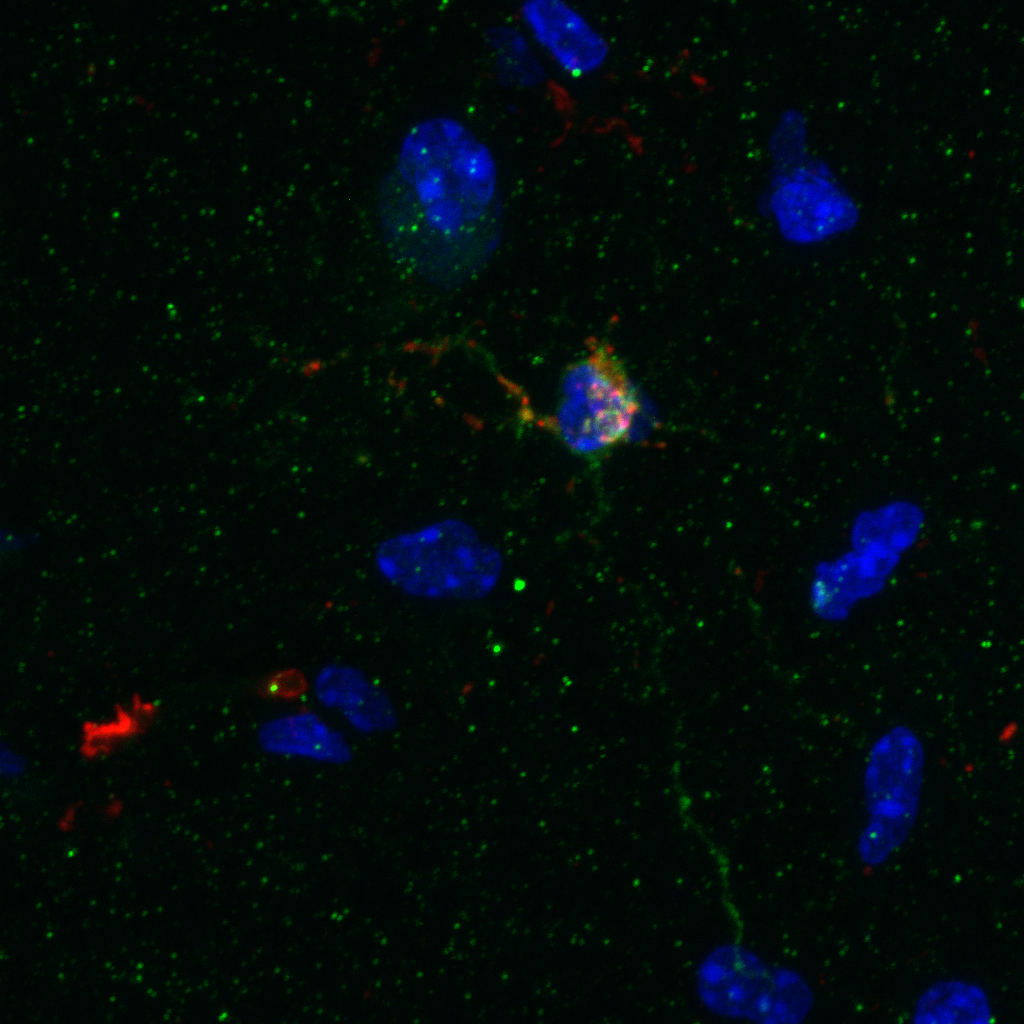

Supplement: Supplementary file 11 — Figure EV2 Source Data [file 44318_2026_817_MOESM11_ESM.zip › EV2E/EV2E-4-dKO_Merge.tif]

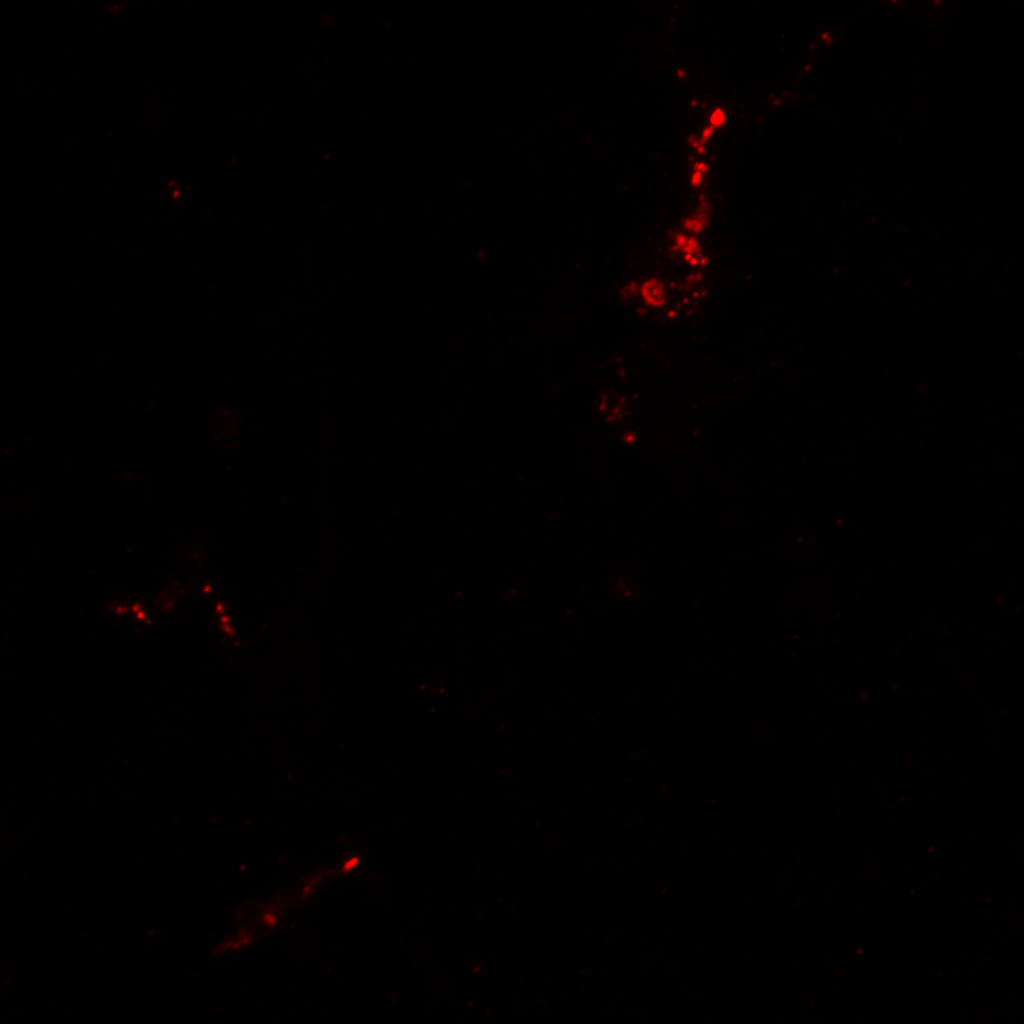

Supplement: Supplementary file 11 — Figure EV2 Source Data [file 44318_2026_817_MOESM11_ESM.zip › EV2F/EV2F-1-WT_CD68.tif]

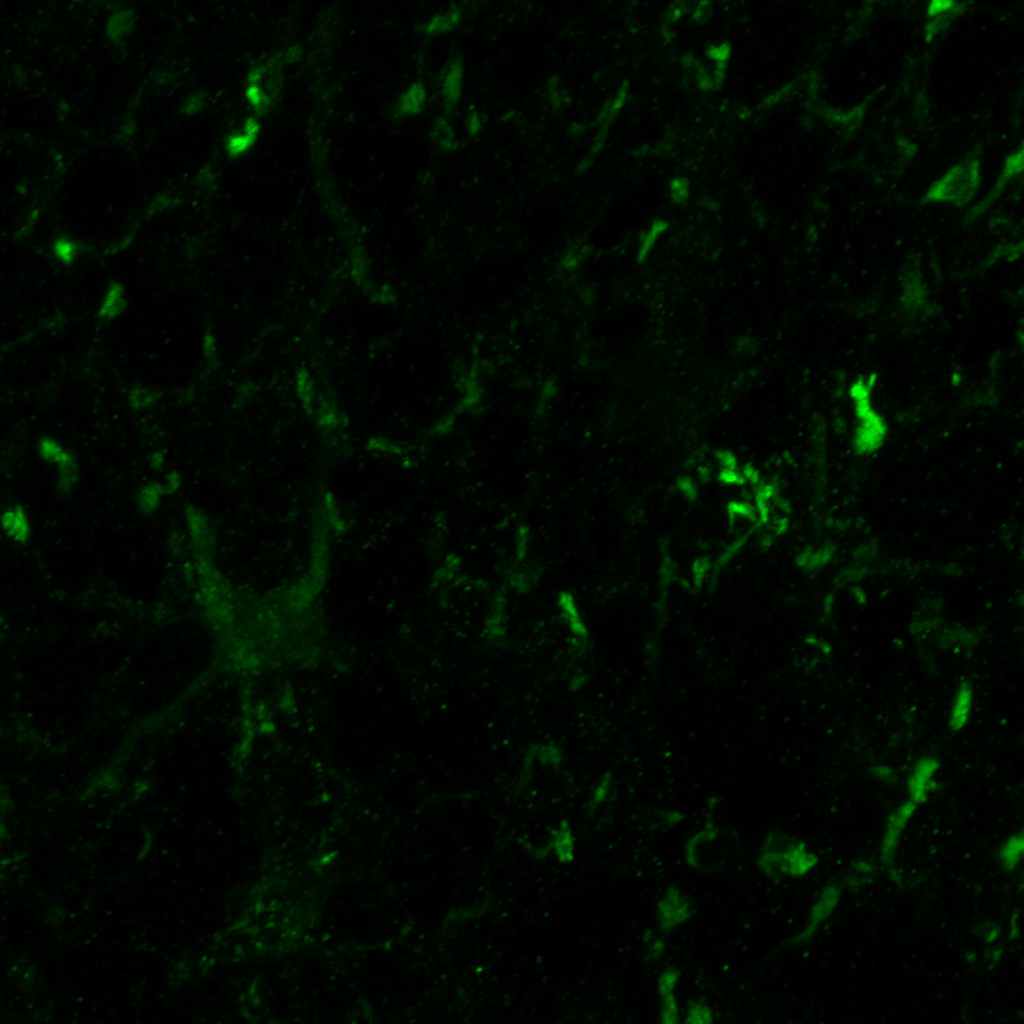

Supplement: Supplementary file 11 — Figure EV2 Source Data [file 44318_2026_817_MOESM11_ESM.zip › EV2F/EV2F-1-WT_GAL3.tif]

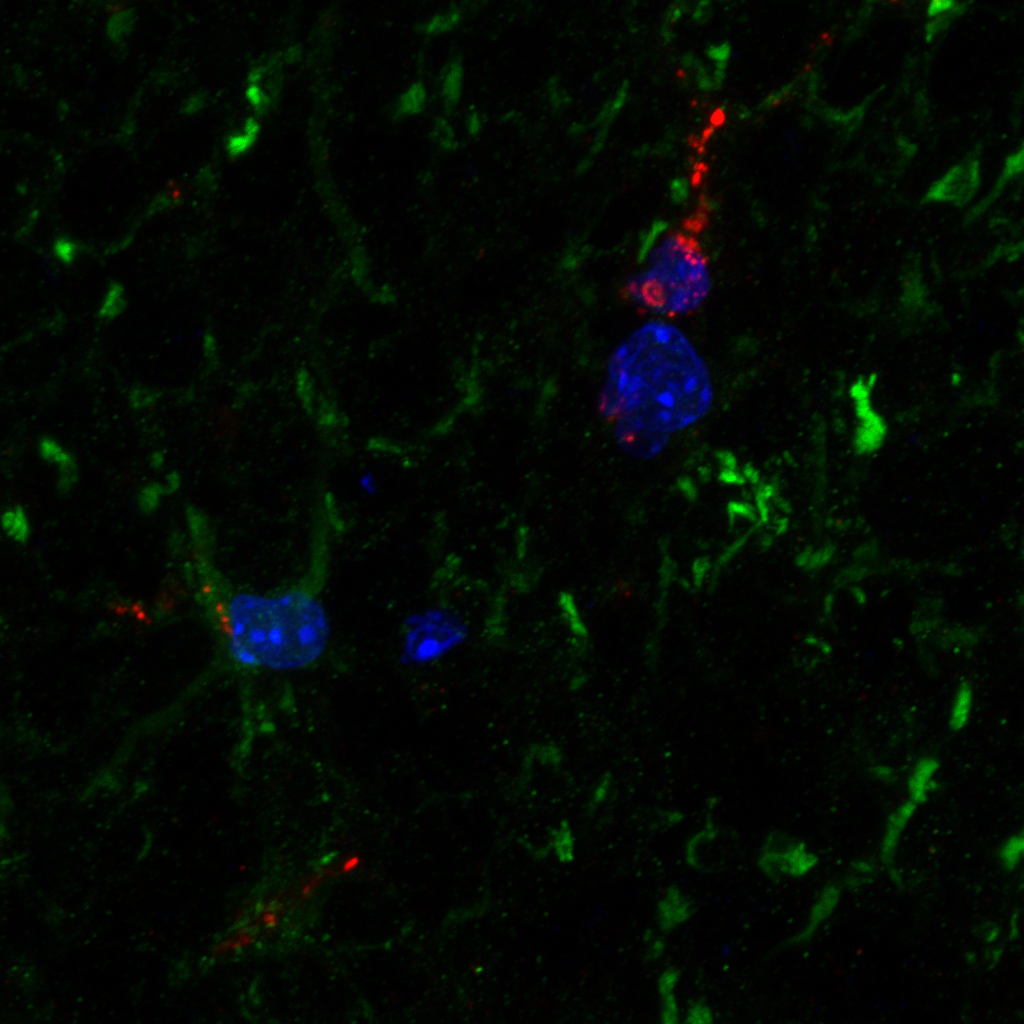

Supplement: Supplementary file 11 — Figure EV2 Source Data [file 44318_2026_817_MOESM11_ESM.zip › EV2F/EV2F-1-WT_Merge.tif]

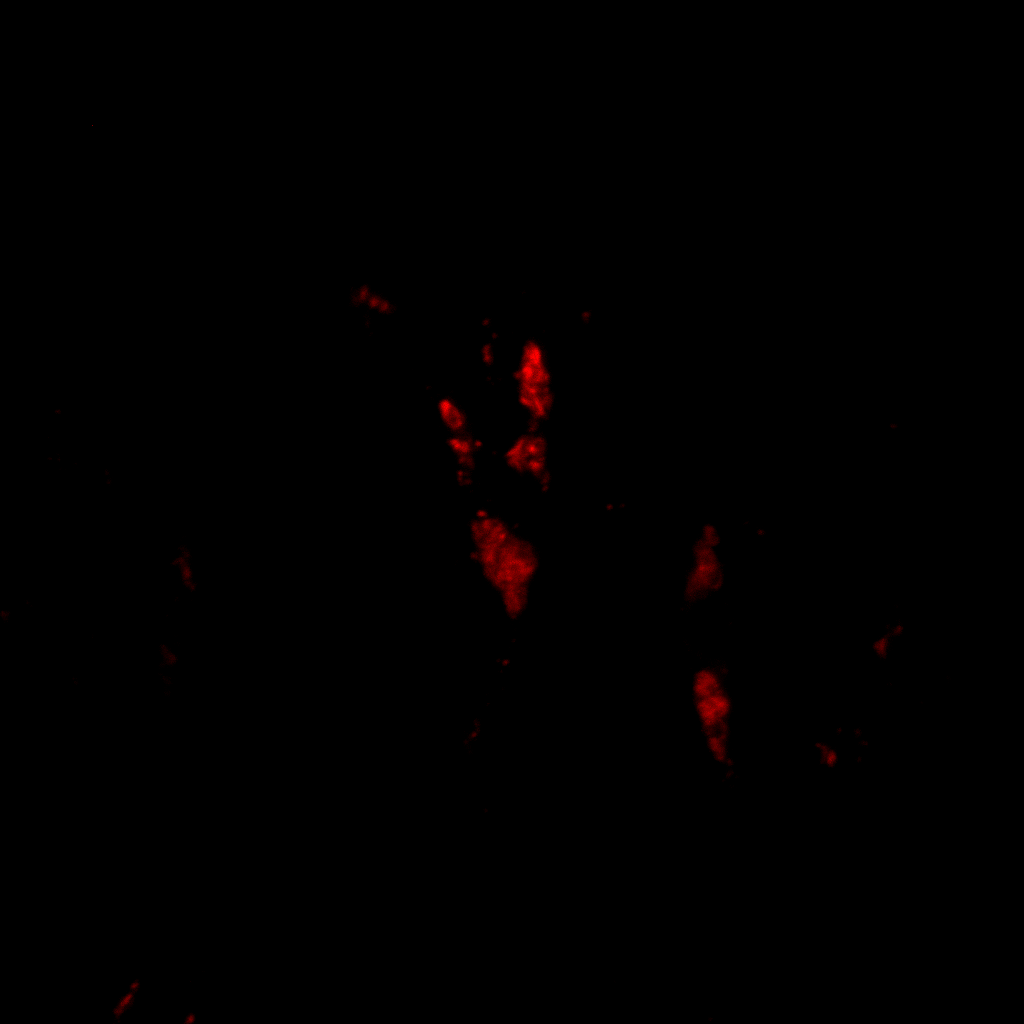

Supplement: Supplementary file 11 — Figure EV2 Source Data [file 44318_2026_817_MOESM11_ESM.zip › EV2F/EV2F-2-C9orf72 KO_CD68.tif]

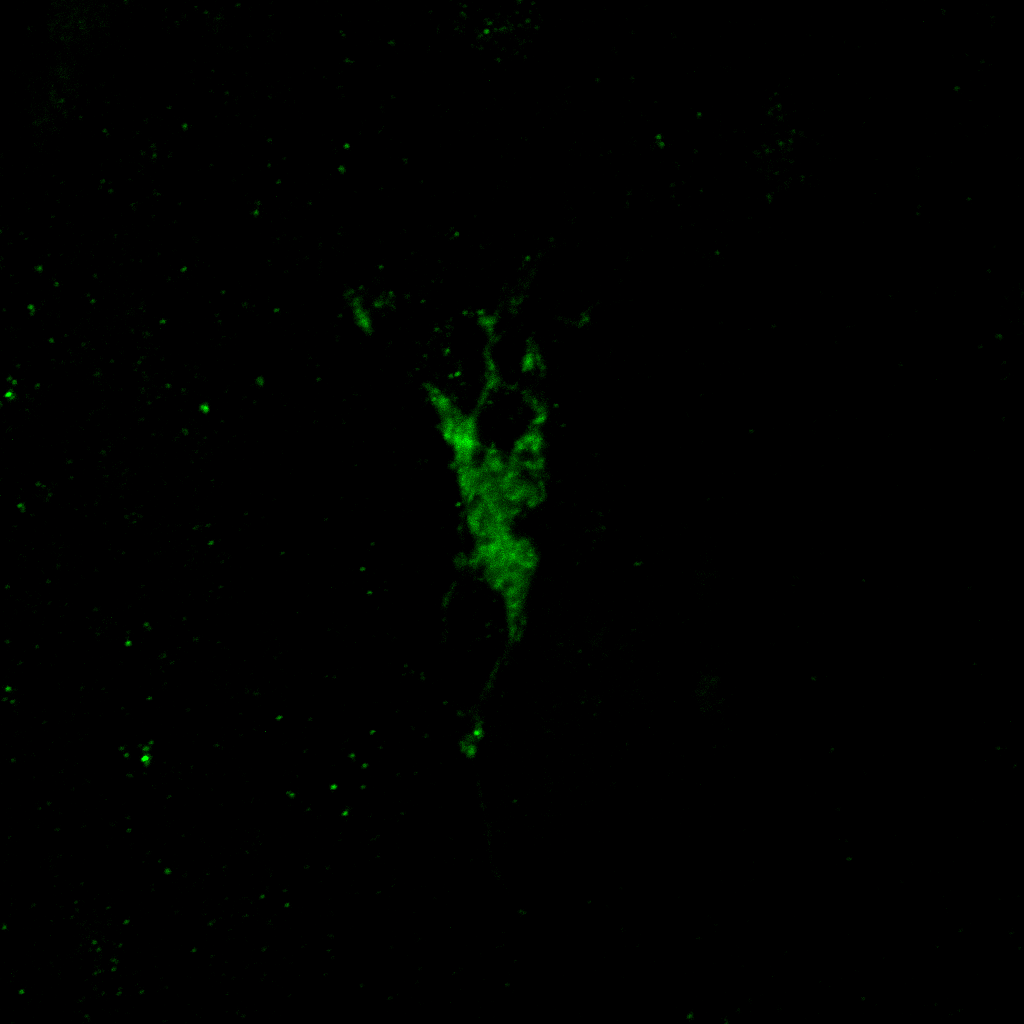

Supplement: Supplementary file 11 — Figure EV2 Source Data [file 44318_2026_817_MOESM11_ESM.zip › EV2F/EV2F-2-C9orf72 KO_GAL3.tif]

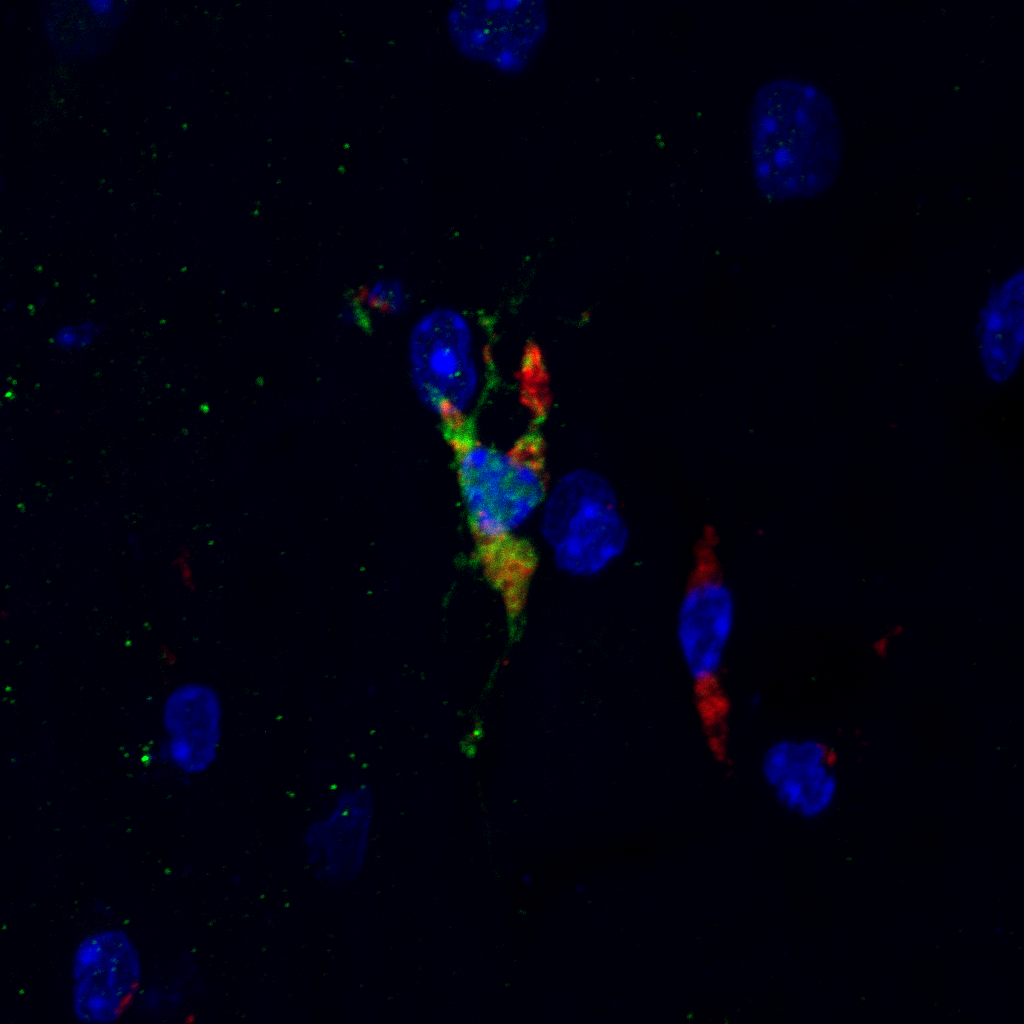

Supplement: Supplementary file 11 — Figure EV2 Source Data [file 44318_2026_817_MOESM11_ESM.zip › EV2F/EV2F-2-C9orf72 KO_Merge.tif]

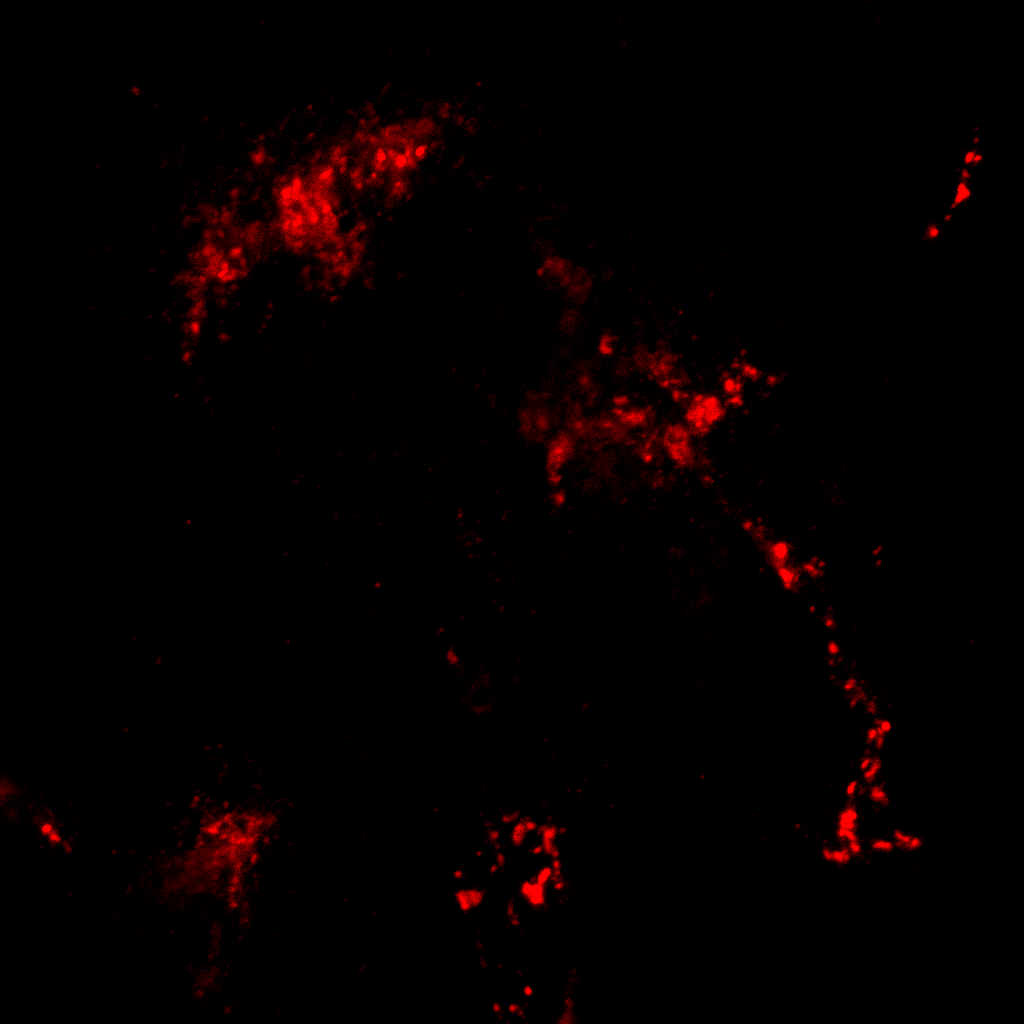

Supplement: Supplementary file 11 — Figure EV2 Source Data [file 44318_2026_817_MOESM11_ESM.zip › EV2F/EV2F-3-Smcr8 KO_CD68.tif]

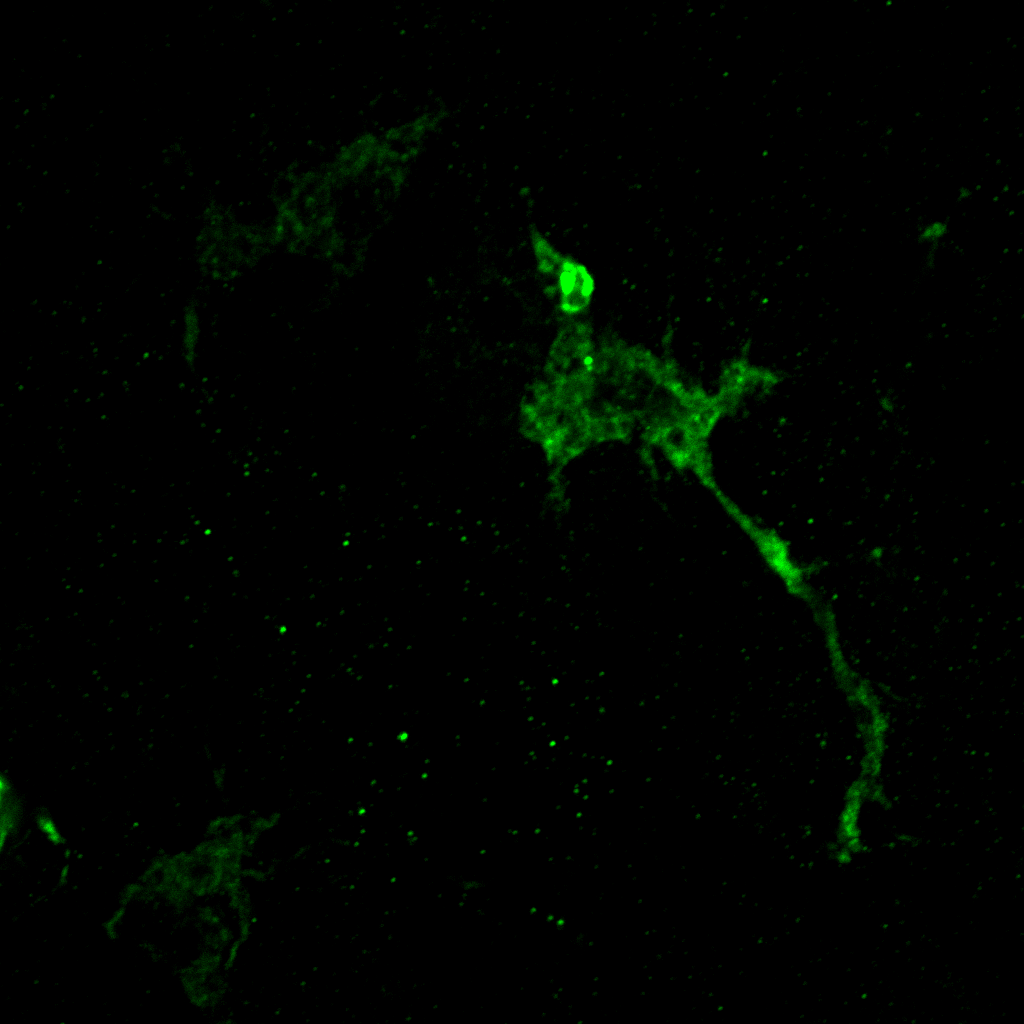

Supplement: Supplementary file 11 — Figure EV2 Source Data [file 44318_2026_817_MOESM11_ESM.zip › EV2F/EV2F-3-Smcr8 KO_GAL3.tif]

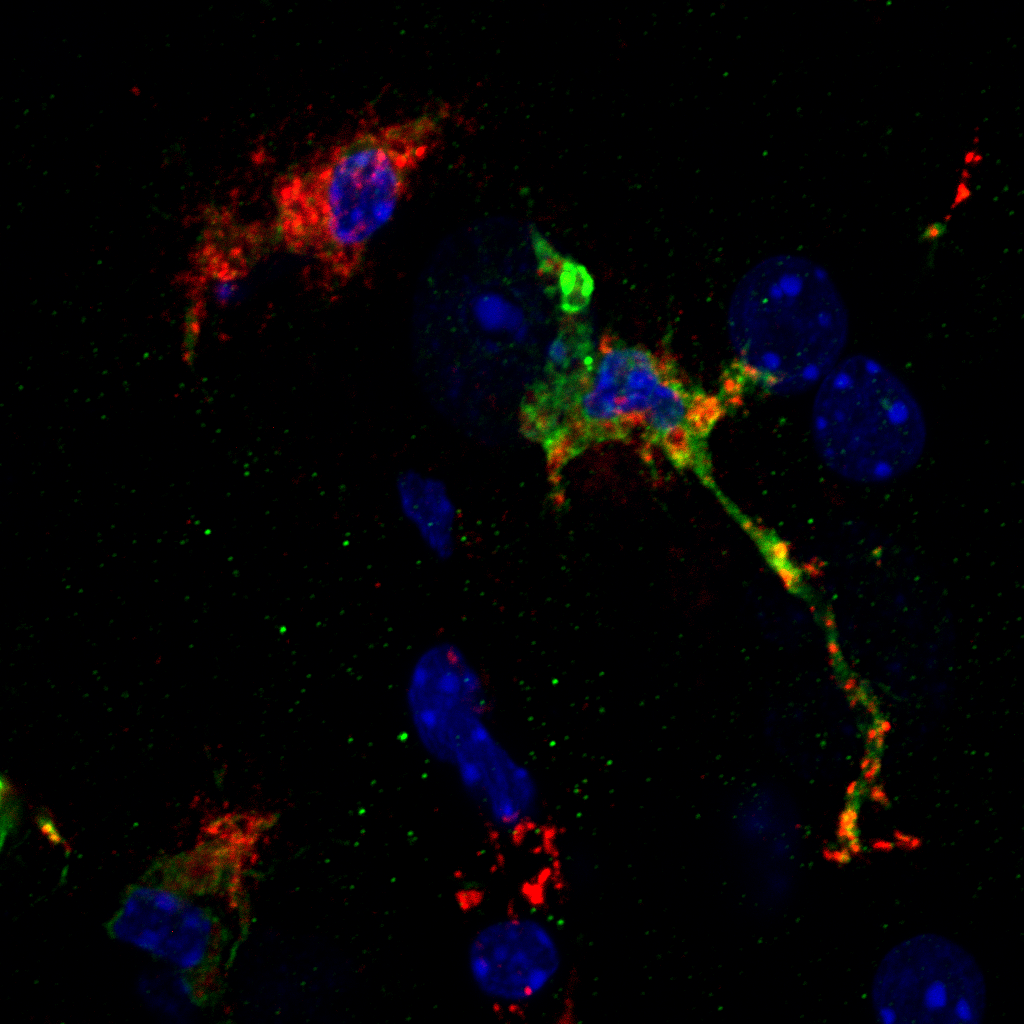

Supplement: Supplementary file 11 — Figure EV2 Source Data [file 44318_2026_817_MOESM11_ESM.zip › EV2F/EV2F-3-Smcr8 KO_Merge.tif]

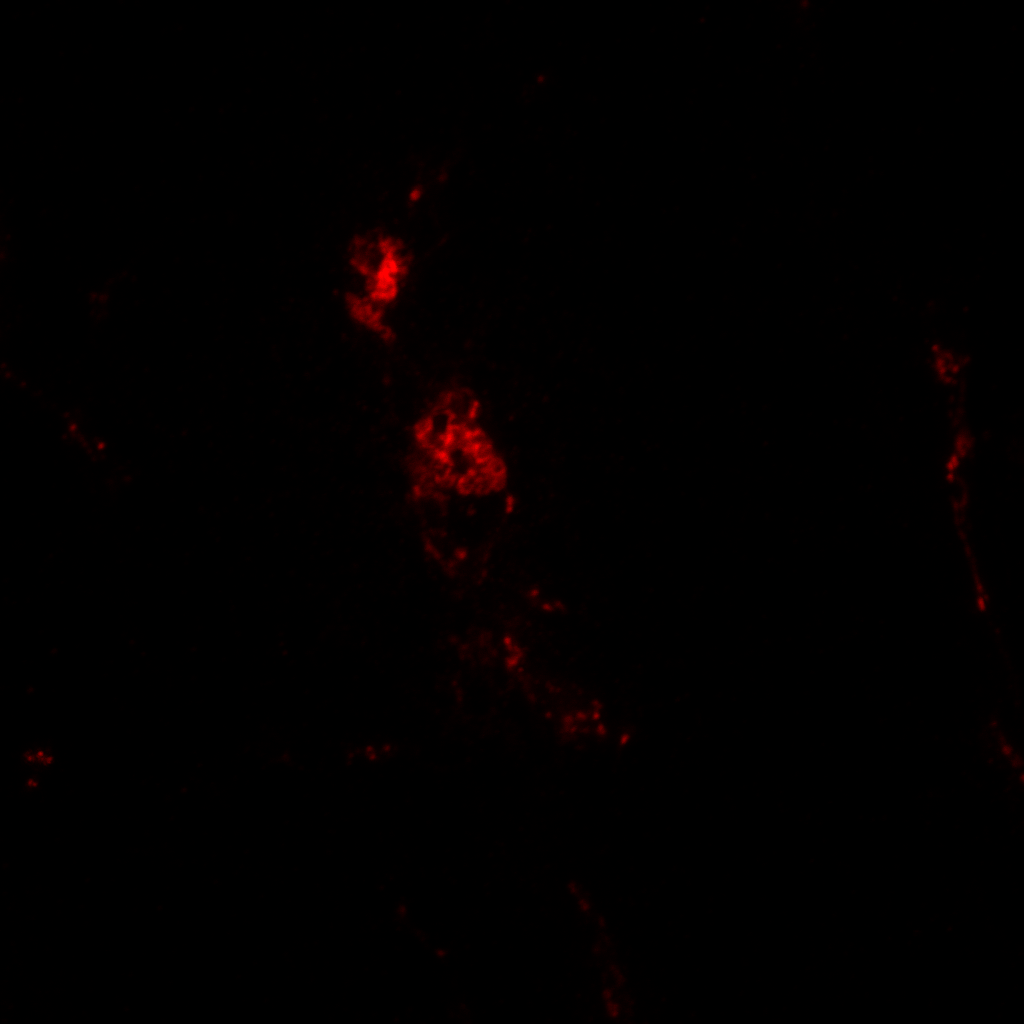

Supplement: Supplementary file 11 — Figure EV2 Source Data [file 44318_2026_817_MOESM11_ESM.zip › EV2F/EV2F-4-dKO_CD68.tif]

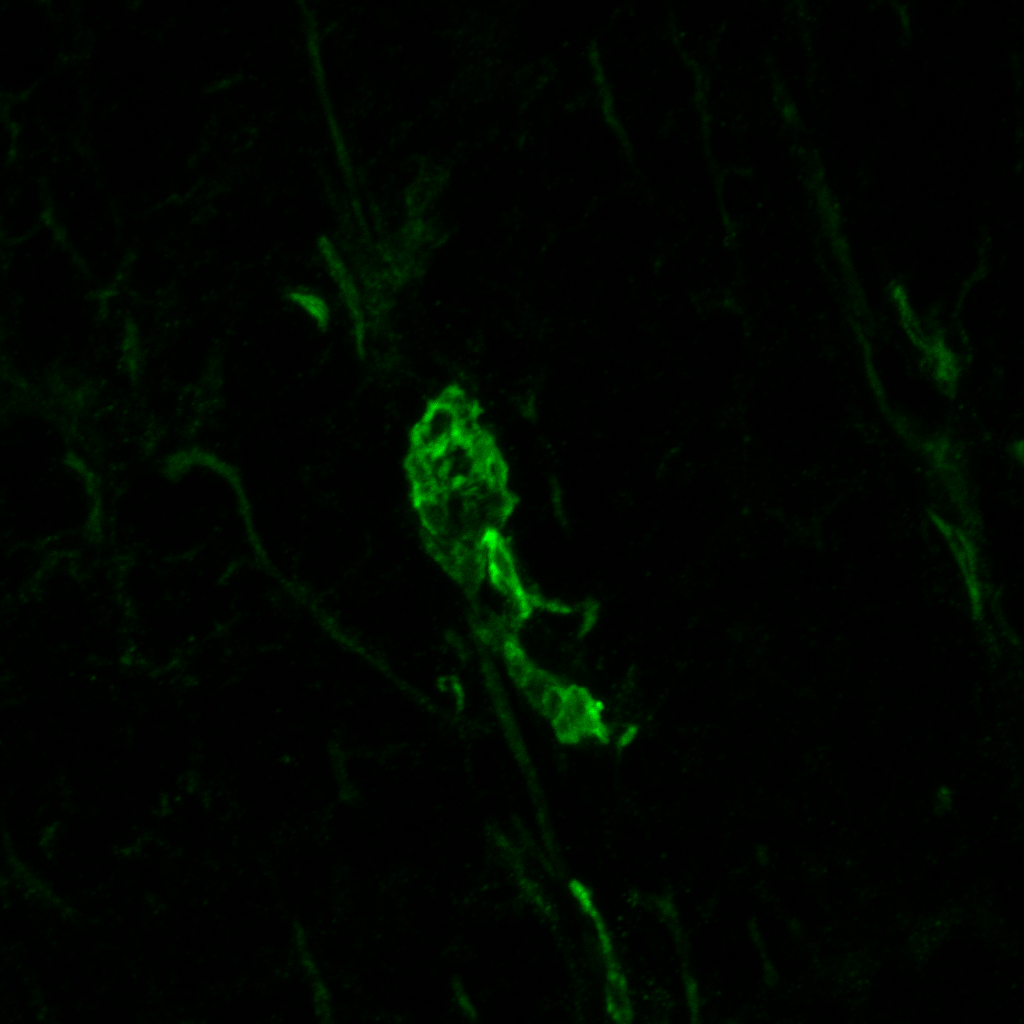

Supplement: Supplementary file 11 — Figure EV2 Source Data [file 44318_2026_817_MOESM11_ESM.zip › EV2F/EV2F-4-dKO_GAL3.tif]
