# Supplementary figures and images for: The C9orf72/SMCR8 complex maintains microglial homeostasis via RAB8A-ESCRT-mediated lysosomal repair (part 3 of 3)
Source: EMBO J. 2026 May 29;45(13):4531–68. doi: 10.1038/s44318-026-00817-w (PMC13324726; doi:10.1038/s44318-026-00817-w)

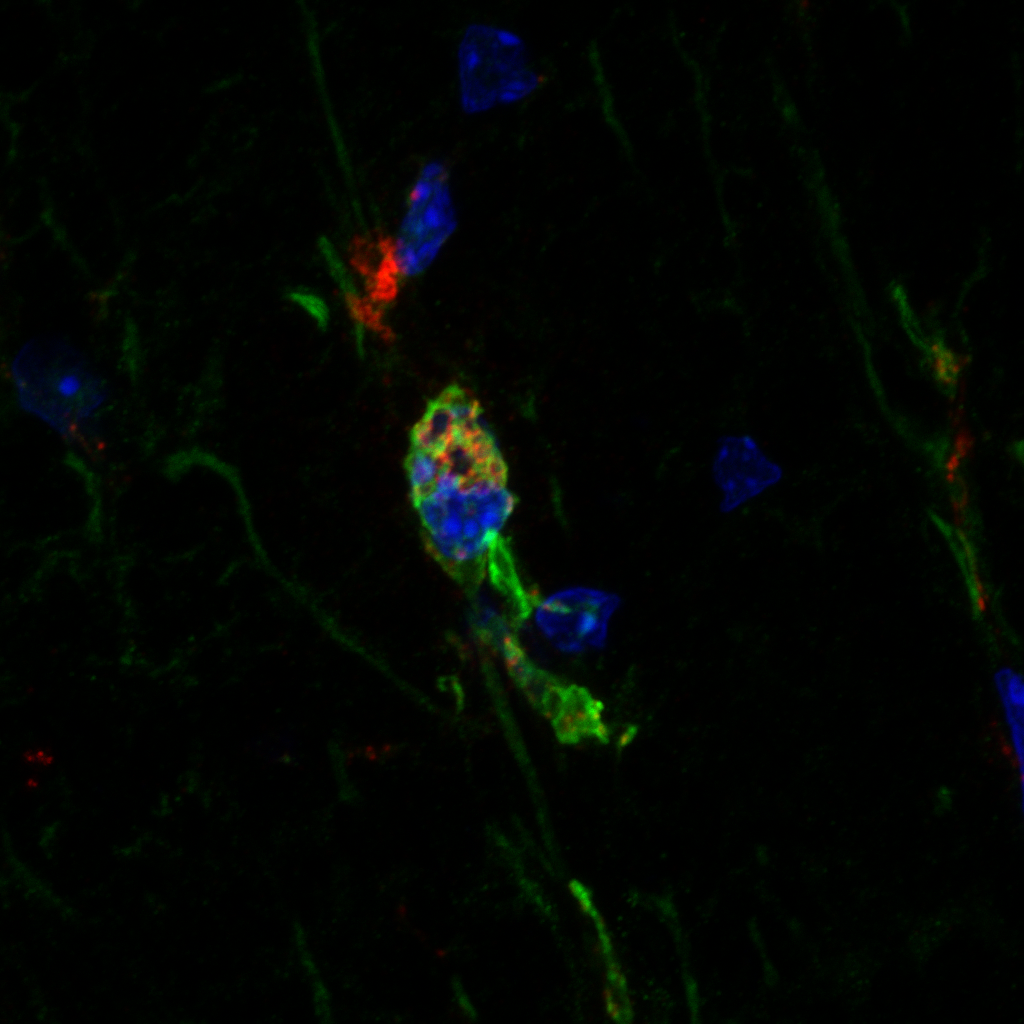

Supplement: Supplementary file 11 — Figure EV2 Source Data [file 44318_2026_817_MOESM11_ESM.zip › EV2F/EV2F-4-dKO_Merge.tif]

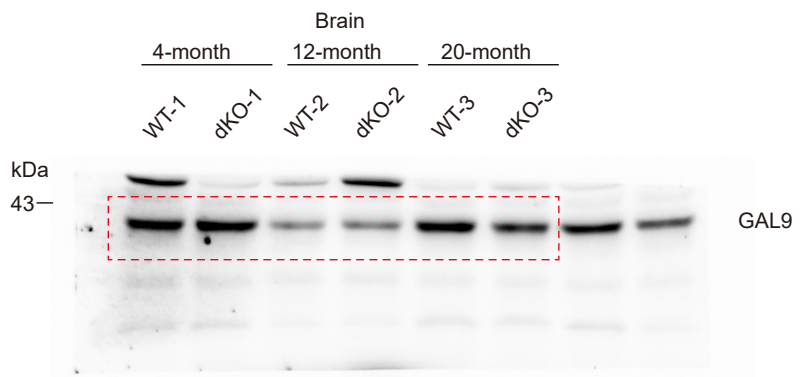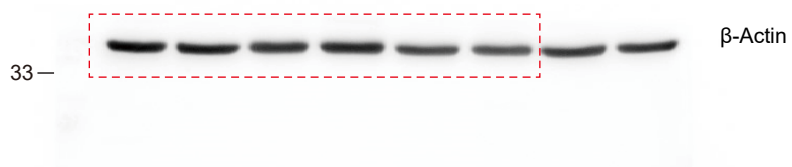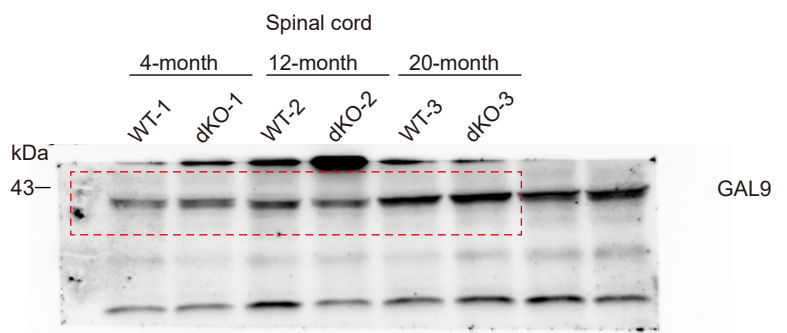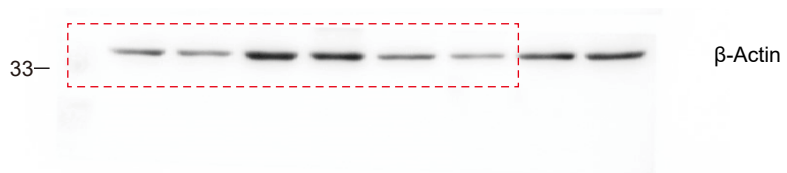

Supplement: Supplementary file 11 — Figure EV2 Source Data [file 44318_2026_817_MOESM11_ESM.zip › EV2H/EV2H.pdf]

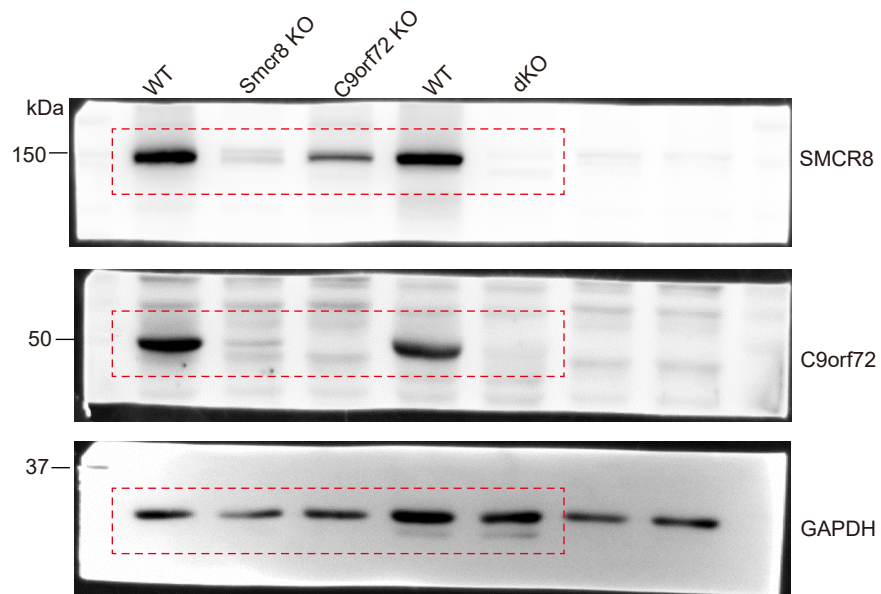

Supplement: Supplementary file 12 — Figure EV3 Source Data [file 44318_2026_817_MOESM12_ESM.zip › EV3B/EV3B.pdf]

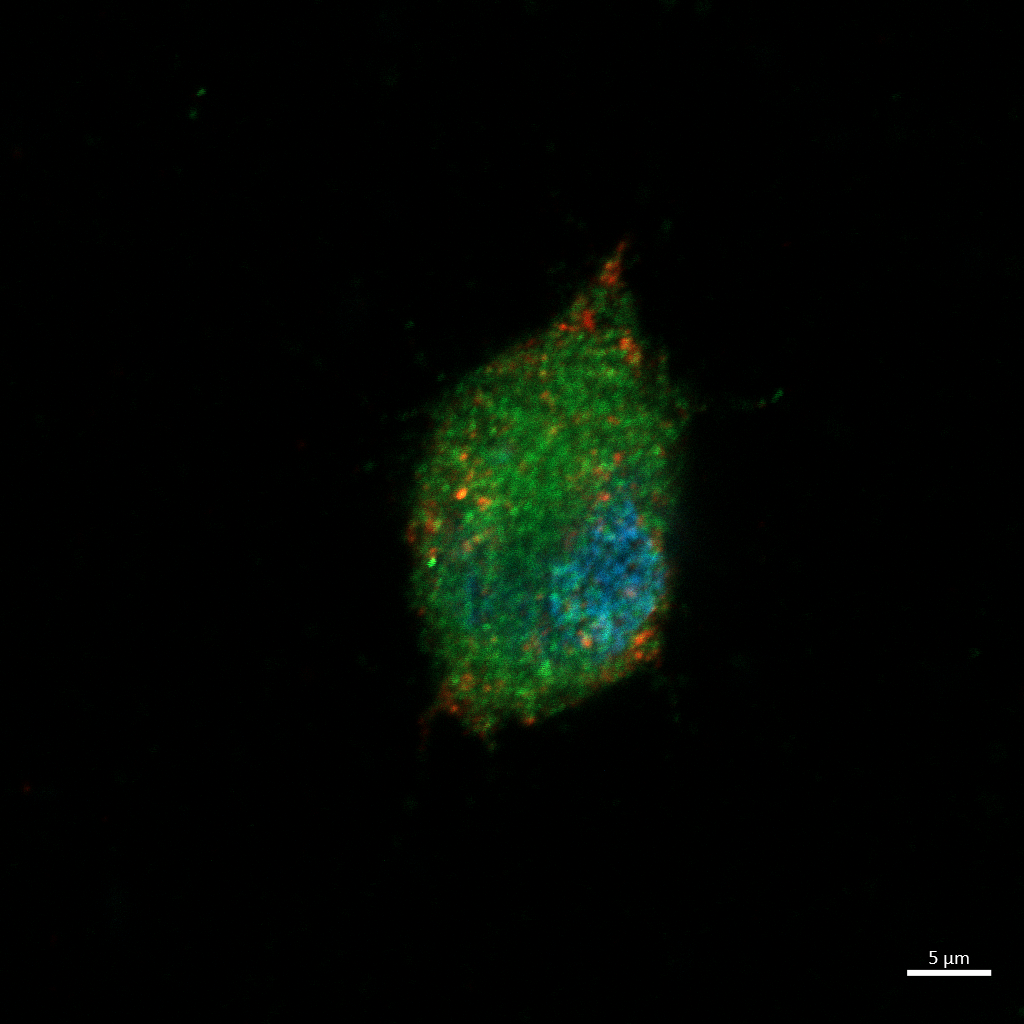

Supplement: Supplementary file 12 — Figure EV3 Source Data [file 44318_2026_817_MOESM12_ESM.zip › EV3C/EV3C-Basal.tif]

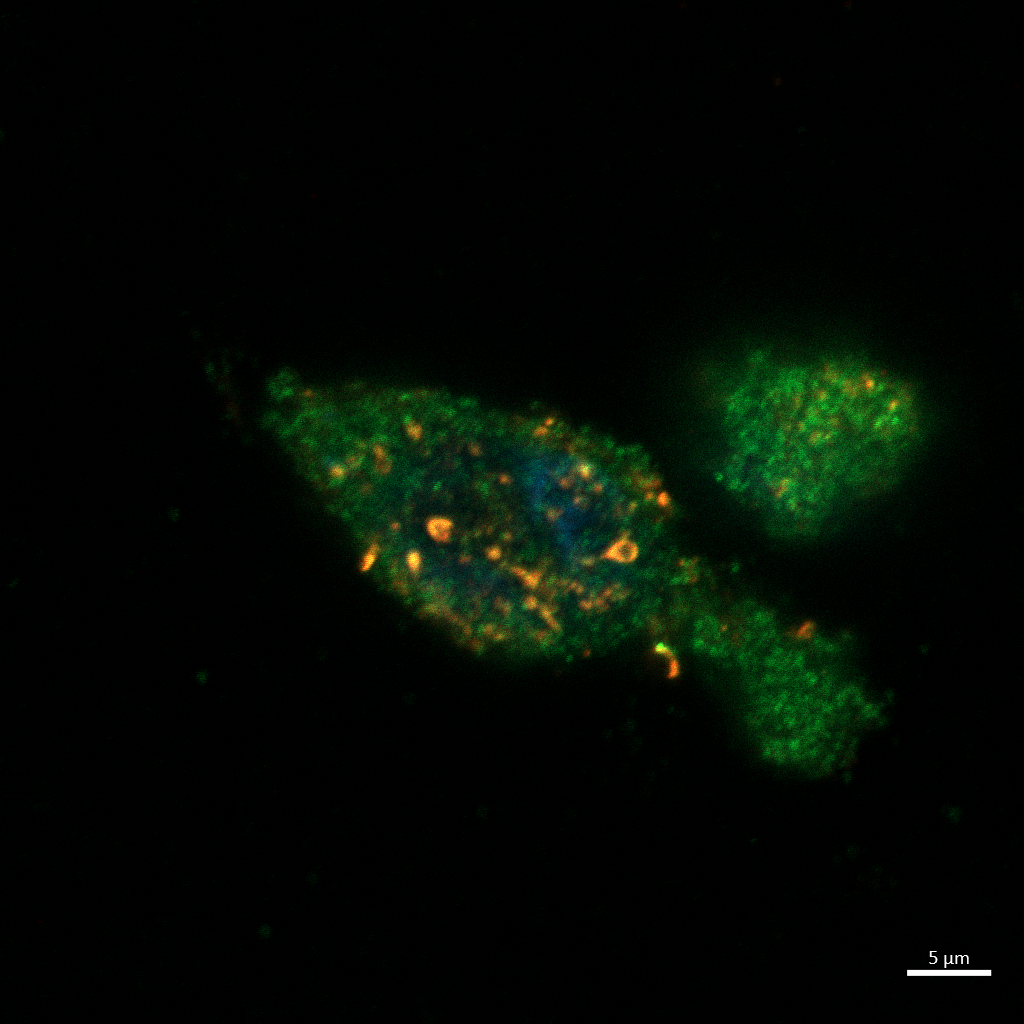

Supplement: Supplementary file 12 — Figure EV3 Source Data [file 44318_2026_817_MOESM12_ESM.zip › EV3C/EV3C-LLOMe 10 min.tif]

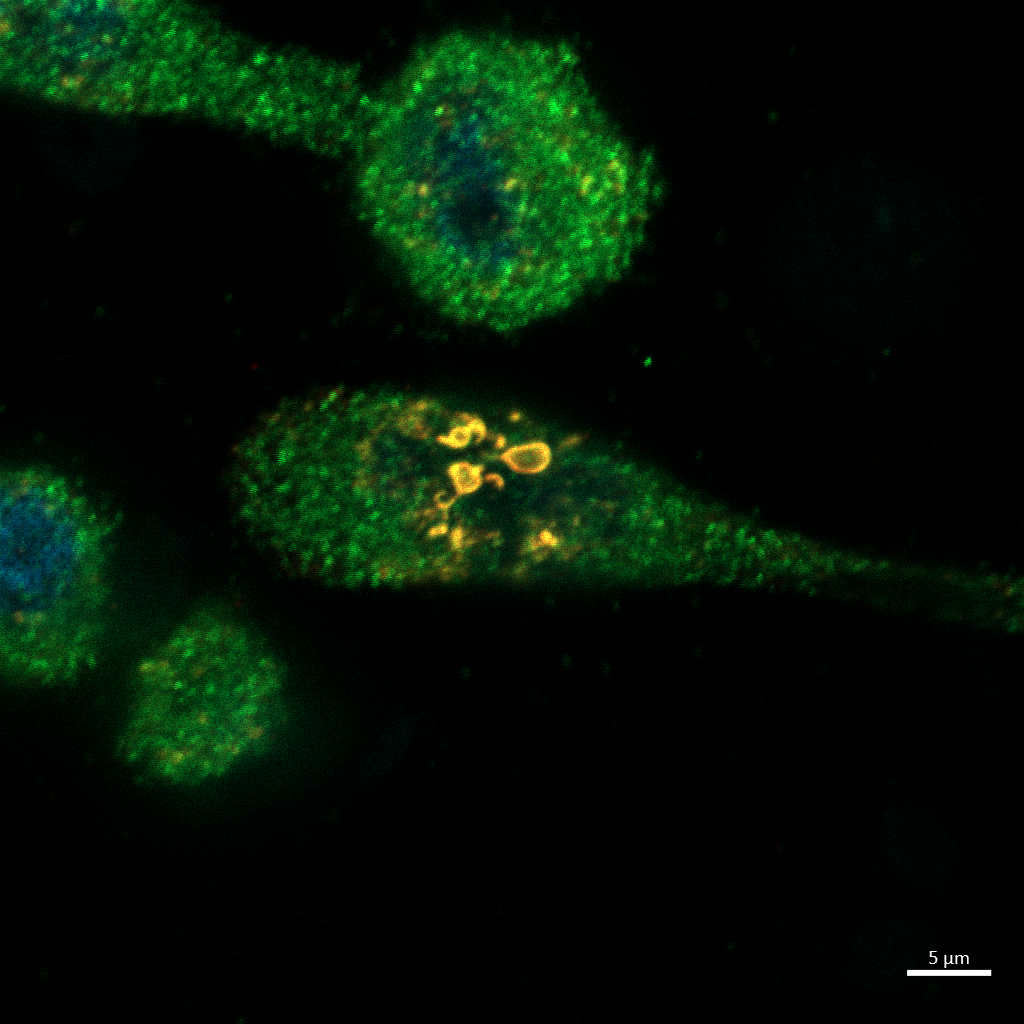

Supplement: Supplementary file 12 — Figure EV3 Source Data [file 44318_2026_817_MOESM12_ESM.zip › EV3C/EV3C-LLOMe 30 min.tif]

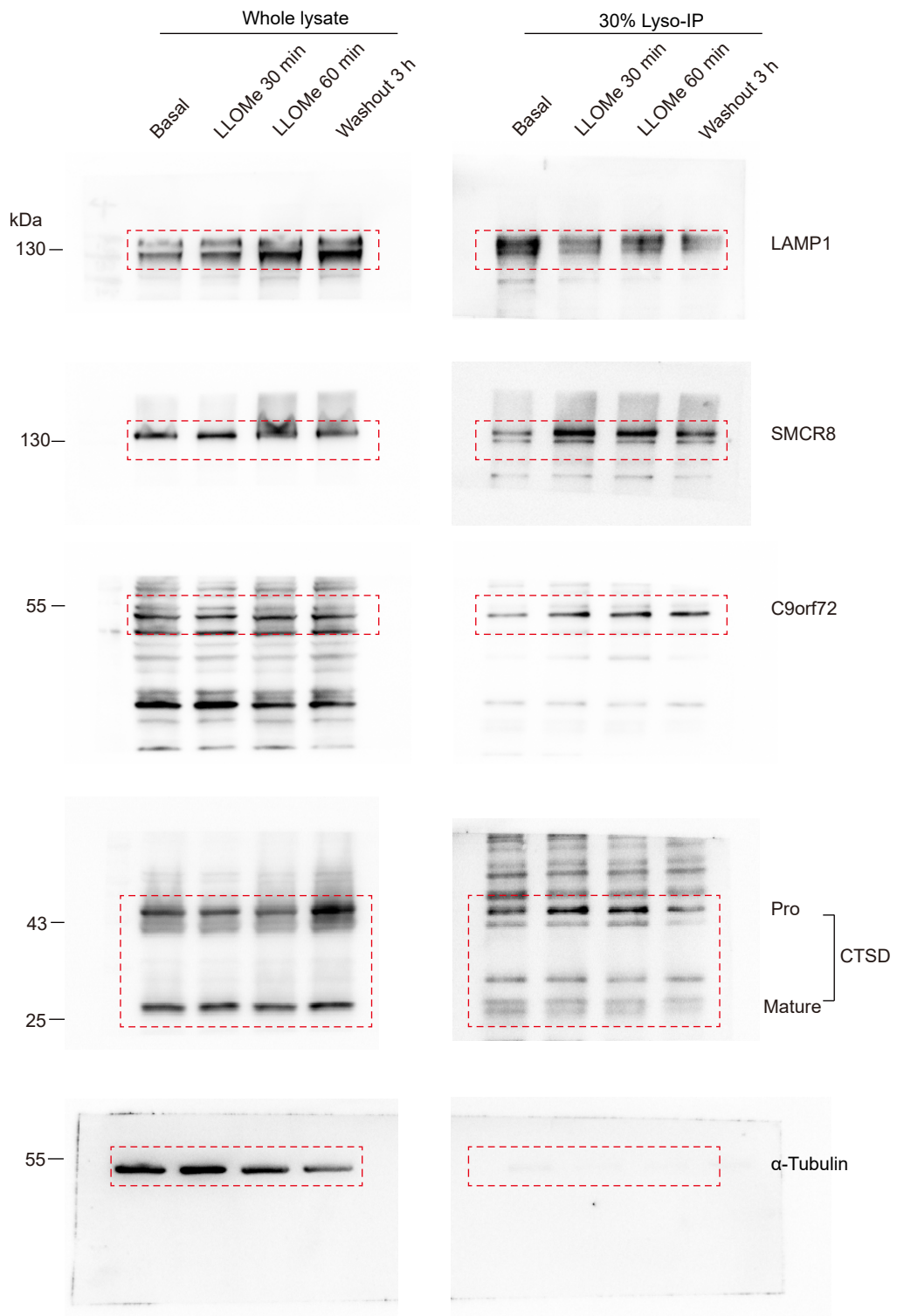

Supplement: Supplementary file 12 — Figure EV3 Source Data [file 44318_2026_817_MOESM12_ESM.zip › EV3D/EV3D.pdf]

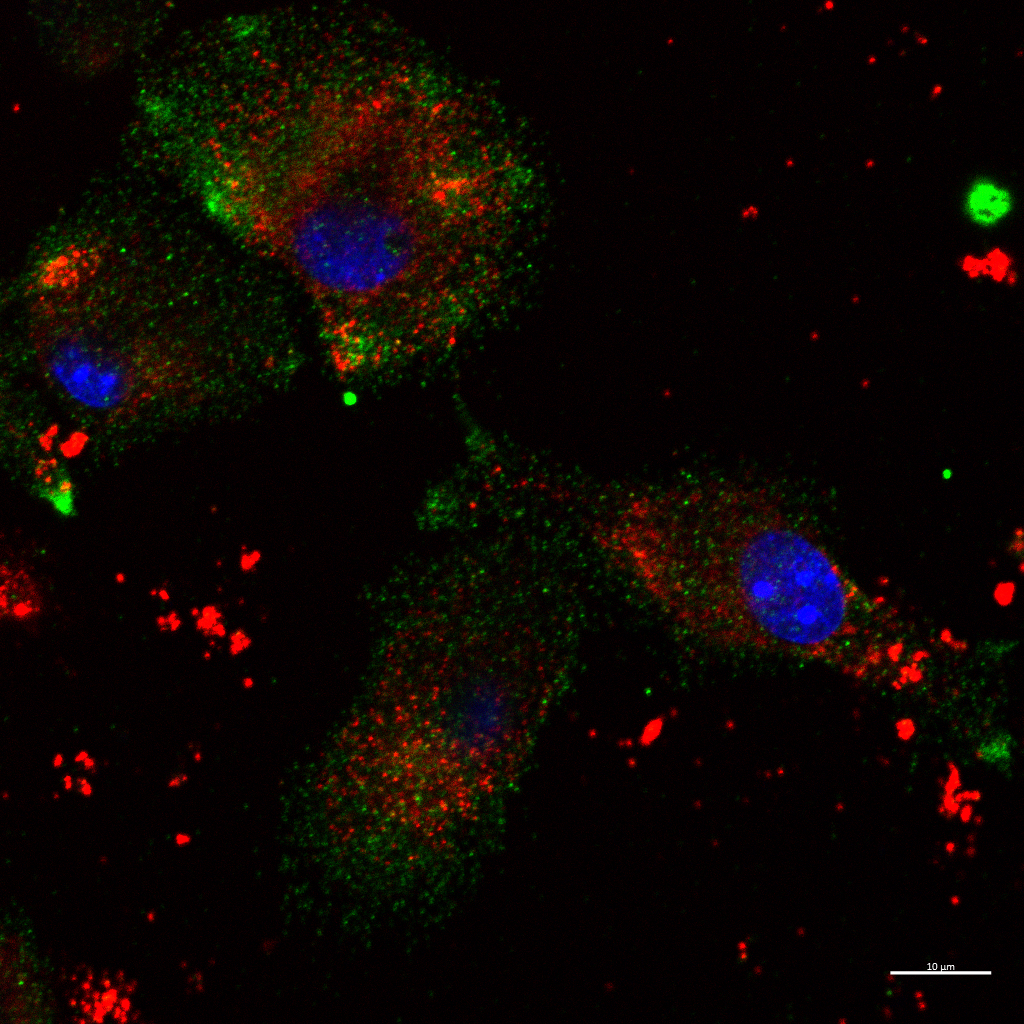

Supplement: Supplementary file 12 — Figure EV3 Source Data [file 44318_2026_817_MOESM12_ESM.zip › EV3F/EV3F-1-Basal-C9orf72 KO.tif]

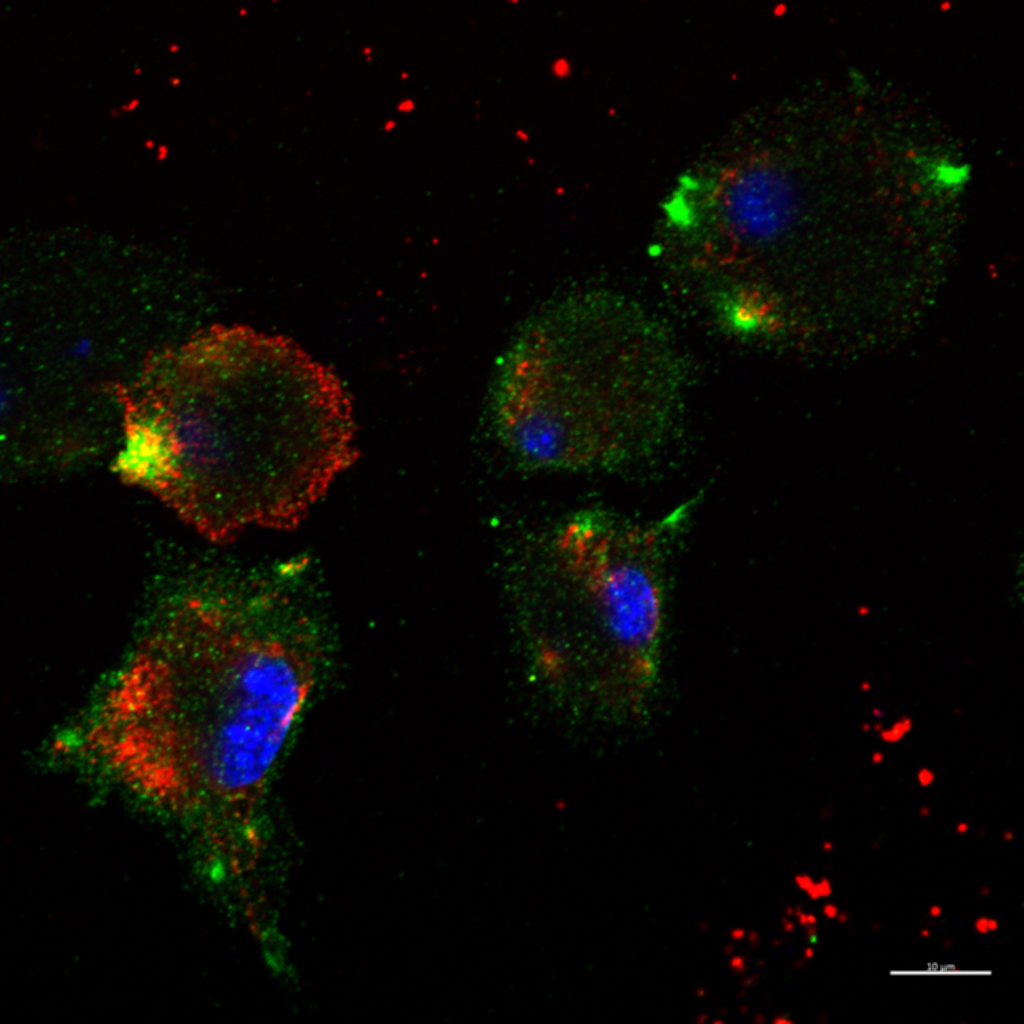

Supplement: Supplementary file 12 — Figure EV3 Source Data [file 44318_2026_817_MOESM12_ESM.zip › EV3F/EV3F-1-Basal-dKO.tif]

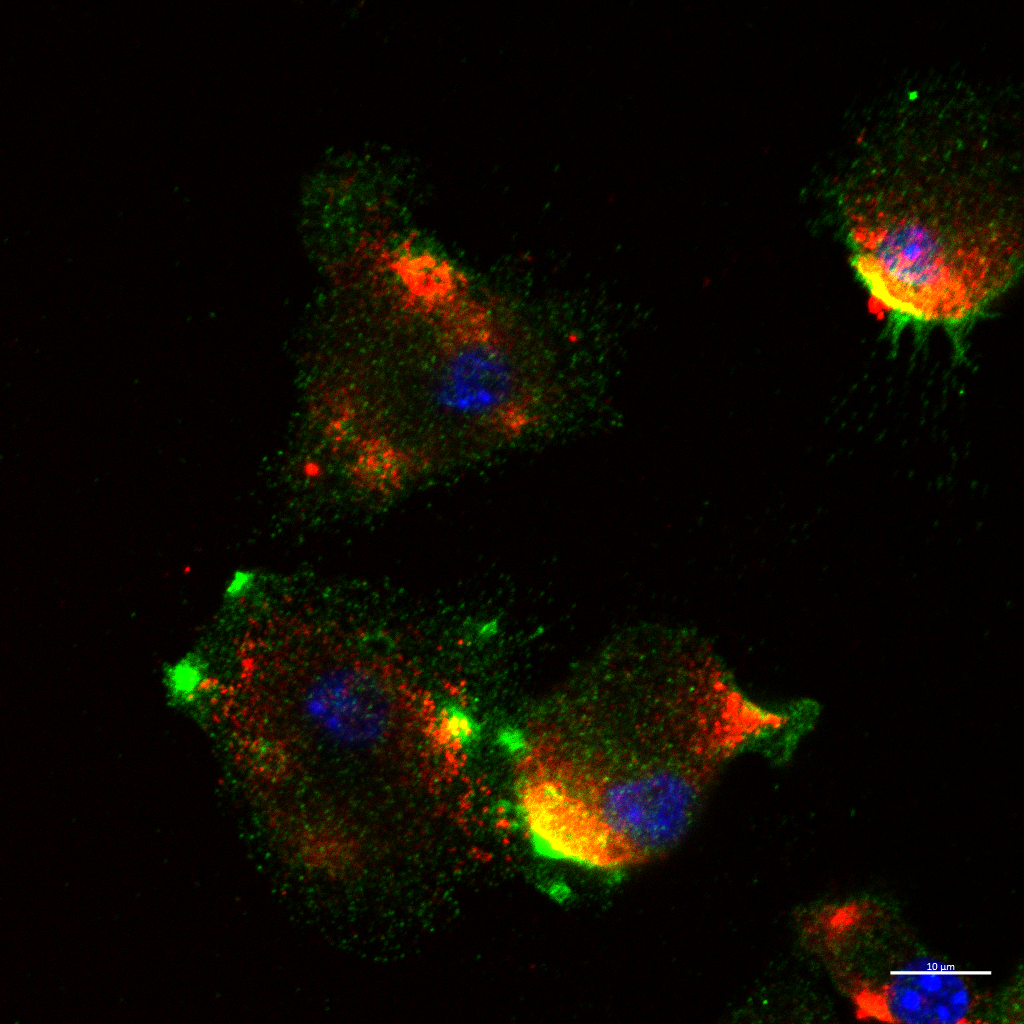

Supplement: Supplementary file 12 — Figure EV3 Source Data [file 44318_2026_817_MOESM12_ESM.zip › EV3F/EV3F-1-Basal-Smcr8 KO.tif]

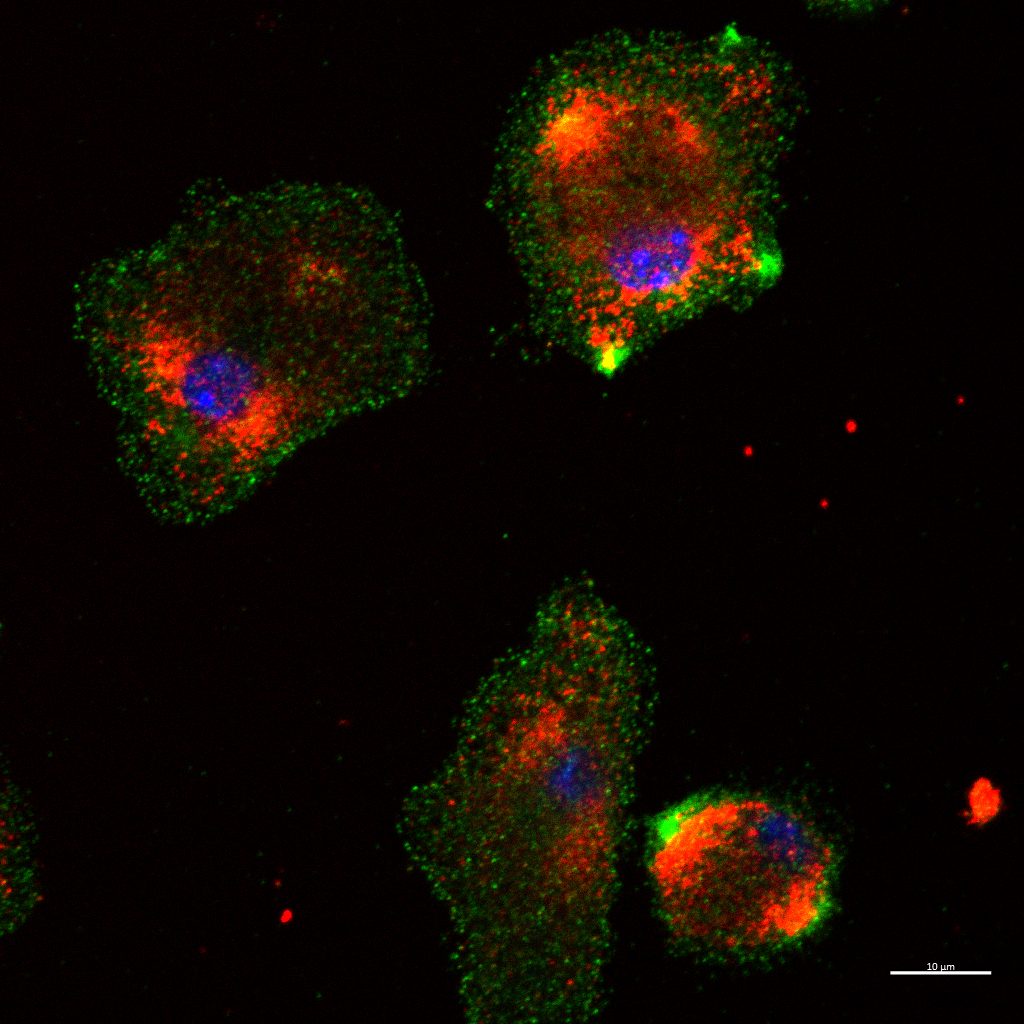

Supplement: Supplementary file 12 — Figure EV3 Source Data [file 44318_2026_817_MOESM12_ESM.zip › EV3F/EV3F-1-Basal-WT.tif]

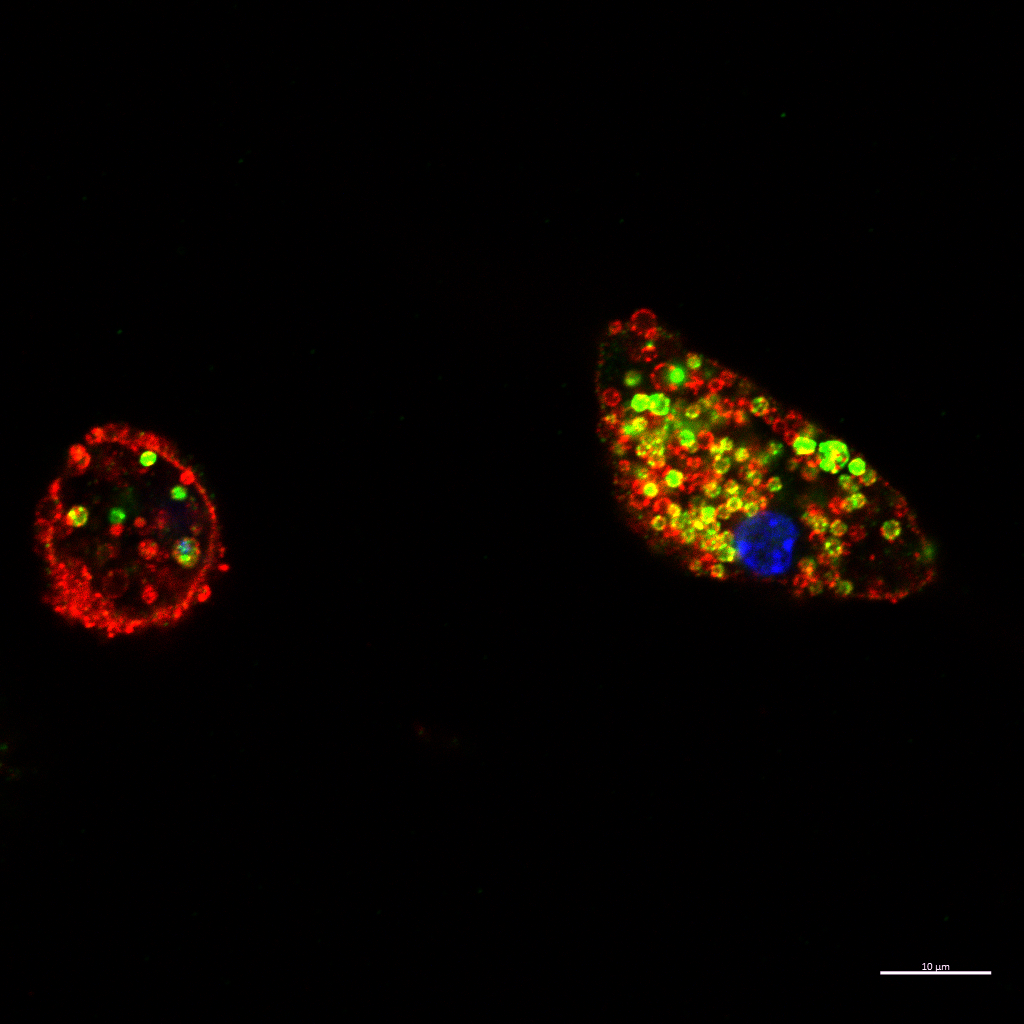

Supplement: Supplementary file 12 — Figure EV3 Source Data [file 44318_2026_817_MOESM12_ESM.zip › EV3F/EV3F-2-LLOMe 30 min-C9orf72 KO.tif]

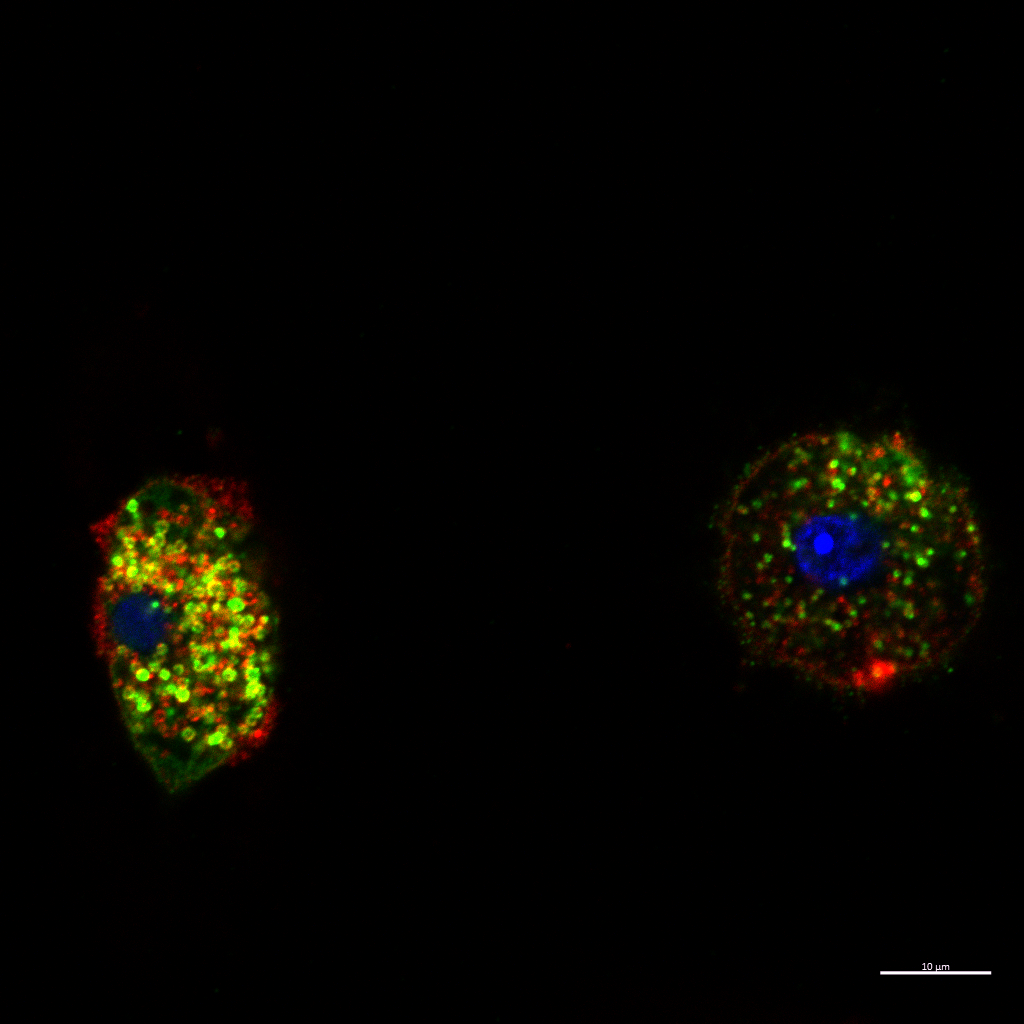

Supplement: Supplementary file 12 — Figure EV3 Source Data [file 44318_2026_817_MOESM12_ESM.zip › EV3F/EV3F-2-LLOMe 30 min-dKO.tif]

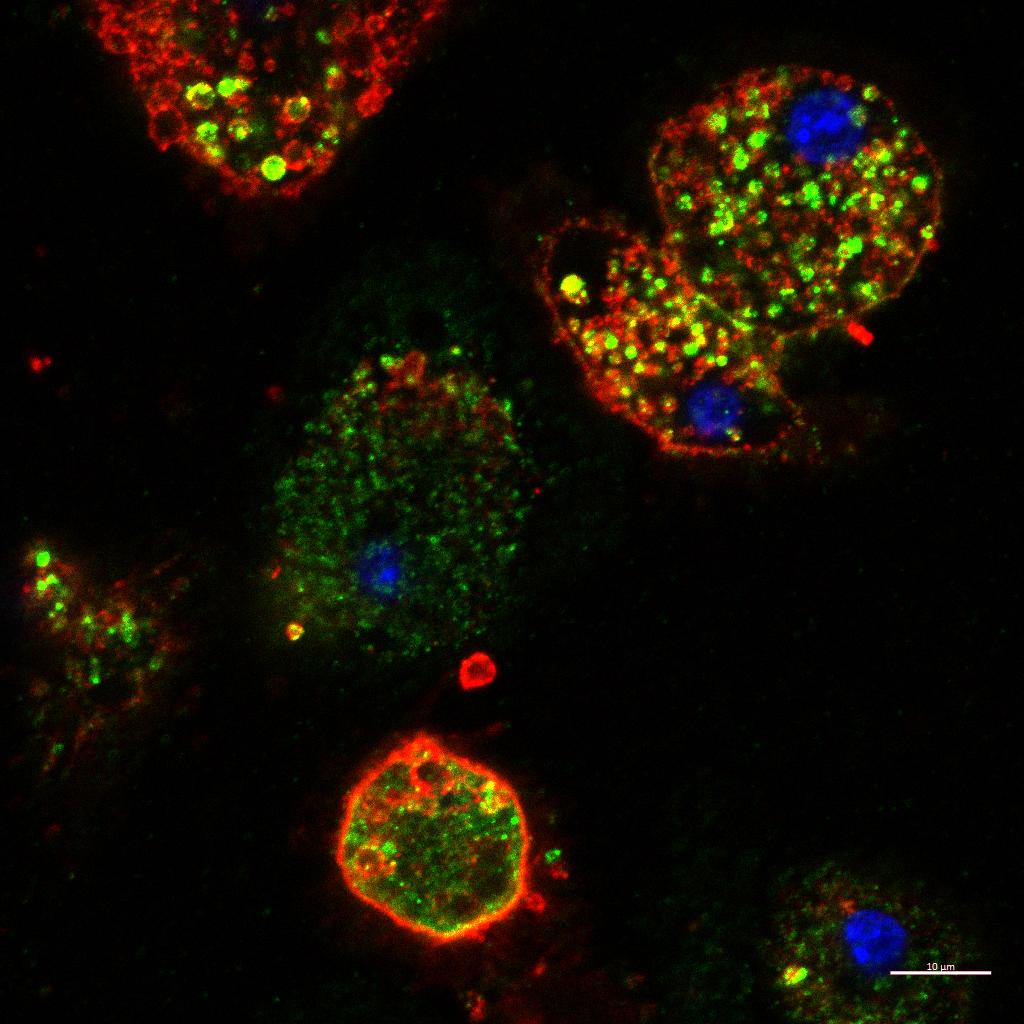

Supplement: Supplementary file 12 — Figure EV3 Source Data [file 44318_2026_817_MOESM12_ESM.zip › EV3F/EV3F-2-LLOMe 30 min-Smcr8 KO.tif]

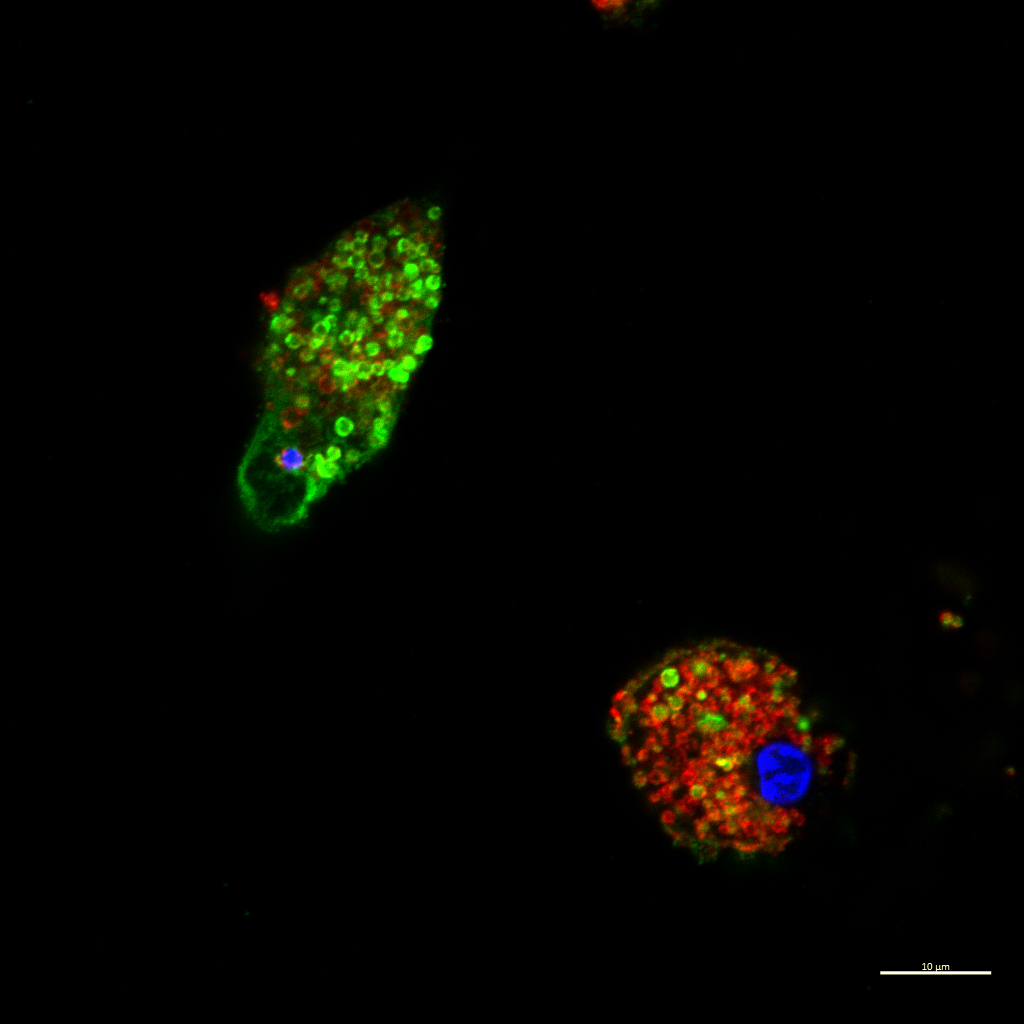

Supplement: Supplementary file 12 — Figure EV3 Source Data [file 44318_2026_817_MOESM12_ESM.zip › EV3F/EV3F-2-LLOMe 30 min-WT.tif]

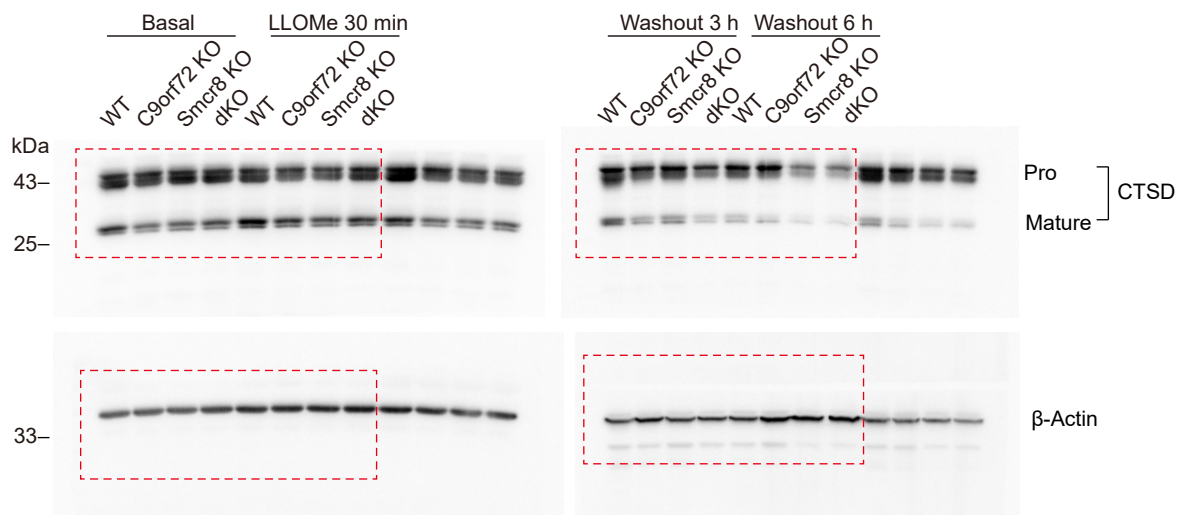

Supplement: Supplementary file 12 — Figure EV3 Source Data [file 44318_2026_817_MOESM12_ESM.zip › EV3H/EV3H.pdf]

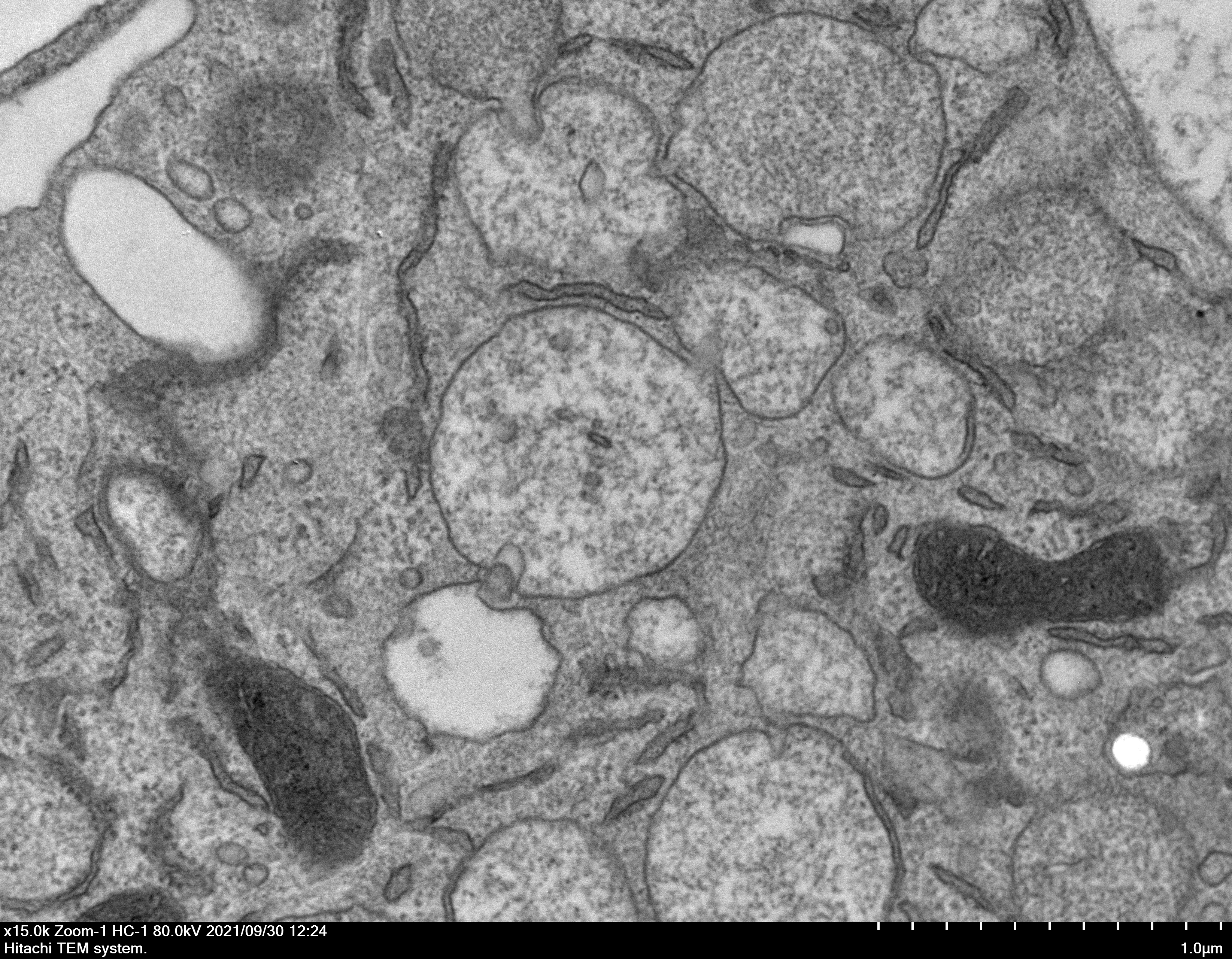

Supplement: Supplementary file 12 — Figure EV3 Source Data [file 44318_2026_817_MOESM12_ESM.zip › EV3I/EV3I-1-Basal-C9orf72 KO_TEM.tif]

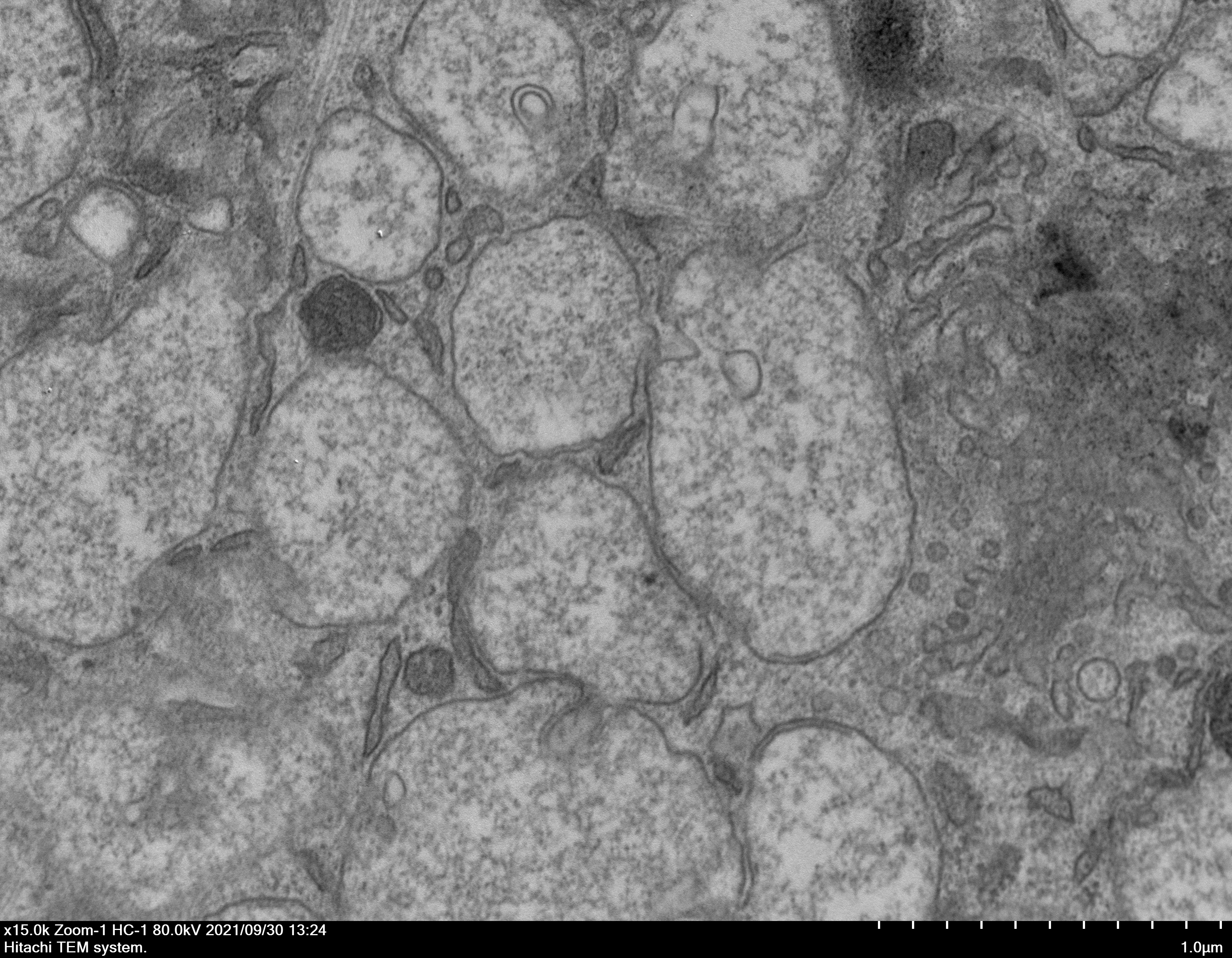

Supplement: Supplementary file 12 — Figure EV3 Source Data [file 44318_2026_817_MOESM12_ESM.zip › EV3I/EV3I-1-Basal-dKO_TEM.tif]

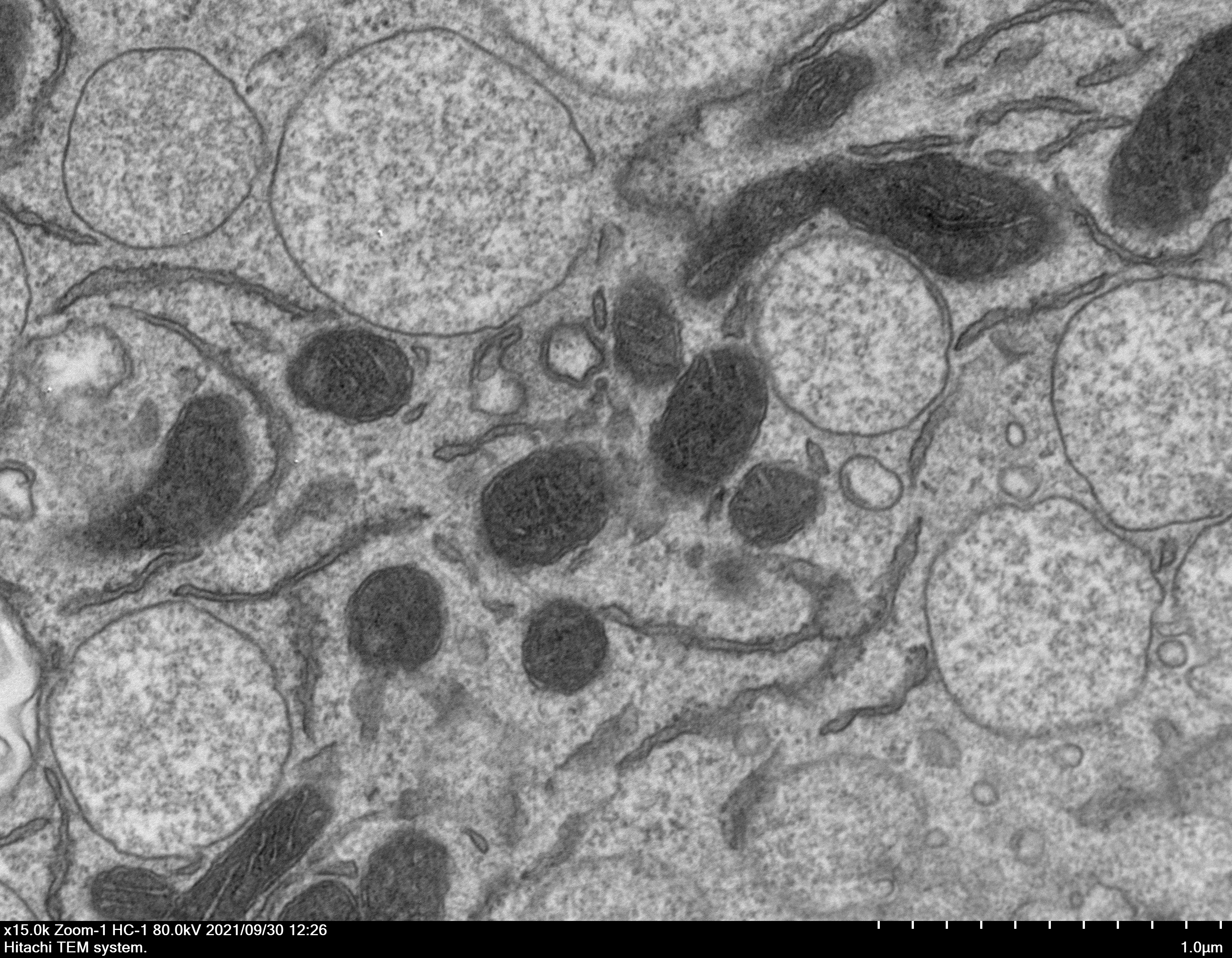

Supplement: Supplementary file 12 — Figure EV3 Source Data [file 44318_2026_817_MOESM12_ESM.zip › EV3I/EV3I-1-Basal-Smcr8 KO_TEM.tif]

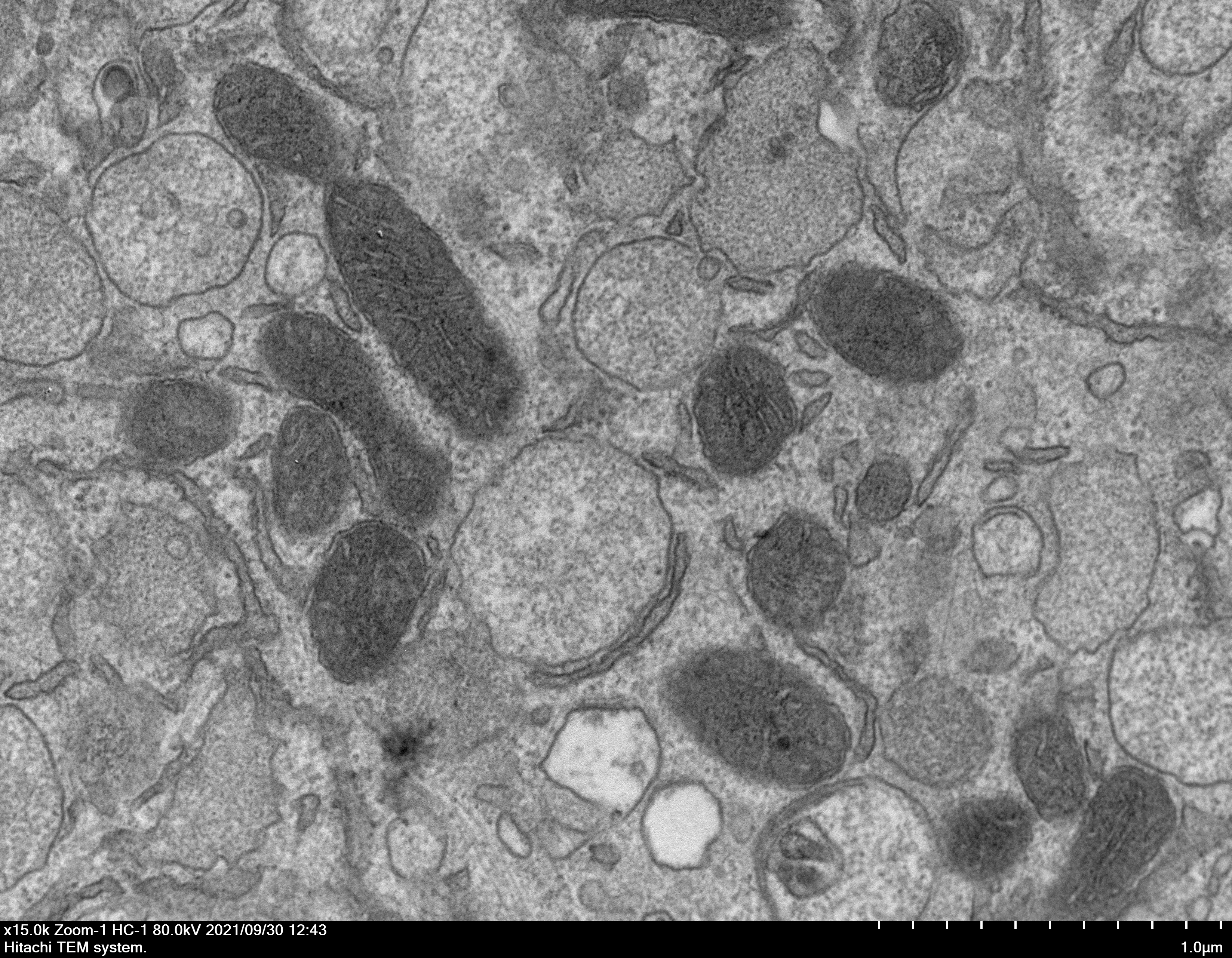

Supplement: Supplementary file 12 — Figure EV3 Source Data [file 44318_2026_817_MOESM12_ESM.zip › EV3I/EV3I-1-Basal-WT_TEM.tif]

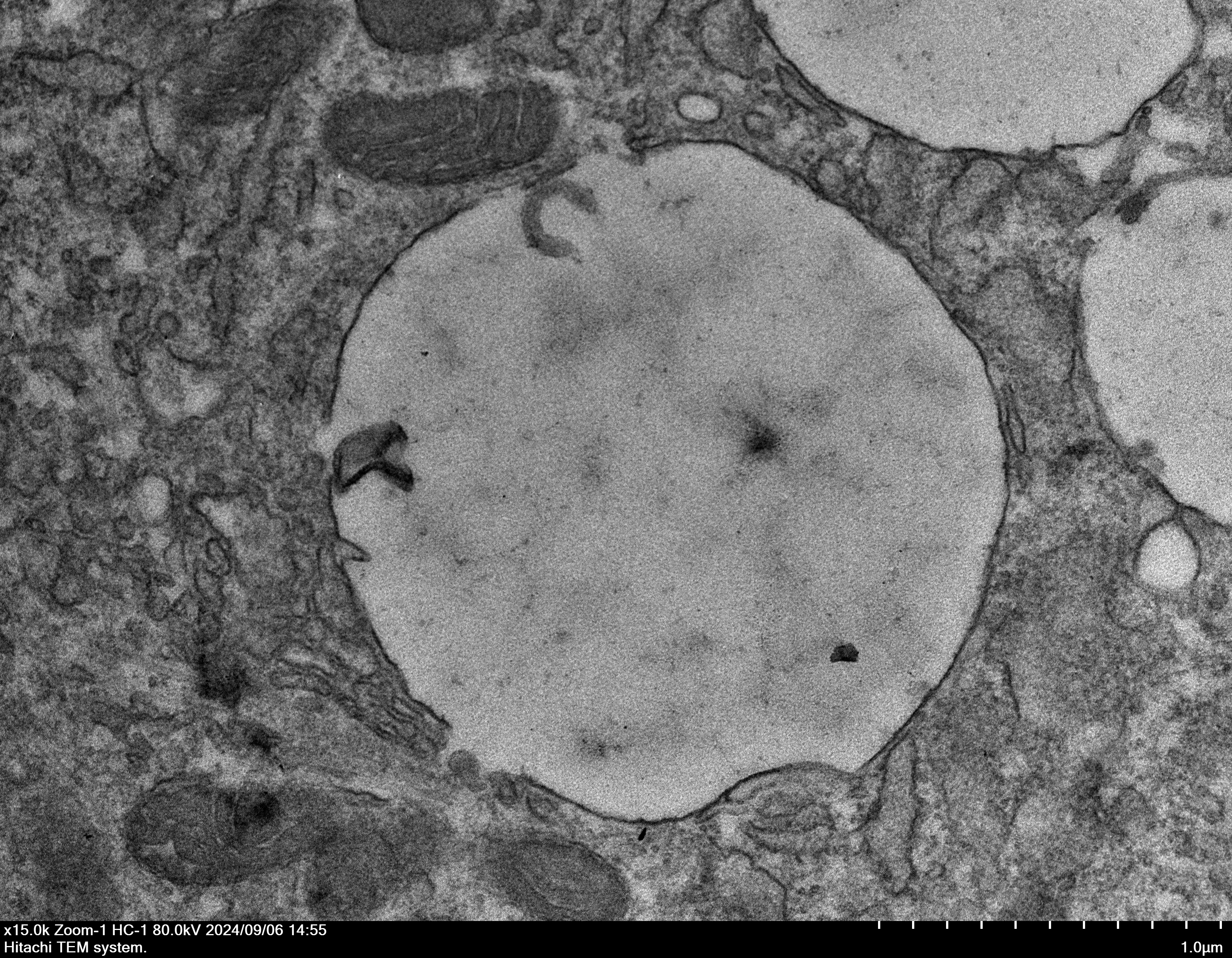

Supplement: Supplementary file 12 — Figure EV3 Source Data [file 44318_2026_817_MOESM12_ESM.zip › EV3I/EV3I-2-LLOMe-C9orf72 KO_TEM.tif]

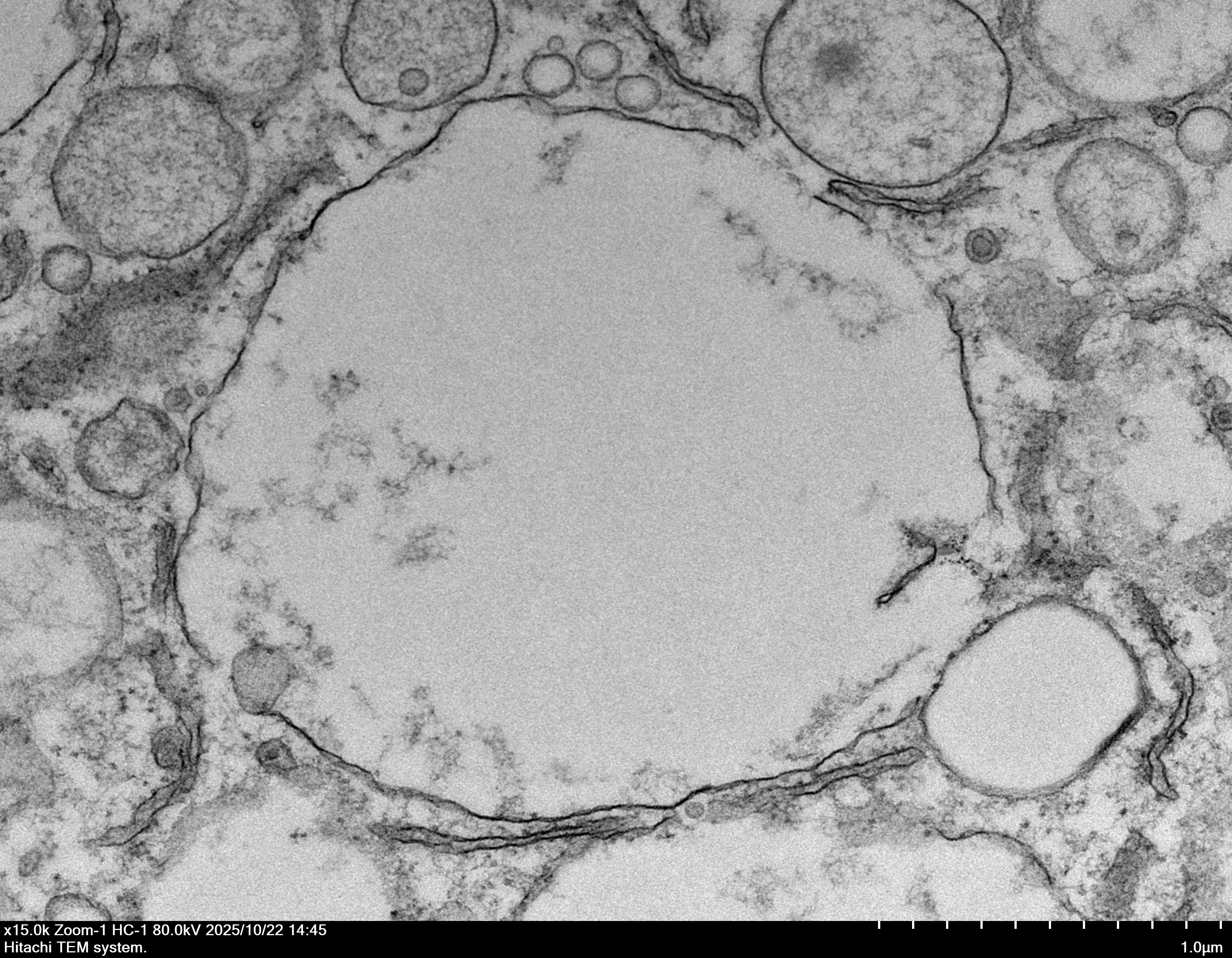

Supplement: Supplementary file 12 — Figure EV3 Source Data [file 44318_2026_817_MOESM12_ESM.zip › EV3I/EV3I-2-LLOMe-dKO_TEM.tif]

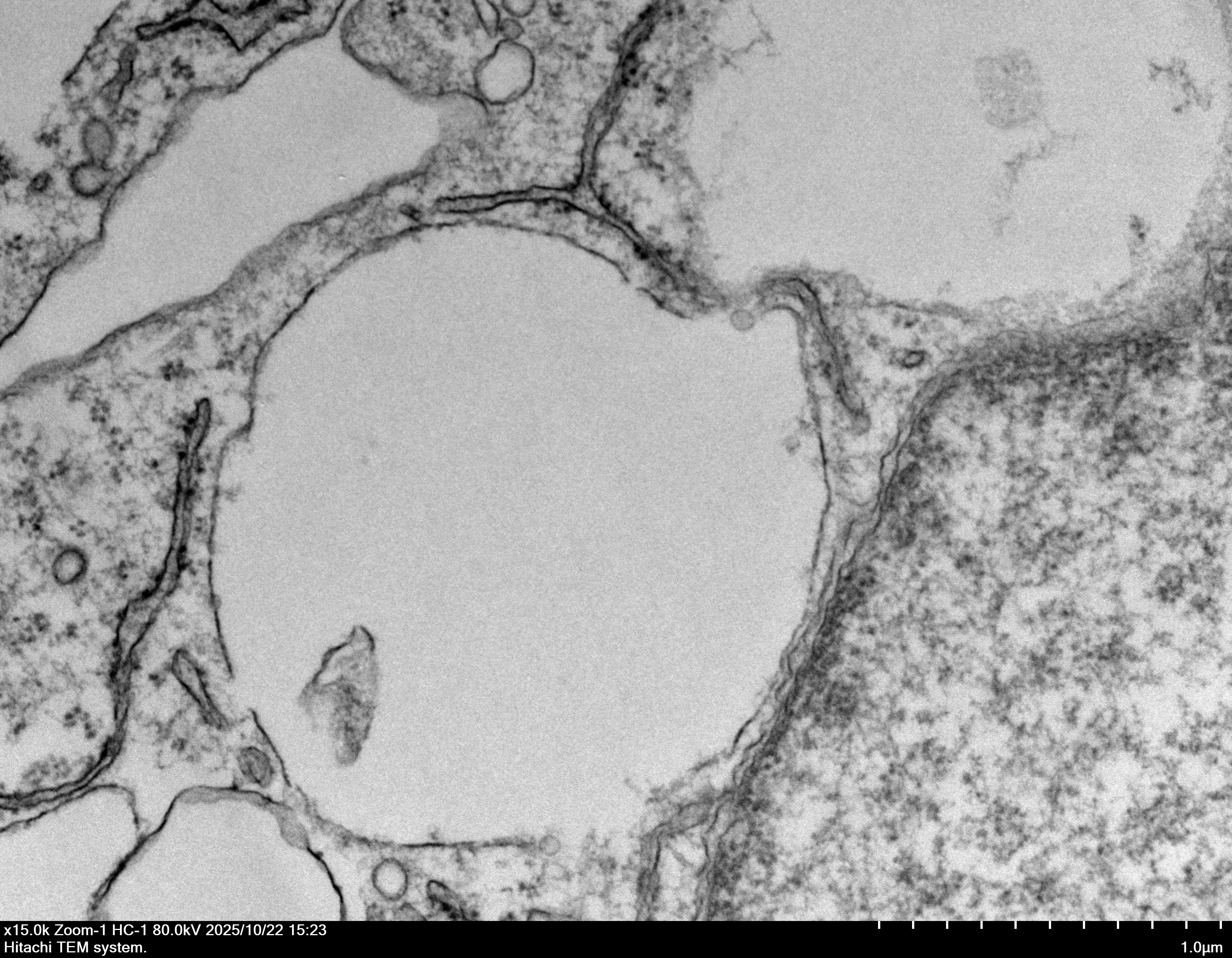

Supplement: Supplementary file 12 — Figure EV3 Source Data [file 44318_2026_817_MOESM12_ESM.zip › EV3I/EV3I-2-LLOMe-Smcr8 KO_TEM.tif]

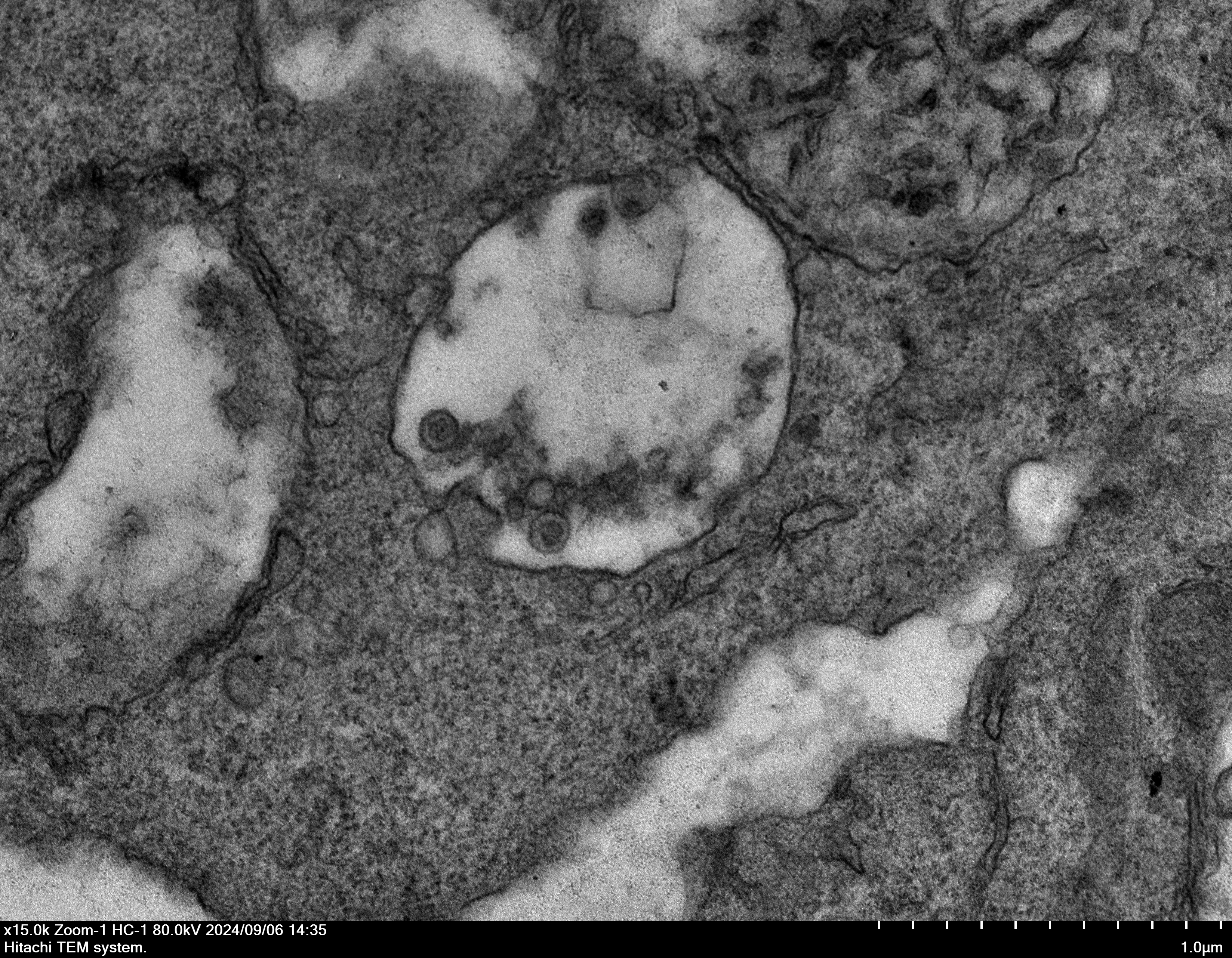

Supplement: Supplementary file 12 — Figure EV3 Source Data [file 44318_2026_817_MOESM12_ESM.zip › EV3I/EV3I-2-LLOMe-WT_TEM.tif]

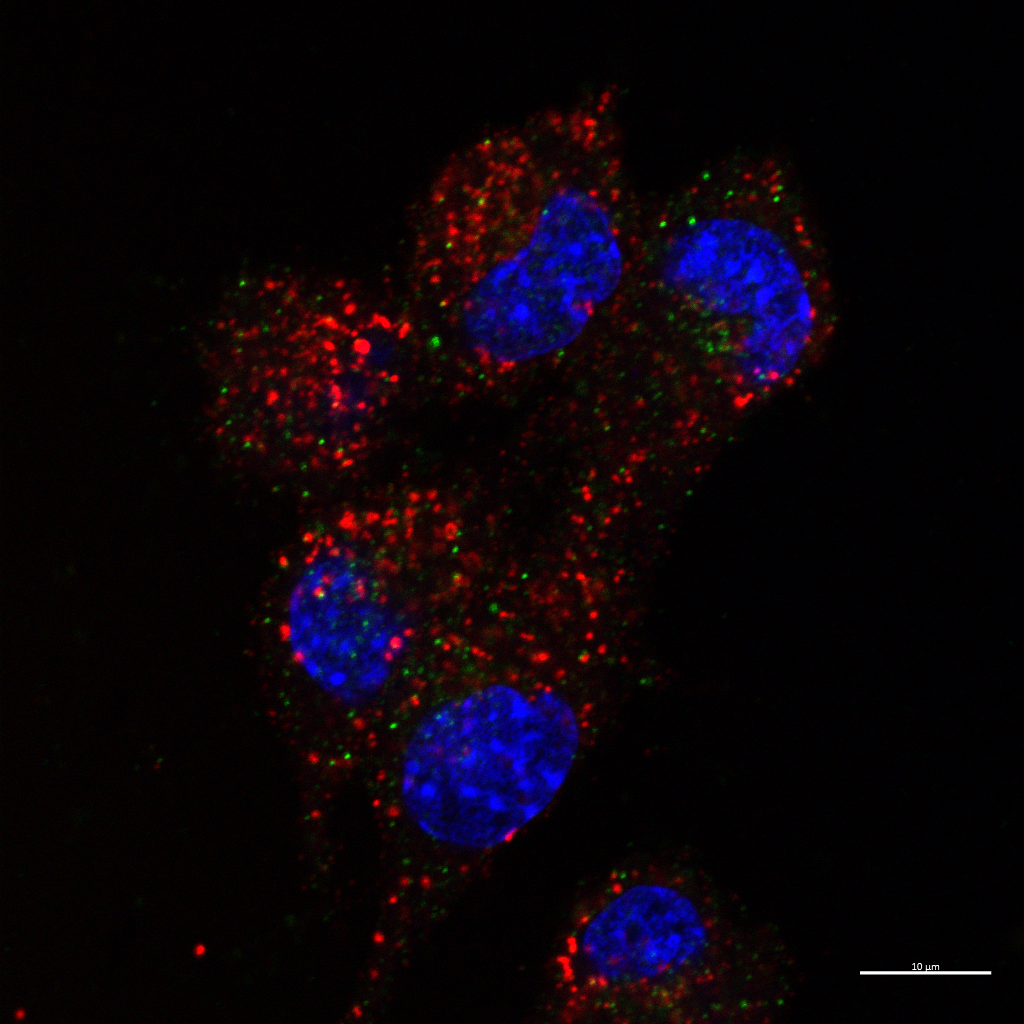

Supplement: Supplementary file 13 — Figure EV4 Source Data [file 44318_2026_817_MOESM13_ESM.zip › EV4A/EV4A-Basal.tif]

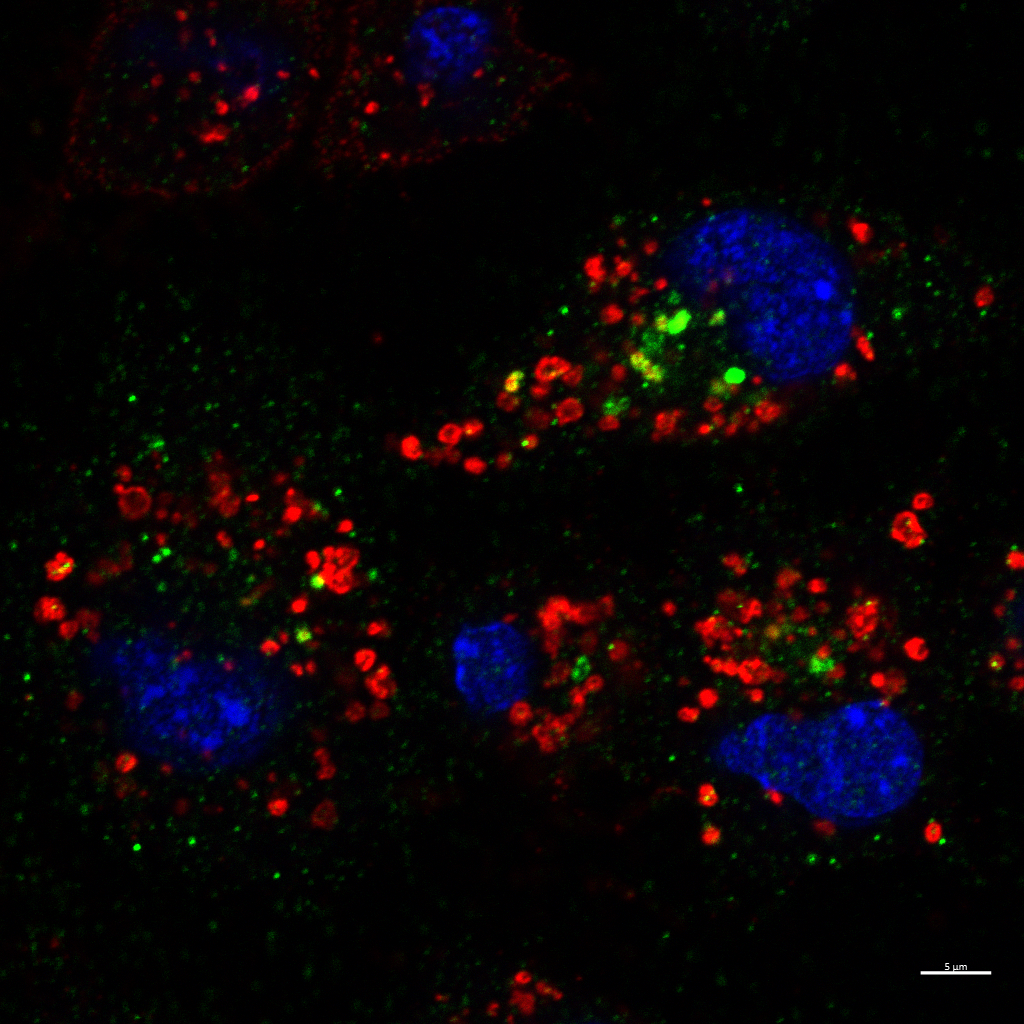

Supplement: Supplementary file 13 — Figure EV4 Source Data [file 44318_2026_817_MOESM13_ESM.zip › EV4A/EV4A-LLOMe 30 min.tif]

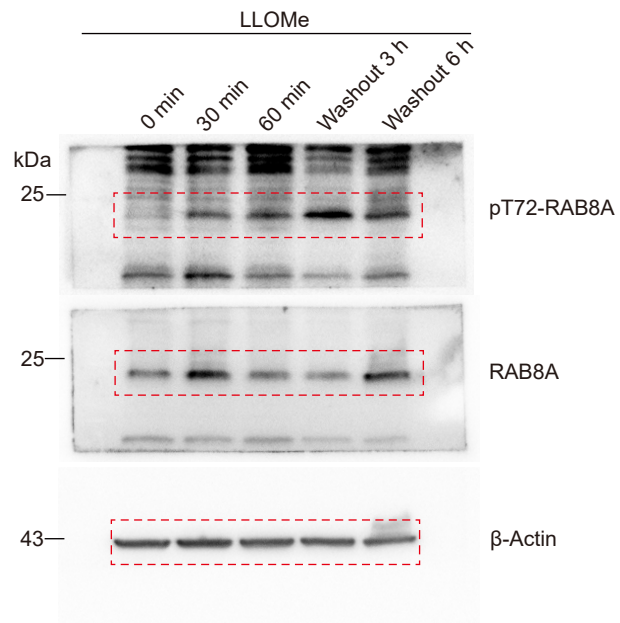

Supplement: Supplementary file 13 — Figure EV4 Source Data [file 44318_2026_817_MOESM13_ESM.zip › EV4B/EV4B.pdf]

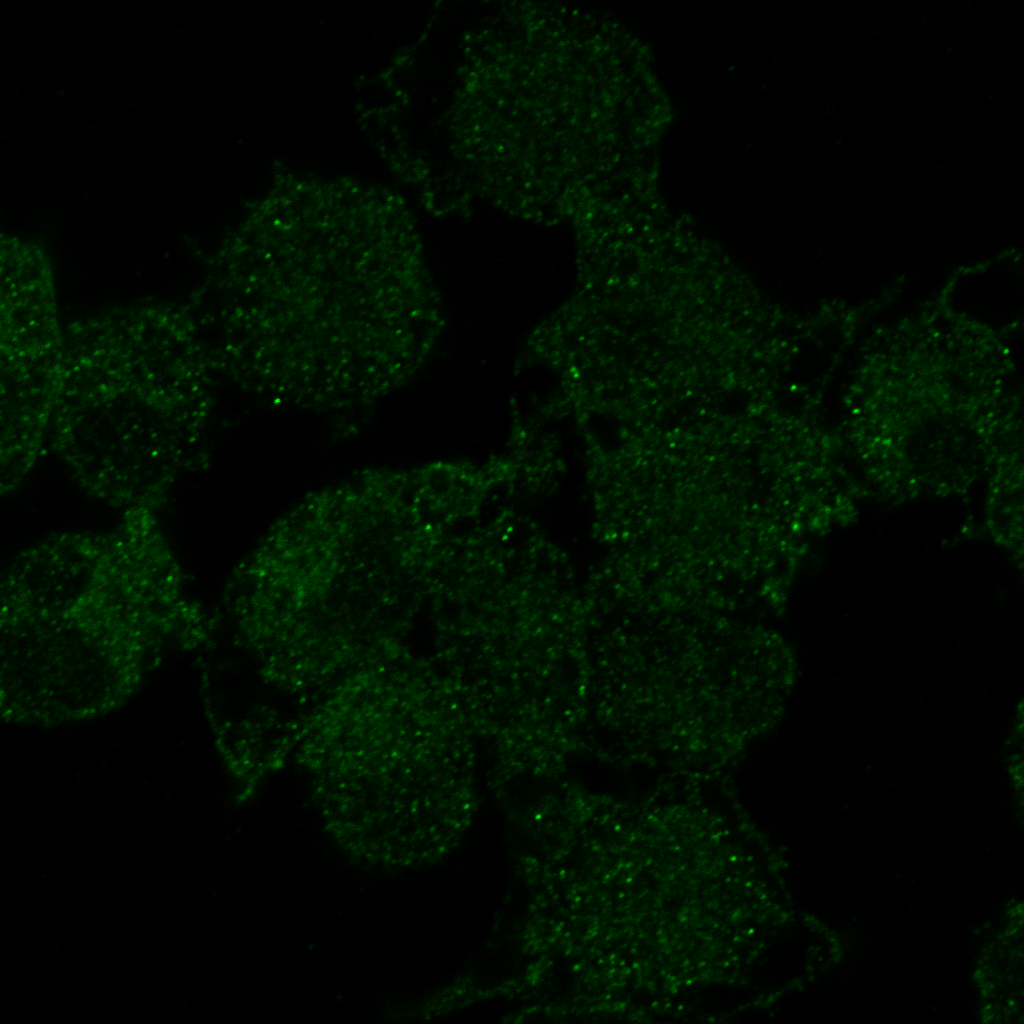

Supplement: Supplementary file 13 — Figure EV4 Source Data [file 44318_2026_817_MOESM13_ESM.zip › EV4C/EV4C-1-LLOMe 2 min_CHMP2B.tif]

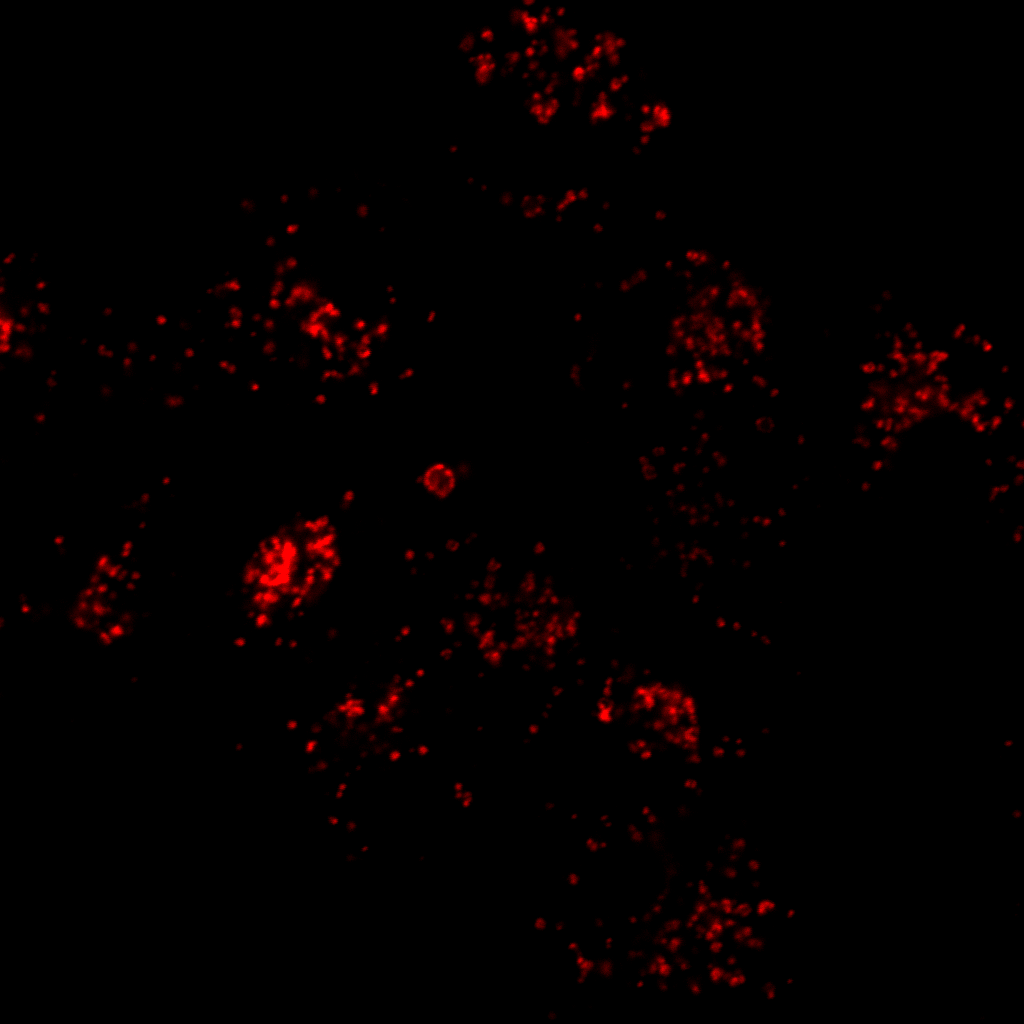

Supplement: Supplementary file 13 — Figure EV4 Source Data [file 44318_2026_817_MOESM13_ESM.zip › EV4C/EV4C-1-LLOMe 2 min_LAMP1.tif]

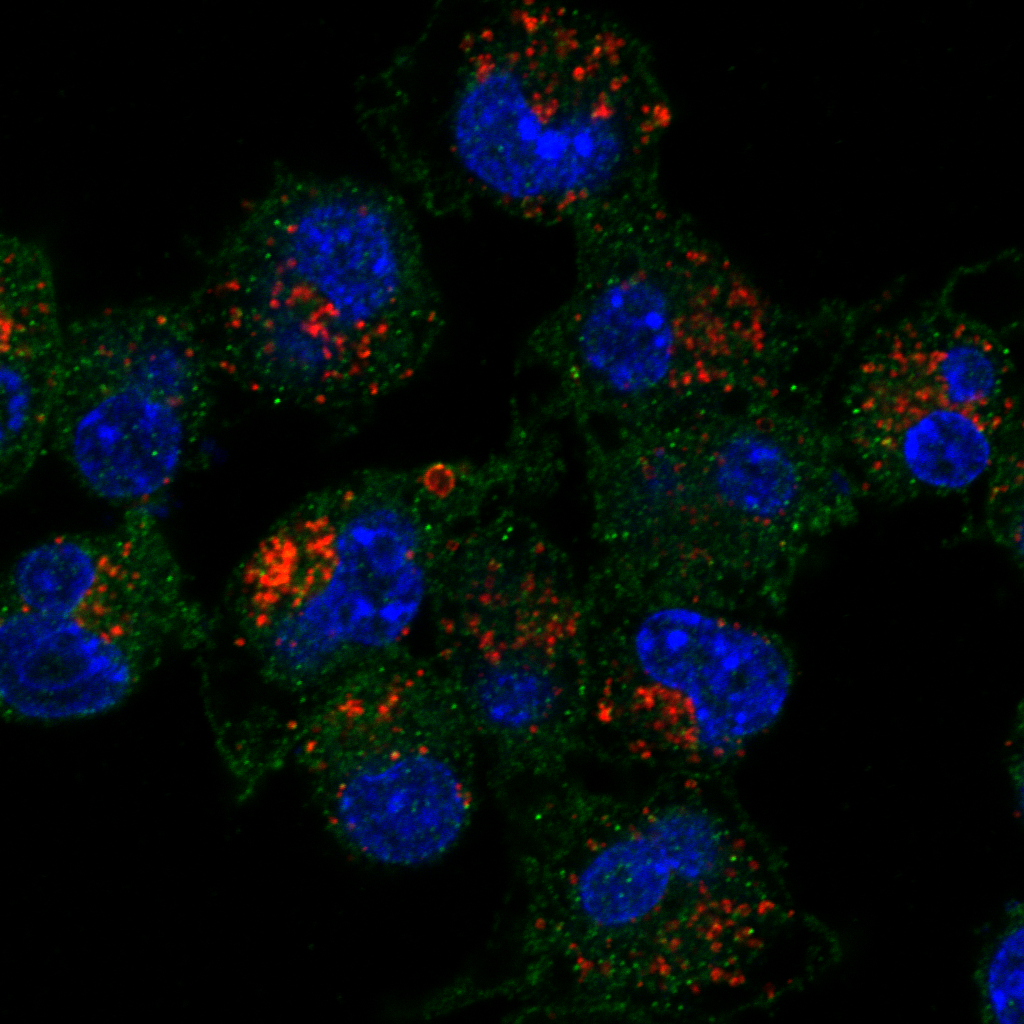

Supplement: Supplementary file 13 — Figure EV4 Source Data [file 44318_2026_817_MOESM13_ESM.zip › EV4C/EV4C-1-LLOMe 2 min_Merge.tif]

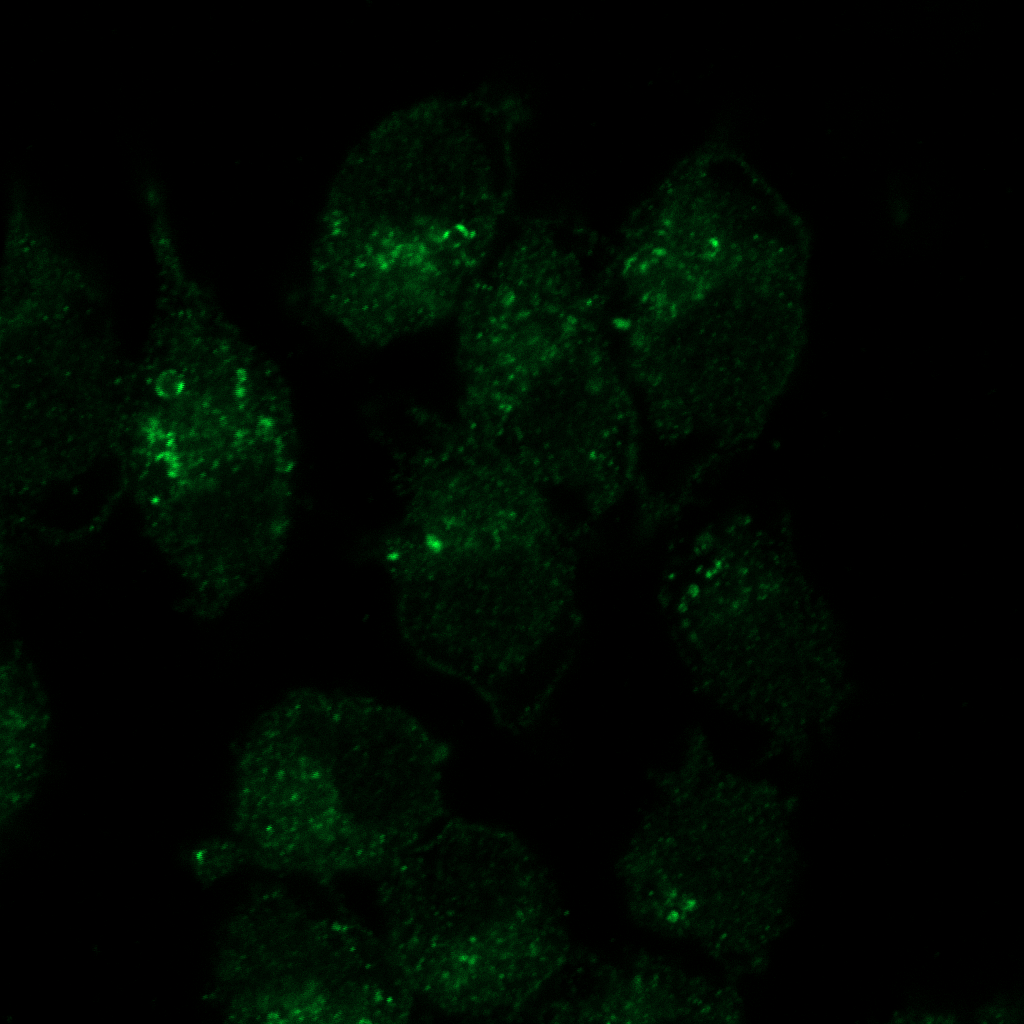

Supplement: Supplementary file 13 — Figure EV4 Source Data [file 44318_2026_817_MOESM13_ESM.zip › EV4C/EV4C-2-LLOMe 5 min_CHMP2B.tif]

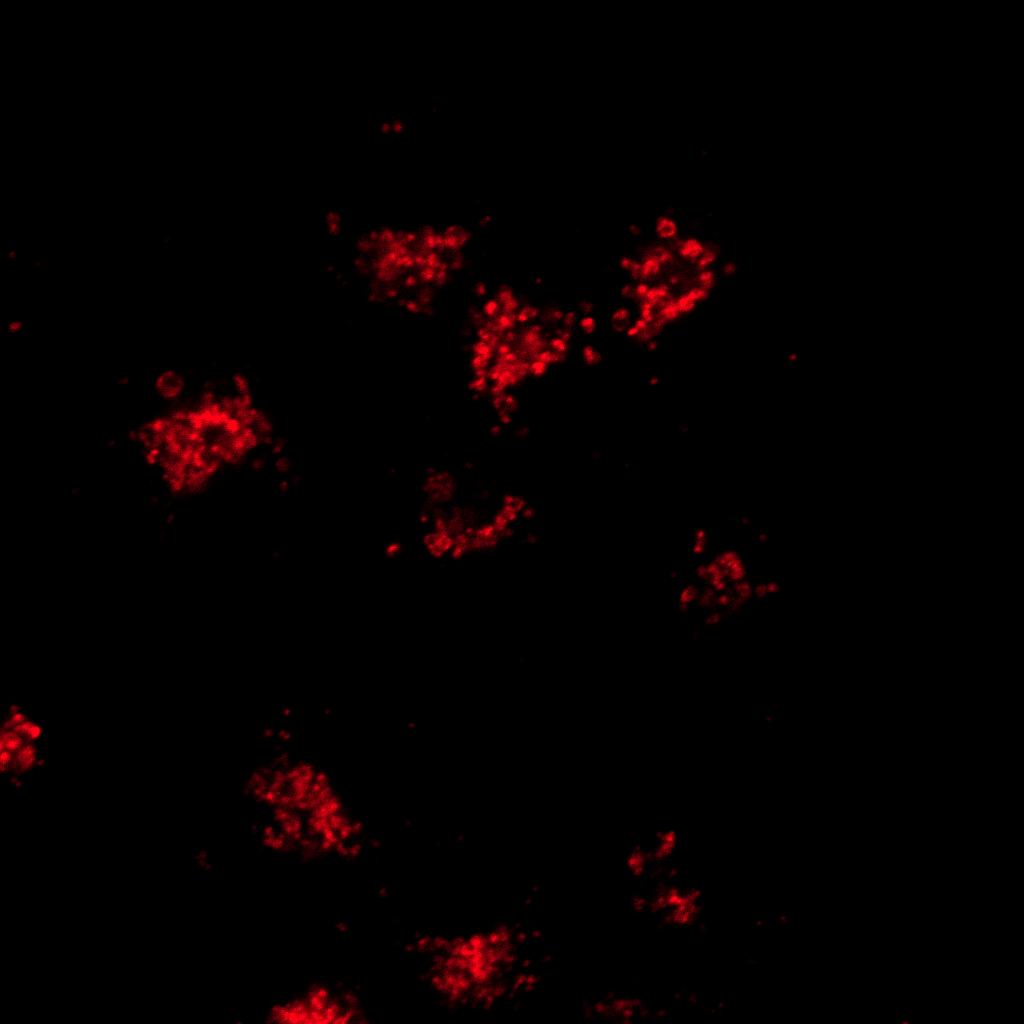

Supplement: Supplementary file 13 — Figure EV4 Source Data [file 44318_2026_817_MOESM13_ESM.zip › EV4C/EV4C-2-LLOMe 5 min_LAMP1.tif]

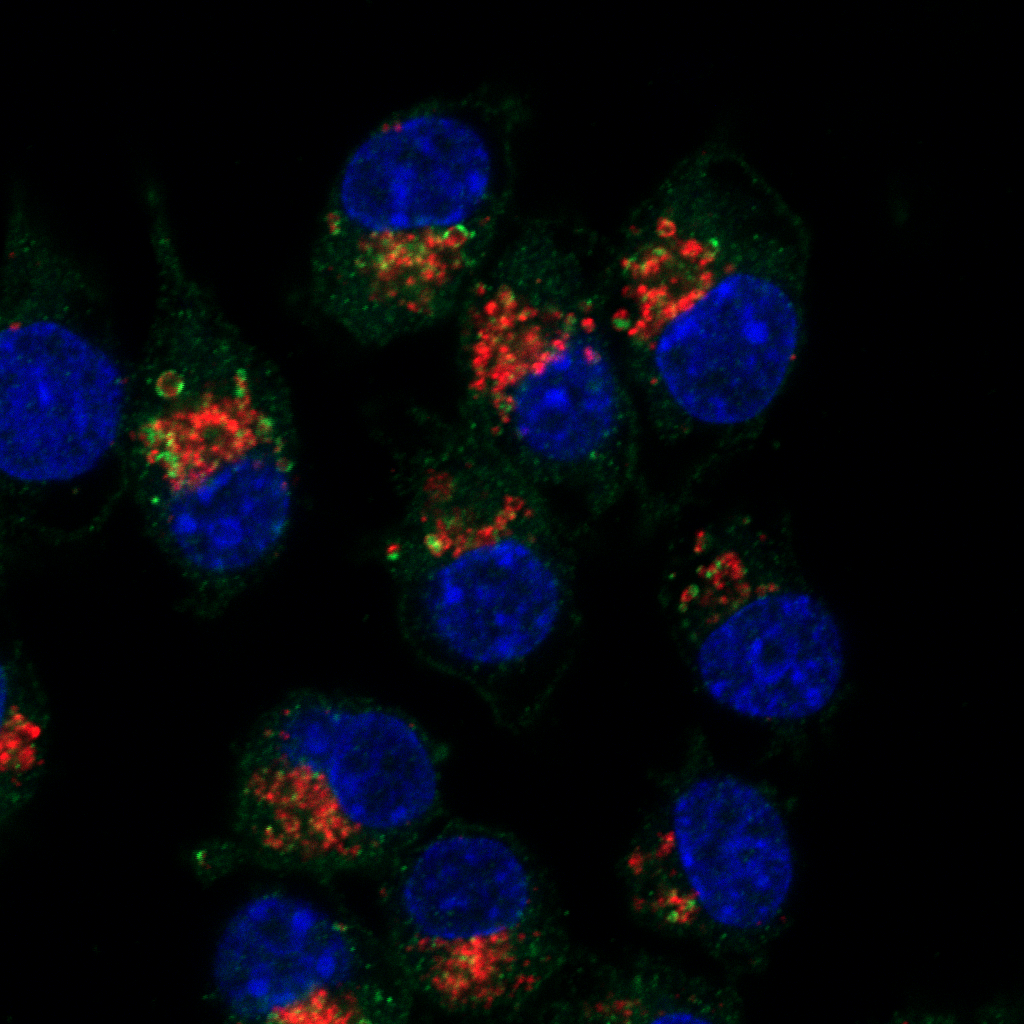

Supplement: Supplementary file 13 — Figure EV4 Source Data [file 44318_2026_817_MOESM13_ESM.zip › EV4C/EV4C-2-LLOMe 5 min_Merge.tif]

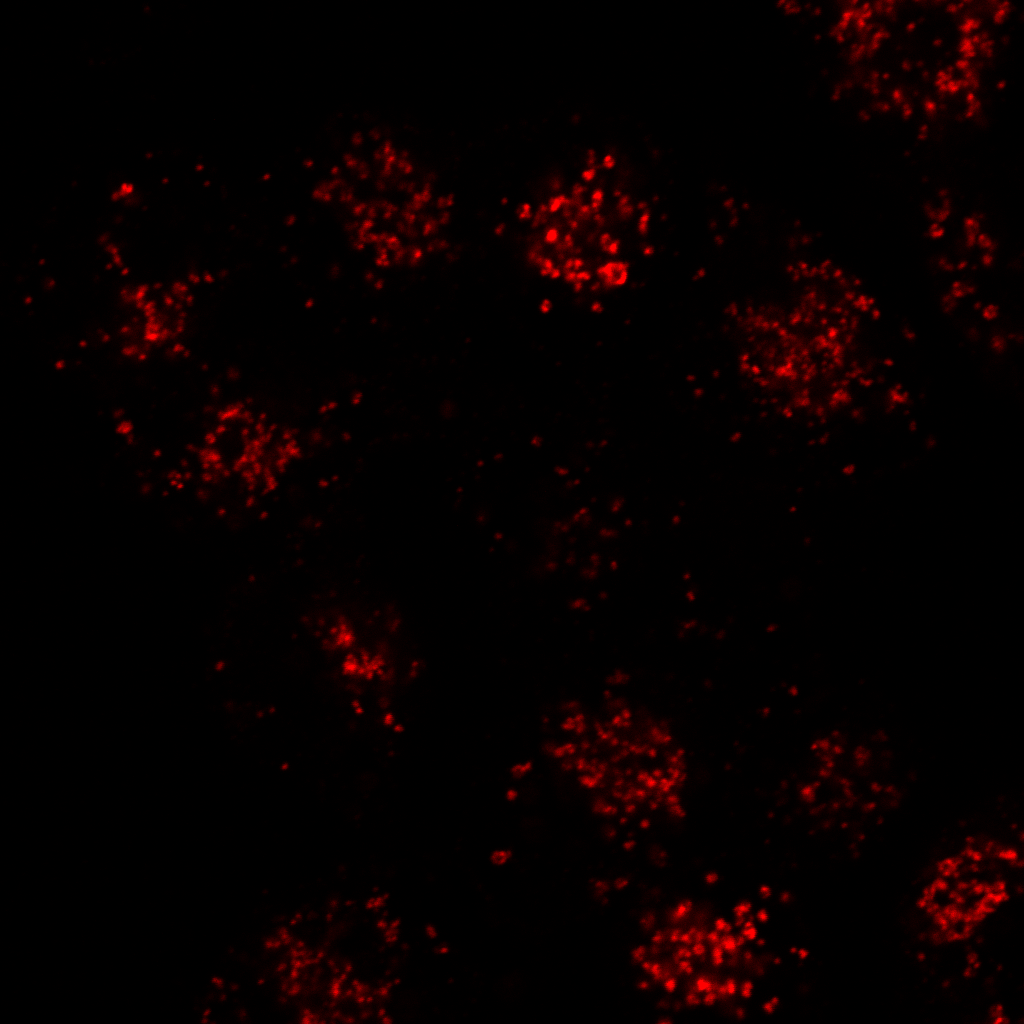

Supplement: Supplementary file 13 — Figure EV4 Source Data [file 44318_2026_817_MOESM13_ESM.zip › EV4D/EV4D-1-LLOMe 2 min_LAMP1.tif]

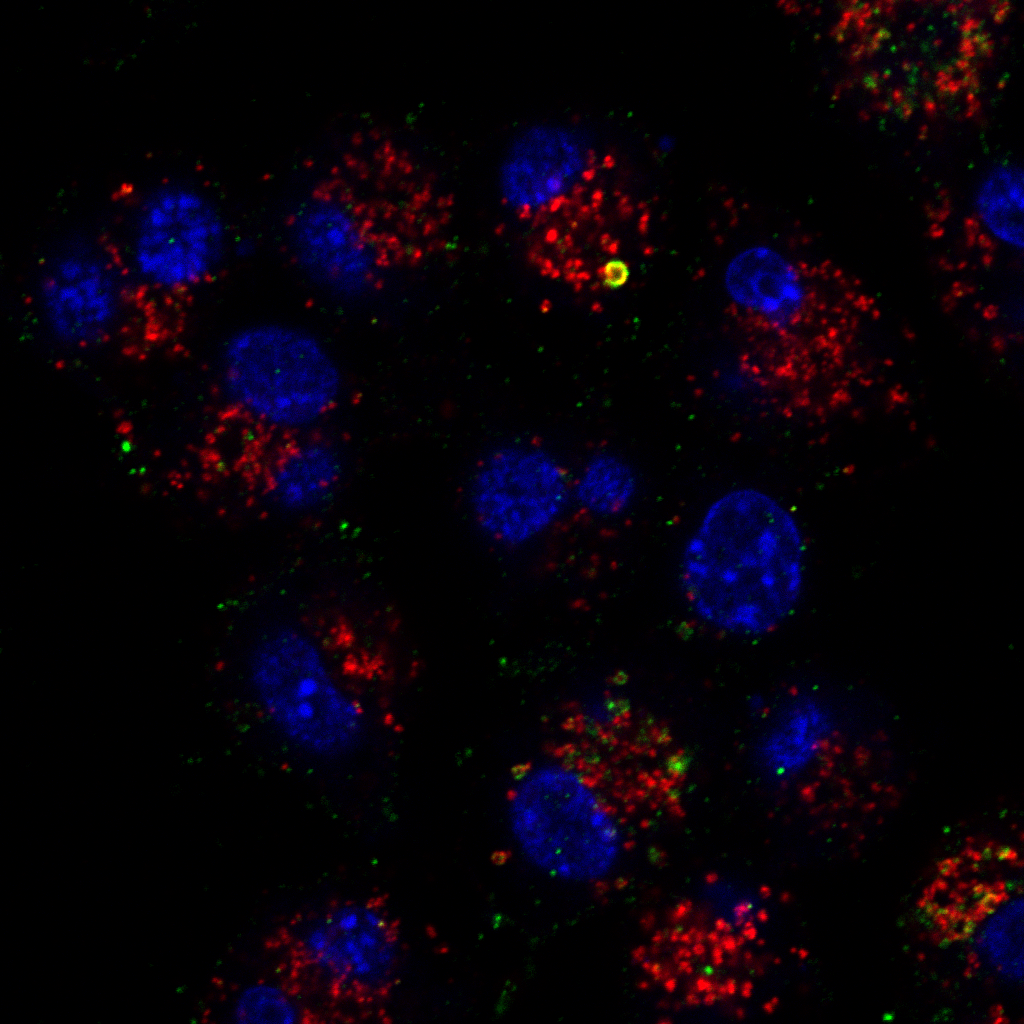

Supplement: Supplementary file 13 — Figure EV4 Source Data [file 44318_2026_817_MOESM13_ESM.zip › EV4D/EV4D-1-LLOMe 2 min_Merge.tif]

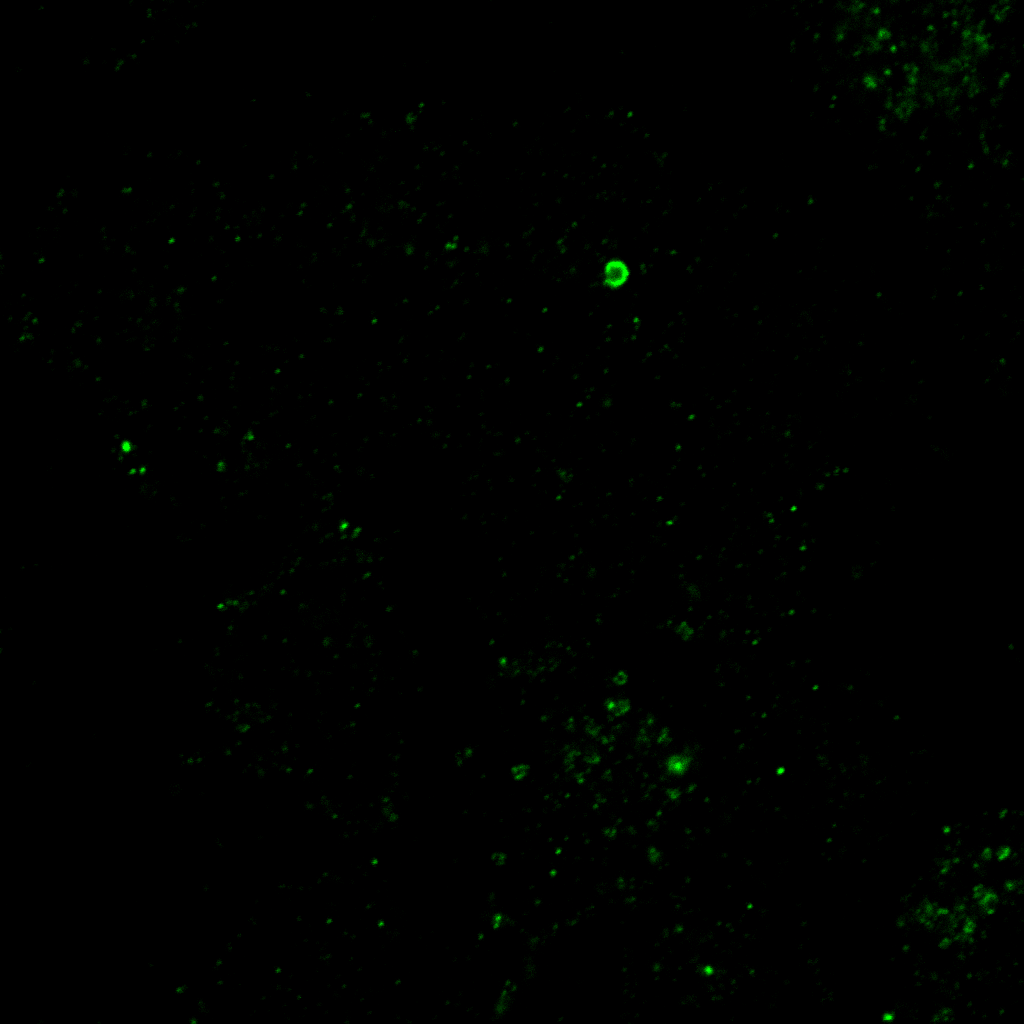

Supplement: Supplementary file 13 — Figure EV4 Source Data [file 44318_2026_817_MOESM13_ESM.zip › EV4D/EV4D-1-LLOMe 2 min_pT72-RAB8A.tif]

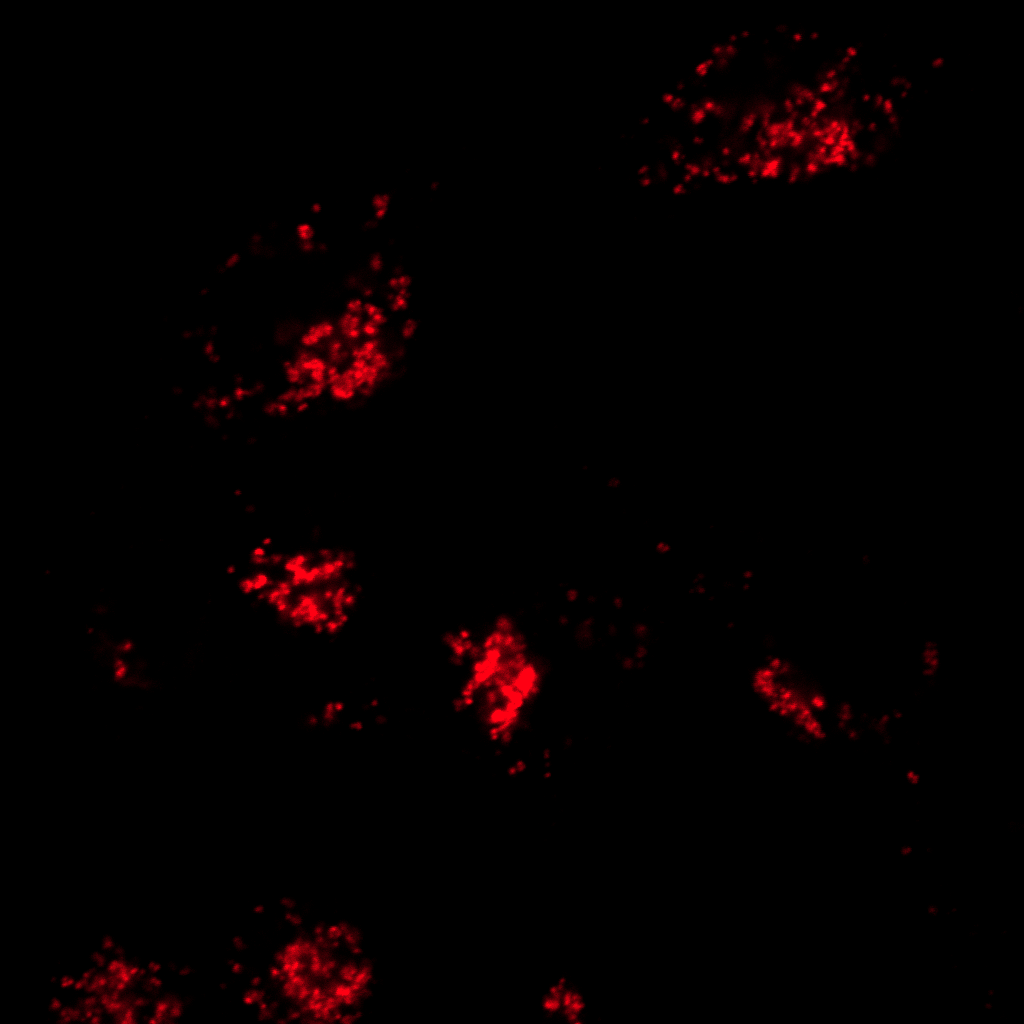

Supplement: Supplementary file 13 — Figure EV4 Source Data [file 44318_2026_817_MOESM13_ESM.zip › EV4D/EV4D-2-LLOMe 5 min_LAMP1.tif]

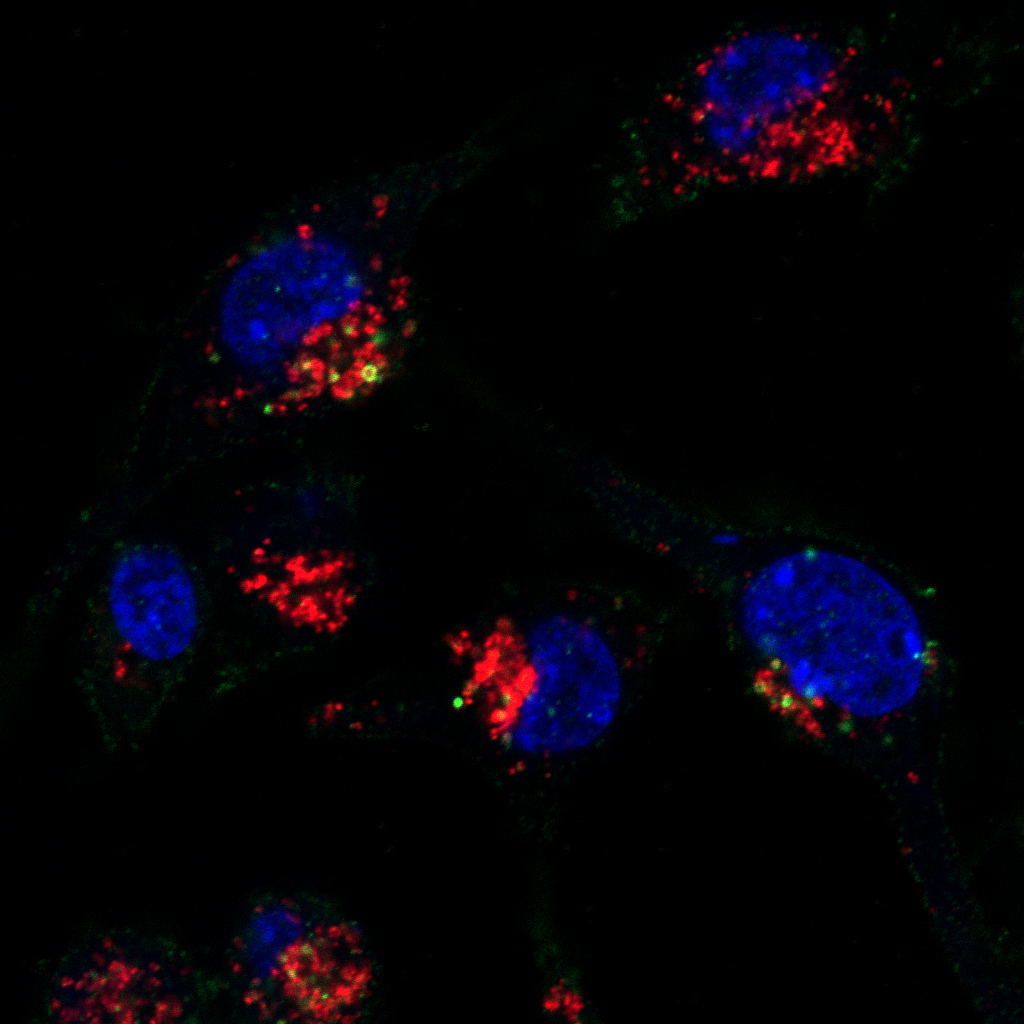

Supplement: Supplementary file 13 — Figure EV4 Source Data [file 44318_2026_817_MOESM13_ESM.zip › EV4D/EV4D-2-LLOMe 5 min_Merge.tif]

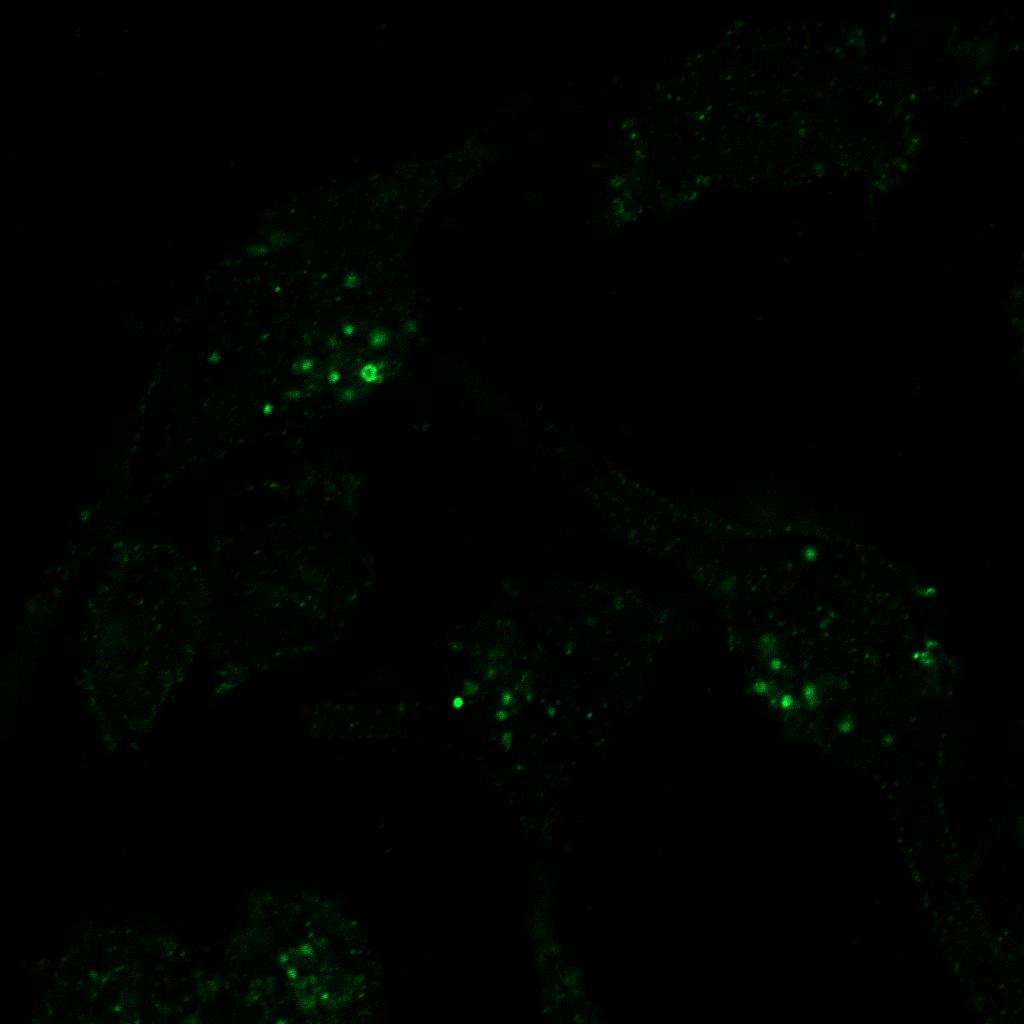

Supplement: Supplementary file 13 — Figure EV4 Source Data [file 44318_2026_817_MOESM13_ESM.zip › EV4D/EV4D-2-LLOMe 5 min_pT72-RAB8A.tif]

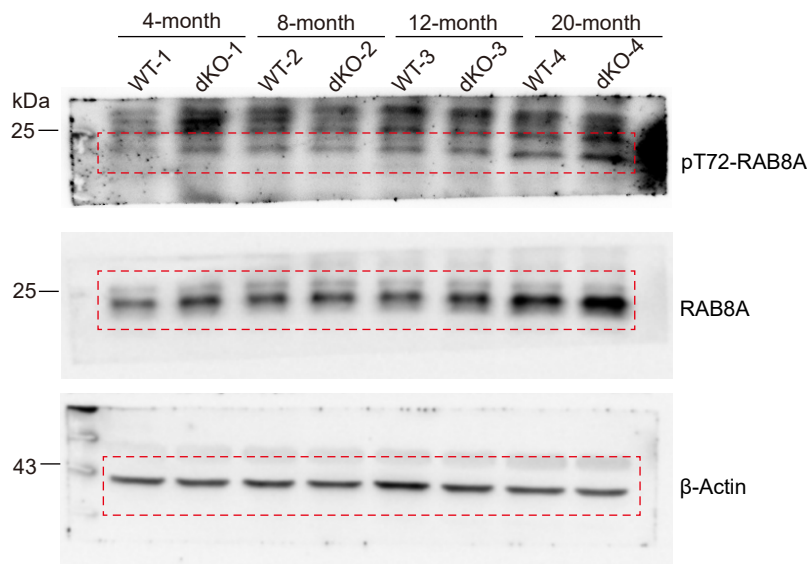

Supplement: Supplementary file 13 — Figure EV4 Source Data [file 44318_2026_817_MOESM13_ESM.zip › EV4E/EV4E.pdf]

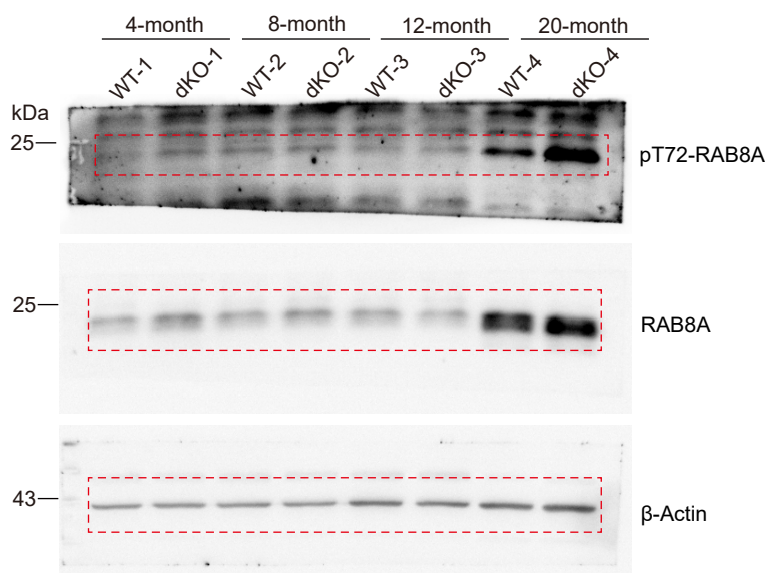

Supplement: Supplementary file 13 — Figure EV4 Source Data [file 44318_2026_817_MOESM13_ESM.zip › EV4I/EV4I.pdf]

|            |   |   |
|------------|---|---|
| HA         | + | - |
| GFP-RAB8A  | + | + |
| HA-MICALL2 | - | + |

IP:GFP

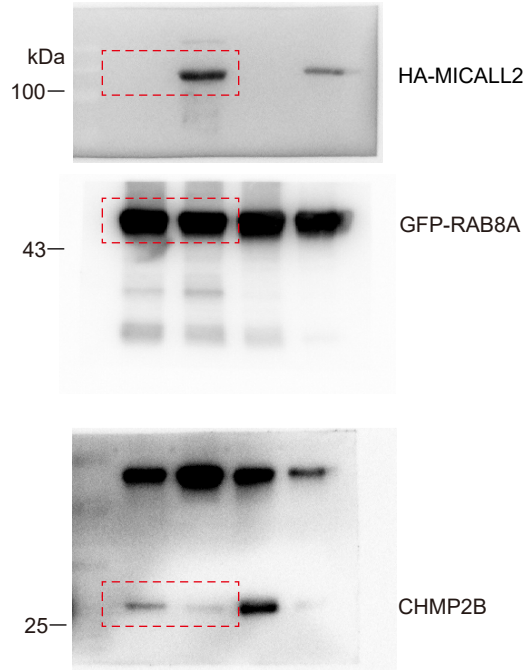

Input

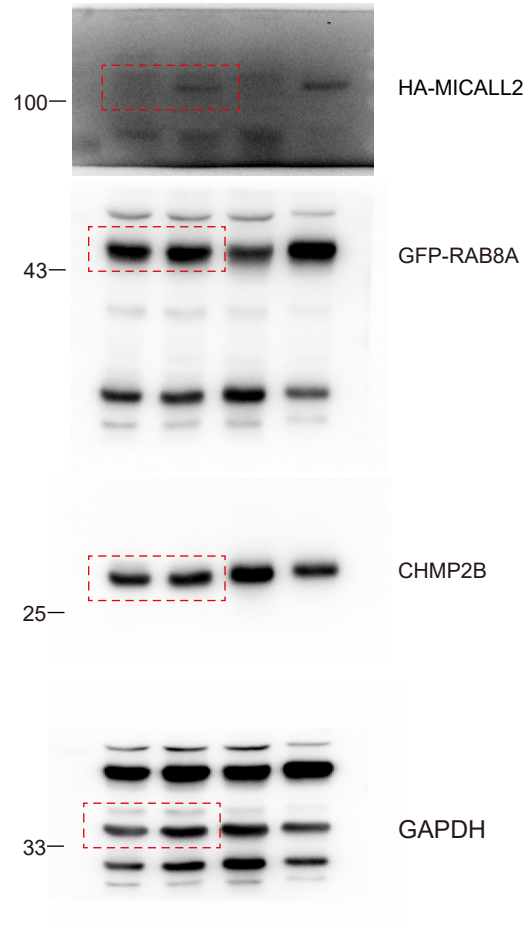

Supplement: Supplementary file 13 — Figure EV4 Source Data [file 44318_2026_817_MOESM13_ESM.zip › EV4N/EV4N.pdf]

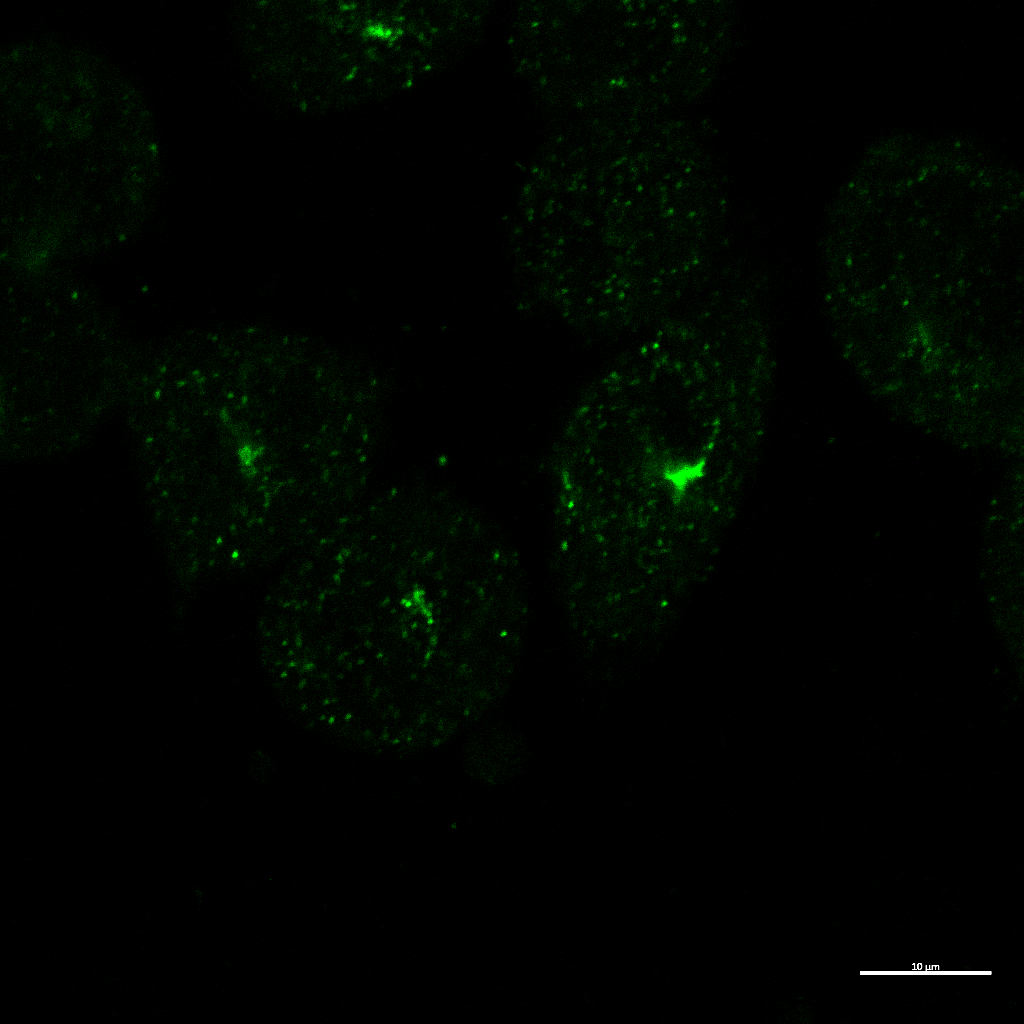

Supplement: Supplementary file 14 — Figure EV5 Source Data [file 44318_2026_817_MOESM14_ESM.zip › EV5A/EV4A-dKO_Ac-Tubulin.tif]

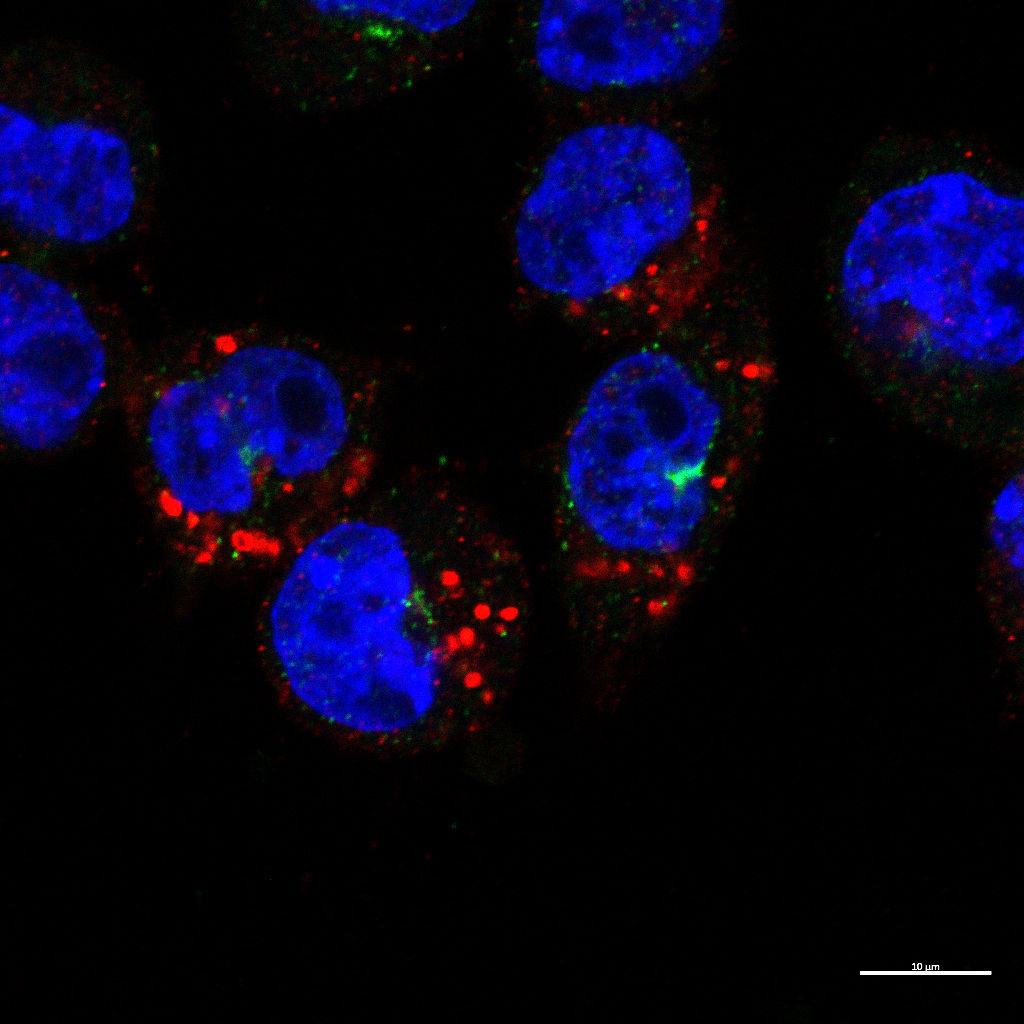

Supplement: Supplementary file 14 — Figure EV5 Source Data [file 44318_2026_817_MOESM14_ESM.zip › EV5A/EV4A-dKO_Merge.tif]

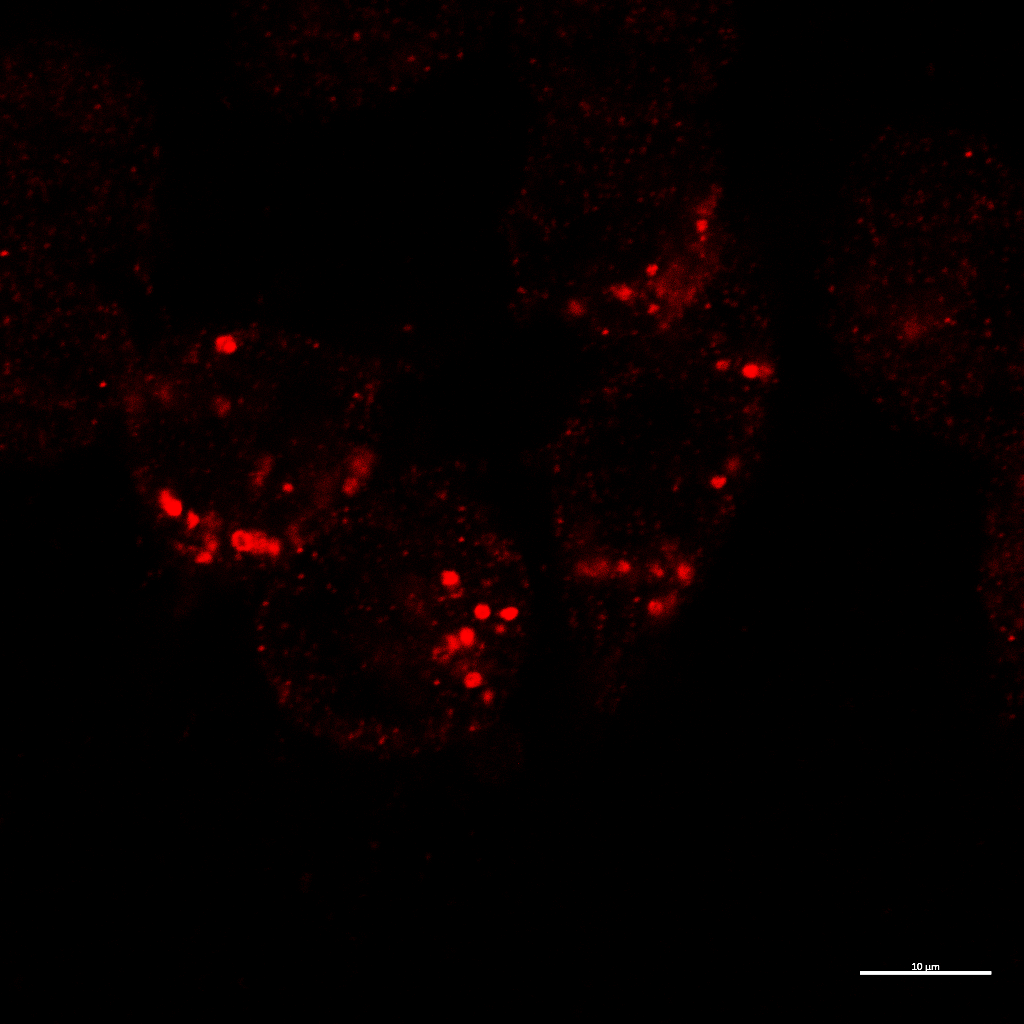

Supplement: Supplementary file 14 — Figure EV5 Source Data [file 44318_2026_817_MOESM14_ESM.zip › EV5A/EV4A-dKO_pT72-RAB8A.tif]

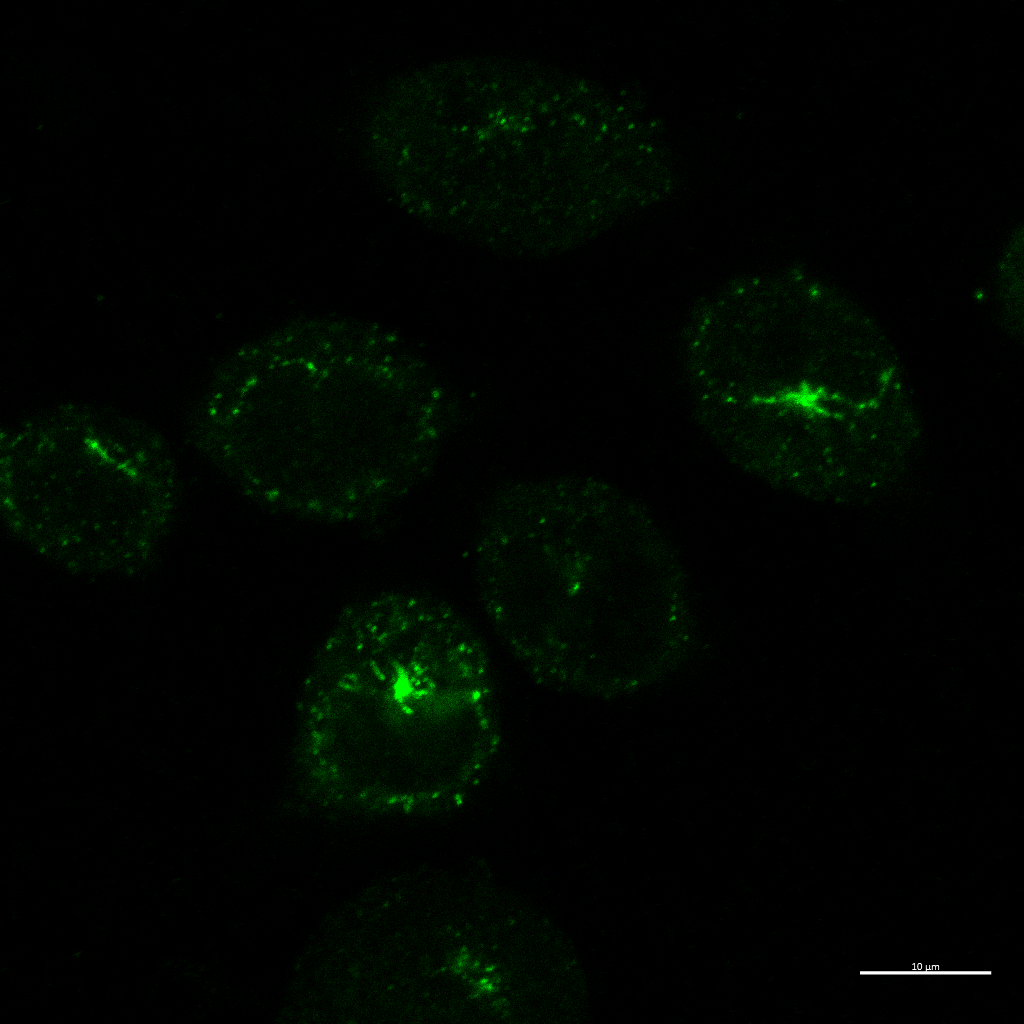

Supplement: Supplementary file 14 — Figure EV5 Source Data [file 44318_2026_817_MOESM14_ESM.zip › EV5A/EV4A-WT_Ac-Tubulin.tif]

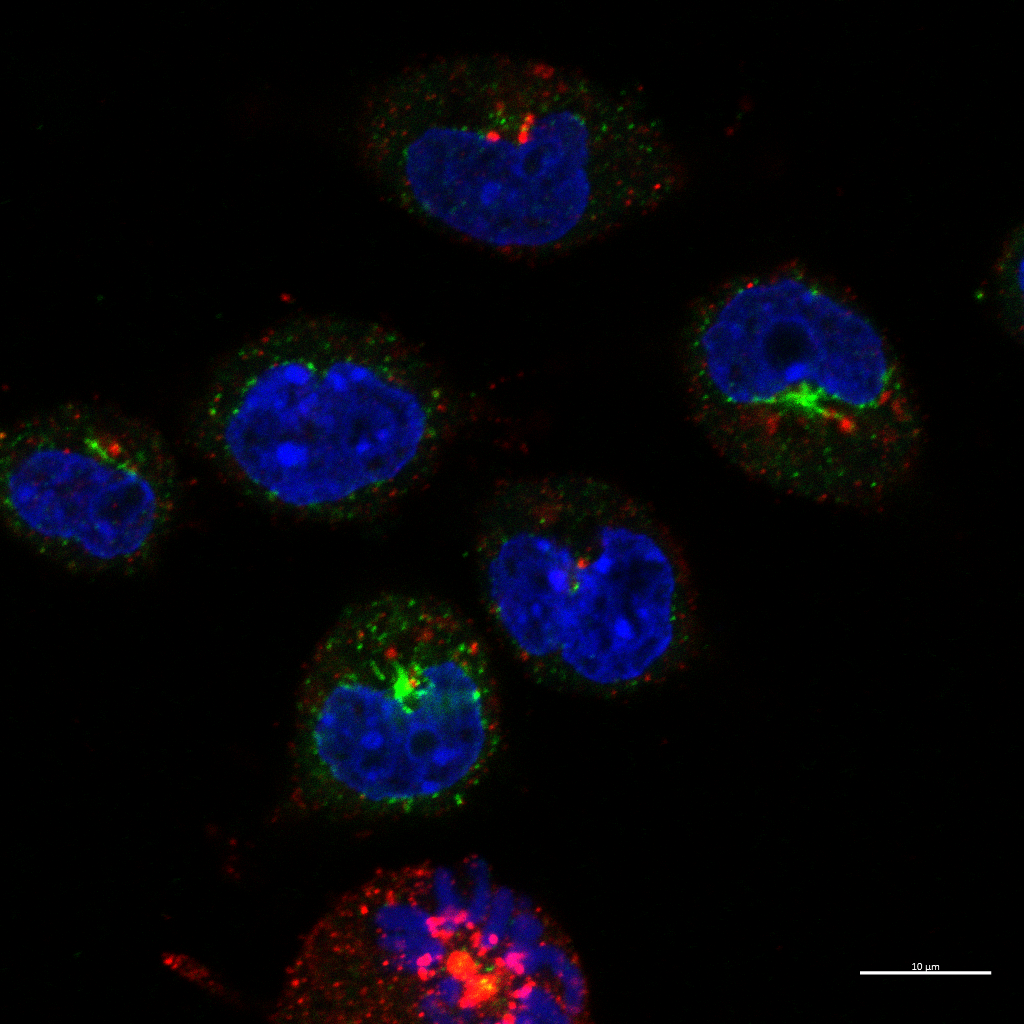

Supplement: Supplementary file 14 — Figure EV5 Source Data [file 44318_2026_817_MOESM14_ESM.zip › EV5A/EV4A-WT_Merge.tif]

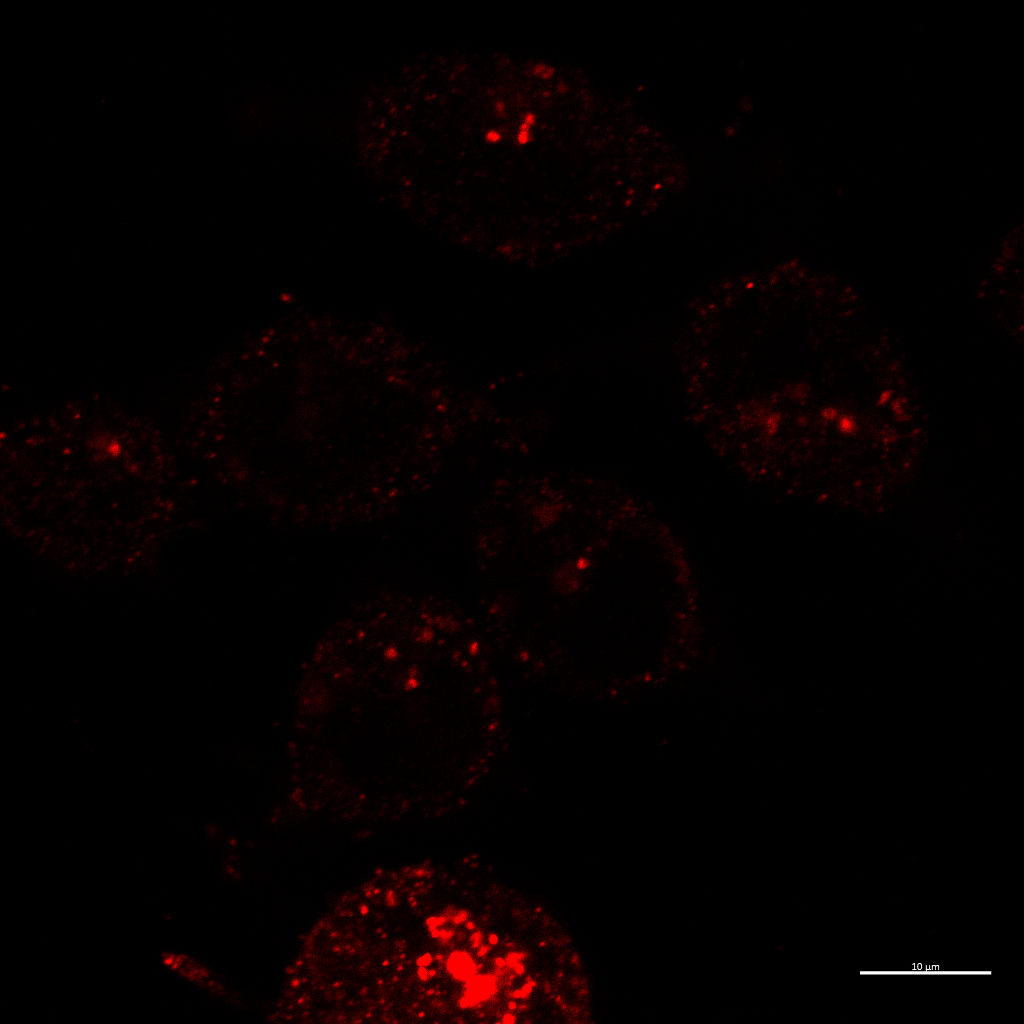

Supplement: Supplementary file 14 — Figure EV5 Source Data [file 44318_2026_817_MOESM14_ESM.zip › EV5A/EV4A-WT_pT72-RAB8A.tif]

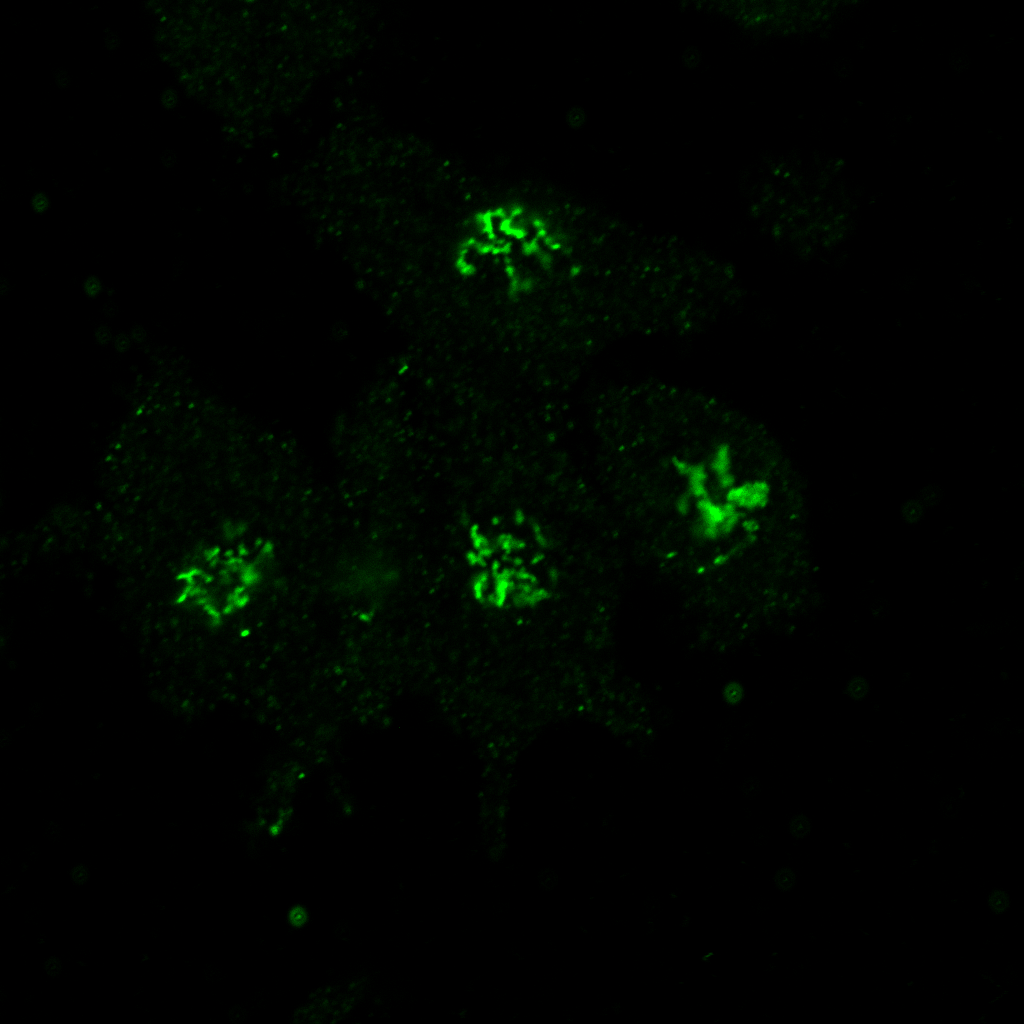

Supplement: Supplementary file 14 — Figure EV5 Source Data [file 44318_2026_817_MOESM14_ESM.zip › EV5B/EV4B-dKO_GM130.tif]

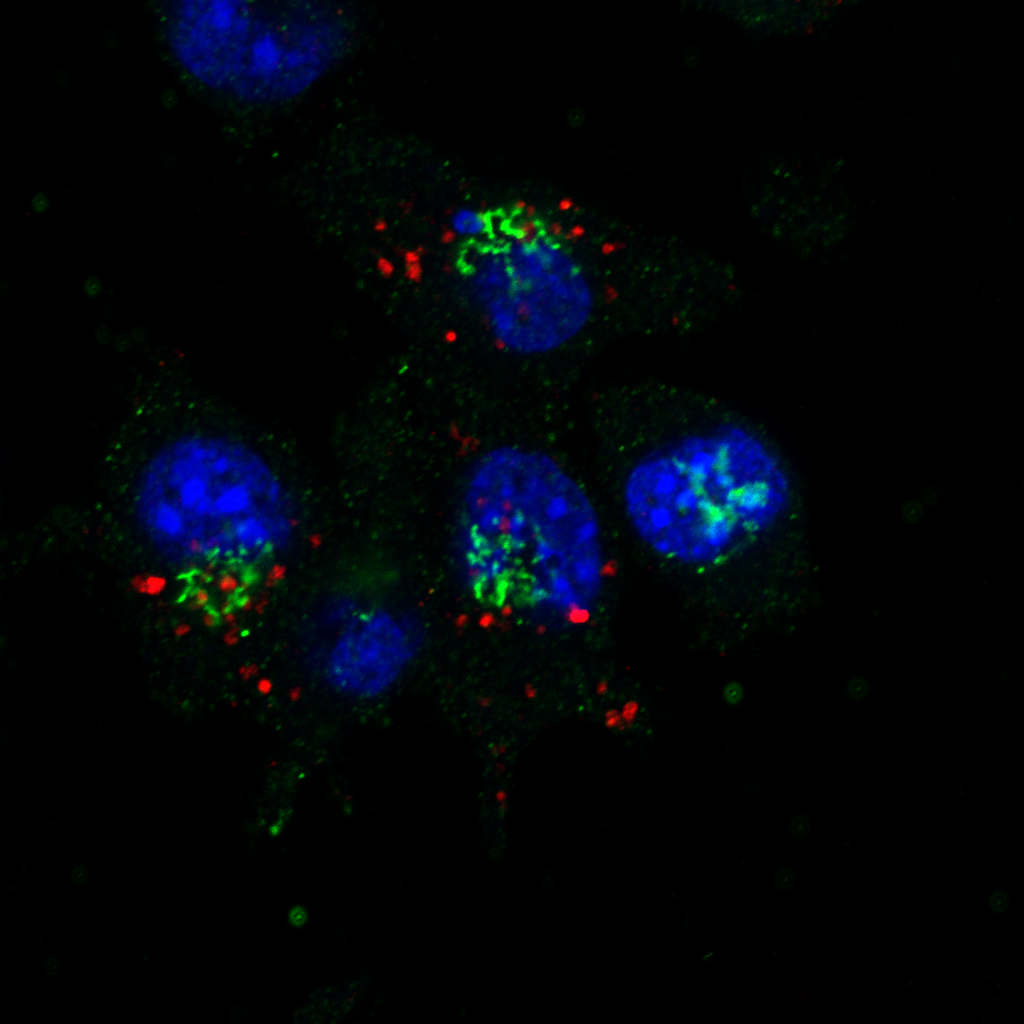

Supplement: Supplementary file 14 — Figure EV5 Source Data [file 44318_2026_817_MOESM14_ESM.zip › EV5B/EV4B-dKO_Merge.tif]

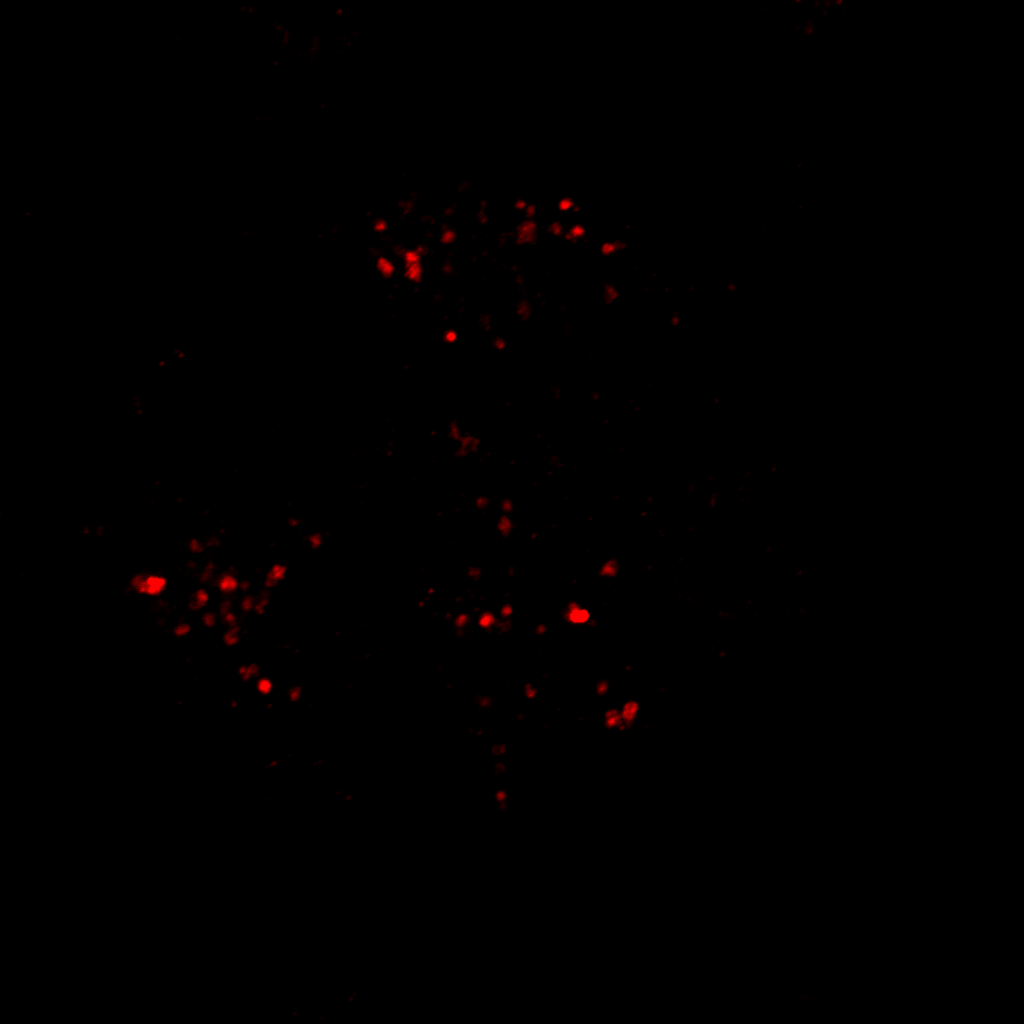

Supplement: Supplementary file 14 — Figure EV5 Source Data [file 44318_2026_817_MOESM14_ESM.zip › EV5B/EV4B-dKO_pT72-RAB8A.tif]

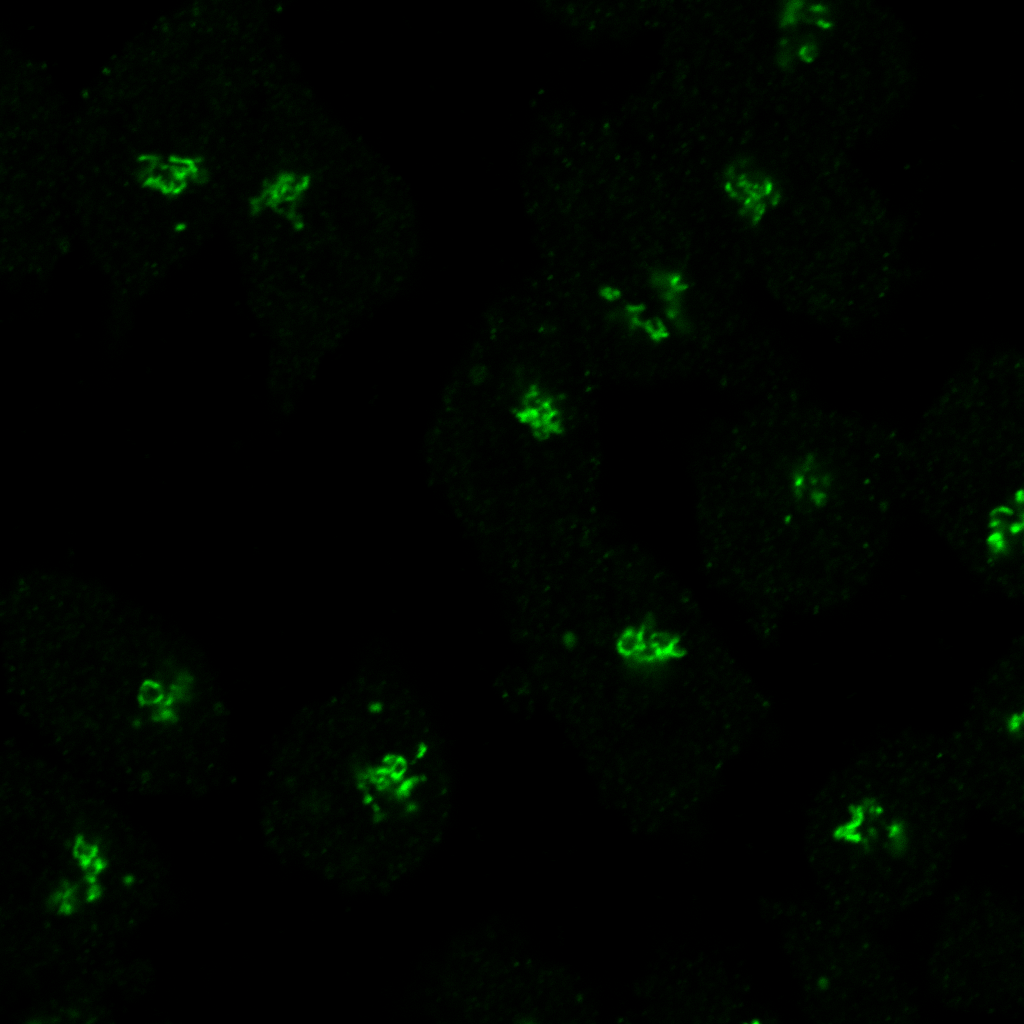

Supplement: Supplementary file 14 — Figure EV5 Source Data [file 44318_2026_817_MOESM14_ESM.zip › EV5B/EV4B-WT_GM130.tif]

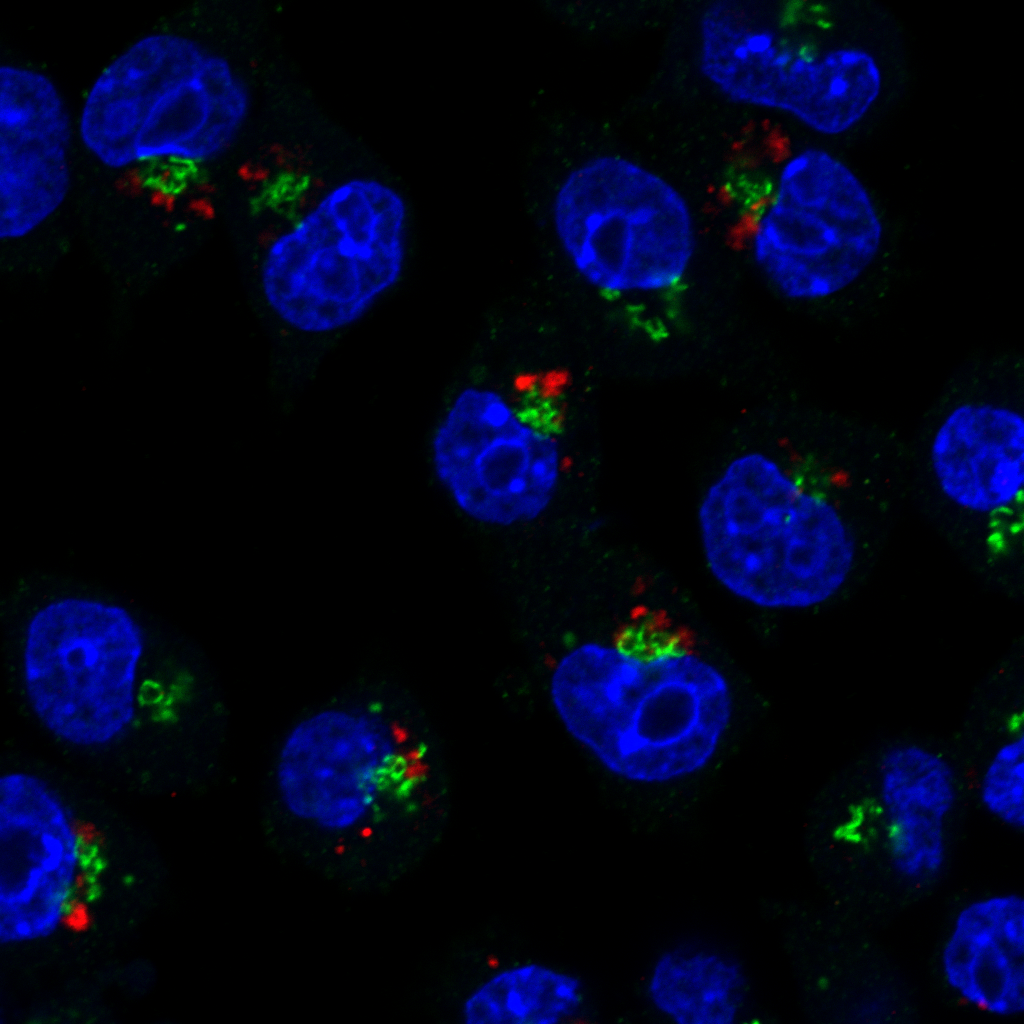

Supplement: Supplementary file 14 — Figure EV5 Source Data [file 44318_2026_817_MOESM14_ESM.zip › EV5B/EV4B-WT_Merge.tif]

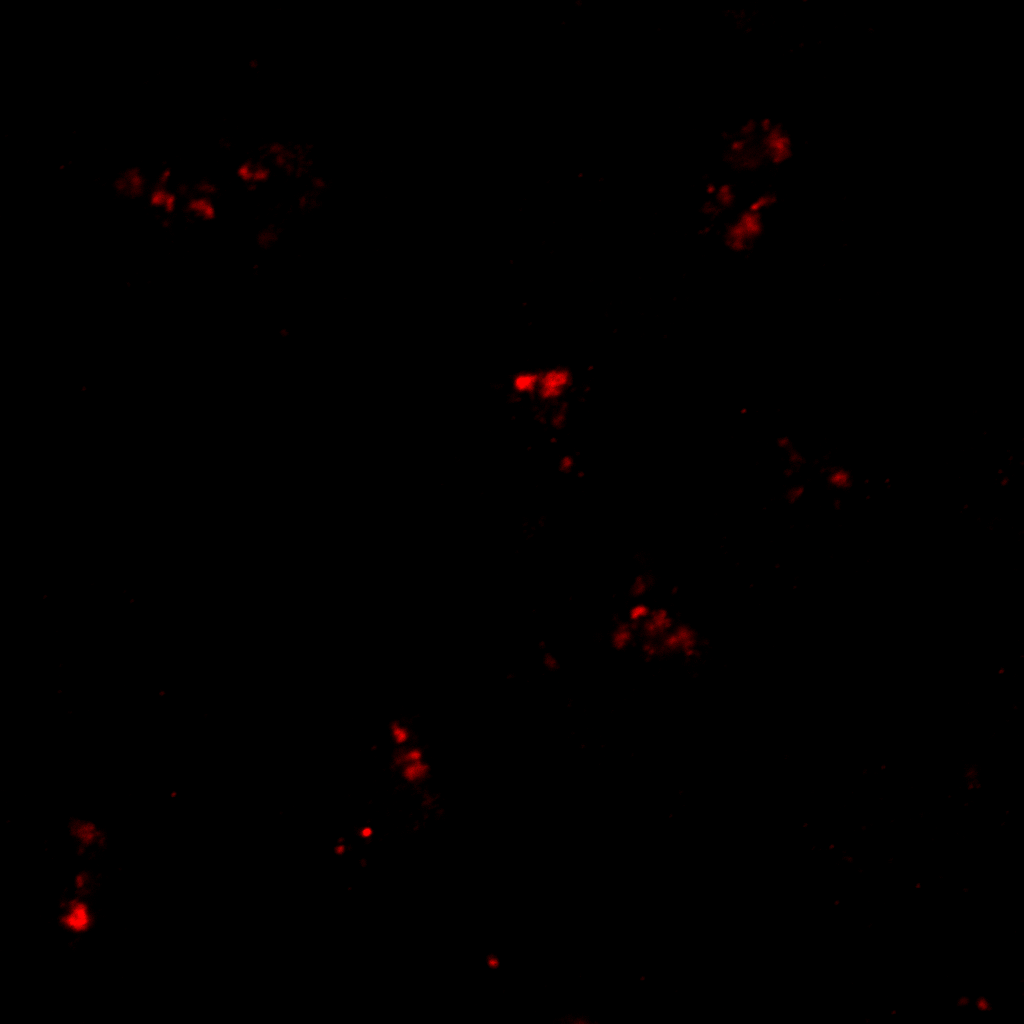

Supplement: Supplementary file 14 — Figure EV5 Source Data [file 44318_2026_817_MOESM14_ESM.zip › EV5B/EV4B-WT_pT72-RAB8A.tif]

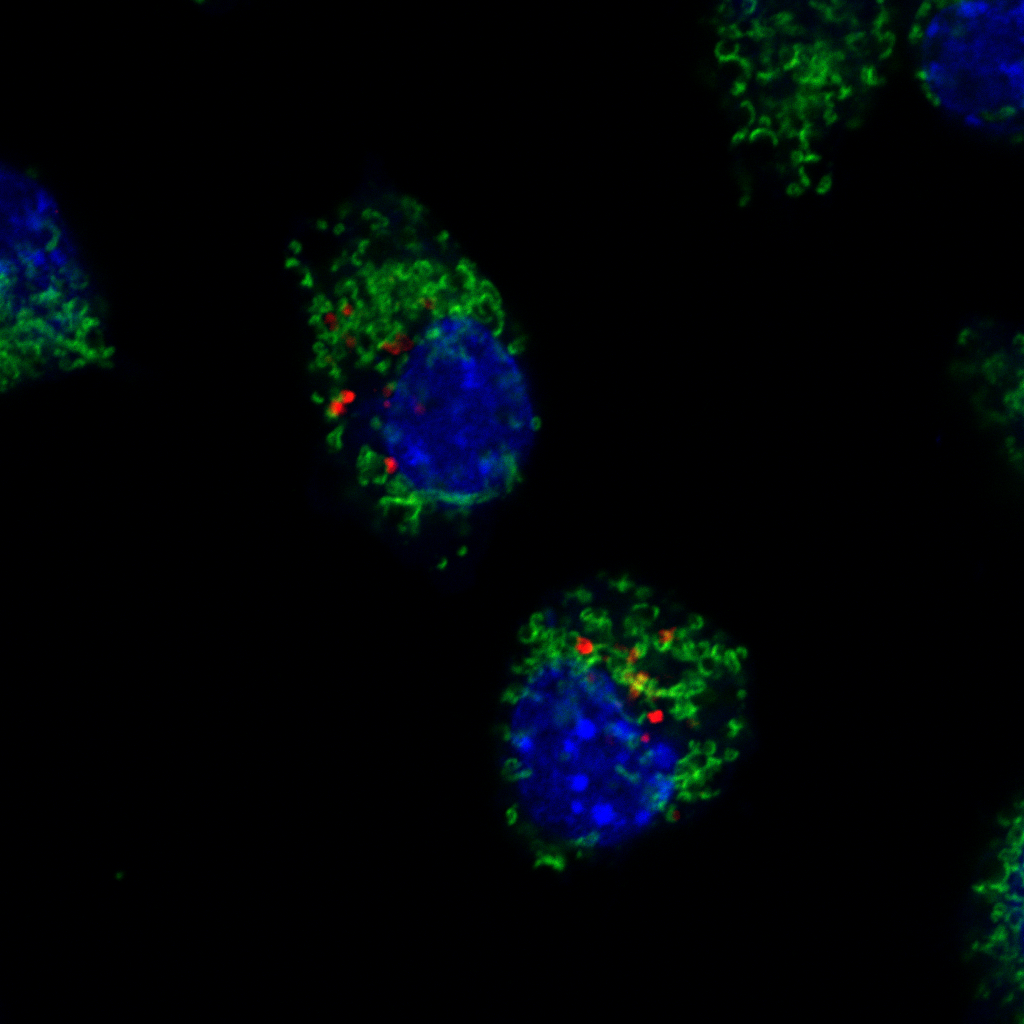

Supplement: Supplementary file 14 — Figure EV5 Source Data [file 44318_2026_817_MOESM14_ESM.zip › EV5C/EV4C-dKO_Merge.tif]

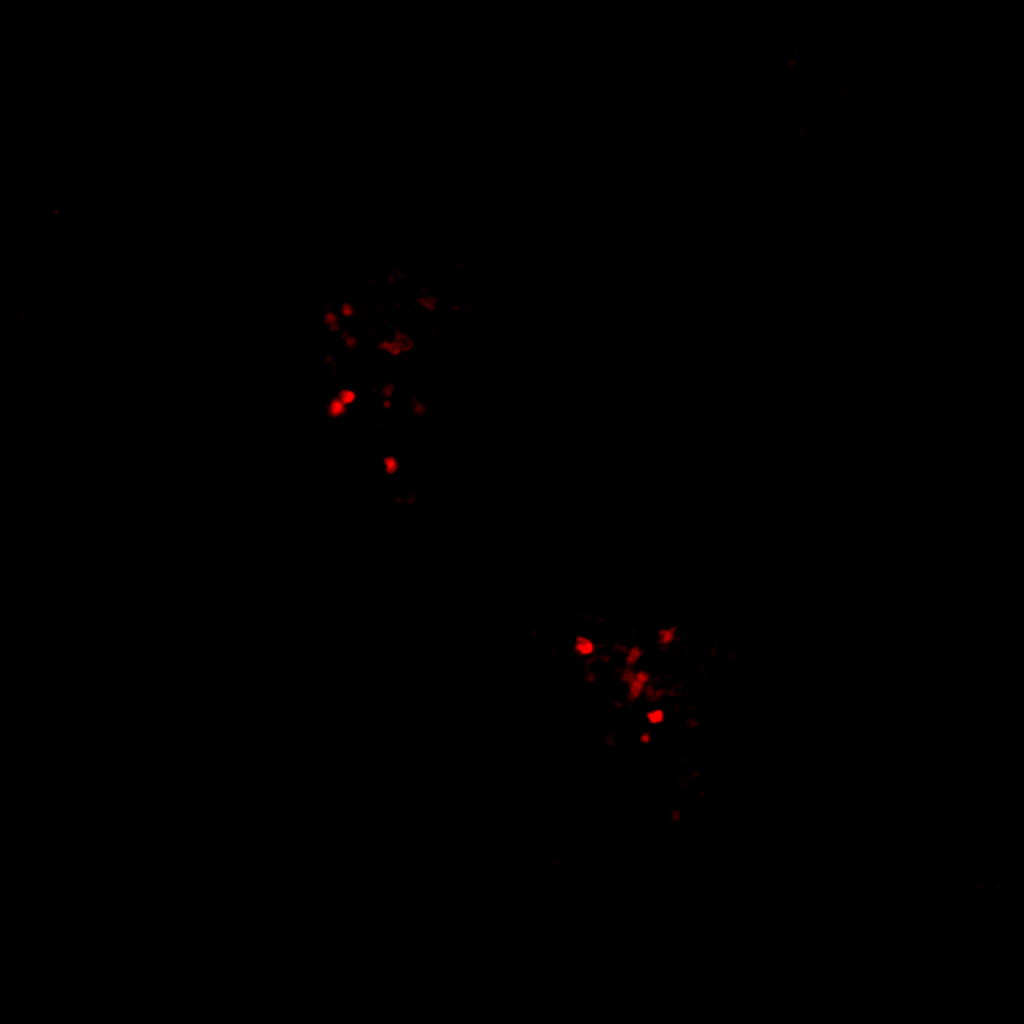

Supplement: Supplementary file 14 — Figure EV5 Source Data [file 44318_2026_817_MOESM14_ESM.zip › EV5C/EV4C-dKO_pT72-RAB8A.tif]

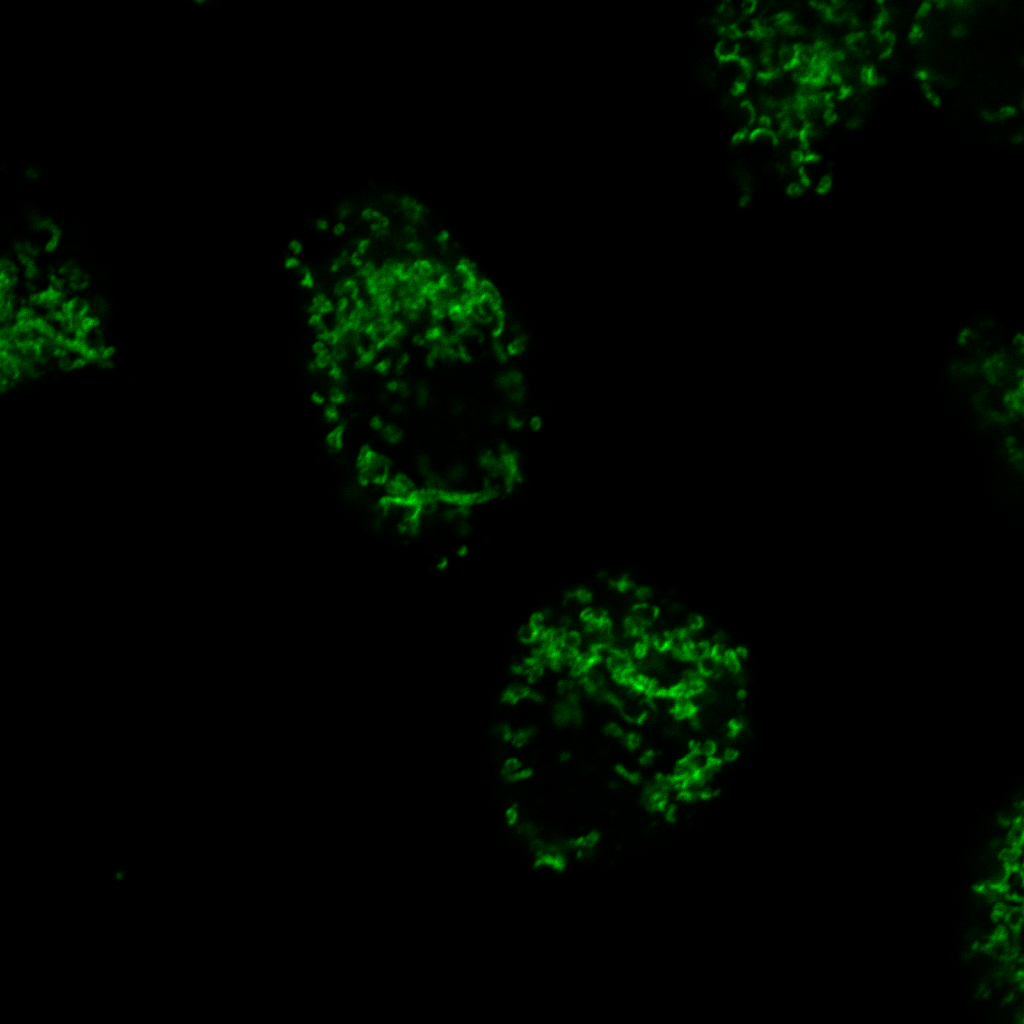

Supplement: Supplementary file 14 — Figure EV5 Source Data [file 44318_2026_817_MOESM14_ESM.zip › EV5C/EV4C-dKO_TOMM20.tif]

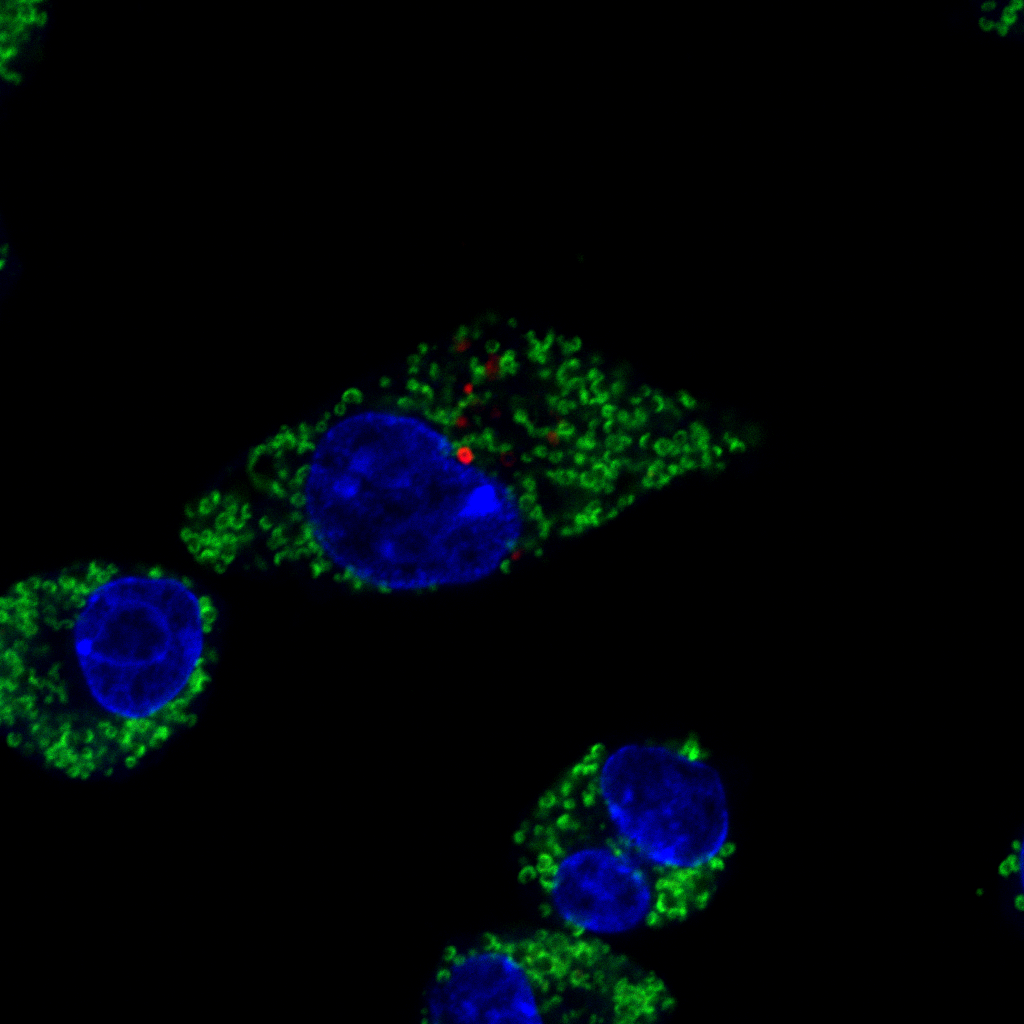

Supplement: Supplementary file 14 — Figure EV5 Source Data [file 44318_2026_817_MOESM14_ESM.zip › EV5C/EV4C-WT_Merge.tif]

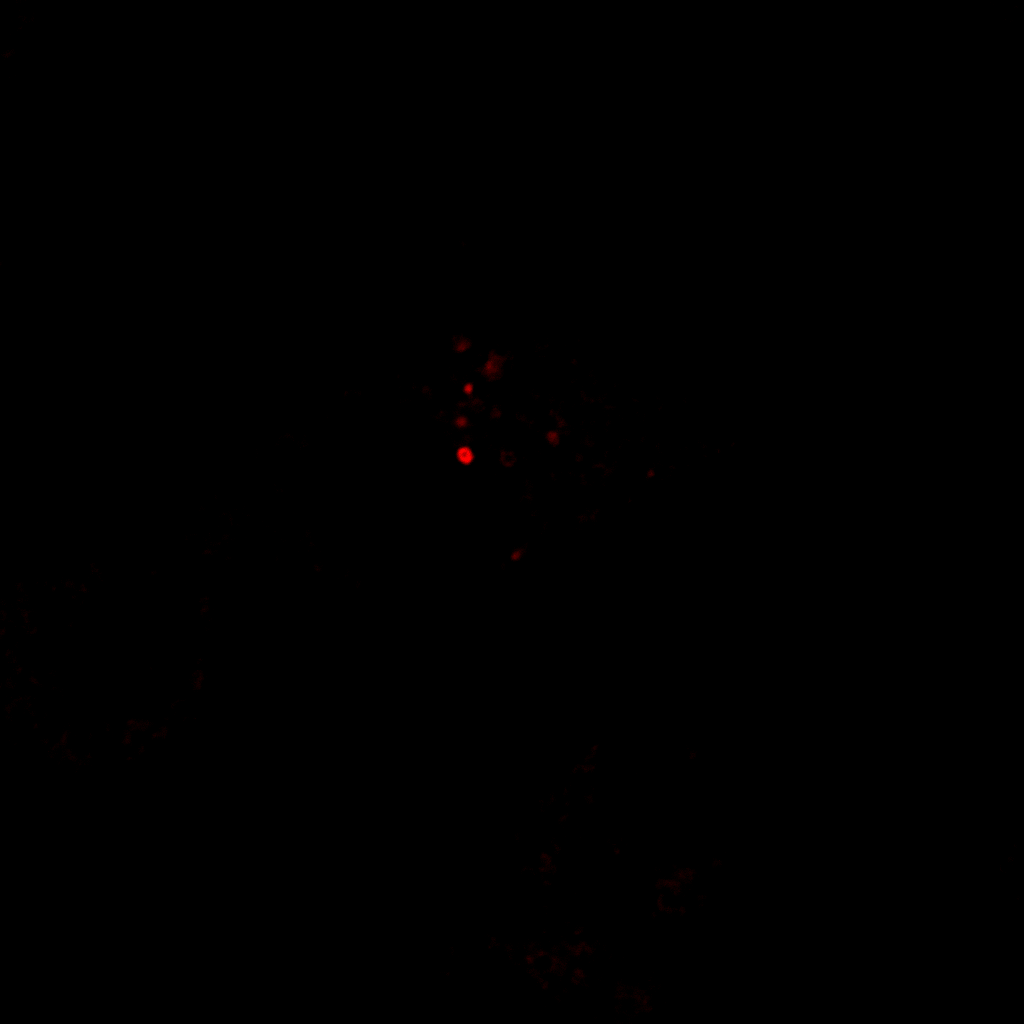

Supplement: Supplementary file 14 — Figure EV5 Source Data [file 44318_2026_817_MOESM14_ESM.zip › EV5C/EV4C-WT_pT72-RAB8A.tif]

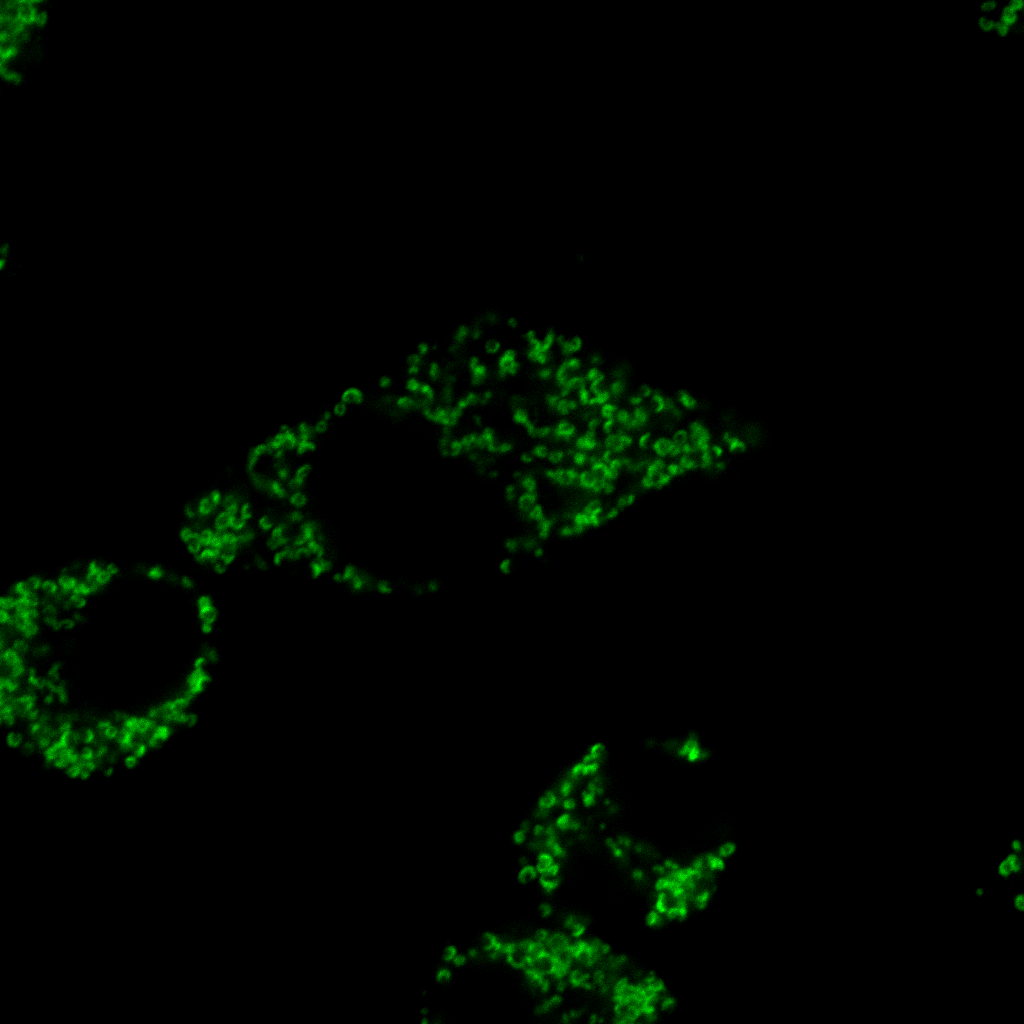

Supplement: Supplementary file 14 — Figure EV5 Source Data [file 44318_2026_817_MOESM14_ESM.zip › EV5C/EV4C-WT_TOMM20.tif]

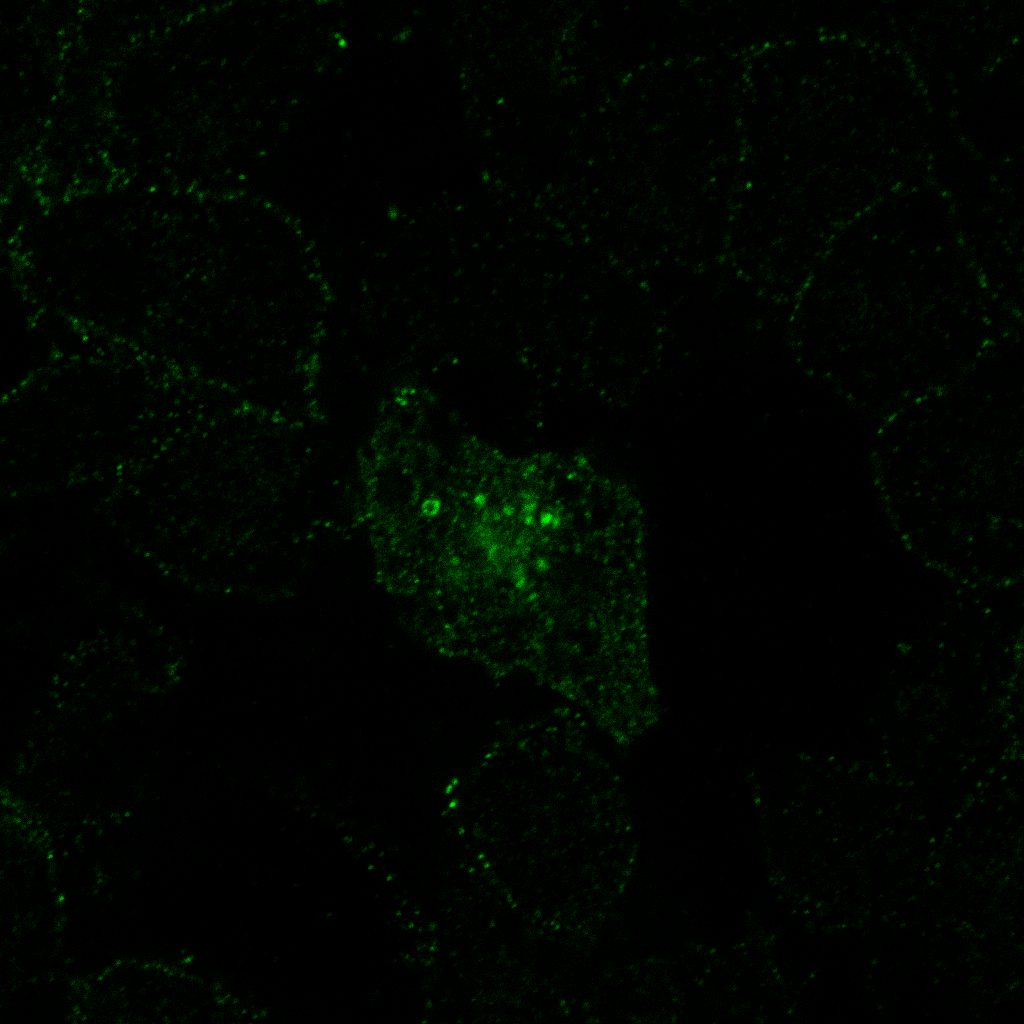

Supplement: Supplementary file 14 — Figure EV5 Source Data [file 44318_2026_817_MOESM14_ESM.zip › EV5D/EV4D-dKO_expressing-GFP-RAB5-_GFP.tif]

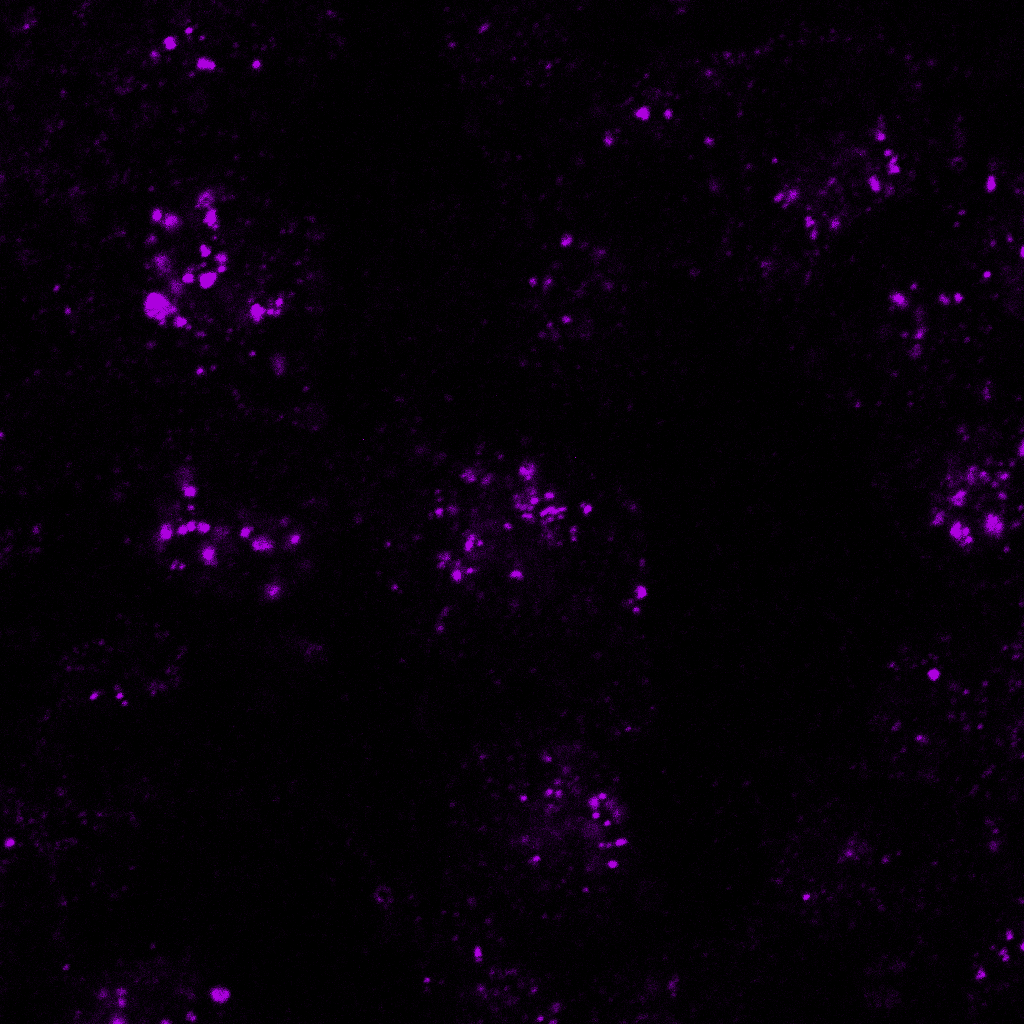

Supplement: Supplementary file 14 — Figure EV5 Source Data [file 44318_2026_817_MOESM14_ESM.zip › EV5D/EV4D-dKO_expressing-GFP-RAB5-_LAMP1.tif]

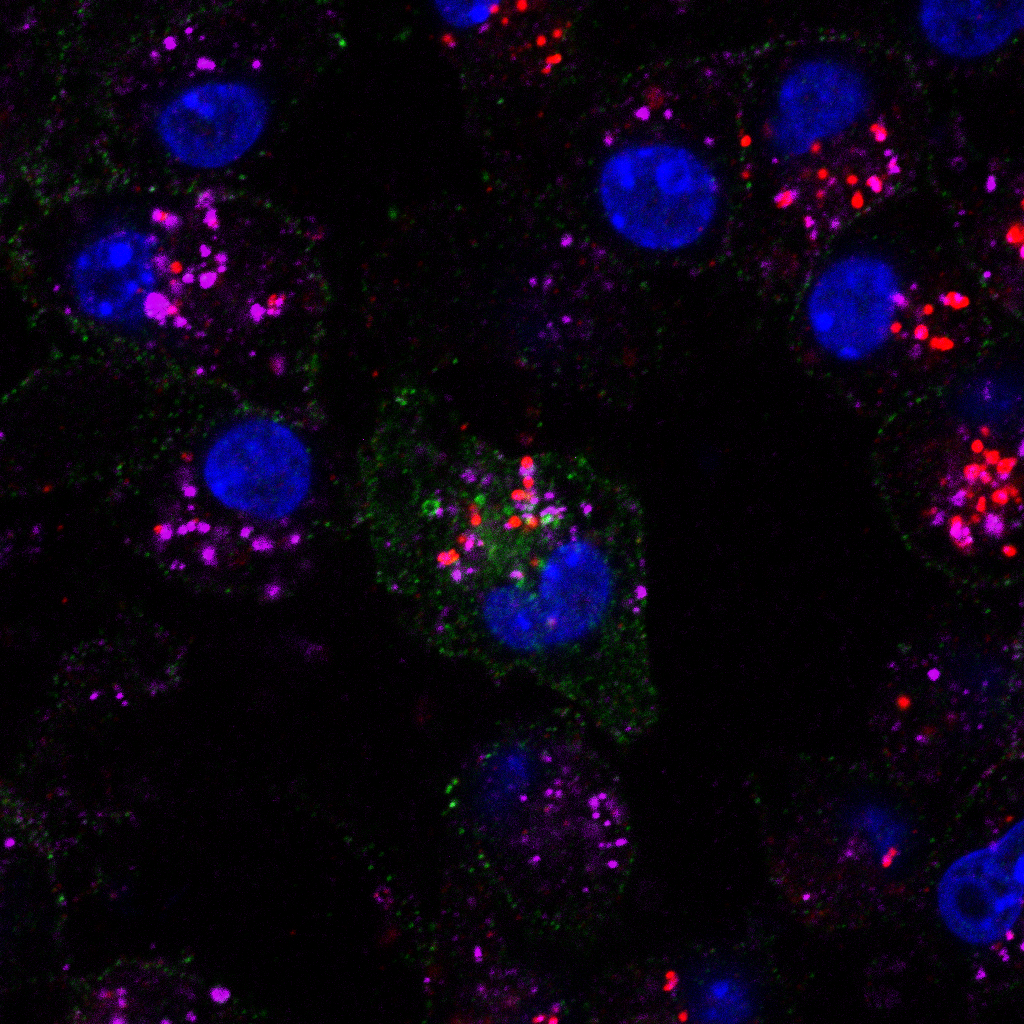

Supplement: Supplementary file 14 — Figure EV5 Source Data [file 44318_2026_817_MOESM14_ESM.zip › EV5D/EV4D-dKO_expressing-GFP-RAB5-_Merge.tif]

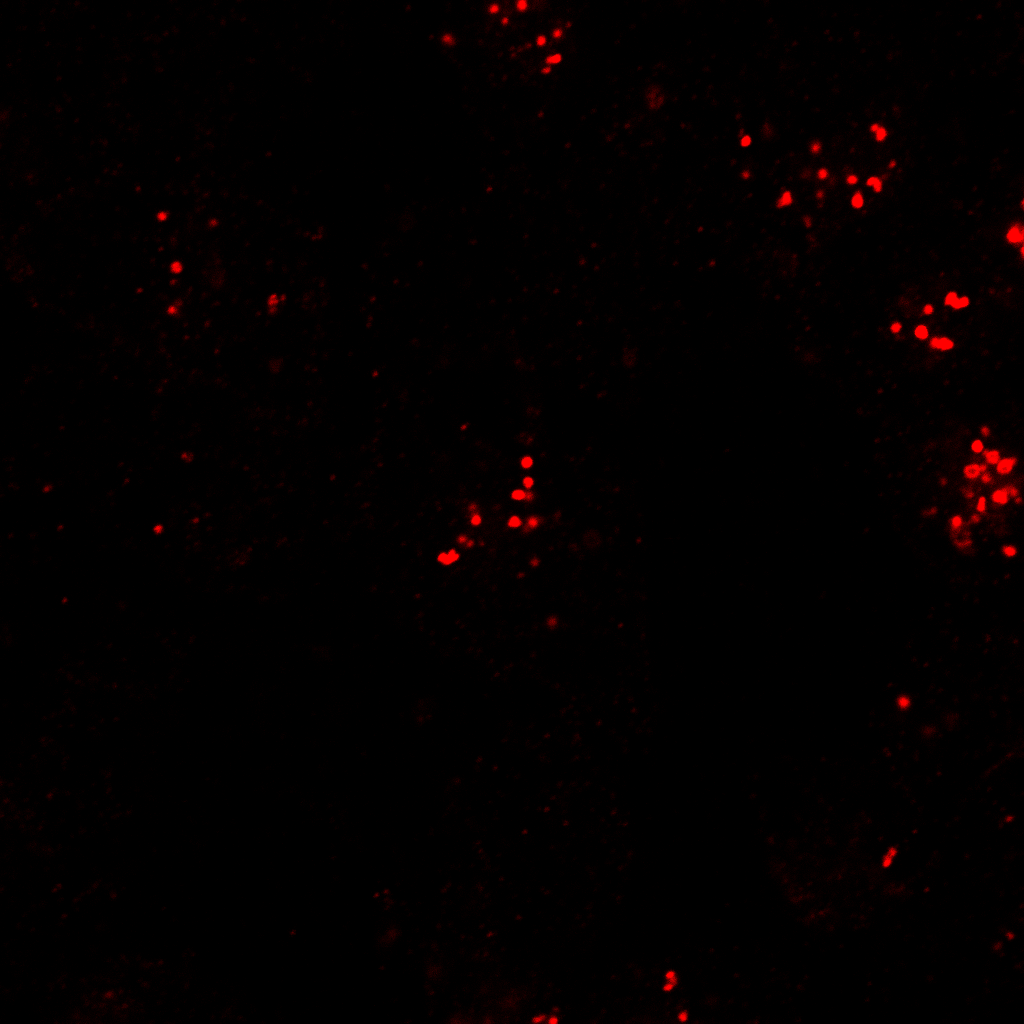

Supplement: Supplementary file 14 — Figure EV5 Source Data [file 44318_2026_817_MOESM14_ESM.zip › EV5D/EV4D-dKO_expressing-GFP-RAB5-_pT72-RAB8A.tif]

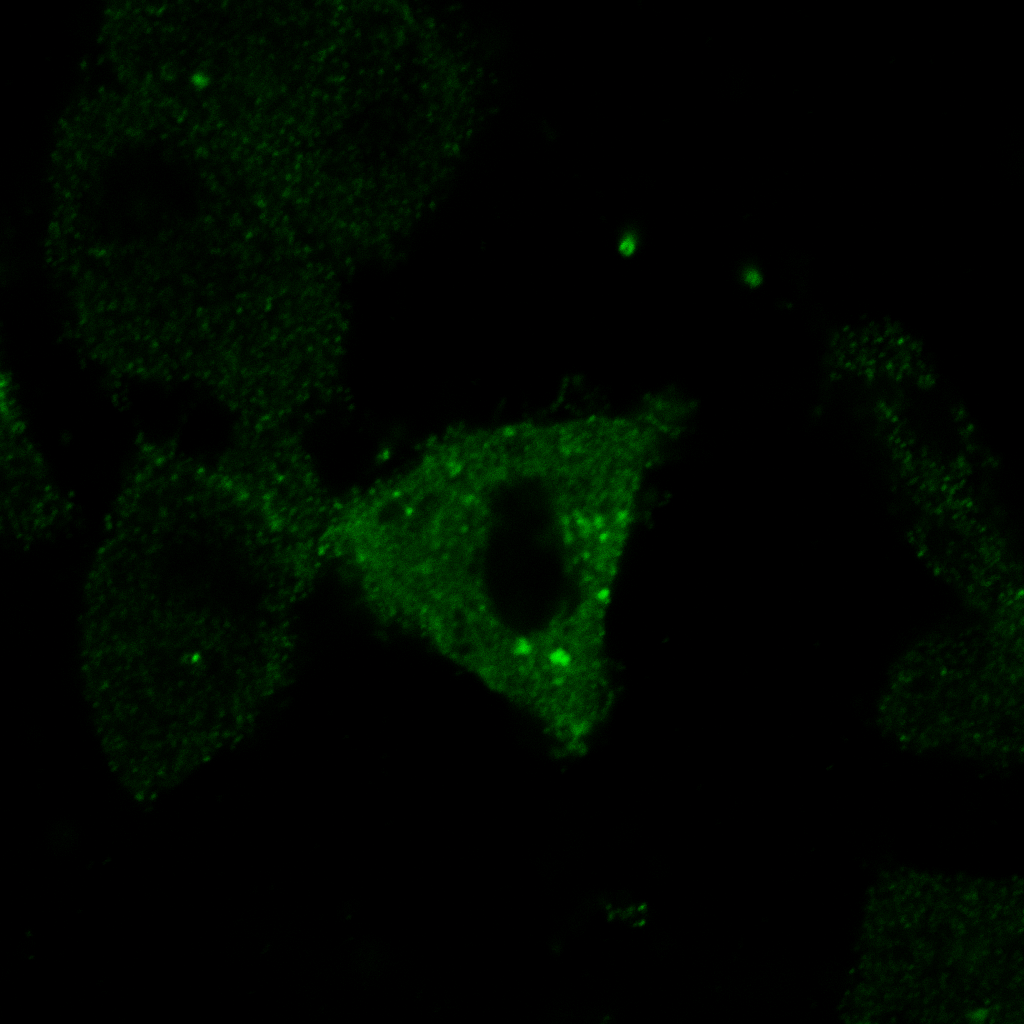

Supplement: Supplementary file 14 — Figure EV5 Source Data [file 44318_2026_817_MOESM14_ESM.zip › EV5D/EV4D-WT_expressing-GFP-RAB5-_GFP.tif]

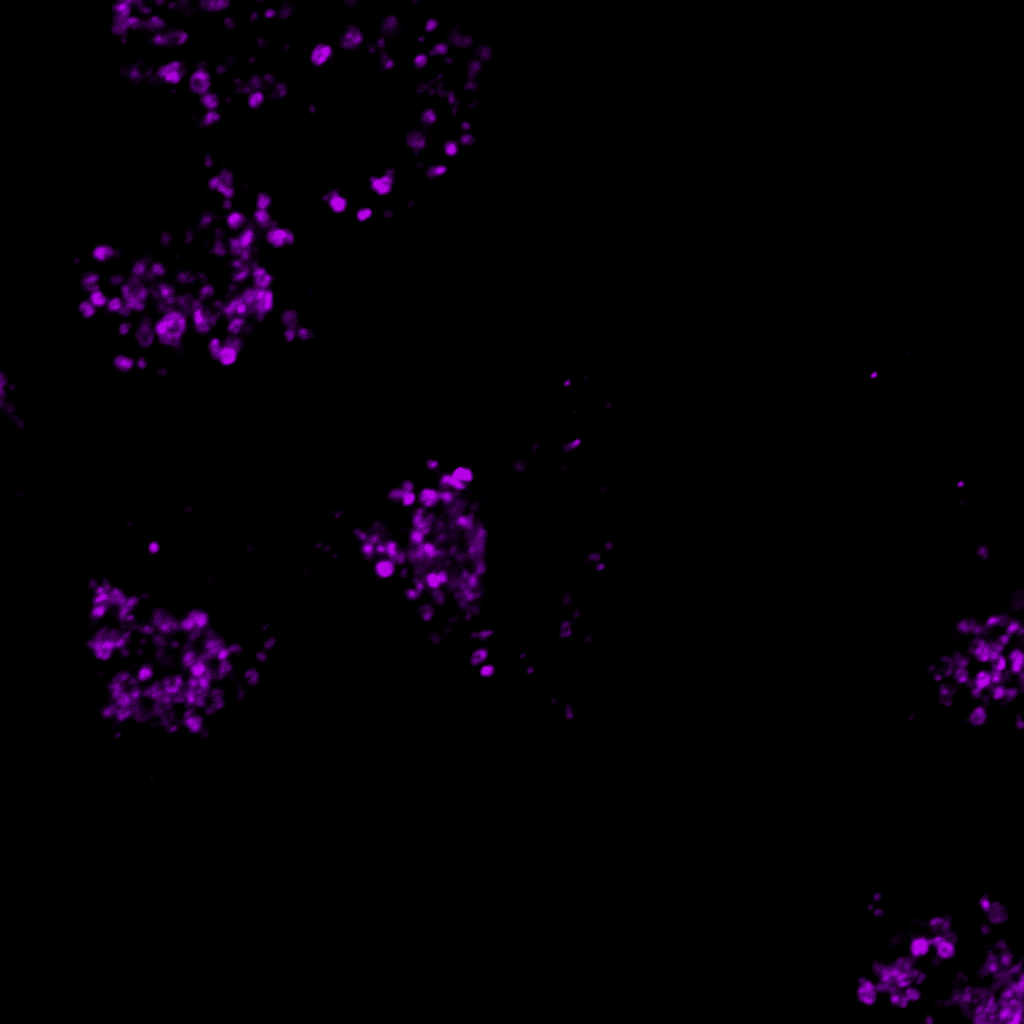

Supplement: Supplementary file 14 — Figure EV5 Source Data [file 44318_2026_817_MOESM14_ESM.zip › EV5D/EV4D-WT_expressing-GFP-RAB5-_LAMP1.tif]

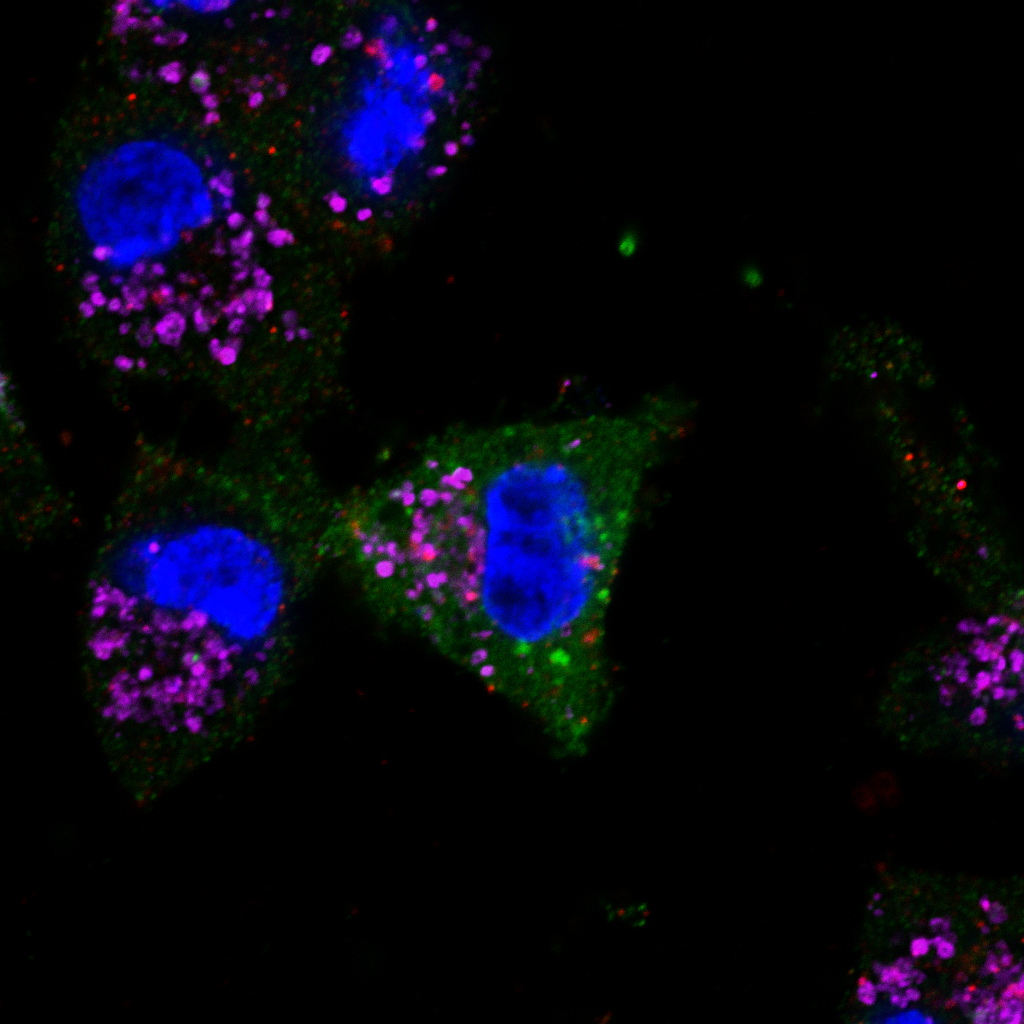

Supplement: Supplementary file 14 — Figure EV5 Source Data [file 44318_2026_817_MOESM14_ESM.zip › EV5D/EV4D-WT_expressing-GFP-RAB5-_Merge.tif]

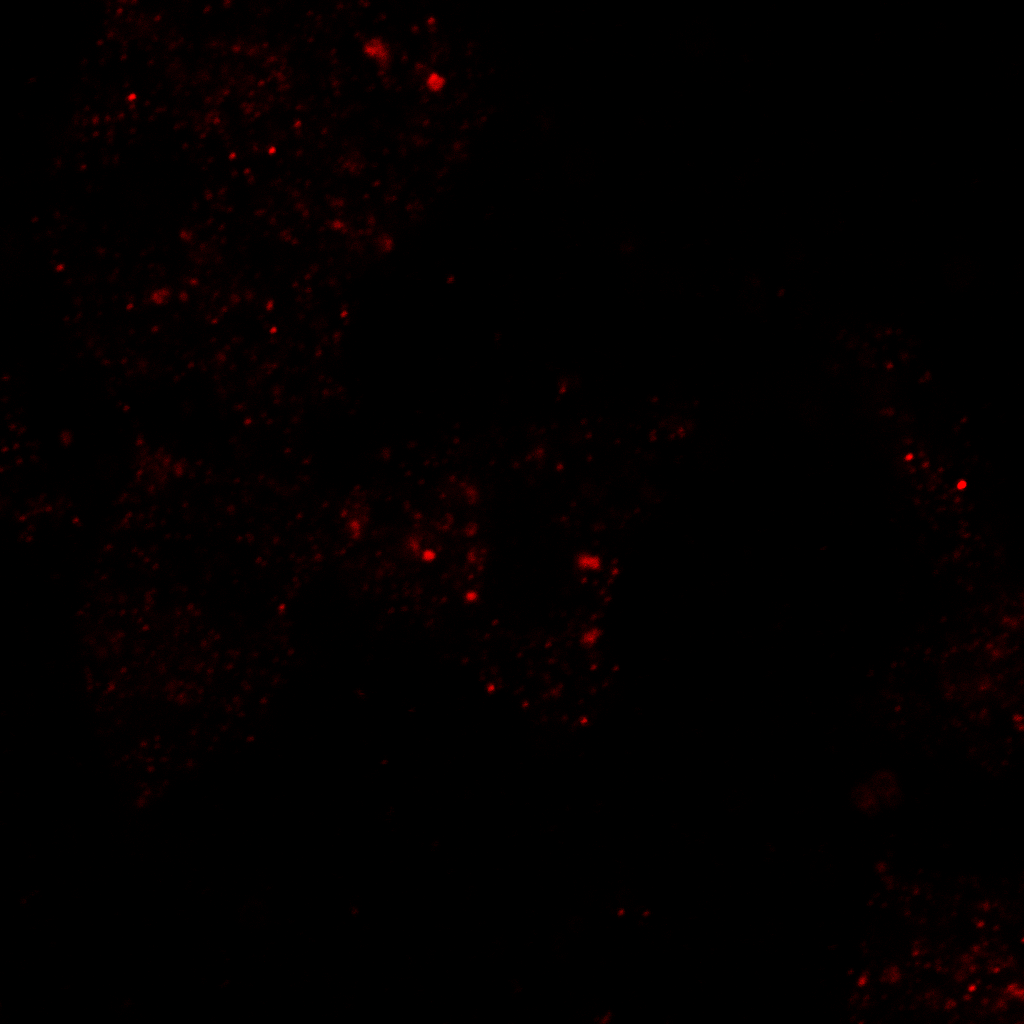

Supplement: Supplementary file 14 — Figure EV5 Source Data [file 44318_2026_817_MOESM14_ESM.zip › EV5D/EV4D-WT_expressing-GFP-RAB5-_pT72-RAB8A.tif]

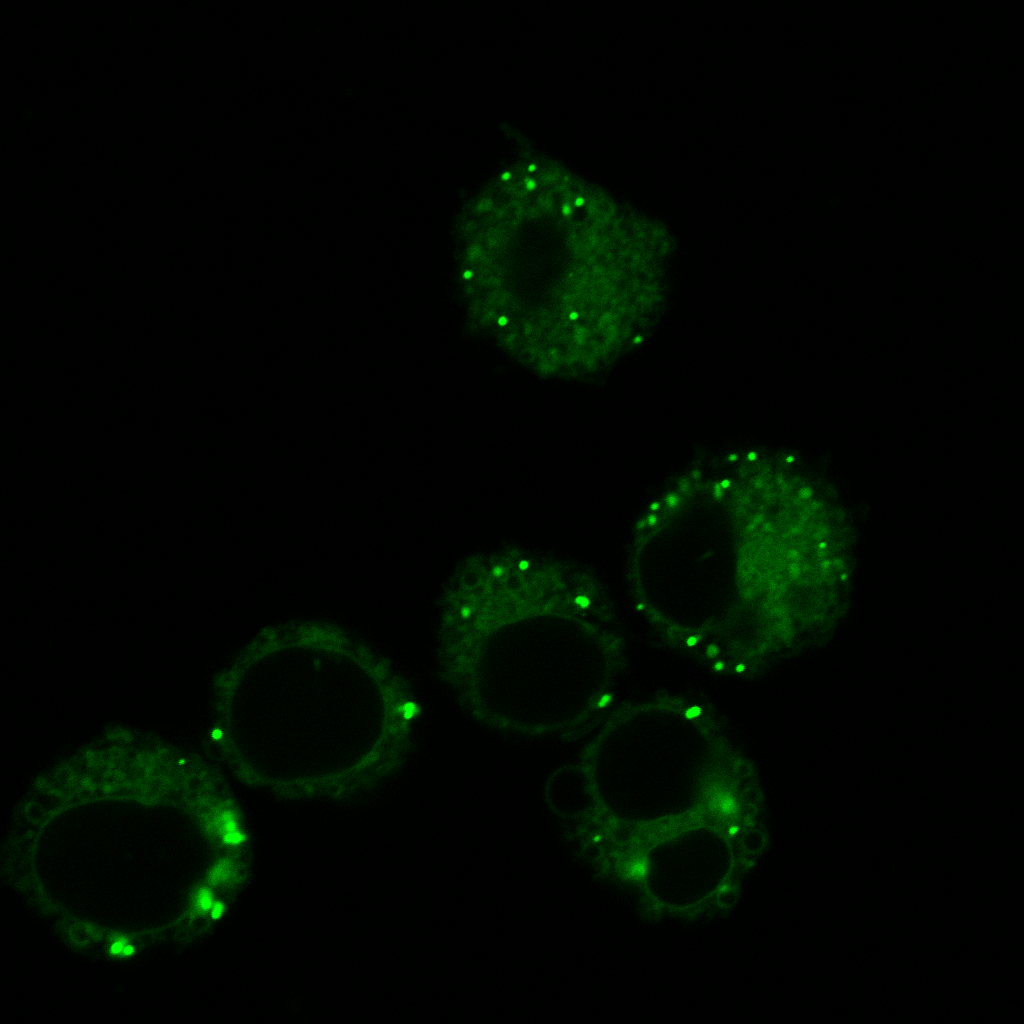

Supplement: Supplementary file 14 — Figure EV5 Source Data [file 44318_2026_817_MOESM14_ESM.zip › EV5E/EV4E-dKO_BIODPY.tif]

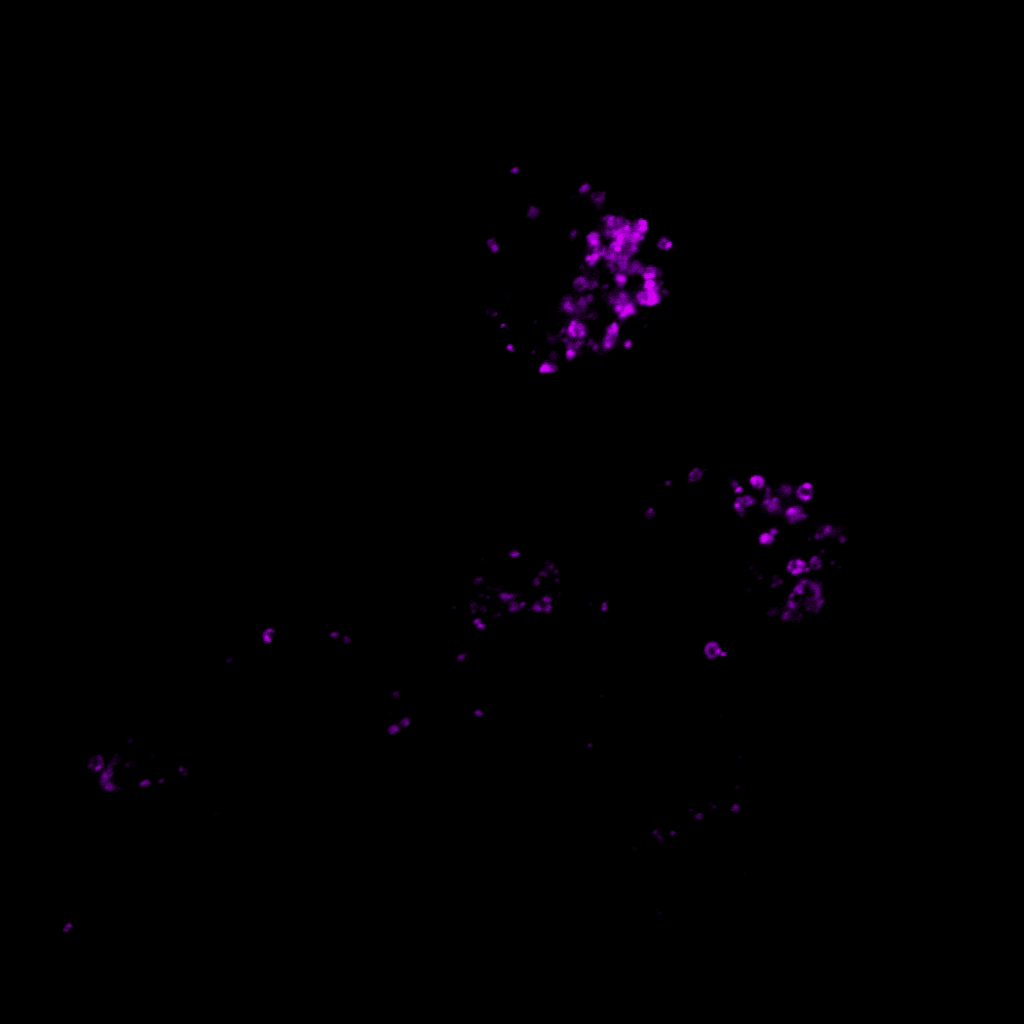

Supplement: Supplementary file 14 — Figure EV5 Source Data [file 44318_2026_817_MOESM14_ESM.zip › EV5E/EV4E-dKO_LAMP1.tif]

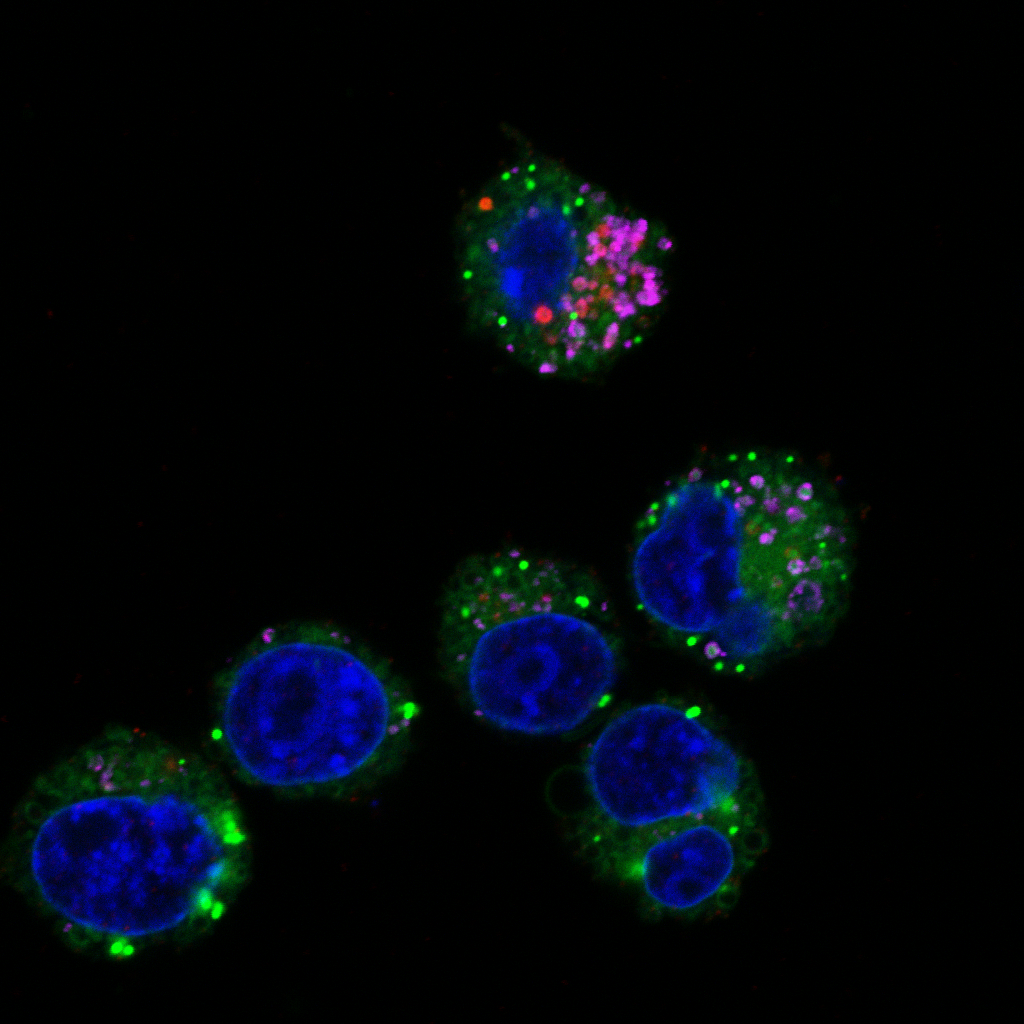

Supplement: Supplementary file 14 — Figure EV5 Source Data [file 44318_2026_817_MOESM14_ESM.zip › EV5E/EV4E-dKO_Merge.tif]

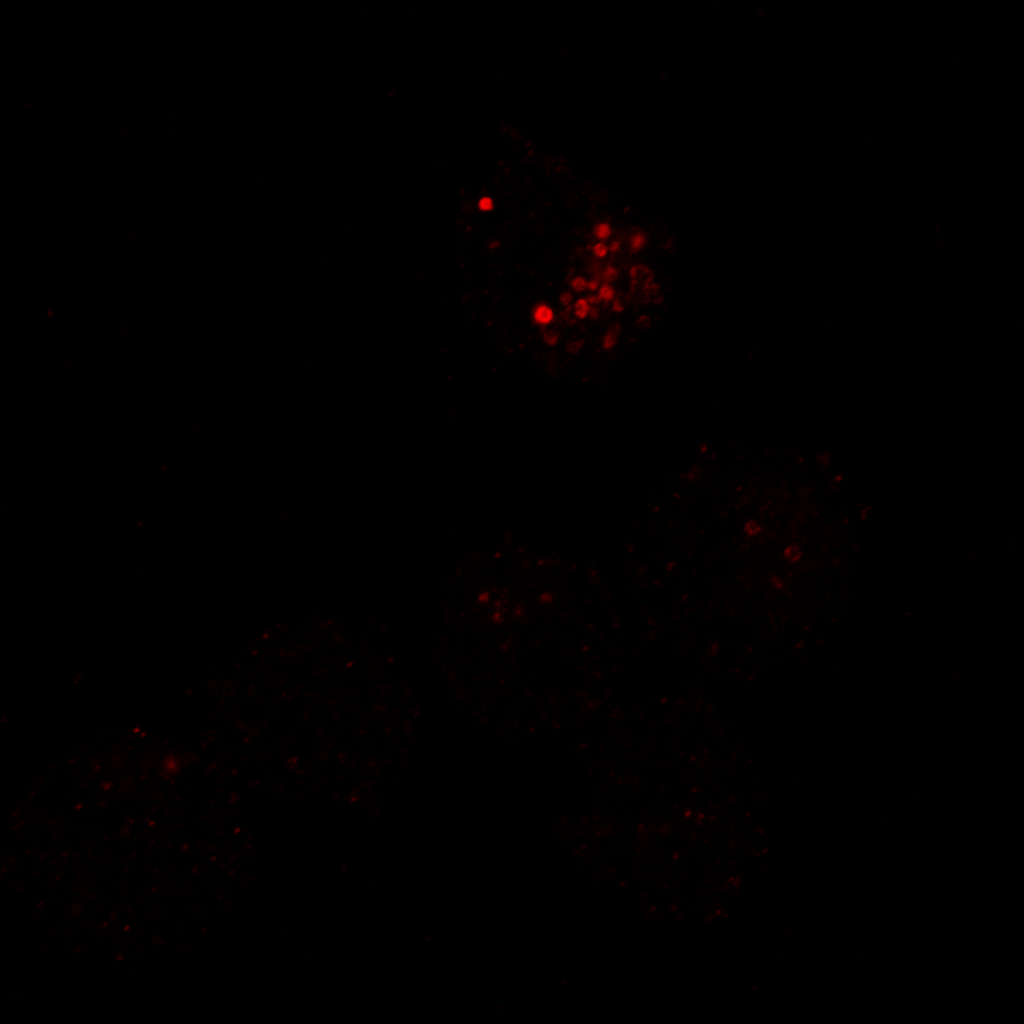

Supplement: Supplementary file 14 — Figure EV5 Source Data [file 44318_2026_817_MOESM14_ESM.zip › EV5E/EV4E-dKO_pT72-RAB8A.tif]

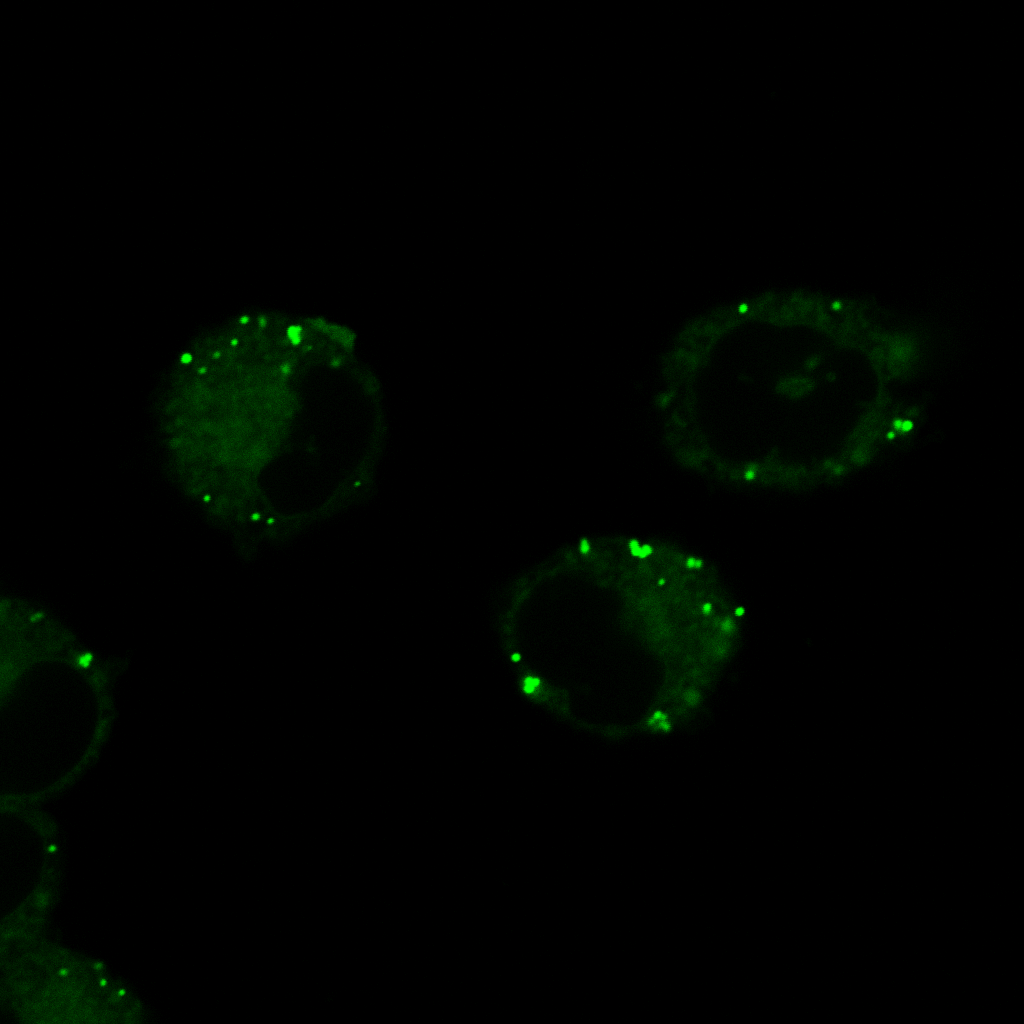

Supplement: Supplementary file 14 — Figure EV5 Source Data [file 44318_2026_817_MOESM14_ESM.zip › EV5E/EV4E-WT_BIODPY.tif]

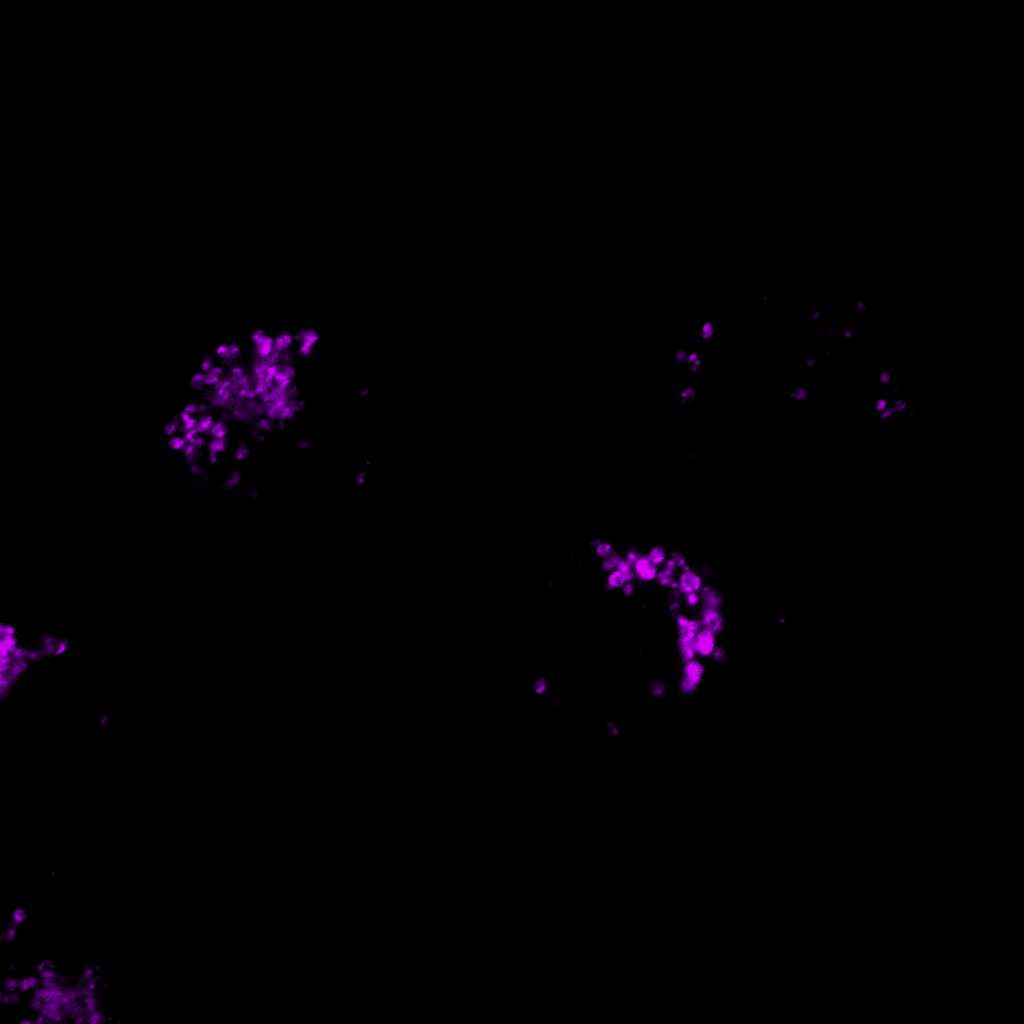

Supplement: Supplementary file 14 — Figure EV5 Source Data [file 44318_2026_817_MOESM14_ESM.zip › EV5E/EV4E-WT_LAMP1.tif]

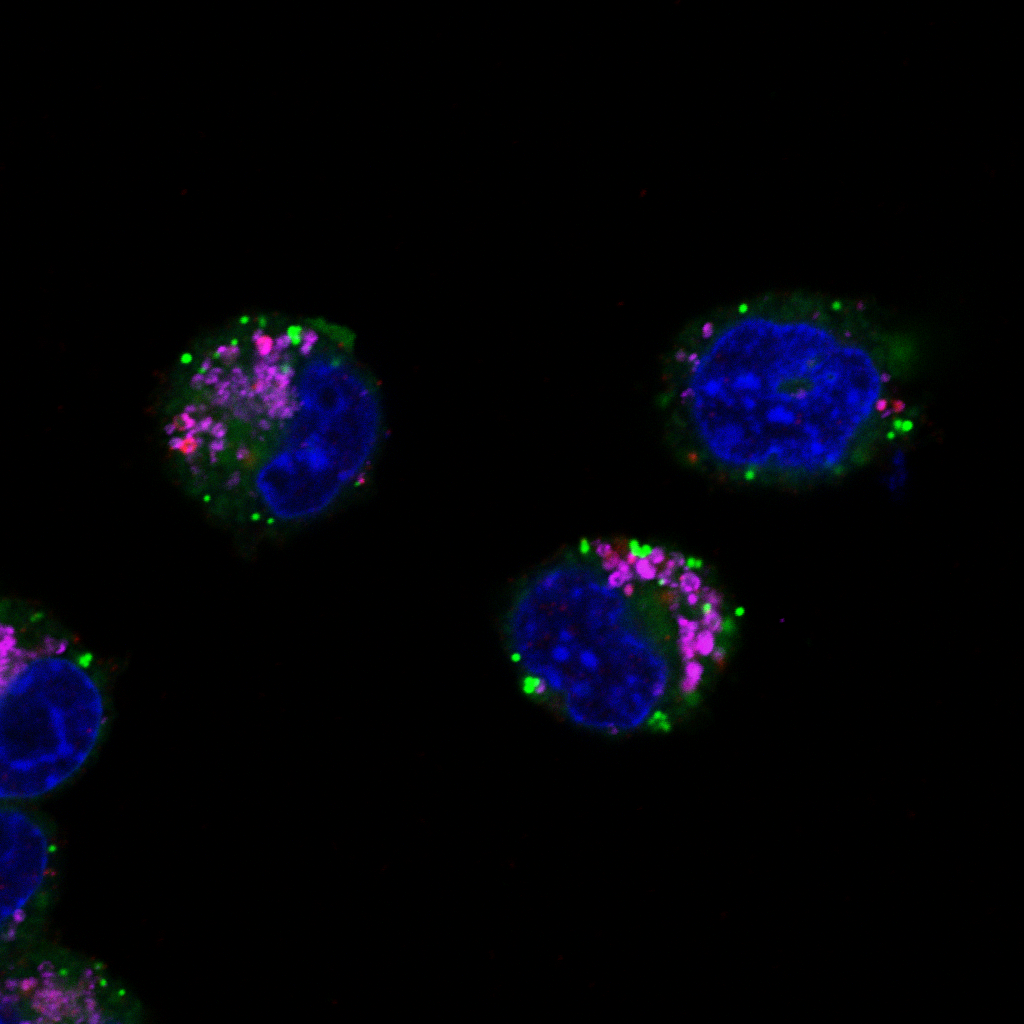

Supplement: Supplementary file 14 — Figure EV5 Source Data [file 44318_2026_817_MOESM14_ESM.zip › EV5E/EV4E-WT_Merge.tif]

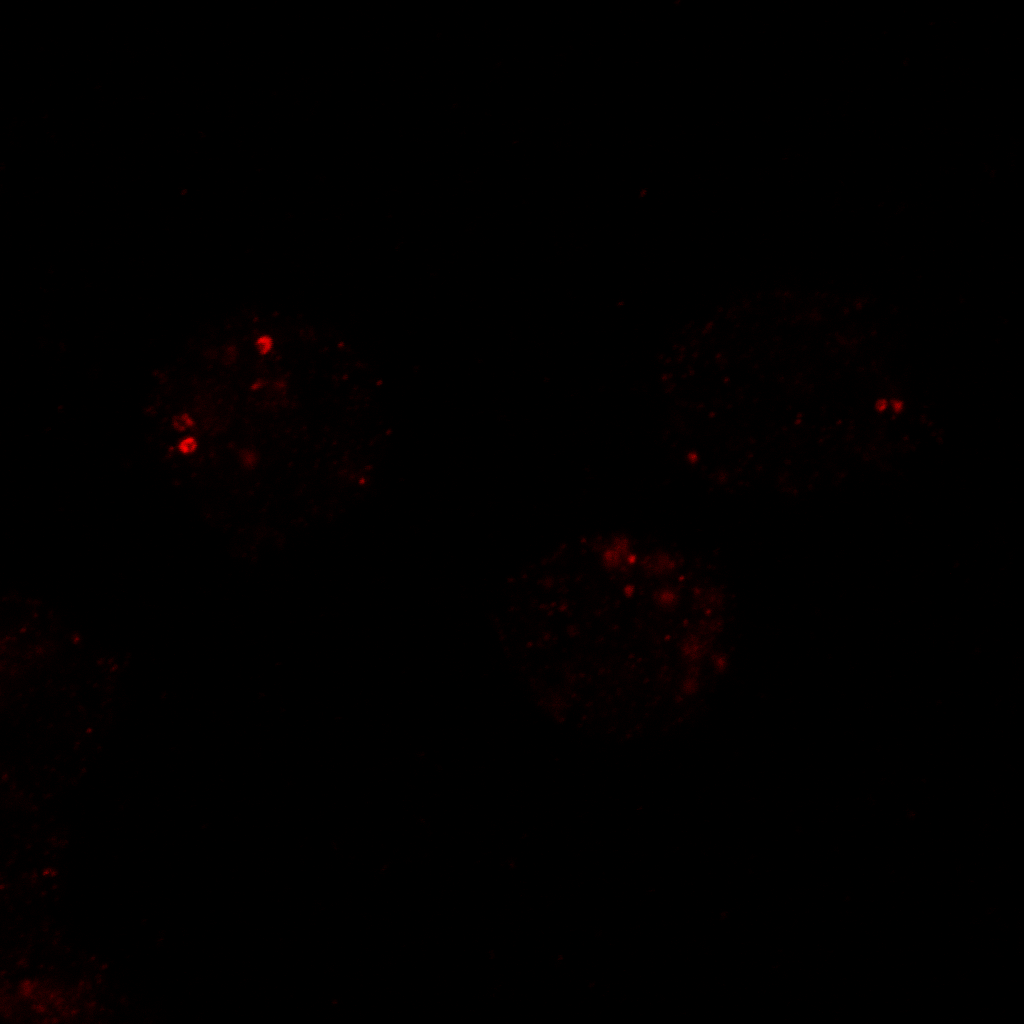

Supplement: Supplementary file 14 — Figure EV5 Source Data [file 44318_2026_817_MOESM14_ESM.zip › EV5E/EV4E-WT_pT72-RAB8A.tif]

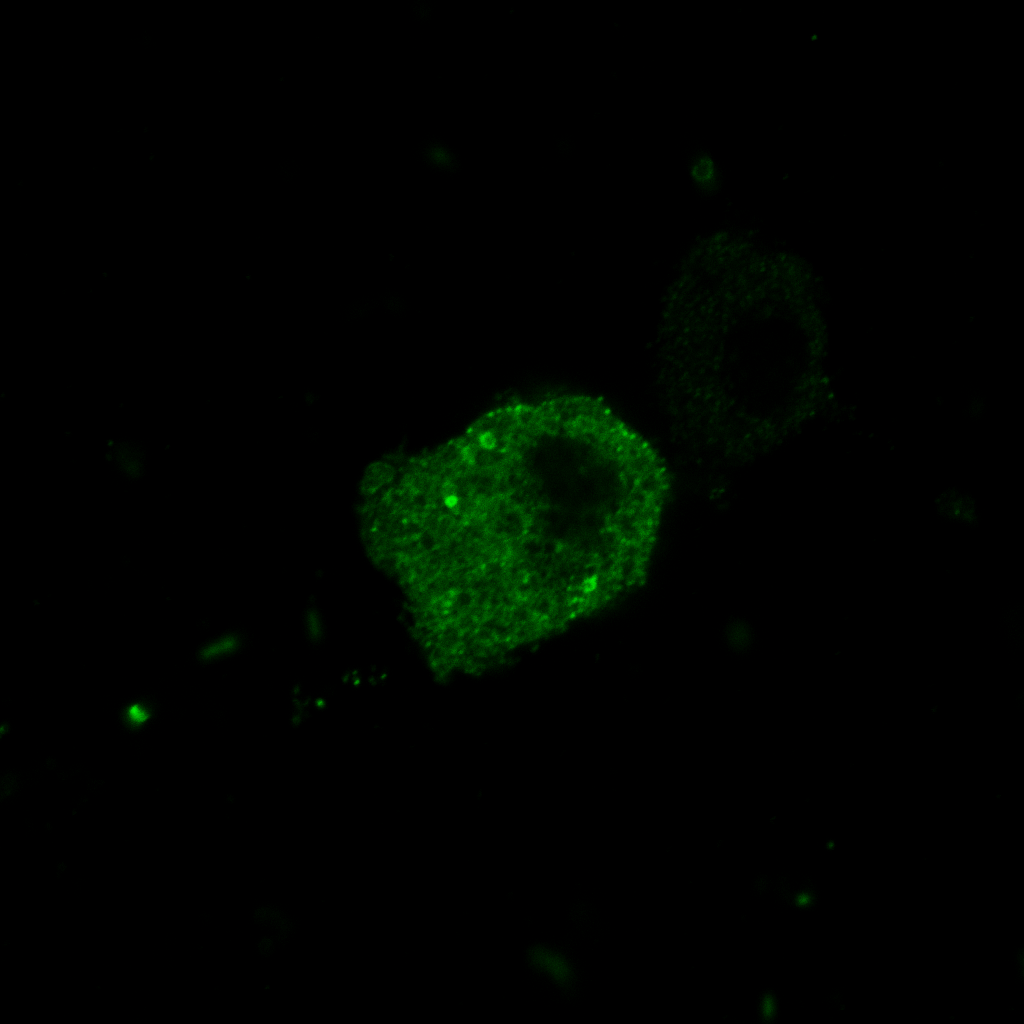

Supplement: Supplementary file 14 — Figure EV5 Source Data [file 44318_2026_817_MOESM14_ESM.zip › EV5F/EV4F-dKO_expressing-GFP-RAB11-_GFP.tif]

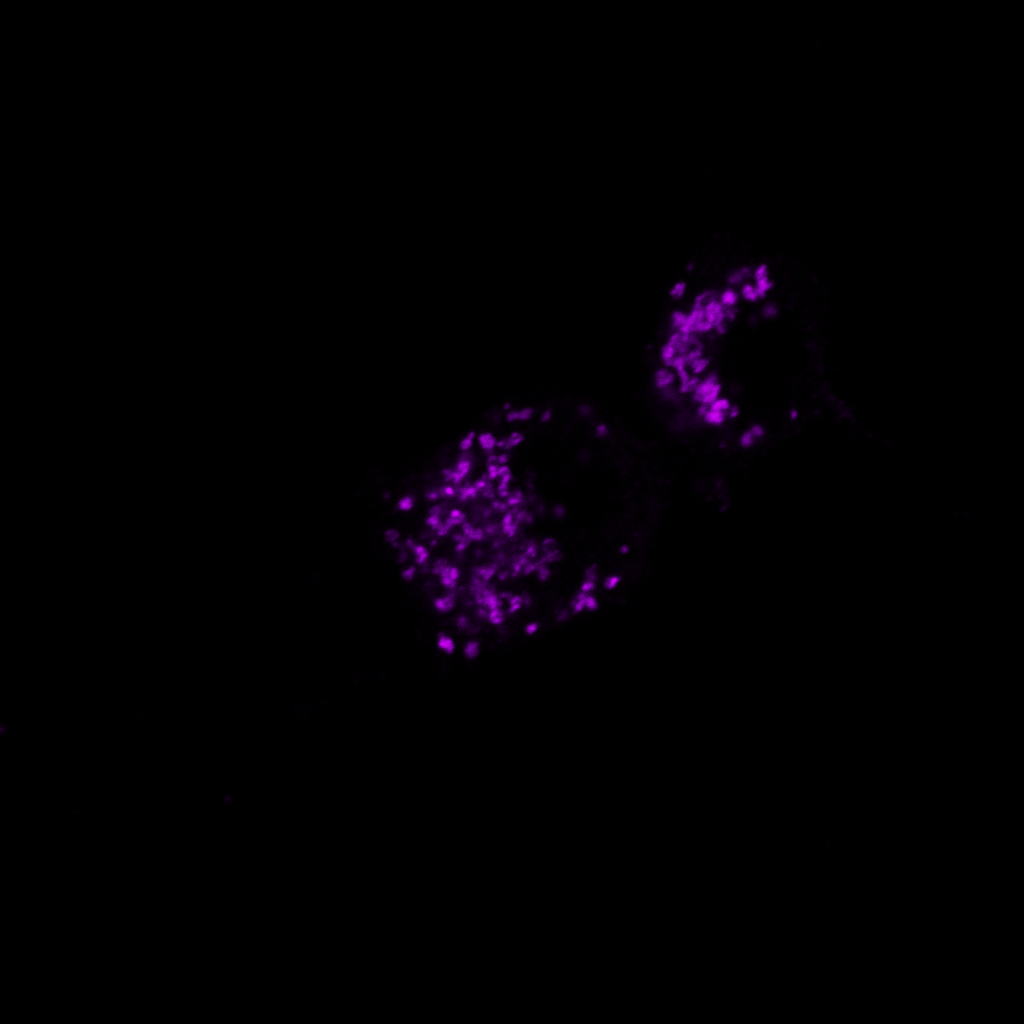

Supplement: Supplementary file 14 — Figure EV5 Source Data [file 44318_2026_817_MOESM14_ESM.zip › EV5F/EV4F-dKO_expressing-GFP-RAB11-_LAMP1.tif]

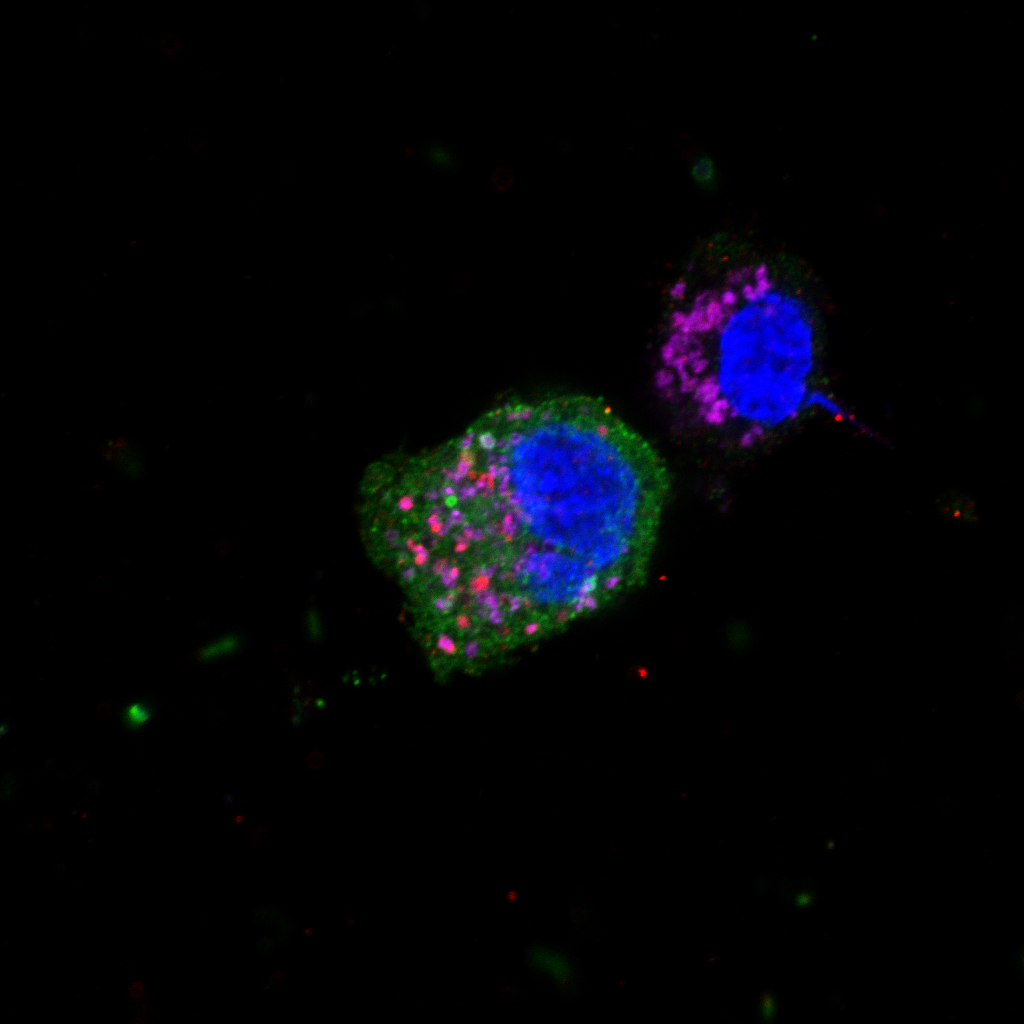

Supplement: Supplementary file 14 — Figure EV5 Source Data [file 44318_2026_817_MOESM14_ESM.zip › EV5F/EV4F-dKO_expressing-GFP-RAB11-_Merge.tif]

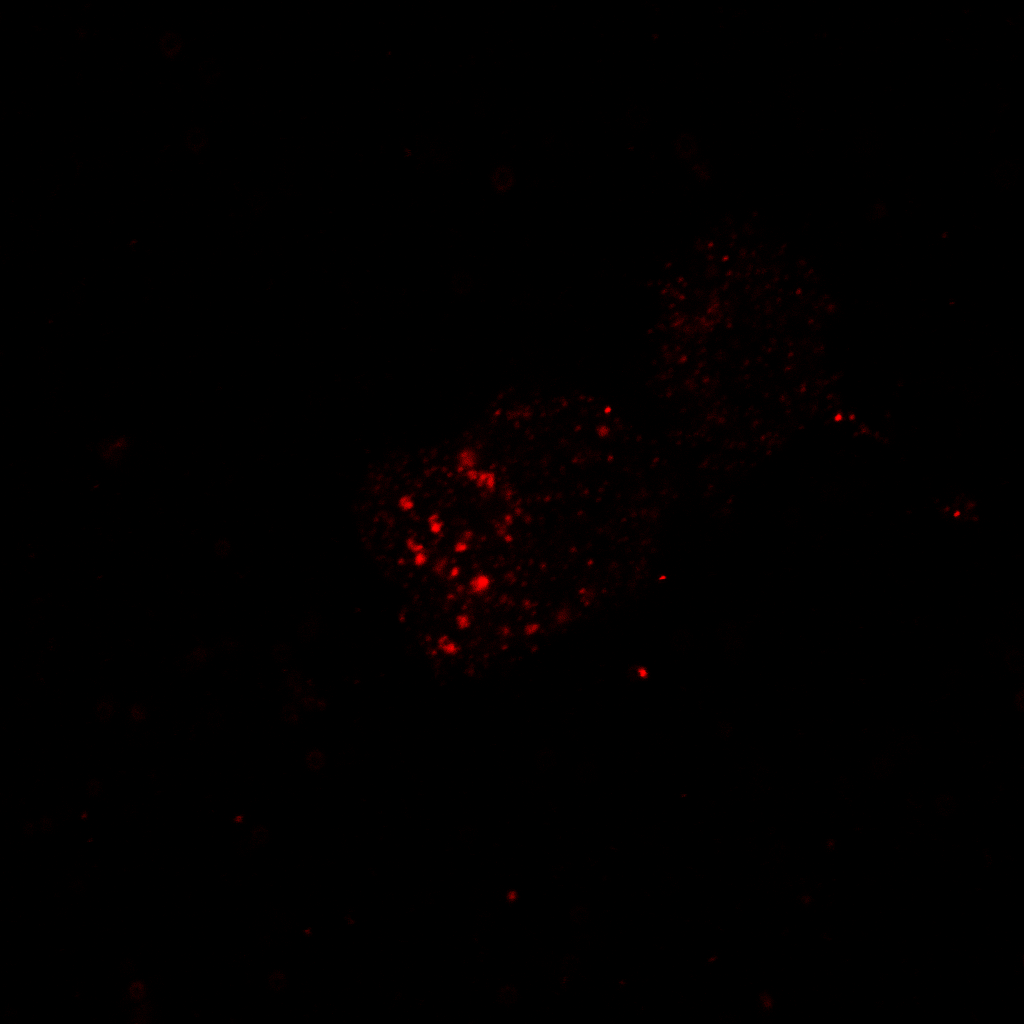

Supplement: Supplementary file 14 — Figure EV5 Source Data [file 44318_2026_817_MOESM14_ESM.zip › EV5F/EV4F-dKO_expressing-GFP-RAB11-_pT72-RAB8A.tif]

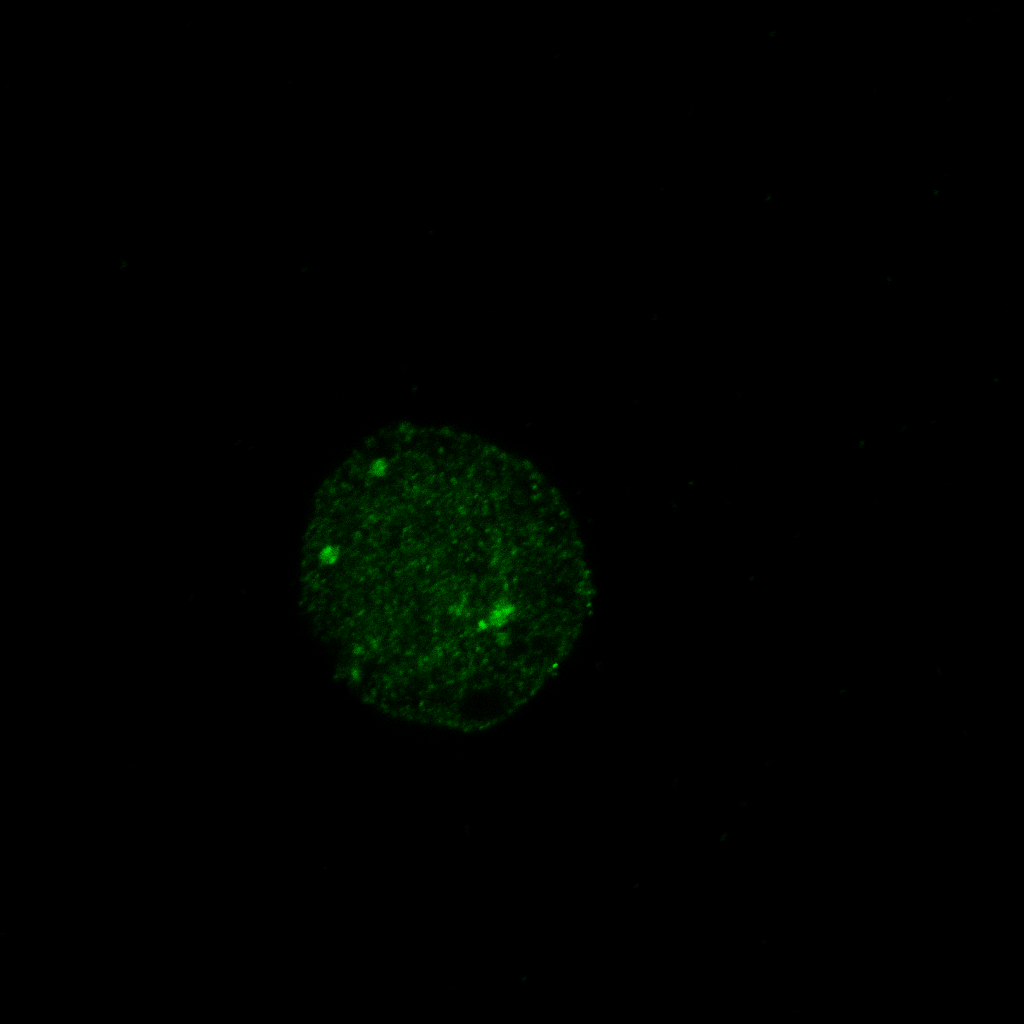

Supplement: Supplementary file 14 — Figure EV5 Source Data [file 44318_2026_817_MOESM14_ESM.zip › EV5F/EV4F-WT_expressing-GFP-RAB11-_GFP.tif]

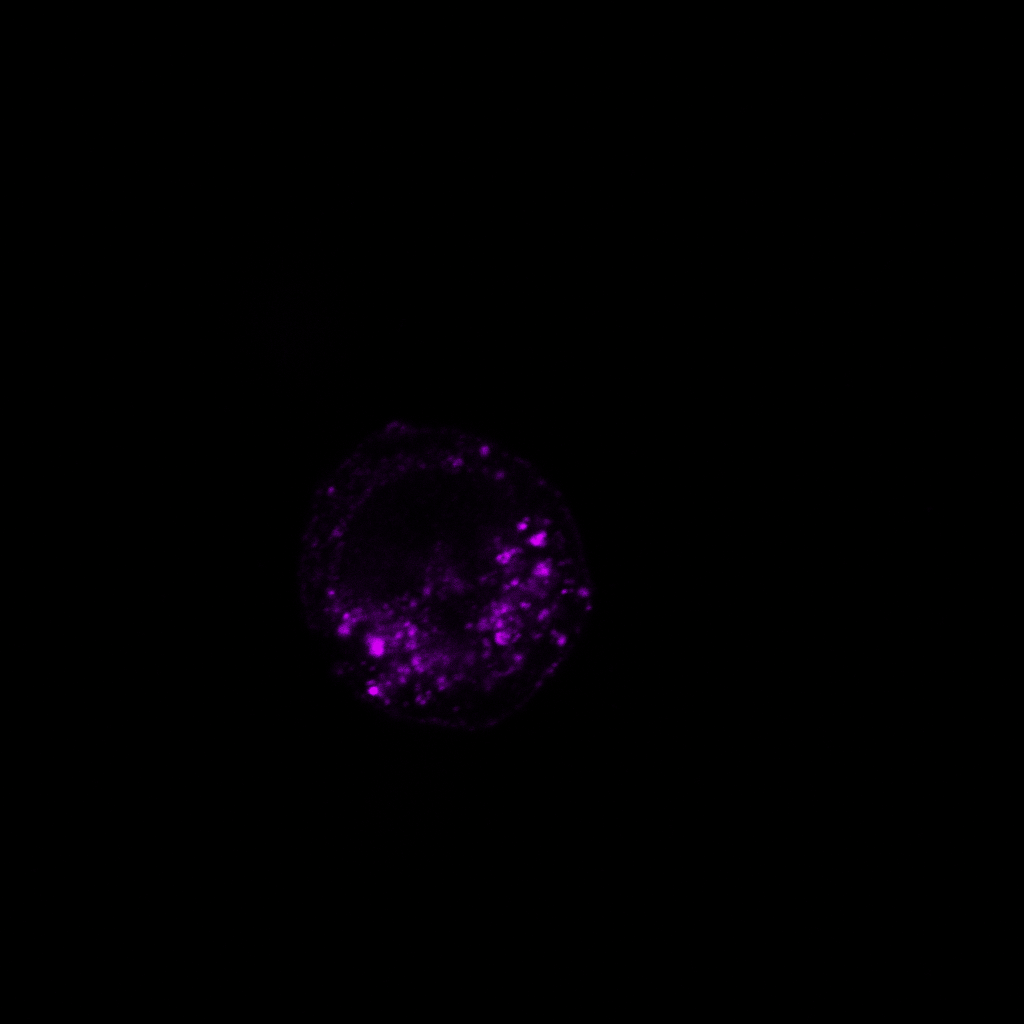

Supplement: Supplementary file 14 — Figure EV5 Source Data [file 44318_2026_817_MOESM14_ESM.zip › EV5F/EV4F-WT_expressing-GFP-RAB11-_LAMP1.tif]

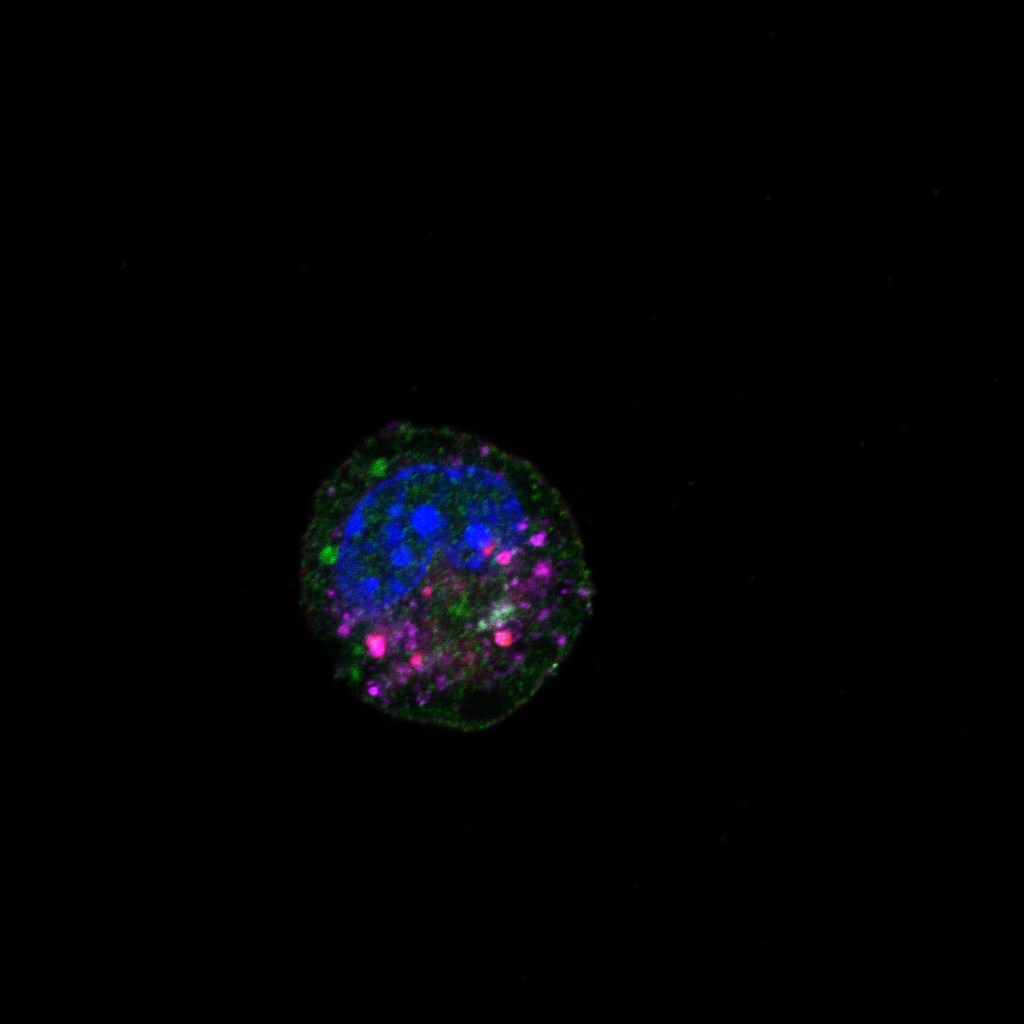

Supplement: Supplementary file 14 — Figure EV5 Source Data [file 44318_2026_817_MOESM14_ESM.zip › EV5F/EV4F-WT_expressing-GFP-RAB11-_Merge.tif]

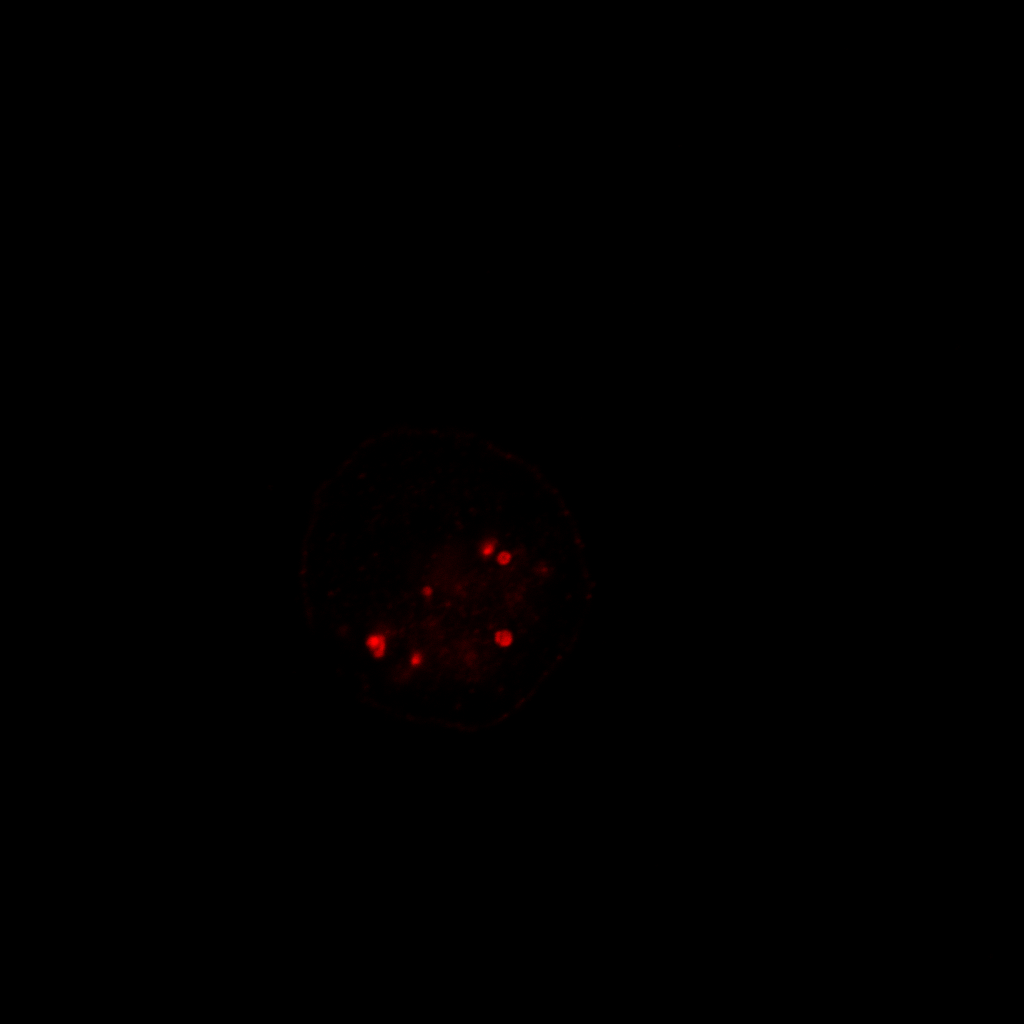

Supplement: Supplementary file 14 — Figure EV5 Source Data [file 44318_2026_817_MOESM14_ESM.zip › EV5F/EV4F-WT_expressing-GFP-RAB11-_pT72-RAB8A.tif]
