# Supplementary material for: The C9orf72/SMCR8 complex maintains microglial homeostasis via RAB8A-ESCRT-mediated lysosomal repair
Source: EMBO J. 2026 May 29;45(13):4531–68. doi: 10.1038/s44318-026-00817-w (PMC13324726; doi:10.1038/s44318-026-00817-w)
Supplement: Supplementary file 15 — Expanded View Figures [file 44318_2026_817_MOESM15_ESM.pdf]

Expanded View Figures

Figure EV1. C9orf72 and SMCR8 are highly expressed in microglia and exhibit age-dependent upregulation in the CNS.

(**A**) Violin plots showing *C9orf72* and *Smcr8* expression levels across major CNS cell types from single-cell RNA-sequencing dataset (Yao et al, 2021; $n = 54,390$ cells). (**B**, **C**) Immunoblotting analysis of C9orf72 and SMCR8 protein levels in primary neurons and microglia (**B**). GAPDH serves as loading control. Quantification of protein levels normalized to GAPDH is shown in (**C**) ($n = 3$ independent experiments, SMCR8: $P = 0.0472$, C9orf72: $P = 0.0008$). (**D**) Validation of knockout efficiency in C9orf72/SMCR8 dKO mice by Western blot analysis of brain and spinal cord tissue lysates. Representative blots show protein expression in WT, heterozygous (Het), and dKO genotypes. GAPDH serves as loading control. Asterisks indicate nonspecific bands. (**E**, **F**) Age-dependent expression of SMCR8 and C9orf72 in brain (**E**) and spinal cord (**F**) from WT mice at 4, 12, and 20 months of age. β-Actin serves as loading control. Asterisks indicate nonspecific bands. (**G–J**) Representative immunofluorescence images of IBA1 (green) in brain (**G**) and spinal cord (**I**) sections from 20-month-old WT and dKO mice, with three-dimensional surface reconstructions of microglia (Imaris Surface module). Scale bar, 5 µm. Quantitative analysis of microglial branch number (branch points) and process length in brain (**H**: branch points, $P = 0.0038$; process length, $P = 0.0192$) and spinal cord (**J**: branch points, $P = 0.0125$; process length, $P = 0.0147$) using Imaris Filament module ($n = 40$–45 cells per genotype from three independent experiments). (**K–N**) Representative confocal images of NeuN immunostaining (green) and DAPI (blue) in the hippocampus (**K**), and ChAT immunostaining (green) and DAPI (blue) in the spinal cord (**L**) of 12-month-old WT, *C9orf72* KO, *Smcr8* KO, and dKO mice, with quantification of NeuN-positive neurons in the hippocampus (**M**) and ChAT-positive motor neurons in the spinal cord (**N**). Boxed areas are enlarged below; white dotted lines in (**K**) indicate part of the CA3 subregion. Scale bars, 200 µm (main), 50 µm (**K**, insets), 100 µm (**L**, insets). $n = 3$ mice per group. Hippocampus (**M**): WT vs *C9orf72* KO: $P = 0.4718$; WT vs *Smcr8* KO: $P = 0.5031$; WT vs dKO: $P = 0.0194$. Spinal cord (**N**): WT vs *C9orf72* KO: $P = 0.1848$; WT vs *Smcr8* KO: $P = 0.055$; WT vs dKO: $P = 0.0139$. Data information: In (**C**, **H**, **J**, **M**, **N**), data are presented as mean ± SEM from three independent experiments. Statistical significance was assessed by unpaired two-tailed Student's *t* test. $^*P < 0.05$, $^{**}P < 0.01$; ns, not significant.

© The Author(s)

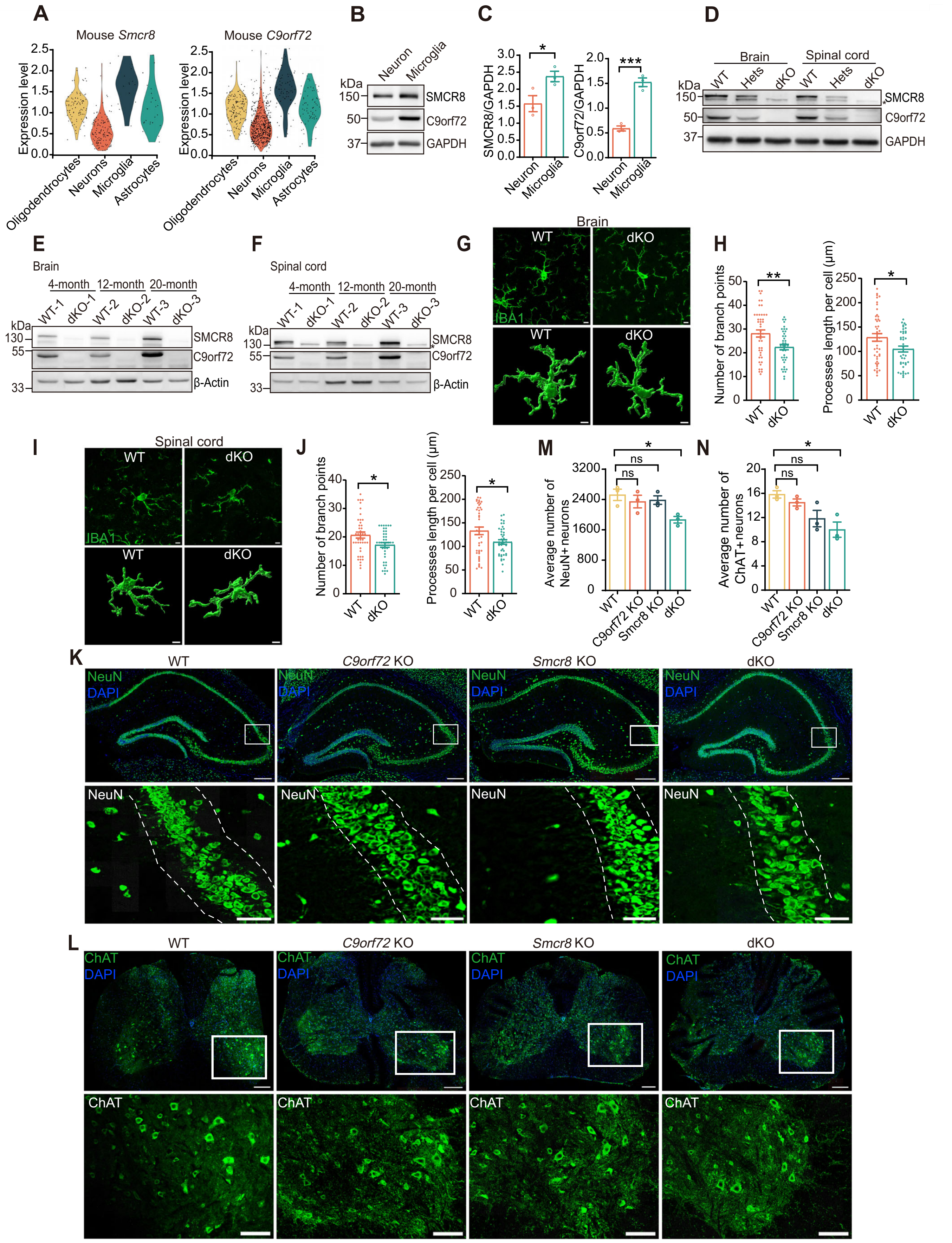
A
Mouse Smcr8
Mouse C9orf72
Expression level
Oligodendrocytes
Neurons
Microglia
Astrocytes
B
Neuron
Microglia
SMCR8
C9orf72
GAPDH
C
SMCR8/GAPDH
C9orf72/GAPDH
D
Brain
Spinal cord
WT
Hets
dKO
E
Brain
4-month
12-month
20-month
β-Actin
F
Spinal cord
G
IBA1
H
Number of branch points
Processes length per cell (μm)
I
J
M
Average number of NeuN+ neurons
C9orf72 KO
Smcr8 KO
N
Average number of ChAT+neurons
K
NeuN
DAPI
L
ChAT

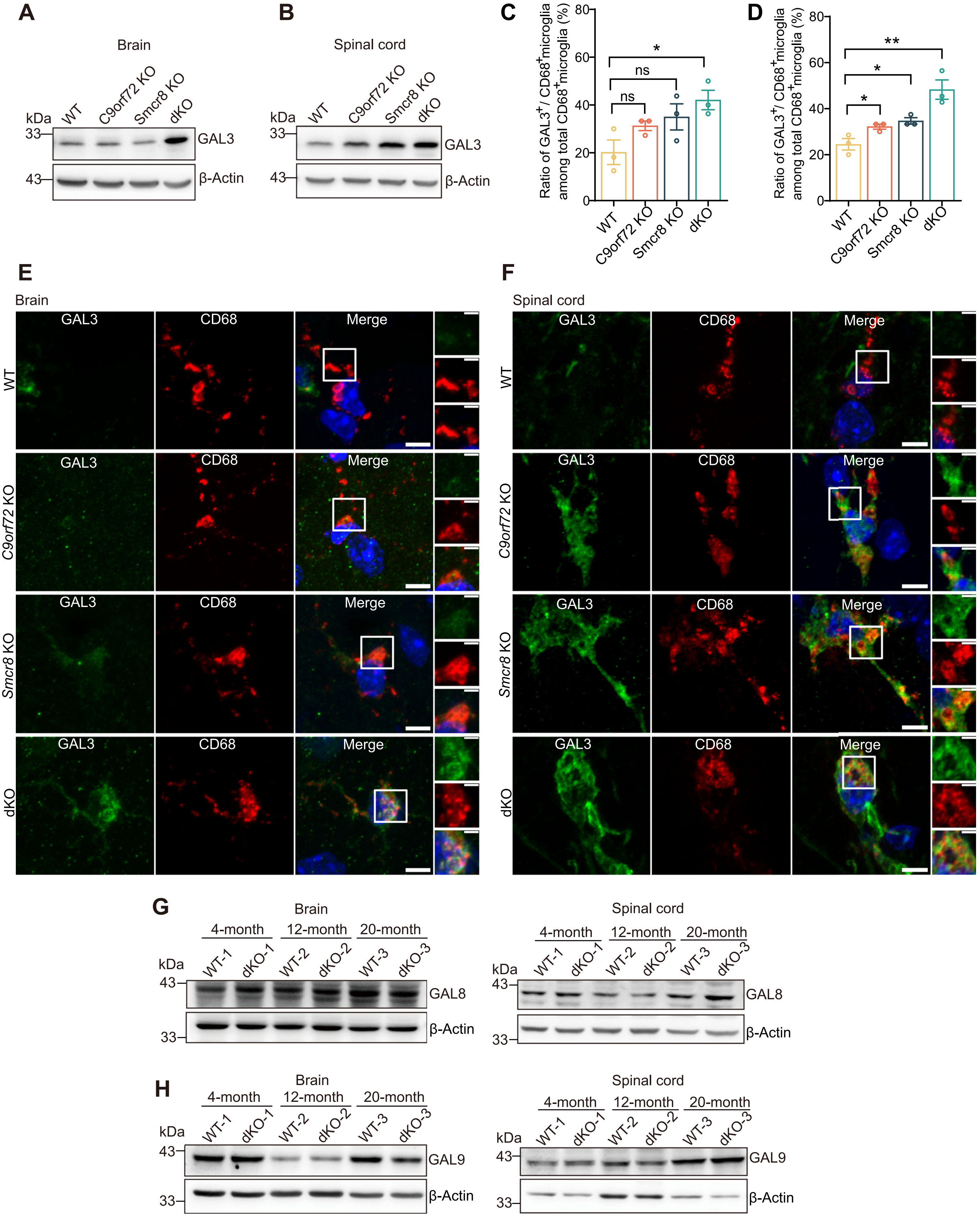
A
Brain
kDa
WT
C9orf72 KO
Smcr8 KO
dKO
33
GAL3
43
β-Actin
B
Spinal cord
kDa
WT
C9orf72 KO
Smcr8 KO
dKO
33
GAL3
43
β-Actin
C
Ratio of GAL3+/ CD68+microglia among total CD68+microglia (%)
80
60
40
20
0
ns
ns
*
WT
C9orf72 KO
Smcr8 KO
dKO
D
Ratio of GAL3+/ CD68+microglia among total CD68+microglia (%)
80
60
40
20
0
*
*
**
WT
C9orf72 KO
Smcr8 KO
dKO
E
Brain
GAL3
CD68
Merge
WT
C9orf72 KO
Smcr8 KO
dKO
F
Spinal cord
GAL3
CD68
Merge
WT
C9orf72 KO
Smcr8 KO
dKO
G
Brain
4-month
12-month
20-month
kDa
WT-1
dKO-1
WT-2
dKO-2
WT-3
dKO-3
43
GAL8
33
β-Actin
Spinal cord
4-month
12-month
20-month
kDa
WT-1
dKO-1
WT-2
dKO-2
WT-3
dKO-3
43
GAL8
33
β-Actin
H
Brain
4-month
12-month
20-month
kDa
WT-1
dKO-1
WT-2
dKO-2
WT-3
dKO-3
43
GAL9
33
β-Actin
Spinal cord
4-month
12-month
20-month
kDa
WT-1
dKO-1
WT-2
dKO-2
WT-3
dKO-3
43
GAL9
33
β-Actin

© The Author(s)

Figure EV2. GAL3 accumulation predominantly occurs in *C9orf72/Smcr8* double knockout mice.

(**A**, **B**) Immunoblotting analysis of GAL3 protein levels in brain (**A**) and spinal cord (**B**) tissue from 12-month-old WT, *C9orf72* KO, *Smcr8* KO, and dKO mice. β-Actin serves as loading control. (**C–F**) Representative immunofluorescence images of GAL3 (green), CD68 (red), and DAPI (blue) in brain cortex (**E**) and spinal cord (**F**) sections from 12-month-old WT, *C9orf72* KO, *Smcr8* KO, and dKO mice. Boxed areas are enlarged on the right showing individual channels and merged images. Scale bars, 5 μm (main), 2 μm (insets). Quantification of GAL3-positive puncta in CD68-positive microglia in brain cortex (**C**) and spinal cord (**D**) ($n = 3$ mice per genotype; 3–5 fields from 3 sections per mouse analyzed). Brain: WT vs *C9* KO: $P = 0.1158$, WT vs *S8* KO: $P = 0.1196$, WT vs dKO: $P = 0.0291$; Spinal cord: WT vs *C9* KO: $P = 0.0475$, WT vs *S8* KO: $P = 0.0231$, WT vs dKO: $P = 0.0082$. (**G**, **H**) Age-dependent expression of GAL8 (**G**) and GAL9 (**H**) in brain and spinal cord tissue from WT and dKO mice at 4, 12, and 20 months of age. β-Actin serves as loading control. Data information: In (**C**, **D**), data are presented as mean ± SEM from three independent experiments. Statistical significance was assessed by unpaired two-tailed Student's *t* test. *$P < 0.05$, **$P < 0.01$; ns, not significant. Source data are available online for this figure.

 © The Author(s)

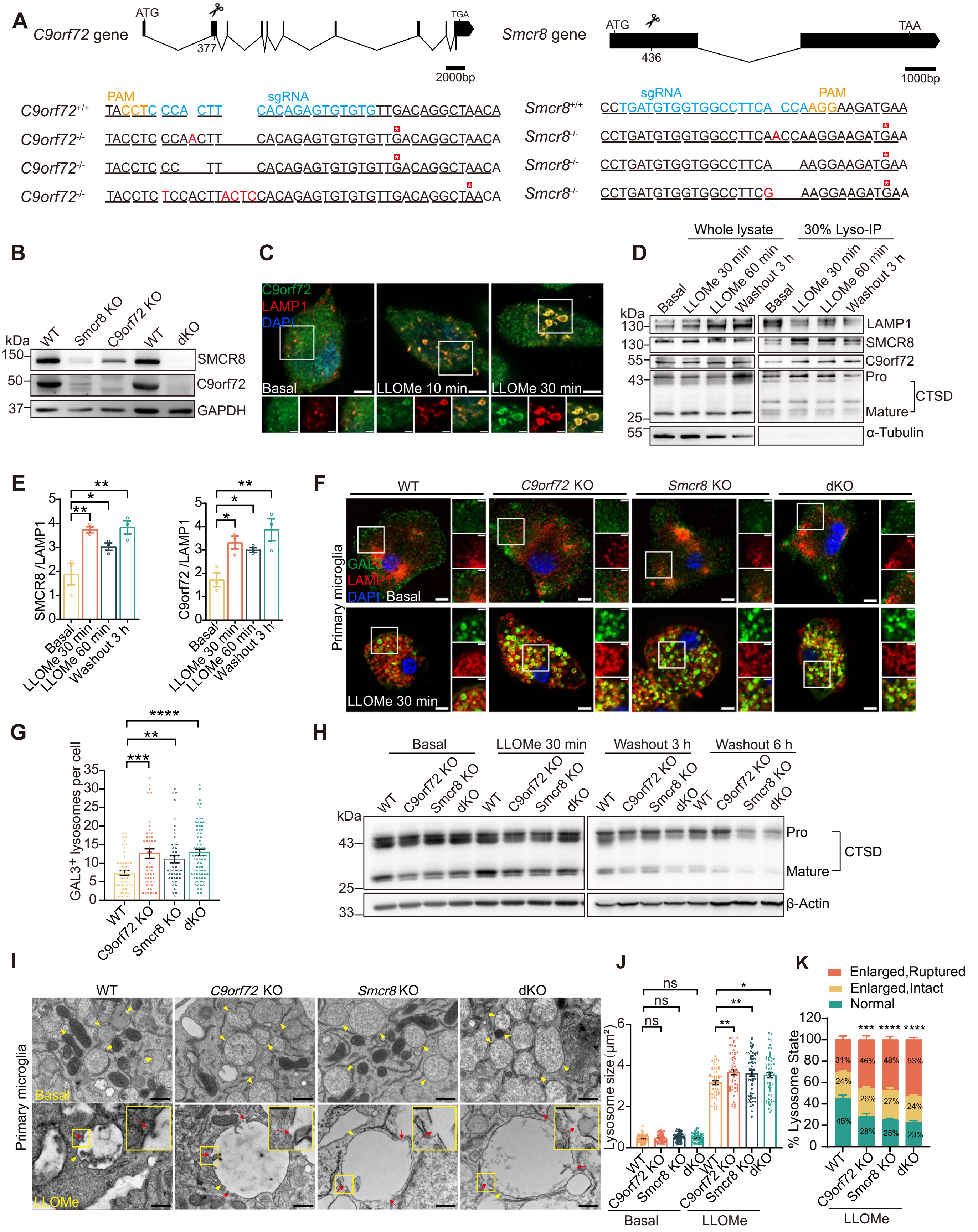
A
C9orf72 gene
ATG
377
TGA
2000bp
Smcr8 gene
ATG
436
TAA
1000bp
PAM
sgRNA
C9orf72+/+ TACCTC CCA CTT CACAGAGTGTGTGTTGACAGGCTAACA
C9orf72-/- TACCTC CCAACTT CACAGAGTGTGTGTTGACAGGCTAACA
C9orf72-/- TACCTC CC TT CACAGAGTGTGTGTTGACAGGCTAACA
C9orf72-/- TACCTC TCCACTTACTCCACAGAGTGTGTGTTGACAGGCTAACA
Smcr8+/+ CCTGATGTGGTGGCCTTCA CCAAGGAAGATGAA
Smcr8-/- CCTGATGTGGTGGCCTTCAACCAAGGAAGATGAA
Smcr8-/- CCTGATGTGGTGGCCTTCA AAGGAAGATGAA
Smcr8-/- CCTGATGTGGTGGCCTTCG AAGGAAGATGAA
B
kDa
WT
Smcr8 KO
C9orf72 KO
WT
dKO
150
50
37
SMCR8
C9orf72
GAPDH
C
C9orf72
LAMP1
DAPI
Basal
LLOMe 10 min
LLOMe 30 min
D
Whole lysate
30% Lyso-IP
kDa
Basal
LLOMe 30 min
LLOMe 60 min
Washout 3 h
130
130
55
43
25
55
LAMP1
SMCR8
C9orf72
Pro
CTSD
Mature
α-Tubulin
E
SMCR8 /LAMP1
C9orf72 /LAMP1
Basal
LLOMe 30 min
LLOMe 60 min
Washout 3 h
F
WT
C9orf72 KO
Smcr8 KO
dKO
Primary microglia
GAL3
LAMP1
DAPI
Basal
LLOMe 30 min
G
GAL3+ lysosomes per cell
WT
C9orf72 KO
Smcr8 KO
dKO
H
Basal
LLOMe 30 min
Washout 3 h
Washout 6 h
kDa
WT
C9orf72 KO
Smcr8 KO
dKO
43
25
33
Pro
CTSD
Mature
β-Actin
I
WT
C9orf72 KO
Smcr8 KO
dKO
Primary microglia
Basal
LLOMe
J
Lysosome size (μm²)
ns
Basal
LLOMe
K
Enlarged,Ruptured
Enlarged,Intact
Normal
% Lysosome State
LLOMe

Figure EV3. C9orf72 and SMCR8 are critical for maintaining lysosomal membrane integrity and repair.

(**A**) Schematic of *C9orf72* and *Smcr8* gene structures showing CRISPR/Cas9 targeting strategy. Representative sequencing results from knockout clones showing insertions/deletions at target sites leading to frameshift mutations and premature stop codons. Blue indicates sgRNA target sites, orange indicates PAM sequences, and asterisks mark stop codons. Scale bars, 2000 bp (*C9orf72*), 1000 bp (*Smcr8*). (**B**) Immunoblotting analysis confirming knockout efficiency in generated BV2 cell lines. SMCR8, C9orf72, and GAPDH protein levels were assessed in WT, *Smcr8* KO, *C9orf72* KO, and dKO cells. GAPDH serves as loading control. (**C**) Representative immunofluorescence images of endogenous C9orf72 (green) and LAMP1 (red) in microglia under basal conditions and after LLOMe treatment (1 mM) for 10 and 30 min. Boxed areas are enlarged below showing colocalization. Scale bars, 5 µm (main), 2 µm (insets). (**D**, **E**) LysoIP analysis showing association of endogenous C9orf72 and SMCR8 with lysosomes under basal conditions, after LLOMe treatment (1 mM, 30 and 60 min), and following 3 h washout (**D**). Input and LAMP1-IP fractions were analyzed by immunoblotting. Quantification of SMCR8/LAMP1 and C9orf72/LAMP1 ratios in IP fractions is shown in (**E**) ($n = 3$ independent experiments). SMCR8: LLOMe 30 min vs Basal, $P = 0.0042$; LLOMe 60 min vs Basal, $P = 0.05$; Washout 3 h vs Basal, $P = 0.0031$; C9orf72: LLOMe 30 min vs Basal, $P = 0.0174$; LLOMe 60 min vs Basal, $P = 0.0491$; Washout 3 h vs Basal, $P = 0.0033$). (**F**, **G**) Representative immunofluorescence images of GAL3 (green), LAMP1 (red), and DAPI (blue) in WT, *C9orf72* KO, *Smcr8* KO, and dKO primary microglia under basal conditions and after LLOMe treatment (0.5 mM, 30 min) (**F**). Boxed areas are enlarged on the right. Scale bar, 5 µm. Quantification of GAL3-positive lysosomes per cell is shown in (**G**). Each dot represents an individual cell ($n = 51$–78 cells per genotype from three independent experiments). WT vs *C9* KO, $P = 0.0005$; WT vs *S8* KO, $P = 0.0019$; WT vs dKO, $P < 0.0001$. (**H**) Immunoblotting analysis of cathepsin D (CTSD) processing in WT, *C9orf72* KO, *Smcr8* KO, and dKO BV2 cells under basal conditions, after LLOMe treatment (1 mM, 30 min), and during washout periods (3 and 6 h). Pro-CTSD and mature CTSD are indicated. β-Actin serves as loading control. (**I–K**) TEM images of primary microglia from WT, *C9orf72* KO, *Smcr8* KO, and dKO mice under basal conditions (upper row) and after LLOMe treatment (1 mM, 30 min; lower row) (**I**). Yellow arrowheads indicate lysosomes. Red arrowheads indicate sites of lysosomal membrane rupture (membrane discontinuity). Yellow boxed areas showing ruptured lysosomes are enlarged in the upper right corner of each panel. Scale bars, 500 nm (main), 200 nm (insets). Quantification of lysosome area is shown in (**J**). Each dot represents a single lysosome ($n = 58$–64 lysosomes per genotype from three independent experiments). *P* values - LLOMe: WT vs *C9* KO, $P = 0.0015$; WT vs *S8* KO, $P = 0.0075$; WT vs dKO, $P = 0.0272$. Percentage distribution of lysosome states following LLOMe treatment is shown in (**K**). Normal (green, area ≤3 µm²); enlarged with intact membrane (yellow, area >3 µm²); enlarged with ruptured membrane (orange, area >3 µm² with membrane discontinuity) ($n = 21$–23 cells per genotype from three independent experiments). WT vs *C9* KO, $P = 0.0002$; WT vs *S8* KO, $P < 0.0001$; WT vs dKO, $P < 0.0001$. Data information: In (**E**, **G**, **J**, **K**), data are presented as mean ± SEM from three independent experiments. Statistical significance was assessed by one-way ANOVA with Tukey's post-hoc test (**E**) or unpaired two-tailed Student's *t* test (**G**, **J**, **K**). *$P < 0.05$, **$P < 0.01$, ***$P < 0.001$, ****$P < 0.0001$; ns, not significant. Source data are available online for this figure.

 © The Author(s)

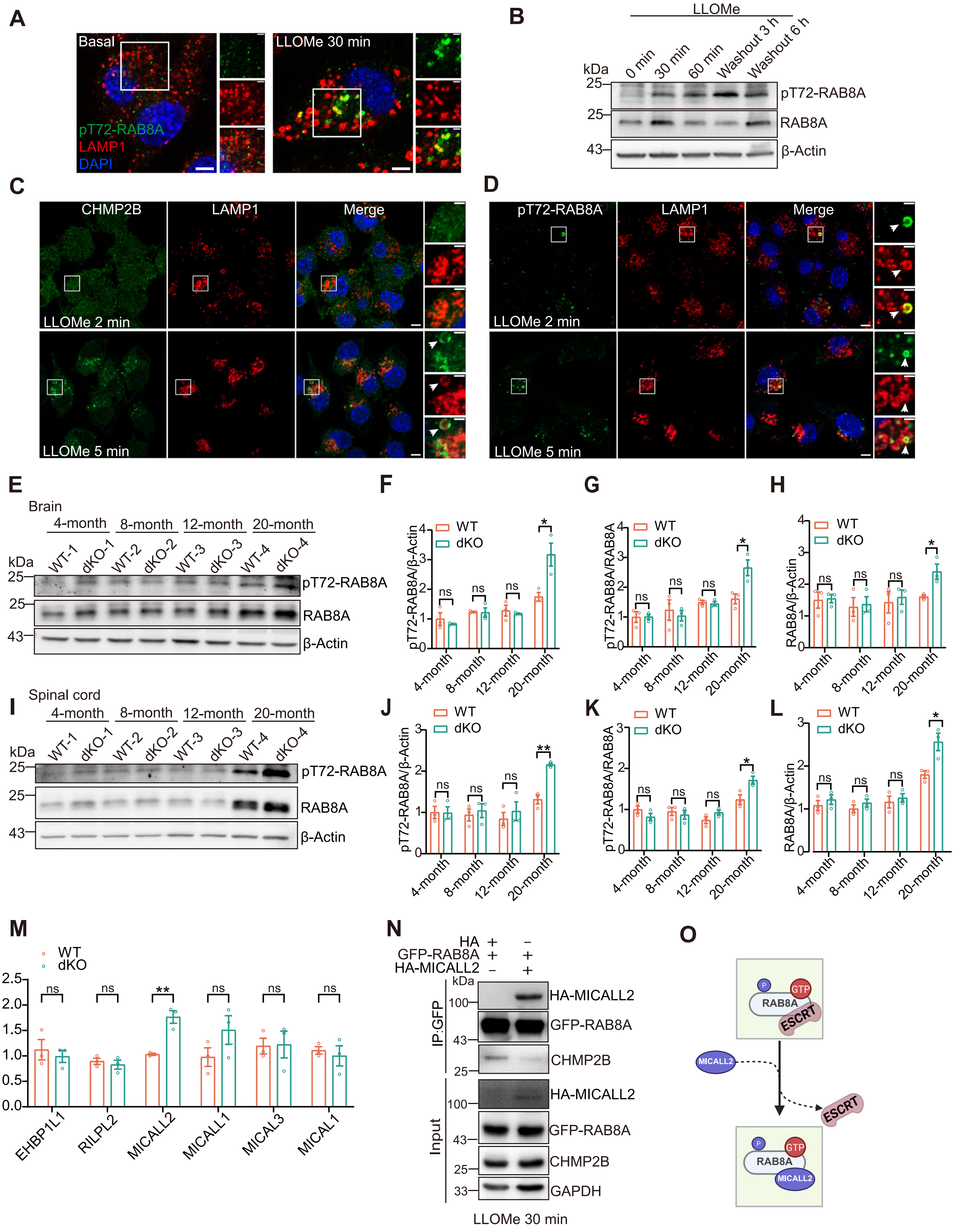
A
Basal
LLOMe 30 min
pT72-RAB8A
LAMP1
DAPI
B
LLOMe
0 min
30 min
60 min
Washout 3 h
Washout 6 h
kDa
pT72-RAB8A
RAB8A
β-Actin
C
CHMP2B
LAMP1
Merge
LLOMe 2 min
LLOMe 5 min
D
pT72-RAB8A
LAMP1
Merge
LLOMe 2 min
LLOMe 5 min
E
Brain
4-month
8-month
12-month
20-month
WT-1
dKO-1
WT-2
dKO-2
WT-3
dKO-3
WT-4
dKO-4
pT72-RAB8A
RAB8A
β-Actin
F
pT72-RAB8A/β-Actin
G
pT72-RAB8A/RAB8A
H
RAB8A/β-Actin
WT
dKO
ns
I
Spinal cord
J
K
L
M
Fold Change
EHBP1L1
RILPL2
MICALL2
MICALL1
MICAL3
MICAL1
N
HA
GFP-RAB8A
HA-MICALL2
IP:GFP
Input
HA-MICALL2
GFP-RAB8A
CHMP2B
GAPDH
LLOMe 30 min
O
P
GTP
RAB8A
ESCRT
MICALL2

Figure EV4. pT72-RAB8A accumulation in C9orf72/SMCR8-deficient tissues and cells.

(**A**) Representative immunofluorescence images of pT72-RAB8A (green), LAMP1 (red), and DAPI (blue) in BV2 cells under basal conditions and after LLOMe treatment (1 mM, 30 min). Boxed areas are enlarged on the right. Scale bars, 5 μm (main), 2 μm (insets). (**B**) Time-course immunoblotting analysis of pT72-RAB8A dynamics in BV2 cells following LLOMe treatment and washout. Cell lysates were probed with antibodies against pT72-RAB8A (top), total RAB8A (middle), and β-actin (loading control). (**C**, **D**) Representative immunofluorescence images showing temporal recruitment of CHMP2B (**C**) and pT72-RAB8A (**D**) (green) to lysosomes (LAMP1, red) at 2 and 5 min after LLOMe treatment (1 mM). DAPI (blue) marks nuclei. White arrows indicate colocalization of CHMP2B or pT72-RAB8A with LAMP1-positive lysosomes. Boxed areas are enlarged on the right showing individual channels and merged images. Scale bars, 5 μm (main), 2 μm (insets). (**E–H**) Age-dependent expression of pT72-RAB8A and total RAB8A in brain tissue from WT and dKO mice at 4, 8, 12, and 20 months of age (**E**). β-Actin serves as loading control. Quantification of pT72-RAB8A normalized to β-actin (**F**), pT72-RAB8A normalized to total RAB8A (**G**), and total RAB8A normalized to β-actin (**H**) ($n = 3$ mice per group). 20-month: pT72-RAB8A/β-actin, $P = 0.0256$; pT72-RAB8A/RAB8A, $P = 0.0281$; RAB8A/β-actin, $P = 0.0299$ (**I–L**) Age-dependent expression of pT72-RAB8A and total RAB8A in spinal cord tissue from WT and dKO mice at 4, 8, 12, and 20 months of age (**I**). β-Actin serves as loading control. Quantification of pT72-RAB8A normalized to β-actin (**J**), pT72-RAB8A normalized to total RAB8A (**K**), and total RAB8A normalized to β-actin (**L**) ($n = 3$ mice per group). 20-month: pT72-RAB8A/β-actin, $P = 0.0018$; pT72-RAB8A/RAB8A, $P = 0.0347$; RAB8A/β-actin, $P = 0.0257$). (**M**) Quantification of RAB8A effector protein binding fold-change (dKO/WT) from mass spectrometry analysis of RAB8A immunoprecipitates. WT and dKO BV2 cells were treated with LLOMe (1 mM, 30 min) prior to lysis and immunoprecipitation ($n = 3$ biological replicates; MICALL2: $P = 0.0041$, all other effectors: ns). (**N**) Co-IP analysis of GFP-RAB8A with HA-MICALL2 in HEK293T cells after LLOMe treatment (1 mM, 30 min). Input and IP fractions were analyzed by immunoblotting for HA-MICALL2, GFP-RAB8A, CHMP2B, and GAPDH. (**O**) Schematic model illustrating competitive binding of ESCRT machinery and MICALL2 to RAB8A upon lysosomal damage. Data information: In (**F–H**, **J–L**, **M**), data are presented as mean ± SEM from three independent experiments. Statistical significance was assessed unpaired two-tailed Student's *t* test. $^{*}P < 0.05$, $^{**}P < 0.01$, $^{****}P < 0.0001$; ns, not significant. Source data are available online for this figure.

 © The Author(s)

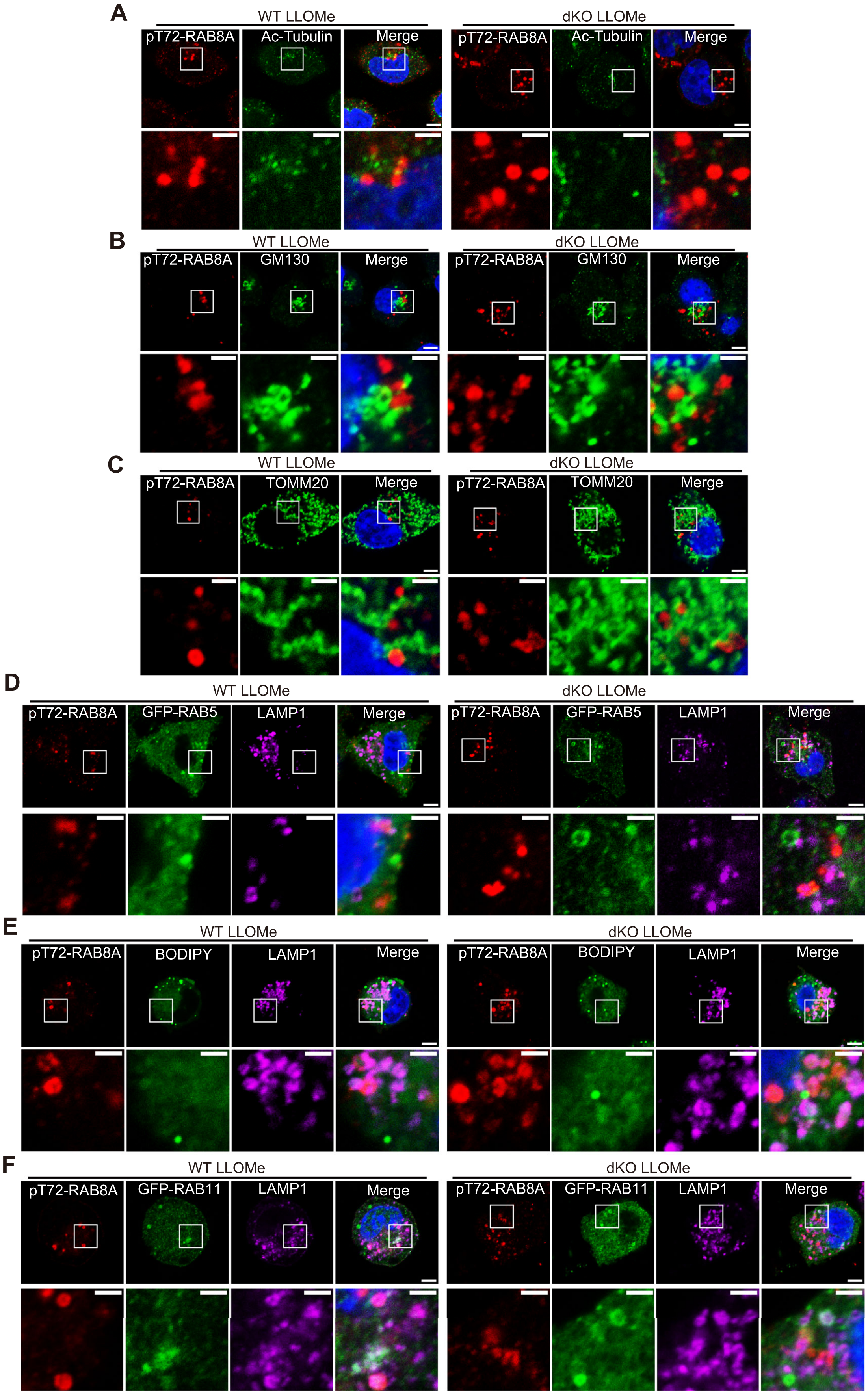
A
WT LLOMe
dKO LLOMe
pT72-RAB8A
Ac-Tubulin
Merge
B
GM130
C
TOMM20
D
GFP-RAB5
LAMP1
E
BODIPY
F
GFP-RAB11

Figure EV5. pT72-RAB8A does not localize to primary cilia, Golgi, mitochondria, or lipid droplets following lysosomal damage.

(**A**) Representative immunofluorescence images of pT72-RAB8A (red) and acetylated α-tubulin (Ac-tubulin, green; primary cilia marker) in WT and dKO BV2 cells after LLOMe treatment (1 mM, 30 min). DAPI (blue) marks nuclei. Boxed areas are enlarged below showing individual channels and merged images. Scale bar, 5 µm. (**B**) Representative immunofluorescence images of pT72-RAB8A (red) and GM130 (green; Golgi marker) in WT and dKO BV2 cells after LLOMe treatment (1 mM, 30 min). DAPI (blue) marks nuclei. Boxed areas are enlarged below. Scale bar, 5 µm. (**C**) Representative immunofluorescence images of pT72-RAB8A (red) and TOMM20 (green; mitochondrial marker) in WT and dKO BV2 cells after LLOMe treatment (1 mM, 30 min). DAPI (blue) marks nuclei. Boxed areas are enlarged below. Scale bar, 5 µm. (**D**) Representative immunofluorescence images of pT72-RAB8A (red), GFP-RAB5 (green; early endosome marker), and LAMP1 (magenta; lysosome marker) in WT and dKO BV2 cells after LLOMe treatment (1 mM, 30 min). DAPI (blue) marks nuclei. Boxed areas are enlarged below. Scale bar, 5 µm. (**E**) Representative immunofluorescence images of pT72-RAB8A (red), BODIPY (green; lipid droplet marker), and LAMP1 (magenta) in WT and dKO BV2 cells after LLOMe treatment (1 mM, 30 min). DAPI (blue) marks nuclei. Boxed areas are enlarged below. Scale bar, 5 µm. (**F**) Representative immunofluorescence images of pT72-RAB8A (red), GFP-RAB11 (green; recycling endosome marker), and LAMP1 (magenta) in WT and dKO BV2 cells after LLOMe treatment (1 mM, 30 min). DAPI (blue) marks nuclei. Boxed areas are enlarged below. Scale bar, 5 µm. Data information: Images are representative of three independent experiments. Source data are available online for this figure.

 © The Author(s)
