# Supplementary material for: The C9orf72/SMCR8 complex maintains microglial homeostasis via RAB8A-ESCRT-mediated lysosomal repair
Source: EMBO J. 2026 May 29;45(13):4531–68. doi: 10.1038/s44318-026-00817-w (PMC13324726; doi:10.1038/s44318-026-00817-w)
Supplement: Supplementary file 9 — Source data Fig. 8 [file 8F.pdf › 8F/8F.pdf]

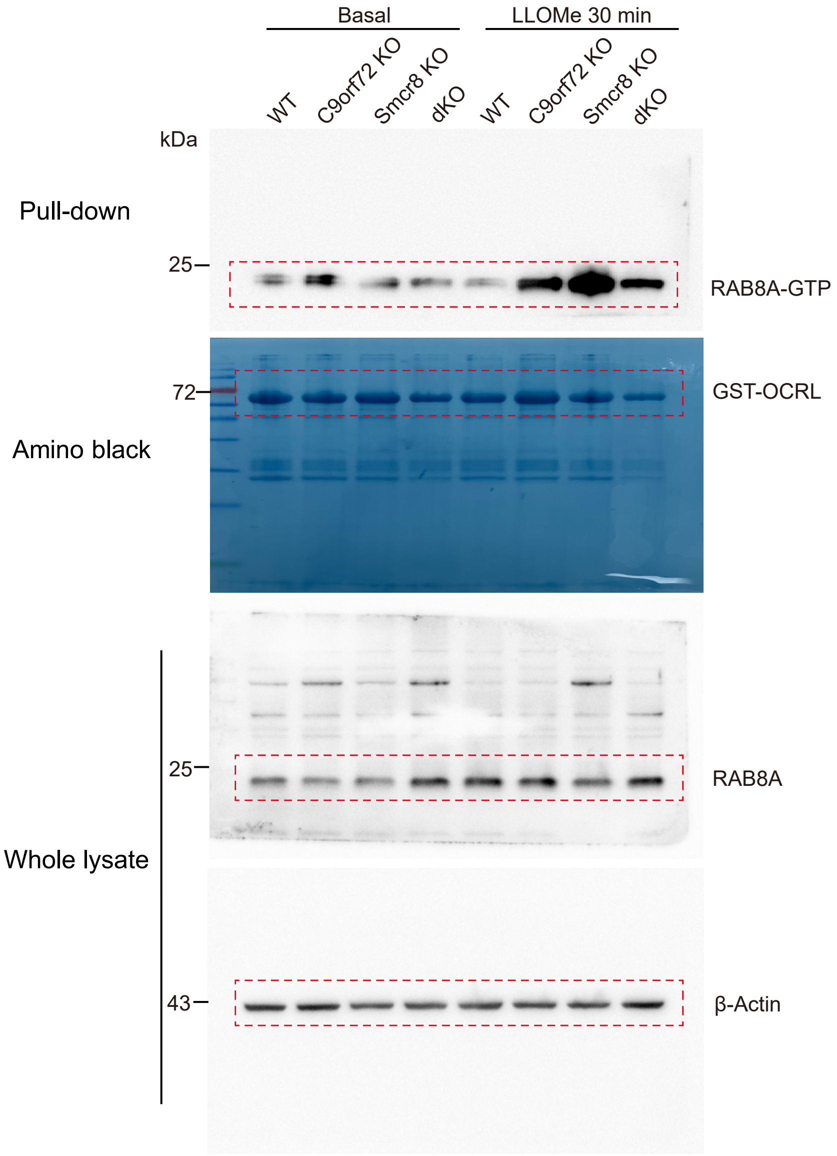

Basal
LLOMe 30 min
WT
C9orf72 KO
Smcr8 KO
dKO
WT
C9orf72 KO
Smcr8 KO
dKO
kDa
Pull-down
25
RAB8A-GTP
72
GST-OCRL
Amino black
25
RAB8A
Whole lysate
43
β-Actin
