# Supplementary material for: The C9orf72/SMCR8 complex maintains microglial homeostasis via RAB8A-ESCRT-mediated lysosomal repair
Source: EMBO J. 2026 May 29;45(13):4531–68. doi: 10.1038/s44318-026-00817-w (PMC13324726; doi:10.1038/s44318-026-00817-w)

Brain

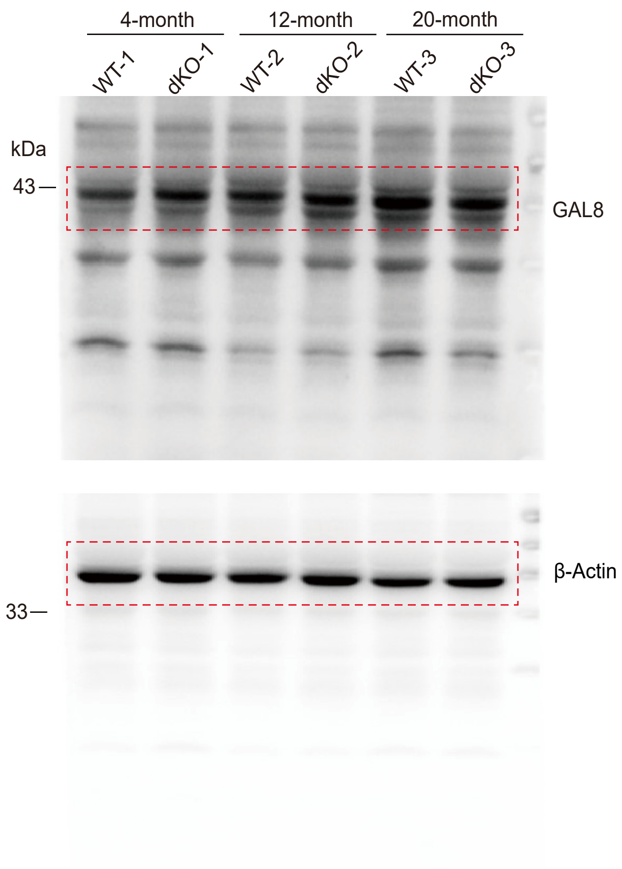

Spinal cord

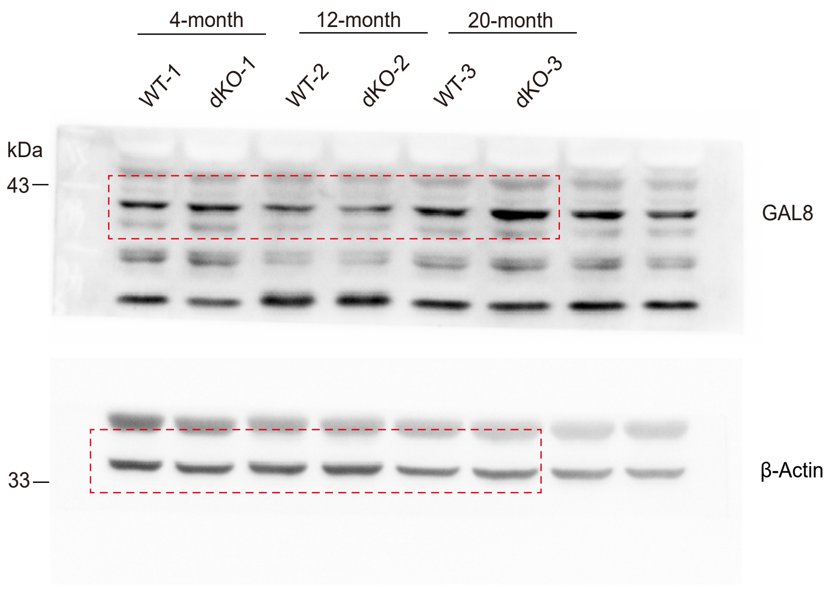

Supplement: Supplementary file 11 — Figure EV2 Source Data [file EV2G.pdf › EV2G/EV2G.pdf]
