# Supplementary material for: The C9orf72/SMCR8 complex maintains microglial homeostasis via RAB8A-ESCRT-mediated lysosomal repair
Source: EMBO J. 2026 May 29;45(13):4531–68. doi: 10.1038/s44318-026-00817-w (PMC13324726; doi:10.1038/s44318-026-00817-w)
Supplement: Supplementary file 2 — Peer Review File [file 44318_2026_817_MOESM2_ESM.pdf]

THE EMBO JOURNAL

The C9orf72/SMCR8 complex maintains microglial homeostasis via RAB8A ESCRT-mediated lysosomal repair

Shan Li, Shidong Xu, Feng Li, Qirui Zhao, Penghui Zhang, Qinghua Guan, Xiangxiang Sun, Jundong Bi, Hu Xiao, Yiyuli Tang, Cheng Peng, Qingfeng Chen, Yonghua Wang, and Mei Yang

Corresponding author(s): Mei Yang (meiyang@ynu.edu.cn) , Yonghua Wang (yonghuaw8002@ynu.edu.cn)

Review Timeline:

Submission Date:	21st Aug 25
Editorial Decision:	29th Sep 25
Revision Received:	5th Mar 26
Editorial Decision:	20th Apr 26
Revision Received:	1st May 26
Accepted:	8th May 26

Editor: Ieva Gailite

Transaction Report:

(Note: With the exception of the correction of typographical or spelling errors that could be a source of ambiguity, letters and reports are not edited. Depending on transfer agreements, referee reports obtained elsewhere may or may not be included in this compilation. Referee reports are anonymous unless the Referee chooses to sign their reports.)

Dear Dr. Yang,

Thank you for submitting your manuscript for consideration by the EMBO Journal. We have now received comments three reviewers, which are included below for your information.

As you will see, all reviewers are generally positive in their assessment and appreciate the contribution of the study to the research field. At the same time, they indicate several concerns that would need to be addressed before they can support publication here. From my side, I find these requests generally reasonable. Therefore, I would like to invite you to address these comments in a revised manuscript. I think that it would be useful to discuss the revision in more detail via email or phone/videoconferencing - please let me know which option you prefer.

We generally allow three months as standard revision time, which can be extended to six months in the case of major revisions. Should you foresee a problem in meeting this deadline, please let us know in advance to discuss an extension.

As a matter of policy, competing manuscripts published during this period will not negatively impact on our assessment of the conceptual advance presented by your study. However, please contact me as soon as possible upon publication of any related work to discuss the appropriate course of action.

When preparing your letter of response to the referees' comments, please bear in mind that this will form part of the Review Process File and will therefore be available online to the community. For more details on our Transparent Editorial Process, please visit our website: https://www.embopress.org/page/journal/14602075/authorguide#transparentprocess. Please also see the attached instructions for further guidelines on preparation of the revised manuscript.

Please feel free to contact me if have any further questions regarding the revision. Thank you for the opportunity to consider your work for publication, and I look forward to discussing your revision with you.

With best wishes,

Ieva Gailite

Ieva Gailite, PhD
Senior Scientific Editor
The EMBO Journal
Meyerhofstrasse 1
D-69117 Heidelberg
Tel: +4962218891309
i.gailite@embojournal.org

Instructions for preparing your revised manuscript:

Please make sure you upload a letter of response to the referees' comments together with the revised manuscript.

Please also check that the title and abstract of the manuscript are brief, yet explicit, even to non-specialists.

When assembling figures, please refer to our figure preparation guideline in order to ensure proper formatting and readability in print as well as on screen:
https://bit.ly/EMBOPressFigurePreparationGuideline
See also guidelines for figure legends: https://www.embopress.org/page/journal/14602075/authorguide#figureformat

At EMBO Press we ask authors to provide source data for the main manuscript figures. You will receive a separate email with instructions for providing source data with your revised manuscript, including how to upload and organize the files.

IMPORTANT: When you send the revision we will require
- a point-by-point response to the referees' comments, with a detailed description of the changes made (as a word file).
- a word file of the manuscript text.
- individual production quality figure files (one file per figure)
- a complete author checklist, which you can download from our author guidelines (https://www.embopress.org/page/journal/14602075/authorguide).

- Expanded View files (replacing Supplementary Information)
Please see out instructions to authors
https://www.embopress.org/page/journal/14602075/authorguide#expandedview
- a Reagents and Tools Table as part of the Methods section, which can be downloaded from our author guidelines (https://www.embopress.org/page/journal/14602075/authorguide#structuredmethods)

Please remember: Digital image enhancement is acceptable practice, as long as it accurately represents the original data and conforms to community standards. If a figure has been subjected to significant electronic manipulation, this must be noted in the figure legend or in the 'Materials and Methods' section. The editors reserve the right to request original versions of figures and the original images that were used to assemble the figure.

Further information is available in our Guide For Authors: https://www.embopress.org/page/journal/14602075/authorguide

We realize that it is difficult to revise to a specific deadline. In the interest of protecting the conceptual advance provided by the work, we recommend a revision within 3 months (28th Dec 2025). Please discuss the revision progress ahead of this time with the editor if you require more time to complete the revisions. Use the link below to submit your revision:

https://emboj.msubmit.net/cgi-bin/main.plex

Referee #1:

The topic of lysosome damage and repair is currently under intensive investigation as it is assumed that lysosomal membrane repair plays important roles in physiological contexts. Because current models are based almost exclusively on studies of cultured cells, there is a need for studies that investigate the importance of lysosomal membrane repair in vivo. In the present manuscript by Li and co-workers it is reported that the RAB8A GAP complex, C9orf72/SMCR8, maintains microglial homeostasis via RAB8A- and ESCRT-mediated lysosomal membrane repair. The authors have established mouse KO models of C9orf72 and SMCR8 and find that loss of these proteins causes age-dependent neuroinflammation and microgliosis with aged microglia showing accumulation of GAL3 on lysosomes, a sign of lysosome damage. For mechanistic studies the authors isolated primary microglia cells from wild-type and KO mice. They found that lack of C9orf72 or SMCR8 resulted in decreased recruitment of RAB8A and ESCRT proteins to lysosomes damaged with LLOMe. Curiously, the lack of C9orf72 or SMCR8 resulted in accumulation of GTP-bound and hyperphosphorylated on non-lysosomal vesicles upon lysosome damage. Finally, the authors found that the GAP activity of the C9orf72/SMCR8 complex is required for lysosome repair.

Since this manuscript provides in vivo evidence that lysosome repair is important for physiological functions, it is potentially of great interest. However, from the mechanistic side there are some issues that need to be addressed prior to eventual publication.

Major points:

1. The identity of the "non-lysosomal" vesicles where p-RAB8A accumulates in C9orf72/SMCR8-deficient cells should be clarified with relevant organelle markers as this is important for understanding the mechanism of C9orf72/SMCR8 in lysosomal membrane repair.

2. The timing of protein recruitment to damaged lysosomes needs to be considered. Previous live imaging experiments have shown that ESCRT recruitment occurs very rapidly after LLOMe-induced lysosome damage, peaking already at about 5 minutes. The data in Fig 6BC are consistent with this. If RAB8A is required for ESCRT recruitment, it should be recruited earlier than ESCRT.

3. The authors should include a summary figure or graphical abstract that summarizes their findings into a model for C9orf72/SMCR8 and RAB8A function in lysosome repair and microglial maintenance. The figure should explain why GTP-bound and hyperphosphorylated RAB8A accumulates on non-lysosomal vesicles in the absence of C9orf72/SMCR8 and why the GAP activity of C9orf72/SMCR8 is required for lysosomal membrane repair.

Minor points:

1. The findings in this manuscript should be more thoroughly discussed in relation to those of Herbst et al, EMBO J 2020. In that paper it was found that pS72-RAB8A mediates ESCRT recruitment to damaged lysosomes. Here, it is found that pS72-RAB8A accumulates on non-lysosomal vesicles in the absence of C9orf72/SMCR8 and that ESCRT recruitment to damaged lysosomes is impaired. If pS72-RAB8A recruits ESCRT, why is ESCRT not recruited to the non-lysosomal vesicles? Do other factors, only present on damaged lysosomes, participate in ESCRT recruitment together with pS72-RAB8A?

2. The C9orf72/SMCR8 complex has been found to be more active as a GAP for ARF1 than for RAB8A (Su et al.,Nat.Commun. 2020). Would ARF1 play any role in lysosomal membrane repair?

3. At the end of the Discussion the authors mention lipid transfer from the ER as an alternative mechanism of lysosome repair. They should include references for this.

Referee #2:

Hexanucleotide repeat expansion in the C9orf72 gene is a leading cause of ALS/FTD, and reduced expression of the C9ORF72 protein is one of the disease mechanisms. C9orf72 has been shown to form a GAP complex with SMCR8 to regulate RAB and ARF GTPases. Deficiency of this complex has also been shown to lead to immune dysregulation. In this manuscript, Li et al examined lysosomal abnormalities in microglia deficient in C9orf72, SMCR8, or both and demonstrated the deficiency of this C9orf72/SMCR8 complex leads to alteration in Rab8A dynamics and a defect in ESCRT mediated lysosomal repair. Overall, this manuscript is well written, the data is well presented, and supports the overall conclusion. However, there are several issues that should be addressed before publication.

Major comments:
1. Since C9orf72 and SMCR8 require each other for stability, have the authors detected neuronal loss and lysosomal damage in the brain of single knockout mice?
2. Are LRRK2 activities altered in C9orf72 or SMCR8 knockout mice?
3. Could the authors speculate what prevents Rab8A being recruited to the lysosome in C9orf72 KO despite having increased phosphorylation? Could the authors determine lysosomal recruitment of Rab8A GEF and GAP mutants (Q67L and T22N) under LLOME-treated conditions?

Detailed comments:
Fig. 1. Micorglial morphology and intensity do not seem to differ in WT and KO spinal cord, with the images shown in Fig. 1A (also 3H for brain).
Fig. 1B and 1C: Is TNF increased in the spinal cord and IL6 increased in the brain? What is used as control? It's labelled as WT in the figure but heterozygous was mentioned in the text.
Fig. 5F and G: The criteria for "defective lysosomes" need to be better defined. Some of the lysosome examples shown in basal conditions seem to be double-membraned autophagosome.
Fig. 6B and Fig. 7A-D Can lysosomal recruitment of CHMP2B and p-Rab8A be verified by western blot analysis, as shown in Fig. 6A?
Fig. 7A-D: In addition to p-Rab8A, could the authors also stain for total Rab8A?
Fig. 7E-M: Total Rab8A levels should be quantified and shown in the graphs too.
Fig. 8A and 8C: both Rab8 and pT72-RAB8A should be shown in both input and IP. And the experiment should be done in both basal and LLOME treated conditions.
Fig. 8F and G: basal conditions should be included as a control.
Fig. 9: Could the authors also look at pT72-RAB8A levels and recruitment to lysosome ?

Minor comments:
Introduction "Structural analyses have shown that this complex exhibits GTPase-activating protein (GAP) activity toward multiple RAB GTPases, including RAB8A": Arf GTPases should be mentioned too.

Referee #3:

Li and co-workers report on a novel mechanism by which the C9orf72/SMCR8 complex maintains homeostasis of microglia through a Rab8A-ESCRT mediated lysosomal repair. For this they generate a double C9/S8 KO mice wherein they observed age-dependent increased microgliosis and which resulted as well in increased pro-inflammatory responses, both in brain as well as in primary microglia. To dissect the underlying mechanism they combined dKO BV2 and primary microglia and modelled lysosomal damage through LLOME treatments (and washouts) followed by Gal3 stainings. In brief, they found in both cell models that single and dKO resulted in a defective recruitment of ESCRT components, known to be involved in early stages of lysosomal repair. Interestingly, they also demonstrate that in KO microglia, GTP-Rab8a accumulates and fails to be become recruited to damaged lysosomes, and which is parallelled by aberrantly phosphorylated Rab8a in a LRRK2 dependent manner. The data are as well further supported by co-IP and lyso-IP demonstrating the preferential binding to GTP-Rab8a. Overall the authors convincingly demonstrate a dual role for the C9/S8 complex in regulaing Rab8a during lysosomal repair, through its function as a GAP forRab8a but as well through regulating its phosphorylation status. Although several of these mechanistic insights are confirmed/supported by mouse brain data, translational relevance in ALS/FTD human models is lacking, diminishing a bit the overall enthusiasm. Nevertheless it remains a very relevant and systematic study, experimentally well scrutinized with

the necessary controls, and which provides a solid foundation for future validation. Overall the manuscript is suitable for publication in EMBO J, provided the authors address the following experimental points, which are in most cases minor but include as well additional experiments to support the intermittent conclusions with more quantified data.
Figure 2 shows snRNAseq data that show a 'significant' expansion of the microglial population in dKO mice: however, the graph doesn't show p-values and statistics should be done to claim significancy. Related, are the microglia the only cell population that shows alterations? A quantitative set for all cell types should be include to claim a more selective effect on microglial populations.
Figure 4B: besides dKO cells also single KO cells displayed increased Tnf and IL1b expression. Please correct.
Suppl Figure S3B: from this blot it is not clear that C9 and S8 are increased on isolated lysosomes. These experiments should be repeated and quantified. Related, why are the levels of Lamp1 decreased in the lyso-IP of LLOME-treated cells?
The EM data in Figure 5F-G (BV2) and Figure S3F-G (primary microglia) present defective lysosomes only in single KO cells. EM of dKO cells should be included. Also the authors state that in KO cells lysosomes have a 'decreased electron density': this is not evident from these pictures. For instance there is no real difference between WT and C9 KO BV2 cells and S8 KO have even a bit more dense lysosomes. Also the lysosomes in C9 KO primary microglia show more dense accumulations. The authors should provide more representative images or adjust the text to match the observations.
Related to Figure 6, the authors have investigated the recruitment of CHMP2B to lysosomes in the different genotypes. They conclude that more CHMP2B puncta are recruited to lysosomes in WT cells in contrast to the reduction in KO cells. However the data are only single images and based on these, one cannot conclude that: even in WT cells most CHMP2B spots are as well adjacent to Lamp1 positive spots. The authors should provide more convincing images and indicate as well co-localized spots.
Minor remark on Figure 8E,F: this is not an immunoprecipitation as explained in the text, but a GST pull-down. Please correct.
Finally, the authors use dKO cells to perform rescue experiments with WT C9/S8 or mutant variants. This set of data is limited to a wash-out experiment showing that Gal3-positive lysosomes numbers decreased in WT rescued but not mutant rescued cells. As for the other data sets, the authors should perform the full range of experiments, including the 10 and 30min LLOME followed by washout to get a more integrated idea on the rescue of dKO cells.
Minor comments: for dKO cells, the zoomed inset of the Gal3 staining shows an artifactul red dotted rastering. Please replace and correct this.

The authors must improve the discussion section. The current format merely reads as an extended abstract mainly re-iterating the major (and minor) findings, but hardly putting it in the context of the current literature. For instance, some more in depth discussion on what is known on lysosomal repair mechanisms in neurodegeneration-related models (human neurons, organoids etc) could provide more relevance (eg doi: 10.1038/s41556-025-01623-y using iPSC-derived neurons and tNeurons from familial AD patients). Or how this novel function of C9orf72 can be seen in the light of its other roles in modulating transport and organelle homeostasis? The authors could as well discuss in more depth to what extend other disease-related targets of C9orf72 and LRRK2 can be related to lysosomal repair (Rab10 is such an example, but the paragraph lacks references). The authors make an interesting link to lipid transfer between the ER and lysosomes through MCSs to facilitate lysosome repair: in this respect it is worth mentioning that a Parkinson-related gene, VPS13C, and which mediates lipid transfer between the ER and lysosomes was recently been implicated in lysosomal repair (doi: 10.1038/s41556-025-01653-6): hence the relevance of MCSs in maintaining organellar homeostasis could be highlighted (for instance doi: 10.1038/s41580-025-00864-x, doi: 10.3389/fnins.2022.900338).

Point-by-point responses to the comments raised by the reviewers

Referee #1:

The topic of lysosome damage and repair is currently under intensive investigation as it is assumed that lysosomal membrane repair plays important roles in physiological contexts. Because current models are based almost exclusively on studies of cultured cells, there is a need for studies that investigate the importance of lysosomal membrane repair in vivo. In the present manuscript by Li and co-workers it is reported that the RAB8A GAP complex, C9orf72/SMCR8, maintains microglial homeostasis via RAB8A- and ESCRT-mediated lysosomal membrane repair. The authors have established mouse KO models of C9orf72 and SMCR8 and find that loss of these proteins causes age-dependent neuroinflammation and microgliosis with aged microglia showing accumulation of GAL3 on lysosomes, a sign of lysosome damage. For mechanistic studies the authors isolated primary microglia cells from wild-type and KO mice. They found that lack of C9orf72 or SMCR8 resulted in decreased recruitment of RAB8A and ESCRT proteins to lysosomes damaged with LLOMe. Curiously, the lack of C9orf72 or SMCR8 resulted in accumulation of GTP-bound and hyperphosphorylated on non-lysosomal vesicles upon lysosome damage. Finally, the authors found that the GAP activity of the C9orf72/SMCR8 complex is required for lysosome repair.

Since this manuscript provides in vivo evidence that lysosome repair is important for physiological functions, it is potentially of great interest. However, from the mechanistic side there are some issues that need to be addressed prior to eventual publication.

Major points:

1. The identity of the "non-lysosomal" vesicles where p-RAB8A accumulates in C9orf72/SMCR8-deficient cells should be clarified with relevant organelle markers as this is important for understanding the mechanism of C9orf72/SMCR8 in lysosomal

membrane repair.

Response: We thank the reviewer for the positive and encouraging comments and insightful suggestions.

RAB8A has been shown to regulate centrosome cohesion, lysosomal membrane repair, lipid droplet fusion, autophagy and ciliogenesis (Dhekne *et al*, 2018; Eguchi *et al*, 2018; Herbst *et al*, 2020; Kania *et al*, 2023; Madero-Pérez *et al*, 2018; Mamais *et al*, 2021; Yu *et al*, 2018) Notably, in macrophages, Phosphorylated RAB8A (pT72-RAB8A) is recruited to damaged lysosomes following LLOMe treatment or bacterial infection, where it accumulates on damaged organelles to facilitate ESCRT-mediated repair (Eguchi *et al.*, 2018; Herbst *et al.*, 2020). In microglia, LRRK2 mutations linked to Parkinson's disease cause aberrant accumulation of phosphorylated RAB8A on lysosomes, leading to trafficking defects (Mamais *et al.*, 2021).

In our study, we observed a distinct phenotype from previous reports. C9orf72/SMCR8-deficient cells exhibit elevated pT72-RAB8A levels that fail to properly localize to damaged lysosomes in microglia (revised Fig. 7A-I and Fig. EV4E-L). Moreover, pT72-RAB8A in knockout cells displays a punctate vesicular pattern rather than the tubular morphology characteristic of the endocytic recycling compartment (ERC), excluding ERC localization as reported in other contexts (Hattula *et al*, 2006; Sharma *et al*, 2009).

To identify these non-lysosomal vesicles, we systematically examined colocalization with various organellar markers following LLOMe treatment (1 mM, 30 min). Systematic colocalization analysis revealed that hyperphosphorylated RAB8A specifically accumulates on RAB7-positive, LAMP1-negative vesicles (revised Fig. 7J-L), with no colocalization detected in early endosomes (RAB5), recycling endosomes (RAB11), mitochondria (TOMM20), Golgi (GM130), primary cilia (acetylated α-tubulin), or lipid droplets (BODIPY) (revised Fig. EV5).

Importantly, RAB7-positive, LAMP1-negative vesicles represent late endosomal intermediates in the endolysosomal maturation pathway. In neurons, dendritic cargos are degraded in $RAB7^{+}$/$LAMP1^{-}$ late endosomes (termed "early late endosomes") that require RAB7-dependent transport to somatic LAMP1-positive lysosomes for terminal

degradation(Yap *et al*, 2018). During Listeria monocytogenes infection of macrophages, bacteria exploit this maturation window by escaping from RAB7-positive, LAMP1-negative compartments before fusion with antimicrobial LAMP1-positive lysosomes (Henry *et al*, 2006). These findings establish $RAB7^{+}/LAMP1^{-}$ vesicles as critical trafficking intermediates in the endolysosomal pathway. Our finding that hyperphosphorylated RAB8A accumulates specifically on these intermediates in C9orf72/SMCR8-deficient cells suggests that loss of C9orf72/SMCR8 GAP activity traps phosphorylated RAB8A in a pre-lysosomal compartment, preventing its recruitment to sites of lysosomal membrane damage.

2. The timing of protein recruitment to damaged lysosomes needs to be considered. Previous live imaging experiments have shown that ESCRT recruitment occurs very rapidly after LLOMe-induced lysosome damage, peaking already at about 5 minutes. The data in Fig 6BC are consistent with this. If RAB8A is required for ESCRT recruitment, it should be recruited earlier than ESCRT.

Response: We thank the reviewer for this important point. We have examined the early kinetics of pT72-RAB8A and CHMP2B recruitment at 2 and 5 minutes following LLOMe treatment (revised Fig. EV4C-D). Due to species incompatibility (both antibodies are rabbit-derived), we performed parallel experiments staining cells separately with anti-pT72-RAB8A or anti-CHMP2B, together with anti-LAMP1. Our results show that pT72-RAB8A recruitment precedes CHMP2B: pT72-RAB8A puncta were already recruited to lysosomes at 2 minutes (white arrow, Fig. EV4D), while CHMP2B showed minimal recruitment at this time point (Fig. EV4C). By 5 minutes, both proteins showed lysosomal localization (white arrow, Fig. EV4 C and D). These data demonstrate that pT72-RAB8A is recruited prior to CHMP2B, supporting a model where RAB8A phosphorylation serves as an upstream signal for ESCRT recruitment.

3. The authors should include a summary figure or graphical abstract that summarizes their findings into a model for C9orf72/SMCR8 and RAB8A function in lysosome repair and microglial maintenance. The figure should explain why GTP-bound and

hyperphosphorylated RAB8A accumulates on non-lysosomal vesicles in the absence of C9orf72/SMCR8 and why the GAP activity of C9orf72/SMCR8 is required for lysosomal membrane repair.

Response: We thank the reviewer for this excellent suggestion. We have now included a comprehensive summary model in the revised manuscript (revised Fig. 9I) that illustrates the framework of C9orf72/SMCR8-dependent lysosomal repair and the consequences of its loss in microglia.

Our model (revised Fig. 9I) depicts two scenarios:

In WT microglia (left panel):

Upon lysosomal membrane damage, RAB8A undergoes GTP loading and is phosphorylated to generate pT72-RAB8A-GTP, which is recruited to damaged lysosomes where it facilitates ESCRT machinery recruitment for membrane repair. Following repair, the C9orf72/SMCR8 complex acts as a GAP to recognize and bind the phosphorylated GTP-RAB8A, promoting GTP hydrolysis back to its GDP-bound inactive state, completing the repair cycle.

In C9orf72/SMCR8-deficient microglia (right panel):

Loss of GAP activity causes RAB8A to accumulate on $RAB7^{+}$/$LAMP1^{-}$ late endosomes under basal conditions (Fig. R1, Figure for Reviewer1). Upon lysosomal damage, activated LRRK2 phosphorylates this mislocalized GTP-RAB8A pool *in situ* on $RAB7^{+}$/$LAMP1^{-}$ late endosomes—spatially sequestered away from damaged lysosomes. This aberrantly phosphorylated RAB8A binds to the effector MICALL2, which competitively reduces RAB8A association with ESCRT components. Trapped on late endosomes and unable to recruit ESCRT components, hyperphosphorylated RAB8A cannot traffic to damaged lysosomes, leading to impaired repair, persistent GAL3 accumulation, and chronic microglial activation.

Why GTP-bound and hyperphosphorylated RAB8A accumulates on non-lysosomal vesicles:

<u>Response:</u> To address this question, we examined RAB8A localization under basal and stress conditions. Under basal conditions, we did not detect elevated phospho-RAB8A levels by either Western blot (revised Fig. 7F) or immunofluorescence (revised Fig. 7A, D) in knockout cells. To visualize total RAB8A distribution, we transfected equal amounts of GFP-RAB8A into WT and dKO microglia, as multiple commercially

associated localization. Biochemical analysis further revealed a modest increase in total GTP-bound RAB8A in *C9orf72* single KO cells, though no obvious difference was observed in dKO cells relative to WT under basal conditions (revised Fig. 8E-F), suggesting that C9orf72/SMCR8 may not significantly regulate bulk RAB8A GTPase cycling during basal homeostasis, and that the basal accumulation on $RAB7^{+}/LAMP1^{-}$ vesicles represents a spatially restricted pool.

Upon LLOMe treatment, GTP-RAB8A levels dramatically increased in knockout cells (revised Fig. 8F) and became hyperphosphorylated by LRRK2 (revised Fig. 7F and 8G-J), accumulating on $RAB7^{+}/LAMP1^{-}$ late endosomes (revised Fig. 7J-L). Based on RAB8A's established role at recycling endosomes (Hattula *et al.*, 2006; Kanerva *et al*, 2013) and the observation that total GTP-RAB8A levels are comparable between WT and dKO basally (revised Fig. 8F), we propose that C9orf72/SMCR8 GAP activity may not be essential for bulk RAB8A cycling under homeostatic conditions. The majority of RAB8A GTPase cycling at recycling endosomes appears to be maintained by other compensatory GAP activities. However, the basal accumulation of GFP-RAB8A on $RAB7^{+}/LAMP1^{-}$ late endosomes in knockout cells (Fig.R1A-B) suggests that a minor subpopulation of GTP-RAB8A undergoes aberrant trafficking, converting to late endosomes in the absence of C9orf72/SMCR8. This low-level constitutive aberrant maturation is tolerated under basal conditions but becomes severely exacerbated upon lysosomal damage, when LRRK2 activation phosphorylates the accumulated GTP-RAB8A pool and amplifies sequestration on $RAB7^{+}/LAMP1^{-}$ late endosomes.

Why GAP activity is required for lysosomal membrane repair:

Response: Our data reveal that the C9orf72/SMCR8 complex functions as a damage-responsive GAP for RAB8A. Key observations include: (1) C9orf72 relocalizes to lysosomes upon damage (Fig. EV3C), (2) the complex shows increased lysosomal association after LLOMe (Fig. EV3D-E), (3) LLOMe enhances complex interaction with phosphorylated GTP-RAB8A (Fig. 8A, C), (4) knockout cells show baseline accumulation of RAB8A puncta on $RAB7^{+}/LAMP1^{-}$ vesicles (Fig. R1), and (5) upon damage, knockout cells accumulate excessive GTP-RAB8A (Fig. 8E-F), and (6) this accumulated GTP-RAB8A becomes hyperphosphorylated and sequestered on

RAB7+/LAMP1- late endosomes (Fig. 7J-K).

We propose the following model: Under basal conditions, C9orf72/SMCR8 maintains proper RAB8A vesicular distribution. Upon lysosomal damage, the complex is recruited to damaged lysosomes where it interacts with phosphorylated GTP-RAB8A recruited by damage signals. After repair, C9orf72/SMCR8 GAP activity hydrolyzes GTP to inactivate RAB8A and terminate the repair cycle. In knockout cells, loss of GAP activity causes baseline vesicular dysregulation and, upon damage, excessive GTP-RAB8A accumulation that becomes hyperphosphorylated but fails to reach lysosomes—instead being trapped on late endosomal intermediates. This prevents ESCRT recruitment and impairs repair. GAP-deficient mutants fail to rescue (revised Fig. 9), demonstrating that enzymatic activity, not mere protein presence, is essential. This reveals that C9orf72/SMCR8 functions not only in acute damage response but also in maintaining RAB8A trafficking homeostasis, explaining progressive lysosomal dysfunction in aged knockout mice.

Minor points:

1. The findings in this manuscript should be more thoroughly discussed in relation to those of Herbst et al, EMBO J 2020. In that paper it was found that pS72-RAB8A mediates ESCRT recruitment to damaged lysosomes. Here, it is found that pS72-RAB8A accumulates on non-lysosomal vesicles in the absence of C9orf72/SMCR8 and that ESCRT recruitment to damaged lysosomes is impaired. If pS72-RAB8A recruits ESCRT, why is ESCRT not recruited to the non-lysosomal vesicles? Do other factors, only present on damaged lysosomes, participate in ESCRT recruitment together with pS72-RAB8A?

Response: We thank the reviewer for this important question regarding the spatial specificity of ESCRT recruitment. Herbst et al. discovered that the p-RAB8A mutant (T72A) and GDP-locked RAB8A (T22N) fail to be recruited to damaged lysosomes upon LLOMe treatment, thereby preventing ESCRT recruitment, and treatment with the Ca^{2+} chelator BAPTA-AM inhibits the recruitment of LRRK2, Rab8A, and ESCRT

components to lysosomes(Herbst *et al.*, 2020). These findings indicate that pS72-RAB8A mediates ESCRT recruitment to damaged lysosomes. Consistent with this, in our C9orf72/SMCR8-deficient mutants, we observed reduced ESCRT recruitment to lysosomes (Fig. 6), attributable to the failure of p-RAB8A to localize to lysosomes (revised Fig.7 A-C). Instead, hyperphosphorylated RAB8A accumulates on $RAB7^+/LAMP1^-$ late endosomes (revised Fig. 7J-K), raising the reviewer's important question: if pT72-RAB8A recruits ESCRT, why does ESCRT not accumulate on these non-lysosomal $RAB7^+/LAMP1^-$ vesicles?

To address this question experimentally, we investigated whether RAB8A effector proteins competitively inhibit ESCRT binding on non-lysosomal compartments. We performed RAB8A immunoprecipitation from WT and dKO cell lysates following LLOMe treatment, followed by mass spectrometry analysis to identify interacting proteins. Among the detected effectors, MICALL2 showed significantly elevated association with RAB8A in knockout cells (revised Fig. EV4M). To test whether MICALL2 competitively inhibits ESCRT binding, we performed co-IP experiments and found that overexpression of MICALL2 significantly reduced the association between RAB8A and the ESCRT component CHMP2B (revised Fig. EV4N, O), indicating competitive binding between MICALL2 and ESCRT for RAB8A interaction. These data demonstrate that competitive effector binding contributes to impaired ESCRT recruitment in dKO cells. Additionally, Herbst *et al.* (2020) demonstrated that lysosomal calcium release is required for recruiting phosphorylated RAB8A and ESCRT to damaged lysosomes. Sequestration of pT72-RAB8A on $RAB7^+/LAMP1^-$ vesicles distant from damaged lysosomes may prevent it from receiving these calcium-dependent recruitment signals, further impairing ESCRT complex assembly and trafficking. Together, these mechanisms ensure that hyperphosphorylated RAB8A remains trapped on $RAB7^+/LAMP1^-$ compartments and cannot participate in lysosomal membrane repair.

2. The C9orf72/SMCR8 complex has been found to be more active as a GAP for ARF1 than for RAB8A (Su et al., Nat.Commun. 2020). Would ARF1 play any role in lysosomal membrane repair?

Response: We thank the reviewer for raising this important question. While Su et al. (2020) showed higher C9orf72/SMCR8 GAP activity toward ARF1 than RAB8A *in vitro*, we examined whether ARF1 participates in lysosomal repair in our cellular system. Immunofluorescence revealed that ARF1 localizes to the Golgi (GM130-positive) under both basal and LLOMe-treated conditions, with no relocalization to damaged lysosomes (Fig. R2 AB). Western blot showed no changes in ARF1 protein levels in WT or knockout cells (Fig. R2C). These data suggest that ARF1 does not directly participate in lysosomal membrane repair. This highlights that substrate specificity is determined by subcellular compartmentalization: while C9orf72/SMCR8 may have higher intrinsic GAP activity toward ARF1, the complex is recruited to damaged lysosomes (Fig. EV3C-E) where it encounters RAB8A, not ARF1. This context-dependent regulation allows the same GAP to control different GTPases in different cellular processes.

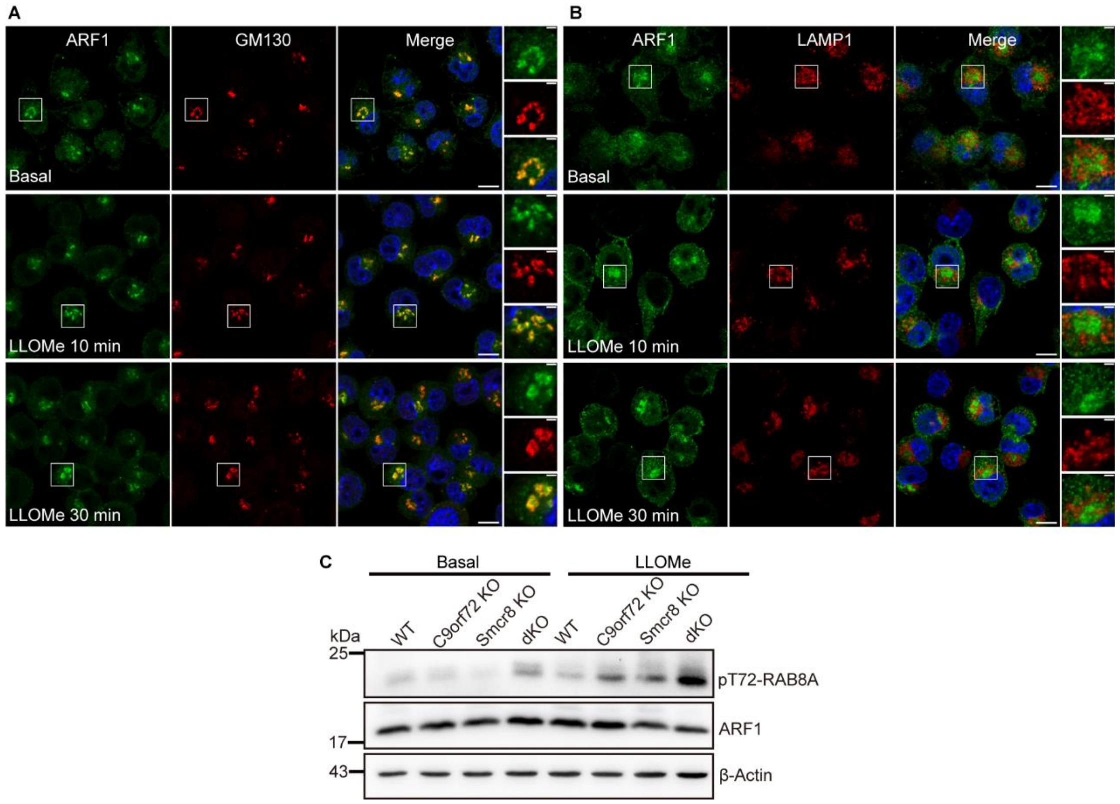

Figure R2. ARF1 does not participate in lysosomal membrane repair. (A, B) Immunofluorescence showing ARF1 (green) with GM130 (Golgi marker, red) (A) or LAMP1 (lysosome marker, red) (B) and DAPI (blue) in WT and dKO cells under basal and LLOMe conditions (1 mM, 10 and 30 min). Boxed areas enlarged on right. ARF1 remains at Golgi, no relocalization to lysosomes. Scale bar, 5 μm.(C) Western blot of ARF1 in WT and dKO cells (basal and LLOMe 30 min). pT72-RAB8A (positive control), β-Actin (loading control).

3. At the end of the Discussion the authors mention lipid transfer from the ER as an alternative mechanism of lysosome repair. They should include references for this.

Response: We thank the reviewer for this suggestion. We have now added the following references to support the statement regarding ER-mediated lipid transfer in lysosomal repair:

Radulovic, M. et al. Cholesterol transfer via endoplasmic reticulum contacts mediates lysosome damage repair. EMBO J. 41, e112677 (2022).

Tan, J. X. & Finkel, T. A phosphoinositide signalling pathway mediates rapid lysosomal repair. Nature 609, 815–821 (2022).

These references have been incorporated into the revised Discussion.

Referee #2:

Hexanucleotide repeat expansion in the C9orf72 gene is a leading cause of ALS/FTD, and reduced expression of the C9ORF72 protein is one of the disease mechanisms. C9orf72 has been shown to form a GAP complex with SMCR8 to regulate RAB and ARF GTPases. Deficiency of this complex has also been shown to lead to immune dysregulation. In this manuscript, Li et al examined lysosomal abnormalities in microglia deficient in C9orf72, SMCR8, or both and demonstrated the deficiency of this C9orf72/SMCR8 complex leads to alteration in Rab8A dynamics and a defect in ESCRT mediated lysosomal repair. Overall, this manuscript is well written, the data is well presented, and supports the overall conclusion. However, there are several issues that should be addressed before publication.

Major comments:

1. Since C9orf72 and SMCR8 require each other for stability, have the authors detected neuronal loss and lysosomal damage in the brain of single knockout mice?

Response: We sincerely thank the reviewer for the thoughtful comments and suggestions. To systematically assess whether neuronal loss and lysosomal damage occur in single knockout mice, we analyzed brain and spinal cord tissues from 12-month-old WT, *C9orf72* KO, *Smcr8* KO, and dKO mice (revised Fig. EV1K-N and Fig. EV2A-F). We examined general neurons using NeuN immunostaining in the hippocampus and motor neurons using ChAT staining in the spinal cord (revised Fig. EV1K-N). Double knockout mice showed significant neuronal loss: NeuN-positive neurons were significantly reduced in the hippocampus of dKO mice, while single knockout mice (*C9orf72* or *Smcr8* KO) showed no significant reduction (Fig. EV1K, M). ChAT-positive motor neurons in the spinal cord were significantly reduced in dKO mice, while single knockout mice showed a trend toward reduction that did not reach statistical significance (Fig. EV1L, N). We assessed lysosomal damage by examining GAL3 protein levels and GAL3-positive microglia. Western blot analysis revealed

elevated GAL3 protein levels in brain and spinal cord tissues (Fig. EV2A, B). In brain tissue, GAL3 elevation was observed predominantly in dKO mice, with minimal changes in single knockout mice (revised Fig. EV2A). In spinal cord tissue, both *C9orf72* KO and *Smcr8* KO mice showed modest GAL3 elevation; dKO mice displayed markedly higher GAL3 levels compared to both WT and single knockout mice (revised Fig. EV2B). These data support our conclusion that C9orf72 and SMCR8 function cooperatively in the same complex to maintain lysosomal homeostasis: partial complex function (via residual protein levels in single knockouts) provides protection (Fig. EV3B), whereas complete loss of the complex (in dKO) causes defective lysosomal repair, neuroinflammation, and neuronal loss.

2. Are LRRK2 activities altered in C9orf72 or SMCR8 knockout mice?

Response: We thank the reviewer for this important question. We observed increased pT72-RAB8A levels in knockout cells under both LLOMe-treated conditions and in aged knockout tissues (Fig.7C-I, 8G, EV4 E-L). The specificity of the pT72-RAB8A antibody has been validated by Herbst et al. (Herbst *et al.*, 2020), who demonstrated that it specifically recognizes LRRK2-phosphorylated RAB8A and that the signal is abolished by LRRK2 inhibitors. Since T72 is a known LRRK2 phosphorylation site on RAB8A(Steger *et al*, 2016; Waschbüsch *et al*, 2020) , and our *in vitro* experiments showed that addition of LRRK2 enhances RAB8A phosphorylation (Fig. 8J), the elevated pT72-RAB8A in knockout cells suggests potential involvement of LRRK2 kinase activity. To directly assess LRRK2 activity in knockout cells, we examined phosphorylation of LRRK2 at Ser935 (pS935-LRRK2), a phosphorylation site whose level correlates with LRRK2 kinase activity (Chiang *et al*, 2025; Dzamko *et al*, 2010; Kalogeropulou *et al*, 2022). Western blot analysis revealed that pS935-LRRK2 levels were elevated in dKO BV2 cells following LLOMe treatment compared to WT cells (Fig. R3), indicating enhanced LRRK2 kinase activity in knockout cells upon lysosomal damage.

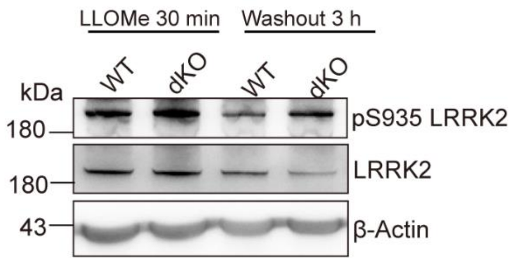

Figure R3. LRRK2 kinase activity is elevated in dKO BV2 cells following lysosomal damage. Western blot of pS935-LRRK2 (phosphorylation site correlated with kinase activity), total LRRK2, and β-actin in WT and dKO BV2 cells treated with LLOMe (1 mM, 30 min) followed by washout (3 h). Representative blot from n = 3 independent experiments.

3. Could the authors speculate what prevents Rab8A being recruited to the lysosome in C9orf72 KO despite having increased phosphorylation? Could the authors determine lysosomal recruitment of Rab8A GEF and GAP mutants (Q67L and T22N) under LLOME-treated conditions?

Response: We thank the reviewer for these insightful questions.

Question 1: What prevents pRAB8A from being recruited to lysosomes in knockout cells?

In our study, we observed that following LLOMe-induced lysosomal damage, pT72-RAB8A showed significantly reduced colocalization with LAMP1-positive damaged lysosomes in KO cells compared to WT (Fig. 7A-B). Instead, pT72-RAB8A aberrantly accumulated on $RAB7^+/LAMP1^-$ vesicles (Fig. 7J-L), spatially segregated from sites of ESCRT-mediated lysosomal membrane repair.

To understand the underlying mechanism, we examined RAB8A distribution under basal conditions. Since pRAB8A was undetectable basally by both Western blot and immunofluorescence (revised Fig. 7A, D, F), and commercially available RAB8A antibodies failed to provide specific signal in BV2 cells, we transfected equal amounts of GFP-RAB8A into WT and dKO microglia to visualize total RAB8A localization. Interestingly, GFP-RAB8A showed increased accumulation on $RAB7^+/LAMP1^-$ late endosomal vesicles in dKO cells under basal conditions (Fig. R1A-B), indicating that a

subpopulation of RAB8A aberrantly traffics to late endosomes even without lysosomal stress, potentially reflecting enrichment of its GTP-bound active form given the membrane-associated localization. This low-level constitutive mislocalization is tolerated under basal conditions but becomes severely exacerbated upon lysosomal damage. Upon LLOMe treatment, GTP-RAB8A levels dramatically increased in KO cells (revised Fig. 8F), and the accumulated pool on $RAB7^{+}/LAMP1^{-}$ late endosomes became hyperphosphorylated by activated LRRK2 (revised Fig. 7F, 8G-J). Consequently, pRAB8A remained sequestered on late endosomes rather than being recruited to damaged lysosomes. We propose that loss of C9orf72/SMCR8 GAP activity leads to a low-level constitutive accumulation of GTP-bound RAB8A on $RAB7^{+}/LAMP1^{-}$ late endosomes under basal conditions, which serves as a pre-formed mislocalized pool that is aberrantly phosphorylated by LRRK2 upon lysosomal damage.

Question 2: Lysosomal recruitment of RAB8A mutants (Q67L and T22N) under LLOMe treatment.

Lysosomal recruitment of RAB8A mutants (Q67L and T22N) has been reported by Herbst *et al.*(Herbst *et al.*, 2020) in macrophages, demonstrating that GTP-locked Q67L shows enhanced lysosomal localization while GDP-locked T22N and non-phosphorylatable T72A fail to recruit to damaged lysosomes upon LLOMe treatment. This Q67L mutant data reveals a critical distinction: while exogenously expressed GTP-locked Q67L is efficiently recruited to damaged lysosomes in WT cells, endogenous pT72-GTP-bound RAB8A that accumulates in *C9orf72/Smcr8* knockout cells fails to reach lysosomes. We propose this difference reflects the compartmental context of GTP loading: Q67L is constitutively GTP-loaded upon synthesis and available for de novo recruitment to damaged lysosomes. In contrast, in KO cells, endogenous RAB8A accumulates on $RAB7^{+}/LAMP1^{-}$ vesicles under basal conditions (Fig. R1) where it is stably membrane-anchored. Upon LLOMe treatment and LRRK2 activation, this pre-existing membrane-associated GTP-RAB8A cannot mobilize from its basal compartment and instead becomes phosphorylated *in situ*.

Detailed comments:

Fig. 1. Micorglial morphology and intensity do not seem to differ in WT and KO spinal cord, with the images shown in Fig. 1A (also 3H for brain).

Response: We thank the reviewer for this observation. The increased total IBA1 intensity in dKO tissues (Fig. 1C-D) is primarily due to increased microglial cell numbers (microgliosis). When microglial density is high, the imaging field captures processes from many cells, including those whose cell bodies lie outside the field, contributing to elevated IBA1 signal.

To comprehensively assess microglial morphology at the single-cell level, we performed three-dimensional surface reconstruction analysis using the Imaris Surface module (revised Fig. EV1G-J). dKO microglia showed significantly reduced branch number and total process length compared to WT, indicating decreased ramification and process retraction characteristic of activated microglia transitioning from a ramified "surveillant" state to an amoeboid “reactive” state, consistent with increased pro-inflammatory cytokine expression.

Fig. 1B and 1C: Is TNF increased in the spinal cord and IL6 increased in the brain? What is used as control? It's labelled as WT in the figure but heterozygous was mentioned in the text.

Response: We apologize for the error in the text. The control group used in Fig. 1 is WT, not heterozygous mice. We have corrected this mistake in the revised manuscript text. Regarding cytokine expression in different CNS regions, we have now included the additional requested data in revised Fig. 1:

Figure 1F (revised): *Il6* in brain tissue shows no significant difference between WT and knockout mice at 4 or 20 months of age.

Figure 1G (revised): *Tnfα* in spinal cord tissue is significantly upregulated in aged (20-month-old) knockout mice compared to WT.

Fig. 5F and G: The criteria for "defective lysosomes" need to be better defined. Some of the lysosome examples shown in basal conditions seem to be double-membraned

autophagosome.

Response: We thank the reviewer for this important point. We agree that the criteria for "defective lysosomes" needed more precise definition and better examples. We have revised Fig.5F-H to address this concern.

We now classify lysosomes into three distinct morphological categories based on ultrastructural features: (1) Normal lysosomes with intact single-limiting membrane and electron-dense content, area $\leqslant$0.6 μm^2 in BV2 cells; (2) Enlarged lysosomes with intact membranes, showing increased area (>0.6 μm^2) with continuous limiting membrane and reduced electron density; (3) Enlarged lysosomes with ruptured membranes, characterized by visible membrane breaks or discontinuities (red arrows in revised Fig. 5F).

Quantitative analysis of lysosome area distribution revealed that *C9orf72* KO, *Smcr8* KO, and dKO cells harbored significantly larger lysosomes compared to WT cells (revised Fig. 5G). To further characterize lysosomal damage, we performed morphological classification using the three categories defined above. Quantification in revised Fig. 5H shows that knockout cells have a significantly higher proportion of enlarged lysosomes compared to WT, and among these enlarged lysosomes, a significantly higher percentage display membrane ruptures following LLOMe treatment. Lysosomal membrane ruptures, characterized by loss of membrane continuity (red arrows in Fig. 5F), were readily visible in mutant cells, indicating impaired membrane repair capacity. These morphological changes—enlarged lysosomes with increased membrane rupture—provide ultrastructural evidence for defective lysosomal membrane repair in C9orf72/SMCR8-deficient cells.

We have updated the basal condition examples in revised Fig. 5F to ensure that all labeled structures are single-membrane lysosomes, not double-membraned autophagosomes, which can be distinguished by their characteristic double membrane and often contain recognizable cytoplasmic cargo at various stages of degradation.

Fig. 6B and Fig. 7A-D Can lysosomal recruitment of CHMP2B and p-Rab8A be verified by western blot analysis, as shown in Fig. 6A?

Response: Yes, we thank the reviewer for this suggestion. We have performed LysoIP followed by western blot analysis to biochemically verify CHMP2B and pT72-RAB8A recruitment to lysosomes. Knockout cells showed reduced association of both CHMP2B (revised Fig. 6B) and pT72-RAB8A (revised Fig. 7C) with LAMP1-positive lysosomes, consistent with the immunofluorescence observations.

Fig. 7A-D: In addition to p-Rab8A, could the authors also stain for total Rab8A?

Response: We thank the reviewer for this suggestion. We tested multiple RAB8A antibodies for immunofluorescence but were unable to obtain specific signal in mouse microglia. As an alternative, we transfected equal amounts of GFP-RAB8A into WT and knockout primary microglia. Following LLOMe treatment, GFP-RAB8A colocalization with $LAMP1^{+}$ lysosomes was reduced in dKO cells (Fig. R4A, B), while total GFP-RAB8A-positive vesicles were increased in dKO cells (Fig. R4C).

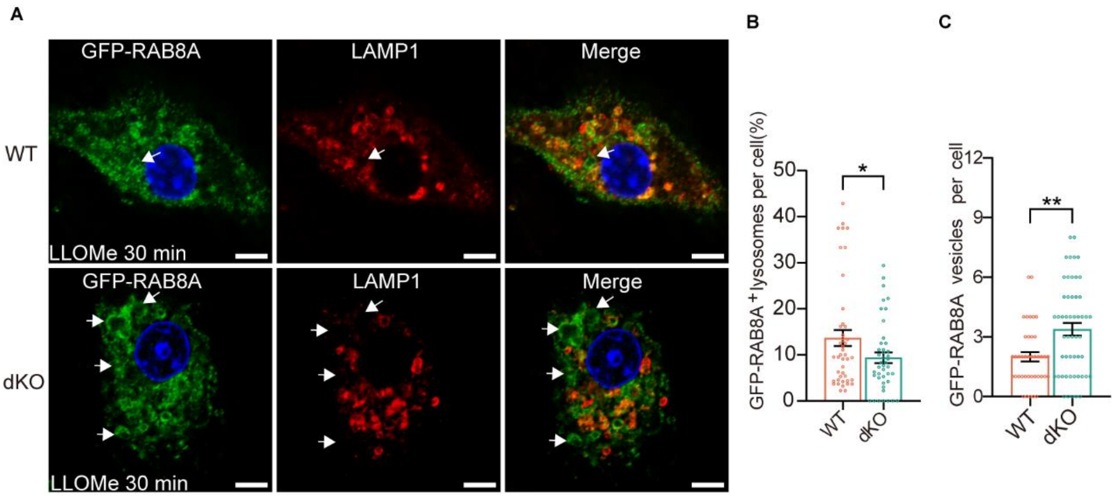

Figure R4. Total RAB8A localization and lysosomal recruitment in microglia. (A) Representative images of WT and dKO primary microglia transfected with GFP-RAB8A (green) and stained for LAMP1 (red) and DAPI (blue) following LLOMe treatment (0.5 mM, 30 min). Arrows indicate RAB8A positive and LAMP1 negative vesicles. Scale bar, 5 μm. (B) Percentage of $GFP\text{-}RAB8A^{+}/LAMP1^{+}$ lysosomes per cell. dKO cells show reduced colocalization (WT: n = 44 cells; dKO: n = 43 cells; from 3 experiments). (C) GFP-RAB8A positive vesicles per cell. dKO cells show increased total vesicles (WT: n = 37 cells; dKO: n = 49 cells; from 3 experiments). Data are mean ± SEM. *P = 0.0456, **P = 0.0014; Student's t test.

Fig. 7E-M: Total Rab8A levels should be quantified and shown in the graphs too.

Response: We thank the reviewer for this suggestion. We have quantified total RAB8A protein levels in BV2 cells (revised Fig. 7I), brain (revised Fig. EV4H), and spinal cord (revised Fig. EV4L) tissues.

Total RAB8A levels were elevated in knockout samples. When pT72-RAB8A signals were normalized to total RAB8A, phosphorylation levels remained significantly increased, confirming elevated phosphorylation status in knockout cells.

The increased total RAB8A may reflect enhanced protein stability of GTP-bound and phosphorylated RAB8A. Previous studies demonstrated that GTP-bound RAB8A is protected from ubiquitin-mediated degradation, while GDP-bound RAB8A is rapidly degraded(Takahashi *et al*, 2023).

Fig. 8A and 8C: both Rab8 and pT72-RAB8A should be shown in both input and IP. And the experiment should be done in both basal and LLOME treated conditions.

Fig. 8F and G: basal conditions should be included as a control.

Response: We have revised Figures 8A, 8C, 8F, and 8G to include both basal and LLOMe-treated conditions. Revised Figure 8A: Co-IP of C9orf72/SMCR8 under basal and LLOMe conditions, showing both total RAB8A and pT72-RAB8A in input and IP. LLOMe treatment enhanced pT72-RAB8A association with the C9orf72/SMCR8 complex compared to basal. Revised Figure 8C: Co-IP showing that GTP-locked RAB8A-Q67L displays enhanced association with C9orf72/SMCR8 under LLOMe treatment compared to basal conditions. Revised Figure 8F: Included basal conditions, revealing a modest increase in GTP-RAB8A levels specifically in *C9orf72* single KO cells compared to WT, while *Smcr8* KO and dKO cells showed no appreciable difference at baseline, with dramatic elevation in all knockout cells upon LLOMe treatment. Revised Figure 8G: Included basal conditions showing that GTP-locked RAB8A-Q67L exhibits higher pT72-RAB8A signal under LLOMe treatment, confirming GTP-RAB8A is the preferred LRRK2 substrate during damage.

Together, these revised experiments demonstrate that: (1) lysosomal damage triggers enhanced pT72-RAB8A association with C9orf72/SMCR8 complex (Fig. 8A), (2) GTP-

loaded RAB8A shows increased binding to C9orf72/SMCR8 upon damage (Fig. 8C), and (3) GTP-locked RAB8A undergoes enhanced phosphorylation during damage (Fig. 8G).

Fig. 9: Could the authors also look at pT72-RAB8A levels and recruitment to lysosome?

Response: We have now examined pT72-RAB8A levels and lysosomal recruitment in the rescue experiments and updated Fig. 9E-H accordingly. Re-expression of WT C9orf72/SMCR8 complex rescued pT72-RAB8A recruitment to lysosomes, while the GAP-deficient mutant (C9-W33A/S8-R147A) failed to do so (Fig. 9E, F). Following LLOMe treatment (10 and 30 min), dKO cells expressing WT complex showed restored pT72-RAB8A colocalization with LAMP1^{+} lysosomes to levels comparable to WT cells (Fig. 9E, F). In contrast, dKO cells expressing the GAP mutant complex showed impaired pT72-RAB8A recruitment, similar to untransfected dKO cells (Fig. 9F).
Additionally, we quantified total pT72-RAB8A puncta per cell (Fig. 9G, H). dKO cells showed elevated total pT72-RAB8A puncta, which were reduced to WT levels by re-expression of the WT complex but not by the GAP-deficient mutant (Fig. 9H). This demonstrates that GAP activity is required not only for pT72-RAB8A recruitment to damaged lysosomes but also for preventing aberrant accumulation of pT72-RAB8A on non-lysosomal compartments. These findings provide direct evidence that the C9orf72/SMCR8 complex GAP activity is essential for proper pT72-RAB8A localization and lysosomal repair, complementing the rescue data for ESCRT recruitment (Fig. 9A-D).

Minor comments:

Introduction "Structural analyses have shown that this complex exhibits GTPase-activating protein (GAP) activity toward multiple RAB GTPases, including RAB8A": Arf GTPases should be mentioned too.

Response: We thank the reviewer for this suggestion. We have revised the Introduction to include ARF GTPases.

Referee #3:

Li and co-workers report on a novel mechanism by which the C9orf72/SMCR8 complex maintains homeostasis of microglia through a Rab8A-ESCRT mediated lysosomal repair. For this they generate a double C9/S8 KO mice wherein they observed age-dependent increased microgliosis and which resulted as well in increased pro-inflammatory responses, both in brain as well as in primary microglia. To dissect the underlying mechanism they combined dKO BV2 and primary microglia and modelled lysosomal damage through LLOME treatments (and washouts) followed by Gal3 stainings. In brief, they found in both cell models that single and dKO resulted in a defective recruitment of ESCRT components, known to be involved in early stages of lysosomal repair. Interestingly, they also demonstrate that in KO microglia, GTP-Rab8a accumulates and fails to be become recruited to damaged lysosomes, and which is parallelled by aberrantly phosphorylated Rab8a in a LRRK2 dependent manner. The data are as well further supported by co-IP and lyso-IP demonstrating the preferential binding to GTP-Rab8a. Overall the authors convincingly demonstrate a dual role for the C9/S8 complex in regulaing Rab8a during lysosomal repair, through its function as a GAP for Rab8a but as well through regulating its phosphorylation status. Although several of these mechanistic insights are confirmed/supported by mouse brain data, translational relevance in ALS/FTD human models is lacking, diminishing a bit the overall enthusiasm. Nevertheless it remains a very relevant and systematic study, experimentally well scrutinized with the necessary controls, and which provides a solid foundation for future validation. Overall the manuscript is suitable for publication in EMBO J, provided the authors address the following experimental points, which are in most cases minor but include as well additional experiments to support the intermittent conclusions with more quantified data.

Comments:

1. Figure 2 shows snRNAseq data that show a 'significant' expansion of the microglial population in dKO mice: however, the graph doesn't show p-values and statistics should

be done to claim significancy. Related, are the microglia the only cell population that shows alterations? A quantitative set for all cell types should be include to claim a more selective effect on microglial populations.

Response: We thank the reviewer for this important comment. In our study, biological replicates were pooled prior to single-cell capture, which was a common practice in early single-cell studies to ensure sufficient cell numbers and reduce sequencing cost. This pooled experimental design (biological replicates pooled for snRNAseq library preparation) precludes formal statistical comparison of cell proportions between groups. We have therefore revised the manuscript to replace "significant" with descriptive language such as "trend" when describing changes in microglial populations. Although formal statistical testing of snRNAseq proportions is not possible with our pooled design, we have validated the microglial expansion through multiple orthogonal approaches:

1. Quantitative IHC: IBA1 staining showed significantly increased microglial numbers in dKO mice (Fig. 1A-D).
2. Flow cytometry validation (new data): To further validate our single-cell findings, we performed independent flow cytometry analysis on brain tissues from 20-month-old WT and dKO mice (n = 3 per group). Since our archived brain tissues were cryopreserved, only nuclear antigens could be reliably detected. We therefore selected SPI1, a nuclear transcription factor identified as upregulated in our snRNAseq data. Flow cytometry analysis confirmed that both the proportion of $SPI1^+$ cells and SPI1 expression levels (mean fluorescence intensity) were significantly increased in dKO mice compared to WT (Fig. R5).
3. Functional validation of DAM phenotype: Primary microglia isolated from dKO mice showed significantly elevated expression of DAM marker genes by qPCR (Fig. 2G), providing independent confirmation of microglial activation.

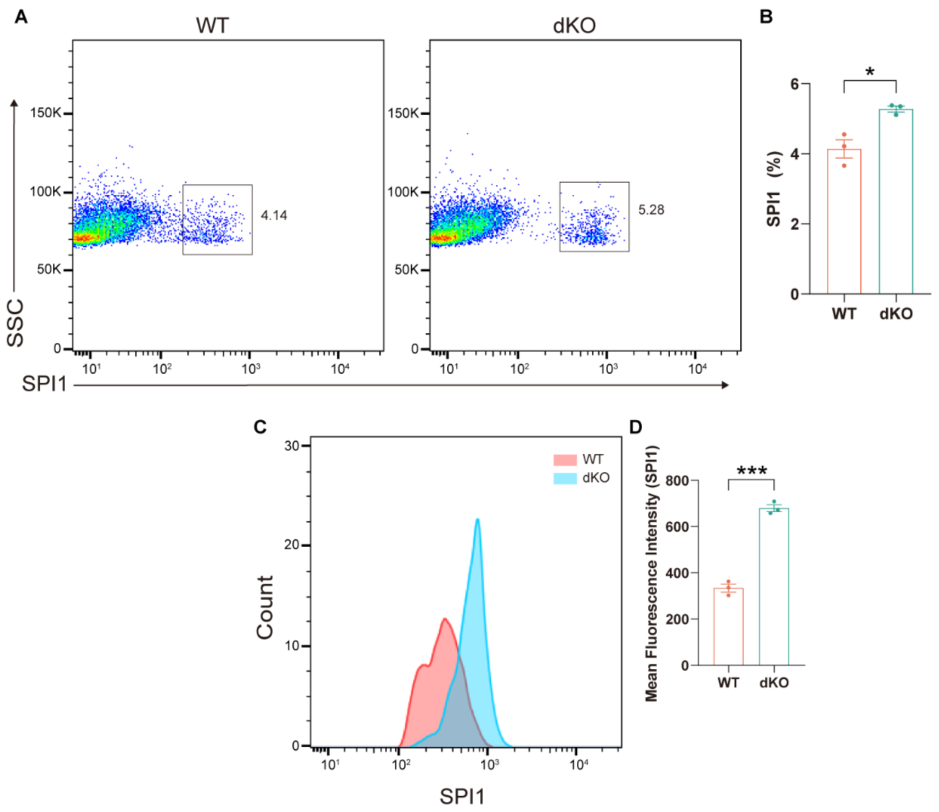

Figure R5. Flow cytometry validation of SPI1$^+$ microglial expansion. (A) Representative flow cytometry scatter plots (SSC vs SPI1) showing gated SPI1$^+$ cell populations in brain nuclei from 20-month-old WT and dKO mice (WT: 4.14%, dKO: 5.28%). (B) Quantification of SPI1$^+$ cell proportion showing significant increase in dKO mice (n = 3 mice per genotype). (C) Histogram overlay showing SPI1 fluorescence intensity distribution in WT (pink) and dKO (blue) SPI1$^+$ cells. dKO shows rightward shift and higher cell counts at elevated intensity, indicating increased SPI1 expression. (D) Mean fluorescence intensity of SPI1 in SPI1$^+$ cells is significantly elevated in dKO mice (n = 3 mice per genotype). Data are mean ± SEM. *P = 0.0142, ***P = 0.0001; Student's t test.

2. Figure 4B: besides dKO cells also single KO cells displayed increased Tnf and IL1b expression. Please correct.

Response: We thank the reviewer for this correction. We have revised the text accordingly.

3. Figure S3B: from this blot it is not clear that C9 and S8 are increased on isolated lysosomes. These experiments should be repeated and quantified. Related, why are the levels of Lamp1 decreased in the lyso-IP of LLOME-treated cells?

Response: We thank the reviewer for this comment. We have repeated the LysoIP

experiments and performed quantification from three independent experiments (revised Fig. EV3D, E). The updated data confirm that both SMCR8 and C9orf72 show increased association with LAMP1-positive lysosomes following LLOMe treatment. Combined with immunofluorescence showing C9orf72 relocalization to damaged lysosomes (Fig. EV3C), these results demonstrate that the C9orf72/SMCR8 complex is recruited to lysosomes upon damage.

Regarding the decreased LAMP1 levels in LysoIP after LLOMe treatment, we speculate that this may result from LLOMe-induced lysosomal exocytosis. Following membrane permeabilization, damaged lysosomes can undergo exocytosis and fuse with the plasma membrane as part of the damage response, effectively reducing the pool of intracellular LAMP1-positive lysosomes available for immunoprecipitation. This would result in lower total LAMP1 recovery in the IP from LLOMe-treated cells despite increased C9orf72/SMCR8 association with the remaining lysosomal pool.

4. The EM data in Figure 5F-G (BV2) and Figure S3F-G (primary microglia) present defective lysosomes only in single KO cells. EM of dKO cells should be included. Also the authors state that in KO cells lysosomes have a 'decreased electron density': this is not evident from these pictures. For instance there is no real difference between WT and C9 KO BV2 cells and S8 KO have even a bit more dense lysosomes. Also the lysosomes in C9 KO primary microglia show more dense accumulations. The authors should provide more representative images or adjust the text to match the observations.

Response: We have now included TEM images of dKO cells in both BV2 (revised Fig. 5F) and primary microglia (revised Fig. EV3I). Regarding electron density under basal conditions, we acknowledge that our original description was inaccurate. We have revised the text to clarify that the lysosomal defects—including altered electron density—are observed specifically upon LLOMe treatment, not under basal conditions. Under basal conditions, lysosomal size and electron density are comparable between genotypes (revised Fig. 5F, EV3I, upper panels). Following LLOMe treatment, knockout cells display distinct lysosomal abnormalities: (1) lysosomal enlargement with increased

area, (2) decreased electron density of luminal contents due to content dilution and leakage, and (3) lysosomal membrane ruptures characterized by membrane discontinuities (red arrows in revised Fig. 5F, EV3I, lower panels). We have quantified these features systematically (Fig. 5G, H for BV2; Fig. EV3J, K for primary microglia) and revised the text to match these observations.

5. Related to Figure 6, the authors have investigated the recruitment of CHMP2B to lysosomes in the different genotypes. They conclude that more CHMP2B puncta are recruited to lysosomes in WT cells in contrast to the reduction in KO cells. However the data are only single images and based on these, one cannot conclude that: even in WT cells most CHMP2B spots are as well adjacent to Lamp1 positive spots. The authors should provide more convincing images and indicate as well co-localized spots.

Response: We thank the reviewer for this comment. We have provided more representative images with clearer visualization of CHMP2B-LAMP1 association (revised Fig. 6C). Following LLOMe treatment, CHMP2B is recruited to lysosomes but displays diverse localization patterns: puncta attached to or adjacent to $LAMP1^{+}$ lysosomes, ring-like structures surrounding lysosomes, or partial colocalization. We observed similar heterogeneous CHMP2B localization patterns in Radulovic et al. (Radulovic *et al*, 2018) and Skowyra et al.(Skowyra *et al*, 2018), where CHMP2B and CHMP4B also showed varied spatial relationships with GAL3 or LAMP1 following LLOMe treatment. This likely reflects the dynamic nature of ESCRT assembly during membrane repair. We have added panels showing CHMP2B puncta formation across multiple cells (revised Fig. 6E, F). These images demonstrate that: LLOMe induces robust CHMP2B puncta in WT cells, and CHMP2B puncta formation is significantly reduced in KO cells (quantified in Fig. 6F).

6. Minor remark on Figure 8E, F: this is not an immunoprecipitation as explained in the text, but a GST pull-down. Please correct.

Response: We thank the reviewer for this correction. The experiment shown in Fig. 8E, F is indeed a GST pull-down, not an immunoprecipitation. We have corrected the text in

both the main text and figure legend accordingly.

7. Finally, the authors use dKO cells to perform rescue experiments with WT C9/S8 or mutant variants. This set of data is limited to a wash-out experiment showing that Gal3-positive lysosomes numbers decreased in WT rescued but not mutant rescued cells. As for the other data sets, the authors should perform the full range of experiments, including the 10 and 30min LLOME followed by washout to get a more integrated idea on the rescue of dKO cells.

Response: We thank the reviewer for this suggestion. In BV2 cells, GAL3 recruitment to damaged lysosomes occurs later than that of pT72-RAB8A and ESCRT components. Specifically, GAL3-positive puncta do not appear until 30 min of LLOMe treatment (Fig. 5C), whereas pT72-RAB8A and ESCRT components are recruited at earlier time points. Therefore, to provide systematic rescue analysis, we performed comprehensive experiments examining multiple time points and readouts in WT, dKO, dKO + WT C9/S8, and dKO + GAP-mutant C9/S8 cells (revised Fig. 9C-H): (1) $GAL3^+$ lysosomes at 30 min LLOMe plus washout 3 and 6 h (Fig. 9C-D), (2) pT72-RAB8A lysosomal recruitment at 10 and 30 min LLOMe (Fig. 9E-F), and (3) total pT72-RAB8A puncta at 10 and 30 min (Fig. 9G-H). These comprehensive rescue data demonstrate that: (1) GAP activity is essential for all aspects of lysosomal repair including GAL3 clearance, pT72-RAB8A recruitment, and prevention of aberrant RAB8A accumulation; (2) re-expression of WT C9orf72/SMCR8 complex fully rescues the lysosomal repair defect in dKO cells; and (3) the GAP-deficient mutant fails to rescue any of these phenotypes, providing strong evidence that GAP activity toward RAB8A is the critical function of the C9orf72/SMCR8 complex in lysosomal membrane repair.

8. Minor comments: for dKO cells, the zoomed inset of the Gal3 staining shows an artifactul red dotted rastering. Please replace and correct this.

Response: We thank the reviewer for pointing this out. We have replaced the image with a corrected version without the artifactual red dotted pattern in the zoomed inset (revised Fig. 9C).

9. The authors must improve the discussion section. The current format merely reads as an extended abstract mainly re-iterating the major (and minor) findings, but hardly putting it in the context of the current literature. For instance, some more in depth discussion on what is known on lysosomal repair mechanisms in neurodegeneration-related models (human neurons, organoids etc) could provide more relevance (eg doi: 10.1038/s41556-025-01623-y using iPSC-derived neurons and tNeurons from familial AD patients). Or how this novel function of C9orf72 can be seen in the light of its other roles in modulating transport and organelle homeostasis? The authors could as well discuss in more depth to what extend other disease-related targets of C9orf72 and LRRK2 can be related to lysosomal repair (Rab10 is such an example, but the paragraph lacks references). The authors make an interesting link to lipid transfer between the ER and lysosomes through MCSs to facilitate lysosome repair: in this respect it is worth mentioning that a Parkinson-related gene, VPS13C, and which mediates lipid transfer between the ER and lysosomes was recently been implicated in lysosomal repair (doi: 10.1038/s41556-025-01653-6): hence the relevance of MCSs in maintaining organellar homeostasis could be highlighted (for instance doi: 10.1038/s41580-025-00864-x, doi: 10.3389/fnins.2022.900338).

Response: We thank the reviewer for this constructive feedback. We have substantially revised and expanded the Discussion to better contextualize our findings within the current literature. Major improvements include:

1. Lysosomal repair in neurodegeneration: Added extensive discussion of lysosomal damage across neurodegenerative diseases, emphasizing human disease models. Incorporated Chou et al (Chou *et al*, 2025) on lysosome repair defects in AD patient neurons, and discussed convergent lysosomal pathology in Niemann-Pick, synucleinopathies, Huntington’s, and Parkinson’s diseases.

2. C9orf72/SMCR8’s broader roles: Added paragraph on the complex as a master coordinator of organellar homeostasis, discussing its GAP activity toward multiple GTPases and regulation of trafficking, autophagy, mTORC1, and ciliogenesis. Incorporated ARF1 data showing context-dependent GTPase engagement.

3. Disease-related targets: Expanded discussion of LRRK2 substrates with proper citations (Dhekne *et al.*, 2018; Eguchi *et al.*, 2018) and now provide the supporting data (Fig. R6) for our previously mentioned observation that RAB10 phosphorylation remained unchanged under basal and lysosomal damage conditions. Distinguished C9orf72/SMCR8 deficiency from PD-LRRK2 mutations (Madero-Pérez *et al.*, 2018; Mamais *et al.*, 2021).

4. Membrane contact sites (MCSs): Substantially expanded MCS discussion incorporating both suggested references. Added: (1) VPS13C-mediated ER-lysosome lipid transfer in repair(Wang *et al*, 2025); (2) MCSs coordinating organellar quality control in neurodegeneration (Calì *et al*, 2025; Vrijsen *et al*, 2022); (3) RAB8A at lipid droplet-mitochondria contacts(Ouyang *et al*, 2023); (4) need for investigating multi-organelle MCSs in disease.

5. Coordinated repair pathways: Added new paragraph on multiple lysosomal repair mechanisms working in concert (ESCRT, lipid transfer, stress granules, lysophagy/exocytosis), with extensive new references (Bussi *et al*, 2023; Domingues *et al*, 2024; Niekamp *et al*, 2022; Radulovic *et al*, 2025; Yim *et al*, 2022).

References:

Bussi C, Mangiarotti A, Vanhille-Campos C, Aylan B, Pellegrino E, Athanasiadi N, Fearns A, Rodgers A, Franzmann TM, Šarić A *et al* (2023) Stress granules plug and stabilize damaged endolysosomal membranes. *Nature* 623: 1062-1069

Calì T, Bayer EM, Eden ER, Hajnóczky G, Kornmann B, Lackner L, Liou J, Reinisch K, Rhee HW,

Rizzuto R *et al* (2025) Key challenges and recommendations for defining organelle membrane contact sites. *Nature reviews Molecular cell biology* 26: 776-796

Chiang CY, Pratuseviciute N, Lin YE, Adhikari A, Yeshaw WM, Flitton C, Sherpa PL, Tonelli F, Rektorova I, Lynch T *et al* (2025) PPM1M, an LRRK2-counteracting, phosphoRab12-preferring phosphatase with a potential link to Parkinson's disease. *Cell Rep* 44: 116031

Chou CC, Vest R, Prado MA, Wilson-Grady J, Paulo JA, Shibuya Y, Moran-Losada P, Lee TT, Luo J, Gygi SP *et al* (2025) Proteostasis and lysosomal repair deficits in transdifferentiated neurons of Alzheimer's disease. *Nature cell biology* 27: 619-632

Dhekne HS, Yanatori I, Gomez RC, Tonelli F, Diez F, Schüle B, Steger M, Alessi DR, Pfeffer SR (2018) A pathway for Parkinson's Disease LRRK2 kinase to block primary cilia and Sonic hedgehog signaling in the brain. *Elife* 7

Domingues N, Catarino S, Cristóvão B, Rodrigues L, Carvalho FA, Sarmento MJ, Zuzarte M, Almeida J, Ribeiro-Rodrigues T, Correia-Rodrigues Â *et al* (2024) Connexin43 promotes exocytosis of damaged lysosomes through actin remodelling. *The EMBO journal* 43: 3627-3649

Dzamko N, Deak M, Hentati F, Reith AD, Prescott AR, Alessi DR, Nichols RJ (2010) Inhibition of LRRK2 kinase activity leads to dephosphorylation of Ser(910)/Ser(935), disruption of 14-3-3 binding and altered cytoplasmic localization. *The Biochemical journal* 430: 405-413

Eguchi T, Kuwahara T, Sakurai M, Komori T, Fujimoto T, Ito G, Yoshimura SI, Harada A, Fukuda M, Koike M *et al* (2018) LRRK2 and its substrate Rab GTPases are sequentially targeted onto stressed lysosomes and maintain their homeostasis. *Proceedings of the National Academy of Sciences of the United States of America* 115: E9115-E9124

Hattula K, Furuhjelm J, Tikkanen J, Tanhuanpää K, Laakkonen P, Peränen J (2006) Characterization of the Rab8-specific membrane traffic route linked to protrusion formation. *Journal of cell science* 119: 4866-4877

Henry R, Shaughnessy L, Loessner MJ, Alberti-Segui C, Higgins DE, Swanson JA (2006) Cytolysin-dependent delay of vacuole maturation in macrophages infected with Listeria monocytogenes. *Cellular microbiology* 8: 107-119

Herbst S, Campbell P, Harvey J, Bernard EM, Papayannopoulos V, Wood NW, Morris HR, Gutierrez MG (2020) LRRK2 activation controls the repair of damaged endomembranes in macrophages. *The EMBO journal* 39: e104494

Kalogeropulou AF, Purlyte E, Tonelli F, Lange SM, Wightman M, Prescott AR, Padmanabhan S, Sammler E, Alessi DR (2022) Impact of 100 LRRK2 variants linked to Parkinson's disease on kinase activity and microtubule binding. *The Biochemical journal* 479: 1759-1783

Kanerva K, Uronen RL, Blom T, Li S, Bittman R, Lappalainen P, Peranen J, Raposo G, Ikonen E (2013) LDL cholesterol recycles to the plasma membrane via a Rab8a-Myosin5b-actin-dependent membrane transport route. *Dev Cell* 27: 249-262

Kania E, Long JS, McEwan DG, Welkenhuyzen K, La Rovere R, Luyten T, Halpin J, Lobbestael E, Baekelandt V, Bultynck G *et al* (2023) LRRK2 phosphorylation status and kinase activity regulate (macro)autophagy in a Rab8a/Rab10-dependent manner. *Cell Death Dis* 14: 436

Madero-Pérez J, Fdez E, Fernández B, Lara Ordóñez AJ, Blanca Ramírez M, Gómez-Suaga P, Waschbüsch D, Lobbestael E, Baekelandt V, Nairn AC *et al* (2018) Parkinson disease-associated mutations in LRRK2 cause centrosomal defects via Rab8a phosphorylation. *Molecular neurodegeneration* 13: 3

Mamais A, Kluss JH, Bonet-Ponce L, Landeck N, Langston RG, Smith N, Beilina A, Kaganovich A,

Ghosh MC, Pellegrini L *et al* (2021) Mutations in LRRK2 linked to Parkinson disease sequester Rab8a to damaged lysosomes and regulate transferrin-mediated iron uptake in microglia. *PLoS Biol* 19: e3001480

Niekamp P, Scharte F, Sokoya T, Vittadello L, Kim Y, Deng Y, Südhoff E, Hilderink A, Imlau M, Clarke CJ *et al* (2022) Ca(2+)-activated sphingomyelin scrambling and turnover mediate ESCRT-independent lysosomal repair. *Nat Commun* 13: 1875

Ouyang Q, Chen Q, Ke S, Ding L, Yang X, Rong P, Feng W, Cao Y, Wang Q, Li M *et al* (2023) Rab8a as a mitochondrial receptor for lipid droplets in skeletal muscle. *Dev Cell* 58: 289-305.e286

Radulovic M, Schink KO, Wenzel EM, Nähse V, Bongiovanni A, Lafont F, Stenmark H (2018) ESCRT-mediated lysosome repair precedes lysophagy and promotes cell survival. *The EMBO journal* 37

Radulovic M, Yang C, Stenmark H (2025) Lysosomal membrane homeostasis and its importance in physiology and disease. *Nature reviews Molecular cell biology*

Sharma M, Giridharan SS, Rahajeng J, Naslavsky N, Caplan S (2009) MICAL-L1 links EHD1 to tubular recycling endosomes and regulates receptor recycling. *Mol Biol Cell* 20: 5181-5194

Skowyra ML, Schlesinger PH, Naismith TV, Hanson PI (2018) Triggered recruitment of ESCRT machinery promotes endolysosomal repair. *Science* 360

Steger M, Tonelli F, Ito G, Davies P, Trost M, Vetter M, Wachter S, Lorentzen E, Duddy G, Wilson S *et al* (2016) Phosphoproteomics reveals that Parkinson's disease kinase LRRK2 regulates a subset of Rab GTPases. *Elife* 5

Takahashi T, Shirai J, Matsuda M, Nakanaga S, Matsushita S, Wakita K, Hayashishita M, Suzuki R, Noguchi A, Yokota N *et al* (2023) Protein quality control machinery supports primary ciliogenesis by eliminating GDP-bound Rab8-family GTPases. *iScience* 26

Vrijsen S, Vrancx C, Del Vecchio M, Swinnen JV, Agostinis P, Winderickx J, Vangheluwe P, Annaert W (2022) Inter-organellar Communication in Parkinson's and Alzheimer's Disease: Looking Beyond Endoplasmic Reticulum-Mitochondria Contact Sites. Volume 16 - 2022

Wang X, Xu P, Bentley-DeSousa A, Hancock-Cerutti W, Cai S, Johnson BT, Tonelli F, Shao L, Talaia G, Alessi DR *et al* (2025) The bridge-like lipid transport protein VPS13C/PARK23 mediates ER-lysosome contacts following lysosome damage. *Nature cell biology* 27: 776-789

Waschbüsch D, Purlyte E, Pal P, McGrath E, Alessi DR, Khan AR (2020) Structural Basis for Rab8a Recruitment of RILPL2 via LRRK2 Phosphorylation of Switch 2. *Structure (London, England : 1993)* 28: 406-417.e406

Yap CC, Digilio L, McMahon LP, Garcia ADR, Winckler B (2018) Degradation of dendritic cargos requires Rab7-dependent transport to somatic lysosomes. *J Cell Biol* 217: 3141-3159

Yim WW, Yamamoto H, Mizushima N (2022) Annexins A1 and A2 are recruited to larger lysosomal injuries independently of ESCRTs to promote repair. *FEBS letters* 596: 991-1003

Yu M, Arshad M, Wang W, Zhao D, Xu L, Zhou L (2018) LRRK2 mediated Rab8a phosphorylation promotes lipid storage. *Lipids in health and disease* 17: 34

Detailed Description of Changes Made in Revised Manuscript

1. Statistical Analysis

In accordance with EMBO Journal guidelines, we have updated all statistical presentations to include the exact sample size (n) for each experimental group/condition, displayed directly on statistical graphs, with both n values and P values clearly stated in figure legends.

2. Figure Modifications and New Experimental Data

Figure 1: Restructured panel 1A by relocating spinal cord staining to Figure 1B and statistical results to panels 1C-D. Added panels 1G (IL-6 mRNA in brain) and 1J (TNF-α in spinal cord) to address Reviewer 2's concerns.

Figure EV1: Added panels G-J analyzing microglial morphology. Panels EV1 K-N present new analysis of neurons in hippocampus and spinal cord across WT, *C9orf72* KO, *Smcr8* KO, and dKO genotypes (addressing Reviewer 2's concerns).

Figure EV2: Completely updated to analyze GAL3 protein levels across four genotypes (panels A-B), with panels E-F and C-D showing GAL3/CD68 double-positive cells with quantitative analysis to address Reviewer 2's questions.

Figure 2: Panel 2B now includes distribution plots for all cell types (addressing Reviewer 3's concerns). Corrected microglial cell numbers in panel 2C.

Figure EV3: Original Figure S2 panels A-C were relocated to current Figure EV3 A-B. Added panel EV3E showing Western blot quantification in panel D (addressing Reviewer 3's concerns).

Figure 5: Panel 5B corrected statistical comparison at washout 30-minute timepoint: changed from ** (WT vs *Smcr*8 KO) to **** (WT vs dKO), as all timepoints in this panel compare WT vs dKO. Figure 5G replaced with more representative images showing clearer lysosomal labeling, with further lysosomal characterization including size quantification (5G) and lysosomal status proportions (5H). Added TEM images of dKO cells in both BV2 (revised Figure 5F) and primary microglia (revised Figure EV3I) to address Reviewers 2 and 3's concerns.

Figure EV4: Panel B replaced with clearer phospho-RAB8A Western blot image. New panels C-D examine CHMP2B and pRAB8A localization at 2 and 5 minutes post-LLOMe treatment (addressing Reviewer 1's concerns). Panels M-O show mass spectrometry-based proteomic analysis of RAB8A-interacting effector proteins in WT and dKO cells under LLOMe treatment, with co-IP validation (addressing Reviewer 1's questions).

Figure EV5: Examines pRAB8A localization beyond ciliogenesis (panel A) with

new panels B-F analyzing early endosomes, lipid droplets, and recycling endosomes (addressing Reviewer 1's concerns).

Figure 6: Added panel 6B showing LysoIP for CHMP2B-lysosome association (addressing Reviewer 2's questions). Panel 6E presents representative confocal images from multiple cells showing CHMP2B puncta formation under basal conditions and LLOMe treatment, with panel 6F providing quantitative analysis of CHMP2B puncta (addressing Reviewer 3's concerns).

Figure 7: Added panel 7C showing pRAB8A LysoIP (addressing Reviewer 2's questions). Panel 7I provides RAB8A protein level quantification (addressing Reviewer 2's concerns). Panels 7J-L analyze pRAB8A localization on RAB7-positive/LAMP1-negative vesicles (addressing Reviewer 1's concerns). Original panels 7H-M were relocated to Figure EV4E-L with added RAB8A protein level statistics (addressing Reviewer 2's concerns).

Figure 8: Panels A, C, F, G now include basal condition results to address Reviewer 2's questions.

Figure 9: Panels C-D added three timepoints (30 min LLOMe, 3 h and 6 h washout) for GAL3 puncta analysis, with WT and dKO cell lines analyzed in the same experimental batch. Panels E-F analyze pRAB8A recruitment to lysosomes in rescue experiments. Panels G-H examine overall pRAB8A puncta formation (addressing Reviewers 2 and 3's concerns). Panel 9I provides comprehensive summary diagram (addressing Reviewer 1's concerns).

3. Data Availability Statement
The snRNA-seq reads were deposited in National Genomics Data Center (https://ngdc.cncb.ac.cn) with the BioProject accession number PRJCA055728. The temporarily shared URL for referees is https://ngdc.cncb.ac.cn/gsa/s/ReWpFSm9. Large image files from Figure 1 and Figure EV1 were uploaded to BioImage Archive.

4. Materials and Methods Updates
We have added animal ethics approval statements, detailed qRT-PCR methodologies, microglial morphology analysis protocols, and co-immunoprecipitation methods.

5. Main Text and Figure Legend Updates
Yellow highlighted sections indicate: updated statistics (n values and P values), new experimental data, revised Discussion (per Reviewer 3's suggestions), and added methods. The revised manuscript with all changes highlighted is provided as a separate file ("Revised manuscript EMBO J – highlight") for reviewers' convenience.

1st Revision - Editorial Decision 20th Apr 2026

Dear Dr. Yang,

Thank you for submitting a revised version of your manuscript. We have now received input from two of the original reviewers, who now find that their main concerns have been addressed satisfactorily and recommend acceptance of the manuscript after a minor revision along the lines indicated by reviewer #2.

There also remain a few editorial and formatting points that need to be addressed before I can extend official acceptance of the manuscript:

1. Please note that it is The EMBO Journal policy for the transcript of the editorial process (containing referee reports and your response letters) to be published as an online supplement to each paper. If you should prefer removal of any referee-only figures included in the point-by-point response(s), e.g. because they may still be used for future publication or because they have been reproduced from published work by others, please do let us know immediately via response email.
More information is available here: https://www.embopress.org/transparent-process#Review_Process
2. We noticed an author name discrepancy between Chen Peng (in the manuscript) and Cheng Peng (in our online system). Please check and correct as needed.
3. Please remove the "Abbreviations" section from the manuscript. Abbreviations should be defined in brackets after their first mention in the text.
4. Please list all corresponding authors in the Author Checklist file.
5. CRediT has replaced the traditional author contributions section because it offers a systematic, machine-readable author contributions format that allows for more effective research assessment. Please remove the Authors Contributions from the manuscript and use the free text boxes beneath each contributing author's name in our online submission system to add specific details on the author's contribution. More information is available in our guide to authors.
6. Please rename "Ethics declarations" section into "Disclosure and competing interests statement".
7. Please remove BioRender disclaimer from figure legends and add to a dedicated section in the Methods section using the following format:
Graphics:
(some of the... OR Figure #... OR synopsis) Graphics were created with BioRender.com.
8. Please rename Table 1-2 into Appendix Table 1-2 and update the nomenclature throughout the manuscript. Please move Appendix Tables 1-2 to the Appendix file. Appendix should be in a pdf format, prefaced by a title page containing a brief table of contents including page numbers.
9. Please add a title "Expanded View Figure Legends" before listing EV figure legends.
10. Source data for Figure 2D and 2F appear to be missing - please check.
11. Our data editors have flagged the following issues in figure legends that need correcting:
- Please note that the legends for the panels in figure 3 are not provided in the sequential manner - i.e., currently panel F is listed before E, and J before I. Please check and correct.
- Please indicate the statistical test used for data analysis in the legends of figures 2E,F.
- Please define the white dotted lines in the legend of figure 1K; EV-1K.
12. Please remove the synopsis text from the legend of figure 9I and update the legend accordingly to reduce its length. I will meanwhile look into the proposed text and will let you know if any edits to the journal's style are needed. The synopsis image can be used both in the figure 9I as a summary figure as an individual synopsis image with a 550x300 pixels format (height x width). I have attached two size-adjusted versions that I have generated based on the figure 9I - please check which one you prefer. The smaller section has better readability but lacks the more general information about the physiological impact.

Please let me know if you have any questions regarding any of these points. You can use the link below to upload the revised files.

Thank you again for giving us the chance to consider your manuscript for The EMBO Journal. I look forward to receiving the final version.

With best wishes,

Ieva

Ieva Gailite, PhD
Senior Scientific Editor
The EMBO Journal
Meyerhofstrasse 1
D-69117 Heidelberg
Tel: +4962218891309
i.gailite@embojournal.org

We realize that it is difficult to revise to a specific deadline. In the interest of protecting the conceptual advance provided by the work, we recommend a revision within 3 months (19th Jul 2026). Please discuss the revision progress ahead of this time with the editor if you require more time to complete the revisions. Use the link below to submit your revision:

https://emboj.msubmit.net/cgi-bin/main.plex

Referee #1:

The authors have successfully addressed the points I raised, and I am happy to recommend publication of this revised manuscript,

Referee #2:

The authors have addressed most of my concerns during the revision and the manuscript is much improved. I have a few minor comments:
It is interesting that DKO has much stronger phenotypes than single KO. Since SMCR8 is thought to possess GAP activities in the C9ORF72/SMCR8 complex, this is surprising. The data would suggest that C9orf72 and SMCR8 might have other functions besides functioning as the C9orf72/SMCR8 GAP complex. Maybe the authors can add some speculations in the discussion?
Fig. 2B: Is there an increase in the number of oligodendrocytes in the DKO?
Fig. R3: If the increase in LRRK2 activities is repeatable, it should be quantified and included in the manuscript.
Fig. 5F: Normal lysosomes should be labelled on the image as well.

Referee #1:

The authors have successfully addressed the points I raised, and I am happy to recommend publication of this revised manuscript.

Referee #2:

The authors have addressed most of my concerns during the revision and the manuscript is much improved. I have a few minor comments:
It is interesting that DKO has much stronger phenotypes than single KO. Since SMCR8 is thought to possess GAP activities in the C9ORF72/SMCR8 complex, this is surprising. The data would suggest that C9orf72 and SMCR8 might have other functions besides functioning as the C9orf72/SMCR8 GAP complex. Maybe the authors can add some speculations in the discussion?
Fig. 2B: Is there an increase in the number of oligodendrocytes in the DKO?
Fig. R3: If the increase in LRRK2 activities is repeatable, it should be quantified and included in the manuscript.
Fig. 5F: Normal lysosomes should be labelled on the image as well.

Point-by-point responses to the comments raised by the reviewers

Referee #1:

The authors have successfully addressed the points I raised, and I am happy to recommend publication of this revised manuscript.

Response: We sincerely thank Referee #1 for the careful review of our manuscript and for recommending publication.

Referee #2:

We thank Referee #2 for the thorough re-evaluation of our revised manuscript and for the constructive minor comments. We have carefully addressed each point as detailed below.

1. It is interesting that DKO has much stronger phenotypes than single KO. Since SMCR8 is thought to possess GAP activities in the C9ORF72/SMCR8 complex, this is surprising. The data would suggest that C9orf72 and SMCR8 might have other functions besides functioning as the C9orf72/SMCR8 GAP complex. Maybe the authors can add some speculations in the discussion?

Response: We thank the reviewer for this insightful observation. We agree that the substantially more severe phenotype of dKO mice compared to either single knockout

raises the intriguing possibility that C9orf72 and SMCR8 may possess functions beyond their shared role in the GAP complex. We have added a discussion of this possibility on page 28 (paragraph 5) of the revised manuscript:

"*Beyond their shared activities within the complex, C9orf72 and SMCR8 may also possess GAP complex-independent functions, which could help explain why dKO mice exhibit substantially more severe in vivo phenotypes than either single knockout.*"

Direct dissection of these GAP complex-independent activities will be an important focus of future investigations.

Fig. 2B: Is there an increase in the number of oligodendrocytes in the DKO?

Response: We thank the reviewer for this interesting question. Our snRNA-seq dataset showed an increase in both the proportion (16.3% vs. 21.41%) and absolute count (2,723 vs. 3,543) of oligodendrocytes in dKO compared to WT mice. Several genes potentially associated with disease-associated oligodendrocyte (DOL) states, such as *Klk6*, *Ifitm3*, and *H2-Aa*, were upregulated in dKO oligodendrocytes, in line with reports that DOL populations expand in multiple neurodegenerative disease contexts (Falcão et al., 2018; Kenigsbuch et al., 2022; Kaya et al., 2022; Pandey et al., 2022). Oligodendrocyte dysfunction has also been documented in C9orf72-related disease, including TDP-43-positive glial inclusions and decreased myelin gene expression in C9-ALS/FTD patient cortex (Lorente Pons et al., 2020; Sirisi et al., 2022). Detailed characterization of oligodendrocyte states will be an important direction for future studies.
In the present study, we focused on microglia because (i) C9orf72 and SMCR8 are predominantly expressed in microglia, as shown by published single-cell RNA-seq data (Yao et al., 2021) and our immunoblotting in primary neurons and microglia (Fig. EV1); and (ii) consistent microglial alterations were observed across multiple approaches: histological analyses of aged dKO brain and spinal cord revealed microgliosis, GAL3 upregulation, and lysosomal damage in microglia (Figs. 1, 3); and qRT-PCR of primary microglia isolated from dKO mice confirmed upregulation of DAM-associated markers (Fig. 2). Together, these convergent findings provided a clear cell-type-specific entry point for mechanistic investigation.

References:
Falcão AM, van Bruggen D, Marques S, et al. (2018). Disease-specific oligodendrocyte lineage cells arise in multiple sclerosis. *Nature Medicine* 24, 1837-1844.
Kenigsbuch M, Bost P, Halevi S, et al. (2022). A shared disease-associated oligodendrocyte signature among multiple CNS pathologies. *Nature Neuroscience* 25, 876-886.
Kaya T, Mattugini N, Liu L, et al. (2022). $CD8^+$ T cells induce interferon-responsive oligodendrocytes and microglia in white matter aging. *Nature Neuroscience* 25, 1446-1457.

Pandey S, Shen K, Lee SH, et al. (2022). Disease-associated oligodendrocyte responses across neurodegenerative diseases. *Cell Reports* 40, 111189.
Lorente Pons A, Higginbottom A, Cooper-Knock J, et al. (2020). Oligodendrocyte pathology exceeds axonal pathology in white matter in human amyotrophic lateral sclerosis. *Journal of Pathology* 251, 262-271.
Sirisi S, Querol-Vilaseca M, Dols-Icardo O, et al. (2022). Myelin loss in C9orf72 hexanucleotide expansion carriers. *Journal of Neuroscience Research* 100, 1862-1875.
Yao Z, van Velthoven CTJ, Nguyen TN, et al. (2021). A taxonomy of transcriptomic cell types across the isocortex and hippocampal formation. *Cell* 184, 3222-3241.

Fig. R3: If the increase in LRRK2 activities is repeatable, it should be quantified and included in the manuscript.

Response: We thank the reviewer for this important suggestion. We have repeated the experiment with additional biological replicates and confirmed the reproducibility of the increase in LRRK2 activity in dKO cells. The representative immunoblot and quantification of pS935-LRRK2 normalized to total LRRK2 are now included as new panels (K) and (L) in Figure 8 of the revised manuscript. Statistical analysis confirmed significant differences between WT and dKO cells at both time points (LLOMe 30 min: $P = 0.0279$; Washout 3 h: $P = 0.0486$; n = 3 independent experiments).

Fig. 5F: Normal lysosomes should be labelled on the image as well.

Response: We thank the reviewer for this suggestion. In Figure 5F, intact lysosomes are present and indicated by yellow arrowheads in the basal condition panels. The LLOMe-treated panels were specifically selected to show representative fields where ruptured lysosomes are most clearly visible; intact lysosomes are not present in these selected fields, and the yellow arrowheads in these panels indicate damaged lysosomes. Red arrowheads indicate sites of membrane discontinuity. We have clarified these points in the revised figure legend (page 54) and the Results section (page 14).

Dear Mei,

Thank you for incorporating the final editorial and formatting requests in the manuscript. I am now pleased to inform you that your manuscript has been accepted for publication. Congratulations with a nice study!

Before we forward your manuscript to our publishers, we would like to propose some edits in the manuscript abstract and synopsis. Furthermore, we would like to suggest a somewhat shorter and snappier title for your study, and have written a short blurb that will accompany the title of your manuscript in our online table of contents. Please take a look at the proposed textual changes in the attached text file and let me know if any corrections are needed.

Please note that it is The EMBO Journal policy for the transcript of the editorial process (containing referee reports and your response letters) to be published as an online supplement to each paper. If you should prefer removal of any referee-only figures included in the point-by-point response(s), e.g. because they may still be used for future publication or because they have been reproduced from published work by others, please do let us know immediately via response email.
More information is available here: https://link.springer.com/partners/embo-press/editorial-policies#Peer%20review

Once we have your input on textual edits, we will transfer your manuscript for typesetting. Your manuscript will be processed for publication by EMBO Press. It will be copy edited and you will receive page proofs prior to publication. Please note that you will be contacted by Springer Nature Author Services to complete licensing and payment information.

You may qualify for financial assistance for your publication charges - either via a Springer Nature fully open access agreement or an EMBO initiative. Check your eligibility: https://link.springer.com/journal/44318/how-to-publish-with-us

Should you be planning a Press Release on your article, please get in contact with embo_production@springernature.com as early as possible in order to coordinate publication and release dates.

If you have any questions, please do not hesitate to contact the Editorial Office. Thank you for this interesting contribution to The EMBO Journal!

Best wishes,

Ieva

Ieva Gailite, PhD
Senior Scientific Editor
The EMBO Journal
Meyerhofstrasse 1
D-69117 Heidelberg
Tel: +4962218891309
i.gailite@embojournal.org

** Click here to be directed to your login page: https://emboj.msubmit.net

EMBO Press Author Checklist

Corresponding Author Name: Mei Yang and Yonghua Wang	
Journal Submitted to: The EMBO Journal	
Manuscript Number: EMBOJ-2025-122231	

USEFUL LINKS FOR COMPLETING THIS FORM
The EMBO Journal - Author Guidelines
EMBO Reports - Author Guidelines
Molecular Systems Biology - Author Guidelines
EMBO Molecular Medicine - Author Guidelines

Reporting Checklist for Life Science Articles (updated January 2022)
This checklist is adapted from Materials Design Analysis Reporting (MDAR) Checklist for Authors. MDAR establishes a minimum set of requirements in transparent reporting in the life sciences (see Statement of Task: 10.31222/osf.io/9sm4x). Please follow the journal's guidelines in preparing your manuscript.
Please note that a copy of this checklist will be published alongside your article.

Abridged guidelines for figures
1. Data
The data shown in figures should satisfy the following conditions:
- the data were obtained and processed according to the field's best practice and are presented to reflect the results of the experiments in an accurate and unbiased manner.
- ideally, figure panels should include only measurements that are directly comparable to each other and obtained with the same assay.
- plots include clearly labeled error bars for independent experiments and sample sizes. Unless justified, error bars should not be shown for technical
- if n<5, the individual data points from each experiment should be plotted. Any statistical test employed should be justified.
- Source Data should be included to report the data underlying figures according to the guidelines set out in the authorship guidelines on Data Presentation.

2. Captions
Each figure caption should contain the following information, for each panel where they are relevant:
- a specification of the experimental system investigated (eg cell line, species name).
- the assay(s) and method(s) used to carry out the reported observations and measurements.
- an explicit mention of the biological and chemical entity(ies) that are being measured.
- an explicit mention of the biological and chemical entity(ies) that are altered/varied/perturbed in a controlled manner.
- the exact sample size (n) for each experimental group/condition, given as a number, not a range;
- a description of the sample collection allowing the reader to understand whether the samples represent technical or biological replicates (including how many animals, litters, cultures, etc.).
- a statement of how many times the experiment shown was independently replicated in the laboratory.
- definitions of statistical methods and measures:
  - common tests, such as t-test (please specify whether paired vs. unpaired), simple χ2 tests, Wilcoxon and Mann-Whitney tests, can be unambiguously identified by name only, but more complex techniques should be described in the methods section;
  - are tests one-sided or two-sided?
  - are there adjustments for multiple comparisons?
  - exact statistical test results, e.g., P values = x but not P values < x;
  - definition of 'center values' as median or average;
  - definition of error bars as s.d. or s.e.m.

Please complete ALL of the questions below.
Select "Not Applicable" only when the requested information is not relevant for your study.

Materials

Newly Created Materials	Information included in the manuscript?	In which section is the information available? (Reagents and Tools Table, Materials and Methods, Figures, Data Availability Section)
New materials and reagents need to be available; do any restrictions apply?	Not Applicable	

Antibodies	Information included in the manuscript?	In which section is the information available? (Reagents and Tools Table, Materials and Methods, Figures, Data Availability Section)
For **antibodies** provide the following information:<br>- Commercial antibodies: RRID (if possible) or supplier name, catalogue number and or/clone number<br>- Non-commercial: RRID or citation	Yes	Reagents and Tools Table

DNA and RNA sequences	Information included in the manuscript?	In which section is the information available? (Reagents and Tools Table, Materials and Methods, Figures, Data Availability Section)
**Short novel DNA or RNA including primers, probes**: provide the sequences.	Yes	Reagents and Tools Table

Cell materials	Information included in the manuscript?	In which section is the information available? (Reagents and Tools Table, Materials and Methods, Figures, Data Availability Section)
**Cell lines:** Provide species information, strain. Provide accession number in repository **OR** supplier name, catalog number, clone number, and/**OR** RRID.	Yes	Reagents and Tools Table
**Primary cultures:** Provide species, strain, sex of origin, genetic modification status.	Yes	Reagents and Tools Table and Materials and Methods
Report if the cell lines were recently **authenticated** (e.g., by STR profiling) and tested for mycoplasma contamination.	Not Applicable	

Experimental animals	Information included in the manuscript?	In which section is the information available? (Reagents and Tools Table, Materials and Methods, Figures, Data Availability Section)
**Laboratory animals or Model organisms:** Provide species, strain, sex, age, genetic modification status. Provide accession number in repository **OR** supplier name, catalog number, clone number, **OR** RRID.	Yes	Materials and Methods
**Animal observed in or captured from the field:** Provide species, sex, and age where possible**.**	Not Applicable	
Please detail **housing and husbandry conditions**.	Yes	Materials and Methods

Plants and microbes	Information included in the manuscript?	In which section is the information available? (Reagents and Tools Table, Materials and Methods, Figures, Data Availability Section)
**Plants:** provide species and strain, ecotype and cultivar where relevant, unique accession number if available, and source (including location for collected wild specimens).	Not Applicable	
**Microbes:** provide species and strain, unique accession number if available, and source.	Not Applicable	

Human research participants	Information included in the manuscript?	In which section is the information available? (Reagents and Tools Table, Materials and Methods, Figures, Data Availability Section)
If collected and within the bounds of privacy constraints report on age, sex and gender or ethnicity for all study participants.	Not Applicable	

Core facilities	Information included in the manuscript?	In which section is the information available? (Reagents and Tools Table, Materials and Methods, Figures, Data Availability Section)
If your work benefited from core facilities, was their service mentioned in the acknowledgments section?	Yes	Achnowledgements

Design

Study protocol	Information included in the manuscript?	In which section is the information available? (Reagents and Tools Table, Materials and Methods, Figures, Data Availability Section)

If study protocol has been **pre-registered, provide DOI in the manuscript**. For clinical trials, provide the trial registration number **OR** cite DOI.	Not Applicable	
Report the **clinical trial registration number** (at ClinicalTrials.gov or equivalent), where applicable.	Not Applicable	

Laboratory protocol	Information included in the manuscript?	In which section is the information available? (Reagents and Tools Table, Materials and Methods, Figures, Data Availability Section)
Provide DOI OR other citation details if **external detailed step-by-step protocols** are available.	Not Applicable	

Experimental study design and statistics	Information included in the manuscript?	In which section is the information available? (Reagents and Tools Table, Materials and Methods, Figures, Data Availability Section)
Include a statement about **sample size** estimate even if no statistical methods were used.	Not Applicable	
Were any steps taken to minimize the effects of subjective bias when allocating animals/samples to treatment (e.g. **randomization procedure**)? If yes, have they been described?	Yes	Materials and Methods and Figure Legends
Include a statement about **blinding** even if no blinding was done.	Not Applicable	
Describe **inclusion/exclusion criteria** if samples or animals were excluded from the analysis. Were the criteria pre-established?<br><br>If sample or data points were omitted from analysis, report if this was due to attrition or intentional exclusion and provide justification.	Not Applicable	
For every figure, are **statistical tests** justified as appropriate? Do the data meet the assumptions of the tests (e.g., normal distribution)? Describe any methods used to assess it. Is there an estimate of variation within each group of data? Is the variance similar between the groups that are being statistically compared?	Yes	Materials and Methods (Statistical analysis) and Figure Legends

Sample definition and in-laboratory replication	Information included in the manuscript?	In which section is the information available? (Reagents and Tools Table, Materials and Methods, Figures, Data Availability Section)
In the figure legends: state number of times the experiment was **replicated** in laboratory.	Yes	Figure legends
In the figure legends: define whether data describe **technical or biological replicates**.	Yes	Figure legends

Ethics

Ethics	Information included in the manuscript?	In which section is the information available? (Reagents and Tools Table, Materials and Methods, Figures, Data Availability Section)
Studies involving **human participants**: State details of **authority granting ethics approval** (IRB or equivalent committee(s), provide reference number for approval.	Not Applicable	
Studies involving **human participants**: Include a statement confirming that **informed consent** was obtained from all subjects and that the experiments conformed to the principles set out in the WMA Declaration of Helsinki and the Department of Health and Human Services Belmont Report.	Not Applicable	
Studies involving **human participants: For publication of patient photos**, include a statement confirming that consent to publish was obtained.	Not Applicable	
Studies involving experimental **animals**: State details of **authority granting ethics approval** (IRB or equivalent committee(s), provide reference number for approval. Include a statement of compliance with ethical regulations.	Yes	Materials and Methods
Studies involving **specimen and field samples:** State if relevant **permits** obtained, provide details of authority approving study; if none were required, explain why.	Yes	Materials and Methods

Dual Use Research of Concern (DURC)	Information included in the manuscript?	In which section is the information available? (Reagents and Tools Table, Materials and Methods, Figures, Data Availability Section)
Could your study fall under dual use research restrictions? Please check biosecurity documents and list of **select agents and toxins** (CDC): https://www.selectagents.gov/sat/list.htm	Not Applicable	
If you used a select agent, is the security level of the lab appropriate and reported in the manuscript?	Not Applicable	
If a study is subject to dual use research of concern regulations, is the name of the **authority granting approval and reference number** for the regulatory approval provided in the manuscript?	Not Applicable	

Reporting

The MDAR framework recommends adoption of discipline-specific guidelines, established and endorsed through community initiatives. Journals have their own policy about requiring specific guidelines and recommendations to complement MDAR.

Adherence to community standards	Information included in the manuscript?	In which section is the information available? (Reagents and Tools Table, Materials and Methods, Figures, Data Availability Section)
State if relevant guidelines or checklists (e.g., **ICMJE, MIBBI, ARRIVE, PRISMA**) have been followed or provided.	Not Applicable	
For **tumor marker prognostic studies,** we recommend that you follow the **REMARK** reporting guidelines (see link list at top right). See author guidelines, under 'Reporting Guidelines'. Please confirm you have followed these guidelines.	Not Applicable	
For **phase II and III randomized controlled trials**, please refer to the **CONSORT** flow diagram (see link list at top right) and submit the CONSORT checklist (see link list at top right) with your submission. See author guidelines, under 'Reporting Guidelines'. Please confirm you have submitted this list.	Not Applicable	

Data Availability

Data availability	Information included in the manuscript?	In which section is the information available? (Reagents and Tools Table, Materials and Methods, Figures, Data Availability Section)
Have **primary datasets** been deposited according to the journal's guidelines (see 'Data Deposition' section) and the respective accession numbers provided in the Data Availability Section?	Yes	Materials and Methods
Were **human clinical and genomic datasets** deposited in a public access-controlled repository in accordance to ethical obligations to the patients and to the applicable consent agreement?	Not Applicable	
Are **computational models** that are central and integral to a study available without restrictions in a machine-readable form? Were the relevant accession numbers or links provided?	Not Applicable	
If publicly available data were reused, provide the respective **data citations in the reference list**.	Yes	References
